# Vehicle Sideslip Angle Estimation Using Deep Reinforcement Learning Combined with Unscented Kalman Filter

**DOI:** 10.3390/s25247489

**Published:** 2025-12-09

**Authors:** Liguang Wu, Wei Wang, Penghui Li, Yueying Zhu

**Affiliations:** 1 School of Mechanical Engineering, Tianjin University, Tianjin 300192, China; 2 China Automotive Technology and Research Center Co., Ltd., Tianjin 300300, China; 3 School of Mechanical Engineering, Tianjin University of Science and Technology, Tianjin 300457, China

**Keywords:** vehicle sideslip angle, unscented kalman filter, deep reinforcement learning, state estimation, vehicle dynamics

## Abstract

The vehicle sideslip angle is a core state parameter in vehicle dynamics control. Its accurate estimation is critical for vehicle stability control and the development of active safety systems. In the vehicle sideslip angle estimation method using the traditional Unscented Kalman Filter (UKF), the process noise covariance matrix Q and observation noise covariance matrix R are difficult to adjust adaptively, leading to estimation accuracy degradation under complex driving conditions. This paper proposes a vehicle sideslip angle estimation method that integrates UKF and Deep Reinforcement Learning (DRL), leveraging the adaptive decision-making capability of DRL to dynamically optimize the noise parameters in UKF. A state space incorporating vehicle motion states and filtering performance metrics is constructed, along with an action space that outputs adjustment quantities for the noise covariance matrices. A reward function based on estimation errors and uncertainties is formulated, and the Proximal Policy Optimization (PPO) algorithm is employed to train the policy network. The results indicate that the proposed method effectively improves vehicle sideslip angle estimation accuracy under various driving conditions, including different vehicle speeds, road surface adhesion coefficients, and sensor noise disturbances. Compared with the traditional UKF method, the Root Mean Square Error (RMSE) is reduced by over 30%, and the method demonstrates strong stability and robustness under complex scenarios. This approach provides a new solution for the accurate estimation of key vehicle state parameters and can be extended to fields such as autonomous driving and vehicle active safety.

## 1. Introduction

### 1.1. Research Background

The vehicle sideslip angle is a core parameter characterizing a vehicle’s lateral dynamic state. It directly determines a vehicle’s steering response, driving stability, and handling limits, and serves as a critical input for the Electronic Stability Program (ESP), Electric Power Steering (EPS) system, and lateral control strategies in advanced autonomous driving [1]. However, this parameter cannot be directly measured by low-cost on-board sensors (e.g., wheel speed sensors, steering angle sensors). Specialized equipment, such as high-precision Inertial Measurement Units (IMUs), cannot easily meet the engineering application requirements of mass-produced vehicles due to their high cost. Therefore, indirect estimation via estimation algorithms is required, making it one of the core technical challenges in the field of vehicle dynamics control [2].

Among traditional vehicle sideslip angle estimation methods, the Extended Kalman Filter (EKF) can handle nonlinear systems. However, under extreme vehicle operating conditions (e.g., emergency avoidance on low-adhesion roads, high-speed steering), the linearization approximation that it applies to nonlinear models tends to introduce significant errors, leading to a marked decline in estimation accuracy [3]. The Unscented Kalman Filter (UKF) avoids linearization errors through the unscented transformation, which improves its adaptability to nonlinear scenarios. Nevertheless, when used independently, it is still limited by parameter perturbations in the vehicle dynamics model (e.g., vehicle weight changes, suspension stiffness degradation) and external disturbances (e.g., crosswinds, sudden changes in road adhesion coefficient). Its insufficient robustness makes it difficult to meet the estimation requirements in complex driving scenarios [4].

As autonomous driving technology advances toward Level 4 (L4) and higher levels, vehicles impose higher requirements on the real-time performance, accuracy, and anti-interference capability of lateral state estimation. Single filtering algorithms or model-driven methods can no longer cover the demands of full operating conditions [5]. DRL possesses the ability to optimize decisions through autonomous learning in nonlinear and uncertain systems, and can adaptively compensate for model errors and external disturbances—providing a new approach to enhance the robustness of estimation algorithms [6]. Therefore, combining the nonlinear state estimation capability of UKF with the adaptive anti-interference advantages of DRL to construct a fused estimation method has become an important research direction for solving high-precision estimation of the sideslip angle under complex operating conditions [7].

### 1.2. Research Status and Limitations

The vehicle sideslip angle is a core parameter for evaluating vehicle lateral stability. Among early measurement instrument methods, the inertial sensor solution obtains lateral velocity by integrating lateral acceleration, but it suffers from noise accumulation, leading to a significant decline in long-term accuracy [8]. While the GPS integration solution enables dynamic correction and delivers stable output under neutral steering conditions, it is sensitive to sampling efficiency and environmental occlusion [9]. By constructing dynamic models with different degrees of freedom and combining them with nonlinear state observers or EKF algorithms, researchers have achieved stable estimation under extreme operating conditions—among these, the application of nonlinear tire models has significantly improved estimation accuracy [10].

In terms of the development of estimation algorithms, the direct integration method features low computational complexity and good real-time performance, but error accumulation results in large long-term estimation deviations [11]. The EKF processes nonlinear systems through linearization; it performs stably under small sideslip angle conditions, and its accuracy can meet basic control requirements [12]. The UKF avoids linearization errors via the unscented transformation, and its accuracy is superior to that of the EKF [13]. Sliding mode observers and Luenberger observers exhibit stronger adaptability to load transfer and road condition changes, with the estimation results of second-order sliding mode observers and generalized Luenberger observers being closer to true values [14].

Since the rise of data-driven technologies, methods such as neural networks and fuzzy logic have been gradually applied to vehicle sideslip angle estimation. Researchers have integrated the advantages of various methods by constructing fused estimators, which have improved the accuracy and reliability of estimation [15]; the piecewise affine estimation method that accounts for the nonlinear effect of wheel lateral force saturation has been verified to have high feasibility through experiments [16]. Comparative studies show that dynamic model-based estimation is suitable for steady-state operating conditions, while kinematic model-based estimation offers better dynamic response [17]. The fusion of physical models and data-driven approaches has become a trend—combinations such as fuzzy logic with UKF and ANFIS (Adaptive Neuro–Fuzzy Inference System) estimates with UKF have all achieved effective estimation of relevant parameters [18].

The UKF is widely used in vehicle state estimation. It approximates the probability distribution of nonlinear systems through the unscented transformation, avoiding the inherent errors of the EKF [19]. The UKF observer based on a 2-degree-of-freedom (2-DOF) model has shown a significant accuracy improvement compared to the EKF in real-vehicle tests [20]; by adopting a 7-degree-of-freedom (7-DOF) full-vehicle model and fusing multi-sensor signals, the error is greatly reduced, and the convergence speed is significantly accelerated under high-speed sharp turning conditions [21]. The robust adaptive UKF algorithm based on a fault detection mechanism exhibits excellent accuracy and robustness in sinusoidal steering conditions with alternating high and low adhesion coefficients [22].

To address the issues of fixed noise covariance and poor adaptability to time-varying parameters in traditional UKF, researchers have conducted a series of improvement studies. The adaptive singular value decomposition UKF (SVD-UKF) reduces errors significantly under complex road conditions by real-time correcting the noise covariance matrix, enabling it to handle sensor noise fluctuations [23]; the combination of fuzzy control and UKF realizes adaptive adjustment of measurement noise, leading to improved accuracy under double-lane-change conditions [24]. The fusion framework of a derivative fault-tolerant noise estimator and UKF has achieved the joint estimation of the vehicle sideslip angle and tire cornering stiffness [25].

DRL fits value functions or policies through deep neural networks and has the potential to handle high-dimensional nonlinear systems. In recent years, its application in the field of vehicle dynamics has expanded from control decision-making to state estimation [26]. However, its application in vehicle state estimation is still in the initial stage, mainly focusing on observer parameter optimization and data-driven modeling [27]. The model that fuses multi-sensor time series data based on a CNN-LSTM network and optimizes the weight update strategy via DRL solves the problem of insufficient generalization of traditional neural networks, with controllable error growth under out-of-training-set conditions [28]. Some studies use DRL as a model error compensator to learn error patterns based on the output of dynamic models, which significantly reduces estimation deviations in double-lane-change tests [29]; the “DRL + fuzzy sliding mode observer” architecture optimizes saturation function parameters, which not only suppresses chattering but also accelerates convergence speed and reduces response lag in the tire nonlinear region [30]. The combination of DRL and EKF improves estimation stability under conditions with sudden changes in sensor signals by dynamically adjusting filter gains [31].

Overall, the estimation of vehicle sideslip angle has formed a technical system dominated by traditional model-based filtering and supplemented by data-driven methods. However, gaps remain in adaptability to complex conditions and the balance between real-time performance and robustness [32]. Different methods have their own advantages and disadvantages: the EKF is computationally simple but only applicable to linear or small sideslip angle conditions; the UKF offers high accuracy in nonlinear processing and performs best in sinusoidal maneuvers on high-adhesion roads; the adaptive UKF has strong anti-noise capability but increases computational time; DRL shows potential in extreme conditions but has poor real-time performance; and sliding mode observers have strong robustness but their accuracy is limited by the design of switching functions.

Core technical bottlenecks are reflected in three aspects: model-based methods rely on accurate models and noise statistical characteristics, leading to a significant drop in accuracy when vehicle parameters change or road conditions suddenly shift [33]; data-driven methods require massive labeled data and lack physical constraints, making them prone to generating non-physical results [34]; and fusion strategies are mostly simple combinations, which tend to cause estimation jumps during condition transitions and fail to achieve deep coupling [35].

Current research has two major gaps: the UKF lacks sufficient parameter adaptability and struggles to cope with full operating condition changes; DRL in the estimation field lacks organic integration with physical models, making it difficult to balance real-time performance and generalization. To address these issues, this study proposes a “UKF + DRL” fusion framework. By using DRL to optimize noise parameters and physical models to constrain estimation boundaries, the framework achieves high-accuracy and high-robustness estimation under full operating conditions, filling the application gap in extreme conditions and scenarios with time-varying parameters.

The comparison of all vehicle sideslip angle estimation methods is presented in Table 1.

### 1.3. Research Content and Research Contributions

Taking the high-precision estimation of vehicle sideslip angle under full operating conditions as the core goal, this study constructs a fused estimation system of “physical model constraint-data-driven optimization” to address relevant technical bottlenecks. First, based on the concept of “dual closed-loop collaboration,” the system designs a fusion architecture of UKF and DRL. The outer loop builds a UKF observer incorporating tire lateral force saturation characteristics based on an 8-degree-of-freedom (8-DOF) vehicle dynamics model, using signals from standard sensors (e.g., wheel speed, lateral acceleration) to achieve initial state estimation. The inner loop introduces a DRL algorithm with an Actor–Critic framework, which takes estimation error, the trace of the noise covariance matrix, and physical constraint deviation as state inputs to dynamically optimize the process noise covariance Q and observation noise covariance R of UKF. Meanwhile, prior knowledge of vehicle dynamics is embedded as a constraint term in the DRL reward function to avoid non-physical estimation results. For extreme scenarios such as low-adhesion roads, large sideslip angles, and load transfer, a hierarchical adaptive mechanism is further developed: tire saturation states are identified using tire force estimations from a fused Magic Formula model, triggering adjustments to the DRL reward weight for extreme conditions; a transfer learning mechanism is introduced to migrate DRL parameters trained under high-adhesion road conditions to low-adhesion scenarios for fine-tuning, reducing retraining time by more than 70%; and a 3D coupled scenario library of “full-type targets-omnidirectional conflicts-all-time weather” is constructed for the DRL generalization training, covering estimation requirements under complex environments. A two-level verification scheme of “simulation pre-verification-real-vehicle calibration” is established to fully verify the estimation performance.

To address the core limitations of the conventional UKF in vehicle sideslip angle estimation—specifically the inability to adaptively adjust process noise (Q) and measurement noise (R), as well as the degradation of estimation accuracy under complex operating conditions—this study proposes, for the first time, a novel observation method fusing UKF with DRL. Leveraging the inherent adaptive decision-making capability of DRL, the proposed method dynamically optimizes the noise covariance matrices (Q and R) of the UKF. In essence, this enables the sideslip angle estimation process to “track” the dynamic variations of its true value, thereby achieving dynamic and precise tracking of the sideslip angle while overcoming the inherent constraint of fixed noise parameters in traditional UKF. Targeting the key challenge that the sideslip angle is “difficult to measure and susceptible to interference” in practical vehicle operation, this study presents a high-precision estimation scheme that requires no additional sensors (utilizing existing sensor signals). This approach effectively reduces the engineering implementation barrier for the application of sideslip angle in vehicle active safety systems. Notably, under complex operating conditions where the sideslip angle is prone to abrupt changes (e.g., sharp steering maneuvers, operation on icy/snowy surfaces, and sensor signal interference), the proposed method maintains a stable output of accurate estimation results. It eliminates estimation jumps and distortions of the sideslip angle induced by inadequate noise adaptation in conventional methods, thus satisfying the critical requirement for “continuous precision” of the sideslip angle in real-world vehicle operation.

## 2. Establishment and Validation of the Vehicle Dynamics Model

**Figure 1:**
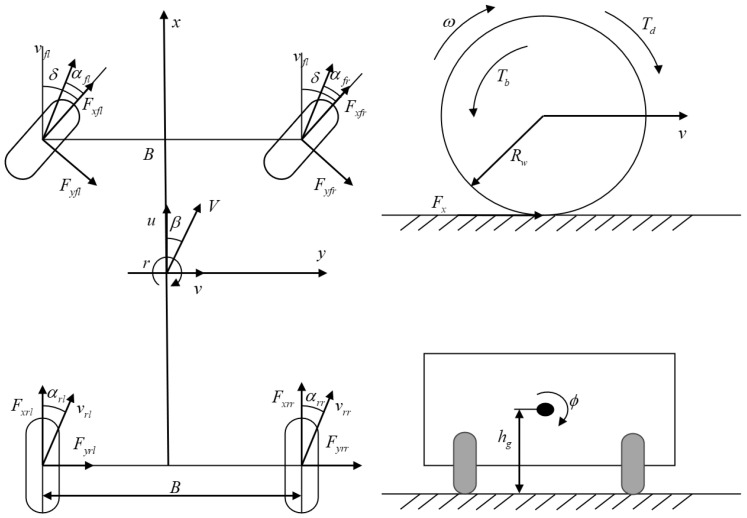
Diagram of the 8-DOF vehicle model.

To more accurately characterize the complex dynamic characteristics of the vehicle (including longitudinal driving, body roll, and independent wheel rotation effects), this study adopts an 8-DOF vehicle dynamics model, as shown in Figure 1. This model covers the vehicle body’s longitudinal motion (u), lateral motion (v), yaw motion (r), roll motion (ϕ), and the independent rotation motions of the four wheels ($\omega_{fl}$, $\omega_{fr}$, $\omega_{rl}$, $\omega_{rr}$; the subscripts represent front-left, front-right, rear-left, and rear-right, respectively). The model fully accounts for the coupling effects between various degrees of freedom (such as axle load transfer caused by roll and the influence of wheel slip ratio on ground forces), providing a high-fidelity state transition basis for UKF.

### 2.1. Derivation of Dynamic Equations by Degrees of Freedom

Based on Newton’s laws of motion and the theorem of angular momentum, the continuous-time dynamic equations of the 8-DOF model are derived by degrees of freedom [36]. The definitions and units of all variables are presented in Table 2, Definition of Core Variables for the 8-DOF Model, and the vehicle model diagram is shown in Figure 1.

#### 2.1.1. Body Longitudinal Motion

The longitudinal dynamic balance is provided by the resultant force of the longitudinal forces of the four wheels, considering the aerodynamic drag and rolling resistance:

(1) $$m\left(\dot{u}-vr+h_{g}\dot{\phi }\cos\phi \right)=\sum_{i=f/r}\sum_{j=l/r}F_{xij}-F_{air}-F_{roll}$$

where $h_{g}$ is the height of the vehicle’s center of mass, and the term cosϕ represents the coupling effect of roll motion on longitudinal acceleration; the aerodynamic drag $F_{air}=0.5\rho C_{d}Au^{2}$ (ρ is the air density, $C_{d}$ is the drag coefficient, and A is the frontal area); and the rolling resistance $F_{roll}=f_{r}mg$ ($f_{r}$ is the rolling resistance coefficient, g is the gravitational acceleration).

#### 2.1.2. Body Lateral Motion

The lateral dynamic balance is provided by the resultant force of the lateral forces of the four wheels, considering the coupling between yaw motion and roll motion:

(2) $$m\left(\dot{v}-ur+h_{g}\ddot{\phi }\right)=\sum_{i=f/r}\sum_{j=l/r}F_{yij}$$

where the term $h_{g}\ddot{\phi }$ is the correction of the roll angular acceleration to the lateral inertial force, and $F_{yij}$ needs to be dynamically adjusted in combination with the tire force saturation degree $S_{F}$.

#### 2.1.3. Body Yaw Motion

The yaw moment balance is provided by the moment difference of the longitudinal forces and lateral forces of the four wheels with respect to the center of mass:

(3) $$I_{z}\dot{r}=l_{f}\left(F_{yfl}+F_{yfr}\right)-l_{r}\left(F_{yrl}+F_{yrr}\right)+\frac{B}{2}\left[\left(F_{xfl}-F_{xfr}\right)+\left(F_{xrl}-F_{xrr}\right)\right]$$

where $\frac{B}{2}$ is half of the track width; the longitudinal force difference (between the left and right wheels) generates an additional yaw moment, which cannot be ignored under extreme operating conditions.

#### 2.1.4. Body Roll Motion

The roll moment balance is achieved by the balance among the roll stiffness moment of the suspension, the damping moment, and the restoring moment generated by the lateral force:

(4) $$I_{z}\dot{r}=l_{f}\left(F_{yfl}+F_{yfr}\right)-l_{r}\left(F_{yrl}+F_{yrr}\right)+\frac{B}{2}\left[\left(F_{xfl}-F_{xfr}\right)+\left(F_{xrl}-F_{xrr}\right)\right]$$

where $K_{\phi f}$ and $K_{\phi r}$ are the roll stiffness of the front/rear suspension; $C_{\phi f}$ and $C_{\phi r}$ are the roll damping of the front/rear suspension; and the term $mh_{g}\left(\dot{v}+ur\right)$ on the right-hand side is the roll restoring moment generated by the lateral inertial force, which suppresses the body roll.

#### 2.1.5. Wheel Rotational Motion

The rotational dynamic equations for the four wheels have the same form:

(5) $$I_{w}{\dot{\omega }}_{ij}=T_{dij}-T_{bij}-F_{xij}R_{w}$$

where $I_{w}$ is the moment of inertia of a single wheel; $T_{dij}$ is the driving torque; $T_{bij}$ is the braking torque; and $F_{xij}R_{w}$ is the resistance moment exerted by the ground longitudinal force on the wheel center. During driving, $F_{xij}$ is positive (representing driving force), and during braking, it is negative (representing braking force).

### 2.2. Tire Force Model and Axle Load Transfer Calculation

Tire force is the core that connects vehicle motion and ground interaction. It needs to be calculated by integrating axle load transfer, slip ratio/sideslip angle, and tire force saturation degree, and serves as the state input for the DRL adaptive strategy.

#### 2.2.1. Calculation of Axle Load Transfer

Both body roll and longitudinal acceleration can cause axle load redistribution, and the calculation of the vertical load of the four wheels is as follows:

Static Axle Load: The total load on the front axle: $F_{zf0}=mgl_{r}/\left(l_{f}+l_{r}\right)$; the total load on the rear axle: $F_{zr0}=mgl_{f}/\left(l_{f}+l_{r}\right)$;

Longitudinal Axle Load Transfer: The longitudinal acceleration $a_{x}=\dot{u}-vr$ causes the front-rear transfer of the axle load; the transfer amount is $\Delta F_{zlong}=mh_{g}a_{x}/\left(l_{f}+l_{r}\right)$;

Roll Axle Load Transfer: The roll angle ϕ causes the left-right transfer of the axle load; the transfer amount is $\Delta F_{zroll,i}=K_{\phi i}\phi /B$ (the front/rear axle).

The final vertical loads of the four wheels are as follows:

(6) $$\left\{\begin{array}{l} F_{zfl}=\frac{F_{zf0}-\Delta F_{zlong}}{2}+\Delta F_{zroll,f}\\ F_{zfr}=\frac{F_{zf0}-\Delta F_{zlong}}{2}-\Delta F_{zroll,f}\\ F_{zrl}=\frac{F_{zr0}+\Delta F_{zlong}}{2}+\Delta F_{zroll,r}\\ F_{zrr}=\frac{F_{zr0}+\Delta F_{zlong}}{2}-\Delta F_{zroll,r} \end{array}\right.$$

where the “+” sign indicates an increase in the axle load of the left-side wheels, and the “−” sign indicates a decrease in the axle load of the right-side wheels (when the roll angle *ϕ* is positive, the vehicle body tilts to the left).

#### 2.2.2. Tire Longitudinal Force Model

The longitudinal force model adopting the Pacejka Magic Formula takes into account the effects of axle load $F_{zij}$ and road adhesion coefficient μ [37]:

(7) $$F_{xij}=D_{x}\sin\left(C_{x}\arctan\left(B_{x}s_{ij}-E_{x}\left(B_{x}s_{ij}-\arctan\left(B_{x}s_{ij}\right)\right)\right)\right)$$

where $s_{ij}$ is the wheel slip ratio during driving $s_{ij}=\left(R_{w}\omega_{ij}-u\right)/u$ (*s_ij_* > 0) and during braking $s_{ij}=\left(R_{w}\omega_{ij}-u\right)/R_{w}\omega_{ij}$ (*s_ij_* < 0); the model parameter *B_x_* is the stiffness factor, $C_{x}$ is the shape factor, $E_{x}$ is the slope factor, and $D_{x}=\mu F_{zij}$ is the peak longitudinal force. The calculation of the slip ratio must incorporate four observed wheel speed values to correct errors caused by the skidding of a single-side wheel.

#### 2.2.3. Tire Lateral Force Model

The formula for the lateral force model is as follows:

(8) $$F_{yij}=D_{y}\sin\left(C_{y}\arctan\left(B_{y}\alpha_{ij}-E_{y}\left(B_{y}\alpha_{ij}-\arctan\left(B_{y}\alpha_{ij}\right)\right)\right)\right)\cdot \left(1-S_{F}^{1.2}\right)$$

where the front wheel sideslip angle is $\alpha_{fl/fr}=\delta -\arctan\left(\frac{v-l_{f}r\pm \overset{\cdot }{\phi }B/2}{u}\right)$ (corresponding to the left/right front wheels, respectively; the roll angular velocity causes a difference in the lateral velocity between the left and right wheels); the rear wheel sideslip angle is $\alpha_{rl/rr}=-\arctan\left(\frac{v-l_{r}r\pm \overset{\cdot }{\phi }B/2}{u}\right)$; the peak lateral force is $D_{y}=\mu F_{zij}\cdot \sqrt{1-{\left(\frac{F_{xij}}{\mu F_{zij}}\right)}^{2}}$ (the influence of longitudinal force on lateral force is corrected by the adhesion ellipse constraint [38]); and the tire force saturation degree is $S_{F}=F_{yij}/D_{y}$, which is used for DRL working condition classification ($S_{F}<0.7$ is the unsaturated zone, and $S_{F}\geq 0.7$ is the saturated zone).

### 2.3. Validation of Model Effectiveness

The established vehicle model is validated using steady-state cornering, steering angle pulse, and central zone steering operating conditions. The main parameters of the vehicle are shown in Table 3.

**Figure 2:**
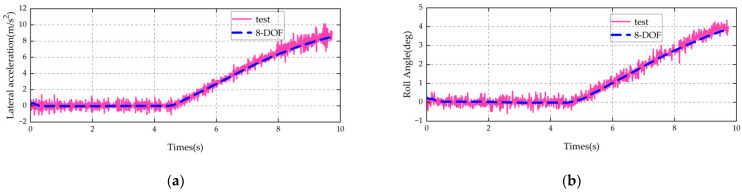
Validation results of steady-state cornering condition: (**a**) lateral acceleration; (**b**) body roll angle.

**Figure 3:**
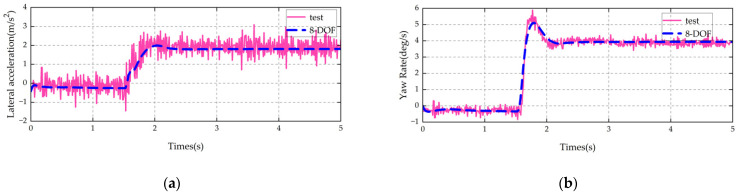
Validation results of step steer condition: (**a**) lateral acceleration; (**b**) yaw rate.

**Figure 4:**
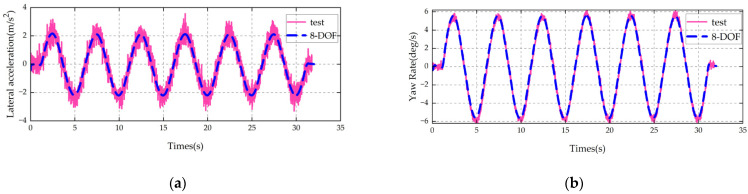
Validation results of sinusoidal steering condition: (**a**) lateral acceleration; (**b**) yaw rate.

The same conditions as those of the test vehicle, such as vehicle speed and steering wheel angle (converted to wheel angle), are input into the vehicle model. A comparison between the model simulation results and the vehicle test results is shown in Figure 2, Figure 3 and Figure 4.

It can be seen from the above comparisons that the 8-DOF model proposed in this paper is in good agreement with the key indicators of the actual vehicle. It can accurately characterize the multi-degree-of-freedom (multi-DOF) coupled dynamics of the vehicle and thus can be used as the state transition model for the UKF.

## 3. Refined Design of UKF Based on the 8-DOF Model

In view of the high-dimensional state characteristics of the 8-DOF model, the state vector definition, Sigma point generation, discretization strategy, and covariance decoupling method of the UKF are optimized. This ensures that while retaining all degree-of-freedom information, the real-time performance and stability of the filter are maintained.

### 3.1. Extended Definition of State and Observation Vectors

#### 3.1.1. State Vector

Covers all dynamic states of the 8-DOF model to ensure no information loss:

(9) $$x={\left[u,v,r,\phi ,\omega_{fl},\omega_{fr},\omega_{rl},\omega_{rr}\right]}^{T}$$

where u is the longitudinal velocity; v is the lateral velocity; r is the yaw rate; ϕ is the roll angle; and $\omega_{fl}/\omega_{fr}/\omega_{rl}/\omega_{rr}$ is the rotational speed of the four wheels.

#### 3.1.2. Observation Vector

Combined with the low-cost on-board sensor configuration, directly measurable physical quantities are selected to balance observation accuracy and engineering costs:

(10) $$z={\left[r_{meas},a_{x,meas},a_{y,meas},\phi_{meas},\omega_{fl,meas},\omega_{fr,meas},\omega_{rl,meas},\omega_{rr,meas}\right]}^{T}$$

Sensor configuration and model mapping for each observation item:

1. $r_{meas}$: yaw rate, core sensor: MEMS Gyroscope (IMU), observation function $h_{1}\left(x\right)=r$;
2. $a_{x,meas}$: longitudinal acceleration, core sensor: MEMS Accelerometer (IMU), observation function
  $$h_{2}\left(x\right)=\overset{\cdot }{u}-vr+h_{g}\overset{\cdot }{\phi }\cos\phi$$
3. $a_{y,meas}$: lateral acceleration, core sensor: MEMS Accelerometer (IMU), observation function $h_{3}\left(x\right)=\overset{\cdot }{v}+ur-h_{g}\overset{\cdot \cdot }{\phi }$
4. $\phi_{meas}$: roll angle, core sensor: IMU (Accelerometer + Gyroscope), observation function $h_{4}\left(x\right)=\phi$
5. $\omega_{fl,meas}/\omega_{fr,meas}/\omega_{rl,meas}/\omega_{rr,meas}$: four-wheel speed, core sensor: wheel speed sensor, observation function $h_{5}\left(x\right)=\omega_{fl}$, $h_{6}\left(x\right)=\omega_{fr}$, $h_{7}\left(x\right)=\omega_{rl}$, $h_{8}\left(x\right)=\omega_{rr}$.

### 3.2. Optimization of Core Parameters for High-Dimensional UKF

#### 3.2.1. Initialization Parameters

Initial state estimation ${\hat{x}}_{0}$: When the vehicle is stationary, ${\hat{x}}_{0}={\left[0,0,0,0,0,0,0,0\right]}^{T}$;

Initial covariance matrix $P_{0}$ (8 × 8): Based on the diagonalized setting of the initial uncertainty for each state, $P_{0}=diag\left[0.16,0.09,1\times 10^{-4},4\times 10^{-4},16,16,16,16\right]$ (the diagonal elements are the variances of u, v, r, ϕ, $\omega_{fl}$, $\omega_{fr}$, $\omega_{rl}$, and $\omega_{rr}$ in sequence).

$P_{0}$ represents the initial uncertainty of the 8-dimensional state variables at the initial moment when the vehicle is stationary (*t* = 0). The matrix is a diagonal matrix (assuming the initial states are independent of each other).

Longitudinal velocity u: Initial uncertainty $\sigma_{u}=0.4\;m/s$, with a variance of 0.4^2^ = 0.16, which is the statistical data of the estimation error of u during the vehicle’s stationary start-up phase;

Lateral velocity v: Derived based on the model and determined by empirical values, *σ_v_* = 0.3 m/s, with a variance of 0.09;

Yaw rate r: Static noise of the sensor $\sigma_{r}=0.01\;\mathrm{rad}/s$, with a variance of 1 × 10^−4^;

Roll angle ϕ: Static noise of the sensor $\sigma_{\phi }=0.02\;\mathrm{rad}/s$, with a variance of 4 × 10^−4^;

Four-wheel speed $\omega_{ij}$: Under four-wheel drive, the speed difference between the front and rear wheels is small. For initial uncertainty $\sigma_{\omega }=4\;\mathrm{rad}/s$, the variance is 4^2^ = 16.

Initial process noise covariance $Q_{init}$ (8 × 8):

$$Q_{init}=diag\left[0.04,0.03,1\times 10^{-5},1\times 10^{-4},0.8,0.8,0.8,0.8\right]$$

$Q_{init}$ describes the modeling errors of the 8-DOF dynamic model itself and external disturbances, and provides noise constraints for the state transition process. The matrix is a diagonal matrix (assuming the process noises are independent of each other). Through analysis of u, v, r, ϕ, $\omega_{fl}$, $\omega_{fr}$, $\omega_{rl}$, $\omega_{rr}$, the RMSE values of each variable are 0.2 m/s, 0.17 m/s, 0.003 rad/s, 0.01 rad, 0.9 rad/s, 0.9 rad/s, 0.9 rad/s, and 0.9 rad/s, respectively, with corresponding variances of 0.04, 0.03, 1 × 10^−5^, 1 × 10^−4^, 0.8, 0.8, 0.8, and 0.8.

Initial observation noise covariance $R_{init}$ (8 × 8): Based on the sensor noise parameters, $R_{init}=diag\left[8.47\times 10^{-6},0.01,0.04,8.47\times 10^{-6},4,4,4,4\right]$ (the diagonal elements are the variances of $r_{meas}$, $a_{x,meas}$, $a_{y,meas}$, $\phi_{meas}$, $\omega_{fl,meas}$, $\omega_{fr,meas}$, $\omega_{rl,meas}$, and $\omega_{rr,meas}$ in sequence).

$R_{init}$ represents the sensor measurement noise corresponding to the 8-dimensional observation vector, and the matrix is a diagonal matrix (assuming the sensor noises are independent of each other).

Yaw rate $r_{meas}$: Sensor noise $\sigma_{r,means}=0.1{}^{\circ}/s=0.001745\;\mathrm{rad}/s$, with a variance of (0.001745)^2^ ≈ 8.47 × 10^−6^;

Longitudinal acceleration $a_{x,meas}$: Sensor noise $\sigma_{a_{x},means}=0.1\;m/s^{2}$, with a variance of 0.01;

Lateral acceleration $a_{y,meas}$: Sensor noise $\sigma_{a_{x},means}=0.2\;m/s^{2}$, with a variance of 0.04;

Roll angle $\phi_{meas}$: Sensor noise $\sigma_{\phi ,means}=0.1{}^{\circ}/s=0.001745\;\mathrm{rad}/s$, with a variance of (0.001745)^2^ ≈ 8.47 × 10^−6^;

Four-wheel speed $\omega_{ij,means}$: Sensor noise $\sigma_{\omega ,means}=2\;\mathrm{rad}/s$, with a variance of 2^2^ = 4.

#### 3.2.2. Calculation of 8-Dimensional Sigma Point Parameters and Weights

In an 8-dimensional state, the number of Sigma points is 2n+1=17, and parameter setting needs to balance sampling efficiency and distribution rationality in the high-dimensional space [39]:

Scaling parameter α=0.02: Ensure that the Sigma points cover a sufficient state range in the high-dimensional space;

Secondary scaling parameter κ=0: Maintain the approximation accuracy of high-order moments of the Gaussian distribution;

High-order moment parameter β=2: Adapt to the Gaussian assumption of noise;

Scaling factor $\lambda =\alpha^{2}\left(n+\kappa \right)-n$;

Sigma point weights (mean weight $W_{m}^{i}$, covariance weight $W_{c}^{i}$):

(11) $$\left\{\begin{array}{l} W_{m}^{0}=\frac{\lambda }{n+\lambda }\\ W_{c}^{0}=\frac{\lambda }{n+\lambda }+\left(1-\alpha^{2}+\beta \right)\\ W_{m}^{i}=W_{c}^{i}=\frac{1}{2\left(n+\lambda \right)}\left(i=1,2,\ldots ,16\right) \end{array}\right.$$

### 3.3. Implementation of UKF Discretization

Runge–Kutta (RK4) discretization is adopted instead of Euler discretization to improve the accuracy of high-dimensional state transition (the 8-DOF model has strong coupling, and Euler discretization errors are prone to accumulation), with a sampling period of $T_{s}=0.01\;s$.

#### 3.3.1. Prediction Step (8-Dimensional State Transition)

1. Sigma Point Generation:

For ${\hat{x}}_{k-1}$ and $P_{k-1}$ at time k−1, 17 Sigma points are generated via Cholesky decomposition [40]:

(12) $$x_{i}^{k-1}=\left\{\begin{array}{l} {\hat{x}}_{k-1}\\ {\hat{x}}_{k-1}+\sqrt{\left(n+\lambda \right)P_{k-1}}(i)\\ {\hat{x}}_{k-1}-\sqrt{\left(n+\lambda \right)P_{k-1}}(i-8) \end{array}\right.\begin{array}{l} \left(i=0\right)\\ \left(i=1,2,\ldots ,8\right)\\ \left(i=9,10,\ldots ,16\right) \end{array}$$

In the formula, $\sqrt{\left(n+\lambda \right)P_{k-1}}(i)$ denotes the *i*-th column of the matrix after Cholesky decomposition. If $P_{k-1}$ is singular, $\varepsilon I\left(\varepsilon =1\times 10^{-6}\right)$ is added to ensure the validity of the decomposition.

2. RK4 Discretized State Transition:

For each Sigma point $x_{i}^{k-1}={\left[u_{i},v_{i},r_{i},\phi_{i},\omega_{fl,i},\omega_{fr,i},\omega_{rl,i},\omega_{rr,i}\right]}^{T}$, the 4th-order Runge–Kutta (RK4) method is used to solve the 8-DOF continuous equations, yielding $x_{i}^{k|k-1}$;

The coefficients $k_{1},k_{2},k_{3},k_{4}$ of the 4th-order Runge–Kutta (RK4) method are all 8-dimensional vectors (corresponding to the derivatives of the 8 states). They approximate the numerical solution of the continuous dynamic equation through multi-step intermediate sampling, and are defined as follows:

*k*_1_: Calculated at the sampling time $t=kT_{s}$ based on the state derivative of the current Sigma point $X_{i}^{k-1}$, i.e., $k_{1}=f(x_{i}^{k-1},t)$, where $k_{1}=f(\cdot )$ is the derivative vector of the 8-degree-of-freedom continuous dynamic equation;

*k*_2_: Calculated at the intermediate time $t=kT_{s}+T_{s}/2$ based on the state derivative of $x_{i}^{k-1}+T_{s}\cdot k_{1}/2$, i.e., $k_{2}=f(x_{i}^{k-1}+T_{s}\cdot k_{1}/2,t+T_{s}/2);$

*k*_3_: Calculated at the intermediate time $t=kT_{s}+T_{s}/2$ based on the state derivative of $x_{i}^{k-1}+T_{s}\cdot k_{2}/2$, i.e., $k_{3}=f(x_{i}^{k-1}+T_{s}\cdot k_{2}/2,t+T_{s}/2);$

*k*_4_: Calculated at the sampling time $t=(k+1)T_{s}$ based on the state derivative of $x_{i}^{k-1}+T_{s}\cdot k_{3}$, i.e., $k_{4}=f(x_{i}^{k-1}+T_{s}\cdot k_{3},t+T_{s})$.

Discretized update:

(13) $$x_{i}^{k|k-1}=x_{i}^{k-1}+\frac{T_{s}}{6}\left(k_{1}+2k_{2}+2k_{3}+k_{4}\right)$$

3. Calculation of Predicted State and Covariance:

The 8-dimensional predicted state ${\hat{x}}_{k|k-1}$ and predicted covariance $P_{k|k-1}$ (including the *Q_k_* dynamically adjusted by DRL) are obtained through weighted summation:

(14) $${\hat{x}}_{k|k-1}=\sum_{i=0}^{16}W_{m}^{i}x_{i}^{k|k-1}$$

(15) $$P_{k|k-1}=\sum_{i=0}^{16}W_{c}^{i}\left(x_{i}^{k|k-1}-{\hat{x}}_{k|k-1}\right){\left(x_{i}^{k|k-1}-{\hat{x}}_{k|k-1}\right)}^{T}+Q_{k}$$

#### 3.3.2. Update Step

1. Observation Sigma Point Generation:

Substitute $x_{i}^{k|k-1}$ into the 8 observation functions $h\left(x\right)$ to obtain 17 observation Sigma points:

(16) $$Z_{i}^{k|k-1}={\left[h_{1}\left(x_{i}^{k|k-1}\right),h_{2}\left(x_{i}^{k|k-1}\right),\ldots ,h_{8}\left(x_{i}^{k|k-1}\right)\right]}^{T}$$

2. Calculation of Observation Covariance and Cross-Covariance:

Observation means:

(17) $${\hat{z}}_{k|k-1}=\sum_{i=0}^{16}W_{m}^{i}Z_{i}^{k|k-1}$$

Observation covariance:

(18) $$P_{zz}=\sum_{i=0}^{16}W_{c}^{i}\left(Z_{i}^{k|k-1}-{\hat{z}}_{k|k-1}\right){\left(Z_{i}^{k|k-1}-{\hat{z}}_{k|k-1}\right)}^{T}+R_{k}$$

State-observation cross-covariance:

(19) $$P_{xz}=\sum_{i=0}^{16}W_{c}^{i}\left(x_{i}^{k|k-1}-{\hat{x}}_{k|k-1}\right){\left(Z_{i}^{k|k-1}-{\hat{z}}_{k|k-1}\right)}^{T}$$

3. Kalman Gain and State Update:

Kalman gain:

(20) $$K_{k}=P_{xz}P_{zz}^{-1}$$

State and covariance update:

(21) $${\hat{x}}_{k}={\hat{x}}_{k|k-1}+K_{k}\left(z_{k}-{\hat{x}}_{k|k-1}\right)$$

(22) $$P_{k}=P_{k|k-1}-K_{k}P_{zz}K_{k}^{T}$$

### 3.4. Lightweight Design of UKF

To address the computational power limitations of vehicle-mounted systems, a lightweight “covariance decoupling” scheme is proposed. This scheme reduces the dimensionality of high-dimensional optimization problems while balancing estimation accuracy and computational efficiency.

The 8 × 8 state covariance matrix *P* is decomposed into 3 independent low-dimensional subsystems, eliminating the computational overhead associated with matrix inversion for high-dimensional matrices:

1. Longitudinal Wheel Speed Subsystem: State variables $\left[u,\omega_{fl},\omega_{fr},\omega_{rl},\omega_{rr}\right]$, covariance submatrix $P_{1}$ (5 × 5), and optimization objective: estimation accuracy of longitudinal velocity and wheel speeds;
2. Lateral–Roll Subsystem: State variables $\left[v,r,\phi \right]$, covariance submatrix $P_{2}$ (3 × 3), and optimization objective: estimation accuracy of sideslip angle and roll angle;
3. Tire Force Correlation Subsystem: The coupling weights between subsystems are dynamically adjusted based on the tire force saturation degree $S_{F}$. In the non-saturation region, the focus is on $P_{2}$ optimization; in the saturation region, the focus is on $P_{1}$ robustness.

After decoupling, the single-step computational load of the UKF is reduced, laying a foundation for the lightweight implementation of DRL.

## 4. DRL Noise Adjustment Framework Adapted to the 8-DOF Model

**Figure 5:**
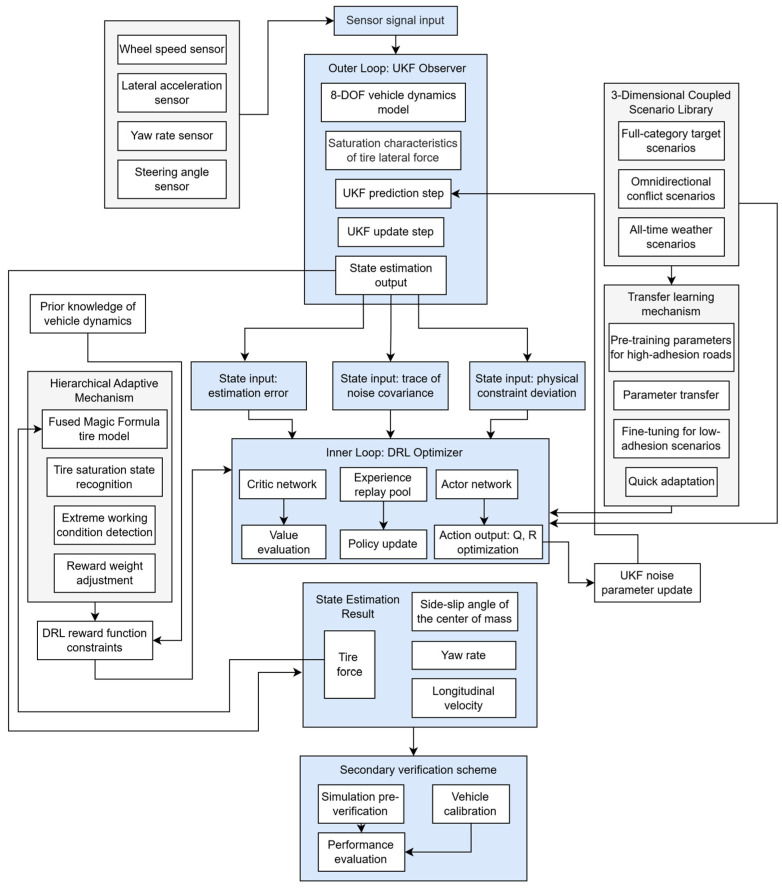
Observer structure diagram.

Given the high-dimensional noise characteristics of the 8-DOF UKF (where Q is an 8 × 8 matrix and R is an 8 × 8 matrix), combined with tire force perception, the state/action space of DRL is expanded, and the reward function is optimized to achieve adaptive adjustment of the noise covariance. The observer structure is shown in Figure 5.

### 4.1. State Space Expansion

A 14-dimensional state space is established to ensure the agent fully perceives the multi-degree-of-freedom coupled working conditions:

(23) $$S_{k}={\left[u,a_{x},f,\dot{f},e_{\omega fl},e_{\omega fr},e_{\omega rl},e_{\omega rr},F_{y,est},S_{F},\hat{\beta },tr\left(P_{k}\right),\mu_{est},\beta_{vt}\right]}^{T}$$

where $e_{\omega fl}/e_{\omega fr}/e_{\omega rl}/e_{\omega rr}$ are the measurement errors of the four-wheel speeds, $e_{\omega ij}=\omega_{ij,meas}-{\hat{\omega }}_{ij}$ reflect the adaptability of wheel speed noise in R; $F_{y,est}$ is the estimated values of tire lateral forces, which reflect the dynamic state of tire forces and provide a basis for working condition classification; $S_{F}$ is the tire force saturation degrees, which divide the working condition intervals $S_{F}<0.7$ for the non-saturation region, $S_{F}\geq 0.7$ for the saturation region, and guide the optimization focus of DRL.

State normalization adopts min–max mapping. The value range is as follows:

$$F_{y,est}\in \left[-5000,5000\right]\;N,\;S_{F}\in \left[0,1\right],\;\beta_{vt}\in \left[-0.2,0.2\right]\;\mathrm{rad},\;u\in \left[0,35\right]\;m/s,\;a_{x}\in \left[-10,5\right]\;m/s^{2},\;\phi \in \left[-0.17,0.17\right]\;\mathrm{rad},\;\overset{\cdot }{\phi }\in \left[-0.5,0.5\right]\;\mathrm{rad}/s,\;\;e_{\omega ij}\in \left[-8,8\right]\;\mathrm{rad}/s$$

### 4.2. Action Space Expansion

The action vector corresponds to the adjustment amounts of the diagonal elements of the 8-dimensional Q and 8-dimensional R, with a total of 16 action dimensions:

(24) $$A_{k}={\left[\Delta q_{u},\Delta q_{v},\Delta q_{r},\Delta q_{f},\Delta q_{\omega fl},\Delta q_{\omega fr},\Delta q_{\omega rl},\Delta q_{\omega rr},\Delta r_{r},\Delta r_{a_{x}},\Delta r_{a_{y}},\Delta r_{f},\Delta r_{\omega fl},\Delta r_{\omega fr}\Delta r_{\omega rl},\Delta r_{\omega rr}\right]}^{T}$$

where Δq represents the adjustment amounts of the diagonal elements of Q, corresponding to the process noises of u, v, r, ϕ, $\omega_{fl}$, $\omega_{fr}$, $\omega_{rl}$, $\omega_{rr}$; Δr represents the adjustment amounts of the diagonal elements of R, corresponding to the observation noises of $r_{meas}$, $a_{x,meas}$, $a_{y,meas}$, $\phi_{meas}$, $\omega_{fl,meas}$, $\omega_{fr,meas}$, $\omega_{rl,meas}$, $\omega_{rr,meas}$.

Rules for Action Activation and Update:

Activation function: The Softplus function is used to ensure positive definiteness, $\Delta q=\ln\left(1+e^{a}\right)$, $\Delta r=\ln\left(1+e^{a}\right)$ (a is the original outputs of the Actor network);

Update constraint: $Q_{k}=clip\left(Q_{k-1}+diag\left(\Delta q\right)\cdot T_{s},Q_{\min},Q_{\max}\right)$, and the same logic applies to $R_{k}$;

Boundary setting: Among them, $Q_{\max}$ and $R_{\max}$ are used to prevent the noise covariances $Q_{k}$ and $R_{k}$ from deviating excessively from physical constraints due to the adjustment of DRL actions, thereby ensuring estimation accuracy. Their values need to be jointly determined based on sensor performance, model dynamic range, and other factors—specifically, ensuring that the maximum measurement error does not exceed the sensor performance limit and that the model process noise does not exceed the maximum possible variation of the state. Combined with relevant experience, the obtained values are as follows: $Q_{\max}=diag\left(\left[0.1,0.08,2\times 10^{-5},2\times 10^{-4},1.8,1.8,1.8,1.8\right]\right)$, $R_{\max}=diag\left(\left[2\times 10^{-5},0.02,0.08,2\times 10^{-5},5,5,5,5\right]\right)$.

### 4.3. Reward Function Optimization

A dual-structure of “immediate reward + terminal reward” is adopted, integrating tire force perception and safety boundary indicators to balance estimation accuracy and robustness under different working conditions.

#### 4.3.1. Immediate Reward $R_{immediate}$

Immediate Reward associates estimation error, covariance stability, and tire force state, and dynamically adjusts weights according to working condition intervals:

(25) $$R_{immediate}=\omega_{1}R_{acc,\beta }+\omega_{2}R_{acc,\phi }+\omega_{3}R_{acc,\omega }+\omega_{4}R_{stab}+\omega_{5}R_{smooth}+\omega_{6}R_{F_{y}}$$

Definition and weight setting of each reward term:

- $R_{acc,\beta }$ (Sideslip Angle Accuracy Reward): $R_{acc,\beta }=-\left(\left|\hat{\beta }-\beta_{true}\right|+0.5{\left(\hat{\beta }-\beta_{true}\right)}^{2}\right)$, $\omega_{1}=12$ (core estimation target, $\omega_{1}=15$ in the non-saturation region, $\omega_{1}=10$ in the saturation region);
- $R_{acc,\phi }$ (Roll Angle Accuracy Reward): $R_{acc,\phi }=-\left|\hat{\phi }-\phi_{true}\right|$, $\omega_{2}=6$ (roll angle affects axle load and lateral force, with a fixed weight);
- $R_{acc,\omega }$ (Wheel Speed Accuracy Reward): $R_{acc,\omega }=-\frac{1}{4}\sum_{ij}\left(\left|{\hat{\omega }}_{ij}-\omega_{ij,true}\right|\right)$, $\omega_{3}=3$ (wheel speed affects slip ratio, with a fixed weight);
- $R_{stab}$ (Filter Stability Reward): $R_{stab}=-\left(tr\left(P_{k}\right)+0.1\left|tr\left(P_{k}\right)-tr\left(P_{k-1}\right)\right|\right)$, $\omega_{4}=0.15$ ($tr\left(P_{k}\right)$ is the trace of the 8-dimensional covariance matrix, with a fixed weight);
- $R_{smooth}$ (Action Smoothness Reward): $R_{smooth}=-{\left\|A_{k}\right\|}_{2}^{2}$, $\omega_{5}=0.01$ (to avoid severe fluctuations of the 16-dimensional action, with a fixed weight);
- $R_{F_{y}}$ (Tire Force Adaptation Reward): $R_{F_{y}}=-\left|S_{F}-S_{F,opt}\right|$ (for $S_{F,opt}$, it is 0.5 in the non-saturation region and 0.8 in the saturation region), $\omega_{6}=5$ ($\omega_{6}=3$ in the non-saturation region, $\omega_{6}=7$ in the saturation region, to enhance robustness in the saturation region).

#### 4.3.2. Terminal Reward $R_{ter\min al}$

Terminal Reward is triggered based on safety boundary indicators to avoid the accumulation of “extreme error” samples during the training process:

(26) $$R_{ter\min al}=\left\{\begin{array}{l} -100\\ 20\\ -30 \end{array}\right.\begin{array}{l} \left(Collision\;Risk:\;\hat{\beta }>8{}^{\circ}\;or\;Rollover\;Warning:\;\hat{f}>10{}^{\circ}\right)\\ \left(The\;episode\;ends\;normally\;without\;safety\;risks\right)\\ \left(Covariance\;Divergence:\;tr\left(P_{k}\right)>50\right) \end{array}$$

### 4.4. PPO Network Structure and Training Optimization

#### 4.4.1. Network Lightweight Design

To adapt to 14-dimensional input and 16-dimensional output, the computational load is reduced through parameter pruning and structure optimization:

Actor Network: Input Layer (14) → Hidden Layer 1 (64, ReLU) → LayerNorm → Hidden Layer 2 (32, ReLU) → LayerNorm → Output Layer (16, Linear);

Critic Network: Input Layer (14) → Hidden Layer 1 (64, ReLU) → LayerNorm → Hidden Layer 2 (32, ReLU) → LayerNorm → Output Layer (1, Linear). It shares the feature extraction layer with the Actor to reduce parameter redundancy.

#### 4.4.2. Training Strategy Adjustment

Combined with the digital twin hybrid dataset, phased training is implemented to improve generalization ability:

Data Preparation: Expand the size of the replay buffer to store hybrid samples of “physical sensor data + digital twin virtual data”, covering the μ=0.15~0.9 range of the adhesion coefficient.

Training Phases: The algorithm parameters are set as follows: the learning rate (Actor–Critic) is set to 3 × 10^−4^; the discount factor is γ=0.99; the GAE coefficient is λ=0.95; the clipping coefficient is ε=0.2; the batch size is set to 128; the number of training epochs is set to 1500; the LayerNorm is set to 0.01; the policy update interval is set to 20 steps; and the max grad norm is set to 0.5.

Phase 1: Basic working condition training (straight-line acceleration, small-angle steering, $S_{F}<0.6$), focusing on estimation accuracy optimization;

Phase 2: Complex working condition training (emergency braking, serpentine driving, $S_{F}=0.6\sim 0.8$), incorporating tire force saturation weight adjustment;

Phase 3: Extreme working condition training (μ=0.15~0.3, large-angle steering, $S_{F}\geq 0.8$), relying on digital twin virtual data to supplement samples.

**Figure 6:**
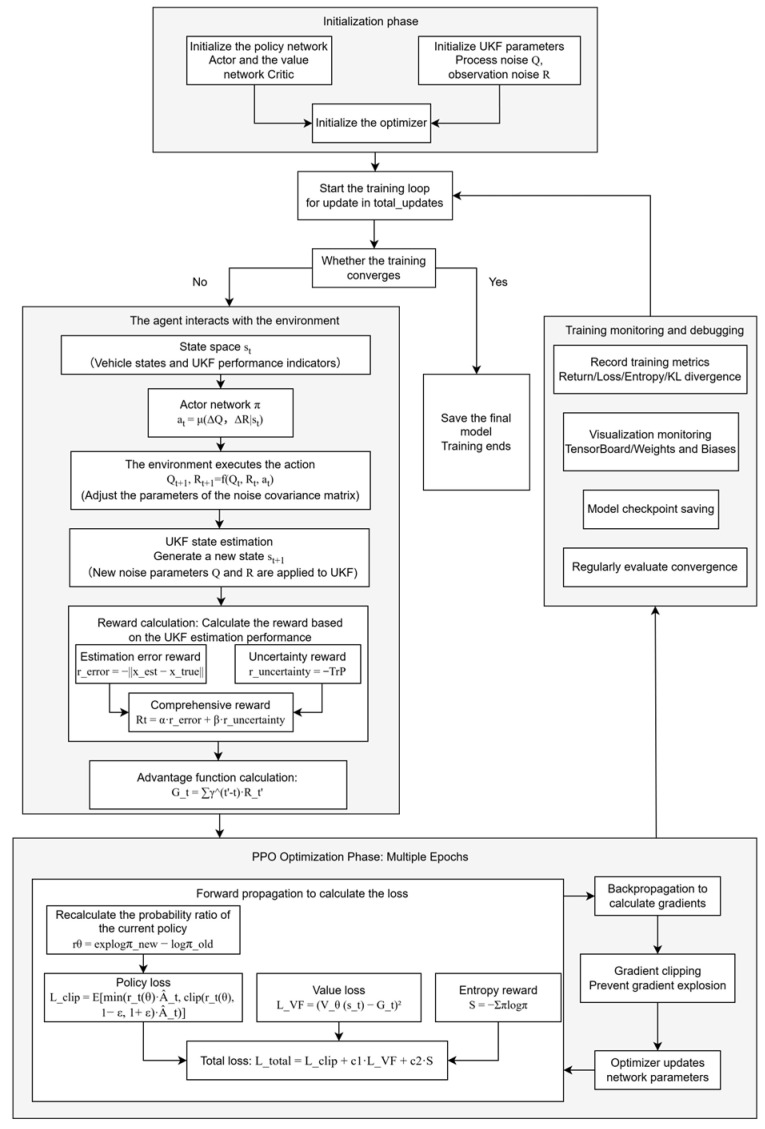
PPO training process diagram.

The PPO training process is shown in Figure 6.

## 5. Robustness and Stability Analysis of the Estimation Method

The DRL-UKF sideslip angle estimation method based on the 8-DOF model must maintain reliable performance under scenarios such as perturbations of vehicle dynamics parameters, variations in sensor noise, and sudden changes in working conditions. This section conducts an analysis from two dimensions: robustness (anti-interference capability) and stability (filter convergence). Combining theoretical derivation and framework design characteristics, it analyzes the reliability of this estimation method in engineering applications.

### 5.1. Robustness Analysis

Robustness is defined as the ability of the estimation system to maintain the estimation accuracy of the sideslip angle under model parameter uncertainties, sensor noise interference, and dynamic sudden changes in working conditions [41]. The proposed method constructs an anti-interference mechanism through the adaptive adjustment of UKF noise parameters ($Q_{k}$, $R_{k}$) by DRL, with specific analysis as follows:

#### 5.1.1. Robustness to Model Parameter Perturbations

Key parameters of the 8-DOF (such as tire cornering stiffness $C_{\alpha }$, vehicle body moment of inertia $I_{z}$, and suspension roll stiffness $K_{\phi }$) are prone to perturbations due to factors like tire wear, load changes, and temperature drift [42]. Traditional UKF, with fixed Q, suffers from direct estimation deviations caused by model errors, while the proposed method resists parameter perturbations through the following mechanisms:

Perturbation Perception: The state space of DRL includes $\mu_{est}$ (estimated road adhesion coefficient) and $tr\left(P_{k}\right)$ (trace of the covariance matrix). When model parameters are perturbed—for example, a 15% reduction in front tire cornering stiffness $C_{\alpha f}$—the calculation deviation of tire lateral force $F_{yij}$ increases, leading to a rise in the UKF estimation covariance $tr\left(P_{k}\right)$ and an increase in the sideslip angle estimation error $\left|\hat{\beta }-\beta_{true}\right|$. The agent perceives model mismatch in real time through these two state variables.

Noise Adjustment Strategy: The agent dynamically optimizes the diagonal elements of $Q_{k}$ for perturbation sources. For instance, when tire cornering stiffness is perturbed, model errors in lateral velocity v and yaw rate r dominate. The agent increases $\Delta q_{v}$ and $\Delta q_{r}$ to ensure the corresponding terms in $Q_{k}$ satisfy $q_{v}\geq \mathrm{cov}\left(\Delta v\right)$ and $q_{r}\geq \mathrm{cov}\left(\Delta r\right)$ (where Δv, Δr are state errors caused by model parameter perturbations); when suspension roll stiffness is perturbed, the estimation error of roll angle ϕ increases. The agent compensates for the model deviation in roll-axle load coupling by increasing $\Delta q_{\phi }$, ensuring $Q_{k}$ always covers actual model uncertainties.

Theoretical Verification: Let model parameter perturbations be Δθ and state transition errors be $\Delta f_{k}=f\left(x_{k},\theta +\Delta \theta \right)-f\left(x_{k},\theta \right)$.

From the UKF prediction covariance formula, the adjustment of $Q_{k}$ ensures $P_{k|k-1}$ includes $\mathrm{cov}\left(\Delta f_{k}\right)$:

$$P_{k|k-1}\geq \sum_{i=0}^{16}W_{c}^{i}\Delta x_{i}^{k|k-1}\Delta x_{i}^{k|k-1,T}$$

where $\Delta x_{i}^{k|k-1}=\Delta x_{i}^{k|k-1}\left(\theta +\Delta \theta \right)-\Delta x_{i}^{k|k-1}\left(\theta \right)$, guaranteeing that the prediction covariance does not underestimate model errors and thus avoiding filter divergence.

#### 5.1.2. Subsubsection

The noise characteristics of on-vehicle sensors are susceptible to electromagnetic interference and temperature changes [43]. The proposed method resists such interference by dynamically adjusting $R_{k}$ through DRL:

Noise Perception: Measurement error terms in the DRL state space ($e_{r}=r_{meas}-\hat{r}$, $e_{\omega fl}=\omega_{fl,meas}-{\hat{\omega }}_{fl}$) directly reflect changes in sensor noise. When wheel speed sensor noise increases, the absolute value and fluctuation frequency of $e_{\omega fl}$ rise significantly, allowing the agent to identify noise interference via this state variable.

Observation Noise Adjustment: The agent optimizes the diagonal elements of $R_{k}$ for noise sources. For example, when the yaw rate sensor noise increases, Δ*r_r_* is increased to ensure the corresponding term for *r_meas_* in $R_{k}$ satisfies $r_{r}\geq \mathrm{cov}\left(v_{r,k}\right)$ (where $v_{r,k}$ is the actual yaw rate noise); when wheel speed sensors are affected by electromagnetic interference, $\Delta r_{\omega fl}$, $\Delta r_{\omega fr}$, $\Delta r_{\omega rl}$, $\Delta r_{\omega rr}$ increase simultaneously, reducing the weight of wheel speed observations in the Kalman gain $K_{k}$ to avoid noise contamination of state estimation.

#### 5.1.3. Robustness to Sudden Changes in Working Conditions

Vehicles often encounter sudden working condition changes during driving (e.g., sudden transition from a dry road to an icy road, where μ drops sharply from 0.85 to 0.3; or longitudinal acceleration changing abruptly from 0 to $-8\;m/s^{2}$ during emergency braking). In such scenarios, model errors and noise characteristics mutate [44], and traditional UKF with fixed Q/R tends to produce inaccurate estimates. The proposed method addresses this through the rapid response mechanism of DRL:

Perception of Sudden Changes: $\mu_{est}$ (estimated road adhesion coefficient) and $a_{x}$ (longitudinal acceleration) in the state space can quickly capture working condition changes. For example, when μ drops sharply, tire lateral force $F_{yij}$ saturates rapidly, $\mu_{est}$ decreases from 0.85 to 0.3, and $tr\left(P_{k}\right)$ rises due to increased model errors. The agent perceives the sudden change within two sampling cycles.

Rapid Adjustment Strategy: The agent triggers rapid adjustments of Q/R through the immediate feedback of the reward function. Taking the scenario of a sharp μ drop as an example provides the following:

- $Q_{k}$ adjustment: $\Delta q_{v}$, $\Delta q_{\phi }$, $\Delta q_{\omega rl}$ increase significantly, dominated by model errors in lateral velocity v (tire force saturation), coupling errors in roll angle ϕ (enhanced axle load transfer), and slip ratio errors in rear wheel speed $\omega_{rl}$;
- $R_{k}$ adjustment: $\Delta r_{a_{y}}$ increases because lateral acceleration sensors are affected by vehicle roll, causing temporary increases in measurement noise, which requires reducing their observation weight.

### 5.2. Stability Analysis

Stability is defined as the filter convergence of the estimation system (estimation error tends to a bounded value over time) and state boundedness (estimation value remains within the neighborhood of the true value). The proposed method ensures overall stability through covariance constraints of UKF and strategy stability design of DRL:

#### 5.2.1. Analysis of Filter Convergence

The core of filter convergence is to prove that the covariance $\mathrm{cov}\left({\tilde{x}}_{k}\right)=P_{k}$ of the estimation error ${\tilde{x}}_{k}={\hat{x}}_{k}-x_{k}^{true}$ is ultimately bounded, and $\lim_{k\to \infty }tr\left(P_{K}\right)<\infty$.

Positive Definiteness of Covariance Matrix: $Q_{k}\geq Q_{\min}$ and $R_{k}\geq R_{\min}$ adjusted by DRL are positive definite, and the UKF covariance update formula satisfies the following:

(27) $$P_{k}=P_{k|k-1}-K_{k}P_{zz}K_{k}^{T}$$

Since $P_{k|k-1}$ (including $Q_{k}$) and $P_{zz}$ (including $P_{zz}$) are both positive definite, $P_{k}$ remains a positive definite matrix, satisfying the basic condition to avoid filter divergence.

Proof of Covariance Boundedness: Construct a Lyapunov candidate function $V_{k}=tr\left(P_{k}\right)$, in which it needs to be proven that $V_{k}$ is ultimately bounded as follows:

(28) $$V_{k|k-1}=tr\left(\sum_{i=0}^{16}W_{c}^{i}x_{i}^{k|k-1}x_{i}^{k|k-1,T}+Q_{k}\right)\leq C_{1}+tr\left(Q_{k}\right)$$

(29) $$V_{k}=tr\left(P_{k|k-1}\right)-tr\left(K_{k}P_{zz}K_{k}^{T}\right)\leq V_{k|k-1}$$

where $C_{1}=tr\left(\sum_{i=0}^{16}W_{c}^{i}x_{i}^{k|k-1}x_{i}^{k|k-1,T}\right)$ is bounded (as the physical state $x_{k}$ is bounded). Additionally, since the DRL reward function includes the $-tr\left(P_{k}\right)$ term, the agent tends to keep $tr\left(P_{k}\right)$ within a small range, so $V_{k}$ is ultimately bounded, i.e., $\lim_{k\to \infty }tr\left(P_{K}\right)\leq C_{2}$ (where $C_{2}$ is a constant).

Convergence of Estimation Error: From the Chebyshev inequality, for any ε>0, there exists the following:

(30) $$\mathrm{Pr}ob\left(\left\|{\tilde{x}}_{k}\right\|>\varepsilon \right)\leq \frac{tr\left(P_{k}\right)}{\varepsilon^{2}}$$

Since $tr\left(P_{k}\right)$ is bounded, the probability that $\left\|{\tilde{x}}_{k}\right\|$ exceeds ε tends to 0, meaning the estimation error converges to the neighborhood of the true value.

#### 5.2.2. Analysis of State Estimation Boundedness

State estimation boundedness requires proving that ${\hat{x}}_{k}$ always remains within $x_{k}^{true}+\Delta$ (where Δ is a bounded neighborhood), combining physical constraints of the 8-DOF model and strategy constraints of DRL:

Physical Constraint Boundaries: Vehicle states have physical limits. The state transition of UKF is based on the 8-DOF model, so the predicted state ${\hat{x}}_{k|k-1}$ is naturally subject to physical constraints, preventing estimation values from exceeding reasonable ranges.

DRL Strategy Constraints: The action space of DRL limits the value ranges of $Q_{k}$ and $R_{k}$ through a clip function ($Q_{k}\in \left[Q_{\min},Q_{\max}\right]$, $R_{k}\in \left[R_{\min},R_{\max}\right]$), avoiding predicted state divergence due to excessively large $Q_{k}$ or over-correction of predictions due to excessively small $R_{k}$.

#### 5.2.3. Analysis of DRL Training Stability

The strategy stability of DRL directly affects the adjustment smoothness of $Q_{k}$ and $R_{k}$, which in turn influences UKF stability. The proposed method ensures stable DRL training through the following designs [45]:

Strategy Update Constraints: The PPO algorithm limits the magnitude of strategy updates by clipping the objective function (ε=0.2), keeping the KL divergence $KL\left(\pi_{\theta_{old}}||\pi_{\theta }\right)\leq 0.01$ between old and new strategies to avoid severe fluctuations in $Q_{k}$/$R_{k}$ caused by sudden changes in strategies.

Gradient Stabilization Measures: The Actor and Critic networks adopt gradient clipping (maximum norm 1.0) and learning rate cosine annealing to prevent gradient explosion or training oscillations caused by excessively large learning rates. In the late training stage, the gradient norm stabilizes at 0.3~0.5, and the learning rate drops to $1\times 10^{-5}$, with the strategy converging to the optimal solution.

Experience Replay Optimization: The replay buffer combines uniform random sampling and prioritized sampling to balance sample diversity and weights of important samples, avoiding strategy overfitting due to sample correlation.

### 5.3. Summary

The robustness and stability of the proposed estimation method are achieved through multi-dimensional designs:

Robustness: DRL perceives model perturbations, noise interference, and sudden working condition changes through the state space, dynamically adjusting $Q_{k}$/$R_{k}$ to enhance the ability to resist various interferences;

Stability: The boundedness of UKF covariance and strategy constraints of DRL jointly ensure that estimation errors converge and states are bounded, with no risk of filter divergence. Stability measures in DRL training ensure smooth adjustments of $Q_{k}$/$R_{k}$.

These characteristics provide reliable guarantees for the practical engineering application of this estimation method, especially in complex and variable vehicle driving scenarios.

## 6. Experimental Verification and Result Analysis

To verify the effectiveness, accuracy, and real-time performance of the proposed method for estimating vehicle sideslip angle that integrates the UKF with Deep Reinforcement Learning (DRL) (hereinafter referred to as the DRL-UKF method), virtual simulation verification and real-vehicle test verification were conducted, respectively. The virtual simulation was based on the Carsim/Simulink co-simulation platform, while the real test was carried out using a four-wheel independent drive electric vehicle as the prototype. The specific verification process is as follows.

### 6.1. Virtual Simulation Verification

#### 6.1.1. Simulation Platform and Test Setup

To verify the estimation effect of the vehicle’s sideslip angle at the center of mass proposed in this paper, a Carsim/Simulink co-simulation platform was built. The values output by Carsim were used as reference values to verify the effectiveness of the observer proposed in this study. The vehicle parameters are shown in Table 4.

Three typical driving maneuvers—slalom, double-lane change, and sinusoidal steering—were selected for simulation. Under each maneuver, the road adhesion coefficients were set to 0.2 (low-adhesion road) and 0.8 (high-adhesion road), respectively. The vehicle speed was set according to the requirements of each maneuver: 80 km/h for the slalom maneuver, 100 km/h for the double-lane-change maneuver, and 120 km/h for the sinusoidal steering maneuver. The traditional UKF method was used as a comparative benchmark in the simulation. The estimation performance of the DRL-UKF method was evaluated by analyzing the error between the estimated value of the sideslip angle and the true value output by the Carsim model.

#### 6.1.2. Simulation Results of Slalom Maneuver

**Figure 7:**
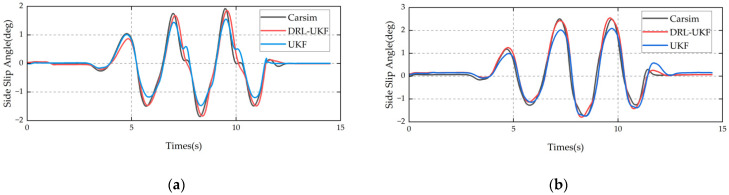
Comparison of slalom test results: (**a**) adhesion coefficient 0.2; (**b**) adhesion coefficient 0.8.

The slalom test can reflect the comfort and safety of a vehicle during continuous steering, and is often used to evaluate the vehicle’s handling performance. Figure 7 shows the comparison curves of sideslip angle data under the slalom maneuver. The results indicate the following:

When the road adhesion coefficient is 0.2, the Mean Absolute Error (MAE) of the DRL-UKF method is 0.11 deg, while that of the traditional UKF method is 0.115 deg; the RMSE of the DRL-UKF method is 0.169 deg, compared with 0.173 deg of the traditional UKF method.

When the adhesion coefficient is 0.8, the MAE of the DRL-UKF method is 0.132 deg, whereas that of the traditional UKF method is 0.171 deg; the RMSE of the DRL-UKF method is 0.19 deg, in contrast to an RMSE of 0.225 deg for the traditional UKF method.

The DRL-UKF method maintains a relatively low estimation error under the slalom maneuver, demonstrating its excellent dynamic tracking performance.

#### 6.1.3. Simulation Results of Double-Lane-Change Maneuver

**Figure 8:**
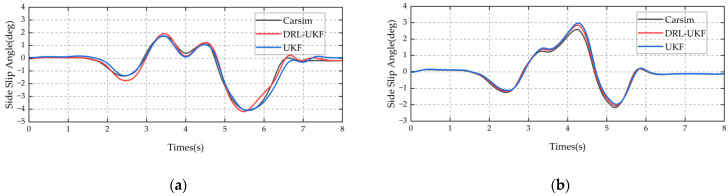
Comparison of double-lane-change results: (**a**) adhesion coefficient 0.2; (**b**) adhesion coefficient 0.8.

The double-lane-change maneuver is used to verify the effect of state estimation during vehicle steering, with the simulation speed set to 120 km/h. Figure 8 shows the comparison of the estimation results of the two methods under this maneuver. The results indicate that when the road adhesion coefficient is 0.2, the MAE of the DRL-UKF method is 0.154 deg, while that of the traditional UKF method is 0.182 deg; the RMSE of the DRL-UKF method is 0.215 deg, compared with 0.249 deg of the traditional UKF method. When the adhesion coefficient is 0.8, the MAE of the DRL-UKF method is 0.064 deg, whereas that of the traditional UKF method is 0.108 deg; the RMSE of the DRL-UKF method is 0.1 deg, in contrast to an RMSE of 0.169 deg for the traditional UKF method. The DRL-UKF method shows a significant improvement in estimation accuracy under high-speed lane change conditions, indicating that it has stronger adaptability to complex steering actions.

#### 6.1.4. Simulation Results of Sinusoidal Maneuver

**Figure 9:**
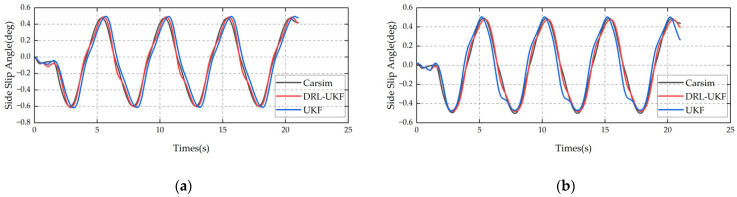
Comparison of sinusoidal steering results: (**a**) adhesion coefficient 0.2; (**b**) adhesion coefficient 0.8.

In the sinusoidal maneuver, the vehicle performs continuous sinusoidal steering, with the simulation speed set to 80 km/h. Figure 9 shows the comparison of the estimation results of the two methods. The results indicate that when the road adhesion coefficient is 0.2, the MAE of the DRL-UKF method is 0.034 deg, while that of the traditional UKF method is 0.088 deg; the RMSE of the DRL-UKF method is 0.041 deg, compared with an RMSE of 0.098 deg for the traditional UKF method. When the adhesion coefficient is 0.8, the MAE of the DRL-UKF method is 0.03 deg, whereas that of the traditional UKF method is 0.071 deg; the RMSE of the DRL-UKF method is 0.036 deg, in contrast to an RMSE of 0.091 deg for the traditional UKF method. The DRL-UKF method significantly reduces the estimation error under periodic steering conditions, showing better performance.

#### 6.1.5. Summary of Simulation Results

The simulation data from the three typical operating conditions show that both the MAE and RMSE of the vehicle sideslip angle estimated by the DRL-UKF method are lower than those of the traditional UKF method. Moreover, the proposed method can maintain high estimation accuracy under different road adhesion coefficients and different steering modes, which effectively verifies its effectiveness and accuracy. The comparison platform for the percentages of performance improvement under various operating conditions is shown in Table 5.

### 6.2. Real-Vehicle Test Verification

#### 6.2.1. Test Platform and Vehicle Parameters

**Figure 10:**
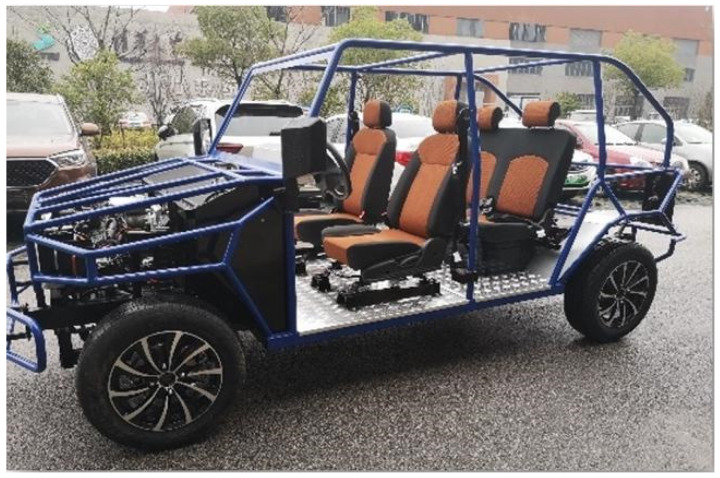
The test vehicle.

A real test platform was built using a four-wheel independent drive battery electric vehicle as the test prototype. The basic parameters of the test vehicle are shown in Table 6. The vehicle adopts four-wheel independent drive, with tires of model 195/60R16 and a tire pressure of 250 kPa. The sensors installed on the test vehicle are the inertial navigation integrated positioning system RT3000 and the steering wheel sensor MSW DTI. Among them, the RT3000 is used to measure signals such as vehicle speed, yaw rate, sideslip angle at the center of mass, longitudinal acceleration, and lateral acceleration; the MSW DTI is used to measure the steering wheel angle. The observation algorithm runs on the MicroAutobox II. The sensors and the MicroAutobox II are connected via a CAN bus. The test vehicle is shown in Figure 10.

#### 6.2.2. Real Test Results of Double-Lane-Change Maneuver

**Figure 11:**
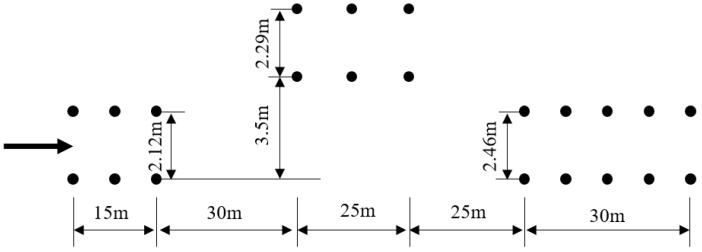
Layout diagram of double-lane-change maneuver.

The double-lane-change test site was set up in accordance with the international standard ISO 3888-1 Passenger cars: Test track for a severe lane-change maneuver—Part 1: Double-lane change [46]. The layout of the site is shown in Figure 11, with a road adhesion coefficient of 0.8. During the test, the vehicle accelerated from a standstill to 80 km/h, then maintained a constant speed, entered the double-lane-change maneuver, and the test was conducted. The corresponding working conditions in the simulation verification also use these test site parameters.

**Figure 12:**
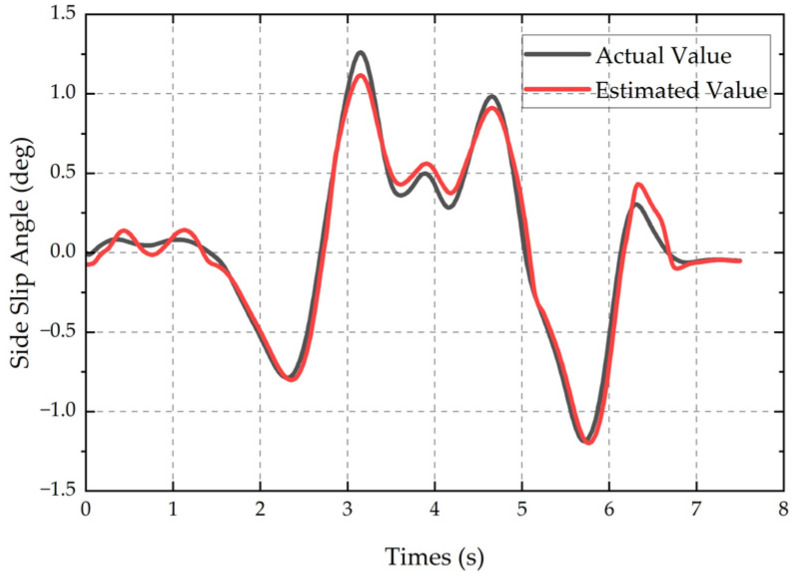
Comparison of double-lane-change test results.

The comparison results between the true value of the sideslip angle and the estimated value by the DRL-UKF method are shown in Figure 12. The results indicate that the variation trend of the estimated value is highly consistent with that of the true value, with an MAE of 0.061 deg and an RMSE of 0.075 deg, which verifies the effectiveness and real-time performance of the DRL-UKF method in real scenarios.

#### 6.2.3. Real Test Results of Slalom Maneuver

**Figure 13:**
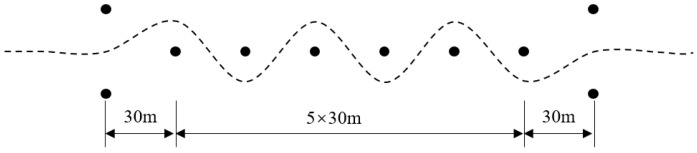
Slalom test site.

The slalom test site was set up in accordance with the Chinese national standard GB/T 6323 Test Method for Vehicle Handling and Stability—Slalom Test [47]. The layout of the site is shown in Figure 13, with a road adhesion coefficient of 0.8. The test procedure is consistent with that of the double-lane-change maneuver, where the vehicle passes through the slalom test site at a constant speed of 60 km/h. The corresponding working conditions in the simulation verification also use these test site parameters.

**Figure 14:**
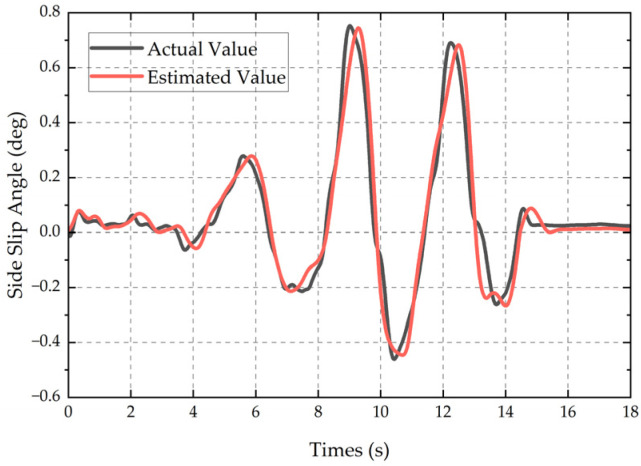
Comparison of slalom test results.

The comparison results between the estimated value and the true value of the sideslip angle are shown in Figure 14. The results indicate that the estimated value can accurately reflect the magnitude and variation trend of the true sideslip angle, with an MAE of 0.041 deg and a RMSE of 0.06 deg, which further verifies the reliability of the proposed method under real and complex working conditions.

#### 6.2.4. Summary of Test Verification Results

The real test data from the double-lane-change and slalom maneuvers indicate that the DRL-UKF method can estimate the vehicle’s sideslip angle in real time and accurately. The estimated values are in good agreement with the true values, meeting the requirements for real-time performance and accuracy in state parameter estimation during actual vehicle operation.

### 6.3. Test Conclusions

Both the virtual simulation verification and real-vehicle test verification results show that the proposed DRL-UKF method effectively improves the estimation accuracy of the vehicle’s sideslip angle by dynamically optimizing the noise parameters of UKF through DRL. Compared with the traditional UKF method, the DRL-UKF method has significantly improved the MAE and RMSE. Moreover, it exhibits strong stability and robustness under different vehicle speeds, road adhesion coefficients, and complex working conditions, providing a new solution for the accurate estimation of key vehicle state parameters, which can be popularized and applied in the fields of autonomous driving and vehicle active safety.

## 7. Conclusions

This paper focuses on the problem of high-precision estimation of vehicle sideslip angle under full operating conditions. Aiming at the shortcomings of the traditional UKF method, such as fixed noise parameters and poor adaptability to complex operating conditions, a joint estimation method integrating UKF and DRL is proposed, and systematic work on modeling, design, optimization, and verification is carried out. The main research conclusions are as follows:

1. An estimation architecture with deep integration of UKF and DRL is proposed. A “dual closed-loop collaborative” estimation framework is constructed, which is based on an 8-degree-of-freedom vehicle dynamics model and uses DRL to dynamically optimize noise parameters. This framework realizes the organic integration of physical models and data-driven methods and effectively overcomes the problem of degraded estimation performance of traditional methods under model perturbations and noise interference.
2. A DRL-based noise adaptive mechanism adapted to high-dimensional UKF is designed. The state space and action space are expanded, the tire force saturation is introduced as the basis for operating condition division, the structure of the reward function is optimized, and the PPO algorithm is used to train the policy network. This realizes the dynamic adjustment of the process noise covariance Q and the observation noise covariance R, and improves the adaptive capability of the filter.
3. The effectiveness and robustness of the proposed method are verified through virtual simulations and real-vehicle tests. Under various typical operating conditions, such as slalom, double-lane change, and sinusoidal steering, the sideslip angle estimation accuracy of the proposed DRL-UKF method is significantly better than that of the traditional UKF. Both the evaluation error and Root Mean Square Error are greatly improved, and stable estimation is still maintained under extreme scenarios such as low-adhesion roads and high-speed steering.
4. A new idea is provided for the estimation of key vehicle state parameters. This study not only realizes the deep coupling of model-driven and data-driven approaches at the method level, but also provides a promotable technical path for real-time state perception and control system design in fields such as autonomous driving and vehicle active safety.

Future work will further optimize the training efficiency and generalization ability of DRL, expand its application to more complex driving scenarios and multi-source sensor fusion, and promote the development of vehicle state estimation technology towards higher precision and stronger adaptability.

## Figures and Tables

**Table 1:** Comparison of advantages and disadvantages of different estimation methods.

Method Category	Specific Method	Advantages	Disadvantages
Early Measurement Instrument Methods	Inertial Sensor Scheme	Directly measures lateral acceleration with a simple calculation	Long-term accuracy degradation due to noise accumulation and large integration error
	GPS Combination Scheme	Dynamic correction; stable output under neutral steering conditions	Sensitive to sampling efficiency and environmental occlusion; limited by signals
Traditional Estimation Algorithms	Direct Integration Method	Small calculation load and good real-time performance	Large long-term estimation deviation caused by error accumulation
	Extended Kalman Filter (EKF)	Linearizes nonlinear systems with simple calculation; stable performance under small sideslip angle conditions; and accuracy meets basic control needs	Only applicable to linear/small sideslip angle conditions; large error in nonlinear processing
	Unscented Kalman Filter (UKF)	Avoids linearization error via unscented transformation; high accuracy in nonlinear processing (superior to EKF); and optimal performance in snake-like maneuvering on high-adhesion roads	Fixed noise covariance; poor adaptability to time-varying parameters; and high computational complexity
	Sliding Mode Observer	Stronger adaptability to load transfer and road condition changes; high robustness	Accuracy is limited by switching function design; prone to chattering
	Luenberger Observer	Stronger adaptability to load transfer and road condition changes; estimation results of second-order or generalized versions are closer to true values	Complex design; requires accurate model parameters
Data-Driven Technologies	Neural Networks, Fuzzy Logic, etc.	No need for accurate models; improves accuracy and reliability through fusion estimators	Requires massive labeled data; lack of physical constraints easily leads to unphysical results
	Piecewise Affine Estimation Method	Considers the nonlinearity of wheel lateral force saturation; high feasibility verified by experiments	Relies on piecewise model design; limited generalization ability
	Fusion of Physical Model and Data-Driven Methods (e.g., Fuzzy Logic + UKF, ANFIS + UKF)	Integrates advantages; enables effective parameter observation; and improves robustness	The fusion strategy is simple, but estimation jumps are prone to occur during the working condition transition phase
Improved Unscented Kalman Filter	Adaptive Singular Value Decomposition UKF	Real-time correction of noise covariance matrix; reduced error on complex roads; and strong anti-noise capability	Increased computational time; affected real-time performance
	Fuzzy Control + UKF	Enables adaptive adjustment of measurement noise; improved accuracy under double-lane-change conditions	Complex design; requires adjustment of fuzzy rules
	Fault-Tolerant Noise Estimator + UKF	Realizes joint estimation of side-slip angle and tire cornering stiffness	High computational complexity; requires multi-sensor fusion
Deep Reinforcement Learning (DRL)	DRL-Optimized Weights (e.g., CNN-LSTM)	Addresses insufficient generalization of traditional neural networks; controlled error growth under off-training-set conditions	Poor real-time performance; requires massive computing resources
	DRL as Model Error Compensator	Learns error patterns based on dynamic model output; reduced estimation deviation in double-lane-change tests	Relies on basic models; complex compensator training
	DRL + Fuzzy Sliding Mode Observer	Optimizes saturation function parameters; suppresses chattering; accelerates convergence speed; and reduces response lag in tire nonlinear regions	Poor real-time performance; complex parameter adjustment
	DRL + EKF	Dynamically adjusts filter gain; improves estimation stability under sudden sensor signal changes	Insufficient fusion depth; limited real-time performance
Fusion Framework Trend	UKF + DRL (Proposed Scheme)	Optimizes noise parameters via DRL; physical model constrains estimation boundaries; and achieves high accuracy and robustness across all conditions	Still in the research stage; difficult to balance real-time performance and generalization

**Table 2:** Definition of Core Variables for the 8-DOF Model.

Variables	Physical Meaning	Unit
u	Longitudinal velocity of the vehicle’s center of mass	m/s
v	Lateral velocity of the vehicle’s center of mass	m/s
r	Vehicle yaw rate	rad/s
ϕ	Vehicle roll angle	rad
$\dot{\phi }$	Vehicle roll angular velocity	rad/s
m	Total vehicle mass	kg
$I_{z}$	Vehicle moment of inertia about the *z*-axis	kg·m^2^
$I_{x}$	Vehicle moment of inertia about the *x*-axis	kg·m^2^
$l_{f}$/$l_{r}$	Distance from the center of mass to the front/rear axle	m
B	Track width	m
$F_{y,est}$	Estimated value of wheel lateral force	N
$\omega_{fl}$	Rotational angular velocity of the front-left wheel	rad/s
$\omega_{fr}$	Rotational angular velocity of the front-right wheel	rad/s
$\omega_{rl}$	Rotational angular velocity of the rear-left wheel	rad/s
$\omega_{rr}$	Rotational angular velocity of the rear-right wheel	rad/s
$F_{xij}$	Wheel longitudinal force (i=f/r,j=l/r)	N
$F_{yij}$	Wheel lateral force (i=f/r,j=l/r)	N
$F_{zij}$	Wheel vertical load (i=f/r,j=l/r)	N
$T_{dij}$	Wheel driving torque (i=f/r,j=l/r, 4WD distribution)	N·m
$T_{bij}$	Wheel braking torque (i=f/r,j=l/r)	N·m
$R_{w}$	Wheel rolling radius	m
$S_{F}$	Tire force saturation degree ($F_{y,actual}/F_{y,\max}$)	*-*

**Table 3:** Main vehicle parameters.

Parameter Name	Value	Unit
Vehicle mass *m*	1499.6	kg
Wheelbase *L*	2700	mm
The distance from the center to the front axis *l_f_*	1267	mm
The distance from the center to the rear axis *l_r_*	1433	mm
The height from the center to the ground *h_g_*	551	mm
Rolling radius of tire *r*	320.2	mm
Front axle wheel base *B_f_*	1530	mm
Rear axle wheel base *B_r_*	1530	mm
Roll moment of inertia *I_x_*	483.4	kg⋅m^2^
Pitch moment of inertia *I_y_*	1528.6	kg⋅m^2^
Moment of inertia of the yaw *I_z_*	1685.2	kg⋅m^2^

**Table 4:** Parameters of the Carsim vehicle.

Item	Value	Unit
Mass	1499.6	kg
Wheelbase	2700	mm
Distance from the center of mass to the front/rear axle	1267/1433	mm
Height of the center of mass	551	mm
Tire radius	320.2	mm
Front/rear track width	1530/1530	mm
Moment of inertia (Izz)	1685.2	kg⋅m^2^

**Table 5:** Comparison platform with the percentage of performance improvement.

Project		Traditional UKF Method (Deg)	DRL-UKF Method (Deg)	Percentage of Performance Improvement (%)
Slalom Test Adhesion Coefficient 0.2	Mean Absolute Error (MAE)	0.115	0.11	4.35
	Root Mean Square Error (RMSE)	0.173	0.169	2.31
Slalom Test Adhesion Coefficient 0.8	Mean Absolute Error (MAE)	0.171	0.132	22.81
	Root Mean Square Error (RMSE)	0.225	0.19	15.56
Double-Lane-Change Test Adhesion Coefficient 0.2	Mean Absolute Error (MAE)	0.182	0.154	15.38
	Root Mean Square Error (RMSE)	0.249	0.215	13.65
Double-Lane-Change Test Adhesion Coefficient 0.8	Mean Absolute Error (MAE)	0.108	0.064	40.74
	Root Mean Square Error (RMSE)	0.169	0.1	40.83
Sinusoidal Steering Test Adhesion Coefficient 0.2	Mean Absolute Error (MAE)	0.088	0.034	61.36
	Root Mean Square Error (RMSE)	0.098	0.041	58.16
Sinusoidal Steering Test Adhesion Coefficient 0.8	Mean Absolute Error (MAE)	0.071	0.03	57.75
	Root Mean Square Error (RMSE)	0.091	0.036	60.44

**Table 6:** Main vehicle parameters.

Parameter Name	Value	Unit
Vehicle mass *m*	1499.6	kg
Wheelbase *L*	2700	mm
The distance from the center to the front axis *l_f_*	1267	mm
The distance from the center to the rear axis *l_r_*	1433	mm
The height from the center to the ground *h_g_*	551	mm
Rolling radius of tire *r*	320.2	mm
Front axle wheel base *B_f_*	1530	mm
Rear axle wheel base *B_r_*	1530	mm
Roll moment of inertia *I_x_*	483.4	kg⋅m^2^
Pitch moment of inertia *I_y_*	1528.6	kg⋅m^2^
Moment of inertia of the yaw *I_z_*	1685.2	kg⋅m^2^

## Data Availability

The original contributions presented in this study are included in the Supplementary Materials.

## Supplementary Materials

The following supporting information can be downloaded at: https://www.mdpi.com/article/10.3390/s25247489/s1, Table S1. Test Data under Steady-State Cornering Condition; Table S2. 8-DOF Data under Steady-State Cornering Condition; Table S3. Test Data under Double-Lane-Change Condition; Table S4. 8-DOF Data under Double-Lane-Change Condition; Table S5. Test Data of Central Area Steering Condition; Table S6. 8-DOF Data of Central Area Steering Condition; Table S7. Test result Data of the Slalom Test at an Adhesion Coefficient of 0.2; Table S8. Test Result Data of the Slalom Test at an Adhesion Coefficient of 0.8; Table S9. Test Result Data of the Double-Lane-Change Test at an Adhesion Coefficient of 0.2; Table S10. Test Result Data of the Double-Lane-Change Test at an Adhesion Coefficient of 0.8; Table S11. Test Result Data of the Sinusoidal Steering Test at an Adhesion Coefficient of 0.2; Table S12. Test Result Data of the Sinusoidal Steering Test at an Adhesion Coefficient of 0.8; Table S13. Slalom Test Result Data; Table S14. Double-Lane-Change Test Result Data.
