# Supplementary material for: Vehicle Sideslip Angle Estimation Using Deep Reinforcement Learning Combined with Unscented Kalman Filter
Source: Sensors (Basel). 2025 Dec 9;25(24):7489. doi: 10.3390/s25247489 (PMC12736524; doi:10.3390/s25247489)
Supplement: Supplementary file 1 [file sensors-25-07489-s001.zip › sensors-4007953-supplementary.pdf]

Table S1. Test Data under Steady-State Cornering Condition

Time	ay	rollangle
0.01	-0.565	-0.26915
0.02	0.71	-0.03844
0.03	-0.515	0.166837
0.04	0.35	-0.44017
0.05	-0.055	-0.12481
0.06	0.175	-0.06352
0.07	-0.315	-0.08173
0.08	-0.305	0.183028
0.09	0.785	-0.07157
0.1	0.235	0.131599
0.11	0.27	-0.1539
0.12	-1.11	0.211761
0.13	0.53	-0.20564
0.14	0.5	-0.0709
0.15	0.125	0.097708
0.16	-0.27	0.533327
0.17	-0.485	0.220331
0.18	0.89	0.437717
0.19	-0.355	0.259247
0.2	-0.23	0.045552
0.21	0.245	0.039398
0.22	0.05	0.305766
0.23	0.365	-0.14367
0.24	-0.5	0.524688
0.25	0.04	0.31435
0.26	0.65	0.231181
0.27	-1.105	0.127554
0.28	0.76	0.025239
0.29	-0.25	-0.04387
0.3	0.365	0.004123
0.31	0.09	-0.01969
0.32	-0.655	0.085349
0.33	0.52	0.124272
0.34	-0.005	-0.16492
0.35	-0.61	0.253201
0.36	1.385	0.245387
0.37	-1.08	0.230148
0.38	0.285	-0.18148
0.39	0.365	0.126139
0.4	-0.635	0.043579
0.41	0.55	-0.125
0.42	0.43	-0.07652
0.43	-0.33	0.196682
0.44	0.205	0.042468

0.45	-0.09	0.104919
0.46	-0.23	-0.06987
0.47	0.58	0.192311
0.48	-0.08	0.122095
0.49	-0.54	0.180713
0.5	-0.54	-0.00442
0.51	0.755	0.01241
0.52	-0.55	-0.11549
0.53	-0.01	-0.18305
0.54	-0.145	0.138924
0.55	-0.295	-0.3667
0.56	0.37	-0.12558
0.57	-0.185	-0.24384
0.58	0.29	-0.0042
0.59	0.265	0.091978
0.6	-0.995	-0.16293
0.61	0.865	0.150715
0.62	-0.415	0.121602
0.63	-0.37	0.184975
0.64	0.04	-0.19308
0.65	0.22	0.25295
0.66	0.34	0.00245
0.67	-0.36	0.087014
0.68	-0.54	-0.00121
0.69	0.61	0.132379
0.7	-0.045	0.271045
0.71	0.115	-0.21948
0.72	0.045	-0.12944
0.73	-0.45	0.430623
0.74	0.25	-0.01792
0.75	0.235	-0.05134
0.76	-0.9	-0.32015
0.77	1.35	0.110147
0.78	-0.82	0.099308
0.79	0.095	0.264793
0.8	0.43	-0.08067
0.81	0.03	0.304317
0.82	-0.105	-0.15251
0.83	0.01	-0.03276
0.84	-0.035	-0.0781
0.85	-0.37	0.05617
0.86	0.91	0.029508
0.87	-0.15	0.034168
0.88	-0.535	0.035014
0.89	0.515	0.301861
0.9	-0.165	-0.16239
0.91	0.03	-0.10614
0.92	-0.23	0.362063

0.93	0.565	-0.12176
0.94	-0.28	0.092615
0.95	-0.345	0.339666
0.96	0.61	-0.25706
0.97	-0.025	0.174899
0.98	-0.78	-0.24427
0.99	0.525	0.026881
1	-0.12	-0.01643
1.01	0.34	0.193154
1.02	-0.355	0.114323
1.03	0.27	-0.26671
1.04	-0.34	-0.0495
1.05	-0.15	0.111329
1.06	0.225	0.233331
1.07	0.15	-0.14526
1.08	-0.295	0.068582
1.09	0.585	-0.31672
1.1	-0.62	-0.00124
1.11	0.2	0.180323
1.12	0.24	0.355649
1.13	-0.315	0.099456
1.14	0.33	-0.00599
1.15	0.105	0.260262
1.16	-0.165	0.209318
1.17	0.03	-0.27236
1.18	-0.19	0.077899
1.19	0.025	0.009308
1.2	0.35	-0.03349
1.21	-0.09	-0.08094
1.22	-0.225	-0.10097
1.23	0.36	0.214949
1.24	-0.03	0.131086
1.25	-0.495	-0.16986
1.26	0.705	0.326005
1.27	-0.59	0.174833
1.28	0.465	0.136923
1.29	-0.26	-0.12618
1.3	0.33	-0.12425
1.31	-0.725	-0.08846
1.32	0.815	0.510402
1.33	-0.03	0.282237
1.34	-0.54	0.159178
1.35	0.405	-0.2025
1.36	0.22	-0.03683
1.37	-0.29	-0.04717
1.38	-0.08	-0.01164
1.39	0.07	0.0456
1.4	-0.02	-0.15321

1.41	0.42	-0.25519
1.42	-0.215	-0.23168
1.43	0.205	-0.03771
1.44	-0.33	0.167665
1.45	0.275	-0.04899
1.46	-0.475	0.053614
1.47	0.765	-0.08015
1.48	-0.52	-0.33623
1.49	0.165	-0.21184
1.5	0.57	-0.13012
1.51	-0.27	-0.20417
1.52	-0.105	-0.27157
1.53	0.265	0.149082
1.54	0.035	-0.0023
1.55	0.33	-0.09694
1.56	-0.105	0.133049
1.57	0.61	-0.11244
1.58	-0.57	-0.17335
1.59	0.305	-0.07974
1.6	0.18	0.037674
1.61	-0.605	-0.13515
1.62	0.65	-0.10446
1.63	0.49	0.176181
1.64	-0.6	-0.0043
1.65	0.14	0.144614
1.66	0.07	0.07565
1.67	-0.225	-0.1345
1.68	0.545	0.001821
1.69	-0.215	-0.15482
1.7	0.06	0.22273
1.71	0.215	0.218862
1.72	0.265	0.102685
1.73	-0.235	-0.20044
1.74	0.155	0.449614
1.75	-0.21	-0.24889
1.76	0.265	-0.05456
1.77	-0.07	0.470801
1.78	0.775	-0.18616
1.79	-0.71	0.219996
1.8	0.115	0.147731
1.81	0.27	0.205433
1.82	0.285	-0.20047
1.83	-0.39	0.264019
1.84	-0.07	-0.12007
1.85	0.275	0.101281
1.86	0.51	-0.23477
1.87	-0.325	0.473003
1.88	-0.05	0.166431

1.89	-0.21	-0.21919
1.9	0.475	0.029941
1.91	0.23	-0.22941
1.92	0.03	-0.01375
1.93	-0.15	-0.01766
1.94	0.335	-0.17494
1.95	0.045	0.077248
1.96	-0.615	0.186864
1.97	1.2	0.061691
1.98	0.095	-0.10056
1.99	-0.835	0.128678
2	0.935	-0.04349
2.01	-0.635	0.031404
2.02	0.35	-0.08477
2.03	0.07	-0.13818
2.04	0.08	0.255136
2.05	0.38	-0.0883
2.06	-0.015	-0.36761
2.07	-0.515	-0.47553
2.08	0.455	-0.01079
2.09	0.135	-0.49305
2.1	-0.375	0.086361
2.11	0.37	-0.41244
2.12	0.205	-0.44754
2.13	0.1	-0.15518
2.14	0.135	0.164742
2.15	-0.565	0.122461
2.16	0.83	-0.09816
2.17	-0.36	0.029411
2.18	0.09	0.043171
2.19	0.155	-0.10894
2.2	0.08	-0.01795
2.21	0.64	-0.09511
2.22	-0.585	-0.16056
2.23	0.525	0.221403
2.24	-0.47	0.143458
2.25	0.43	-0.26221
2.26	0.18	-0.2435
2.27	0.065	-0.14018
2.28	0.125	0.155639
2.29	0.2	0.003282
2.3	-0.285	-0.02768
2.31	0.27	-0.11881
2.32	0.355	0.124931
2.33	-0.225	0.101465
2.34	0.385	0.267216
2.35	-0.03	-0.33475
2.36	-0.37	-0.0035

2.37	0.88	-0.46898
2.38	-0.885	0.351972
2.39	0.37	0.09259
2.4	0.48	-0.08908
2.41	-0.27	-0.12041
2.42	0.325	-0.34115
2.43	-0.08	0.280512
2.44	0.12	0.209065
2.45	-0.27	0.41637
2.46	0.58	0.186229
2.47	-0.175	-0.30114
2.48	0.28	0.349621
2.49	-0.18	-0.1249
2.5	-0.41	0.287337
2.51	1.015	0.170711
2.52	-0.635	0.187504
2.53	0.12	0.141432
2.54	0.41	0.207625
2.55	-0.25	0.078965
2.56	-0.11	0.320828
2.57	0.305	-0.08756
2.58	0.01	0.224101
2.59	-0.11	0.395112
2.6	0.095	-0.59099
2.61	-0.13	-0.00477
2.62	0.1	0.035317
2.63	0.045	-0.07782
2.64	0.125	-0.05133
2.65	-0.2	0.22333
2.66	0.235	0.317965
2.67	-0.195	0.109204
2.68	0.37	0.30482
2.69	-0.285	-0.33609
2.7	0.165	-0.03747
2.71	-0.06	-0.22999
2.72	0.155	-0.10827
2.73	-0.115	0.022023
2.74	0.125	0.050893
2.75	0.555	-0.15368
2.76	-0.24	0.096851
2.77	-0.04	0.594161
2.78	-0.225	0.16195
2.79	0.755	0.024579
2.8	-0.595	0.139273
2.81	-0.08	-0.08423
2.82	1.035	0.084589
2.83	-0.645	0.173729
2.84	0.14	-0.08932

2.85	0.215	-0.28894
2.86	-0.555	-0.14251
2.87	0.81	0.232276
2.88	-0.615	-0.01801
2.89	0.16	0.290856
2.9	0.155	-0.10756
2.91	0.04	-0.05041
2.92	0.075	0.205332
2.93	-0.59	-0.06198
2.94	0.73	0.029045
2.95	-0.19	0.264026
2.96	-0.07	-0.1093
2.97	0.265	-0.24017
2.98	-0.25	0.120005
2.99	-0.215	-0.03473
3	1.04	0.01478
3.01	-0.825	-0.06191
3.02	0.245	0.211864
3.03	0.215	0.012712
3.04	-0.235	0.125754
3.05	0.215	-0.10995
3.06	0.565	-0.12613
3.07	-0.505	0.027762
3.08	-0.255	0.211136
3.09	0.865	0.397379
3.1	-0.57	-0.14246
3.11	0.38	-0.15864
3.12	0.19	-0.03833
3.13	-0.205	0.075352
3.14	-0.175	0.088901
3.15	0.78	0.220711
3.16	-0.545	0.027067
3.17	0.095	-0.11409
3.18	0.145	0.081589
3.19	0.55	0.157729
3.2	-0.62	0.076158
3.21	-0.14	-0.09163
3.22	0.365	-0.03893
3.23	0.385	0.03456
3.24	-0.085	-0.07163
3.25	0.085	-0.16222
3.26	-0.295	0.156079
3.27	0.08	-0.31673
3.28	0.105	0.053222
3.29	-0.045	-0.20916
3.3	0.015	-0.1098
3.31	0.23	0.477392
3.32	0.135	0.298719

3.33	0.13	0.000404
3.34	-0.245	0.152855
3.35	0.405	-0.043
3.36	-0.275	-0.43065
3.37	0.225	-0.29437
3.38	0.46	0.268016
3.39	-0.845	-0.05472
3.4	1.085	-0.03876
3.41	-0.25	-0.05612
3.42	-0.27	-0.15465
3.43	0.16	-0.52298
3.44	0.715	-0.35485
3.45	-0.6	0.059008
3.46	0.145	-0.05223
3.47	0.4	0.102195
3.48	-0.475	0.005272
3.49	0.555	0.179059
3.5	-0.51	-0.09537
3.51	0.315	-0.11815
3.52	0.145	0.547777
3.53	-0.295	0.201659
3.54	0.505	0.03574
3.55	0.255	0.279935
3.56	-0.775	0.385468
3.57	0.3	0.19162
3.58	0.41	0.008865
3.59	-0.685	-0.40247
3.6	0.855	0.050706
3.61	-0.165	0.196778
3.62	-0.295	-0.05123
3.63	0.47	-0.00627
3.64	-0.225	-0.30415
3.65	0.51	-0.20296
3.66	-0.435	-0.22554
3.67	-0.1	0.131918
3.68	0.48	0.497291
3.69	-0.455	0.192321
3.7	0.57	0.138959
3.71	-0.32	0.007527
3.72	-0.005	-0.11297
3.73	-0.035	-0.23412
3.74	-0.035	0.126037
3.75	0.94	-0.1401
3.76	-1.055	-0.14741
3.77	0.46	0.141775
3.78	0.085	-0.03661
3.79	-0.39	0.217656
3.8	0.295	-0.00829

3.81	-0.14	0.047439
3.82	0.715	-0.00863
3.83	-0.835	0.541089
3.84	0.075	-0.1976
3.85	0.66	-0.27142
3.86	-0.17	-0.24128
3.87	-0.18	0.08683
3.88	0.135	-0.05508
3.89	0.305	-0.21993
3.9	0.235	0.054338
3.91	-0.385	0.313261
3.92	0.45	0.192503
3.93	-0.075	0.107121
3.94	-0.115	-0.13529
3.95	0.29	-0.17832
3.96	0.23	-0.17912
3.97	-0.245	0.028202
3.98	0.045	0.082044
3.99	0.12	0.141468
4	-0.6	0.208928
4.01	1.075	0.208468
4.02	-0.67	0.079988
4.03	0.48	-0.18072
4.04	0.18	0.065237
4.05	-0.585	-0.02987
4.06	0.56	0.165134
4.07	-0.515	0.01639
4.08	0.28	0.046255
4.09	0.34	0.10213
4.1	-0.3	-0.27105
4.11	-0.08	0.356844
4.12	0.19	0.356526
4.13	0.145	0.053671
4.14	-0.5	0.015308
4.15	0.855	0.049606
4.16	-0.675	0.211894
4.17	0.515	-0.03538
4.18	0.22	-0.19259
4.19	-0.65	-0.32606
4.2	0.6	0.190215
4.21	-0.015	-0.05692
4.22	-0.08	0.029193
4.23	0.28	0.192573
4.24	0.245	0.056471
4.25	-0.21	-0.35268
4.26	-0.095	0.488404
4.27	0.24	0.186732
4.28	-0.045	-0.28389

4.29	0.05	-0.26008
4.3	0.11	-0.1271
4.31	-0.085	-0.03016
4.32	0.215	0.189549
4.33	-0.24	0.023139
4.34	0.27	-0.09981
4.35	-0.3	0.026774
4.36	0.245	0.267024
4.37	0.06	-0.0711
4.38	0.03	-0.10924
4.39	-0.205	0.005369
4.4	0.415	0.392742
4.41	-0.71	0.047105
4.42	0.375	-0.3574
4.43	0.065	-0.21539
4.44	0.1	0.055848
4.45	-0.165	-0.2166
4.46	0.675	-0.1627
4.47	-0.685	-0.24458
4.48	-0.055	0.149636
4.49	0.37	0.220051
4.5	-0.13	-0.004
4.51	0.355	0.140581
4.52	-0.63	-0.15544
4.53	1.025	-0.05398
4.54	-1.08	-0.06663
4.55	0.635	-0.10767
4.56	-0.38	-0.29564
4.57	0.67	-0.00157
4.58	-0.32	-0.17414
4.59	0.265	-0.1914
4.6	-0.17	-0.22225
4.61	0.225	0.113357
4.62	0.32	-0.09809
4.63	0.045	-0.10638
4.64	-0.255	-0.11758
4.65	0.42	0.054745
4.66	0.625	-0.2379
4.67	-0.48	-0.40181
4.68	0.505	0.220935
4.69	0.015	0.324301
4.7	-0.105	-0.14938
4.71	0.655	0.048681
4.72	0.38	-0.19962
4.73	-0.14	-0.15712
4.74	0.58	0.290868
4.75	0.155	0.226226
4.76	-0.295	0.099192

4.77	0.89	-0.11774
4.78	0.21	0.074858
4.79	0.495	-0.11937
4.8	0.19	0.402507
4.81	0.4	-0.07968
4.82	0.68	0.430676
4.83	-0.13	0.15233
4.84	0.56	0.278724
4.85	0.655	0.275707
4.86	0.43	-0.05174
4.87	0.7	0.216885
4.88	0.6	0.284167
4.89	0.025	0.282444
4.9	0.845	0.220358
4.91	0.735	0.068273
4.92	0.275	0.34306
4.93	1.265	0.01771
4.94	0.45	0.222223
4.95	0.495	0.243459
4.96	1.04	0.080709
4.97	0.07	0.128417
4.98	1.27	0.347512
4.99	0.34	0.6212
5	1.135	0.018948
5.01	0.98	0.48374
5.02	0.52	0.204411
5.03	0.51	0.23819
5.04	1.465	0.42335
5.05	0.385	0.287614
5.06	0.92	0.280101
5.07	0.95	0.253297
5.08	1.16	0.056894
5.09	0.75	0.158123
5.1	0.71	0.443111
5.11	1.045	0.369082
5.12	0.835	0.425538
5.13	1.5	0.219563
5.14	0.445	0.64087
5.15	1	0.372027
5.16	1.41	0.101085
5.17	0.955	0.379755
5.18	1.185	0.212676
5.19	1.285	0.412394
5.2	0.665	0.384875
5.21	0.995	0.074972
5.22	1.605	0.624858
5.23	1.455	0.375327
5.24	1.005	0.245606

5.25	0.94	0.472511
5.26	1.13	0.429064
5.27	1.435	0.559997
5.28	1.76	0.189258
5.29	0.745	0.937268
5.3	1.535	0.55438
5.31	1.615	0.585004
5.32	1.005	0.290095
5.33	1.35	0.089254
5.34	1.345	0.746594
5.35	1.65	0.342341
5.36	1.295	0.306096
5.37	2.085	0.412633
5.38	1.275	0.413643
5.39	0.63	0.726035
5.4	2.06	0.613745
5.41	1.665	0.873892
5.42	1.72	0.190557
5.43	1.155	0.807605
5.44	2.02	0.54826
5.45	1.69	0.542308
5.46	1.515	0.945657
5.47	1.56	0.521083
5.48	1.8	0.558257
5.49	2.13	0.430641
5.5	1.67	0.833852
5.51	1.93	0.848699
5.52	1.73	0.744756
5.53	1.62	0.865403
5.54	2.125	0.504213
5.55	2.24	0.81748
5.56	1.285	1.119551
5.57	2.335	0.531822
5.58	2.265	0.58806
5.59	1.935	1.046715
5.6	1.4	1.013623
5.61	2.73	0.566527
5.62	1.6	0.722813
5.63	2.46	0.814886
5.64	2.485	0.797439
5.65	1.21	0.883354
5.66	2.365	0.739621
5.67	2.17	0.601099
5.68	2.31	0.945233
5.69	1.84	0.848219
5.7	2.565	1.006667
5.71	1.675	0.69571
5.72	2.84	0.85091

5.73	2.215	1.172124
5.74	2.42	0.605733
5.75	1.65	0.709916
5.76	2.725	0.681168
5.77	2.305	0.631081
5.78	2.165	0.704637
5.79	2.755	0.992298
5.8	2.105	1.194297
5.81	2.655	1.093187
5.82	2.265	1.061941
5.83	2.765	0.72896
5.84	2.44	1.082313
5.85	2.34	1.145638
5.86	3.255	0.809295
5.87	1.715	0.928842
5.88	3.2	0.893338
5.89	2.49	1.199172
5.9	2.165	0.783774
5.91	2.93	0.890873
5.92	3.32	1.027238
5.93	2.255	1.333238
5.94	2.695	1.000934
5.95	2.75	0.960423
5.96	2.555	1.386755
5.97	2.685	0.971875
5.98	3.16	1.09797
5.99	2.28	1.100774
6	2.995	1.182307
6.01	2.895	1.137799
6.02	3.18	0.950278
6.03	2.38	0.756005
6.04	3.185	1.020377
6.05	2.705	1.330421
6.06	3.075	1.231663
6.07	2.775	1.060718
6.08	2.86	0.973948
6.09	3.12	0.808976
6.1	3.035	0.916448
6.11	3.14	1.319884
6.12	2.71	1.245627
6.13	2.99	1.530878
6.14	3.555	1.18356
6.15	2.96	1.382436
6.16	3.145	1.307017
6.17	2.685	0.794285
6.18	3.455	1.136349
6.19	3.095	0.977576
6.2	3.49	1.461338

6.21	3.11	1.281591
6.22	3.135	1.208577
6.23	3.14	0.987703
6.24	3.41	1.171146
6.25	3.035	1.362141
6.26	3.155	1.697677
6.27	3.365	1.205174
6.28	3.755	1.439316
6.29	2.945	1.251406
6.3	3.445	1.488665
6.31	3.015	1.616893
6.32	3.715	1.109639
6.33	3.465	1.252249
6.34	3.095	1.149134
6.35	3.645	1.461928
6.36	3.495	1.768421
6.37	3.285	1.285051
6.38	3.685	1.133344
6.39	3.5	1.190575
6.4	3.49	1.308652
6.41	3.74	1.288164
6.42	3.12	1.543427
6.43	4.09	1.09946
6.44	3.58	1.785257
6.45	3.525	1.758463
6.46	3.87	1.351779
6.47	3.485	1.09052
6.48	4.03	1.433461
6.49	3.91	1.543432
6.5	3.625	1.081588
6.51	3.995	1.344905
6.52	3.745	1.304126
6.53	3.825	1.546941
6.54	4.4	1.658735
6.55	2.925	1.526787
6.56	5.11	1.333191
6.57	3.15	1.964722
6.58	4.585	1.702385
6.59	3.825	1.895596
6.6	3.935	1.783291
6.61	3.88	1.542689
6.62	4.025	1.724298
6.63	4.365	1.427026
6.64	3.785	1.12586
6.65	4.745	1.304603
6.66	3.325	1.696299
6.67	4.415	1.653703
6.68	4.175	1.473208

6.69	3.925	1.595675
6.7	4.815	1.714492
6.71	3.99	1.586089
6.72	4.585	1.496836
6.73	4.18	1.79344
6.74	3.885	1.249926
6.75	4.38	1.825445
6.76	4.325	1.620525
6.77	4.815	1.529171
6.78	4.205	1.576789
6.79	4.485	1.626406
6.8	4.615	1.284409
6.81	3.84	1.727346
6.82	4.885	2.110688
6.83	3.99	1.756427
6.84	4.67	1.23666
6.85	4.995	2.248603
6.86	4.26	1.835297
6.87	4.685	1.872884
6.88	4.37	1.81807
6.89	4.575	1.921671
6.9	5.025	1.985717
6.91	4.37	1.936253
6.92	5.135	2.048889
6.93	4.755	1.863783
6.94	4.275	1.591104
6.95	4.93	1.799876
6.96	4.89	2.166029
6.97	4.66	1.851947
6.98	5.135	1.673703
6.99	4.8	2.168571
7	5.26	1.988116
7.01	3.945	1.863488
7.02	5.875	1.459518
7.03	4.42	1.892332
7.04	4.785	1.963293
7.05	5.57	2.11599
7.06	4.55	2.18622
7.07	4.99	2.209511
7.08	4.85	1.971904
7.09	5.385	2.058619
7.1	4.45	1.79075
7.11	5.265	1.975753
7.12	5.02	2.02286
7.13	4.905	2.129427
7.14	5.445	2.028813
7.15	5.105	2.062927
7.16	4.735	2.101794

7.17	5.235	1.952145
7.18	4.835	2.344101
7.19	5.995	2.071014
7.2	4.835	2.466056
7.21	5.615	2.079789
7.22	5.185	2.120913
7.23	4.745	2.031689
7.24	6.26	2.17352
7.25	4.425	1.844504
7.26	5.875	1.992238
7.27	5.805	1.952391
7.28	5.245	2.283781
7.29	5.22	2.177728
7.3	5.21	1.919619
7.31	5.67	2.820253
7.32	5.835	2.063573
7.33	5.21	2.013328
7.34	5.55	2.15945
7.35	5.855	2.413011
7.36	5.215	2.39054
7.37	5.715	2.087393
7.38	5.675	2.303714
7.39	5.315	2.028864
7.4	6.21	2.319822
7.41	5.24	2.335658
7.42	5.885	2.288516
7.43	6.11	2.786471
7.44	5.57	2.021506
7.45	4.865	1.94789
7.46	6.455	2.376809
7.47	5.36	2.683347
7.48	6.45	2.332301
7.49	5.415	2.285543
7.5	5.615	2.425627
7.51	6.05	2.32522
7.52	5.635	2.766936
7.53	5.96	2.413079
7.54	6.03	2.697736
7.55	5.5	2.118643
7.56	6.18	2.626507
7.57	6.32	3.021938
7.58	5.37	2.685395
7.59	6.26	2.382397
7.6	5.44	2.480902
7.61	6.305	2.623417
7.62	6.08	2.359254
7.63	6.06	2.419051
7.64	5.635	2.412771

7.65	6.24	2.545461
7.66	5.765	2.268202
7.67	5.835	2.429973
7.68	6.57	2.774815
7.69	5.55	2.49556
7.7	6.375	2.60153
7.71	6.47	2.818072
7.72	6.19	2.707453
7.73	5.76	2.484523
7.74	6.26	2.510165
7.75	5.915	2.456536
7.76	6.925	2.591403
7.77	5.94	2.573759
7.78	6.205	2.474395
7.79	6.4	2.58847
7.8	5.955	2.490223
7.81	5.965	2.694947
7.82	6.595	2.534823
7.83	6.75	2.817216
7.84	5.965	2.451902
7.85	5.95	2.548513
7.86	6.5	2.716914
7.87	6.53	2.866141
7.88	5.655	2.490436
7.89	6.68	2.696818
7.9	6.36	2.74832
7.91	6.595	2.223918
7.92	6.28	2.714383
7.93	6.945	2.674111
7.94	6.16	2.738268
7.95	6.005	3.035849
7.96	7.635	2.773592
7.97	6.16	3.077454
7.98	6.335	2.931064
7.99	7.985	2.698974
8	6.395	2.661399
8.01	6.17	2.697056
8.02	7.155	2.547763
8.03	6.45	2.61378
8.04	7.46	2.817042
8.05	6.81	2.703942
8.06	6.565	2.780141
8.07	7.45	2.650951
8.08	6.66	2.839879
8.09	5.93	2.712783
8.1	8.09	2.71372
8.11	6.265	2.651684
8.12	7.19	2.806566

8.13	7.04	2.656804
8.14	6.205	2.840682
8.15	6.75	3.264037
8.16	7.6	2.074978
8.17	6.29	3.256174
8.18	6.95	3.119981
8.19	7.34	2.884545
8.2	6.43	2.876786
8.21	7.73	3.315014
8.22	5.4	2.991378
8.23	7.265	2.997352
8.24	7.655	2.789597
8.25	6.45	2.738824
8.26	7.365	3.154518
8.27	6.59	3.197683
8.28	6.78	3.198411
8.29	7.64	2.872139
8.3	6.185	2.996665
8.31	7.795	3.43705
8.32	6.98	2.998707
8.33	7.12	2.865234
8.34	7.815	2.706922
8.35	7.265	2.599202
8.36	6.67	3.025971
8.37	7.415	3.39246
8.38	7.06	3.011226
8.39	7.87	3.14007
8.4	6.685	3.280889
8.41	8.235	3.221173
8.42	7.42	3.418117
8.43	7.05	2.919926
8.44	7	3.129672
8.45	7.95	3.19886
8.46	7.165	3.241979
8.47	6.81	3.449185
8.48	7.605	3.584746
8.49	8.935	3.360835
8.5	6.39	3.255476
8.51	7.04	3.026157
8.52	7.97	3.270654
8.53	7.41	3.285107
8.54	7.815	3.129684
8.55	7.935	2.840415
8.56	7.255	3.067614
8.57	7.35	3.523207
8.58	7.655	3.459899
8.59	7.675	3.632308
8.6	7.655	3.217451

8.61	7.11	3.332284
8.62	8.72	3.81599
8.63	7.265	3.751319
8.64	7.23	3.203911
8.65	7.46	3.311117
8.66	7.495	3.481922
8.67	7.59	3.12412
8.68	8.39	3.302428
8.69	7.565	3.439224
8.7	7.58	3.706375
8.71	7.655	3.30603
8.72	8.6	3.630641
8.73	6.445	3.408556
8.74	7.815	3.562454
8.75	8.795	3.478049
8.76	7.265	3.390208
8.77	7.475	3.275063
8.78	7.765	3.632164
8.79	8.135	3.882727
8.8	8.045	3.805664
8.81	6.6	3.429538
8.82	8.025	3.684359
8.83	8.785	3.530765
8.84	6.645	3.483424
8.85	8.35	3.379707
8.86	7.68	3.834943
8.87	6.82	3.52575
8.88	8.045	3.748277
8.89	8.845	3.219967
8.9	7.125	3.478571
8.91	7.76	3.266803
8.92	8.305	3.162
8.93	6.92	3.547206
8.94	7.955	3.737869
8.95	8.66	3.287404
8.96	6.785	3.911368
8.97	8.225	3.447248
8.98	8.855	3.566599
8.99	7.57	3.588719
9	7.455	3.812862
9.01	8.475	3.764138
9.02	7.71	3.191464
9.03	7.59	3.694127
9.04	8.34	3.53855
9.05	8.37	3.825017
9.06	8.12	3.568195
9.07	7.275	3.785427
9.08	7.825	3.534632

9.09	8.885	3.63849
9.1	8.615	3.697199
9.11	7.355	3.389303
9.12	8.29	3.74325
9.13	8.96	3.922857
9.14	8.385	3.465955
9.15	7.585	3.866301
9.16	8.81	3.774593
9.17	8.72	3.865699
9.18	8.485	3.798022
9.19	7.84	3.374186
9.2	7.75	4.052228
9.21	9.755	3.921917
9.22	7.89	3.523926
9.23	6.705	3.635034
9.24	9.505	3.627906
9.25	8.085	4.163722
9.26	8.665	4.143316
9.27	7.875	3.961744
9.28	8.18	3.909531
9.29	8.66	3.726514
9.3	8	4.259034
9.31	8.11	3.949763
9.32	8.92	3.771899
9.33	8.59	3.941953
9.34	8.345	3.981493
9.35	8.285	3.961427
9.36	8.155	4.103775
9.37	8.895	3.613006
9.38	7.8	3.641553
9.39	8.65	3.634536
9.4	8.285	4.213706
9.41	9.03	3.935955
9.42	8.52	3.759005
9.43	7.71	3.779793
9.44	9.54	4.104225
9.45	7.625	3.923729
9.46	7.595	3.525035
9.47	10.14	3.499299
9.48	7.77	3.984251
9.49	8.865	3.931248
9.5	7.385	3.843985
9.51	8.66	4.230791
9.52	10.125	4.161025
9.53	6.76	4.055112
9.54	8.075	3.968838
9.55	9.66	3.998011
9.56	9.245	3.71505

9.57	7.225	4.097763
9.58	8.45	4.140475
9.59	9.55	4.072775
9.6	7.965	3.81933
9.61	9.03	4.133698
9.62	8.45	4.147363
9.63	8.865	3.901071
9.64	8.765	3.791253
9.65	8.515	3.844008
9.66	8.945	3.966316
9.67	8.03	3.666855
9.68	8.925	3.89441
9.69	9.26	4.346599
9.7	8.8	3.556196
9.71	8.58	3.984982
9.72	8.165	3.748616
9.73	9.2	4.006314

Table S2. 8-DOF Data under Steady-State Cornering Condition

Time	ay	rollangle
0	0.184682	0.238523
0.025	0.3114	0.229984
0.05	0.351681	0.211654
0.075	0.342897	0.19412
0.1	0.302811	0.183472
0.125	0.253638	0.179568
0.15	0.207088	0.178335
0.175	0.168894	0.175246
0.2	0.138763	0.167802
0.225	0.112792	0.155866
0.25	0.0861	0.141555
0.275	0.056779	0.127508
0.3	0.027954	0.115399
0.325	0.003901	0.105548
0.35	-0.01462	0.097295
0.375	-0.0272	0.089551
0.4	-0.03335	0.081133
0.425	-0.03429	0.071382
0.45	-0.03266	0.060736
0.475	-0.03199	0.050575
0.5	-0.03379	0.042244
0.525	-0.03585	0.036582
0.55	-0.03757	0.033449

0.575	-0.03743	0.0322
0.6	-0.0349	0.03194
0.625	-0.03134	0.032154
0.65	-0.02639	0.032719
0.675	-0.02344	0.033871
0.7	-0.02126	0.035357
0.725	-0.01858	0.036785
0.75	-0.0156	0.037609
0.775	-0.01278	0.037546
0.8	-0.01102	0.036591
0.825	-0.01028	0.035003
0.85	-0.01036	0.033181
0.875	-0.01067	0.031598
0.9	-0.01308	0.030824
0.925	-0.01651	0.031103
0.95	-0.02049	0.032168
0.975	-0.02221	0.033474
1	-0.02323	0.034309
1.025	-0.02318	0.034327
1.05	-0.02296	0.033606
1.075	-0.02353	0.032656
1.1	-0.0251	0.031987
1.125	-0.02746	0.031955
1.15	-0.02969	0.032341
1.175	-0.03077	0.032815
1.2	-0.03058	0.032918
1.225	-0.03133	0.032458
1.25	-0.03162	0.031532
1.275	-0.03169	0.030508
1.3	-0.03211	0.029853
1.325	-0.03403	0.029855
1.35	-0.03639	0.030375
1.375	-0.03772	0.031145
1.4	-0.03896	0.031711
1.425	-0.03826	0.031915
1.45	-0.03782	0.031724
1.475	-0.03778	0.031345
1.5	-0.03901	0.030966
1.525	-0.03974	0.030849
1.55	-0.04083	0.03108
1.575	-0.04198	0.031689
1.6	-0.04299	0.032455
1.625	-0.04361	0.033197
1.65	-0.0436	0.033632
1.675	-0.04305	0.033769
1.7	-0.04262	0.033635
1.725	-0.04237	0.033302
1.75	-0.04216	0.032564

1.775	-0.04133	0.031359
1.8	-0.04091	0.0296
1.825	-0.04076	0.02767
1.85	-0.04082	0.025931
1.875	-0.04268	0.024859
1.9	-0.04417	0.024605
1.925	-0.04539	0.025123
1.95	-0.04573	0.025841
1.975	-0.04473	0.026109
2	-0.04334	0.025318
2.025	-0.04068	0.023457
2.05	-0.03867	0.020978
2.075	-0.03704	0.018626
2.1	-0.03716	0.01685
2.125	-0.0374	0.015789
2.15	-0.03849	0.015081
2.175	-0.0378	0.014231
2.2	-0.03645	0.012823
2.225	-0.03439	0.01094
2.25	-0.03324	0.008843
2.275	-0.03303	0.00709
2.3	-0.0329	0.005891
2.325	-0.03296	0.005305
2.35	-0.03224	0.004943
2.375	-0.03081	0.004512
2.4	-0.02866	0.003652
2.425	-0.02616	0.002456
2.45	-0.0249	0.001116
2.475	-0.02468	6.00E-06
2.5	-0.02503	-0.00078
2.525	-0.02495	-0.00121
2.55	-0.02393	-0.00158
2.575	-0.02269	-0.00208
2.6	-0.0209	-0.00301
2.625	-0.01876	-0.00429
2.65	-0.01718	-0.00574
2.675	-0.01624	-0.00697
2.7	-0.01645	-0.00783
2.725	-0.01663	-0.00834
2.75	-0.01605	-0.00888
2.775	-0.01548	-0.00968
2.8	-0.01372	-0.011
2.825	-0.01182	-0.0126
2.85	-0.0102	-0.01422
2.875	-0.00913	-0.01547
2.9	-0.00927	-0.01629
2.925	-0.00926	-0.01672
2.95	-0.0096	-0.01705

2.975	-0.00791	-0.0174
3	-0.00619	-0.01789
3.025	-0.00477	-0.01848
3.05	-0.00385	-0.01918
3.075	-0.00364	-0.01981
3.1	-0.00368	-0.02044
3.125	-0.00272	-0.02092
3.15	-0.00177	-0.02136
3.175	-0.00097	-0.02164
3.2	-0.00033	-0.02182
3.225	-0.00022	-0.02179
3.25	0.000299	-0.02163
3.275	0.000493	-0.02132
3.3	0.000665	-0.02098
3.325	0.001	-0.02057
3.35	0.001651	-0.02022
3.375	0.002565	-0.01987
3.4	0.003704	-0.01956
3.425	0.003937	-0.01922
3.45	0.003814	-0.01897
3.475	0.003956	-0.01875
3.5	0.004214	-0.01865
3.525	0.00403	-0.0186
3.55	0.003831	-0.01867
3.575	0.004817	-0.01876
3.6	0.004571	-0.0189
3.625	0.00439	-0.01901
3.65	0.005213	-0.01914
3.675	0.005733	-0.01919
3.7	0.005498	-0.01919
3.725	0.005181	-0.0191
3.75	0.004533	-0.01905
3.775	0.004399	-0.01898
3.8	0.004458	-0.01899
3.825	0.004633	-0.019
3.85	0.004996	-0.01906
3.875	0.005324	-0.01909
3.9	0.005713	-0.01912
3.925	0.006516	-0.01906
3.95	0.006112	-0.01898
3.975	0.005394	-0.01885
4	0.003955	-0.0188
4.025	0.004133	-0.01882
4.05	0.004704	-0.01896
4.075	0.00463	-0.0191
4.1	0.004757	-0.01928
4.125	0.004477	-0.01943
4.15	0.004249	-0.01959

4.175	0.003874	-0.01972
4.2	0.003694	-0.01986
4.225	0.003504	-0.01997
4.25	0.003459	-0.0201
4.275	0.003328	-0.02017
4.3	0.003167	-0.02027
4.325	0.003056	-0.02032
4.35	0.002872	-0.02039
4.375	0.00293	-0.02042
4.4	0.002959	-0.02047
4.425	0.002961	-0.02046
4.45	0.003798	-0.02047
4.475	0.015369	-0.02022
4.5	0.032829	-0.01916
4.525	0.051773	-0.0165
4.55	0.071298	-0.01187
4.575	0.092802	-0.00527
4.6	0.117148	0.002585
4.625	0.146363	0.010713
4.65	0.181082	0.018327
4.675	0.218799	0.025271
4.7	0.258734	0.031939
4.725	0.300367	0.039037
4.75	0.343154	0.047069
4.775	0.387603	0.05621
4.8	0.434071	0.066094
4.825	0.483164	0.07655
4.85	0.532628	0.087725
4.875	0.582365	0.099677
4.9	0.632064	0.112354
4.925	0.68179	0.125559
4.95	0.731393	0.138765
4.975	0.781501	0.151613
5	0.830149	0.164505
5.025	0.876233	0.178156
5.05	0.921228	0.193494
5.075	0.966218	0.210941
5.1	1.010866	0.230457
5.125	1.055832	0.251852
5.15	1.101249	0.27478
5.175	1.147613	0.298773
5.2	1.194106	0.323304
5.225	1.24133	0.347864
5.25	1.289214	0.372029
5.275	1.33697	0.395543
5.3	1.384709	0.418298
5.325	1.43262	0.44031
5.35	1.479766	0.461667

5.375	1.526726	0.482495
5.4	1.573433	0.502961
5.425	1.619552	0.523235
5.45	1.665965	0.543473
5.475	1.712544	0.563812
5.5	1.758496	0.584366
5.525	1.804997	0.605192
5.55	1.85193	0.626318
5.575	1.898774	0.647757
5.6	1.946068	0.669486
5.625	1.993785	0.69147
5.65	2.041726	0.713661
5.675	2.089756	0.735998
5.7	2.138168	0.758428
5.725	2.186933	0.780903
5.75	2.235502	0.803384
5.775	2.283531	0.825807
5.8	2.331963	0.848084
5.825	2.381326	0.870159
5.85	2.429885	0.892022
5.875	2.478796	0.913696
5.9	2.527728	0.935228
5.925	2.576267	0.956673
5.95	2.624611	0.978068
5.975	2.673219	0.999456
6	2.721768	1.020876
6.025	2.770013	1.042349
6.05	2.818523	1.06389
6.075	2.867219	1.085512
6.1	2.916347	1.107239
6.125	2.965092	1.129077
6.15	3.013769	1.151019
6.175	3.062161	1.173036
6.2	3.110955	1.195086
6.225	3.160308	1.21715
6.25	3.2092	1.239231
6.275	3.258132	1.26132
6.3	3.307435	1.2834
6.325	3.356269	1.30549
6.35	3.404916	1.32758
6.375	3.453661	1.34965
6.4	3.502602	1.37168
6.425	3.551377	1.39365
6.45	3.600583	1.41553
6.475	3.649563	1.43735
6.5	3.698789	1.45915
6.525	3.747446	1.48097
6.55	3.795622	1.5028

6.575	3.844319	1.52466
6.6	3.893025	1.54656
6.625	3.941437	1.56849
6.65	3.990162	1.59048
6.675	4.038555	1.61253
6.7	4.087035	1.63461
6.725	4.135369	1.65673
6.75	4.183536	1.67888
6.775	4.232016	1.70103
6.8	4.280095	1.7232
6.825	4.328399	1.74536
6.85	4.376292	1.76753
6.875	4.424145	1.78968
6.9	4.471568	1.8118
6.925	4.519686	1.83388
6.95	4.56796	1.85596
6.975	4.615373	1.87805
7	4.662668	1.90017
7.025	4.709365	1.9223
7.05	4.756503	1.94445
7.075	4.80319	1.96663
7.1	4.849348	1.98886
7.125	4.89581	2.01113
7.15	4.942154	2.03341
7.175	4.9874	2.05569
7.2	5.033206	2.07793
7.225	5.078942	2.10011
7.25	5.124404	2.12221
7.275	5.169543	2.14423
7.3	5.21382	2.16613
7.325	5.258351	2.1879
7.35	5.302911	2.20954
7.375	5.347149	2.23106
7.4	5.391298	2.25249
7.425	5.435162	2.27386
7.45	5.478576	2.29516
7.475	5.521745	2.31642
7.5	5.564973	2.33762
7.525	5.608132	2.35878
7.55	5.651311	2.37991
7.575	5.693882	2.40092
7.6	5.736405	2.42172
7.625	5.779309	2.44219
7.65	5.821861	2.46232
7.675	5.86453	2.48218
7.7	5.906934	2.50185
7.725	5.949241	2.52148
7.75	5.990783	2.54113

7.775	6.031404	2.56087
7.8	6.071065	2.58067
7.825	6.111362	2.6005
7.85	6.150896	2.62033
7.875	6.190203	2.64012
7.9	6.229668	2.65984
7.925	6.268829	2.67947
7.95	6.307402	2.69899
7.975	6.346161	2.71839
8	6.384831	2.73768
8.025	6.423365	2.75685
8.05	6.461575	2.77596
8.075	6.498217	2.79498
8.1	6.535154	2.81386
8.125	6.572766	2.83259
8.15	6.609967	2.85121
8.175	6.647491	2.86975
8.2	6.684888	2.88825
8.225	6.720589	2.90673
8.25	6.757114	2.92517
8.275	6.793403	2.94356
8.3	6.829203	2.96189
8.325	6.865747	2.98019
8.35	6.901301	2.99847
8.375	6.936287	3.01667
8.4	6.971832	3.03475
8.425	7.006063	3.05266
8.45	7.041069	3.07038
8.475	7.076535	3.08794
8.5	7.111178	3.10541
8.525	7.146164	3.12287
8.55	7.180807	3.14037
8.575	7.214186	3.15793
8.6	7.24731	3.17551
8.625	7.281041	3.19307
8.65	7.314714	3.21058
8.675	7.348642	3.22803
8.7	7.382275	3.24545
8.725	7.415164	3.26283
8.75	7.448033	3.28015
8.775	7.480285	3.2974
8.8	7.512606	3.31459
8.825	7.545485	3.33172
8.85	7.577531	3.34879
8.875	7.609537	3.36582
8.9	7.641397	3.3828
8.925	7.672865	3.39972
8.95	7.704343	3.41658

8.975	7.735771	3.4334
9	7.766279	3.45013
9.025	7.796884	3.46676
9.05	7.827519	3.48329
9.075	7.857536	3.4997
9.1	7.887328	3.516
9.125	7.916875	3.53219
9.15	7.945746	3.54827
9.175	7.974979	3.56423
9.2	8.003282	3.58008
9.225	8.030604	3.59577
9.25	8.057897	3.61127
9.275	8.084867	3.62657
9.3	8.112111	3.64166
9.325	8.139423	3.6566
9.35	8.165717	3.67141
9.375	8.190873	3.6861
9.4	8.216373	3.70063
9.425	8.241226	3.71502
9.45	8.265941	3.72923
9.475	8.290089	3.74326
9.5	8.313961	3.75706
9.525	8.336835	3.77063
9.55	8.359473	3.78395
9.575	8.382395	3.797
9.6	8.403955	3.80981
9.625	8.425446	3.82237
9.65	8.445869	3.83466
9.675	8.466244	3.84667
9.7	8.485942	3.85841
9.725	8.504532	3.86985

Table S3. Test Data under Double Lane Change Condition

Time	ay	yawrate
0.01	-0.275	-0.325
0.02	-0.22	-0.34
0.03	-0.085	-0.23
0.04	0.155	-0.185
0.05	0.01	-0.13
0.06	-0.73	-0.34
0.07	-0.005	-0.215
0.08	-0.035	-0.175

0.09	-0.235	-0.405
0.1	-0.015	-0.255
0.11	-0.06	-0.315
0.12	-0.31	-0.36
0.13	0.015	-0.27
0.14	0.01	-0.275
0.15	-0.43	-0.305
0.16	-0.32	-0.575
0.17	0.84	0.305
0.18	0.84	0.305
0.19	-0.71	-0.735
0.2	-0.71	-0.735
0.21	-0.185	-0.085
0.22	-0.655	-0.52
0.23	0.54	-0.025
0.24	-0.16	-0.12
0.25	-0.695	-0.54
0.26	0.585	0.215
0.27	-0.24	-0.23
0.28	-0.24	-0.23
0.29	0.11	-0.465
0.3	0.27	-0.195
0.31	-0.14	-0.175
0.32	0.105	-0.08
0.33	-0.455	-0.505
0.34	-0.3	-0.4
0.35	0.155	-0.225
0.36	0.16	-0.25
0.37	-0.55	-0.315
0.38	0.135	-0.375
0.39	-0.045	-0.155
0.4	-0.22	-0.28
0.41	-0.115	-0.415
0.42	-0.085	-0.23
0.43	-0.35	-0.295
0.44	0.27	-0.17
0.45	0.27	-0.17
0.46	0.335	-0.09
0.47	-0.15	-0.17
0.48	-0.845	-0.46
0.49	0.29	-0.19
0.5	-0.16	-0.11
0.51	-0.19	-0.35
0.52	-0.355	-0.36
0.53	0.055	-0.17
0.54	-0.33	-0.56
0.55	-0.075	-0.115
0.56	0.075	-0.04

0.57	-0.36	-0.41
0.58	-0.415	-0.54
0.59	0.49	-0.03
0.6	-0.635	-0.335
0.61	-0.635	-0.335
0.62	0.16	-0.125
0.63	-0.43	-0.385
0.64	-0.17	-0.215
0.65	-0.155	-0.255
0.66	0.15	-0.3
0.67	-0.635	-0.465
0.68	0.335	-0.075
0.69	-0.445	-0.33
0.7	0.085	0.075
0.71	0.185	-0.065
0.72	-1.255	-0.64
0.73	0.68	-0.11
0.74	-0.145	-0.25
0.75	-0.435	-0.29
0.76	0.33	-0.045
0.77	-0.265	-0.265
0.78	-0.18	-0.115
0.79	-0.645	-0.46
0.8	0.435	-0.225
0.81	-0.165	-0.35
0.82	-0.095	-0.165
0.83	-0.1	-0.25
0.84	-0.425	-0.335
0.85	0.47	0.04
0.86	-0.84	-0.655
0.87	0.145	-0.35
0.88	0.03	-0.295
0.89	-0.15	-0.335
0.9	0.065	-0.32
0.91	-0.53	-0.455
0.92	0.37	-0.015
0.93	-0.77	-0.735
0.94	0.25	-0.255
0.95	-0.065	-0.395
0.96	0.11	-0.32
0.97	-0.52	-0.58
0.98	-0.1	-0.29
0.99	0.04	-0.37
1	-0.28	-0.36
1.01	-0.11	-0.24
1.02	-0.15	-0.39
1.03	-0.195	-0.43
1.04	-0.355	-0.575

1.05	0.61	-0.06
1.06	-1.065	-0.47
1.07	0.425	-0.2
1.08	-0.095	-0.29
1.09	-0.56	-0.5
1.1	-0.07	-0.4
1.11	0.33	-0.195
1.12	-0.58	-0.175
1.13	0.005	-0.22
1.14	-0.385	-0.375
1.15	0.2	-0.065
1.16	-0.405	-0.375
1.17	0.005	-0.17
1.18	-0.05	-0.135
1.19	-0.325	-0.185
1.2	-0.06	-0.215
1.21	-0.53	-0.14
1.22	-0.13	-0.385
1.23	0.415	-0.11
1.24	-0.61	-0.375
1.25	0.36	0.085
1.26	-0.485	-0.245
1.27	-0.045	-0.15
1.28	-0.23	-0.32
1.29	-0.23	-0.345
1.3	0.1	-0.265
1.31	-0.045	-0.32
1.32	-0.08	-0.2
1.33	0.2	0.09
1.34	-0.75	-0.56
1.35	-0.065	-0.2
1.36	0.225	-0.08
1.37	-0.31	-0.395
1.38	-0.38	-0.495
1.39	0.265	-0.205
1.4	-0.005	-0.045
1.41	-0.605	-0.375
1.42	-0.13	-0.36
1.43	0.35	-0.045
1.44	-0.835	-0.68
1.45	0.225	-0.2
1.46	0.155	-0.205
1.47	-0.025	-0.015
1.48	-0.875	-0.47
1.49	-0.875	-0.47
1.5	-0.655	-0.65
1.51	0.36	-0.07
1.52	0.265	-0.085

1.53	-1.46	-0.84
1.54	1.095	-0.015
1.55	-0.425	-0.07
1.56	-0.48	-0.4
1.57	0.225	-0.335
1.58	-0.565	-0.565
1.59	1.235	0.135
1.6	0.1	0.1
1.61	1.05	0.855
1.62	0.765	1.21
1.63	2.03	2.405
1.64	1	2.845
1.65	0.82	3.6
1.66	2.095	4.285
1.67	0.37	4.36
1.68	0.25	4.65
1.69	1.625	5.295
1.7	1.035	5.26
1.71	0.275	5.06
1.72	1.12	5.365
1.73	1.12	5.365
1.74	0.88	5.215
1.75	2.2	5.555
1.76	0.74	4.975
1.77	2.52	5.9
1.78	0.83	5.18
1.79	1.71	5.165
1.8	2.315	5.48
1.81	1.08	4.66
1.82	2.695	5.47
1.83	1.51	5.075
1.84	1.51	5.075
1.85	1.745	5.015
1.86	2.22	4.83
1.87	1.405	4.48
1.88	2.6	4.92
1.89	1.895	4.615
1.9	1.855	4.37
1.91	2.185	4.4
1.92	2.335	4.615
1.93	1.405	4.015
1.94	2.48	4.085
1.95	2.24	4.24
1.96	2.04	4.14
1.97	2.12	3.97
1.98	1.925	3.92
1.99	2.375	4.125
2	2.3	4.055

2.01	1.97	3.895
2.02	1.915	3.625
2.03	2.24	3.785
2.04	2.545	4.18
2.05	1.21	3.535
2.06	2.5	3.86
2.07	2.155	3.81
2.08	1.67	3.49
2.09	2.765	4.055
2.1	1.325	3.5
2.11	2.285	3.63
2.12	2.25	4.03
2.13	1.69	3.69
2.14	2.395	3.98
2.15	1.565	3.585
2.16	2.015	3.745
2.17	2.615	4.13
2.18	1.26	3.69
2.19	2.435	3.845
2.2	1.715	3.795
2.21	2.015	3.78
2.22	1.955	3.82
2.23	2.45	4.175
2.24	1.385	3.73
2.25	1.74	3.515
2.26	1.74	3.515
2.27	1.47	3.965
2.28	2.02	4.12
2.29	2.065	4.005
2.3	1.78	3.945
2.31	1.78	3.945
2.32	2.13	3.99
2.33	2.055	4.145
2.34	2.165	4.19
2.35	1.595	3.935
2.36	2.045	3.805
2.37	2.03	4.065
2.38	2.25	4.175
2.39	1.86	4.02
2.4	1.585	3.75
2.41	2.52	4.29
2.42	1.7	3.995
2.43	1.525	3.84
2.44	2.72	4.13
2.45	1.61	4.055
2.46	1.78	3.95
2.47	2.185	4
2.48	2.105	4.15

2.49	1.74	4.24
2.5	2.12	3.95
2.51	1.365	3.655
2.52	2.49	4.22
2.53	2.095	4.01
2.54	1.765	4.095
2.55	1.79	4.075
2.56	1.9	3.875
2.57	1.7	3.83
2.58	2.025	3.89
2.59	2.305	4.07
2.6	1.41	3.75
2.61	2.26	4.09
2.62	2.015	4.015
2.63	1.865	4.195
2.64	1.705	3.9
2.65	2.24	4.09
2.66	1.74	3.87
2.67	1.925	3.985
2.68	2.105	4.165
2.69	1.54	3.89
2.7	2.165	4.185
2.71	2.03	4.12
2.72	1.6	3.845
2.73	2.34	4.055
2.74	1.99	4.195
2.75	1.965	4.095
2.76	1.41	3.735
2.77	2.535	4.2
2.78	1.42	3.855
2.79	2.205	4.1
2.8	2.165	3.965
2.81	1.79	3.94
2.82	1.965	4.135
2.83	2.145	4
2.84	1.7	4.03
2.85	2.345	4.335
2.86	1.24	3.79
2.87	2.375	4.085
2.88	2.07	3.81
2.89	2.005	4.045
2.9	1.805	4.105
2.91	2.175	4.19
2.92	2.135	4.055
2.93	1.44	3.855
2.94	2.145	3.975
2.95	2.05	4.005
2.96	1.69	3.85

2.97	2.32	4.095
2.98	1.89	4.06
2.99	1.895	4.045
3	1.9	3.965
3.01	1.825	3.79
3.02	1.825	3.79
3.03	2.49	4.375
3.04	1.245	3.74
3.05	2.255	4.005
3.06	1.87	3.98
3.07	1.985	4.125
3.08	2.04	3.88
3.09	2.04	3.945
3.1	1.85	3.825
3.11	1.705	3.785
3.12	2.23	4
3.13	1.615	3.755
3.14	2.14	3.75
3.15	1.685	3.775
3.16	1.97	3.65
3.17	2.325	4.01
3.18	1.61	3.795
3.19	1.57	3.675
3.2	2.395	3.98
3.21	1.635	3.75
3.22	1.945	3.765
3.23	1.73	3.635
3.24	2.36	4.045
3.25	1.21	3.685
3.26	2.01	3.85
3.27	2.015	3.82
3.28	2.015	3.82
3.29	2.03	3.775
3.3	1.655	3.695
3.31	2.135	3.905
3.32	1.65	3.965
3.33	2.115	3.91
3.34	1.5	3.785
3.35	1.665	3.695
3.36	2.36	3.865
3.37	1.51	3.8
3.38	1.82	3.805
3.39	2.42	4.1
3.4	0.71	3.415
3.41	2.815	4.275
3.42	1.73	3.835
3.43	1.615	3.835
3.44	1.77	3.655

3.45	2.5	4.04
3.46	2.5	4.04
3.47	2.55	4.265
3.48	1.63	3.795
3.49	2.19	4.14
3.5	1.185	3.77
3.51	2.49	3.88
3.52	1.37	3.705
3.53	2.035	3.88
3.54	2.355	4.175
3.55	1.23	3.685
3.56	2.23	4.105
3.57	1.91	3.9
3.58	1.805	3.99
3.59	1.405	3.565
3.6	3.1	4.425
3.61	1.125	3.84
3.62	1.695	3.93
3.63	2.375	4.085
3.64	2.375	4.085
3.65	2.125	3.945
3.66	1.54	3.745
3.67	2.36	4.095
3.68	1.255	3.635
3.69	2.46	4.12
3.7	1.745	3.84
3.71	1.75	3.8
3.72	2.115	4.01
3.73	2.03	3.9
3.74	1.87	4.005
3.75	1.4	3.575
3.76	2.67	4.355
3.77	1.485	3.89
3.78	1.79	3.785
3.79	1.995	3.88
3.8	2.125	3.81
3.81	1.63	3.82
3.82	1.795	3.705
3.83	2.295	4.005
3.84	1.655	3.81
3.85	1.855	3.86
3.86	2.42	4.07
3.87	1.52	3.745
3.88	1.745	3.73
3.89	1.985	3.79
3.9	2.195	4.055
3.91	1.69	3.78
3.92	1.6	3.77

3.93	2.34	4.01
3.94	1.815	3.755
3.95	1.77	3.95
3.96	1.87	3.74
3.97	2.17	3.97
3.98	1.535	3.845
3.99	1.775	3.695
4	2.5	3.98
4.01	1.38	3.79
4.02	2.07	3.75
4.03	2.07	3.75
4.04	1.51	3.735
4.05	2.49	4.135
4.06	1.315	3.745
4.07	1.905	3.765
4.08	2.33	3.805
4.09	1.49	3.845
4.1	1.565	3.52
4.11	2.97	4.255
4.12	0.785	3.54
4.13	2.73	4.155
4.14	1.68	3.77
4.15	1.765	3.75
4.16	2.085	3.92
4.17	1.715	3.63
4.18	1.9	3.97
4.19	1.635	3.725
4.2	2.49	4.09
4.21	1.445	3.61
4.22	1.865	3.71
4.23	2.405	4.15
4.24	1.6	3.81
4.25	1.62	3.76
4.26	1.91	3.82
4.27	1.795	3.69
4.28	1.795	3.69
4.29	1.6	3.855
4.3	1.595	3.68
4.31	2.31	3.935
4.32	1.48	3.875
4.33	2.265	4.02
4.34	1.85	4.025
4.35	1.28	3.58
4.36	2.275	3.975
4.37	2.015	3.88
4.38	1.53	3.74
4.39	2.195	4.005
4.4	1.72	3.865

4.41	2.1	4.04
4.42	1.505	3.785
4.43	2.285	3.89
4.44	2.285	3.89
4.45	2.125	3.925
4.46	1.53	3.79
4.47	2.28	4.015
4.48	1.6	3.77
4.49	1.945	3.93
4.5	1.985	3.885
4.51	1.795	3.755
4.52	1.965	4.055
4.53	1.46	3.59
4.54	2.5	4.13
4.55	1.53	3.65
4.56	2.22	4.13
4.57	1.475	3.61
4.58	2.455	4.15
4.59	1.21	3.685
4.6	2.81	4.42
4.61	0.92	3.635
4.62	2.155	3.92
4.63	1.77	3.735
4.64	2.15	4.03
4.65	1.645	3.63
4.66	1.885	3.81
4.67	2.325	4.235
4.68	1.41	3.78
4.69	2.155	4.045
4.7	1.52	3.875
4.71	2.035	3.81
4.72	2.1	3.865
4.73	1.405	3.695
4.74	2.565	4.23
4.75	1.05	3.65
4.76	2.69	4.095
4.77	1.535	3.955
4.78	1.765	3.66
4.79	1.88	3.675
4.8	2.505	4.065
4.81	1.435	3.735
4.82	1.93	3.88
4.83	2.45	4.115
4.84	1.35	3.67
4.85	2.17	3.985
4.86	1.85	3.67
4.87	1.71	3.58
4.88	1.83	3.715

4.89	2.345	3.985
4.9	1.825	4.005
4.91	1.65	3.885
4.92	1.81	3.855
4.93	2.3	3.845
4.94	1.315	3.755
4.95	2.02	3.915
4.96	2.02	3.915
4.97	1.935	3.87
4.98	1.825	3.945
4.99	1.835	3.965
5	2.295	3.985

Table S4. 8-DOF Data under Double Lane Change Condition

Time	ay	yawrate
0	-0.41532	0
0.025	-0.16775	-0.23527
0.05	-0.10419	-0.32185
0.075	-0.10665	-0.35446
0.1	-0.13225	-0.36181
0.125	-0.15769	-0.35518
0.15	-0.17438	-0.33971
0.175	-0.18222	-0.31937
0.2	-0.18539	-0.29833
0.225	-0.19008	-0.28078
0.25	-0.19653	-0.26485
0.275	-0.20323	-0.25155
0.3	-0.21034	-0.23828
0.325	-0.21492	-0.22798
0.35	-0.21683	-0.21889
0.375	-0.21611	-0.21171
0.4	-0.21433	-0.20691
0.425	-0.21295	-0.20676
0.45	-0.21315	-0.21055
0.475	-0.21387	-0.21576
0.5	-0.21442	-0.22104
0.525	-0.21456	-0.22544
0.55	-0.21351	-0.23098
0.575	-0.21377	-0.23655
0.6	-0.2137	-0.24268
0.625	-0.21437	-0.25058
0.65	-0.21454	-0.25804

0.675	-0.21737	-0.26492
0.7	-0.22005	-0.27001
0.725	-0.22291	-0.27592
0.75	-0.22593	-0.28049
0.775	-0.22805	-0.28632
0.8	-0.22925	-0.29069
0.825	-0.2299	-0.29457
0.85	-0.2299	-0.29628
0.875	-0.23179	-0.29909
0.9	-0.23422	-0.30165
0.925	-0.23656	-0.30342
0.95	-0.2402	-0.30485
0.975	-0.24318	-0.30625
1	-0.24433	-0.30671
1.025	-0.24442	-0.30778
1.05	-0.24484	-0.30864
1.075	-0.24568	-0.30996
1.1	-0.2468	-0.31349
1.125	-0.24815	-0.31672
1.15	-0.24951	-0.3201
1.175	-0.25142	-0.32222
1.2	-0.25231	-0.32453
1.225	-0.25291	-0.32525
1.25	-0.25285	-0.32723
1.275	-0.2525	-0.32808
1.3	-0.25366	-0.33011
1.325	-0.25528	-0.33176
1.35	-0.25653	-0.33344
1.375	-0.25817	-0.33575
1.4	-0.26011	-0.33748
1.425	-0.26052	-0.33992
1.45	-0.26079	-0.34177
1.475	-0.26063	-0.34367
1.5	-0.26002	-0.34386
1.525	-0.25628	-0.34315
1.55	-0.02084	-0.23644
1.575	0.440313	0.427007
1.6	0.59694	1.50073
1.625	0.631055	2.58099
1.65	0.664082	3.45539
1.675	0.75177	4.08812
1.7	0.905509	4.52532
1.725	1.088132	4.82173
1.75	1.249548	5.01065
1.775	1.386337	5.10028
1.8	1.509572	5.10458
1.825	1.616921	5.04796
1.85	1.708218	4.94991

1.875	1.785794	4.82677
1.9	1.847789	4.69272
1.925	1.896044	4.55533
1.95	1.934225	4.42149
1.975	1.962714	4.29902
2	1.981295	4.1906
2.025	1.990291	4.09896
2.05	1.990379	4.02411
2.075	1.983411	3.96844
2.1	1.969446	3.92449
2.125	1.948964	3.88628
2.15	1.922798	3.8546
2.175	1.895368	3.83421
2.2	1.870613	3.82643
2.225	1.852591	3.83
2.25	1.839773	3.84191
2.275	1.830551	3.85632
2.3	1.822358	3.87018
2.325	1.814822	3.87969
2.35	1.806502	3.88603
2.375	1.799573	3.88982
2.4	1.795408	3.89561
2.425	1.795134	3.90186
2.45	1.79777	3.90765
2.475	1.799799	3.91329
2.5	1.799632	3.91722
2.525	1.796947	3.92038
2.55	1.794418	3.92113
2.575	1.792782	3.92194
2.6	1.792272	3.92449
2.625	1.793282	3.92834
2.65	1.794193	3.93014
2.675	1.796418	3.93234
2.7	1.799446	3.93098
2.725	1.802219	3.93122
2.75	1.803297	3.93132
2.775	1.803082	3.93343
2.8	1.801906	3.93354
2.825	1.799955	3.9337
2.85	1.798877	3.93252
2.875	1.798574	3.93273
2.9	1.799759	3.93286
2.925	1.801161	3.93583
2.95	1.803434	3.93715
2.975	1.805483	3.93903
3	1.806619	3.93762
3.025	1.805698	3.93685
3.05	1.803915	3.93424

3.075	1.803248	3.93206
3.1	1.803258	3.92993
3.125	1.804601	3.93033
3.15	1.806041	3.93198
3.175	1.806649	3.93446
3.2	1.807119	3.93528
3.225	1.806933	3.9355
3.25	1.806031	3.93353
3.275	1.80559	3.93111
3.3	1.805728	3.92955
3.325	1.806688	3.92961
3.35	1.806502	3.93105
3.375	1.80611	3.93245
3.4	1.80608	3.93315
3.425	1.806463	3.93433
3.45	1.80608	3.93516
3.475	1.806453	3.93537
3.5	1.806855	3.93549
3.525	1.807472	3.93589
3.55	1.807952	3.93683
3.575	1.808099	3.93777
3.6	1.808442	3.93847
3.625	1.808481	3.93935
3.65	1.80804	3.93924
3.675	1.808864	3.93917
3.7	1.809305	3.93915
3.725	1.809412	3.93934
3.75	1.809344	3.93923
3.775	1.808805	3.93881
3.8	1.808785	3.93745
3.825	1.809069	3.93626
3.85	1.80954	3.9354
3.875	1.809726	3.93567
3.9	1.809824	3.9364
3.925	1.809951	3.93714
3.95	1.810392	3.93854
3.975	1.810392	3.94037
4	1.810255	3.9416
4.025	1.810147	3.94238
4.05	1.81001	3.9425
4.075	1.809393	3.94178
4.1	1.809422	3.94029
4.125	1.809785	3.93896
4.15	1.810069	3.93791
4.175	1.810334	3.93769
4.2	1.810343	3.9379
4.225	1.810255	3.93838
4.25	1.810461	3.93903

4.275	1.81099	3.94062
4.3	1.81102	3.9424
4.325	1.810579	3.9438
4.35	1.810373	3.94441
4.375	1.810255	3.94418
4.4	1.809922	3.94318
4.425	1.809677	3.94188
4.45	1.809814	3.9401
4.475	1.810539	3.93836
4.5	1.811245	3.93884
4.525	1.811127	3.93999
4.55	1.809942	3.94049
4.575	1.80951	3.93982
4.6	1.810128	3.93966
4.625	1.810628	3.94062
4.65	1.809961	3.9415
4.675	1.809363	3.94158
4.7	1.809608	3.94114
4.725	1.810089	3.94088
4.75	1.80952	3.94073
4.775	1.80952	3.94129
4.8	1.809883	3.94084
4.825	1.809589	3.9413
4.85	1.808687	3.9412
4.875	1.808677	3.93999
4.9	1.809069	3.93947
4.925	1.808952	3.93937
4.95	1.808491	3.93994
4.975	1.808334	3.93884
5	1.808305	3.93854

Table S5. Test Data of Central Area Steering Condition

Time	ay	yawrate
0.01	0.165	0.13
0.02	-0.485	-0.08
0.03	-0.485	-0.08
0.04	0.78	0.135
0.05	0.78	0.135
0.06	-0.185	-0.13
0.07	-0.185	-0.13
0.08	-0.415	0.12
0.09	0.35	0.42

0.1	-0.185	-0.125
0.11	0.06	0.2
0.12	0.06	0.2
0.13	0.345	0.11
0.14	0.145	0.255
0.15	-0.25	-0.06
0.16	-0.25	-0.06
0.17	0.2	0.245
0.18	-0.25	0.07
0.19	0.625	0.295
0.2	-0.535	-0.045
0.21	0.13	-0.1
0.22	0.185	0.19
0.23	-0.18	-0.015
0.24	-0.065	-0.3
0.25	0.26	0.26
0.26	0.26	0.26
0.27	-0.145	0.09
0.28	0.11	-0.125
0.29	0.025	0.115
0.3	0.025	0.115
0.31	0.125	-0.105
0.32	0.465	0.155
0.33	-0.62	-0.225
0.34	0.41	0.27
0.35	0.41	0.27
0.36	0.54	-0.09
0.37	-0.37	-0.15
0.38	0.255	0.145
0.39	0.135	-0.055
0.4	-0.17	-0.125
0.41	-0.17	-0.125
0.42	-0.365	-0.14
0.43	0.305	-0.04
0.44	0.305	-0.04
0.45	0.12	0.125
0.46	0.12	0.125
0.47	0.415	0.09
0.48	-0.04	-0.105
0.49	0.075	0.08
0.5	-0.27	-0.135
0.51	-0.14	0.005
0.52	0.24	0.04
0.53	-0.09	0.025
0.54	-0.3	-0.12
0.55	-0.3	-0.12
0.56	-0.1	-0.065
0.57	0.215	0.17

0.58	-0.31	-0.01
0.59	-0.31	-0.01
0.6	-0.66	-0.41
0.61	0.25	0.11
0.62	0.415	0.185
0.63	-0.115	0.12
0.64	-0.375	-0.055
0.65	0.17	-0.105
0.66	0.17	-0.105
0.67	0.295	-0.01
0.68	-0.045	0.165
0.69	-0.045	0.165
0.7	0.335	0.09
0.71	0.335	0.09
0.72	0.075	0.15
0.73	-0.22	-0.11
0.74	-0.325	-0.35
0.75	0.95	0.265
0.76	-0.66	-0.185
0.77	0.28	-0.175
0.78	0.005	0.005
0.79	0	-0.035
0.8	0.25	0.135
0.81	0.25	0.135
0.82	-0.02	-0.12
0.83	0.43	-0.065
0.84	-0.065	-0.075
0.85	-0.065	-0.075
0.86	0.415	0.205
0.87	-0.555	-0.09
0.88	0.03	-0.195
0.89	0.51	0.27
0.9	-0.255	-0.005
0.91	-0.255	-0.005
0.92	0.62	0.08
0.93	0.07	0.055
0.94	0.07	0.055
0.95	-0.385	0.105
0.96	-0.09	-0.015
0.97	-0.7	-0.31
0.98	1.435	0.64
0.99	1.435	0.64
1	-0.045	-0.285
1.01	0.795	0.37
1.02	-0.4	-0.06
1.03	-0.185	-0.065
1.04	-0.185	-0.065
1.05	0.06	0.15

1.06	0.115	0.085
1.07	0.28	0.03
1.08	0.28	0.03
1.09	0.44	0.185
1.1	0.44	0.185
1.11	0.76	0.125
1.12	-0.34	0.11
1.13	-0.39	-0.325
1.14	0.37	0.045
1.15	0.37	0.045
1.16	-0.075	-0.21
1.17	-0.01	0.07
1.18	0.365	0.155
1.19	-0.03	0.215
1.2	-0.025	0.2
1.21	-0.025	0.2
1.22	-0.17	0.085
1.23	0.15	0.39
1.24	0.15	0.39
1.25	0.05	0.28
1.26	0.525	0.74
1.27	0.045	0.495
1.28	0.225	0.625
1.29	0.225	0.625
1.3	0.09	0.795
1.31	-0.035	0.695
1.32	-0.035	0.695
1.33	0.795	1.14
1.34	0.555	1.35
1.35	0.555	1.35
1.36	0.19	1.08
1.37	0.41	1.365
1.38	0.55	1.535
1.39	-0.08	1.085
1.4	-0.08	1.085
1.41	0.26	1.495
1.42	0.555	1.57
1.43	0.56	1.705
1.44	1.115	1.995
1.45	-0.42	1.435
1.46	1.47	2.02
1.47	1.47	2.02
1.48	0.755	2.315
1.49	0.27	1.825
1.5	1.37	2.225
1.51	0.34	2.135
1.52	0.895	2.32
1.53	1.215	2.355

1.54	0.73	2.585
1.55	0.73	2.585
1.56	0.805	2.325
1.57	0.91	2.735
1.58	0.91	2.735
1.59	1.245	2.55
1.6	1.245	2.55
1.61	0.625	2.67
1.62	1.295	2.79
1.63	1.585	3.19
1.64	1.585	3.19
1.65	0.935	3.095
1.66	0.935	3.095
1.67	1.54	3.065
1.68	1.55	3.36
1.69	0.465	3.105
1.7	1.675	3.51
1.71	1.675	3.51
1.72	1.295	3.435
1.73	1.35	3.57
1.74	0.845	3.405
1.75	1.64	3.805
1.76	1.64	3.805
1.77	0.835	3.38
1.78	2.035	4.01
1.79	1.185	3.825
1.8	1.46	3.935
1.81	1.46	3.935
1.82	1.355	3.92
1.83	1.355	3.92
1.84	1.31	4.065
1.85	1.925	4.49
1.86	0.595	3.885
1.87	2.815	4.43
1.88	2.815	4.43
1.89	2.15	4.735
1.9	1.425	4.345
1.91	1.45	4.44
1.92	1.465	4.46
1.93	2.02	4.6
1.94	2.255	4.865
1.95	1.365	4.57
1.96	1.55	4.46
1.97	1.55	4.46
1.98	1.66	4.63
1.99	1.66	4.63
2	2.165	4.855
2.01	1.56	4.715

2.02	1.85	4.6
2.03	1.9	4.685
2.04	2.295	4.995
2.05	2.295	4.995
2.06	1.485	4.705
2.07	2.035	4.895
2.08	1.815	5.045
2.09	1.815	5.045
2.1	1.81	5.095
2.11	2.31	5.31
2.12	1.155	4.745
2.13	2.61	5.22
2.14	2.61	5.22
2.15	2.15	5.19
2.16	2.15	5.19
2.17	1.46	5.135
2.18	1.46	5.135
2.19	2.28	5.255
2.2	2.23	5.34
2.21	1.835	5.23
2.22	1.84	5.105
2.23	2.89	5.57
2.24	1.015	4.9
2.25	2.435	5.51
2.26	2.21	5.275
2.27	2.21	5.275
2.28	1.085	5
2.29	3.125	5.54
2.3	1.3	5.125
2.31	2.585	5.42
2.32	2.585	5.42
2.33	1.54	5.36
2.34	1.605	4.975
2.35	2.855	5.64
2.36	1.675	5.3
2.37	2.635	5.705
2.38	1.46	4.975
2.39	2.695	5.665
2.4	2.695	5.665
2.41	2.285	5.605
2.42	2.38	5.665
2.43	1.87	5.425
2.44	1.87	5.425
2.45	2.3	5.555
2.46	1.985	5.29
2.47	2.475	5.83
2.48	1.795	5.455
2.49	2.59	5.665

2.5	1.315	5.105
2.51	3.165	5.815
2.52	3.165	5.815
2.53	2.475	5.59
2.54	2.685	5.52
2.55	1.395	4.915
2.56	1.395	4.915
2.57	2.345	5.3
2.58	2.125	5.445
2.59	1.265	4.855
2.6	1.265	4.855
2.61	2.04	5.35
2.62	2.01	5.115
2.63	2.2	5.26
2.64	1.99	5.31
2.65	2.33	5.3
2.66	1.955	5.055
2.67	1.9	5.065
2.68	2.485	5.51
2.69	2.485	5.51
2.7	2.89	5.38
2.71	2.89	5.38
2.72	1.89	5.275
2.73	1.92	4.81
2.74	2.305	5.03
2.75	2.305	5.03
2.76	1.775	4.925
2.77	1.775	4.925
2.78	1.875	4.925
2.79	2.44	5.06
2.8	1.495	4.6
2.81	2.065	4.68
2.82	2.175	4.845
2.83	1.93	4.71
2.84	1.71	4.435
2.85	1.685	4.525
2.86	2.2	4.755
2.87	1.785	4.56
2.88	1.415	4.35
2.89	2.36	4.455
2.9	1.51	4.235
2.91	1.585	4.18
2.92	2.33	4.49
2.93	2.33	4.49
2.94	1.75	4.16
2.95	1.65	4.045
2.96	1.485	3.93
2.97	1.81	3.955

2.98	2.03	3.965
2.99	2.03	3.965
3	1.77	3.92
3.01	1.33	3.615
3.02	1.805	3.94
3.03	1.3	3.605
3.04	1.3	3.605
3.05	1.27	3.515
3.06	2.17	3.735
3.07	1.15	3.255
3.08	1.15	3.255
3.09	1.795	3.385
3.1	1.25	3.21
3.11	0.87	3.145
3.12	0.87	3.145
3.13	1.58	3.115
3.14	1.54	3.02
3.15	1.315	2.865
3.16	1.6	2.965
3.17	0.795	2.54
3.18	1.955	2.955
3.19	1.955	2.955
3.2	1.255	2.56
3.21	1.76	2.865
3.22	0.085	1.975
3.23	0.085	1.975
3.24	1.14	2.37
3.25	1.4	2.49
3.26	0.455	2.035
3.27	1.005	2.055
3.28	0.875	1.785
3.29	0.875	1.785
3.3	1.15	2.085
3.31	0.995	1.89
3.32	0.995	1.89
3.33	1.03	1.72
3.34	0.675	1.51
3.35	0.935	1.72
3.36	1.15	1.765
3.37	1.15	1.765
3.38	1.17	1.605
3.39	-0.215	1.16
3.4	1.155	1.245
3.41	1.005	1.32
3.42	-0.04	0.945
3.43	-0.04	0.945
3.44	0.595	1.195
3.45	0.205	0.87

3.46	0.02	0.585
3.47	0.02	0.585
3.48	0.83	1.295
3.49	-0.885	0.28
3.5	1.59	0.975
3.51	1.59	0.975
3.52	-0.425	0.385
3.53	0.48	0.81
3.54	-0.365	0.16
3.55	0.665	0.565
3.56	0.665	0.565
3.57	0.265	0.265
3.58	0.265	0.265
3.59	0.08	0.075
3.6	0.08	0.075
3.61	0.535	0.12
3.62	0.09	-0.11
3.63	-0.305	-0.255
3.64	-0.045	-0.205
3.65	-0.045	-0.205
3.66	-0.14	-0.23
3.67	0.345	-0.235
3.68	-0.49	-0.6
3.69	-0.49	-0.6
3.7	0.625	-0.165
3.71	-0.585	-0.885
3.72	-0.005	-0.505
3.73	-0.125	-0.57
3.74	-0.125	-0.57
3.75	0.12	-0.97
3.76	-0.47	-0.89
3.77	-0.2	-0.925
3.78	-0.2	-0.925
3.79	-0.51	-1.2
3.8	-0.44	-1.38
3.81	-0.3	-1.015
3.82	-1.01	-1.69
3.83	-1.01	-1.69
3.84	-0.83	-1.505
3.85	-0.825	-1.875
3.86	0.05	-1.43
3.87	0.05	-1.43
3.88	-0.61	-1.715
3.89	-1.705	-2.49
3.9	0.475	-1.72
3.91	-1.045	-2.07
3.92	-0.71	-2.02
3.93	-0.66	-2.24

3.94	-0.66	-2.24
3.95	-1.15	-2.145
3.96	-0.77	-2.21
3.97	-0.82	-2.455
3.98	-1.13	-2.575
3.99	-1.13	-2.575
4	-1.015	-2.7
4.01	-0.56	-2.565
4.02	-1.095	-2.715
4.03	-0.81	-2.735
4.04	-1.5	-3.06
4.05	-0.485	-2.8
4.06	-0.965	-2.855
4.07	-1.745	-3.32
4.08	-0.725	-3.175
4.09	-1.375	-3.26
4.1	-0.97	-3.13
4.11	-1.555	-3.675
4.12	-1.555	-3.675
4.13	-0.845	-3.28
4.14	-1.52	-3.53
4.15	-1.535	-3.715
4.16	-1.41	-3.615
4.17	-1.27	-3.75
4.18	-1.27	-3.75
4.19	-1.365	-3.84
4.2	-1.8	-4.01
4.21	-1.52	-4.01
4.22	-1.085	-3.855
4.23	-1.845	-4.09
4.24	-1.845	-4.09
4.25	-1.575	-4.1
4.26	-1.76	-4.43
4.27	-1.465	-4.195
4.28	-1.36	-4.215
4.29	-1.36	-4.215
4.3	-1.59	-4.4
4.31	-0.985	-3.96
4.32	-2.595	-4.755
4.33	-2.595	-4.755
4.34	-1.545	-4.56
4.35	-0.92	-4.385
4.36	-1.86	-4.515
4.37	-1.86	-4.515
4.38	-2.055	-4.76
4.39	-1.825	-4.765
4.4	-1.53	-4.645
4.41	-1.94	-4.93

4.42	-1.775	-4.76
4.43	-2.41	-5.1
4.44	-1.205	-4.955
4.45	-2.29	-5.19
4.46	-1.62	-4.975
4.47	-2.1	-5.355
4.48	-1.91	-5.23
4.49	-1.91	-5.23
4.5	-1.76	-5.16
4.51	-2.38	-5.245
4.52	-2.38	-5.245
4.53	-1.69	-5.28
4.54	-2.805	-5.82
4.55	-2.805	-5.82
4.56	-1.65	-5.25
4.57	-2.31	-5.375
4.58	-1.955	-5.285
4.59	-2.37	-5.34
4.6	-2.28	-5.56
4.61	-1.89	-5.39
4.62	-2.045	-5.265
4.63	-2.505	-5.76
4.64	-1.5	-5.205
4.65	-2.74	-5.895
4.66	-1.225	-5.13
4.67	-2.485	-5.45
4.68	-2.485	-5.45
4.69	-2.29	-5.44
4.7	-2.085	-5.975
4.71	-2.085	-5.975
4.72	-2.56	-5.54
4.73	-2.29	-5.785
4.74	-2.08	-5.805
4.75	-1.93	-5.57
4.76	-1.93	-5.57
4.77	-2.335	-5.77
4.78	-2.325	-5.91
4.79	-1.835	-5.84
4.8	-2.43	-5.78
4.81	-2.33	-5.795
4.82	-2.145	-6.045
4.83	-2.145	-6.045
4.84	-2.25	-5.805
4.85	-1.96	-5.615
4.86	-3.045	-6.08
4.87	-1.81	-5.75
4.88	-2.22	-5.93
4.89	-2.46	-5.68

4.9	-2.46	-5.68
4.91	-2.335	-6.155
4.92	-2.21	-5.645
4.93	-1.855	-5.63
4.94	-2.59	-5.87
4.95	-2.865	-5.995
4.96	-1.62	-5.845
4.97	-1.62	-5.845
4.98	-2.325	-5.9
4.99	-2.325	-5.9
5	-2.065	-5.94
5.01	-2.5	-5.875
5.02	-1.725	-5.74
5.03	-3.025	-6.14
5.04	-3.025	-6.14
5.05	-2.27	-5.86
5.06	-2.395	-6.045
5.07	-2.48	-5.91
5.08	-2.48	-5.91
5.09	-2.06	-5.695
5.1	-2.16	-5.555
5.11	-2.88	-6.135
5.12	-2.88	-6.135
5.13	-1.925	-5.7
5.14	-1.93	-5.52
5.15	-2.72	-5.845
5.16	-2.225	-5.575
5.17	-2.365	-5.81
5.18	-2.365	-5.81
5.19	-1.93	-5.575
5.2	-2.705	-5.685
5.21	-2.395	-5.75
5.22	-2.395	-5.75
5.23	-2.05	-5.39
5.24	-2.345	-5.37
5.25	-2.755	-5.83
5.26	-2.755	-5.83
5.27	-2.925	-5.66
5.28	-1.865	-5.385
5.29	-2.14	-5.075
5.3	-2.375	-5.38
5.31	-1.845	-5.085
5.32	-1.845	-5.085
5.33	-2.455	-5.42
5.34	-1.73	-5.15
5.35	-1.645	-4.925
5.36	-1.645	-4.925
5.37	-2.135	-5.19

5.38	-2.135	-5.19
5.39	-1.75	-4.96
5.4	-1.535	-4.74
5.41	-2.31	-4.94
5.42	-2.32	-5.065
5.43	-1.505	-4.86
5.44	-1.605	-4.53
5.45	-2.42	-4.935
5.46	-1.755	-4.775
5.47	-1.755	-4.775
5.48	-1.755	-4.515
5.49	-2.05	-4.74
5.5	-1.645	-4.155
5.51	-1.645	-4.155
5.52	-1.7	-4.44
5.53	-2.24	-4.45
5.54	-1.245	-4.165
5.55	-1.245	-4.165
5.56	-1.675	-3.985
5.57	-1.445	-3.86
5.58	-1.94	-4.14
5.59	-1.94	-4.14
5.6	-1.42	-3.88
5.61	-0.91	-3.46
5.62	-1.74	-3.62
5.63	-1.575	-3.78
5.64	-1.805	-3.71
5.65	-1.265	-3.35
5.66	-1.265	-3.35
5.67	-1.16	-3.32
5.68	-1.695	-3.34
5.69	-1.875	-3.45
5.7	-1.875	-3.45
5.71	-1.02	-2.885
5.72	-1.79	-3.215
5.73	-1.01	-2.8
5.74	-1.665	-2.965
5.75	-1.665	-2.965
5.76	-2	-3.205
5.77	-0.565	-2.495
5.78	-1.35	-2.425
5.79	-1.32	-2.68
5.8	-1.32	-2.68
5.81	-1.36	-2.615
5.82	-0.995	-2.455
5.83	-0.45	-1.88
5.84	-0.45	-1.88
5.85	-0.295	-1.925

5.86	-0.975	-2.135
5.87	-1.57	-2.235
5.88	-1.57	-2.235
5.89	-1.155	-2.105
5.9	-0.795	-1.96
5.91	-0.92	-1.785
5.92	-0.67	-1.57
5.93	-0.985	-1.805
5.94	-0.985	-1.805
5.95	-0.83	-1.51
5.96	-0.83	-1.51
5.97	-1	-1.585
5.98	-0.15	-0.965
5.99	-1.685	-1.5
6	-0.25	-1.17
6.01	-0.25	-1.17
6.02	-0.5	-1.19
6.03	-0.605	-1.12
6.04	-0.735	-0.985
6.05	-0.065	-0.695
6.06	-0.69	-0.985
6.07	-0.69	-0.985
6.08	0.1	-0.48
6.09	0.305	-0.41
6.1	0.305	-0.41
6.11	-0.285	-0.24
6.12	0.425	-0.12
6.13	-0.73	-0.715
6.14	0.26	-0.035
6.15	0.26	-0.035
6.16	0.14	-0.1
6.17	-0.625	-0.13
6.18	0.015	0.065
6.19	0.015	0.065
6.2	0.04	0.005
6.21	-0.06	0.41
6.22	0.13	0.405
6.23	-0.325	0.005
6.24	-0.325	0.005
6.25	0.355	0.74
6.26	-0.425	0.3
6.27	0.305	0.72
6.28	0.35	0.485
6.29	0.35	0.485
6.3	0.495	0.905
6.31	0.235	0.525
6.32	0.815	1.05
6.33	-0.035	0.99

6.34	0.625	1.185
6.35	0.03	0.87
6.36	0.495	1.425
6.37	0.365	1.115
6.38	0.91	1.56
6.39	-0.61	1.175
6.4	1.64	1.85
6.41	1.64	1.85
6.42	0.645	1.6
6.43	1.565	2.135
6.44	1.565	2.135
6.45	1.16	1.96
6.46	0.81	2.22
6.47	0.36	1.785
6.48	0.36	1.785
6.49	1.17	2.195
6.5	0.915	2.395
6.51	0.06	1.875
6.52	1.22	2.35
6.53	1.22	2.35
6.54	0.615	2.325
6.55	1.045	2.435
6.56	1.045	2.435
6.57	1.04	2.62
6.58	1.04	2.62
6.59	0.9	2.69
6.6	1.06	2.815
6.61	0.705	2.69
6.62	0.705	2.69
6.63	0.635	2.705
6.64	1.325	2.9
6.65	1.06	2.895
6.66	1.06	2.895
6.67	1.695	3.55
6.68	1.04	2.98
6.69	1.04	2.98
6.7	1.385	3.415
6.71	0.82	3.295
6.72	1.295	3.49
6.73	1.295	3.49
6.74	1.2	3.285
6.75	1.555	3.49
6.76	1.35	3.705
6.77	1.235	3.6
6.78	1.53	3.71
6.79	1.53	3.71
6.8	1.645	3.915
6.81	1.565	4.015

6.82	1.075	3.81
6.83	1.455	3.88
6.84	1.36	3.94
6.85	1.7	4.06
6.86	1.465	4.15
6.87	1.465	4.15
6.88	1.24	4.06
6.89	1.59	4.19
6.9	2.015	4.495
6.91	1.475	4.425
6.92	0.79	3.89
6.93	1.94	4.38
6.94	1.94	4.38
6.95	1.735	4.65
6.96	1.555	4.57
6.97	1.185	4.39
6.98	2.175	4.715
6.99	1.675	4.7
7	1.485	4.525
7.01	2.05	4.825
7.02	2.05	4.825
7.03	2.105	5.125
7.04	1.22	4.46
7.05	2.725	5.09
7.06	2.725	5.09
7.07	1.415	4.97
7.08	1.745	4.83
7.09	1.935	4.845
7.1	1.89	5.12
7.11	1.495	4.71
7.12	1.495	4.71
7.13	1.625	4.975
7.14	1.94	5.17
7.15	1.765	5.005
7.16	1.825	5.04
7.17	1.91	4.925
7.18	2.665	5.68
7.19	1.2	5.035
7.2	2.435	5.37
7.21	1.71	4.975
7.22	2.465	5.585
7.23	1.885	5.31
7.24	1.66	5.335
7.25	2.175	5.19
7.26	1.89	5.36
7.27	2.315	5.415
7.28	2.315	5.415
7.29	2.205	5.435

7.3	2.205	5.435
7.31	2.005	5.185
7.32	2.265	5.51
7.33	2.21	5.54
7.34	2.21	5.54
7.35	2.55	5.61
7.36	1.94	5.43
7.37	1.955	5.505
7.38	2.055	5.365
7.39	2.17	5.355
7.4	2.815	5.79
7.41	1.335	5.275
7.42	1.955	5.4
7.43	2.505	5.56
7.44	2.505	5.56
7.45	2.025	5.365
7.46	2.495	5.67
7.47	2.495	5.67
7.48	1.805	5.015
7.49	2.495	5.54
7.5	1.76	5.275
7.51	2.865	5.735
7.52	1.74	5.35
7.53	1.785	5.15
7.54	2.415	5.305
7.55	2.415	5.305
7.56	1.95	5.29
7.57	2.055	5.24
7.58	2.195	5.335
7.59	2.14	5.435
7.6	2.14	5.435
7.61	2.25	5.275
7.62	1.725	5.015
7.63	1.725	5.015
7.64	2.55	5.58
7.65	1.4	5.115
7.66	2.5	5.31
7.67	1.51	5.04
7.68	2.445	5.38
7.69	1.58	5.215
7.7	2.585	5.46
7.71	2.585	5.46
7.72	2.455	5.11
7.73	2.455	5.11
7.74	2.175	5.24
7.75	1.63	4.78
7.76	1.995	5.145
7.77	1.995	5.1

7.78	1.995	5.1
7.79	2.455	5.215
7.8	1.385	4.74
7.81	1.385	4.74
7.82	1.365	4.735
7.83	2.255	4.8
7.84	1.8	4.6
7.85	1.8	4.6
7.86	2.03	4.89
7.87	1.64	4.37
7.88	2.135	4.715
7.89	1.24	4.13
7.9	2.625	4.715
7.91	1.285	4.265
7.92	2.14	4.39
7.93	1.625	4.165
7.94	1.625	4.165
7.95	2.47	4.54
7.96	1.35	3.94
7.97	1.815	3.93
7.98	1.32	3.805
7.99	1.32	3.805
8	1.42	3.64
8.01	1.645	3.62
8.02	1.655	3.485
8.03	1.785	3.99
8.04	1.785	3.99
8.05	1.165	3.585
8.06	0.985	3.1
8.07	0.985	3.1
8.08	1.945	3.665
8.09	1.615	3.25
8.1	2.21	3.68
8.11	0.035	3
8.12	0.035	3
8.13	1.87	3.225
8.14	1.165	3.095
8.15	0.76	2.62
8.16	1.695	3.09
8.17	1.325	3.04
8.18	1.325	3.04
8.19	1.78	2.73
8.2	0.515	2.475
8.21	1.53	2.645
8.22	1	2.72
8.23	1.08	2.445
8.24	1.08	2.445
8.25	0.465	2.16

8.26	1.435	2.385
8.27	0.69	2.095
8.28	1.725	2.745
8.29	1.725	2.745
8.3	1.16	1.995
8.31	1.13	2.03
8.32	0.45	1.83
8.33	0.89	1.885
8.34	0.645	1.675
8.35	1.015	1.77
8.36	0.855	1.795
8.37	0.675	1.74
8.38	0.675	1.74
8.39	0.1	1.295
8.4	0.685	1.365
8.41	0.84	1.355
8.42	0.52	1.155
8.43	0.52	1.155
8.44	0.085	1.27
8.45	0.85	1.11
8.46	0.4	0.99
8.47	0.755	1.195
8.48	0.055	1.09
8.49	0.325	0.7
8.5	0.835	0.865
8.51	0.835	0.865
8.52	-0.435	0.305
8.53	0.39	0.63
8.54	0.29	0.595
8.55	0.35	0.51
8.56	0.35	0.51
8.57	-0.11	0.155
8.58	-0.11	0.155
8.59	1.05	0.615
8.6	-0.46	-0.105
8.61	-0.46	-0.105
8.62	-0.31	-0.165
8.63	0.445	0.07
8.64	-0.11	-0.235
8.65	-0.19	-0.32
8.66	0.575	0.085
8.67	-0.735	-0.57
8.68	0.585	-0.09
8.69	-0.685	-0.86
8.7	0.41	-0.08
8.71	0.41	-0.08
8.72	-0.715	-0.905
8.73	-1.28	-0.915

8.74	-0.215	-0.62
8.75	-0.215	-0.62
8.76	-0.47	-1.075
8.77	-0.515	-0.91
8.78	-0.38	-1.215
8.79	0.635	-0.53
8.8	-1.285	-1.325
8.81	0.43	-0.855
8.82	0.43	-0.855
8.83	-1.07	-1.395
8.84	0.03	-1.08
8.85	-0.645	-1.44
8.86	-0.66	-1.745
8.87	0.075	-1.35
8.88	-0.67	-1.56
8.89	-0.56	-1.815
8.9	0.03	-1.43
8.91	-0.98	-1.89
8.92	-0.815	-1.935
8.93	-0.375	-1.91
8.94	-0.83	-2.065
8.95	-0.76	-2.465
8.96	-0.17	-1.97
8.97	-0.17	-1.97
8.98	-0.595	-2.46
8.99	-0.32	-2.195
9	-1.285	-2.63
9.01	-1.285	-2.63
9.02	-0.395	-2.59
9.03	-1.225	-2.915
9.04	-0.45	-2.6
9.05	-0.45	-2.6
9.06	-0.38	-2.845
9.07	-1.065	-2.875
9.08	-0.995	-3.005
9.09	-0.995	-3.005
9.1	-1.115	-3.19
9.11	-1.105	-3.04
9.12	-1.565	-3.525
9.13	-0.85	-3.345
9.14	-1.17	-3.425
9.15	-1.395	-3.5
9.16	-1.11	-3.45
9.17	-1.11	-3.575
9.18	-1.335	-3.565
9.19	-1.335	-3.565
9.2	-0.76	-3.46
9.21	-2.08	-3.925

9.22	-1.11	-3.985
9.23	-1.11	-3.985
9.24	-1.62	-4.045
9.25	-0.97	-3.675
9.26	-1.995	-4.28
9.27	-1.995	-4.28
9.28	-1.27	-3.87
9.29	-1.925	-4.175
9.3	-1.6	-4.39
9.31	-1.6	-4.39
9.32	-1.665	-4.62
9.33	-1.34	-4.185
9.34	-1.775	-4.425
9.35	-1.405	-4.495
9.36	-1.405	-4.495
9.37	-2.125	-4.755
9.38	-1.565	-4.765
9.39	-1.41	-4.38
9.4	-2.135	-5.01
9.41	-2.135	-5.01
9.42	-2.175	-4.9
9.43	-1.35	-4.555
9.44	-1.93	-4.965
9.45	-2.065	-4.845
9.46	-2.105	-4.99
9.47	-2.105	-4.99
9.48	-1.395	-4.845
9.49	-1.395	-4.845
9.5	-2.41	-5.11
9.51	-1.52	-5.01
9.52	-1.52	-5.01
9.53	-2.01	-5.15
9.54	-2.455	-5.255
9.55	-1.595	-5.135
9.56	-1.595	-5.135
9.57	-2.095	-5.335
9.58	-2.095	-5.335
9.59	-1.71	-5.26
9.6	-2.08	-5.4
9.61	-2.115	-5.365
9.62	-1.95	-5.425
9.63	-2.065	-5.455
9.64	-2.065	-5.455
9.65	-2.17	-5.675
9.66	-1.76	-5.465
9.67	-2.435	-5.935
9.68	-1.775	-5.365
9.69	-2.455	-5.825

9.7	-1.905	-5.745
9.71	-1.91	-5.42
9.72	-2.68	-5.88
9.73	-2.34	-5.845
9.74	-2.34	-5.845
9.75	-2.23	-5.59
9.76	-2.635	-5.81
9.77	-2.635	-5.81
9.78	-2.515	-5.745
9.79	-2.3	-5.72
9.8	-2.065	-5.49
9.81	-2.375	-5.755
9.82	-2.375	-5.755
9.83	-2.22	-5.72
9.84	-1.99	-5.575
9.85	-2.34	-5.71
9.86	-2.105	-5.725
9.87	-1.97	-5.565
9.88	-1.97	-5.565
9.89	-1.795	-5.735
9.9	-2.5	-5.9
9.91	-2.5	-5.9
9.92	-1.795	-5.49
9.93	-2.445	-5.82
9.94	-2.455	-5.93
9.95	-2.17	-5.71
9.96	-2.155	-5.93
9.97	-2.005	-5.86
9.98	-2.555	-6.035
9.99	-2.555	-6.035
10	-2.17	-5.65
10.01	-1.955	-5.8
10.02	-1.955	-5.8
10.03	-3.095	-6.045
10.04	-1.7	-5.595
10.05	-1.7	-5.595
10.06	-2.11	-5.96
10.07	-2.265	-5.67
10.08	-2.265	-5.67
10.09	-2.32	-5.785
10.1	-1.865	-5.625
10.11	-2.72	-5.9
10.12	-2.72	-5.9
10.13	-2.615	-5.92
10.14	-1.635	-5.415
10.15	-2.685	-5.94
10.16	-2.685	-5.94
10.17	-2.695	-5.72

10.18	-1.99	-5.6
10.19	-1.72	-5.395
10.2	-2.915	-5.74
10.21	-1.985	-5.475
10.22	-1.88	-5.405
10.23	-1.88	-5.405
10.24	-2.17	-5.21
10.25	-2.055	-5.37
10.26	-2.635	-5.59
10.27	-2.01	-5.365
10.28	-2.01	-5.365
10.29	-3.145	-5.46
10.3	-3.145	-5.46
10.31	-2.095	-5.175
10.32	-2.315	-5.065
10.33	-2.03	-5.115
10.34	-2.03	-5.115
10.35	-2.525	-4.99
10.36	-1.025	-4.48
10.37	-2.845	-5.265
10.38	-2.015	-5.18
10.39	-1.3	-4.585
10.4	-1.3	-4.585
10.41	-2.22	-4.9
10.42	-2	-4.89
10.43	-1.49	-4.525
10.44	-2.185	-4.57
10.45	-2.185	-4.57
10.46	-2.115	-4.795
10.47	-2.115	-4.795
10.48	-1.51	-4.415
10.49	-1.585	-4.245
10.5	-1.855	-4.345
10.51	-1.855	-4.345
10.52	-1.655	-4.335
10.53	-1.885	-4.09
10.54	-1.55	-4.06
10.55	-1.485	-3.98
10.56	-2.49	-4.415
10.57	-0.805	-3.745
10.58	-1.565	-3.555
10.59	-1.565	-3.555
10.6	-1.265	-3.62
10.61	-1.485	-3.66
10.62	-1.765	-3.78
10.63	-1.6	-3.76
10.64	-0.93	-3.57
10.65	-0.93	-3.57

10.66	-2.045	-3.6
10.67	-1.64	-3.285
10.68	-1.305	-3.245
10.69	-1.305	-3.245
10.7	-1.62	-3.35
10.71	-1.125	-3.005
10.72	-1.125	-3.005
10.73	-1.695	-2.985
10.74	-1.475	-3.16
10.75	-0.905	-2.775
10.76	-0.905	-2.775
10.77	-0.91	-2.68
10.78	-1.255	-2.82
10.79	-0.55	-2.32
10.8	-0.55	-2.32
10.81	-1.055	-2.33
10.82	-1.21	-2.345
10.83	-0.925	-2.195
10.84	-0.925	-2.195
10.85	-0.855	-2.045
10.86	-0.755	-1.99
10.87	-1.225	-2.145
10.88	-0.795	-1.825
10.89	-0.66	-1.77
10.9	-0.145	-1.35
10.91	-1.76	-1.87
10.92	-0.72	-1.755
10.93	-0.72	-1.755
10.94	-0.5	-1.305
10.95	-0.51	-1.425
10.96	-0.95	-1.27
10.97	-1.29	-1.52
10.98	0.685	-0.86
10.99	0.685	-0.86
11	-0.675	-1.07
11.01	-0.55	-1.065
11.02	0.055	-0.72
11.03	0.055	-0.72
11.04	0.015	-0.635
11.05	0.015	-0.635
11.06	-0.82	-0.7
11.07	0.155	-0.27
11.08	-0.61	-0.855
11.09	-0.61	-0.855
11.1	0.285	-0.19
11.11	-0.41	-0.26
11.12	-0.66	-0.475
11.13	0.09	-0.23

11.14	0.08	-0.18
11.15	0.08	-0.18
11.16	0.425	0.295
11.17	-0.27	0.015
11.18	-0.64	0.01
11.19	0.44	0.11
11.2	-0.09	0.005
11.21	-0.09	0.005
11.22	-0.35	0.18
11.23	-0.055	0.36
11.24	-0.32	0.235
11.25	0.525	0.525
11.26	0.005	0.34
11.27	0.005	0.34
11.28	-0.34	0.775
11.29	-0.16	0.61
11.3	0.795	0.995
11.31	0.795	0.995
11.32	-0.04	0.615
11.33	-0.055	1.2
11.34	0.415	1.175
11.35	0.3	1.055
11.36	0.335	1.18
11.37	0.65	1.54
11.38	0.65	1.54
11.39	0.6	1.655
11.4	0.185	1.655
11.41	0.05	1.275
11.42	0.05	1.275
11.43	1.165	2.035
11.44	1.165	2.035
11.45	0.78	1.825
11.46	1.32	2.315
11.47	0.13	1.965
11.48	0.295	1.7
11.49	0.295	1.7
11.5	0.94	2.37
11.51	0.47	2.215
11.52	1.12	2.28
11.53	1.12	2.28
11.54	1.05	2.345
11.55	0.705	2.525
11.56	0.665	2.45
11.57	0.665	2.45
11.58	0.885	2.55
11.59	0.435	2.495
11.6	1.46	2.755
11.61	1.46	2.755

11.62	0.865	2.665
11.63	0.865	2.665
11.64	1.45	3.085
11.65	0.77	2.81
11.66	1.515	3.125
11.67	0.97	3.175
11.68	0.95	3.085
11.69	0.95	3.085
11.7	1.58	3.285
11.71	1.225	3.23
11.72	1.23	3.505
11.73	1.225	3.485
11.74	1.285	3.64
11.75	1.065	3.41
11.76	1.39	3.515
11.77	1.485	3.795
11.78	0.83	3.395
11.79	0.83	3.395
11.8	0.735	3.7
11.81	1.41	3.725
11.82	2.28	4.535
11.83	0.585	3.695
11.84	1.67	3.97
11.85	1.42	4.04
11.86	1.995	4.555
11.87	1.995	4.555
11.88	2.22	4.54
11.89	0.575	3.785
11.9	2.08	4.465
11.91	1.29	4.225
11.92	1.98	4.455
11.93	1.98	4.455
11.94	1.675	4.6
11.95	1.585	4.61
11.96	1.74	4.475
11.97	2.05	4.93
11.98	1.22	4.355
11.99	2.145	4.895
12	1.565	4.84
12.01	1.565	4.84
12.02	2.195	4.885
12.03	1.1	4.57
12.04	1.1	4.57
12.05	2.17	5.025
12.06	1.3	4.715
12.07	2.315	5.08
12.08	2.315	5.08
12.09	2.195	5.2

12.1	1.56	5.095
12.11	1.985	4.905
12.12	1.985	4.905
12.13	1.875	5.12
12.14	1.86	4.93
12.15	2.095	5.31
12.16	1.515	4.99
12.17	2.405	5.42
12.18	2.405	5.42
12.19	1.08	4.93
12.2	2.51	5.33
12.21	2.02	5.325
12.22	2.02	5.325
12.23	2.01	5.435
12.24	2.01	5.435
12.25	1.805	5.26
12.26	1.805	5.26
12.27	2.62	5.63
12.28	1.885	5.375
12.29	1.95	5.1
12.3	2.365	5.415
12.31	2.61	5.82
12.32	1.135	5.115
12.33	1.135	5.115
12.34	2.19	5.615
12.35	1.945	5.415
12.36	2.515	5.7
12.37	2.515	5.7
12.38	2.05	5.44
12.39	2.45	5.475
12.4	1.745	5.25
12.41	2.535	5.53
12.42	1.95	5.52
12.43	2.09	5.495
12.44	1.9	5.255
12.45	2.715	5.565
12.46	1.565	5.27
12.47	2.625	5.615
12.48	1.71	5.33
12.49	1.71	5.33
12.5	1.825	5.05
12.51	2.495	5.45
12.52	2.23	5.55
12.53	2.23	5.55
12.54	1.925	5.33
12.55	2.215	5.26
12.56	2.195	5.315
12.57	2.195	5.315

12.58	2.495	5.56
12.59	1.68	5.08
12.6	1.95	5.245
12.61	2.385	5.38
12.62	2.385	5.38
12.63	1.56	5.285
12.64	2.195	5.185
12.65	2.28	5.525
12.66	1.8	5.355
12.67	1.8	5.355
12.68	1.845	5.16
12.69	1.845	5.16
12.7	1.805	5.265
12.71	1.95	5.115
12.72	1.925	4.835
12.73	1.925	4.835
12.74	1.625	5.105
12.75	2.22	5.005
12.76	1.91	4.945
12.77	2.12	5.095
12.78	1.91	4.98
12.79	2.305	5.24
12.8	2.305	5.24
12.81	2.465	4.965
12.82	1.735	4.8
12.83	1.765	4.735
12.84	1.765	4.735
12.85	1.03	4.33
12.86	2.39	4.62
12.87	2.01	4.815
12.88	2	4.685
12.89	1.74	4.635
12.9	1.615	4.37
12.91	2.07	4.23
12.92	1.985	4.62
12.93	1.635	4.195
12.94	2.075	4.3
12.95	1.63	4.255
12.96	1.98	4.34
12.97	1.56	4.14
12.98	1.56	4.14
12.99	2.28	4.225
13	1.495	4.05
13.01	1.22	3.735
13.02	1.78	3.74
13.03	1.895	3.94
13.04	1.895	3.94
13.05	1.265	3.615

13.06	2.09	3.89
13.07	0.885	3.54
13.08	0.885	3.54
13.09	1.3	3.615
13.1	1.35	3.39
13.11	1.685	3.475
13.12	1.685	3.475
13.13	1.31	3.37
13.14	1.94	3.465
13.15	1.64	3.63
13.16	0.55	2.92
13.17	1.8	3.305
13.18	1.125	3.105
13.19	1.075	2.82
13.2	1.075	2.82
13.21	1.33	2.85
13.22	1.46	2.82
13.23	1.235	2.965
13.24	0.86	2.53
13.25	1.005	2.445
13.26	1.605	2.67
13.27	1.605	2.67
13.28	1.09	2.4
13.29	1.035	2.375
13.3	0.67	1.915
13.31	1.905	2.625
13.32	1.905	2.625
13.33	-0.09	1.7
13.34	0.425	1.765
13.35	1.195	2.09
13.36	0.72	1.81
13.37	0.855	1.65
13.38	0.855	1.65
13.39	1.325	1.815
13.4	0.51	1.575
13.41	0.71	1.515
13.42	0.49	1.475
13.43	0.49	1.475
13.44	0.285	1.23
13.45	1.025	1.465
13.46	1.025	1.465
13.47	0.45	1.195
13.48	0.755	1.055
13.49	0.755	1.055
13.5	-0.045	1.095
13.51	0.03	0.61
13.52	0.995	0.85
13.53	0.04	0.69

13.54	0.04	0.69
13.55	0.42	0.85
13.56	-0.28	0.535
13.57	1.305	0.89
13.58	1.305	0.89
13.59	0.525	0.615
13.6	0.525	0.615
13.61	0.385	0.405
13.62	0.54	0.625
13.63	-0.405	0.02
13.64	0.45	0.405
13.65	0.45	0.405
13.66	0.72	0.315
13.67	0.095	0.055
13.68	-0.495	-0.045
13.69	-0.495	-0.045
13.7	0.43	-0.205
13.71	-0.44	-0.335
13.72	0.65	0.13
13.73	-1.265	-0.95
13.74	0.8	-0.245
13.75	-0.17	-0.665
13.76	-0.49	-0.6
13.77	-0.065	-0.435
13.78	0.285	-0.685
13.79	-0.745	-0.985
13.8	-0.745	-0.985
13.81	-0.015	-0.89
13.82	-0.605	-1.29
13.83	0.595	-0.55
13.84	0.595	-0.55
13.85	-0.06	-0.96
13.86	-0.72	-1.24
13.87	-0.27	-1.335
13.88	-0.655	-1.55
13.89	-0.285	-1.365
13.9	-0.285	-1.365
13.91	-0.735	-1.675
13.92	-0.455	-1.665
13.93	-0.44	-1.675
13.94	-0.78	-1.92
13.95	-0.59	-1.9
13.96	-0.635	-2.08
13.97	-0.88	-2.215
13.98	-0.88	-2.215
13.99	-0.83	-2.195
14	-1.355	-2.82
14.01	0.33	-2.085

14.02	-1.075	-2.385
14.03	-1.205	-2.475
14.04	-0.645	-2.33
14.05	-0.645	-2.33
14.06	-0.76	-2.695
14.07	-0.645	-2.455
14.08	-1.595	-2.95
14.09	-0.665	-3.005
14.1	-0.665	-3.005
14.11	-1.225	-3.085
14.12	-1.56	-3.13
14.13	-0.545	-2.835
14.14	-1.85	-3.565
14.15	-0.805	-3.43
14.16	-0.805	-3.43
14.17	-1.865	-3.76
14.18	-1.865	-3.76
14.19	-0.29	-3.14
14.2	-1.795	-3.695
14.21	-1.555	-3.415
14.22	-1.79	-3.93
14.23	-1.79	-3.93
14.24	-1.63	-3.67
14.25	-1.33	-3.905
14.26	-1.37	-3.765
14.27	-1.94	-4.22
14.28	-0.76	-3.885
14.29	-0.76	-3.885
14.3	-1.62	-3.915
14.31	-1.15	-3.87
14.32	-1.725	-4.065
14.33	-1.695	-4.39
14.34	-1.695	-4.39
14.35	-1.695	-4.4
14.36	-1.73	-4.56
14.37	-1.73	-4.56
14.38	-2.54	-4.89
14.39	-2.54	-4.89
14.4	-1.58	-4.435
14.41	-1.58	-4.435
14.42	-1.65	-4.685
14.43	-1.59	-4.595
14.44	-2.085	-4.79
14.45	-1.695	-5.13
14.46	-1.685	-4.96
14.47	-1.735	-4.78
14.48	-1.735	-4.78
14.49	-1.975	-5.2

14.5	-1.975	-5.2
14.51	-1.78	-4.83
14.52	-1.78	-4.83
14.53	-1.575	-4.895
14.54	-2.02	-5.165
14.55	-2.165	-5.42
14.56	-2.02	-5.27
14.57	-1.945	-5.29
14.58	-1.945	-5.29
14.59	-2.96	-5.585
14.6	-1.495	-5.085
14.61	-2.61	-5.63
14.62	-1.48	-5.455
14.63	-1.54	-4.975
14.64	-3.08	-5.8
14.65	-1.555	-5.335
14.66	-2.305	-5.605
14.67	-1.58	-5.195
14.68	-1.58	-5.195
14.69	-2.705	-5.825
14.7	-2.03	-5.365
14.71	-2.495	-5.615
14.72	-1.81	-5.61
14.73	-2.33	-5.565
14.74	-2.225	-5.525
14.75	-1.855	-5.475
14.76	-2.56	-5.625
14.77	-1.805	-5.44
14.78	-1.805	-5.44
14.79	-1.875	-5.44
14.8	-2.4	-5.555
14.81	-1.785	-5.6
14.82	-2.39	-5.645
14.83	-2.91	-5.835
14.84	-1.575	-5.59
14.85	-1.575	-5.59
14.86	-2.33	-5.7
14.87	-2.37	-5.64
14.88	-1.83	-5.575
14.89	-2.265	-5.86
14.9	-2.425	-5.73
14.91	-2.425	-5.73
14.92	-2.96	-5.86
14.93	-2.03	-5.78
14.94	-1.84	-5.61
14.95	-2.69	-5.775
14.96	-2.69	-5.775
14.97	-1.815	-5.57

14.98	-2.375	-5.955
14.99	-2.38	-5.845
15	-2.005	-5.695
15.01	-2.535	-5.84
15.02	-2.535	-5.84
15.03	-1.83	-5.61
15.04	-2.68	-5.805
15.05	-2.105	-5.655
15.06	-2.295	-5.66
15.07	-2.295	-5.66
15.08	-2.68	-5.875
15.09	-2.295	-5.79
15.1	-2.245	-5.525
15.11	-1.725	-5.435
15.12	-2.84	-6.015
15.13	-2.84	-6.015
15.14	-2.355	-5.605
15.15	-2.42	-5.81
15.16	-2.42	-5.81
15.17	-2.53	-5.495
15.18	-2.25	-5.61
15.19	-1.79	-5.18
15.2	-2.24	-5.475
15.21	-1.795	-5.13
15.22	-1.795	-5.13
15.23	-2.285	-5.46
15.24	-1.565	-4.975
15.25	-2.14	-5.015
15.26	-2.29	-5.275
15.27	-2.29	-5.275
15.28	-2.135	-5.06
15.29	-2.03	-5
15.3	-1.73	-4.92
15.31	-2.605	-5.11
15.32	-1.21	-4.655
15.33	-1.21	-4.655
15.34	-2.365	-4.98
15.35	-1.765	-4.965
15.36	-1.935	-4.72
15.37	-1.935	-4.72
15.38	-1.77	-4.515
15.39	-1.985	-4.405
15.4	-1.945	-4.365
15.41	-1.6	-4.33
15.42	-1.705	-4.285
15.43	-1.855	-4.315
15.44	-1.855	-4.315
15.45	-1.32	-4.185

15.46	-1.705	-3.985
15.47	-1.715	-4.085
15.48	-1.715	-4.085
15.49	-2.43	-4.225
15.5	-0.87	-3.84
15.51	-1.605	-3.87
15.52	-1.435	-4.01
15.53	-2.17	-3.925
15.54	-2.17	-3.925
15.55	-1.39	-3.775
15.56	-1.45	-3.79
15.57	-1.89	-3.695
15.58	-0.875	-3.265
15.59	-1.805	-3.5
15.6	-1.02	-3.41
15.61	-1.945	-3.585
15.62	-1.945	-3.585
15.63	-1.135	-3.105
15.64	-1.345	-3.11
15.65	-1.54	-3.405
15.66	-1.225	-2.875
15.67	-1.31	-3.1
15.68	-1.31	-3.1
15.69	-1.42	-3.11
15.7	-1.3	-2.715
15.71	-1.43	-2.595
15.72	-1.43	-2.595
15.73	-0.805	-2.535
15.74	-1.26	-2.49
15.75	-1.415	-2.835
15.76	-1.415	-2.835
15.77	-0.31	-2.07
15.78	-1.89	-2.73
15.79	-0.95	-2.355
15.8	-0.81	-2.075
15.81	-1	-2.23
15.82	-0.76	-1.935
15.83	-1.265	-2.245
15.84	-1.265	-2.245
15.85	-0.355	-1.695
15.86	-0.76	-1.655
15.87	-0.955	-1.895
15.88	-1.305	-1.885
15.89	-0.72	-1.675
15.9	-0.44	-1.545
15.91	-0.78	-1.56
15.92	-0.75	-1.315
15.93	-0.4	-1.045

15.94	-0.4	-1.045
15.95	-0.735	-1.33
15.96	-0.2	-0.775
15.97	-1.535	-1.58
15.98	-1.535	-1.58
15.99	-0.3	-0.73
16	-0.95	-0.92
16.01	-0.45	-0.93
16.02	-0.5	-0.84
16.03	-0.38	-0.935
16.04	-0.38	-0.935
16.05	0.15	-0.38
16.06	0.15	-0.38
16.07	-0.465	-0.325
16.08	-0.56	-0.54
16.09	0.07	-0.405
16.1	-0.035	-0.13
16.11	-0.74	-0.455
16.12	0.31	0.11
16.13	0.31	0.11
16.14	0.785	0.42
16.15	-0.935	-0.18
16.16	-0.225	0.03
16.17	0.5	0.37
16.18	0	0.31
16.19	-0.44	0.18
16.2	0.39	0.35
16.21	0.045	0.49
16.22	0.53	0.77
16.23	-0.25	0.545
16.24	-0.255	0.565
16.25	0.535	0.685
16.26	0.535	0.685
16.27	0.38	0.96
16.28	0.765	1.035
16.29	0.035	0.925
16.3	0.37	0.865
16.31	0.55	1.205
16.32	0.55	1.205
16.33	0.225	1.175
16.34	0.49	1.38
16.35	0.49	1.38
16.36	0.31	1.36
16.37	0.26	1.335
16.38	1.3	1.92
16.39	0.115	1.665
16.4	-0.125	1.135
16.41	1.23	1.89

16.42	-0.095	1.535
16.43	-0.095	1.535
16.44	1.535	2.105
16.45	0.245	1.715
16.46	0.245	1.715
16.47	0.165	1.785
16.48	1.29	2.365
16.49	1.29	2.365
16.5	-0.315	1.87
16.51	1.29	2.355
16.52	1.295	2.7
16.53	0.27	2.23
16.54	0.965	2.595
16.55	0.56	2.365
16.56	1.415	2.915
16.57	0.95	2.59
16.58	0.95	2.59
16.59	1.19	2.995
16.6	1.335	3.42
16.61	-0.06	2.465
16.62	1.775	2.88
16.63	1.775	2.88
16.64	1.105	3.175
16.65	0.76	3.025
16.66	1.405	3.19
16.67	1.05	3.19
16.68	0.98	3.2
16.69	0.98	3.2
16.7	1.325	3.57
16.71	1.555	3.735
16.72	0.53	3.1
16.73	1.92	3.745
16.74	1.92	3.745
16.75	1.34	3.61
16.76	1.615	3.86
16.77	1.39	3.88
16.78	1.04	3.795
16.79	1.04	3.795
16.8	1.955	4.085
16.81	1.43	3.965
16.82	1.805	4.26
16.83	1.06	3.81
16.84	2.155	4.36
16.85	1.16	4.235
16.86	1.165	3.81
16.87	1.165	3.81
16.88	1.955	4.21
16.89	1.64	4.62

16.9	1.345	4.235
16.91	1.19	4.185
16.92	2.255	4.64
16.93	2.255	4.64
16.94	1.815	4.67
16.95	1.725	4.575
16.96	1.485	4.59
16.97	2.51	5.12
16.98	1.055	4.58
16.99	1.055	4.58
17	1.87	4.925
17.01	1.815	4.895
17.02	1.855	4.95
17.03	1.79	5.155
17.04	1.79	5.155
17.05	1.865	5.01
17.06	1.98	5.095
17.07	2.08	5.185
17.08	1.76	5.105
17.09	2.205	5.41
17.1	2.18	5.345
17.11	2.175	5.58
17.12	1.42	5.195
17.13	1.42	5.195
17.14	2.055	5.685
17.15	1.725	5.19
17.16	2.745	5.715
17.17	1.71	5.465
17.18	2.365	5.635
17.19	2.425	5.825
17.2	1.54	5.42
17.21	2.01	5.395
17.22	2.865	5.9
17.23	1.525	5.6
17.24	2.415	5.795
17.25	2.14	5.595
17.26	2.14	5.595
17.27	3.21	5.95
17.28	2.325	5.85
17.29	1.38	5.53
17.3	1.38	5.53
17.31	2.73	6.07
17.32	1.94	5.72
17.33	2.62	5.905
17.34	2.62	5.905
17.35	2.145	5.905
17.36	2.57	5.865
17.37	2.175	5.72

17.38	1.745	5.775
17.39	2.725	5.885
17.4	1.91	5.7
17.41	2.12	5.775
17.42	2.915	5.965
17.43	1.57	5.595
17.44	1.915	5.56
17.45	3.575	6.24
17.46	3.575	6.24
17.47	1.39	5.6
17.48	2.93	5.88
17.49	1.815	5.735
17.5	2.475	5.735
17.51	1.635	5.325
17.52	1.635	5.325
17.53	1.58	5.63
17.54	2.92	5.765
17.55	1.35	5.215
17.56	1.35	5.215
17.57	1.9	5.415
17.58	2.47	5.705
17.59	2.23	5.705
17.6	1.74	5.465
17.61	2.33	5.43
17.62	2.33	5.43
17.63	2.17	5.485
17.64	2.03	5.54
17.65	1.88	5.295
17.66	2.35	5.495
17.67	2.015	5.355
17.68	2.1	5.345
17.69	2.1	5.345
17.7	2.215	5.15
17.71	2.34	5.33
17.72	1.94	5.175
17.73	1.94	5.175
17.74	2.66	5.445
17.75	1.69	5.205
17.76	2.145	5.045
17.77	1.51	4.62
17.78	2.235	4.825
17.79	2.62	5.405
17.8	1.205	4.56
17.81	2.23	4.735
17.82	2.225	4.895
17.83	1.535	4.86
17.84	2.01	4.805
17.85	1.69	4.53

17.86	2.155	4.7
17.87	2.1	4.815
17.88	1.32	4.25
17.89	1.32	4.25
17.9	1.695	4.59
17.91	2.045	4.725
17.92	1.45	4.455
17.93	1.765	4.455
17.94	1.78	4.57
17.95	1.91	4.28
17.96	1.91	4.28
17.97	2.005	4.3
17.98	1.63	4.07
17.99	2.095	4.305
18	1.315	4.04
18.01	1.925	4.205
18.02	1.055	3.745
18.03	2.055	3.96
18.04	2.055	3.96
18.05	0.99	3.535
18.06	2.15	3.955
18.07	1.715	3.86
18.08	0.855	3.095
18.09	1.86	3.73
18.1	1.24	3.355
18.11	1.405	3.335
18.12	1.64	3.48
18.13	1.64	3.48
18.14	2.11	3.37
18.15	0.71	2.965
18.16	1.65	3.095
18.17	1.65	3.095
18.18	0.545	2.81
18.19	1.025	2.81
18.2	1.56	3.025
18.21	1.24	2.93
18.22	0.635	2.49
18.23	1.225	2.44
18.24	1.225	2.44
18.25	1.13	2.93
18.26	0.71	2.175
18.27	0.93	2.145
18.28	1.25	2.485
18.29	1.25	2.485
18.3	0.535	2.07
18.31	1.695	2.515
18.32	0.1	1.725
18.33	0.1	1.725

18.34	0.55	1.685
18.35	1.54	2.03
18.36	0.46	1.67
18.37	0.46	1.67
18.38	1.09	1.86
18.39	0.76	1.395
18.4	0.905	1.54
18.41	0.19	1.22
18.42	0.855	1.445
18.43	0.855	1.445
18.44	0.36	0.885
18.45	0.62	1.23
18.46	0.445	0.965
18.47	0.445	0.965
18.48	1.185	1.24
18.49	-0.31	0.585
18.5	1.165	1.175
18.51	1.165	1.175
18.52	0.64	0.63
18.53	0.465	0.48
18.54	-0.165	0.195
18.55	0.855	0.855
18.56	-0.075	0.415
18.57	0.375	0.485
18.58	0.375	0.485
18.59	-0.215	0.24
18.6	0.565	0.35
18.61	0.195	0.18
18.62	-0.425	-0.095
18.63	0.495	0.33
18.64	-0.09	-0.08
18.65	0.3	0.18
18.66	0.3	0.18
18.67	-0.745	-0.53
18.68	0.16	-0.14
18.69	0.16	-0.14
18.7	-0.01	-0.31
18.71	-0.465	-0.625
18.72	0.04	-0.39
18.73	-0.125	-0.575
18.74	0.065	-0.495
18.75	-0.01	-0.42
18.76	-0.5	-0.945
18.77	0.105	-0.565
18.78	-0.63	-1.055
18.79	-0.63	-1.055
18.8	-1.205	-1.075
18.81	-0.16	-1.02

18.82	-0.08	-0.91
18.83	-0.57	-1.22
18.84	-0.02	-0.98
18.85	-0.275	-1.07
18.86	-0.655	-1.265
18.87	-0.255	-1.375
18.88	-0.255	-1.375
18.89	-0.2	-1.315
18.9	-0.385	-1.34
18.91	-0.68	-1.51
18.92	-0.61	-1.645
18.93	-0.61	-1.645
18.94	-0.6	-1.985
18.95	0.335	-1.26
18.96	-1.495	-2.07
18.97	-0.465	-2.18
18.98	-0.93	-2.135
18.99	-0.755	-2.275
19	-0.83	-2.315
19.01	-0.83	-2.315
19.02	-1.65	-2.88
19.03	-1.65	-2.88
19.04	0.015	-2.045
19.05	-1.76	-2.895
19.06	-0.44	-2.555
19.07	-1.32	-2.75
19.08	-0.95	-2.915
19.09	-0.98	-2.66
19.1	-1.24	-3.245
19.11	-0.32	-2.68
19.12	-1.455	-3.03
19.13	-1.455	-3.03
19.14	-0.75	-3.03
19.15	-1.145	-3.24
19.16	-1.65	-3.675
19.17	-0.795	-3.2
19.18	-1.345	-3.36
19.19	-1.345	-3.36
19.2	-1.18	-3.51
19.21	-1.18	-3.51
19.22	-0.72	-3.525
19.23	-1.34	-3.595
19.24	-1.435	-3.74
19.25	-1.545	-3.915
19.26	-1.545	-3.915
19.27	-1.895	-4.03
19.28	-1.135	-3.87
19.29	-1.565	-4.365

19.3	-1.03	-3.59
19.31	-1.03	-3.59
19.32	-0.03	-3.67
19.33	-2.11	-4.12
19.34	-2.11	-4.12
19.35	-1.645	-4.52
19.36	-0.83	-4.12
19.37	-2.065	-4.46
19.38	-1.57	-4.49
19.39	-1.85	-4.54
19.4	-1.85	-4.54
19.41	-1.79	-4.745
19.42	-1.77	-4.725
19.43	-1.605	-4.54
19.44	-2.155	-4.975
19.45	-1.47	-4.695
19.46	-1.71	-4.77
19.47	-1.71	-4.77
19.48	-1.93	-4.835
19.49	-1.875	-5.05
19.5	-1.875	-5.18
19.51	-1.875	-5.18
19.52	-1.98	-5.03
19.53	-2.4	-5.325
19.54	-1.53	-5.25
19.55	-1.53	-5.25
19.56	-2.96	-5.64
19.57	-1.165	-4.88
19.58	-1.85	-5.27
19.59	-2.3	-5.385
19.6	-2.62	-5.595
19.61	-1.22	-5.185
19.62	-1.965	-5.295
19.63	-2.745	-5.69
19.64	-2.745	-5.69
19.65	-2.02	-5.305
19.66	-2.76	-5.77
19.67	-1.125	-5.16
19.68	-1.125	-5.16
19.69	-2.65	-5.795
19.7	-1.72	-5.52
19.71	-2.07	-5.585
19.72	-2.05	-5.19
19.73	-2.68	-5.78
19.74	-1.445	-5.39
19.75	-2.365	-5.465
19.76	-2.455	-5.63
19.77	-1.32	-5.21

19.78	-3.27	-5.955
19.79	-1.97	-5.735
19.8	-1.97	-5.735
19.81	-2.65	-5.685
19.82	-2.02	-5.7
19.83	-2.135	-5.56
19.84	-2.135	-5.56
19.85	-1.905	-5.725
19.86	-2.195	-5.81
19.87	-2.345	-5.815
19.88	-2.375	-5.785
19.89	-1.685	-5.55
19.9	-1.685	-5.55
19.91	-2.81	-6.12
19.92	-1.62	-5.475
19.93	-2.33	-5.81
19.94	-2.05	-5.765
19.95	-2.345	-5.87
19.96	-2.39	-5.87
19.97	-2.39	-5.87
19.98	-1.745	-5.82
19.99	-2.26	-5.71
20	-2.585	-6.03
20.01	-1.97	-5.75
20.02	-2.285	-5.88
20.03	-2.315	-5.93
20.04	-2.025	-5.66
20.05	-2.78	-6.335
20.06	-1.83	-5.49
20.07	-2.95	-6.1
20.08	-1.14	-5.475
20.09	-2.865	-5.81
20.1	-2.865	-5.81
20.11	-2.195	-5.67
20.12	-2.235	-5.67
20.13	-2.215	-5.655
20.14	-2.37	-5.645
20.15	-2.055	-5.535
20.16	-2.055	-5.535
20.17	-2.425	-5.51
20.18	-2.345	-5.675
20.19	-2.135	-5.435
20.2	-2.04	-5.59
20.21	-2.02	-5.445
20.22	-2.02	-5.445
20.23	-2.48	-5.54
20.24	-2.48	-5.54
20.25	-2.425	-5.5

20.26	-1.615	-4.975
20.27	-2.885	-5.67
20.28	-1.535	-4.99
20.29	-1.895	-4.935
20.3	-2.3	-5.27
20.31	-2.115	-4.98
20.32	-2.475	-5.46
20.33	-1.355	-4.655
20.34	-2.38	-4.91
20.35	-2.38	-4.91
20.36	-2.02	-4.595
20.37	-2.38	-4.88
20.38	-1.415	-4.515
20.39	-2.445	-4.91
20.4	-2.445	-4.91
20.41	-1.405	-4.375
20.42	-2.25	-4.875
20.43	-1.01	-4.205
20.44	-1.01	-4.205
20.45	-1.835	-4.245
20.46	-2.04	-4.485
20.47	-1.64	-4.315
20.48	-1.64	-4.315
20.49	-1.895	-4.225
20.5	-1.57	-4.255
20.51	-2.005	-4.185
20.52	-2.005	-4.185
20.53	-2.08	-4.3
20.54	-1.14	-3.73
20.55	-2.165	-4.105
20.56	-2.165	-4.105
20.57	-1.525	-3.565
20.58	-1.525	-3.565
20.59	-1.255	-3.735
20.6	-1.81	-3.605
20.61	-1.445	-3.585
20.62	-1.445	-3.585
20.63	-1.795	-3.39
20.64	-1.61	-3.41
20.65	-1.42	-3.43
20.66	-1.175	-3.295
20.67	-1.19	-2.955
20.68	-1.19	-2.955
20.69	-1.055	-3.065
20.7	-1.535	-3.005
20.71	-0.58	-2.73
20.72	-1.555	-2.775
20.73	-0.935	-2.655

20.74	-1.49	-2.645
20.75	-1.425	-3.105
20.76	-1.425	-3.105
20.77	-0.915	-2.255
20.78	-1.3	-2.375
20.79	-0.8	-2.15
20.8	-1.42	-2.435
20.81	-0.92	-2.375
20.82	-0.36	-1.8
20.83	-1.315	-2.215
20.84	-1.485	-2.405
20.85	0.195	-1.555
20.86	-1.65	-2.26
20.87	-1.65	-2.26
20.88	-1.17	-1.835
20.89	-0.41	-1.7
20.9	-1.005	-1.735
20.91	-0.77	-1.66
20.92	-0.5	-1.585
20.93	-0.5	-1.585
20.94	-1.04	-1.56
20.95	0.345	-0.945
20.96	-1.485	-1.465
20.97	-1.485	-1.465
20.98	-0.105	-0.895
20.99	-0.67	-1.15
21	-0.85	-1.095
21.01	-0.85	-1.095
21.02	0.085	-0.62
21.03	-1.1	-1.035
21.04	0.13	-0.405
21.05	-0.785	-0.72
21.06	-0.785	-0.72
21.07	-0.48	-0.655
21.08	-0.48	-0.655
21.09	-0.65	-0.405
21.1	-0.01	-0.245
21.11	-0.15	-0.195
21.12	-0.15	0.1
21.13	0.085	0.045
21.14	-0.7	-0.405
21.15	0.61	0.48
21.16	-0.265	0.105
21.17	0.175	0.4
21.18	-0.24	0.255
21.19	-0.24	0.255
21.2	0.225	0.54
21.21	-0.46	0.195

21.22	0.035	0.49
21.23	0.545	0.77
21.24	0.545	0.77
21.25	-0.235	0.57
21.26	0.415	0.93
21.27	0.04	0.875
21.28	0.44	1
21.29	0.44	1
21.3	0.82	0.965
21.31	0.61	1.41
21.32	0.165	1.215
21.33	0.165	1.215
21.34	0.73	1.315
21.35	0.42	1.47
21.36	0.325	1.27
21.37	0.85	1.625
21.38	-0.015	1.355
21.39	0.87	1.765
21.4	0.87	1.765
21.41	0.8	1.86
21.42	0.735	1.835
21.43	0.43	1.88
21.44	0.43	1.88
21.45	0.67	1.995
21.46	0.71	2.115
21.47	1.28	2.49
21.48	1.28	2.49
21.49	0.03	2.06
21.5	1.08	2.505
21.51	0.485	2.33
21.52	0.975	2.375
21.53	1.345	2.78
21.54	0.355	2.49
21.55	0.355	2.49
21.56	0.705	2.445
21.57	1.32	2.95
21.58	1.22	2.995
21.59	1.22	2.995
21.6	1.475	3.135
21.61	0.43	2.905
21.62	1.545	2.995
21.63	1.195	3.29
21.64	1.19	3.29
21.65	1.19	3.29
21.66	0.955	3.275
21.67	1.275	3.36
21.68	1.7	3.685
21.69	0.775	3.355

21.7	1.61	3.61
21.71	1.61	3.61
21.72	1.32	3.675
21.73	1.49	3.73
21.74	1.245	3.91
21.75	1.1	3.62
21.76	1.695	3.885
21.77	1.275	3.905
21.78	1.275	3.905
21.79	1.265	4.075
21.8	1.06	4.075
21.81	1.745	3.99
21.82	1.72	4.185
21.83	1.72	4.185
21.84	1.38	4.16
21.85	1.855	4.29
21.86	1.86	4.535
21.87	1.5	4.525
21.88	1.625	4.435
21.89	1.485	4.33
21.9	1.975	4.785
21.91	1.72	4.685
21.92	1.015	4.31
21.93	1.015	4.31
21.94	1.6	4.55
21.95	1.905	4.735
21.96	2.13	5.03
21.97	2.13	5.03
21.98	1.59	4.775
21.99	1.915	4.71
22	2.03	5.15
22.01	2.03	5.15
22.02	1.73	4.85
22.03	2.65	5.385
22.04	1.325	5.01
22.05	1.92	5.035
22.06	1.83	5.08
22.07	2.05	5.16
22.08	2.05	5.16
22.09	1.89	5.25
22.1	1.38	4.89
22.11	3.02	5.735
22.12	3.02	5.735
22.13	2.23	5.4
22.14	1.55	5.155
22.15	2.32	5.365
22.16	2.05	5.395
22.17	1.915	5.22

22.18	1.915	5.22
22.19	1.215	5.025
22.2	2.865	5.535
22.21	1.96	5.4
22.22	2.235	5.575
22.23	1.885	5.555
22.24	2.065	5.365
22.25	1.885	5.505
22.26	2	5.25
22.27	2.74	5.86
22.28	1.265	5.19
22.29	1.265	5.19
22.3	2.215	5.385
22.31	2.72	6.075
22.32	1.77	5.615
22.33	1.42	5.08
22.34	2.675	5.595
22.35	2.675	5.595
22.36	2.3	5.55
22.37	1.63	5.355
22.38	2.93	5.895
22.39	1.66	5.365
22.4	1.625	5.32
22.41	3.04	5.74
22.42	1.55	5.465
22.43	1.55	5.465
22.44	2.44	5.685
22.45	1.195	5.27
22.46	2.345	5.495
22.47	2.74	5.61
22.48	2.075	5.585
22.49	1.725	5.39
22.5	1.725	5.39
22.51	1.49	5.445
22.52	2.49	5.53
22.53	2.015	5.38
22.54	2.015	5.38
22.55	2.36	5.7
22.56	2.74	5.735
22.57	1.785	5.325
22.58	1.785	5.325
22.59	1.765	5.36
22.6	2.495	5.64
22.61	2.355	5.705
22.62	2.355	5.705
22.63	2.415	5.585
22.64	2.1	5.465
22.65	2.465	5.59

22.66	2.05	5.505
22.67	1.615	5.01
22.68	2.975	5.69
22.69	2.975	5.69
22.7	1.635	5.16
22.71	2.41	5.375
22.72	1.49	5
22.73	2.61	5.34
22.74	2.655	5.72
22.75	1.185	4.905
22.76	1.185	4.905
22.77	2.32	5.215
22.78	1.665	5.195
22.79	2.17	4.935
22.8	2.17	4.935
22.81	1.685	4.79
22.82	2.33	5.055
22.83	2.195	4.8
22.84	2.195	4.8
22.85	2.15	4.78
22.86	1.82	4.695
22.87	2.305	4.865
22.88	1.48	4.47
22.89	1.935	4.555
22.9	1.71	4.7
22.91	2.235	4.685
22.92	1.37	4.245
22.93	2.08	4.375
22.94	1.515	4.185
22.95	1.515	4.185
22.96	0.965	4.19
22.97	1.97	4.035
22.98	1.61	3.94
22.99	1.675	4.125
23	1.88	3.985
23.01	1.82	3.93
23.02	1.82	3.93
23.03	1.81	3.995
23.04	1.505	3.725
23.05	1.505	3.725
23.06	1.55	3.605
23.07	1.59	3.67
23.08	1.395	3.65
23.09	1.395	3.65
23.1	1.11	3.07
23.11	1.94	3.625
23.12	1.44	3.525
23.13	1.055	2.92

23.14	1.65	3.465
23.15	1.39	3.175
23.16	1.39	3.175
23.17	1.3	2.875
23.18	1.84	3.255
23.19	0.955	2.89
23.2	1.14	2.76
23.21	1.585	2.715
23.22	0.71	2.6
23.23	0.71	2.6
23.24	0.31	2.24
23.25	1.575	2.455
23.26	1.05	2.48
23.27	1.05	2.48
23.28	0.365	2.155
23.29	1.615	2.475
23.3	0.515	2.23
23.31	0.515	2.23
23.32	1.66	2.425
23.33	0.03	1.875
23.34	0.73	1.745
23.35	1.44	2.28
23.36	0.575	1.76
23.37	0.575	1.76
23.38	0.895	1.715
23.39	1.075	1.895
23.4	0.54	1.57
23.41	0.49	1.485
23.42	0.815	1.41
23.43	0.815	1.41
23.44	0.635	1.41
23.45	0.59	1.3
23.46	0.67	1.17
23.47	0.67	1.17
23.48	0.14	1.1
23.49	0.67	1.26
23.5	0.67	1.26
23.51	0.175	0.695
23.52	0.03	0.82
23.53	1.01	0.955
23.54	1.01	0.955
23.55	0.08	0.64
23.56	0.54	0.685
23.57	0.38	0.565
23.58	0.38	0.565
23.59	0.155	0.59
23.6	-0.225	0.125
23.61	0.855	0.74

23.62	0.855	0.74
23.63	0.29	0.08
23.64	0.405	0.265
23.65	0.01	0.155
23.66	-0.15	-0.1
23.67	0.535	0.125
23.68	-0.22	-0.135
23.69	0.21	-0.02
23.7	0.21	-0.02
23.71	0.385	-0.18
23.72	0.56	0.03
23.73	-0.86	-0.6
23.74	-0.86	-0.6
23.75	-0.175	-0.76
23.76	0.185	-0.365
23.77	0.185	-0.365
23.78	-0.84	-1.065
23.79	-0.04	-0.815
23.8	-0.295	-0.88
23.81	0.26	-0.9
23.82	0.26	-0.9
23.83	0.095	-0.94
23.84	-0.255	-1.3
23.85	-0.32	-1.17
23.86	-0.775	-1.34
23.87	-0.125	-1.47
23.88	-0.125	-1.47
23.89	-0.25	-1.505
23.9	-0.465	-1.74
23.91	-0.465	-1.74
23.92	0.01	-1.65
23.93	-0.81	-1.96
23.94	-0.635	-1.905
23.95	-0.79	-2.18
23.96	-0.28	-1.9
23.97	-0.28	-1.9
23.98	-0.92	-2.41
23.99	-0.615	-2.25
24	-1.01	-2.45
24.01	-0.2	-2.145
24.02	-0.2	-2.145
24.03	-1.54	-2.92
24.04	-0.615	-2.535
24.05	-1.19	-2.815
24.06	-1.115	-2.895
24.07	-0.65	-2.805
24.08	-0.65	-2.805
24.09	-0.615	-2.605

24.1	-1.98	-3.485
24.11	-0.48	-3.055
24.12	-1.48	-3.395
24.13	-0.565	-2.97
24.14	-1.79	-3.545
24.15	-1.005	-3.33
24.16	-0.92	-3.26
24.17	-1.395	-3.46
24.18	-1.07	-3.45
24.19	-1.07	-3.45
24.2	-1.475	-3.77
24.21	-1.345	-3.705
24.22	-1.375	-3.89
24.23	-1.375	-3.89
24.24	-1.905	-3.895
24.25	-1.085	-3.79
24.26	-1.705	-4.115
24.27	-1.705	-4.115
24.28	-1.45	-3.755
24.29	-2.135	-4.51
24.3	-0.855	-3.785
24.31	-2.255	-4.355
24.32	-1.345	-4.3
24.33	-1.13	-3.93
24.34	-2.115	-4.395
24.35	-1.425	-4.18
24.36	-1.875	-4.535
24.37	-1.875	-4.535
24.38	-1.74	-4.585
24.39	-1.55	-4.515
24.4	-2.025	-4.78
24.41	-1.54	-4.375
24.42	-1.69	-4.47
24.43	-1.73	-4.775
24.44	-1.73	-4.775
24.45	-1.815	-4.68
24.46	-1.815	-4.68
24.47	-1.61	-4.845
24.48	-1.99	-5.03
24.49	-1.855	-4.935
24.5	-1.97	-5.035
24.51	-1.97	-5.035
24.52	-2.55	-5.37
24.53	-1.115	-4.755
24.54	-2.58	-5.49
24.55	-2.58	-5.49
24.56	-2.17	-5.36
24.57	-1.35	-5.055

24.58	-2.505	-5.495
24.59	-2.505	-5.495
24.6	-2.245	-5.48
24.61	-1.57	-5.27
24.62	-2.365	-5.355
24.63	-1.845	-5.54
24.64	-2	-5.29
24.65	-2.02	-5.455
24.66	-2.16	-5.62
24.67	-1.53	-5.21
24.68	-1.53	-5.21
24.69	-1.535	-5.235
24.7	-2.415	-5.73
24.71	-1.975	-5.51
24.72	-2.385	-5.69
24.73	-2.005	-5.62
24.74	-1.62	-5.44
24.75	-2.86	-5.91
24.76	-1.745	-5.45
24.77	-2.255	-5.63
24.78	-2.255	-5.63
24.79	-2.285	-5.675
24.8	-1.82	-5.38
24.81	-2.525	-5.865
24.82	-1.905	-5.4
24.83	-2.415	-5.74
24.84	-1.975	-5.48
24.85	-2.72	-5.915
24.86	-2.72	-5.915
24.87	-1.735	-5.415
24.88	-2.625	-5.615
24.89	-1.93	-5.6
24.9	-2.61	-6
24.91	-1.975	-5.43
24.92	-1.795	-5.515
24.93	-1.795	-5.515
24.94	-1.87	-5.63
24.95	-2.485	-5.88
24.96	-1.38	-5.25
24.97	-2.645	-5.795
24.98	-2.05	-5.52
24.99	-2.735	-5.95
25	-1.395	-5.355
25.01	-3.185	-6.155
25.02	-1.085	-5.28
25.03	-2.97	-5.78
25.04	-2.97	-5.78
25.05	-1.93	-5.625

25.06	-1.955	-5.415
25.07	-2.59	-5.865
25.08	-1.695	-5.43
25.09	-2.115	-5.485
25.1	-2.115	-5.485
25.11	-1.76	-5.375
25.12	-2.28	-5.655
25.13	-2.04	-5.41
25.14	-2.075	-5.29
25.15	-2.125	-5.37
25.16	-2.175	-5.435
25.17	-2.355	-5.46
25.18	-2.355	-5.46
25.19	-1.87	-5.44
25.2	-2.315	-5.465
25.21	-2.215	-5.455
25.22	-1.635	-5.165
25.23	-2.575	-5.415
25.24	-1.445	-5.12
25.25	-2.67	-5.495
25.26	-1.675	-4.85
25.27	-2.3	-5.36
25.28	-1.805	-5.145
25.29	-1.67	-4.77
25.3	-1.67	-4.77
25.31	-1.755	-4.955
25.32	-2.425	-5.21
25.33	-1.53	-4.69
25.34	-2.11	-4.84
25.35	-2.345	-5.1
25.36	-1.295	-4.59
25.37	-2.285	-4.725
25.38	-2.285	-4.725
25.39	-1.795	-4.47
25.4	-1.82	-4.545
25.41	-1.94	-4.56
25.42	-1.94	-4.56
25.43	-1.175	-4.245
25.44	-1.175	-4.245
25.45	-1.325	-4.095
25.46	-2.075	-4.45
25.47	-1.62	-4.215
25.48	-1.645	-3.935
25.49	-1.645	-3.935
25.5	-1.75	-4.09
25.51	-1.655	-4.03
25.52	-1.45	-3.925
25.53	-1.71	-3.915

25.54	-1.71	-3.915
25.55	-1.63	-3.805
25.56	-1.81	-3.78
25.57	-1.38	-3.675
25.58	-1.38	-3.675
25.59	-1.37	-3.535
25.6	-1.46	-3.295
25.61	-1.815	-3.61
25.62	-1.815	-3.61
25.63	-1.815	-3.41
25.64	-1.475	-3.395
25.65	-1.155	-3.085
25.66	-1.45	-3.09
25.67	-1.315	-3.205
25.68	-1.315	-3.205
25.69	-1.735	-3.31
25.7	-0.395	-2.515
25.71	-1.795	-2.975
25.72	-0.93	-2.775
25.73	-1.525	-2.685
25.74	-1.525	-2.685
25.75	-0.81	-2.245
25.76	-1.54	-2.67
25.77	-0.835	-2.38
25.78	-1.54	-2.54
25.79	-1.54	-2.54
25.8	-1.695	-2.475
25.81	-0.835	-2.1
25.82	-0.725	-1.995
25.83	-1.235	-2.155
25.84	-0.375	-1.6
25.85	-1.505	-2.07
25.86	-0.645	-1.905
25.87	-0.435	-1.44
25.88	-0.435	-1.44
25.89	-1	-1.81
25.9	-0.515	-1.64
25.91	-0.29	-1.145
25.92	-1.37	-1.56
25.93	-1.37	-1.56
25.94	-0.885	-1.405
25.95	-0.385	-0.96
25.96	-0.685	-1.25
25.97	-0.685	-1.25
25.98	-0.08	-0.87
25.99	-0.44	-0.715
26	-0.615	-0.91
26.01	-0.615	-0.91

26.02	-0.555	-0.83
26.03	-0.22	-0.65
26.04	-0.195	-0.425
26.05	-0.33	-0.475
26.06	-0.445	-0.56
26.07	-0.28	-0.435
26.08	-0.19	-0.54
26.09	-0.165	-0.35
26.1	-0.525	-0.265
26.11	0.36	-0.03
26.12	-0.235	-0.065
26.13	-0.695	-0.345
26.14	0.705	0.52
26.15	0.705	0.52
26.16	0.065	0.255
26.17	-0.46	0.07
26.18	0.2	0.295
26.19	0.2	0.295
26.2	0.5	0.72
26.21	-0.305	0.555
26.22	-0.105	0.28
26.23	0.525	0.82
26.24	0	0.525
26.25	0.36	0.845
26.26	-0.335	0.545
26.27	0.945	1.045
26.28	-0.16	0.71
26.29	0.15	0.935
26.3	0.15	0.935
26.31	0.78	1.315
26.32	-0.435	1.095
26.33	0.83	1.27
26.34	0.83	1.27
26.35	0.49	1.255
26.36	0.45	1.3
26.37	0.98	1.75
26.38	-0.365	1.35
26.39	-0.365	1.35
26.4	0.68	1.61
26.41	0.68	1.61
26.42	0.74	2.04
26.43	0.41	1.6
26.44	0.41	1.6
26.45	0.645	2.23
26.46	0.98	2.1
26.47	-0.06	1.695
26.48	1.455	2.635
26.49	0.78	2.34

26.5	0.475	2.205
26.51	1.01	2.65
26.52	0.9	2.345
26.53	0.595	2.42
26.54	0.99	2.51
26.55	0.99	2.51
26.56	0.575	2.61
26.57	0.945	2.725
26.58	1.505	2.975
26.59	1.505	2.975
26.6	1.465	3.02
26.61	1.035	3.26
26.62	0.565	2.82
26.63	1.665	3.305
26.64	1.665	3.305
26.65	1.105	3.245
26.66	1.105	3.245
26.67	0.96	3.31
26.68	0.705	2.99
26.69	2.185	3.675
26.7	2.185	3.675
26.71	1.02	3.37
26.72	2.105	3.925
26.73	0.705	3.36
26.74	2.29	4.28
26.75	0.43	3.49
26.76	1.7	3.975
26.77	1.7	3.975
26.78	1.505	3.91
26.79	1.265	3.82
26.8	1.39	3.775
26.81	1.39	3.775
26.82	1.88	4.335
26.83	0.91	4.1
26.84	2.245	4.345
26.85	1.24	4.27
26.86	1.33	4.2
26.87	2.21	4.66
26.88	1.03	4.265
26.89	2.165	4.65
26.9	1.255	4.35
26.91	1.805	4.615
26.92	1.56	4.64
26.93	1.63	4.67
26.94	1.85	4.65
26.95	1.85	4.65
26.96	2.15	5.01
26.97	1.65	4.785

26.98	1.76	4.85
26.99	1.76	4.85
27	1.64	5.035
27.01	1.045	4.6
27.02	2.135	4.955
27.03	2.17	5.145
27.04	1.665	5.015
27.05	1.665	5.015
27.06	2.065	5.06
27.07	2.01	5.38
27.08	1.62	5.11
27.09	2.38	5.275
27.1	2.38	5.275
27.11	1.755	5.205
27.12	1.915	5.08
27.13	2.65	5.585
27.14	1.465	5.27
27.15	2.08	4.975
27.16	2.545	5.785
27.17	1.38	5.19
27.18	1.38	5.19
27.19	2.015	5.305
27.2	2.4	5.55
27.21	2.165	5.475
27.22	1.59	5.405
27.23	2.78	5.99
27.24	2.78	5.99
27.25	2.275	5.37
27.26	2.74	5.905
27.27	1.53	5.28
27.28	2.565	5.69
27.29	2.565	5.69
27.3	3.08	5.82
27.31	1.585	5.5
27.32	2.455	5.715
27.33	2.455	5.715
27.34	2.095	5.38
27.35	2.89	5.93
27.36	1.75	5.5
27.37	2.155	5.81
27.38	2.05	5.515
27.39	1.92	5.29
27.4	3.005	6.105
27.41	1.945	5.655
27.42	1.82	5.615
27.43	1.82	5.615
27.44	2.835	5.875
27.45	1.74	5.5

27.46	2.47	5.84
27.47	2.31	5.755
27.48	1.81	5.62
27.49	1.81	5.62
27.5	2.18	5.615
27.51	2.1	5.585
27.52	2.095	5.57
27.53	2.6	5.875
27.54	2.6	5.875
27.55	2.375	5.66
27.56	1.97	5.64
27.57	2.165	5.44
27.58	2.745	6.01
27.59	1.375	5.005
27.6	2.325	5.46
27.61	2.325	5.46
27.62	1.54	5.405
27.63	2.015	5.39
27.64	2.6	5.55
27.65	2.6	5.55
27.66	1.79	5.23
27.67	1.555	5.315
27.68	1.555	5.315
27.69	1.595	5.25
27.7	2.305	5.375
27.71	2.19	5.185
27.72	2.16	5.345
27.73	2.16	5.345
27.74	1.345	4.825
27.75	2.57	5.285
27.76	2.085	5.33
27.77	1.9	5.09
27.78	1.775	5.035
27.79	2.6	5.38
27.8	1.49	4.66
27.81	1.49	4.66
27.82	2.575	5.43
27.83	1.92	5.06
27.84	1.415	4.715
27.85	2.045	4.725
27.86	1.95	4.57
27.87	1.95	4.57
27.88	1.495	4.57
27.89	2.455	5.005
27.9	1.295	4.495
27.91	2.04	4.505
27.92	1.935	4.665
27.93	1.62	4.285

27.94	1.985	4.595
27.95	1.56	4.27
27.96	1.56	4.27
27.97	1.775	4.425
27.98	1.62	4.18
27.99	1.765	4.365
28	1.465	4.165
28.01	1.69	4.065
28.02	1.605	3.85
28.03	2.115	4.185
28.04	1.03	3.675
28.05	1.03	3.675
28.06	1.785	3.94
28.07	1.755	3.705
28.08	1.165	3.7
28.09	1.315	3.425
28.1	1.315	3.425
28.11	1.355	3.5
28.12	1.355	3.5
28.13	0.84	3.205
28.14	1.465	3.24
28.15	1.445	3.205
28.16	1.835	3.25
28.17	0.475	2.89
28.18	1.785	3.11
28.19	1.155	2.805
28.2	1.095	2.675
28.21	1.75	3.095
28.22	0.58	2.6
28.23	0.58	2.6
28.24	1.195	2.595
28.25	1.3	2.725
28.26	0.975	2.56
28.27	0.665	2
28.28	1.55	2.445
28.29	1.11	2.38
28.3	0.425	1.86
28.31	1.255	2.205
28.32	1.255	2.205
28.33	1.12	2.17
28.34	0.415	1.73
28.35	1.19	1.85
28.36	0.54	1.785
28.37	0.71	1.65
28.38	0.71	1.65
28.39	0.235	1.225
28.4	1.08	1.83
28.41	0.62	1.41

28.42	0.805	1.425
28.43	0.455	1.295
28.44	0.315	1.07
28.45	1.12	1.435
28.46	1.12	1.435
28.47	0.535	1.09
28.48	0.23	0.88
28.49	0.795	1.12
28.5	0.115	0.62
28.51	0.8	0.92
28.52	0.8	0.92
28.53	0.455	0.65
28.54	0.22	0.495
28.55	0.365	0.545
28.56	0.395	0.51
28.57	0.23	0.465
28.58	0.23	0.465
28.59	-0.21	0.18
28.6	-0.21	0.18
28.61	0.26	0.34
28.62	0.025	0.185
28.63	0.2	0.185
28.64	0.04	-0.005
28.65	-0.045	-0.17
28.66	-0.045	-0.17
28.67	-0.18	-0.175
28.68	-0.235	-0.34
28.69	0.42	-0.02
28.7	0.42	-0.02
28.71	-0.785	-0.62
28.72	0.51	-0.24
28.73	0.07	-0.445
28.74	-0.515	-0.64
28.75	-0.25	-0.51
28.76	-0.1	-0.74
28.77	-0.085	-0.685
28.78	-0.085	-0.685
28.79	-0.3	-0.745
28.8	-0.665	-1.065
28.81	0.07	-0.995
28.82	0.23	-0.77
28.83	-0.98	-1.2
28.84	-0.565	-1.19
28.85	-0.565	-1.19
28.86	-0.33	-1.365
28.87	-1.055	-1.49
28.88	0.175	-1.3
28.89	-0.77	-1.64

28.9	-0.09	-1.525
28.91	-0.645	-1.545
28.92	-1.02	-1.85
28.93	-0.21	-1.765
28.94	-0.21	-1.765
28.95	-0.5	-1.825
28.96	-0.505	-1.8
28.97	-1.11	-2.175
28.98	-0.32	-2.045
28.99	-0.32	-2.045
29	-0.42	-2.25
29.01	-0.935	-2.335
29.02	-0.96	-2.4
29.03	-0.96	-2.4
29.04	-1.02	-2.735
29.05	-0.565	-2.515
29.06	-1.095	-2.825
29.07	-0.435	-2.55
29.08	-1.635	-3
29.09	-0.495	-2.84
29.1	-0.495	-2.84
29.11	-1.4	-3.115
29.12	-0.97	-2.935
29.13	-0.97	-2.935
29.14	-0.575	-3.085
29.15	-1.355	-3.13
29.16	-1.09	-3.3
29.17	-1.515	-3.63
29.18	-1.515	-3.63
29.19	-1.515	-3.52
29.2	-1.255	-3.585
29.21	-1.005	-3.515
29.22	-1.88	-3.945
29.23	-0.9	-3.72
29.24	-0.9	-3.72
29.25	-1.65	-4.14
29.26	-1.045	-3.76
29.27	-1.84	-4.26
29.28	-1.04	-3.82
29.29	-1.04	-3.82
29.3	-1.49	-4.255
29.31	-1.61	-4.2
29.32	-1.82	-4.535
29.33	-1.17	-4.295
29.34	-1.545	-4.32
29.35	-1.445	-4.215
29.36	-2.545	-4.85
29.37	-2.545	-4.85

29.38	-1.46	-4.1
29.39	-2.585	-4.915
29.4	-0.9	-4.2
29.41	-2.765	-4.975
29.42	-0.69	-4.365
29.43	-2.01	-4.74
29.44	-2.01	-4.74
29.45	-1.9	-4.94
29.46	-1.52	-4.8
29.47	-1.935	-4.895
29.48	-1.935	-4.895
29.49	-2.61	-5.33
29.5	-1.38	-4.94
29.51	-1.77	-5.015
29.52	-1.915	-5.055
29.53	-2.115	-5.215
29.54	-2.115	-5.215
29.55	-1.685	-5.125
29.56	-2.035	-5.21
29.57	-1.61	-4.91
29.58	-2.825	-5.42
29.59	-1.605	-5.14
29.6	-1.94	-5.595
29.61	-1.93	-5.165
29.62	-1.93	-5.165
29.63	-1.82	-5.445
29.64	-1.92	-5.275
29.65	-2.21	-5.39
29.66	-2.56	-5.695
29.67	-1.485	-5.275
29.68	-1.485	-5.275
29.69	-1.81	-5.525
29.7	-2.165	-5.695
29.71	-2.25	-5.535
29.72	-2.25	-5.535
29.73	-2.205	-5.545
29.74	-2.46	-5.89
29.75	-1.275	-5.28
29.76	-3.31	-6.01
29.77	-1.225	-5.475
29.78	-2.355	-5.65
29.79	-2.44	-5.855
29.8	-1.72	-5.375
29.81	-1.72	-5.375
29.82	-2	-5.885
29.83	-2	-5.885
29.84	-2.91	-6.12
29.85	-1.545	-5.435

29.86	-2.33	-5.73
29.87	-1.975	-5.595
29.88	-2.605	-5.875
29.89	-2.03	-5.63
29.9	-2.265	-5.535
29.91	-2.245	-5.92
29.92	-2.265	-5.785
29.93	-2.2	-5.59
29.94	-2.2	-5.59
29.95	-1.54	-5.555
29.96	-2.565	-5.63
29.97	-2.275	-5.785
29.98	-2.18	-5.57
29.99	-2.39	-5.775
30	-2.11	-5.79
30.01	-2.295	-5.76
30.02	-2.42	-5.66
30.03	-1.605	-5.375
30.04	-2.19	-5.47
30.05	-2.19	-5.47
30.06	-2.055	-5.705
30.07	-2.24	-5.765
30.08	-1.305	-5.24
30.09	-1.305	-5.24
30.1	-1.74	-5.52
30.11	-2.705	-5.78
30.12	-1.51	-5.345
30.13	-2.31	-5.55
30.14	-2.245	-5.58
30.15	-1.99	-5.37
30.16	-2.55	-5.855
30.17	-1.975	-5.655
30.18	-1.7	-5.205
30.19	-2.74	-5.71
30.2	-2.74	-5.71
30.21	-2.495	-5.745
30.22	-1.27	-5.34
30.23	-2.885	-5.545
30.24	-1.845	-5.325
30.25	-1.88	-5.24
30.26	-2.36	-5.185
30.27	-2.54	-5.68
30.28	-2.54	-5.68
30.29	-2.415	-5.34
30.3	-2.415	-5.34
30.31	-1.535	-4.945
30.32	-2.62	-5.095
30.33	-1.64	-4.88

30.34	-1.64	-4.88
30.35	-2.28	-5.12
30.36	-1.61	-4.92
30.37	-2.125	-4.785
30.38	-1.44	-4.36
30.39	-2.54	-5.005
30.4	-2.54	-5.005
30.41	-1.385	-4.54
30.42	-1.255	-4.285
30.43	-2.4	-4.83
30.44	-1.645	-4.37
30.45	-1.615	-4.305
30.46	-1.615	-4.305
30.47	-2.35	-4.38
30.48	-1.24	-3.935
30.49	-1.96	-4.01
30.5	-2.02	-4.26
30.51	-2.02	-4.26
30.52	-1.5	-3.635
30.53	-2.01	-4.11
30.54	-1.64	-4.06
30.55	-1.11	-3.345
30.56	-2.1	-3.875
30.57	-1.11	-3.685
30.58	-1.72	-3.725
30.59	-1.72	-3.725
30.6	-1	-3.3
30.61	-2.135	-3.64
30.62	-1.905	-3.625
30.63	-1.12	-3.3
30.64	-1.695	-3.36
30.65	-1.695	-3.36
30.66	-1.435	-3.39
30.67	-1.215	-3.035
30.68	-1.62	-3.165
30.69	-0.915	-2.83
30.7	-1.295	-2.9
30.71	-1.295	-2.9
30.72	-1.645	-2.745
30.73	-1.305	-2.82
30.74	-1.305	-2.82
30.75	-0.805	-2.61
30.76	-1.545	-2.76
30.77	-0.765	-2.355
30.78	-0.93	-2.26
30.79	-1.155	-2.34
30.8	-0.945	-2.285
30.81	-1.47	-2.375

30.82	-1.47	-2.375
30.83	-0.54	-1.615
30.84	-1.21	-2.265
30.85	-0.57	-1.71
30.86	-1.245	-1.845
30.87	-1	-1.935
30.88	-1	-1.935
30.89	-0.995	-1.5
30.9	-1.47	-1.925
30.91	0.005	-1.16
30.92	-0.675	-1.36
30.93	-0.675	-1.36
30.94	-0.45	-1.195
30.95	-0.935	-1.225
30.96	-0.31	-1.015
30.97	-0.585	-1.06
30.98	-0.625	-1.085
30.99	-0.625	-1.085
31	-0.54	-0.9
31.01	-0.405	-0.71
31.02	0.115	-0.54
31.03	-1.185	-0.96
31.04	-1.185	-0.96
31.05	-0.625	-0.535
31.06	-0.065	-0.415
31.07	-0.89	-0.49
31.08	0.04	-0.33
31.09	0.075	0.025
31.1	0.075	0.025
31.11	0.435	0.15
31.12	-0.93	-0.27
31.13	0.41	0.255
31.14	-0.47	-0.22
31.15	-0.47	-0.22
31.16	-1.275	-0.34
31.17	-1.275	-0.34
31.18	-0.37	0.25
31.19	0.2	0.38
31.2	0.03	0.315
31.21	-0.08	0.375
31.22	0.18	0.455
31.23	-0.39	0.21
31.24	0.275	0.255
31.25	-0.435	0.015
31.26	-0.435	0.015
31.27	0.175	0.425
31.28	0.415	0.475
31.29	-0.58	0.155

31.3	0.525	0.325
31.31	-0.325	0.13
31.32	-0.11	0.015
31.33	1.1	0.695
31.34	1.1	0.695
31.35	0.935	0.485
31.36	-0.035	0.185
31.37	0.085	0.18
31.38	0.13	0.18
31.39	0.08	0.105
31.4	0.08	0.105
31.41	0.51	0.405
31.42	-0.745	-0.28
31.43	0.57	0.33
31.44	-0.065	0.06
31.45	0.49	0.29
31.46	-0.275	0.02
31.47	0.575	0.395
31.48	0.575	0.395
31.49	0.265	0.165
31.5	-0.03	0.135
31.51	0.2	0.18
31.52	-0.185	0.025
31.53	-0.185	0.025
31.54	0.18	0.195
31.55	0.18	0.195
31.56	0.27	0.245
31.57	-0.35	0.2
31.58	0.19	0.19
31.59	-0.045	0.06
31.6	-0.045	0.06
31.61	0.09	0.46
31.62	-0.42	-0.125
31.63	-0.42	-0.125
31.64	-0.005	0.145
31.65	-0.085	0.22
31.66	0.525	0.615
31.67	0.525	0.615
31.68	-0.54	-0.075
31.69	0.195	0.165
31.7	0.23	0.205
31.71	0.535	0.15
31.72	0.18	0.305
31.73	-0.46	-0.115
31.74	0.675	0.23
31.75	-0.43	-0.07
31.76	0.575	0.4

Table S6. 8-DOF Data of Central Area Steering Condition

Time	ay	yawrate
0	-0.41532	-0.2
0.025	-0.11596	-0.39274
0.05	-0.02217	-0.38501
0.075	-0.01284	-0.30442
0.1	-0.03109	-0.2017
0.125	-0.04526	-0.10341
0.15	-0.04584	-0.01801
0.175	-0.03541	0.050086
0.2	-0.02243	0.096748
0.225	-0.0148	0.126085
0.25	-0.01409	0.141884
0.275	-0.01486	0.14875
0.3	-0.01421	0.148997
0.325	-0.00901	0.142805
0.35	-0.00088	0.132853
0.375	0.007743	0.121401
0.4	0.014039	0.107884
0.425	0.015824	0.092026
0.45	0.012947	0.07545
0.475	0.007302	0.059987
0.5	0.002083	0.04636
0.525	-0.001	0.034823
0.55	-0.00207	0.026559
0.575	-0.00285	0.018296
0.6	-0.00465	0.009537
0.625	-0.008	0.001236
0.65	-0.01215	-0.00472
0.675	-0.01601	-0.01229
0.7	-0.02004	-0.01914
0.725	-0.02372	-0.02538
0.75	-0.02723	-0.03043
0.775	-0.02943	-0.03558
0.8	-0.03161	-0.04014
0.825	-0.0326	-0.04418
0.85	-0.03476	-0.04796
0.875	-0.03695	-0.05203
0.9	-0.03915	-0.05663
0.925	-0.04159	-0.06145
0.95	-0.04401	-0.06682
0.975	-0.0456	-0.07226

1	-0.04746	-0.07784
1.025	-0.04923	-0.08293
1.05	-0.05078	-0.08822
1.075	-0.05283	-0.09334
1.1	-0.04947	-0.09686
1.125	-0.02886	-0.08079
1.15	-0.00225	-0.02963
1.175	0.026192	0.059365
1.2	0.05684	0.182883
1.225	0.08984	0.333939
1.25	0.128982	0.503107
1.275	0.176243	0.68366
1.3	0.229864	0.87283
1.325	0.284696	1.06637
1.35	0.34019	1.26174
1.375	0.397019	1.45716
1.4	0.454859	1.65144
1.425	0.515629	1.84308
1.45	0.579823	2.03043
1.475	0.646785	2.21247
1.5	0.716554	2.38821
1.525	0.78828	2.55848
1.55	0.860359	2.72057
1.575	0.929382	2.87528
1.6	0.995787	3.02509
1.625	1.059928	3.17076
1.65	1.121619	3.31243
1.675	1.182281	3.45075
1.7	1.24111	3.58532
1.725	1.298529	3.716
1.75	1.354692	3.84274
1.775	1.408151	3.96561
1.8	1.459131	4.08498
1.825	1.507915	4.20098
1.85	1.554857	4.31359
1.875	1.599026	4.42156
1.9	1.641097	4.52545
1.925	1.682571	4.62466
1.95	1.723809	4.72026
1.975	1.765067	4.81075
2	1.80563	4.8967
2.025	1.84581	4.97616
2.05	1.883549	5.05097
2.075	1.917565	5.12134
2.1	1.947975	5.18646
2.125	1.975101	5.24691
2.15	2.00016	5.30267
2.175	2.02413	5.35414

2.2	2.047072	5.40074
2.225	2.067838	5.44398
2.25	2.087066	5.48149
2.275	2.102677	5.51315
2.3	2.117113	5.53855
2.325	2.130088	5.55706
2.35	2.142201	5.56883
2.375	2.151824	5.57252
2.4	2.159713	5.56916
2.425	2.164702	5.56105
2.45	2.16578	5.54814
2.475	2.1643	5.52973
2.5	2.160654	5.50594
2.525	2.156979	5.47323
2.55	2.150139	5.43197
2.575	2.139241	5.38449
2.6	2.124208	5.33195
2.625	2.105666	5.27469
2.65	2.084949	5.21319
2.675	2.061606	5.14606
2.7	2.038203	5.07366
2.725	2.014095	4.99508
2.75	1.988811	4.90905
2.775	1.960656	4.81491
2.8	1.930129	4.71324
2.825	1.897857	4.60563
2.85	1.863587	4.49458
2.875	1.826239	4.38067
2.9	1.784785	4.26362
2.925	1.739205	4.14424
2.95	1.691293	4.01983
2.975	1.643783	3.89189
3	1.597958	3.75771
3.025	1.551104	3.61717
3.05	1.501526	3.46994
3.075	1.446528	3.32216
3.1	1.387405	3.17235
3.125	1.328056	3.02189
3.15	1.26855	2.87031
3.175	1.208349	2.71616
3.2	1.147844	2.55791
3.225	1.088828	2.3966
3.25	1.029969	2.23428
3.275	0.969464	2.07124
3.3	0.907489	1.90757
3.325	0.844937	1.7435
3.35	0.781679	1.57988
3.375	0.718613	1.41732

3.4	0.654722	1.25715
3.425	0.590307	1.09989
3.45	0.525816	0.9456
3.475	0.461607	0.795196
3.5	0.396975	0.648183
3.525	0.332386	0.503934
3.55	0.268324	0.36067
3.575	0.20525	0.217329
3.6	0.143317	0.073837
3.625	0.081727	-0.07031
3.65	0.021458	-0.21512
3.675	-0.03843	-0.36095
3.7	-0.09866	-0.50781
3.725	-0.16033	-0.65782
3.75	-0.22196	-0.81197
3.775	-0.28346	-0.97117
3.8	-0.34441	-1.134
3.825	-0.40629	-1.29878
3.85	-0.46776	-1.46506
3.875	-0.52948	-1.63249
3.9	-0.59095	-1.79935
3.925	-0.65332	-1.96489
3.95	-0.71553	-2.12892
3.975	-0.77833	-2.29054
4	-0.84089	-2.44962
4.025	-0.9031	-2.60611
4.05	-0.96412	-2.75899
4.075	-1.02509	-2.90909
4.1	-1.08513	-3.05561
4.125	-1.14398	-3.19931
4.15	-1.20109	-3.33976
4.175	-1.25743	-3.47698
4.2	-1.31253	-3.61081
4.225	-1.36597	-3.74086
4.25	-1.41852	-3.86803
4.275	-1.47004	-3.99198
4.3	-1.51958	-4.113
4.325	-1.5677	-4.23047
4.35	-1.61395	-4.34348
4.375	-1.65876	-4.45225
4.4	-1.70195	-4.55556
4.425	-1.74418	-4.6553
4.45	-1.78453	-4.75083
4.475	-1.82274	-4.84186
4.5	-1.85969	-4.92818
4.525	-1.89475	-5.01037
4.55	-1.92794	-5.08813
4.575	-1.95947	-5.16031

4.6	-1.98865	-5.22737
4.625	-2.01662	-5.28931
4.65	-2.0421	-5.34511
4.675	-2.06595	-5.39558
4.7	-2.08803	-5.44075
4.725	-2.10785	-5.48066
4.75	-2.12576	-5.51507
4.775	-2.14123	-5.54402
4.8	-2.15486	-5.56686
4.825	-2.16645	-5.5842
4.85	-2.17598	-5.59639
4.875	-2.18268	-5.60168
4.9	-2.18862	-5.60204
4.925	-2.192	-5.59643
4.95	-2.1946	-5.58676
4.975	-2.1923	-5.57123
5	-2.18676	-5.55112
5.025	-2.17712	-5.52712
5.05	-2.16627	-5.49783
5.075	-2.15328	-5.4601
5.1	-2.14171	-5.41467
5.125	-2.12824	-5.35967
5.15	-2.11256	-5.29698
5.175	-2.09361	-5.22615
5.2	-2.07087	-5.14817
5.225	-2.04594	-5.06652
5.25	-2.01889	-4.97958
5.275	-1.98881	-4.8868
5.3	-1.95614	-4.7857
5.325	-1.92154	-4.67892
5.35	-1.88541	-4.56362
5.375	-1.84881	-4.44365
5.4	-1.81255	-4.32076
5.425	-1.7737	-4.19325
5.45	-1.73046	-4.06164
5.475	-1.68241	-3.92667
5.5	-1.62933	-3.78915
5.525	-1.57366	-3.64988
5.55	-1.51741	-3.50919
5.575	-1.46205	-3.36683
5.6	-1.40736	-3.22078
5.625	-1.35257	-3.07149
5.65	-1.29485	-2.9174
5.675	-1.23694	-2.75956
5.7	-1.17679	-2.59964
5.725	-1.1153	-2.43721
5.75	-1.05427	-2.27249
5.775	-0.99428	-2.10663

5.8	-0.93409	-1.93875
5.825	-0.87244	-1.76983
5.85	-0.80996	-1.60278
5.875	-0.74678	-1.43647
5.9	-0.68292	-1.27028
5.925	-0.6184	-1.10723
5.95	-0.55316	-0.947
5.975	-0.48762	-0.78957
6	-0.42263	-0.63601
6.025	-0.35808	-0.48624
6.05	-0.2933	-0.33932
6.075	-0.22888	-0.19441
6.1	-0.16586	-0.05032
6.125	-0.1039	0.094282
6.15	-0.04231	0.239512
6.175	0.018048	0.38595
6.2	0.077349	0.534698
6.225	0.135745	0.684637
6.25	0.193938	0.83603
6.275	0.252773	0.98942
6.3	0.311805	1.14612
6.325	0.371437	1.30613
6.35	0.431077	1.46879
6.375	0.490878	1.63369
6.4	0.550525	1.79955
6.425	0.610491	1.96528
6.45	0.670859	2.12979
6.475	0.731016	2.29249
6.5	0.791464	2.45161
6.525	0.851863	2.60773
6.55	0.911821	2.76029
6.575	0.971091	2.90951
6.6	1.029901	3.05554
6.625	1.087074	3.19813
6.65	1.143238	3.3373
6.675	1.198147	3.47255
6.7	1.252126	3.60456
6.725	1.30485	3.73282
6.75	1.356172	3.85791
6.775	1.406387	3.97997
6.8	1.454721	4.09783
6.825	1.50179	4.21202
6.85	1.547351	4.32269
6.875	1.591509	4.42889
6.9	1.634061	4.53165
6.925	1.674937	4.62986
6.95	1.714431	4.72404
6.975	1.752357	4.81396

7	1.788548	4.89926
7.025	1.822701	4.97973
7.05	1.85511	5.05552
7.075	1.885676	5.12617
7.1	1.914478	5.19094
7.125	1.941546	5.25111
7.15	1.966781	5.30628
7.175	1.990056	5.35629
7.2	2.011165	5.40105
7.225	2.030079	5.44008
7.25	2.04717	5.47365
7.275	2.062546	5.50095
7.3	2.07611	5.52274
7.325	2.087585	5.53933
7.35	2.096719	5.55003
7.375	2.103363	5.55485
7.4	2.108842	5.5538
7.425	2.112664	5.54774
7.45	2.113506	5.53676
7.475	2.111341	5.52063
7.5	2.107656	5.49771
7.525	2.101364	5.46666
7.55	2.092603	5.42911
7.575	2.081323	5.38408
7.6	2.06824	5.33445
7.625	2.052227	5.27994
7.65	2.032509	5.22155
7.675	2.009509	5.15814
7.7	1.98594	5.08764
7.725	1.959705	5.00855
7.75	1.933157	4.9221
7.775	1.905639	4.82983
7.8	1.876229	4.73066
7.825	1.843869	4.62746
7.85	1.808187	4.51737
7.875	1.769507	4.40416
7.9	1.730062	4.2849
7.925	1.690685	4.161
7.95	1.650251	4.03154
7.975	1.608052	3.89789
8	1.562168	3.76243
8.025	1.510267	3.62284
8.05	1.454427	3.48122
8.075	1.396489	3.33842
8.1	1.339806	3.19395
8.125	1.283927	3.04703
8.15	1.229693	2.89567
8.175	1.173726	2.73962

8.2	1.116258	2.58014
8.225	1.056086	2.41928
8.25	0.993484	2.25648
8.275	0.931911	2.091
8.3	0.871289	1.92465
8.325	0.810825	1.75807
8.35	0.750976	1.59127
8.375	0.691693	1.42502
8.4	0.631301	1.26174
8.425	0.570074	1.10234
8.45	0.507809	0.94699
8.475	0.444744	0.796092
8.5	0.380449	0.649477
8.525	0.316428	0.505143
8.55	0.252794	0.362925
8.575	0.18959	0.221678
8.6	0.126942	0.079577
8.625	0.065949	-0.0641
8.65	0.005762	-0.20839
8.675	-0.05411	-0.35498
8.7	-0.11377	-0.50408
8.725	-0.17335	-0.65671
8.75	-0.23366	-0.81382
8.775	-0.29413	-0.97594
8.8	-0.35332	-1.14171
8.825	-0.41351	-1.31042
8.85	-0.47426	-1.48124
8.875	-0.53559	-1.65316
8.9	-0.59706	-1.82482
8.925	-0.6593	-1.99549
8.95	-0.72134	-2.16394
8.975	-0.78348	-2.32949
9	-0.84578	-2.4928
9.025	-0.90731	-2.65322
9.05	-0.96823	-2.81037
9.075	-1.02848	-2.96322
9.1	-1.08817	-3.11248
9.125	-1.14727	-3.25893
9.15	-1.20468	-3.40138
9.175	-1.26111	-3.53986
9.2	-1.31648	-3.67542
9.225	-1.37076	-3.80738
9.25	-1.4236	-3.9357
9.275	-1.4747	-4.06047
9.3	-1.5244	-4.18187
9.325	-1.57276	-4.30037
9.35	-1.61893	-4.41392
9.375	-1.66335	-4.52286

9.4	-1.70681	-4.62783
9.425	-1.749	-4.72878
9.45	-1.78928	-4.82538
9.475	-1.82739	-4.91756
9.5	-1.86348	-5.00481
9.525	-1.89831	-5.08695
9.55	-1.93135	-5.16396
9.575	-1.96306	-5.2361
9.6	-1.99249	-5.30323
9.625	-2.02027	-5.36471
9.65	-2.04567	-5.42075
9.675	-2.06983	-5.47144
9.7	-2.09207	-5.51681
9.725	-2.11225	-5.55696
9.75	-2.1298	-5.59131
9.775	-2.14551	-5.61931
9.8	-2.15959	-5.64168
9.825	-2.17156	-5.65833
9.85	-2.18132	-5.66923
9.875	-2.18879	-5.67419
9.9	-2.19485	-5.6739
9.925	-2.19874	-5.6683
9.95	-2.20073	-5.658
9.975	-2.20062	-5.6432
10	-2.19788	-5.62247
10.025	-2.19174	-5.59238
10.05	-2.18098	-5.55705
10.075	-2.16716	-5.51511
10.1	-2.15198	-5.46944
10.125	-2.13635	-5.41684
10.15	-2.11933	-5.35711
10.175	-2.10175	-5.29005
10.2	-2.08132	-5.21391
10.225	-2.0588	-5.13031
10.25	-2.03271	-5.0402
10.275	-2.00292	-4.94537
10.3	-1.96995	-4.84494
10.325	-1.93552	-4.73892
10.35	-1.89875	-4.62699
10.375	-1.86004	-4.50805
10.4	-1.82185	-4.38404
10.425	-1.78157	-4.25462
10.45	-1.74031	-4.12301
10.475	-1.69754	-3.98707
10.5	-1.65023	-3.84596
10.525	-1.59704	-3.70088
10.55	-1.53942	-3.55336
10.575	-1.48055	-3.4049

10.6	-1.42214	-3.25282
10.625	-1.36264	-3.09853
10.65	-1.30461	-2.94268
10.675	-1.24801	-2.7838
10.7	-1.19038	-2.62132
10.725	-1.12974	-2.45614
10.75	-1.06812	-2.28898
10.775	-1.00592	-2.12205
10.8	-0.94284	-1.95485
10.825	-0.87997	-1.78702
10.85	-0.81662	-1.62004
10.875	-0.75304	-1.45388
10.9	-0.68872	-1.28843
10.925	-0.62327	-1.12466
10.95	-0.55802	-0.96217
10.975	-0.49289	-0.80312
11	-0.42799	-0.64824
11.025	-0.36286	-0.49661
11.05	-0.29844	-0.34763
11.075	-0.23422	-0.2008
11.1	-0.17075	-0.05449
11.125	-0.10826	0.091816
11.15	-0.04691	0.238664
11.175	0.014044	0.386987
11.2	0.073852	0.536855
11.225	0.132918	0.687902
11.25	0.191806	0.8401
11.275	0.251086	0.99379
11.3	0.310909	1.14986
11.325	0.371412	1.30897
11.35	0.43184	1.47153
11.375	0.492045	1.6362
11.4	0.552338	1.80138
11.425	0.613031	1.96689
11.45	0.673749	2.13186
11.475	0.734233	2.29407
11.5	0.795277	2.45359
11.525	0.85573	2.61009
11.55	0.915692	2.76355
11.575	0.97505	2.9135
11.6	1.03385	3.05982
11.625	1.09121	3.20273
11.65	1.147628	3.34248
11.675	1.202998	3.47826
11.7	1.25731	3.61039
11.725	1.310053	3.73927
11.75	1.361621	3.86427
11.775	1.41214	3.98588

11.8	1.460973	4.10388
11.825	1.50817	4.21809
11.85	1.553877	4.32831
11.875	1.59835	4.43436
11.9	1.641254	4.53669
11.925	1.682189	4.6353
11.95	1.72183	4.72975
11.975	1.759756	4.81969
12	1.795673	4.90489
12.025	1.829963	4.98505
12.05	1.862989	5.06034
12.075	1.893986	5.13129
12.1	1.922436	5.19694
12.125	1.949366	5.25698
12.15	1.974778	5.3114
12.175	1.998366	5.36145
12.2	2.019377	5.40636
12.225	2.03835	5.4452
12.25	2.055843	5.47802
12.275	2.071278	5.50557
12.3	2.084734	5.52765
12.325	2.096239	5.54366
12.35	2.105294	5.55456
12.375	2.111997	5.55895
12.4	2.117652	5.55806
12.425	2.121395	5.55193
12.45	2.122767	5.54146
12.475	2.122111	5.5256
12.5	2.118671	5.50361
12.525	2.111782	5.47297
12.55	2.101952	5.43611
12.575	2.089673	5.39228
12.6	2.075737	5.34322
12.625	2.060508	5.28821
12.65	2.044505	5.22747
12.675	2.02511	5.16215
12.7	2.00014	5.09043
12.725	1.971514	5.01179
12.75	1.940977	4.92736
12.775	1.911381	4.83619
12.8	1.88155	4.73889
12.825	1.851092	4.63561
12.85	1.817625	4.52745
12.875	1.781394	4.4124
12.9	1.740842	4.2935
12.925	1.698721	4.16773
12.95	1.656993	4.03716
12.975	1.614667	3.90262

13	1.570488	3.76617
13.025	1.521106	3.6255
13.05	1.466491	3.48421
13.075	1.408279	3.34308
13.1	1.349871	3.19759
13.125	1.29211	3.04966
13.15	1.234633	2.89782
13.175	1.179076	2.7423
13.2	1.122432	2.58357
13.225	1.063809	2.42199
13.25	1.001451	2.25947
13.275	0.939408	2.0933
13.3	0.878366	1.92661
13.325	0.817504	1.76004
13.35	0.755918	1.59176
13.375	0.694639	1.42562
13.4	0.633566	1.26141
13.425	0.572173	1.10006
13.45	0.509558	0.94225
13.475	0.446584	0.789634
13.5	0.382923	0.640834
13.525	0.318721	0.49501
13.55	0.254463	0.350889
13.575	0.191056	0.207582
13.6	0.128505	0.064653
13.625	0.066658	-0.07897
13.65	0.005494	-0.22342
13.675	-0.05484	-0.36924
13.7	-0.11472	-0.5172
13.725	-0.1753	-0.66793
13.75	-0.23628	-0.82316
13.775	-0.29692	-0.98329
13.8	-0.35626	-1.14702
13.825	-0.41678	-1.31321
13.85	-0.47764	-1.48231
13.875	-0.53829	-1.65266
13.9	-0.59971	-1.82239
13.925	-0.66164	-1.99084
13.95	-0.72326	-2.1588
13.975	-0.78475	-2.32333
14	-0.84622	-2.48547
14.025	-0.90746	-2.64502
14.05	-0.9679	-2.80094
14.075	-1.0279	-2.95334
14.1	-1.08754	-3.10235
14.125	-1.14638	-3.24782
14.15	-1.20382	-3.38969
14.175	-1.26022	-3.5287

14.2	-1.31579	-3.66318
14.225	-1.3696	-3.79517
14.25	-1.4226	-3.92272
14.275	-1.47425	-4.0474
14.3	-1.5241	-4.16883
14.325	-1.57222	-4.28628
14.35	-1.61877	-4.39969
14.375	-1.66377	-4.50874
14.4	-1.70719	-4.61348
14.425	-1.74929	-4.71485
14.45	-1.78885	-4.81117
14.475	-1.8273	-4.90295
14.5	-1.86425	-4.99065
14.525	-1.89881	-5.07354
14.55	-1.93197	-5.15111
14.575	-1.96342	-5.22334
14.6	-1.99314	-5.2907
14.625	-2.02083	-5.35303
14.65	-2.04664	-5.40952
14.675	-2.07083	-5.46059
14.7	-2.09278	-5.5064
14.725	-2.11311	-5.5469
14.75	-2.13094	-5.58125
14.775	-2.14689	-5.60953
14.8	-2.16082	-5.63178
14.825	-2.17294	-5.64836
14.85	-2.18313	-5.66032
14.875	-2.19033	-5.66607
14.9	-2.19644	-5.66587
14.925	-2.20039	-5.66088
14.95	-2.20274	-5.65163
14.975	-2.20218	-5.63659
15	-2.2002	-5.61632
15.025	-2.19311	-5.59016
15.05	-2.18097	-5.55812
15.075	-2.16517	-5.5198
15.1	-2.14998	-5.47648
15.125	-2.13719	-5.42443
15.15	-2.12367	-5.36363
15.175	-2.10961	-5.29455
15.2	-2.09091	-5.21697
15.225	-2.06711	-5.13299
15.25	-2.03802	-5.04284
15.275	-2.00577	-4.94894
15.3	-1.97189	-4.84942
15.325	-1.93846	-4.74364
15.35	-1.90499	-4.63247
15.375	-1.86992	-4.51335

15.4	-1.83285	-4.38899
15.425	-1.79363	-4.26076
15.45	-1.75167	-4.12873
15.475	-1.70547	-3.9925
15.5	-1.65404	-3.85235
15.525	-1.60019	-3.70945
15.55	-1.54344	-3.56411
15.575	-1.48508	-3.41724
15.6	-1.42816	-3.26895
15.625	-1.37218	-3.11689
15.65	-1.31492	-2.96031
15.675	-1.25608	-2.80033
15.7	-1.19695	-2.637
15.725	-1.13541	-2.4708
15.75	-1.07212	-2.30225
15.775	-1.00947	-2.13231
15.8	-0.94737	-1.96177
15.825	-0.88548	-1.79114
15.85	-0.82284	-1.62091
15.875	-0.75942	-1.45059
15.9	-0.69464	-1.28225
15.925	-0.62883	-1.11662
15.95	-0.5622	-0.95295
15.975	-0.49594	-0.79318
16	-0.42992	-0.63829
16.025	-0.36377	-0.48741
16.05	-0.29779	-0.33942
16.075	-0.2321	-0.193
16.1	-0.16792	-0.0469
16.125	-0.10502	0.099388
16.15	-0.04339	0.246575
16.175	0.018014	0.394782
16.2	0.078217	0.544638
16.225	0.137135	0.695981
16.25	0.196104	0.84881
16.275	0.255452	1.0032
16.3	0.315161	1.16049
16.325	0.375143	1.32042
16.35	0.435722	1.48313
16.375	0.495971	1.64797
16.4	0.556334	1.81367
16.425	0.617059	1.97972
16.45	0.677583	2.1442
16.475	0.73775	2.30658
16.5	0.798218	2.46554
16.525	0.858757	2.6219
16.55	0.919082	2.77417
16.575	0.979078	2.92345

16.6	1.037888	3.06902
16.625	1.095522	3.21092
16.65	1.152352	3.34938
16.675	1.207536	3.48439
16.7	1.261573	3.61583
16.725	1.314326	3.74423
16.75	1.365923	3.86879
16.775	1.415776	3.99038
16.8	1.464198	4.10825
16.825	1.51163	4.22278
16.85	1.557062	4.33399
16.875	1.600614	4.44022
16.9	1.643077	4.54259
16.925	1.684276	4.64147
16.95	1.723751	4.73569
16.975	1.76149	4.82554
17	1.797437	4.91065
17.025	1.831639	4.99077
17.05	1.864136	5.06595
17.075	1.894937	5.13632
17.1	1.92369	5.20162
17.125	1.950523	5.26146
17.15	1.975601	5.31637
17.175	1.998837	5.366
17.2	2.019838	5.41041
17.225	2.038605	5.44884
17.25	2.055696	5.4813
17.275	2.071102	5.50822
17.3	2.084332	5.53013
17.325	2.095514	5.54641
17.35	2.104402	5.55687
17.375	2.110851	5.56128
17.4	2.116094	5.55968
17.425	2.119533	5.55376
17.45	2.120788	5.54392
17.475	2.119102	5.52691
17.5	2.114976	5.50425
17.525	2.10792	5.47363
17.55	2.09812	5.43649
17.575	2.085968	5.39119
17.6	2.072131	5.34251
17.625	2.057372	5.2864
17.65	2.040506	5.22688
17.675	2.018711	5.16133
17.7	1.991849	5.09077
17.725	1.963831	5.01282
17.75	1.934901	4.92712
17.775	1.906648	4.83544

17.8	1.878257	4.73742
17.825	1.847093	4.63435
17.85	1.813372	4.52556
17.875	1.775798	4.41123
17.9	1.734168	4.29225
17.925	1.692802	4.1661
17.95	1.651672	4.03609
17.975	1.609982	3.90203
18	1.564814	3.76488
18.025	1.514129	3.6247
18.05	1.459141	3.48552
18.075	1.401664	3.34347
18.1	1.343413	3.19852
18.125	1.286034	3.05117
18.15	1.229938	2.89991
18.175	1.174265	2.74373
18.2	1.118218	2.58503
18.225	1.059771	2.42358
18.25	0.99811	2.26062
18.275	0.936115	2.09586
18.3	0.875056	1.93051
18.325	0.813798	1.7643
18.35	0.752915	1.59745
18.375	0.691853	1.4313
18.4	0.630186	1.26813
18.425	0.56878	1.10778
18.45	0.506857	0.95151
18.475	0.444359	0.79907
18.5	0.380753	0.65015
18.525	0.317203	0.504099
18.55	0.253879	0.359922
18.575	0.190804	0.216165
18.6	0.128609	0.072408
18.625	0.067307	-0.07144
18.65	0.006675	-0.21632
18.675	-0.05347	-0.36309
18.7	-0.1136	-0.51203
18.725	-0.17419	-0.66421
18.75	-0.23496	-0.82083
18.775	-0.29528	-0.98195
18.8	-0.35491	-1.14646
18.825	-0.41546	-1.31314
18.85	-0.47623	-1.48274
18.875	-0.53737	-1.6536
18.9	-0.59917	-1.82395
18.925	-0.66126	-1.99354
18.95	-0.72253	-2.16145
18.975	-0.78445	-2.327

19	-0.84647	-2.4902
19.025	-0.90736	-2.65017
19.05	-0.96777	-2.80674
19.075	-1.02791	-2.95963
19.1	-1.08768	-3.10921
19.125	-1.1462	-3.25533
19.15	-1.2035	-3.39763
19.175	-1.25982	-3.53609
19.2	-1.31481	-3.67106
19.225	-1.36886	-3.80254
19.25	-1.42163	-3.93032
19.275	-1.47288	-4.05479
19.3	-1.52239	-4.17577
19.325	-1.57041	-4.29328
19.35	-1.61681	-4.40645
19.375	-1.66172	-4.51576
19.4	-1.70444	-4.61996
19.425	-1.74638	-4.72024
19.45	-1.78619	-4.81646
19.475	-1.82412	-4.90777
19.5	-1.86053	-4.99482
19.525	-1.89502	-5.07678
19.55	-1.928	-5.15345
19.575	-1.95919	-5.22495
19.6	-1.98883	-5.29245
19.625	-2.01655	-5.3546
19.65	-2.04172	-5.41035
19.675	-2.06515	-5.46058
19.7	-2.08736	-5.50558
19.725	-2.10714	-5.54509
19.75	-2.1249	-5.57894
19.775	-2.14088	-5.60706
19.8	-2.15453	-5.62935
19.825	-2.1663	-5.64615
19.85	-2.17584	-5.65764
19.875	-2.18293	-5.66288
19.9	-2.18868	-5.66215
19.925	-2.19267	-5.65659
19.95	-2.19478	-5.64637
19.975	-2.19406	-5.63048
20	-2.19131	-5.60866
20.025	-2.18537	-5.57882
20.05	-2.17477	-5.54309
20.075	-2.15955	-5.50265
20.1	-2.14212	-5.45546
20.125	-2.12593	-5.40407
20.15	-2.11082	-5.34443
20.175	-2.09456	-5.277

20.2	-2.07565	-5.19953
20.225	-2.05321	-5.11669
20.25	-2.02604	-5.02653
20.275	-1.99436	-4.93144
20.3	-1.96097	-4.83065
20.325	-1.92701	-4.72448
20.35	-1.89153	-4.61195
20.375	-1.85396	-4.49475
20.4	-1.81503	-4.37049
20.425	-1.77573	-4.24463
20.45	-1.7358	-4.11464
20.475	-1.69309	-3.98041
20.5	-1.64412	-3.84079
20.525	-1.5898	-3.69609
20.55	-1.53154	-3.55003
20.575	-1.47214	-3.4018
20.6	-1.41425	-3.25135
20.625	-1.35822	-3.099
20.65	-1.30237	-2.94319
20.675	-1.24507	-2.78465
20.7	-1.18737	-2.62299
20.725	-1.12589	-2.45905
20.75	-1.06385	-2.29282
20.775	-1.00143	-2.12605
20.8	-0.93937	-1.95854
20.825	-0.87794	-1.79054
20.85	-0.81576	-1.62306
20.875	-0.75238	-1.45596
20.9	-0.68864	-1.28915
20.925	-0.62428	-1.12526
20.95	-0.55931	-0.9638
20.975	-0.49362	-0.80606
21	-0.42891	-0.65248
21.025	-0.36403	-0.5023
21.05	-0.29918	-0.3553
21.075	-0.23466	-0.20955
21.1	-0.17145	-0.06384
21.125	-0.10922	0.081936
21.15	-0.04771	0.228316
21.175	0.012925	0.376803
21.2	0.072085	0.526515
21.225	0.131205	0.67758
21.25	0.189901	0.83012
21.275	0.24894	0.98463
21.3	0.308621	1.14184
21.325	0.368942	1.30228
21.35	0.429313	1.4655
21.375	0.489167	1.63071

21.4	0.549814	1.79715
21.425	0.610205	1.96354
21.45	0.670942	2.12901
21.475	0.731773	2.29227
21.5	0.792709	2.45247
21.525	0.853444	2.60923
21.55	0.913438	2.76248
21.575	0.972973	2.91288
21.6	1.031773	3.05985
21.625	1.089093	3.20332
21.65	1.145443	3.3428
21.675	1.200627	3.47882
21.7	1.254977	3.61083
21.725	1.307897	3.73894
21.75	1.359749	3.86392
21.775	1.410072	3.98576
21.8	1.458935	4.10382
21.825	1.506073	4.21792
21.85	1.551702	4.32808
21.875	1.596213	4.4339
21.9	1.638843	4.53656
21.925	1.679729	4.63474
21.95	1.719047	4.72919
21.975	1.757335	4.81914
22	1.793076	4.90419
22.025	1.82718	4.98464
22.05	1.85998	5.0603
22.075	1.890478	5.131
22.1	1.919114	5.19668
22.125	1.945799	5.25664
22.15	1.971279	5.31133
22.175	1.994417	5.36145
22.2	2.015369	5.40577
22.225	2.03444	5.44445
22.25	2.051757	5.47763
22.275	2.066907	5.50523
22.3	2.080324	5.52677
22.325	2.091721	5.543
22.35	2.100737	5.55386
22.375	2.107381	5.55836
22.4	2.112683	5.55772
22.425	2.116368	5.55191
22.45	2.117662	5.54097
22.475	2.116966	5.52568
22.5	2.113516	5.50316
22.525	2.106539	5.47237
22.55	2.096347	5.43501
22.575	2.083705	5.3908

22.6	2.069759	5.34141
22.625	2.054334	5.28551
22.65	2.037752	5.22439
22.675	2.018103	5.15828
22.7	1.993731	5.08777
22.725	1.964909	5.01072
22.75	1.934549	4.92614
22.775	1.905109	4.83506
22.8	1.875807	4.73872
22.825	1.845339	4.63598
22.85	1.813215	4.52568
22.875	1.776328	4.41116
22.9	1.735403	4.29099
22.925	1.692185	4.16669
22.95	1.650006	4.03835
22.975	1.607787	3.90693
23	1.56357	3.76923
23.025	1.514276	3.62896
23.05	1.461463	3.48729
23.075	1.405476	3.34437
23.1	1.346735	3.19868
23.125	1.28867	3.04839
23.15	1.230703	2.89767
23.175	1.17349	2.74114
23.2	1.116582	2.58136
23.225	1.058164	2.42043
23.25	0.999119	2.2559
23.275	0.938467	2.09081
23.3	0.877145	1.9244
23.325	0.815206	1.75761
23.35	0.75325	1.59041
23.375	0.690247	1.42344
23.4	0.626979	1.26024
23.425	0.565011	1.09872
23.45	0.502863	0.94062
23.475	0.43986	0.785958
23.5	0.376666	0.634573
23.525	0.313691	0.486054
23.55	0.250538	0.339349
23.575	0.187222	0.193718
23.6	0.124663	0.048235
23.625	0.06295	-0.09712
23.65	0.001502	-0.24293
23.675	-0.05938	-0.38981
23.7	-0.11967	-0.53795
23.725	-0.18066	-0.68883
23.75	-0.24207	-0.84346
23.775	-0.30316	-1.0024

23.8	-0.36342	-1.16512
23.825	-0.42461	-1.33083
23.85	-0.48607	-1.49925
23.875	-0.54731	-1.66899
23.9	-0.60866	-1.83898
23.925	-0.67007	-2.00811
23.95	-0.73169	-2.17557
23.975	-0.79339	-2.34091
24	-0.8545	-2.50415
24.025	-0.9155	-2.66477
24.05	-0.97591	-2.82175
24.075	-1.03574	-2.97458
24.1	-1.09505	-3.12366
24.125	-1.15377	-3.26936
24.15	-1.21172	-3.41126
24.175	-1.26794	-3.54916
24.2	-1.3231	-3.68295
24.225	-1.37791	-3.81358
24.25	-1.43063	-3.94054
24.275	-1.48184	-4.06386
24.3	-1.53129	-4.18319
24.325	-1.57967	-4.29903
24.35	-1.62619	-4.41118
24.375	-1.67049	-4.51894
24.4	-1.71336	-4.62229
24.425	-1.75529	-4.72158
24.45	-1.79489	-4.81688
24.475	-1.83261	-4.90754
24.5	-1.86877	-4.99339
24.525	-1.90314	-5.07454
24.55	-1.93614	-5.15049
24.575	-1.9669	-5.22184
24.6	-1.99636	-5.28828
24.625	-2.02381	-5.34855
24.65	-2.04887	-5.40401
24.675	-2.07215	-5.45379
24.7	-2.09411	-5.49774
24.725	-2.11389	-5.536
24.75	-2.13134	-5.56937
24.775	-2.14661	-5.59643
24.8	-2.16034	-5.61827
24.825	-2.17188	-5.6343
24.85	-2.18123	-5.64482
24.875	-2.18793	-5.64865
24.9	-2.19364	-5.64732
24.925	-2.19733	-5.64175
24.95	-2.19873	-5.62994
24.975	-2.19659	-5.61311

25	-2.19286	-5.59038
25.025	-2.18635	-5.55856
25.05	-2.17714	-5.51897
25.075	-2.16536	-5.47313
25.1	-2.1514	-5.42165
25.125	-2.13587	-5.36783
25.15	-2.11779	-5.30795
25.175	-2.09558	-5.24238
25.2	-2.06936	-5.17024
25.225	-2.04267	-5.08955
25.25	-2.01595	-5.00288
25.275	-1.98749	-4.91063
25.3	-1.95732	-4.81035
25.325	-1.92541	-4.70491
25.35	-1.88979	-4.59321
25.375	-1.85018	-4.47499
25.4	-1.81038	-4.35423
25.425	-1.77042	-4.22925
25.45	-1.729	-4.10104
25.475	-1.68564	-3.96801
25.5	-1.63787	-3.82851
25.525	-1.58528	-3.68496
25.55	-1.52861	-3.5369
25.575	-1.47046	-3.38569
25.6	-1.41189	-3.23356
25.625	-1.35479	-3.08
25.65	-1.29753	-2.92356
25.675	-1.24041	-2.76426
25.7	-1.1817	-2.6036
25.725	-1.11979	-2.44038
25.75	-1.05702	-2.27367
25.775	-0.99465	-2.10644
25.8	-0.933	-1.93913
25.825	-0.87115	-1.77024
25.85	-0.8094	-1.6022
25.875	-0.74691	-1.43376
25.9	-0.68286	-1.26658
25.925	-0.61776	-1.10204
25.95	-0.55249	-0.94012
25.975	-0.48692	-0.78147
26	-0.42151	-0.62688
26.025	-0.35618	-0.47632
26.05	-0.29145	-0.32869
26.075	-0.22693	-0.18277
26.1	-0.16345	-0.03842
26.125	-0.10134	0.105489
26.15	-0.04008	0.250635
26.175	0.020546	0.396809

26.2	0.07998	0.544701
26.225	0.138582	0.694226
26.25	0.196583	0.84512
26.275	0.255432	0.99784
26.3	0.314751	1.15321
26.325	0.374212	1.31182
26.35	0.433674	1.47371
26.375	0.493413	1.63798
26.4	0.553362	1.80314
26.425	0.61302	1.96848
26.45	0.672887	2.13244
26.475	0.733001	2.29472
26.5	0.793175	2.45435
26.525	0.852943	2.61044
26.55	0.912742	2.76283
26.575	0.971865	2.91219
26.6	1.030312	3.05794
26.625	1.087603	3.20021
26.65	1.144022	3.33908
26.675	1.198912	3.47483
26.7	1.252537	3.60629
26.725	1.304859	3.73437
26.75	1.356378	3.85927
26.775	1.406231	3.98109
26.8	1.454378	4.09857
26.825	1.501428	4.21296
26.85	1.546733	4.32311
26.875	1.590627	4.42929
26.9	1.632954	4.53182
26.925	1.673702	4.63028
26.95	1.712951	4.72421
26.975	1.750818	4.81392
27	1.786529	4.89931
27.025	1.820408	4.97938
27.05	1.853042	5.05444
27.075	1.883765	5.12493
27.1	1.912205	5.19023
27.125	1.939164	5.24996
27.15	1.964331	5.30511
27.175	1.987429	5.35452
27.2	2.008205	5.39896
27.225	2.027237	5.43753
27.25	2.044465	5.47043
27.275	2.059861	5.49798
27.3	2.073121	5.51991
27.325	2.084136	5.53618
27.35	2.09325	5.54683
27.375	2.099835	5.55095

27.4	2.105294	5.5497
27.425	2.10891	5.54371
27.45	2.110331	5.53321
27.475	2.109528	5.51829
27.5	2.106049	5.49639
27.525	2.099042	5.46635
27.55	2.089016	5.42917
27.575	2.0766	5.38524
27.6	2.062605	5.33612
27.625	2.047317	5.28123
27.65	2.031079	5.2204
27.675	2.012694	5.15456
27.7	1.988635	5.0853
27.725	1.96043	5.00749
27.75	1.929208	4.9226
27.775	1.89918	4.832
27.8	1.869565	4.73587
27.825	1.839675	4.6323
27.85	1.808099	4.52462
27.875	1.772594	4.41014
27.9	1.731189	4.29048
27.925	1.688137	4.16411
27.95	1.645841	4.03449
27.975	1.603583	3.90089
28	1.55912	3.7635
28.025	1.511375	3.62347
28.05	1.458318	3.48124
28.075	1.401007	3.3385
28.1	1.342482	3.1942
28.125	1.28378	3.04606
28.15	1.226401	2.89508
28.175	1.170325	2.73966
28.2	1.114504	2.58175
28.225	1.056851	2.41913
28.25	0.99612	2.25629
28.275	0.933988	2.09025
28.3	0.872656	1.92442
28.325	0.811719	1.75759
28.35	0.749885	1.58966
28.375	0.687746	1.4236
28.4	0.626467	1.25944
28.425	0.565348	1.09737
28.45	0.503308	0.93922
28.475	0.440737	0.784978
28.5	0.377646	0.634188
28.525	0.314134	0.487043
28.55	0.250011	0.341568
28.575	0.186695	0.196864

28.6	0.124542	0.052808
28.625	0.062256	-0.09148
28.65	0.000914	-0.23678
28.675	-0.05938	-0.38325
28.7	-0.11978	-0.5315
28.725	-0.18072	-0.68249
28.75	-0.24172	-0.83762
28.775	-0.30216	-0.99734
28.8	-0.36207	-1.1608
28.825	-0.4233	-1.32734
28.85	-0.48459	-1.49675
28.875	-0.54547	-1.66707
28.9	-0.60706	-1.83765
28.925	-0.6685	-2.0073
28.95	-0.73015	-2.17493
28.975	-0.79201	-2.34105
29	-0.85362	-2.50535
29.025	-0.91435	-2.66578
29.05	-0.97476	-2.82249
29.075	-1.03462	-2.97565
29.1	-1.09417	-3.12483
29.125	-1.15329	-3.27044
29.15	-1.21114	-3.41285
29.175	-1.26751	-3.5514
29.2	-1.32276	-3.68555
29.225	-1.37745	-3.81617
29.25	-1.43036	-3.94357
29.275	-1.48164	-4.06732
29.3	-1.53189	-4.1879
29.325	-1.57995	-4.30473
29.35	-1.62617	-4.41794
29.375	-1.67114	-4.52647
29.4	-1.71474	-4.63064
29.425	-1.75625	-4.73069
29.45	-1.79601	-4.82675
29.475	-1.83469	-4.91834
29.5	-1.87129	-5.0053
29.525	-1.90567	-5.08746
29.55	-1.93861	-5.16428
29.575	-1.97021	-5.23583
29.6	-1.99977	-5.30241
29.625	-2.02693	-5.36354
29.65	-2.05288	-5.41963
29.675	-2.07696	-5.47014
29.7	-2.09905	-5.5151
29.725	-2.11877	-5.55443
29.75	-2.13693	-5.58823
29.775	-2.1526	-5.61638

29.8	-2.16615	-5.63865
29.825	-2.17816	-5.65499
29.85	-2.18788	-5.66549
29.875	-2.1951	-5.66966
29.9	-2.20078	-5.66821
29.925	-2.205	-5.66286
29.95	-2.20645	-5.65109
29.975	-2.20457	-5.63337
30	-2.20046	-5.61021
30.025	-2.19438	-5.57924
30.05	-2.18512	-5.53987
30.075	-2.17341	-5.49277
30.1	-2.15985	-5.44121
30.125	-2.14274	-5.38574
30.15	-2.12205	-5.32511
30.175	-2.10179	-5.25832
30.2	-2.07891	-5.18469
30.225	-2.05361	-5.10418
30.25	-2.02638	-5.0178
30.275	-1.99717	-4.92215
30.3	-1.96483	-4.82365
30.325	-1.93145	-4.71733
30.35	-1.89623	-4.6046
30.375	-1.85929	-4.48518
30.4	-1.82062	-4.3603
30.425	-1.7788	-4.23392
30.45	-1.7345	-4.10398
30.475	-1.68803	-3.97096
30.5	-1.63937	-3.83271
30.525	-1.58841	-3.68904
30.55	-1.53492	-3.54135
30.575	-1.47832	-3.38933
30.6	-1.42003	-3.2341
30.625	-1.35968	-3.07646
30.65	-1.30057	-2.91813
30.675	-1.24206	-2.75898
30.7	-1.18151	-2.59755
30.725	-1.12028	-2.43333
30.75	-1.0589	-2.26668
30.775	-0.9971	-2.09953
30.8	-0.93417	-1.93215
30.825	-0.8716	-1.76473
30.85	-0.80846	-1.59746
30.875	-0.74377	-1.43026
30.9	-0.68003	-1.263
30.925	-0.61555	-1.09831
30.95	-0.54933	-0.93563
30.975	-0.48349	-0.77497

31	-0.41894	-0.61832
31.025	-0.3541	-0.46534
31.05	-0.28892	-0.31565
31.075	-0.22474	-0.16807
31.1	-0.16736	-0.02314
31.125	-0.12825	0.100151
31.15	-0.0957	0.187437
31.175	-0.06412	0.238842
31.2	-0.03342	0.260077
31.225	-0.00591	0.259043
31.25	0.017285	0.243372
31.275	0.034759	0.218676
31.3	0.047008	0.190675
31.325	0.055055	0.163817
31.35	0.059046	0.139468
31.375	0.060818	0.122335
31.4	0.057604	0.110648
31.425	0.05023	0.10638
31.45	0.041006	0.10612
31.475	0.033563	0.110623
31.5	0.031217	0.117376
31.525	0.032703	0.122277
31.55	0.034168	0.12536
31.575	0.034671	0.127257
31.6	0.033079	0.125383
31.625	0.029316	0.122927
31.65	0.026046	0.120191
31.675	0.023878	0.116548
31.7	0.02354	0.114525
31.725	0.024434	0.111622
31.75	0.024239	0.107393
31.775	0.023295	0.102459
31.8	0.02219	0.097322
31.825	0.020138	0.09243
31.85	0.017406	0.087425
31.875	0.014662	0.082447
31.9	0.012209	0.077592
31.925	0.010395	0.072733
31.95	0.009615	0.067763
31.975	0.009718	0.063395
32	0.010167	0.059478

Table S7. Test result Data of the Slalom Test at an Adhesion Coefficient of 0.2

Time	carsim	DRL-UKF	UKF
0	0.030444	0.030493	-0.01145
0.01	0.03087	0.030493	-0.01145
0.02	0.031283	0.030493	-0.01145
0.03	0.031687	0.030493	-0.01145
0.04	0.032089	0.030493	-0.01145
0.05	0.032491	0.030493	-0.01145
0.06	0.0329	0.028444	#######
0.07	0.03332	0.02887	-0.00344
0.08	0.033755	0.029283	-0.00627
0.09	0.034211	0.029689	-0.00852
0.1	0.034692	0.03009	-0.01024
0.11	0.035203	0.030493	-0.01145
0.12	0.035749	0.030903	-0.0122
0.13	0.036335	0.031323	-0.01251
0.14	0.036965	0.031758	-0.01243
0.15	0.037644	0.032215	-0.012
0.16	0.038377	0.032696	-0.01123
0.17	0.039169	0.033208	-0.01018
0.18	0.040025	0.033754	-0.00888
0.19	0.040949	0.03434	-0.00737
0.2	0.041946	0.034971	-0.00567
0.21	0.043017	0.035651	-0.00384
0.22	0.044148	0.036385	-0.00189
0.23	0.045322	0.037178	0.000127
0.24	0.046521	0.038034	0.002179
0.25	0.047727	0.038959	0.00423
0.26	0.048923	0.039957	0.006245
0.27	0.050091	0.041029	0.008191
0.28	0.051214	0.042162	0.010052
0.29	0.052273	0.043337	0.011814
0.3	0.053251	0.044537	0.013463
0.31	0.054134	0.045745	0.014986
0.32	0.054921	0.046942	0.016371
0.33	0.055616	0.048111	0.017603
0.34	0.05622	0.049234	0.018669
0.35	0.056737	0.050295	0.019557
0.36	0.057171	0.051274	0.020252
0.37	0.057522	0.052157	0.020749
0.38	0.057795	0.052945	0.021066
0.39	0.057993	0.05364	0.021231
0.4	0.058118	0.054245	0.02127
0.41	0.058173	0.054763	0.021208
0.42	0.058168	0.055196	0.021074
0.43	0.058108	0.055548	0.020893
0.44	0.058004	0.055822	0.020691
0.45	0.057863	0.05602	0.020496
0.46	0.057693	0.056145	0.020333

0.47	0.057503	0.056201	0.020222
0.48	0.057301	0.056196	0.020158
0.49	0.057095	0.056137	0.020129
0.5	0.056893	0.056033	0.020121
0.51	0.056703	0.055892	0.020122
0.52	0.056524	0.055723	0.02012
0.53	0.056353	0.055532	0.020103
0.54	0.05619	0.055329	0.020057
0.55	0.056032	0.055122	0.019972
0.56	0.055877	0.054919	0.019833
0.57	0.055723	0.054727	0.019634
0.58	0.05557	0.054545	0.019381
0.59	0.055413	0.054372	0.019086
0.6	0.055253	0.054207	0.018761
0.61	0.055087	0.054047	0.018418
0.62	0.054915	0.053891	0.018068
0.63	0.054736	0.053738	0.017723
0.64	0.05455	0.053586	0.017394
0.65	0.054358	0.053433	0.017094
0.66	0.054158	0.053278	0.016834
0.67	0.053952	0.053118	0.016622
0.68	0.053738	0.052954	0.016454
0.69	0.053516	0.052783	0.016321
0.7	0.053286	0.052605	0.016218
0.71	0.05305	0.052418	0.016134
0.72	0.052818	0.05222	0.016064
0.73	0.052601	0.052012	0.015998
0.74	0.05241	0.051792	0.01593
0.75	0.052258	0.051557	0.015851
0.76	0.052154	0.051309	0.015753
0.77	0.052112	0.051047	0.015632
0.78	0.052142	0.050784	0.01549
0.79	0.052257	0.050536	0.015334
0.8	0.052467	0.050317	0.01517
0.81	0.052773	0.050144	0.015004
0.82	0.053137	0.050031	0.01484
0.83	0.053508	0.049994	0.014687
0.84	0.053837	0.050048	0.014548
0.85	0.054072	0.050207	0.014431
0.86	0.054166	0.050488	0.014341
0.87	0.054067	0.050893	0.014283
0.88	0.053725	0.05137	0.014253
0.89	0.053091	0.051856	0.014248
0.9	0.052115	0.052287	0.014264
0.91	0.050765	0.052599	0.014296
0.92	0.049078	0.052728	0.014339
0.93	0.047109	0.05261	0.01439
0.94	0.044913	0.052182	0.014444

0.95	0.042547	0.051379	0.014498
0.96	0.040064	0.050137	0.014546
0.97	0.03752	0.048414	0.014585
0.98	0.034971	0.046255	0.014617
0.99	0.032471	0.043725	0.014642
1	0.030076	0.040889	0.014663
1.01	0.027826	0.037814	0.01468
1.02	0.025707	0.034565	0.014696
1.03	0.02369	0.031208	0.014711
1.04	0.021746	0.027809	0.014728
1.05	0.019844	0.024433	0.014748
1.06	0.017957	0.021147	0.014773
1.07	0.016055	0.017999	0.014803
1.08	0.014108	0.014975	0.014838
1.09	0.012088	0.012046	0.014877
1.1	0.009966	0.00918	0.014918
1.11	0.007726	0.006347	0.014962
1.12	0.005408	0.003516	0.015006
1.13	0.003065	0.000658	0.01505
1.14	0.000752	-0.00266	0.015093
1.15	-0.00174	-0.00619	0.015133
1.16	-0.00421	-0.00987	0.015169
1.17	-0.00645	-0.01371	0.015201
1.18	-0.00841	-0.01764	0.015229
1.19	-0.01001	-0.0216	0.015253
1.2	-0.01121	-0.0255	0.015275
1.21	-0.01195	-0.02927	0.015295
1.22	-0.01228	-0.03284	0.015313
1.23	-0.01227	-0.03611	0.01533
1.24	-0.01196	-0.03903	0.015346
1.25	-0.01144	-0.04151	0.015364
1.26	-0.01076	-0.04347	0.015382
1.27	-0.00999	-0.04487	0.015401
1.28	-0.00918	-0.04574	0.015422
1.29	-0.0084	-0.04617	0.015444
1.3	-0.00772	-0.04622	0.015468
1.31	-0.00719	-0.04598	0.015492
1.32	-0.00679	-0.04551	0.015517
1.33	-0.00652	-0.04488	0.015542
1.34	-0.00634	-0.04418	0.015568
1.35	-0.00625	-0.04347	0.015594
1.36	-0.00623	-0.04282	0.01562
1.37	-0.00624	-0.0423	0.015646
1.38	-0.00629	-0.0419	0.015672
1.39	-0.00633	-0.04161	0.015698
1.4	-0.00637	-0.04141	0.015724
1.41	-0.00638	-0.04129	0.015751
1.42	-0.00636	-0.04123	0.015778

1.43	-0.00632	-0.04123	0.015807
1.44	-0.00627	-0.04125	0.015836
1.45	-0.0062	-0.04129	0.015867
1.46	-0.00611	-0.04134	0.015898
1.47	-0.00603	-0.04137	0.015932
1.48	-0.00594	-0.0414	0.015966
1.49	-0.00586	-0.04141	0.016001
1.5	-0.00579	-0.04141	0.016035
1.51	-0.00572	-0.04141	0.016069
1.52	-0.00567	-0.0414	0.016101
1.53	-0.00563	-0.04138	0.016131
1.54	-0.0056	-0.04136	0.016158
1.55	-0.00557	-0.04134	0.016182
1.56	-0.00556	-0.04132	0.016202
1.57	-0.00554	-0.0413	0.016218
1.58	-0.00553	-0.04127	0.01623
1.59	-0.00552	-0.04125	0.016241
1.6	-0.00551	-0.04123	0.016249
1.61	-0.0055	-0.04121	0.016258
1.62	-0.00549	-0.0412	0.016268
1.63	-0.00548	-0.04119	0.01628
1.64	-0.00547	-0.04118	0.016295
1.65	-0.00545	-0.04118	0.016314
1.66	-0.00544	-0.04118	0.016338
1.67	-0.00542	-0.04119	0.016369
1.68	-0.00541	-0.04121	0.016405
1.69	-0.00539	-0.04122	0.016445
1.7	-0.00537	-0.04124	0.016488
1.71	-0.00535	-0.04126	0.016533
1.72	-0.00533	-0.04128	0.016578
1.73	-0.00531	-0.0413	0.016623
1.74	-0.00529	-0.04131	0.016666
1.75	-0.00526	-0.04133	0.016707
1.76	-0.00524	-0.04133	0.016743
1.77	-0.00521	-0.04133	0.016775
1.78	-0.00519	-0.04133	0.016802
1.79	-0.00516	-0.04132	0.016825
1.8	-0.00513	-0.0413	0.016845
1.81	-0.0051	-0.04129	0.016862
1.82	-0.00507	-0.04127	0.016876
1.83	-0.00504	-0.04125	0.016889
1.84	-0.00501	-0.04124	0.016901
1.85	-0.00498	-0.04122	0.016913
1.86	-0.00495	-0.04121	0.016924
1.87	-0.00492	-0.0412	0.016937
1.88	-0.00488	-0.04119	0.016949
1.89	-0.00485	-0.04119	0.016963
1.9	-0.00481	-0.04118	0.016978

1.91	-0.00478	-0.04118	0.016993
1.92	-0.00474	-0.04118	0.017009
1.93	-0.00471	-0.04118	0.017026
1.94	-0.00467	-0.04118	0.017044
1.95	-0.00464	-0.04117	0.017064
1.96	-0.0046	-0.04117	0.017084
1.97	-0.00456	-0.04116	0.017105
1.98	-0.00453	-0.04115	0.017127
1.99	-0.0045	-0.04114	0.01715
2	-0.00446	-0.04112	0.017173
2.01	-0.00443	-0.04111	0.017197
2.02	-0.0044	-0.04109	0.017221
2.03	-0.00437	-0.04108	0.017245
2.04	-0.00434	-0.04106	0.017269
2.05	-0.00431	-0.04105	0.017292
2.06	-0.00428	-0.04103	0.017316
2.07	-0.00424	-0.04102	0.017338
2.08	-0.00421	-0.04101	0.017361
2.09	-0.00418	-0.041	0.017384
2.1	-0.00414	-0.04099	0.017407
2.11	-0.0041	-0.04098	0.017432
2.12	-0.00406	-0.04096	0.017458
2.13	-0.00401	-0.04095	0.017486
2.14	-0.00396	-0.04094	0.017516
2.15	-0.0039	-0.04092	0.017549
2.16	-0.00383	-0.0409	0.017586
2.17	-0.00375	-0.04088	0.017626
2.18	-0.00367	-0.04086	0.017673
2.19	-0.00357	-0.04083	0.017728
2.2	-0.00346	-0.04079	0.017796
2.21	-0.00333	-0.04075	0.017877
2.22	-0.0032	-0.04071	0.017975
2.23	-0.00305	-0.04065	0.018093
2.24	-0.00289	-0.04059	0.018233
2.25	-0.00272	-0.04052	0.018398
2.26	-0.00254	-0.04045	0.018591
2.27	-0.00235	-0.04036	0.018812
2.28	-0.00216	-0.04026	0.019056
2.29	-0.00195	-0.04015	0.019315
2.3	-0.00175	-0.04004	0.019583
2.31	-0.00154	-0.03992	0.019852
2.32	-0.00132	-0.03979	0.020115
2.33	-0.00111	-0.03966	0.020365
2.34	-0.00089	-0.03952	0.020594
2.35	-0.00068	-0.03938	0.020795
2.36	-0.00047	-0.03924	0.020962
2.37	-0.00027	-0.0391	0.021087
2.38	#######	-0.03895	0.021173

2.39	0.000101	-0.03881	0.021222
2.4	0.000253	-0.03867	0.021236
2.41	0.000396	-0.03854	0.021218
2.42	0.000527	-0.03841	0.021171
2.43	0.000645	-0.03828	0.021098
2.44	0.000749	-0.03817	0.021
2.45	0.000838	-0.03806	0.020881
2.46	0.000911	-0.03797	0.020743
2.47	0.000965	-0.03788	0.020589
2.48	0.000999	-0.03781	0.02042
2.49	0.001013	-0.03776	0.020239
2.5	0.001004	-0.03772	0.020048
2.51	0.000973	-0.03769	0.019848
2.52	0.00092	-0.03768	0.019641
2.53	0.00085	-0.03768	0.01943
2.54	0.000764	-0.03771	0.019216
2.55	0.000667	-0.03775	0.019
2.56	0.00056	-0.03781	0.018786
2.57	0.000447	-0.03789	0.018576
2.58	0.000331	-0.03799	0.018374
2.59	0.000213	-0.0381	0.018188
2.6	9.86E-05	-0.03822	0.018023
2.61	#######	-0.03835	0.017885
2.62	-0.00014	-0.03849	0.017781
2.63	-0.00026	-0.03863	0.017716
2.64	-0.00038	-0.03877	0.017697
2.65	-0.0005	-0.0389	0.01773
2.66	-0.00063	-0.03904	0.017821
2.67	-0.00076	-0.03916	0.017972
2.68	-0.00089	-0.03928	0.01817
2.69	-0.00103	-0.0394	0.018398
2.7	-0.00118	-0.03951	0.018638
2.71	-0.00134	-0.03962	0.018875
2.72	-0.00151	-0.03973	0.01909
2.73	-0.00172	-0.03984	0.019266
2.74	-0.00195	-0.03995	0.019387
2.75	-0.00222	-0.04007	0.019436
2.76	-0.00254	-0.04019	0.019395
2.77	-0.00291	-0.04032	0.019249
2.78	-0.00335	-0.04046	0.018995
2.79	-0.00385	-0.04062	0.01863
2.8	-0.00442	-0.0408	0.018153
2.81	-0.00508	-0.041	0.017561
2.82	-0.00582	-0.04122	0.016852
2.83	-0.00667	-0.04148	0.016025
2.84	-0.00761	-0.04178	0.015077
2.85	-0.00866	-0.04211	0.014007
2.86	-0.00984	-0.04249	0.012812

2.87	-0.01113	-0.04292	0.011493
2.88	-0.01256	-0.04341	0.010055
2.89	-0.01413	-0.04395	0.008507
2.9	-0.01584	-0.04455	0.006856
2.91	-0.01771	-0.04523	0.005111
2.92	-0.01973	-0.04597	0.00328
2.93	-0.02191	-0.04679	0.00137
2.94	-0.02427	-0.04769	-0.00061
2.95	-0.02679	-0.04868	-0.00265
2.96	-0.0295	-0.04975	-0.00475
2.97	-0.03238	-0.05092	-0.0069
2.98	-0.03545	-0.05219	-0.0091
2.99	-0.03872	-0.05355	-0.01138
3	-0.04218	-0.055	-0.01374
3.01	-0.04585	-0.05655	-0.0162
3.02	-0.04971	-0.05819	-0.01876
3.03	-0.05375	-0.05993	-0.02145
3.04	-0.05798	-0.06176	-0.02426
3.05	-0.06237	-0.06368	-0.02722
3.06	-0.06693	-0.06569	-0.03034
3.07	-0.07165	-0.0678	-0.03363
3.08	-0.07651	-0.07	-0.03707
3.09	-0.08152	-0.07229	-0.04068
3.1	-0.08665	-0.07469	-0.04443
3.11	-0.09192	-0.07718	-0.04831
3.12	-0.09729	-0.07977	-0.05234
3.13	-0.10278	-0.08246	-0.05649
3.14	-0.10835	-0.08525	-0.06075
3.15	-0.11401	-0.08815	-0.06513
3.16	-0.11975	-0.09115	-0.06961
3.17	-0.12555	-0.09425	-0.07419
3.18	-0.1314	-0.09746	-0.07884
3.19	-0.13729	-0.10076	-0.08355
3.2	-0.14322	-0.10415	-0.08828
3.21	-0.14917	-0.10761	-0.09303
3.22	-0.15512	-0.11116	-0.09778
3.23	-0.16107	-0.11477	-0.10249
3.24	-0.16699	-0.11844	-0.10715
3.25	-0.17286	-0.12217	-0.11173
3.26	-0.17867	-0.12596	-0.11623
3.27	-0.1844	-0.12978	-0.1206
3.28	-0.19003	-0.13364	-0.12485
3.29	-0.19556	-0.13752	-0.12895
3.3	-0.20095	-0.14142	-0.13288
3.31	-0.2062	-0.14532	-0.13664
3.32	-0.21129	-0.1492	-0.1402
3.33	-0.21621	-0.15308	-0.14354
3.34	-0.22094	-0.15692	-0.14666

3.35	-0.22548	-0.16072	-0.14953
3.36	-0.2298	-0.16448	-0.15214
3.37	-0.2339	-0.16817	-0.15447
3.38	-0.23777	-0.17179	-0.15653
3.39	-0.24138	-0.1753	-0.15831
3.4	-0.24473	-0.1787	-0.15982
3.41	-0.24781	-0.18197	-0.16105
3.42	-0.25061	-0.18508	-0.162
3.43	-0.25313	-0.18801	-0.16268
3.44	-0.25538	-0.19076	-0.16309
3.45	-0.25734	-0.19329	-0.16323
3.46	-0.25904	-0.19559	-0.16309
3.47	-0.26045	-0.19764	-0.16269
3.48	-0.26158	-0.19945	-0.16204
3.49	-0.26244	-0.201	-0.16119
3.5	-0.26301	-0.2023	-0.16017
3.51	-0.26331	-0.20334	-0.159
3.52	-0.26333	-0.20413	-0.15772
3.53	-0.26308	-0.20466	-0.15636
3.54	-0.26255	-0.20493	-0.15496
3.55	-0.26176	-0.20494	-0.15354
3.56	-0.2607	-0.20469	-0.15214
3.57	-0.25939	-0.20418	-0.15078
3.58	-0.25781	-0.20341	-0.14947
3.59	-0.25597	-0.20239	-0.1482
3.6	-0.25388	-0.20112	-0.14696
3.61	-0.25155	-0.1996	-0.14575
3.62	-0.24896	-0.19785	-0.14457
3.63	-0.24613	-0.19586	-0.14341
3.64	-0.24307	-0.19365	-0.14226
3.65	-0.23977	-0.19121	-0.14113
3.66	-0.23624	-0.18856	-0.14
3.67	-0.23248	-0.18569	-0.13887
3.68	-0.2285	-0.18261	-0.1377
3.69	-0.22431	-0.17933	-0.13646
3.7	-0.2199	-0.17584	-0.13511
3.71	-0.21527	-0.17214	-0.13362
3.72	-0.21042	-0.16825	-0.13194
3.73	-0.20533	-0.16416	-0.13005
3.74	-0.19998	-0.15988	-0.1279
3.75	-0.19435	-0.1554	-0.12547
3.76	-0.18843	-0.15074	-0.1227
3.77	-0.18221	-0.14589	-0.11958
3.78	-0.17566	-0.14086	-0.11611
3.79	-0.16877	-0.13565	-0.11227
3.8	-0.16152	-0.13028	-0.10809
3.81	-0.1539	-0.12475	-0.10356
3.82	-0.14591	-0.11907	-0.0987

3.83	-0.13755	-0.11324	-0.0935
3.84	-0.12882	-0.10727	-0.08797
3.85	-0.11972	-0.10117	-0.08211
3.86	-0.11026	-0.09493	-0.07594
3.87	-0.10043	-0.08858	-0.06944
3.88	-0.09023	-0.0821	-0.06264
3.89	-0.07968	-0.07549	-0.05554
3.9	-0.06876	-0.06874	-0.04815
3.91	-0.05748	-0.06184	-0.04047
3.92	-0.04583	-0.0548	-0.03251
3.93	-0.03381	-0.04761	-0.02429
3.94	-0.02142	-0.04025	-0.01579
3.95	-0.00864	-0.03273	-0.00705
3.96	0.003843	-0.02504	0.001948
3.97	0.015366	-0.01718	0.011186
3.98	0.027227	-0.00914	0.02067
3.99	0.039431	-0.00092	0.030407
4	0.051983	0.007467	0.040401
4.01	0.064883	0.01603	0.050659
4.02	0.078115	0.021052	0.061186
4.03	0.091661	0.028625	0.071987
4.04	0.1055	0.036345	0.08307
4.05	0.119613	0.044211	0.094439
4.06	0.133981	0.052222	0.1061
4.07	0.148584	0.060382	0.118058
4.08	0.163403	0.068704	0.130317
4.09	0.178417	0.077205	0.142879
4.1	0.193608	0.085904	0.155747
4.11	0.208959	0.094817	0.168923
4.12	0.224465	0.103961	0.18241
4.13	0.240125	0.113354	0.196212
4.14	0.255936	0.123012	0.21033
4.15	0.271897	0.132954	0.224767
4.16	0.288006	0.143196	0.239527
4.17	0.304261	0.15375	0.254606
4.18	0.320661	0.164606	0.269981
4.19	0.337203	0.175749	0.285625
4.2	0.353886	0.187162	0.30151
4.21	0.370706	0.19883	0.317606
4.22	0.387644	0.210737	0.333886
4.23	0.404683	0.222868	0.350323
4.24	0.421803	0.235207	0.366886
4.25	0.438983	0.247739	0.38355
4.26	0.456205	0.260448	0.400285
4.27	0.47345	0.273318	0.417066
4.28	0.490697	0.286338	0.433875
4.29	0.507927	0.299496	0.4507
4.3	0.525122	0.312781	0.467525

4.31	0.542262	0.32618	0.484337
4.32	0.559337	0.339682	0.501121
4.33	0.576338	0.353276	0.517863
4.34	0.593255	0.366949	0.534548
4.35	0.610079	0.380691	0.551163
4.36	0.6268	0.39449	0.567694
4.37	0.64341	0.408331	0.584127
4.38	0.659899	0.422196	0.60046
4.39	0.676257	0.436062	0.616691
4.4	0.692476	0.449908	0.632817
4.41	0.708544	0.463711	0.648838
4.42	0.724444	0.477449	0.664751
4.43	0.740158	0.491102	0.680555
4.44	0.755666	0.504646	0.696249
4.45	0.77095	0.51806	0.711829
4.46	0.785991	0.531323	0.727296
4.47	0.80077	0.544416	0.742642
4.48	0.815268	0.557339	0.757845
4.49	0.829466	0.570095	0.772877
4.5	0.843347	0.582688	0.78771
4.51	0.856891	0.595121	0.802318
4.52	0.870082	0.607399	0.816671
4.53	0.882905	0.619523	0.830745
4.54	0.895344	0.631499	0.844509
4.55	0.907383	0.643329	0.857938
4.56	0.919008	0.655018	0.871003
4.57	0.930202	0.666565	0.883676
4.58	0.940949	0.677967	0.895924
4.59	0.951235	0.689216	0.907713
4.6	0.961044	0.700305	0.91901
4.61	0.97036	0.711225	0.929779
4.62	0.97917	0.72197	0.939987
4.63	0.987462	0.732533	0.9496
4.64	0.995224	0.742906	0.958584
4.65	1.002442	0.753082	0.966904
4.66	1.009103	0.763053	0.974528
4.67	1.015196	0.772807	0.981427
4.68	1.020708	0.782307	0.987603
4.69	1.025626	0.791514	0.993064
4.7	1.029937	0.800384	0.997819
4.71	1.033631	0.808878	1.001875
4.72	1.036709	0.816954	1.00524
4.73	1.039173	0.824571	1.007923
4.74	1.041027	0.831687	1.009932
4.75	1.042272	0.838262	1.011274
4.76	1.042911	0.844253	1.011959
4.77	1.042948	0.849622	1.011991
4.78	1.042385	0.85433	1.011359

4.79	1.041225	0.858343	1.010053
4.8	1.03947	0.861625	1.008059
4.81	1.03712	0.864139	1.005364
4.82	1.034161	0.86585	1.001956
4.83	1.030577	0.866723	0.997822
4.84	1.02635	0.866722	0.992949
4.85	1.021464	0.86581	0.987325
4.86	1.015901	0.863952	0.980937
4.87	1.009645	0.861118	0.973776
4.88	1.002679	0.857297	0.965851
4.89	0.994985	0.852483	0.957175
4.9	0.986548	0.84667	0.947761
4.91	0.977347	0.839854	0.937622
4.92	0.967352	0.832028	0.92677
4.93	0.956533	0.823187	0.915219
4.94	0.944857	0.813325	0.90298
4.95	0.932292	0.802437	0.890068
4.96	0.918806	0.790518	0.876495
4.97	0.904368	0.777555	0.862247
4.98	0.888945	0.763518	0.847204
4.99	0.872506	0.748369	0.831221
5	0.855018	0.732069	0.814151
5.01	0.836435	0.714582	0.795847
5.02	0.816653	0.695869	0.776165
5.03	0.795554	0.675894	0.754956
5.04	0.773019	0.654618	0.732075
5.05	0.748929	0.632005	0.707376
5.06	0.723166	0.608016	0.680713
5.07	0.695612	0.582633	0.651986
5.08	0.666147	0.555917	0.621281
5.09	0.634654	0.527946	0.588733
5.1	0.601014	0.498799	0.554475
5.11	0.565157	0.468557	0.518641
5.12	0.527211	0.437298	0.481365
5.13	0.487355	0.405101	0.442779
5.14	0.445766	0.372046	0.403018
5.15	0.40262	0.338211	0.362215
5.16	0.358096	0.303677	0.320505
5.17	0.312372	0.268514	0.278024
5.18	0.265623	0.232763	0.23493
5.19	0.218029	0.196455	0.191386
5.2	0.169766	0.159622	0.147551
5.21	0.120998	0.122296	0.103588
5.22	0.071827	0.08451	0.059658
5.23	0.022345	0.046295	0.015924
5.24	-0.03219	0.007682	-0.02745
5.25	-0.09082	-0.03682	-0.07032
5.26	-0.14951	-0.08306	-0.1125

5.27	-0.20814	-0.12965	-0.15386
5.28	-0.26662	-0.17645	-0.19435
5.29	-0.32483	-0.22334	-0.23395
5.3	-0.38267	-0.27017	-0.27263
5.31	-0.44003	-0.31681	-0.31037
5.32	-0.49672	-0.36312	-0.34713
5.33	-0.55255	-0.40897	-0.38289
5.34	-0.60732	-0.45422	-0.41762
5.35	-0.66085	-0.49873	-0.4513
5.36	-0.71294	-0.54237	-0.48391
5.37	-0.7634	-0.58503	-0.51541
5.38	-0.81204	-0.62671	-0.54584
5.39	-0.85865	-0.66742	-0.57522
5.4	-0.90305	-0.7072	-0.60357
5.41	-0.94511	-0.74606	-0.63093
5.42	-0.98489	-0.78404	-0.65733
5.43	-1.02254	-0.82115	-0.68278
5.44	-1.05819	-0.85742	-0.70733
5.45	-1.09197	-0.89288	-0.73099
5.46	-1.12402	-0.92754	-0.75379
5.47	-1.15448	-0.96142	-0.77577
5.48	-1.18347	-0.99449	-0.79698
5.49	-1.21113	-1.0267	-0.81748
5.5	-1.23761	-1.05802	-0.83731
5.51	-1.26299	-1.0884	-0.85655
5.52	-1.28727	-1.11779	-0.87524
5.53	-1.31039	-1.14616	-0.89344
5.54	-1.33232	-1.17345	-0.9112
5.55	-1.353	-1.19964	-0.9286
5.56	-1.37239	-1.22466	-0.94567
5.57	-1.39043	-1.2485	-0.96246
5.58	-1.4071	-1.27113	-0.97892
5.59	-1.42232	-1.29256	-0.995
5.6	-1.43607	-1.31278	-1.01064
5.61	-1.4483	-1.33178	-1.02578
5.62	-1.45906	-1.34957	-1.04035
5.63	-1.46838	-1.36613	-1.05429
5.64	-1.4763	-1.38147	-1.06756
5.65	-1.48289	-1.39559	-1.08008
5.66	-1.48817	-1.40847	-1.09179
5.67	-1.4922	-1.42011	-1.10266
5.68	-1.49503	-1.43054	-1.11268
5.69	-1.4967	-1.43976	-1.12189
5.7	-1.49725	-1.44778	-1.13029
5.71	-1.49672	-1.45462	-1.13792
5.72	-1.49514	-1.4603	-1.14479
5.73	-1.49249	-1.46482	-1.15092
5.74	-1.4888	-1.4682	-1.15634

5.75	-1.48406	-1.47046	-1.16107
5.76	-1.47828	-1.47161	-1.16513
5.77	-1.47147	-1.47166	-1.16854
5.78	-1.46363	-1.47067	-1.17133
5.79	-1.45477	-1.46867	-1.17351
5.8	-1.4449	-1.46573	-1.1751
5.81	-1.43403	-1.46189	-1.17613
5.82	-1.42224	-1.45719	-1.17662
5.83	-1.40961	-1.4517	-1.17658
5.84	-1.39621	-1.44545	-1.17605
5.85	-1.38215	-1.4385	-1.17503
5.86	-1.36749	-1.4309	-1.17356
5.87	-1.35234	-1.42269	-1.17165
5.88	-1.33675	-1.41388	-1.16929
5.89	-1.32083	-1.4045	-1.16648
5.9	-1.30466	-1.39455	-1.16321
5.91	-1.2883	-1.38404	-1.15948
5.92	-1.27176	-1.37299	-1.15527
5.93	-1.25501	-1.36141	-1.15059
5.94	-1.23804	-1.34931	-1.14541
5.95	-1.22084	-1.3367	-1.13975
5.96	-1.20339	-1.3236	-1.13358
5.97	-1.18568	-1.31001	-1.1269
5.98	-1.16768	-1.29592	-1.11974
5.99	-1.14938	-1.28133	-1.11209
6	-1.13077	-1.26622	-1.10399
6.01	-1.11183	-1.25058	-1.09544
6.02	-1.09259	-1.2344	-1.08645
6.03	-1.07306	-1.21768	-1.07705
6.04	-1.05327	-1.20039	-1.06725
6.05	-1.03324	-1.18253	-1.05706
6.06	-1.01299	-1.16408	-1.04651
6.07	-0.99255	-1.14505	-1.0356
6.08	-0.97193	-1.12545	-1.02431
6.09	-0.95117	-1.10529	-1.01265
6.1	-0.93028	-1.08461	-1.0006
6.11	-0.90928	-1.06343	-0.98815
6.12	-0.88821	-1.04177	-0.97527
6.13	-0.86708	-1.01965	-0.96197
6.14	-0.84591	-0.99709	-0.94823
6.15	-0.82473	-0.97412	-0.93403
6.16	-0.80357	-0.95076	-0.91936
6.17	-0.78244	-0.92704	-0.90423
6.18	-0.76136	-0.90301	-0.88869
6.19	-0.74037	-0.87872	-0.8728
6.2	-0.71949	-0.85422	-0.85664
6.21	-0.69872	-0.82957	-0.84028
6.22	-0.67806	-0.80483	-0.82378

6.23	-0.65746	-0.78004	-0.80721
6.24	-0.63689	-0.75527	-0.79064
6.25	-0.61634	-0.73056	-0.77415
6.26	-0.59576	-0.70597	-0.75779
6.27	-0.57513	-0.68154	-0.74159
6.28	-0.55442	-0.65727	-0.72542
6.29	-0.53359	-0.63315	-0.70908
6.3	-0.51263	-0.60916	-0.69238
6.31	-0.49148	-0.58528	-0.67515
6.32	-0.47007	-0.5615	-0.6572
6.33	-0.44833	-0.53781	-0.63833
6.34	-0.42618	-0.51419	-0.61836
6.35	-0.40352	-0.49063	-0.59711
6.36	-0.38029	-0.46711	-0.57438
6.37	-0.3564	-0.44361	-0.55004
6.38	-0.33177	-0.42005	-0.52411
6.39	-0.30632	-0.39636	-0.49664
6.4	-0.27997	-0.37246	-0.4677
6.41	-0.25264	-0.34825	-0.43736
6.42	-0.22426	-0.32366	-0.40568
6.43	-0.19479	-0.29861	-0.37272
6.44	-0.16415	-0.27302	-0.33853
6.45	-0.13229	-0.24679	-0.3032
6.46	-0.09916	-0.21986	-0.26678
6.47	-0.06468	-0.19214	-0.22933
6.48	-0.02881	-0.16358	-0.19098
6.49	0.007244	-0.13414	-0.15183
6.5	0.040264	-0.10375	-0.11201
6.51	0.074606	-0.07239	-0.07163
6.52	0.110238	-0.03999	-0.0308
6.53	0.147109	-0.00651	0.010344
6.54	0.185168	0.028094	0.051702
6.55	0.224366	0.054295	0.09315
6.56	0.264653	0.085752	0.134573
6.57	0.305978	0.118276	0.175866
6.58	0.348292	0.151837	0.216984
6.59	0.391543	0.186389	0.257895
6.6	0.435682	0.221887	0.298565
6.61	0.480646	0.258286	0.338964
6.62	0.526323	0.295541	0.379059
6.63	0.572589	0.333605	0.418817
6.64	0.61932	0.372435	0.458206
6.65	0.66639	0.411984	0.497194
6.66	0.713676	0.452207	0.535749
6.67	0.761053	0.49305	0.573846
6.68	0.808396	0.534422	0.611482
6.69	0.855582	0.576221	0.648665
6.7	0.902486	0.618347	0.685399

6.71	0.948991	0.660699	0.721691
6.72	0.995016	0.703177	0.757547
6.73	1.040487	0.74568	0.792972
6.74	1.08533	0.788107	0.827972
6.75	1.129472	0.830357	0.862553
6.76	1.172838	0.872331	0.89672
6.77	1.215356	0.913931	0.930468
6.78	1.256951	0.95508	0.963743
6.79	1.297549	0.995705	0.996479
6.8	1.337078	1.035732	1.028611
6.81	1.375457	1.075088	1.060074
6.82	1.412581	1.113699	1.090801
6.83	1.44834	1.151494	1.120728
6.84	1.482623	1.188397	1.149789
6.85	1.515319	1.224336	1.177918
6.86	1.546319	1.259238	1.20505
6.87	1.57551	1.293035	1.231116
6.88	1.602782	1.325683	1.256037
6.89	1.628025	1.357145	1.279728
6.9	1.651128	1.387383	1.302106
6.91	1.67199	1.416359	1.323088
6.92	1.690547	1.444036	1.34259
6.93	1.706745	1.470375	1.36053
6.94	1.720531	1.495339	1.376822
6.95	1.731851	1.51889	1.391385
6.96	1.740651	1.54099	1.404135
6.97	1.746878	1.5616	1.415003
6.98	1.750476	1.580676	1.42398
6.99	1.751394	1.598173	1.431074
7	1.749576	1.614045	1.436289
7.01	1.744979	1.628246	1.439633
7.02	1.737597	1.640731	1.441112
7.03	1.727436	1.651456	1.440732
7.04	1.714499	1.660373	1.438499
7.05	1.698792	1.667438	1.43442
7.06	1.680319	1.672605	1.428501
7.07	1.659084	1.675838	1.420751
7.08	1.635092	1.677139	1.41119
7.09	1.608349	1.676521	1.399842
7.1	1.578858	1.673993	1.38673
7.11	1.546651	1.669569	1.371877
7.12	1.51187	1.663258	1.355306
7.13	1.474685	1.655074	1.337041
7.14	1.435265	1.645026	1.317105
7.15	1.393778	1.633128	1.295521
7.16	1.350394	1.619389	1.272312
7.17	1.305283	1.603816	1.24752
7.18	1.258612	1.586386	1.221257

7.19	1.210553	1.567069	1.193653
7.2	1.161273	1.545838	1.164839
7.21	1.110959	1.522663	1.134947
7.22	1.059866	1.497515	1.104106
7.23	1.008263	1.470365	1.072447
7.24	0.956422	1.441185	1.0401
7.25	0.904614	1.409945	1.007197
7.26	0.85311	1.376616	0.973868
7.27	0.80218	1.341205	0.940256
7.28	0.752097	1.303854	0.906558
7.29	0.70313	1.264742	0.872984
7.3	0.655551	1.224047	0.839744
7.31	0.609593	1.181948	0.807047
7.32	0.565332	1.138622	0.775104
7.33	0.522808	1.094248	0.744124
7.34	0.48206	1.049003	0.714316
7.35	0.443127	1.003067	0.685892
7.36	0.406048	0.956617	0.659059
7.37	0.370862	0.909827	0.634016
7.38	0.337609	0.862862	0.610903
7.39	0.306327	0.815881	0.589851
7.4	0.277055	0.769043	0.570986
7.41	0.249834	0.722509	0.554439
7.42	0.224709	0.676437	0.540339
7.43	0.201726	0.630987	0.528813
7.44	0.180931	0.58632	0.51999
7.45	0.162371	0.542594	0.514
7.46	0.146091	0.49997	0.510971
7.47	0.132139	0.458595	0.510938
7.48	0.120559	0.418564	0.51356
7.49	0.111399	0.379962	0.518403
7.5	0.104705	0.342871	0.525031
7.51	0.100458	0.307377	0.533011
7.52	0.09838	0.273561	0.541908
7.53	0.098126	0.241508	0.551287
7.54	0.099353	0.211301	0.560713
7.55	0.101719	0.183024	0.569753
7.56	0.104878	0.156761	0.57797
7.57	0.108489	0.132553	0.58494
7.58	0.112206	0.110274	0.590275
7.59	0.115687	0.089759	0.593597
7.6	0.118587	0.070841	0.594526
7.61	0.120617	0.053351	0.592683
7.62	0.121695	0.037123	0.587692
7.63	0.121794	0.021991	0.579171
7.64	0.120886	0.007787	0.566743
7.65	0.118941	-0.00665	0.55003
7.66	0.115934	-0.02177	0.528652

7.67	0.111835	-0.03637	0.502377
7.68	0.106617	-0.05062	0.471559
7.69	0.100251	-0.06467	0.436695
7.7	0.09271	-0.07866	0.398285
7.71	0.083943	-0.09274	0.356829
7.72	0.073802	-0.10707	0.312825
7.73	0.062117	-0.12179	0.266773
7.74	0.048719	-0.13705	0.219171
7.75	0.033436	-0.153	0.170519
7.76	0.016099	-0.16979	0.121315
7.77	-0.00407	-0.18754	0.071993
7.78	-0.0299	-0.20629	0.02272
7.79	-0.05875	-0.22605	-0.0264
7.8	-0.09082	-0.24682	-0.07528
7.81	-0.12622	-0.26863	-0.1238
7.82	-0.16472	-0.29147	-0.17187
7.83	-0.20604	-0.31535	-0.21939
7.84	-0.24985	-0.3403	-0.26626
7.85	-0.29585	-0.36631	-0.31238
7.86	-0.34373	-0.3934	-0.35764
7.87	-0.39319	-0.42157	-0.40196
7.88	-0.44391	-0.45081	-0.44537
7.89	-0.4956	-0.48112	-0.48788
7.9	-0.54794	-0.51249	-0.52952
7.91	-0.60067	-0.5449	-0.57034
7.92	-0.65368	-0.57835	-0.61035
7.93	-0.7069	-0.61281	-0.64959
7.94	-0.76028	-0.6483	-0.68809
7.95	-0.81375	-0.68478	-0.72588
7.96	-0.86725	-0.72226	-0.76299
7.97	-0.9207	-0.76069	-0.79944
7.98	-0.97405	-0.79993	-0.83521
7.99	-1.02723	-0.83981	-0.87029
8	-1.08017	-0.88016	-0.90463
8.01	-1.13279	-0.92081	-0.93821
8.02	-1.18487	-0.96159	-0.97101
8.03	-1.23617	-1.00232	-1.003
8.04	-1.28645	-1.04284	-1.03415
8.05	-1.33548	-1.08297	-1.06444
8.06	-1.38301	-1.12255	-1.09384
8.07	-1.42881	-1.16143	-1.12232
8.08	-1.47264	-1.19956	-1.14985
8.09	-1.51425	-1.23694	-1.17642
8.1	-1.5534	-1.27355	-1.202
8.11	-1.58992	-1.30937	-1.22655
8.12	-1.62381	-1.3444	-1.25007
8.13	-1.65514	-1.37861	-1.27252
8.14	-1.68398	-1.41199	-1.29387

8.15	-1.71039	-1.44452	-1.31411
8.16	-1.73445	-1.4762	-1.33321
8.17	-1.7562	-1.50699	-1.35115
8.18	-1.77573	-1.53681	-1.36791
8.19	-1.7931	-1.56558	-1.38348
8.2	-1.80837	-1.5932	-1.39787
8.21	-1.82161	-1.61957	-1.41106
8.22	-1.83281	-1.64461	-1.42304
8.23	-1.84198	-1.66822	-1.4338
8.24	-1.84912	-1.6903	-1.44334
8.25	-1.85423	-1.71078	-1.45165
8.26	-1.8573	-1.72955	-1.45872
8.27	-1.85834	-1.74655	-1.46455
8.28	-1.85733	-1.76184	-1.46915
8.29	-1.85429	-1.77548	-1.47254
8.3	-1.84921	-1.78758	-1.47476
8.31	-1.84211	-1.7982	-1.47582
8.32	-1.83309	-1.80742	-1.47575
8.33	-1.82229	-1.81533	-1.47457
8.34	-1.80982	-1.82201	-1.4723
8.35	-1.79583	-1.82754	-1.46896
8.36	-1.78043	-1.832	-1.46458
8.37	-1.76376	-1.83544	-1.45919
8.38	-1.74593	-1.8378	-1.45278
8.39	-1.72708	-1.83896	-1.44538
8.4	-1.70734	-1.83884	-1.437
8.41	-1.68681	-1.83734	-1.42766
8.42	-1.66553	-1.83436	-1.41735
8.43	-1.64351	-1.8298	-1.4061
8.44	-1.62075	-1.82355	-1.39392
8.45	-1.59728	-1.81553	-1.38081
8.46	-1.57311	-1.80564	-1.3668
8.47	-1.54825	-1.79379	-1.35191
8.48	-1.52271	-1.77997	-1.33619
8.49	-1.4965	-1.7642	-1.31972
8.5	-1.46964	-1.74649	-1.30256
8.51	-1.44216	-1.72685	-1.2848
8.52	-1.4141	-1.70528	-1.2665
8.53	-1.38554	-1.68181	-1.24772
8.54	-1.35654	-1.65644	-1.22856
8.55	-1.32718	-1.62918	-1.20906
8.56	-1.29752	-1.60004	-1.18931
8.57	-1.26762	-1.56906	-1.16939
8.58	-1.23757	-1.53634	-1.14936
8.59	-1.20742	-1.50203	-1.12933
8.6	-1.17724	-1.46625	-1.10937
8.61	-1.14709	-1.42913	-1.08957
8.62	-1.11703	-1.39082	-1.07003

8.63	-1.08709	-1.35144	-1.05082
8.64	-1.05732	-1.31113	-1.03204
8.65	-1.02775	-1.27003	-1.01377
8.66	-0.99843	-1.22826	-0.9961
8.67	-0.9694	-1.18596	-0.97909
8.68	-0.94069	-1.14331	-0.96276
8.69	-0.91236	-1.10048	-0.9471
8.7	-0.88444	-1.05764	-0.93211
8.71	-0.85696	-1.01497	-0.91777
8.72	-0.82989	-0.97264	-0.90409
8.73	-0.80322	-0.93083	-0.89105
8.74	-0.7769	-0.88971	-0.87864
8.75	-0.7509	-0.84945	-0.86687
8.76	-0.72519	-0.81024	-0.85572
8.77	-0.69973	-0.7722	-0.84513
8.78	-0.6745	-0.73536	-0.83482
8.79	-0.64945	-0.69967	-0.82445
8.8	-0.62456	-0.66513	-0.81367
8.81	-0.59978	-0.6317	-0.80216
8.82	-0.57505	-0.59936	-0.78956
8.83	-0.55028	-0.56809	-0.77555
8.84	-0.52539	-0.53785	-0.75977
8.85	-0.5003	-0.50863	-0.7419
8.86	-0.47494	-0.48039	-0.72159
8.87	-0.44921	-0.4531	-0.69857
8.88	-0.42305	-0.42659	-0.67288
8.89	-0.39637	-0.4007	-0.64461
8.9	-0.3691	-0.37526	-0.61387
8.91	-0.34113	-0.35009	-0.58075
8.92	-0.31233	-0.32503	-0.54537
8.93	-0.28254	-0.29989	-0.50781
8.94	-0.25161	-0.27451	-0.46818
8.95	-0.21939	-0.24872	-0.42659
8.96	-0.18572	-0.22233	-0.38313
8.97	-0.15046	-0.1952	-0.33792
8.98	-0.11343	-0.1672	-0.29115
8.99	-0.0745	-0.13821	-0.24301
9	-0.03351	-0.10812	-0.19371
9.01	0.008203	-0.07683	-0.14345
9.02	0.046684	-0.04421	-0.09243
9.03	0.086866	-0.01016	-0.04085
9.04	0.128658	0.025444	0.011094
9.05	0.171969	0.062702	0.063193
9.06	0.216709	0.101732	0.115249
9.07	0.262786	0.121225	0.167083
9.08	0.31011	0.157529	0.218598
9.09	0.35859	0.195332	0.269716
9.1	0.408134	0.234585	0.320364

9.11	0.458651	0.275237	0.370463
9.12	0.510044	0.317236	0.419939
9.13	0.562214	0.360533	0.468714
9.14	0.615065	0.405076	0.516714
9.15	0.668497	0.450815	0.563861
9.16	0.722413	0.497699	0.61008
9.17	0.776714	0.545657	0.655307
9.18	0.831303	0.59453	0.699525
9.19	0.886082	0.644142	0.742729
9.2	0.940952	0.694314	0.784915
9.21	0.995802	0.744868	0.826079
9.22	1.050467	0.795626	0.866215
9.23	1.104769	0.846411	0.905319
9.24	1.158527	0.897043	0.943387
9.25	1.211564	0.947346	0.980414
9.26	1.263701	0.997141	1.016395
9.27	1.31476	1.046263	1.05133
9.28	1.364561	1.094607	1.085235
9.29	1.412925	1.14208	1.11813
9.3	1.459675	1.188588	1.150036
9.31	1.504648	1.234039	1.180972
9.32	1.547757	1.27834	1.21096
9.33	1.588931	1.321398	1.24002
9.34	1.628098	1.363121	1.268171
9.35	1.665187	1.403415	1.295434
9.36	1.700128	1.442189	1.321829
9.37	1.73285	1.479361	1.347354
9.38	1.763281	1.514901	1.371913
9.39	1.791352	1.54879	1.395386
9.4	1.81699	1.581011	1.417654
9.41	1.840118	1.611544	1.438599
9.42	1.860634	1.640371	1.458101
9.43	1.878426	1.667475	1.476042
9.44	1.893385	1.692836	1.492302
9.45	1.905402	1.716437	1.506763
9.46	1.914365	1.738258	1.519304
9.47	1.920165	1.758279	1.529824
9.48	1.922692	1.776468	1.538282
9.49	1.921836	1.792791	1.544654
9.5	1.917487	1.807214	1.548917
9.51	1.90956	1.819703	1.551046
9.52	1.898074	1.830223	1.551019
9.53	1.88307	1.838741	1.548809
9.54	1.864592	1.845221	1.544395
9.55	1.842684	1.84963	1.537752
9.56	1.817387	1.851934	1.528855
9.57	1.788746	1.852107	1.517699
9.58	1.756803	1.850161	1.504344

9.59	1.7216	1.846117	1.488867
9.6	1.683182	1.839995	1.471348
9.61	1.641623	1.831816	1.451862
9.62	1.597126	1.821601	1.430489
9.63	1.549927	1.80937	1.407306
9.64	1.500259	1.795145	1.382391
9.65	1.448358	1.778945	1.355822
9.66	1.39446	1.760791	1.327676
9.67	1.338798	1.740696	1.298037
9.68	1.281608	1.718638	1.267006
9.69	1.223125	1.694587	1.234692
9.7	1.163584	1.668512	1.201201
9.71	1.103231	1.640385	1.16664
9.72	1.042363	1.610175	1.131118
9.73	0.981285	1.577851	1.094741
9.74	0.920305	1.543385	1.057616
9.75	0.85973	1.506745	1.019851
9.76	0.799868	1.467903	0.981554
9.77	0.741024	1.426869	0.942867
9.78	0.683506	1.383825	0.904082
9.79	0.627621	1.338994	0.865523
9.8	0.573677	1.292599	0.827518
9.81	0.521933	1.244863	0.790392
9.82	0.472467	1.196009	0.754472
9.83	0.425307	1.14626	0.720085
9.84	0.380484	1.09584	0.687556
9.85	0.338028	1.04497	0.657211
9.86	0.297968	0.993875	0.629378
9.87	0.260334	0.94276	0.604312
9.88	0.225156	0.891763	0.581996
9.89	0.192464	0.841004	0.562339
9.9	0.162288	0.790604	0.545254
9.91	0.134647	0.740682	0.530651
9.92	0.109526	0.69136	0.518442
9.93	0.086896	0.642758	0.508538
9.94	0.066732	0.594996	0.500851
9.95	0.049006	0.548195	0.495291
9.96	0.033693	0.502474	0.491771
9.97	0.020764	0.457949	0.490162
9.98	0.010195	0.414712	0.490177
9.99	0.001957	0.372849	0.491493
10	-0.00468	0.332446	0.493784
10.01	-0.00903	0.29359	0.496723
10.02	-0.01103	0.256366	0.499987
10.03	-0.01103	0.220862	0.503249
10.04	-0.00935	0.187162	0.506185
10.05	-0.00633	0.155355	0.508468
10.06	-0.0023	0.147677	0.509775

10.07	0.002035	0.114971	0.509827
10.08	0.00631	0.084557	0.508543
10.09	0.010583	0.056297	0.505889
10.1	0.014571	0.030052	0.501831
10.11	0.01804	0.005687	0.496335
10.12	0.020965	-0.01694	0.489367
10.13	0.023371	-0.03796	0.480894
10.14	0.025285	-0.05751	0.470882
10.15	0.026733	-0.07574	0.459297
10.16	0.027741	-0.09277	0.446105
10.17	0.028335	-0.10874	0.431288
10.18	0.028541	-0.12376	0.414887
10.19	0.028386	-0.13793	0.396959
10.2	0.027895	-0.15136	0.377563
10.21	0.027057	-0.16415	0.356754
10.22	0.025709	-0.1764	0.334589
10.23	0.02365	-0.18821	0.311127
10.24	0.02068	-0.19969	0.286423
10.25	0.016597	-0.21094	0.260536
10.26	0.011202	-0.22206	0.233521
10.27	0.004294	-0.23316	0.205443
10.28	-0.00509	-0.2443	0.176388
10.29	-0.01749	-0.25554	0.14645
10.3	-0.03238	-0.26697	0.115722
10.31	-0.04991	-0.27863	0.084298
10.32	-0.06997	-0.29061	0.05227
10.33	-0.09233	-0.30295	0.019732
10.34	-0.11679	-0.31574	-0.01322
10.35	-0.14314	-0.32903	-0.0465
10.36	-0.17117	-0.34289	-0.08001
10.37	-0.20068	-0.35739	-0.11366
10.38	-0.23145	-0.37255	-0.1474
10.39	-0.26328	-0.38841	-0.18118
10.4	-0.29596	-0.40499	-0.21496
10.41	-0.32931	-0.42232	-0.24867
10.42	-0.36329	-0.44043	-0.28228
10.43	-0.39789	-0.45935	-0.31574
10.44	-0.43311	-0.47911	-0.34899
10.45	-0.46893	-0.49974	-0.382
10.46	-0.50534	-0.52126	-0.4147
10.47	-0.54234	-0.54368	-0.44706
10.48	-0.57992	-0.56697	-0.47903
10.49	-0.61806	-0.59106	-0.51057
10.5	-0.65676	-0.6159	-0.54164
10.51	-0.69599	-0.64141	-0.57218
10.52	-0.73559	-0.66755	-0.60217
10.53	-0.77541	-0.69425	-0.63156
10.54	-0.81527	-0.72146	-0.66031

10.55	-0.85501	-0.7491	-0.68837
10.56	-0.89446	-0.77713	-0.71571
10.57	-0.93345	-0.80548	-0.74229
10.58	-0.97181	-0.83404	-0.76812
10.59	-1.00937	-0.86272	-0.79324
10.6	-1.04597	-0.89141	-0.81766
10.61	-1.08146	-0.92001	-0.84142
10.62	-1.11575	-0.94841	-0.86454
10.63	-1.14878	-0.97652	-0.88705
10.64	-1.1805	-1.00422	-0.90897
10.65	-1.21084	-1.03142	-0.93033
10.66	-1.23975	-1.05802	-0.95115
10.67	-1.26716	-1.08392	-0.97145
10.68	-1.29301	-1.1091	-0.99116
10.69	-1.31724	-1.13355	-1.01022
10.7	-1.33979	-1.15728	-1.02856
10.71	-1.36062	-1.18026	-1.0461
10.72	-1.37973	-1.2025	-1.06276
10.73	-1.39717	-1.22398	-1.07849
10.74	-1.41295	-1.2447	-1.0932
10.75	-1.42711	-1.26466	-1.10682
10.76	-1.43968	-1.28384	-1.11929
10.77	-1.45069	-1.30224	-1.13054
10.78	-1.46017	-1.31984	-1.14064
10.79	-1.46815	-1.33664	-1.14965
10.8	-1.47466	-1.35261	-1.15763
10.81	-1.47972	-1.36774	-1.16466
10.82	-1.48335	-1.38203	-1.17081
10.83	-1.48556	-1.39545	-1.17615
10.84	-1.48636	-1.40799	-1.18075
10.85	-1.48575	-1.41965	-1.18468
10.86	-1.48375	-1.43039	-1.188
10.87	-1.48037	-1.44022	-1.19078
10.88	-1.47561	-1.44912	-1.19301
10.89	-1.46949	-1.45709	-1.19466
10.9	-1.46201	-1.46412	-1.19572
10.91	-1.45319	-1.47019	-1.19616
10.92	-1.44311	-1.47531	-1.19597
10.93	-1.43185	-1.47946	-1.19512
10.94	-1.41949	-1.48264	-1.1936
10.95	-1.40612	-1.48484	-1.19138
10.96	-1.3918	-1.48605	-1.18844
10.97	-1.37663	-1.48624	-1.18476
10.98	-1.3607	-1.48536	-1.1803
10.99	-1.34407	-1.48333	-1.17501
11	-1.32683	-1.48005	-1.16886
11.01	-1.30906	-1.47546	-1.16181
11.02	-1.29074	-1.46947	-1.1538

11.03	-1.27185	-1.462	-1.14481
11.04	-1.25238	-1.45298	-1.13478
11.05	-1.2323	-1.44232	-1.12368
11.06	-1.2116	-1.42993	-1.11146
11.07	-1.19025	-1.41577	-1.09808
11.08	-1.16823	-1.39981	-1.08342
11.09	-1.14553	-1.38206	-1.06738
11.1	-1.12212	-1.36251	-1.04986
11.11	-1.09797	-1.34118	-1.03072
11.12	-1.07303	-1.31807	-1.00988
11.13	-1.04722	-1.29317	-0.9872
11.14	-1.02048	-1.2665	-0.96259
11.15	-0.99274	-1.23805	-0.93593
11.16	-0.96392	-1.20783	-0.9071
11.17	-0.93396	-1.17584	-0.87605
11.18	-0.90279	-1.14214	-0.84285
11.19	-0.87034	-1.10677	-0.80765
11.2	-0.83653	-1.06978	-0.77058
11.21	-0.80133	-1.03124	-0.73176
11.22	-0.76475	-0.99119	-0.69134
11.23	-0.72684	-0.94968	-0.64944
11.24	-0.68763	-0.90677	-0.6062
11.25	-0.64716	-0.86251	-0.56174
11.26	-0.60549	-0.81695	-0.51622
11.27	-0.56264	-0.77015	-0.46978
11.28	-0.51865	-0.72221	-0.42272
11.29	-0.47358	-0.67325	-0.37538
11.3	-0.42747	-0.62335	-0.32808
11.31	-0.38034	-0.57264	-0.28115
11.32	-0.33228	-0.52121	-0.23493
11.33	-0.28335	-0.46919	-0.18973
11.34	-0.23361	-0.41666	-0.14589
11.35	-0.18314	-0.36374	-0.10374
11.36	-0.13201	-0.31054	-0.0636
11.37	-0.08027	-0.25717	-0.02576
11.38	-0.02801	-0.20378	0.009686
11.39	0.021011	-0.15056	0.04269
11.4	0.013233	-0.09765	0.073205
11.41	0.022311	-0.04523	0.101183
11.42	0.031391	0.006532	0.126574
11.43	0.040418	0.057479	0.149331
11.44	0.049337	0.10744	0.169405
11.45	0.058094	0.132813	0.186748
11.46	0.066636	0.034636	0.040262
11.47	0.074907	0.042459	0.042616
11.48	0.082855	0.050012	0.044434
11.49	0.090423	0.057277	0.045747
11.5	0.097558	0.064235	0.046584

11.51	0.104211	0.070868	0.046975
11.52	0.110347	0.077159	0.04695
11.53	0.115938	0.083089	0.046539
11.54	0.120953	0.08864	0.045772
11.55	0.125364	0.093794	0.044679
11.56	0.129141	0.098533	0.043289
11.57	0.132255	0.102843	0.041635
11.58	0.134677	0.106726	0.039755
11.59	0.136377	0.110189	0.037689
11.6	0.137326	0.113237	0.035479
11.61	0.137505	0.115876	0.033164
11.62	0.136927	0.118114	0.030784
11.63	0.13562	0.119956	0.028381
11.64	0.133607	0.121409	0.025995
11.65	0.130914	0.122478	0.023666
11.66	0.127566	0.12317	0.021434
11.67	0.123588	0.123494	0.019334
11.68	0.119004	0.123469	0.017367
11.69	0.11384	0.123114	0.015531
11.7	0.10812	0.122452	0.013821
11.71	0.101875	0.121503	0.012234
11.72	0.095148	0.120289	0.010766
11.73	0.087991	0.118829	0.009413
11.74	0.080451	0.117147	0.008171
11.75	0.072579	0.115261	0.007037
11.76	0.064425	0.113194	0.006006
11.77	0.056037	0.110967	0.005074
11.78	0.047466	0.108604	0.004236
11.79	0.03876	0.106129	0.003486
11.8	0.02997	0.103566	0.002817
11.81	0.021147	0.10094	0.002225
11.82	0.01235	0.098275	0.001702
11.83	0.003642	0.095595	0.001243
11.84	-0.00578	0.092925	0.000841
11.85	-0.0156	0.090289	0.000492
11.86	-0.0251	0.087711	0.000188
11.87	-0.0342	0.085211	#######
11.88	-0.04283	0.08279	-0.0003
11.89	-0.05093	0.080442	-0.0005
11.9	-0.05841	0.091959	-0.00066
11.91	-0.06522	0.089356	-0.0008
11.92	-0.07136	0.086826	-0.00091
11.93	-0.07686	0.084362	-0.001
11.94	-0.08174	0.08196	-0.00107
11.95	-0.08602	0.079616	-0.00113
11.96	-0.08971	0.077323	-0.00118
11.97	-0.09285	0.075077	-0.00123
11.98	-0.09545	0.072872	-0.00126

11.99	-0.09753	0.070706	-0.00128
12	-0.09912	0.068571	-0.0013
12.01	-0.10024	0.066465	-0.0013
12.02	-0.1009	0.064381	-0.0013
12.03	-0.10112	0.062316	-0.00128
12.04	-0.10094	0.060264	-0.00125
12.05	-0.10037	0.058221	-0.00121
12.06	-0.09942	0.056182	-0.00116
12.07	-0.09813	0.054143	-0.00109
12.08	-0.09651	0.0521	-0.00102
12.09	-0.09458	0.05005	-0.00093
12.1	-0.09237	0.047989	-0.00083
12.11	-0.08989	0.045916	-0.00073
12.12	-0.08718	0.043826	-0.00063
12.13	-0.08425	0.041717	-0.00052
12.14	-0.08113	0.039585	-0.00042
12.15	-0.07786	0.037427	-0.00031
12.16	-0.07445	0.035241	-0.00021
12.17	-0.07094	0.033025	-0.00011
12.18	-0.06734	0.030789	#######
12.19	-0.06369	0.028547	6.17E-05
12.2	-0.06002	0.026311	0.000141
12.21	-0.05634	0.024094	0.000215
12.22	-0.05268	0.021908	0.000283
12.23	-0.04905	0.019766	0.000344
12.24	-0.04549	0.01768	0.000399
12.25	-0.042	0.015664	0.000447
12.26	-0.03862	0.01373	0.000489
12.27	-0.03535	0.011888	0.000523
12.28	-0.03221	0.010142	0.000552
12.29	-0.02924	0.008494	0.000575
12.3	-0.02644	0.006944	0.000593
12.31	-0.02384	0.005494	0.000609
12.32	-0.02141	0.004145	0.000621
12.33	-0.01916	0.002899	0.000631
12.34	-0.01706	0.001757	0.000641
12.35	-0.01511	0.00072	0.00065
12.36	-0.01328	-0.00021	0.000659
12.37	-0.01156	-0.00103	0.00067
12.38	-0.00994	-0.00175	0.000683
12.39	-0.0084	-0.00238	0.000697
12.4	-0.00693	-0.00291	0.000714
12.41	-0.00552	-0.00336	0.000733
12.42	-0.00417	-0.00372	0.000754
12.43	-0.0029	-0.00401	0.000778
12.44	-0.00172	-0.00423	0.000805
12.45	-0.00064	-0.00439	0.000835
12.46	0.000289	-0.00448	0.000868

12.47	0.00102	-0.00452	0.000905
12.48	0.001641	-0.00452	0.000944
12.49	0.002143	-0.00447	0.000985
12.5	0.002519	-0.00439	0.001027
12.51	0.002762	-0.00428	0.001069
12.52	0.002885	-0.00415	0.00111
12.53	0.002905	-0.00401	0.00115
12.54	0.002838	-0.00386	0.001187
12.55	0.002701	-0.00371	0.001221
12.56	0.002509	-0.00356	0.001251
12.57	0.002281	-0.00343	0.001275
12.58	0.002031	-0.00331	0.001295
12.59	0.001777	-0.00321	0.001309
12.6	0.001535	-0.00312	0.001319
12.61	0.001318	-0.00306	0.001323
12.62	0.001125	-0.00301	0.001323
12.63	0.000951	-0.00299	0.001317
12.64	0.000792	-0.003	0.001307
12.65	0.000642	-0.00303	0.001291
12.66	0.000499	-0.00309	0.001271
12.67	0.000356	-0.00318	0.001246
12.68	0.000209	-0.0033	0.001216
12.69	5.37E-05	-0.00344	0.001184
12.7	-0.00013	-0.00361	0.001149
12.71	-0.00035	-0.0038	0.001113
12.72	-0.00059	-0.00402	0.001077
12.73	-0.00083	-0.00425	0.00104
12.74	-0.00109	-0.00449	0.001005
12.75	-0.00136	-0.00475	0.000973
12.76	-0.00162	-0.00503	0.000943
12.77	-0.00189	-0.00531	0.000917
12.78	-0.00215	-0.0056	0.000895
12.79	-0.0024	-0.0059	0.000878
12.8	-0.00263	-0.0062	0.000865
12.81	-0.00286	-0.0065	0.000856
12.82	-0.00306	-0.00679	0.000852
12.83	-0.00325	-0.00709	0.000854
12.84	-0.00343	-0.00737	0.00086
12.85	-0.00359	-0.00765	0.000872
12.86	-0.00373	-0.00792	0.000889
12.87	-0.00386	-0.00817	0.000912
12.88	-0.00397	-0.0084	0.00094
12.89	-0.00408	-0.00863	0.000971
12.9	-0.00416	-0.00883	0.001005
12.91	-0.00424	-0.00902	0.001042
12.92	-0.0043	-0.00919	0.00108
12.93	-0.00435	-0.00934	0.001118
12.94	-0.00438	-0.00947	0.001156

12.95	-0.00441	-0.00958	0.001193
12.96	-0.00443	-0.00967	0.001227
12.97	-0.00444	-0.00974	0.001259
12.98	-0.00444	-0.00979	0.001288
12.99	-0.00443	-0.00982	0.001315
13	-0.00441	-0.00984	0.001341
13.01	-0.00439	-0.00985	0.001367
13.02	-0.00437	-0.00985	0.001394
13.03	-0.00434	-0.00983	0.001421
13.04	-0.0043	-0.00982	0.001449
13.05	-0.00426	-0.00979	0.001481
13.06	-0.00422	-0.00977	0.001515
13.07	-0.00417	-0.00975	0.001552
13.08	-0.00412	-0.00973	0.001593
13.09	-0.00407	-0.00971	0.001635
13.1	-0.00401	-0.00968	0.001679
13.11	-0.00396	-0.00966	0.001724
13.12	-0.0039	-0.00964	0.001768
13.13	-0.00385	-0.00961	0.001811
13.14	-0.00379	-0.00959	0.001853
13.15	-0.00373	-0.00956	0.001892
13.16	-0.00367	-0.00953	0.001929
13.17	-0.00361	-0.00949	0.001961
13.18	-0.00355	-0.00946	0.00199
13.19	-0.00349	-0.00942	0.002016
13.2	-0.00343	-0.00938	0.002039
13.21	-0.00336	-0.00934	0.002061
13.22	-0.0033	-0.0093	0.002082
13.23	-0.00324	-0.00926	0.002102
13.24	-0.00318	-0.00921	0.002122
13.25	-0.00311	-0.00917	0.002143
13.26	-0.00305	-0.00913	0.002164
13.27	-0.00298	-0.00909	0.002188
13.28	-0.00291	-0.00904	0.002213
13.29	-0.00285	-0.009	0.002239
13.3	-0.00278	-0.00896	0.002268
13.31	-0.00271	-0.00892	0.002298
13.32	-0.00263	-0.00889	0.00233
13.33	-0.00256	-0.00885	0.002363
13.34	-0.00249	-0.00881	0.002399
13.35	-0.00242	-0.00878	0.002436
13.36	-0.00235	-0.00874	0.002475
13.37	-0.00227	-0.00871	0.002515
13.38	-0.0022	-0.00868	0.002557
13.39	-0.00213	-0.00865	0.0026
13.4	-0.00206	-0.00862	0.002643
13.41	-0.00199	-0.00859	0.002686
13.42	-0.00192	-0.00856	0.002729

13.43	-0.00185	-0.00853	0.00277
13.44	-0.00178	-0.00851	0.00281
13.45	-0.00172	-0.00848	0.002848
13.46	-0.00165	-0.00846	0.002884
13.47	-0.00159	-0.00843	0.002916
13.48	-0.00153	-0.00841	0.002947
13.49	-0.00146	-0.00839	0.002975
13.5	-0.0014	-0.00837	0.003002
13.51	-0.00134	-0.00834	0.003027
13.52	-0.00128	-0.00832	0.003053
13.53	-0.00123	-0.0083	0.003078
13.54	-0.00117	-0.00827	0.003104
13.55	-0.00111	-0.00825	0.00313
13.56	-0.00106	-0.00822	0.003159
13.57	-0.00101	-0.0082	0.003189
13.58	-0.00096	-0.00817	0.003221
13.59	-0.00091	-0.00814	0.003254
13.6	-0.00086	-0.00811	0.003287
13.61	-0.00081	-0.00808	0.003321
13.62	-0.00077	-0.00805	0.003355
13.63	-0.00073	-0.00803	0.003388
13.64	-0.00069	-0.008	0.00342
13.65	-0.00065	-0.00797	0.003451
13.66	-0.00061	-0.00795	0.00348
13.67	-0.00058	-0.00792	0.003507
13.68	-0.00055	-0.0079	0.003531
13.69	-0.00052	-0.00788	0.003552
13.7	-0.00049	-0.00786	0.003571
13.71	-0.00046	-0.00784	0.003586
13.72	-0.00044	-0.00782	0.003599
13.73	-0.00042	-0.00781	0.003607
13.74	-0.0004	-0.00779	0.003613
13.75	-0.00038	-0.00778	0.003614
13.76	-0.00037	-0.00777	0.003612
13.77	-0.00036	-0.00776	0.003605
13.78	-0.00035	-0.00776	0.003595
13.79	-0.00034	-0.00775	0.003582
13.8	-0.00034	-0.00775	0.003567
13.81	-0.00033	-0.00775	0.003549
13.82	-0.00033	-0.00775	0.003531
13.83	-0.00033	-0.00775	0.003512
13.84	-0.00034	-0.00776	0.003493
13.85	-0.00034	-0.00777	0.003475
13.86	-0.00035	-0.00778	0.003458
13.87	-0.00035	-0.00779	0.003443
13.88	-0.00036	-0.00781	0.00343
13.89	-0.00037	-0.00782	0.00342
13.9	-0.00038	-0.00784	0.003412

13.91	-0.00039	-0.00786	0.003406
13.92	-0.0004	-0.00789	0.003404
13.93	-0.00041	-0.00791	0.003405
13.94	-0.00042	-0.00793	0.003409
13.95	-0.00043	-0.00795	0.003417
13.96	-0.00044	-0.00797	0.003429
13.97	-0.00045	-0.00799	0.003445
13.98	-0.00045	-0.00801	0.003464
13.99	-0.00046	-0.00803	0.003487
14	-0.00046	-0.00805	0.003512
14.01	-0.00046	-0.00806	0.003539
14.02	-0.00046	-0.00808	0.003568
14.03	-0.00046	-0.00809	0.003598
14.04	-0.00046	-0.0081	0.003628
14.05	-0.00045	-0.00811	0.003658
14.06	-0.00044	-0.00811	0.003688
14.07	-0.00043	-0.00812	0.003717
14.08	-0.00042	-0.00812	0.003745
14.09	-0.00041	-0.00812	0.003772
14.1	-0.00039	-0.00812	0.003797
14.11	-0.00038	-0.00812	0.003821
14.12	-0.00036	-0.00812	0.003843
14.13	-0.00034	-0.00811	0.003864
14.14	-0.00032	-0.0081	0.003882
14.15	-0.0003	-0.00809	0.003899
14.16	-0.00028	-0.00808	0.003913
14.17	-0.00026	-0.00807	0.003925
14.18	-0.00024	-0.00806	0.003935
14.19	-0.00022	-0.00805	0.003942
14.2	-0.00021	-0.00803	0.003948
14.21	-0.00019	-0.00801	0.003952
14.22	-0.00017	-0.008	0.003954
14.23	-0.00016	-0.00798	0.003955
14.24	-0.00014	-0.00796	0.003955
14.25	-0.00013	-0.00795	0.003953
14.26	-0.00012	-0.00793	0.003951
14.27	-0.0001	-0.00791	0.003947
14.28	#######	-0.0079	0.003943
14.29	#######	-0.00788	0.003938
14.3	#######	-0.00787	0.003932
14.31	#######	-0.00785	0.003926
14.32	#######	-0.00784	0.00392
14.33	#######	-0.00783	0.003913
14.34	#######	-0.00782	0.003906
14.35	#######	-0.00781	0.0039
14.36	#######	-0.0078	0.003893
14.37	#######	-0.0078	0.003886
14.38	#######	-0.00779	0.00388

14.39	#######	-0.00779	0.003874
14.4	#######	-0.00779	0.003868
14.41	#######	-0.00779	0.003862
14.42	#######	-0.00779	0.003857
14.43	#######	-0.00779	0.003852
14.44	#######	-0.0078	0.003847
14.45	#######	-0.0078	0.003843
14.46	#######	-0.0078	0.00384
14.47	#######	-0.0078	0.003836
14.48	#######	-0.00781	0.003833
14.49	#######	-0.00781	0.003831
14.5	#######	-0.00781	0.003829

Table S8. Test Result Data of the Slalom Test at an Adhesion Coefficient of 0.8

Time	carsim	DRL-UKF	UKF
0	#######	0.085682	0.067181
0.01	-0.00143	0.087421	0.067822
0.02	-0.00214	0.089309	0.068643
0.03	-0.00219	0.091334	0.069631
0.04	-0.00162	0.093481	0.070777
0.05	-0.0005	0.095737	0.072068
0.06	0.00114	0.098089	0.073493
0.07	0.003233	0.100522	0.075042
0.08	0.005732	0.103022	0.076702
0.09	0.008585	0.105577	0.078463
0.1	0.011742	0.108172	0.080313
0.11	0.015149	0.110793	0.082241
0.12	0.018756	0.113427	0.084236
0.13	0.022512	0.11606	0.086286
0.14	0.026364	0.118678	0.088381
0.15	0.03026	0.121268	0.090509
0.16	0.03415	0.123816	0.092658
0.17	0.037982	0.126306	0.094818
0.18	0.041704	0.128725	0.096977
0.19	0.045264	0.131057	0.099124
0.2	0.048611	0.133287	0.101248
0.21	0.051702	0.1354	0.103337
0.22	0.054532	0.137383	0.105379
0.23	0.057104	0.139218	0.107362
0.24	0.05942	0.140893	0.109274
0.25	0.061484	0.142392	0.111102
0.26	0.063299	0.143703	0.112835

0.27	0.064869	0.144835	0.114461
0.28	0.066196	0.145796	0.115966
0.29	0.067284	0.146597	0.117339
0.3	0.068136	0.147249	0.118568
0.31	0.068759	0.147762	0.119644
0.32	0.069174	0.148147	0.120572
0.33	0.069408	0.148413	0.121361
0.34	0.069485	0.148572	0.122018
0.35	0.069433	0.148633	0.122553
0.36	0.069276	0.148608	0.122973
0.37	0.06904	0.148505	0.123289
0.38	0.068751	0.148337	0.123507
0.39	0.068434	0.148112	0.123637
0.4	0.068115	0.14784	0.123686
0.41	0.067815	0.147534	0.123665
0.42	0.067533	0.147201	0.12358
0.43	0.067264	0.146853	0.12344
0.44	0.067002	0.1465	0.123255
0.45	0.066742	0.146153	0.123032
0.46	0.066478	0.145818	0.12278
0.47	0.066204	0.145497	0.122507
0.48	0.065914	0.145189	0.122223
0.49	0.065605	0.144891	0.121935
0.5	0.065268	0.144601	0.121652
0.51	0.064903	0.14432	0.121381
0.52	0.064513	0.144044	0.121122
0.53	0.064108	0.143772	0.120873
0.54	0.063696	0.143503	0.120632
0.55	0.063284	0.143234	0.120397
0.56	0.062881	0.142967	0.120167
0.57	0.062494	0.142702	0.11994
0.58	0.062132	0.142445	0.119715
0.59	0.061803	0.142198	0.119488
0.6	0.061516	0.141967	0.119259
0.61	0.061275	0.141755	0.119028
0.62	0.061077	0.141565	0.118797
0.63	0.060916	0.141402	0.118571
0.64	0.060787	0.14127	0.118357
0.65	0.060682	0.141172	0.118158
0.66	0.060597	0.14111	0.117979
0.67	0.060525	0.141079	0.117826
0.68	0.06046	0.141069	0.117703
0.69	0.060396	0.141072	0.117615
0.7	0.060327	0.14108	0.117568
0.71	0.06025	0.141085	0.117563
0.72	0.060165	0.141078	0.117589
0.73	0.060078	0.14105	0.117634
0.74	0.059993	0.140995	0.117684

0.75	0.059915	0.140902	0.117726
0.76	0.059847	0.140769	0.117747
0.77	0.059793	0.140612	0.117732
0.78	0.059758	0.140455	0.11767
0.79	0.059747	0.140318	0.117546
0.8	0.059763	0.140225	0.117347
0.81	0.05981	0.140196	0.117069
0.82	0.059882	0.140255	0.116745
0.83	0.059977	0.140423	0.116418
0.84	0.060087	0.140723	0.116129
0.85	0.060208	0.141176	0.115921
0.86	0.060335	0.141796	0.115836
0.87	0.060462	0.142562	0.115916
0.88	0.060585	0.143447	0.116204
0.89	0.060698	0.14442	0.116742
0.9	0.060795	0.145454	0.117571
0.91	0.060874	0.146519	0.11872
0.92	0.060934	0.147586	0.120153
0.93	0.060979	0.148627	0.121822
0.94	0.061011	0.149613	0.123676
0.95	0.061032	0.150515	0.125666
0.96	0.061044	0.151312	0.127742
0.97	0.061051	0.15201	0.129855
0.98	0.061054	0.152624	0.131955
0.99	0.061056	0.153168	0.133992
1	0.061058	0.153658	0.135918
1.01	0.061064	0.154107	0.137693
1.02	0.061074	0.15453	0.139328
1.03	0.061087	0.154941	0.140845
1.04	0.061105	0.155356	0.142264
1.05	0.061126	0.155787	0.143606
1.06	0.061151	0.156244	0.144894
1.07	0.061181	0.156712	0.146149
1.08	0.061215	0.157168	0.147392
1.09	0.061254	0.157592	0.148644
1.1	0.061297	0.157962	0.149927
1.11	0.061344	0.158256	0.151252
1.12	0.061396	0.158453	0.152589
1.13	0.06145	0.158532	0.153898
1.14	0.061507	0.158471	0.155139
1.15	0.061566	0.158249	0.156271
1.16	0.061625	0.157853	0.157255
1.17	0.061685	0.157303	0.158049
1.18	0.061743	0.156629	0.158615
1.19	0.0618	0.155859	0.158911
1.2	0.061855	0.155024	0.158898
1.21	0.061906	0.154151	0.15855
1.22	0.061955	0.153271	0.157903

1.23	0.062003	0.152412	0.157005
1.24	0.06205	0.151604	0.155906
1.25	0.062097	0.150875	0.154656
1.26	0.062145	0.15025	0.153305
1.27	0.062195	0.149724	0.151902
1.28	0.062248	0.14929	0.150498
1.29	0.062304	0.148939	0.149141
1.3	0.062364	0.148661	0.147881
1.31	0.062429	0.148449	0.146758
1.32	0.062498	0.148294	0.145772
1.33	0.062569	0.148187	0.144912
1.34	0.062642	0.148119	0.144167
1.35	0.062714	0.148081	0.143527
1.36	0.062785	0.148067	0.142981
1.37	0.062853	0.148075	0.142519
1.38	0.062918	0.148104	0.14213
1.39	0.062977	0.148156	0.141804
1.4	0.063029	0.148229	0.14153
1.41	0.063074	0.148325	0.1413
1.42	0.063112	0.148443	0.14111
1.43	0.063145	0.148582	0.14096
1.44	0.063173	0.148744	0.14085
1.45	0.063197	0.148928	0.14078
1.46	0.06322	0.149134	0.140747
1.47	0.063241	0.149358	0.140752
1.48	0.063262	0.149596	0.140795
1.49	0.063284	0.149846	0.140874
1.5	0.063308	0.150103	0.140988
1.51	0.063336	0.150363	0.141137
1.52	0.063366	0.150623	0.141317
1.53	0.063398	0.150878	0.141521
1.54	0.063433	0.151126	0.141746
1.55	0.063468	0.151362	0.141987
1.56	0.063504	0.151583	0.142237
1.57	0.06354	0.15179	0.142493
1.58	0.063576	0.151983	0.14275
1.59	0.06361	0.152164	0.143002
1.6	0.063644	0.152332	0.143245
1.61	0.063675	0.152491	0.143474
1.62	0.063704	0.152639	0.14369
1.63	0.063732	0.152779	0.143893
1.64	0.063758	0.152911	0.144085
1.65	0.063783	0.153036	0.144266
1.66	0.063806	0.153155	0.144437
1.67	0.063829	0.153267	0.144598
1.68	0.06385	0.153374	0.144752
1.69	0.063871	0.153473	0.144899
1.7	0.063891	0.153566	0.145039

1.71	0.063911	0.153652	0.145174
1.72	0.06393	0.153731	0.145302
1.73	0.063949	0.153802	0.145425
1.74	0.063969	0.153866	0.145539
1.75	0.063988	0.153921	0.145647
1.76	0.064008	0.153969	0.145745
1.77	0.064028	0.154009	0.145835
1.78	0.064049	0.154043	0.145916
1.79	0.064071	0.154072	0.145986
1.8	0.064094	0.154097	0.146046
1.81	0.064117	0.154119	0.146095
1.82	0.064142	0.154138	0.146134
1.83	0.064168	0.154156	0.146164
1.84	0.064194	0.154174	0.146187
1.85	0.064222	0.154192	0.146203
1.86	0.064249	0.154212	0.146214
1.87	0.064278	0.154233	0.14622
1.88	0.064307	0.154255	0.146224
1.89	0.064337	0.154276	0.146226
1.9	0.064366	0.154298	0.146228
1.91	0.064396	0.154318	0.14623
1.92	0.064426	0.154337	0.146232
1.93	0.064455	0.154355	0.146235
1.94	0.064482	0.15437	0.146237
1.95	0.064508	0.154382	0.146239
1.96	0.064531	0.154391	0.14624
1.97	0.06455	0.154397	0.146239
1.98	0.064566	0.1544	0.146238
1.99	0.064578	0.154402	0.146234
2	0.064584	0.154401	0.146229
2.01	0.064586	0.154399	0.146221
2.02	0.064583	0.154397	0.146211
2.03	0.064578	0.154394	0.1462
2.04	0.064572	0.15439	0.146187
2.05	0.064567	0.154387	0.146174
2.06	0.064563	0.154385	0.146161
2.07	0.064564	0.154384	0.146147
2.08	0.064569	0.154386	0.146134
2.09	0.064581	0.154391	0.146122
2.1	0.064601	0.154401	0.146112
2.11	0.064631	0.154415	0.146103
2.12	0.064673	0.154436	0.146097
2.13	0.064731	0.154464	0.146094
2.14	0.064805	0.154499	0.146096
2.15	0.0649	0.154544	0.146103
2.16	0.065017	0.154598	0.146117
2.17	0.065159	0.154661	0.146139
2.18	0.06533	0.154734	0.146168

2.19	0.065531	0.154816	0.146207
2.2	0.065764	0.154907	0.146256
2.21	0.066032	0.155007	0.146316
2.22	0.066327	0.155115	0.146387
2.23	0.066641	0.155232	0.146467
2.24	0.066966	0.155358	0.146557
2.25	0.067293	0.155491	0.146655
2.26	0.067614	0.155633	0.146761
2.27	0.067921	0.155781	0.146874
2.28	0.068205	0.155934	0.146993
2.29	0.068459	0.156092	0.147119
2.3	0.068674	0.156252	0.147249
2.31	0.068843	0.156413	0.147384
2.32	0.068966	0.156575	0.147523
2.33	0.069046	0.156735	0.147663
2.34	0.069084	0.156893	0.147805
2.35	0.069082	0.157047	0.147947
2.36	0.069042	0.157195	0.148088
2.37	0.068967	0.157336	0.148227
2.38	0.068857	0.157467	0.148363
2.39	0.068715	0.157588	0.148495
2.4	0.068543	0.157695	0.148622
2.41	0.068342	0.157786	0.148742
2.42	0.068117	0.157861	0.148854
2.43	0.06787	0.157916	0.148955
2.44	0.067605	0.157949	0.149044
2.45	0.067325	0.15796	0.149119
2.46	0.067034	0.157945	0.149176
2.47	0.066734	0.157908	0.149215
2.48	0.06643	0.15785	0.149234
2.49	0.066125	0.157773	0.149229
2.5	0.065822	0.157681	0.149199
2.51	0.065525	0.157574	0.149144
2.52	0.065242	0.157456	0.149064
2.53	0.064979	0.157328	0.148964
2.54	0.064745	0.157193	0.148845
2.55	0.064547	0.157054	0.148712
2.56	0.064392	0.156912	0.148566
2.57	0.064289	0.156766	0.148411
2.58	0.064245	0.156618	0.14825
2.59	0.064268	0.156466	0.148086
2.6	0.064365	0.15631	0.147921
2.61	0.064539	0.15615	0.147759
2.62	0.064773	0.155986	0.147597
2.63	0.065044	0.155817	0.147434
2.64	0.065331	0.155643	0.147267
2.65	0.065611	0.155463	0.147094
2.66	0.065863	0.155277	0.146913

2.67	0.066064	0.155081	0.146722
2.68	0.066191	0.154869	0.146519
2.69	0.066224	0.154638	0.146302
2.7	0.066139	0.154383	0.146068
2.71	0.065918	0.154099	0.145816
2.72	0.065557	0.153782	0.14554
2.73	0.065055	0.153428	0.145239
2.74	0.064411	0.15303	0.144907
2.75	0.063623	0.152586	0.144541
2.76	0.062691	0.152089	0.144136
2.77	0.061614	0.151535	0.14369
2.78	0.06039	0.150916	0.143198
2.79	0.05902	0.150225	0.142657
2.8	0.057502	0.149457	0.142061
2.81	0.055836	0.148605	0.141408
2.82	0.054029	0.147661	0.140691
2.83	0.052088	0.146621	0.139904
2.84	0.050022	0.145476	0.139039
2.85	0.047837	0.144222	0.138091
2.86	0.045542	0.142851	0.137054
2.87	0.043143	0.141361	0.135919
2.88	0.04065	0.139751	0.134683
2.89	0.038069	0.138018	0.133336
2.9	0.035408	0.13616	0.131874
2.91	0.032671	0.134176	0.130291
2.92	0.029849	0.132064	0.128583
2.93	0.026928	0.129822	0.126749
2.94	0.023896	0.127447	0.124787
2.95	0.020738	0.124939	0.122695
2.96	0.017442	0.122295	0.120471
2.97	0.013995	0.119519	0.118112
2.98	0.010383	0.116614	0.115618
2.99	0.006593	0.113583	0.112986
3	0.002611	0.110428	0.110214
3.01	-0.00157	0.107154	0.107301
3.02	-0.00595	0.103763	0.104248
3.03	-0.01051	0.10026	0.101059
3.04	-0.01525	0.096646	0.097734
3.05	-0.02016	0.092926	0.094277
3.06	-0.02524	0.089102	0.090691
3.07	-0.03046	0.085182	0.086976
3.08	-0.03582	0.081172	0.083136
3.09	-0.04132	0.077079	0.079173
3.1	-0.04694	0.072909	0.07509
3.11	-0.05267	0.06867	0.070889
3.12	-0.0585	0.064367	0.066575
3.13	-0.06438	0.060009	0.062155
3.14	-0.07031	0.0556	0.057634

3.15	-0.07625	0.05115	0.053017
3.16	-0.08217	0.046664	0.048312
3.17	-0.08806	0.042152	0.043522
3.18	-0.09389	0.037625	0.038655
3.19	-0.09962	0.033092	0.033715
3.2	-0.10524	0.028564	0.02871
3.21	-0.11072	0.024052	0.023645
3.22	-0.11604	0.019565	0.018533
3.23	-0.12117	0.015114	0.01339
3.24	-0.1261	0.010709	0.008228
3.25	-0.1308	0.00636	0.003063
3.26	-0.13525	0.002077	-0.00209
3.27	-0.13944	-0.00169	-0.00722
3.28	-0.14333	-0.00496	-0.01231
3.29	-0.14691	-0.00817	-0.01735
3.3	-0.15016	-0.01131	-0.02232
3.31	-0.15305	-0.01438	-0.02721
3.32	-0.15559	-0.01737	-0.032
3.33	-0.15778	-0.02029	-0.03668
3.34	-0.15962	-0.02312	-0.04123
3.35	-0.16111	-0.02586	-0.04563
3.36	-0.16225	-0.02851	-0.04987
3.37	-0.16305	-0.03107	-0.05393
3.38	-0.1635	-0.03352	-0.0578
3.39	-0.16361	-0.03588	-0.06145
3.4	-0.16338	-0.03813	-0.06488
3.41	-0.16282	-0.04027	-0.06807
3.42	-0.16195	-0.0423	-0.07102
3.43	-0.16083	-0.04421	-0.07372
3.44	-0.15949	-0.04601	-0.07618
3.45	-0.15797	-0.04768	-0.07839
3.46	-0.15632	-0.04922	-0.08036
3.47	-0.15456	-0.05064	-0.08209
3.48	-0.15276	-0.05194	-0.08357
3.49	-0.15093	-0.05311	-0.0848
3.5	-0.14913	-0.05416	-0.0858
3.51	-0.14739	-0.05508	-0.08655
3.52	-0.1457	-0.05589	-0.08707
3.53	-0.14407	-0.05657	-0.08736
3.54	-0.14248	-0.05714	-0.08745
3.55	-0.14092	-0.05758	-0.08734
3.56	-0.1394	-0.05791	-0.08705
3.57	-0.13791	-0.05812	-0.08658
3.58	-0.13643	-0.05822	-0.08596
3.59	-0.13496	-0.0582	-0.08518
3.6	-0.1335	-0.05806	-0.08427
3.61	-0.13203	-0.05781	-0.08323
3.62	-0.13052	-0.05744	-0.08208

3.63	-0.12891	-0.05695	-0.0808
3.64	-0.12717	-0.05636	-0.07941
3.65	-0.12526	-0.05564	-0.0779
3.66	-0.12311	-0.05482	-0.07628
3.67	-0.12071	-0.05387	-0.07456
3.68	-0.11799	-0.05279	-0.07274
3.69	-0.11491	-0.05159	-0.07081
3.7	-0.11144	-0.05024	-0.06878
3.71	-0.10753	-0.04875	-0.06666
3.72	-0.10318	-0.0471	-0.06443
3.73	-0.0984	-0.0453	-0.06209
3.74	-0.09318	-0.04333	-0.05962
3.75	-0.08754	-0.0412	-0.05701
3.76	-0.08147	-0.03888	-0.05425
3.77	-0.07498	-0.03636	-0.05133
3.78	-0.06807	-0.03362	-0.04824
3.79	-0.06075	-0.03065	-0.04497
3.8	-0.05302	-0.0274	-0.04151
3.81	-0.04489	-0.02388	-0.03784
3.82	-0.03636	-0.02004	-0.03394
3.83	-0.02744	-0.01588	-0.02977
3.84	-0.01814	-0.01138	-0.02531
3.85	-0.00848	-0.0065	-0.02052
3.86	0.001541	-0.00122	-0.01537
3.87	0.011913	0.005677	-0.00983
3.88	0.022625	0.013507	-0.00387
3.89	0.033668	0.022007	0.002544
3.9	0.045032	0.031233	0.00944
3.91	0.05671	0.041245	0.016845
3.92	0.068704	0.052099	0.024768
3.93	0.081014	0.063854	0.033213
3.94	0.093645	0.076567	0.042184
3.95	0.106598	0.090297	0.051683
3.96	0.119876	0.105079	0.061714
3.97	0.133483	0.120857	0.072282
3.98	0.14742	0.137553	0.08339
3.99	0.161691	0.155087	0.095041
4	0.176298	0.173381	0.10724
4.01	0.191243	0.192357	0.119983
4.02	0.206526	0.211936	0.133241
4.03	0.222148	0.23204	0.146982
4.04	0.238108	0.252589	0.161168
4.05	0.254407	0.273506	0.175767
4.06	0.271043	0.294716	0.190742
4.07	0.288018	0.316166	0.20606
4.08	0.30533	0.337806	0.221685
4.09	0.32298	0.359587	0.237583
4.1	0.340968	0.381459	0.253719

4.11	0.359286	0.403375	0.270059
4.12	0.377899	0.425283	0.286572
4.13	0.396765	0.447136	0.303227
4.14	0.415839	0.468883	0.319995
4.15	0.43508	0.490476	0.336845
4.16	0.454446	0.511869	0.353746
4.17	0.473892	0.533033	0.370669
4.18	0.493377	0.55394	0.387584
4.19	0.512858	0.574566	0.404459
4.2	0.532292	0.594884	0.421264
4.21	0.551641	0.614867	0.437971
4.22	0.570881	0.634491	0.454556
4.23	0.589994	0.653728	0.470996
4.24	0.608962	0.672552	0.48727
4.25	0.627767	0.690938	0.503354
4.26	0.646388	0.708869	0.519226
4.27	0.664808	0.72637	0.534864
4.28	0.683009	0.743476	0.550246
4.29	0.700971	0.760221	0.565349
4.3	0.718676	0.776638	0.580151
4.31	0.736114	0.792764	0.594634
4.32	0.75331	0.808633	0.608805
4.33	0.770298	0.824278	0.622676
4.34	0.78711	0.839735	0.636256
4.35	0.80378	0.855038	0.649558
4.36	0.820341	0.870218	0.662593
4.37	0.836828	0.885289	0.675373
4.38	0.853273	0.900264	0.687908
4.39	0.869709	0.915153	0.700209
4.4	0.886171	0.929968	0.712289
4.41	0.902677	0.944719	0.724157
4.42	0.91919	0.959419	0.735817
4.43	0.935657	0.974078	0.74727
4.44	0.952028	0.988708	0.758517
4.45	0.96825	1.00332	0.769562
4.46	0.98427	1.017912	0.780406
4.47	1.000039	1.032433	0.791051
4.48	1.015502	1.046819	0.801498
4.49	1.030609	1.061005	0.81175
4.5	1.045308	1.074928	0.821808
4.51	1.059546	1.088522	0.831673
4.52	1.07327	1.101723	0.841341
4.53	1.086427	1.114468	0.850807
4.54	1.098961	1.126692	0.860065
4.55	1.110819	1.138331	0.86911
4.56	1.121948	1.149332	0.877937
4.57	1.132294	1.159697	0.88654
4.58	1.141801	1.169436	0.894914

4.59	1.150418	1.178563	0.903055
4.6	1.158089	1.187089	0.910956
4.61	1.164769	1.195027	0.918609
4.62	1.170443	1.202389	0.925995
4.63	1.175105	1.209187	0.93309
4.64	1.178747	1.215434	0.93987
4.65	1.181362	1.221142	0.946315
4.66	1.182944	1.226317	0.952399
4.67	1.183487	1.230944	0.9581
4.68	1.182982	1.235001	0.963395
4.69	1.181424	1.238467	0.968261
4.7	1.178805	1.24132	0.972675
4.71	1.175124	1.24354	0.976613
4.72	1.170395	1.245104	0.980048
4.73	1.164638	1.245991	0.982953
4.74	1.157872	1.24618	0.985299
4.75	1.150117	1.245649	0.987058
4.76	1.141393	1.244378	0.988204
4.77	1.131719	1.242355	0.988708
4.78	1.121114	1.239565	0.988542
4.79	1.109599	1.235999	0.987679
4.8	1.097193	1.231642	0.986091
4.81	1.083913	1.226484	0.983756
4.82	1.069775	1.220512	0.980679
4.83	1.05479	1.213714	0.976869
4.84	1.038971	1.206077	0.972336
4.85	1.022331	1.19759	0.967091
4.86	1.004882	1.188238	0.961144
4.87	0.986638	1.177991	0.954505
4.88	0.967609	1.166818	0.947184
4.89	0.94781	1.154688	0.939191
4.9	0.927253	1.141569	0.930537
4.91	0.905925	1.127429	0.921216
4.92	0.883718	1.112236	0.911162
4.93	0.860498	1.095959	0.900293
4.94	0.836131	1.078566	0.888527
4.95	0.810484	1.060025	0.875783
4.96	0.783422	1.040302	0.861978
4.97	0.754812	1.019345	0.847031
4.98	0.724521	0.9971	0.83086
4.99	0.692414	0.973514	0.813383
5	0.658358	0.948533	0.794519
5.01	0.62228	0.922101	0.774202
5.02	0.584357	0.894166	0.752432
5.03	0.544823	0.864673	0.729226
5.04	0.503916	0.833567	0.704602
5.05	0.461872	0.800796	0.678576
5.06	0.418929	0.766333	0.651165

5.07	0.375321	0.73027	0.622385
5.08	0.331286	0.692727	0.592254
5.09	0.287061	0.653823	0.560789
5.1	0.242881	0.613678	0.528006
5.11	0.198952	0.572413	0.493931
5.12	0.155355	0.530147	0.458626
5.13	0.112137	0.487	0.422161
5.14	0.069348	0.443092	0.384606
5.15	0.027037	0.398542	0.346033
5.16	-0.01475	0.353453	0.306512
5.17	-0.05596	0.307851	0.266112
5.18	-0.09655	0.261745	0.224906
5.19	-0.13646	0.215142	0.182963
5.2	-0.17565	0.168051	0.140354
5.21	-0.21408	0.12048	0.097164
5.22	-0.25172	0.072438	0.053539
5.23	-0.28856	0.023932	0.00964
5.24	-0.32457	-0.0198	-0.03437
5.25	-0.35974	-0.0589	-0.07834
5.26	-0.39404	-0.09832	-0.1221
5.27	-0.42747	-0.13794	-0.16549
5.28	-0.46001	-0.17762	-0.20835
5.29	-0.49163	-0.21721	-0.25052
5.3	-0.52231	-0.25657	-0.29184
5.31	-0.55206	-0.29555	-0.33217
5.32	-0.58093	-0.33401	-0.37144
5.33	-0.60897	-0.3718	-0.40963
5.34	-0.63625	-0.40878	-0.44667
5.35	-0.66283	-0.4448	-0.48254
5.36	-0.68879	-0.47976	-0.51719
5.37	-0.71417	-0.51367	-0.55057
5.38	-0.73905	-0.5466	-0.58265
5.39	-0.76348	-0.57861	-0.61339
5.4	-0.78754	-0.60976	-0.64274
5.41	-0.81127	-0.64011	-0.67067
5.42	-0.83468	-0.66971	-0.69724
5.43	-0.85777	-0.69864	-0.72249
5.44	-0.88053	-0.72694	-0.7465
5.45	-0.90296	-0.75468	-0.76931
5.46	-0.92506	-0.78189	-0.791
5.47	-0.94681	-0.80851	-0.81161
5.48	-0.96823	-0.83444	-0.83121
5.49	-0.9893	-0.85958	-0.84985
5.5	-1.01001	-0.88385	-0.86761
5.51	-1.03036	-0.90714	-0.88452
5.52	-1.05027	-0.92938	-0.90063
5.53	-1.06963	-0.95045	-0.91597
5.54	-1.08836	-0.97027	-0.93057

5.55	-1.10637	-0.98875	-0.94447
5.56	-1.12356	-1.00581	-0.95768
5.57	-1.13984	-1.02149	-0.97025
5.58	-1.15513	-1.03587	-0.98221
5.59	-1.16932	-1.049	-0.99358
5.6	-1.18232	-1.06096	-1.0044
5.61	-1.19407	-1.0718	-1.01469
5.62	-1.20464	-1.08159	-1.0245
5.63	-1.21409	-1.09039	-1.03382
5.64	-1.22252	-1.09828	-1.0427
5.65	-1.23002	-1.10532	-1.05115
5.66	-1.23667	-1.11156	-1.0592
5.67	-1.24255	-1.11701	-1.06687
5.68	-1.24775	-1.12168	-1.07418
5.69	-1.25236	-1.12558	-1.08117
5.7	-1.25647	-1.12871	-1.08784
5.71	-1.26013	-1.13106	-1.09423
5.72	-1.26335	-1.13266	-1.10031
5.73	-1.26611	-1.13349	-1.10607
5.74	-1.26838	-1.13356	-1.11148
5.75	-1.27016	-1.13289	-1.11653
5.76	-1.27142	-1.13147	-1.1212
5.77	-1.27214	-1.12934	-1.12546
5.78	-1.27229	-1.12653	-1.1293
5.79	-1.27187	-1.12308	-1.1327
5.8	-1.27086	-1.11903	-1.13563
5.81	-1.26923	-1.11441	-1.13809
5.82	-1.26701	-1.10926	-1.14006
5.83	-1.26422	-1.10361	-1.14156
5.84	-1.26089	-1.09751	-1.14259
5.85	-1.25703	-1.09098	-1.14315
5.86	-1.25268	-1.08407	-1.14324
5.87	-1.24786	-1.07681	-1.14287
5.88	-1.24259	-1.06923	-1.14204
5.89	-1.23689	-1.06138	-1.14076
5.9	-1.23079	-1.05328	-1.13903
5.91	-1.22431	-1.04499	-1.13684
5.92	-1.21738	-1.03652	-1.13418
5.93	-1.20993	-1.02792	-1.13101
5.94	-1.20191	-1.01923	-1.12733
5.95	-1.19324	-1.01048	-1.12309
5.96	-1.18385	-1.00171	-1.11827
5.97	-1.17367	-0.99289	-1.11284
5.98	-1.16265	-0.98404	-1.10679
5.99	-1.1507	-0.97512	-1.10008
6	-1.13775	-0.96614	-1.09269
6.01	-1.12378	-0.95707	-1.0846
6.02	-1.1088	-0.94791	-1.07586

6.03	-1.09287	-0.93865	-1.06651
6.04	-1.07606	-0.92927	-1.05661
6.05	-1.05842	-0.91976	-1.0462
6.06	-1.04001	-0.91012	-1.03535
6.07	-1.02088	-0.90033	-1.0241
6.08	-1.0011	-0.89041	-1.01251
6.09	-0.98071	-0.88035	-1.00062
6.1	-0.95979	-0.87016	-0.98849
6.11	-0.93838	-0.85984	-0.97616
6.12	-0.91656	-0.84939	-0.96357
6.13	-0.89442	-0.8388	-0.95067
6.14	-0.87202	-0.82809	-0.93739
6.15	-0.84945	-0.81726	-0.92367
6.16	-0.82679	-0.8063	-0.90946
6.17	-0.80412	-0.79518	-0.89468
6.18	-0.7815	-0.78389	-0.87928
6.19	-0.75903	-0.77239	-0.86319
6.2	-0.73677	-0.76066	-0.84636
6.21	-0.71477	-0.74868	-0.82873
6.22	-0.69291	-0.7364	-0.81036
6.23	-0.671	-0.72381	-0.79128
6.24	-0.6489	-0.71088	-0.77157
6.25	-0.62642	-0.69758	-0.75126
6.26	-0.60341	-0.68387	-0.73043
6.27	-0.57969	-0.66966	-0.70913
6.28	-0.55511	-0.65482	-0.68741
6.29	-0.52949	-0.63926	-0.66532
6.3	-0.50266	-0.62285	-0.64293
6.31	-0.47451	-0.60548	-0.62026
6.32	-0.44505	-0.58705	-0.5972
6.33	-0.41434	-0.56743	-0.57359
6.34	-0.38246	-0.54653	-0.5493
6.35	-0.34946	-0.52422	-0.52417
6.36	-0.3154	-0.50042	-0.49807
6.37	-0.28036	-0.47516	-0.47085
6.38	-0.24439	-0.44849	-0.44236
6.39	-0.20756	-0.42046	-0.41245
6.4	-0.16993	-0.39112	-0.38099
6.41	-0.13157	-0.36053	-0.34786
6.42	-0.09252	-0.32875	-0.31315
6.43	-0.05286	-0.29581	-0.27698
6.44	-0.01264	-0.26179	-0.23948
6.45	0.028074	-0.22672	-0.20075
6.46	0.069233	-0.19065	-0.16093
6.47	0.110774	-0.15357	-0.12014
6.48	0.152638	-0.11547	-0.0785
6.49	0.194765	-0.07633	-0.03613
6.5	0.237096	-0.03613	0.006843

6.51	0.279577	0.006512	0.050318
6.52	0.322164	0.06006	0.094245
6.53	0.364819	0.115014	0.138593
6.54	0.407503	0.171397	0.183331
6.55	0.450176	0.22923	0.228427
6.56	0.4928	0.288484	0.27385
6.57	0.535336	0.348925	0.319569
6.58	0.577745	0.410268	0.365552
6.59	0.619988	0.472227	0.411768
6.6	0.662026	0.534516	0.458185
6.61	0.703835	0.596849	0.504755
6.62	0.745453	0.658941	0.551358
6.63	0.786932	0.720507	0.597859
6.64	0.828325	0.781261	0.64412
6.65	0.869685	0.840916	0.690005
6.66	0.911064	0.899241	0.735378
6.67	0.952514	0.956222	0.780103
6.68	0.994089	1.011895	0.824042
6.69	1.035841	1.066299	0.867059
6.7	1.077823	1.119473	0.909019
6.71	1.120073	1.171455	0.949812
6.72	1.162576	1.222282	0.989448
6.73	1.205302	1.271994	1.027962
6.74	1.248223	1.320629	1.065391
6.75	1.291308	1.368224	1.101772
6.76	1.334528	1.414806	1.137141
6.77	1.377854	1.460348	1.171535
6.78	1.421256	1.504814	1.20499
6.79	1.464705	1.548165	1.237544
6.8	1.508172	1.590363	1.269232
6.81	1.551609	1.631371	1.30009
6.82	1.594906	1.671149	1.330148
6.83	1.637932	1.70966	1.359435
6.84	1.680558	1.746867	1.387978
6.85	1.722655	1.78273	1.415807
6.86	1.764094	1.81723	1.44295
6.87	1.804744	1.85042	1.469435
6.88	1.844478	1.882368	1.495292
6.89	1.883166	1.913146	1.520548
6.9	1.920678	1.942822	1.545233
6.91	1.95691	1.971467	1.569368
6.92	1.99186	1.999151	1.592947
6.93	2.025548	2.025943	1.615957
6.94	2.057997	2.051914	1.638385
6.95	2.089229	2.077134	1.660218
6.96	2.119266	2.101657	1.681443
6.97	2.148128	2.125476	1.702047
6.98	2.175839	2.148567	1.722017

6.99	2.20242	2.17091	1.74134
7	2.227893	2.192479	1.760004
7.01	2.252277	2.213254	1.777999
7.02	2.275579	2.233211	1.795335
7.03	2.297803	2.252327	1.812026
7.04	2.318952	2.27058	1.828086
7.05	2.339032	2.287948	1.843528
7.06	2.358046	2.304405	1.858367
7.07	2.375999	2.319925	1.872615
7.08	2.392894	2.33448	1.886288
7.09	2.408735	2.34804	1.899398
7.1	2.423526	2.360578	1.91196
7.11	2.437262	2.372065	1.923979
7.12	2.449894	2.382473	1.935425
7.13	2.461363	2.391772	1.946261
7.14	2.471612	2.399936	1.956449
7.15	2.480581	2.406934	1.965951
7.16	2.488212	2.412755	1.974729
7.17	2.494446	2.417452	1.982746
7.18	2.499225	2.421091	1.989963
7.19	2.502491	2.423742	1.996343
7.2	2.504185	2.425473	2.001848
7.21	2.504258	2.426352	2.006448
7.22	2.502707	2.426447	2.010141
7.23	2.499537	2.425827	2.012933
7.24	2.494754	2.424559	2.014831
7.25	2.488364	2.422713	2.01584
7.26	2.480371	2.420337	2.015966
7.27	2.470783	2.417409	2.015216
7.28	2.459605	2.413886	2.013596
7.29	2.446843	2.409726	2.011111
7.3	2.432502	2.404886	2.007768
7.31	2.416596	2.399325	2.00357
7.32	2.399161	2.393001	1.998509
7.33	2.380242	2.38587	1.992575
7.34	2.359884	2.377892	1.985757
7.35	2.338131	2.369024	1.978044
7.36	2.315028	2.359226	1.969427
7.37	2.290619	2.348465	1.959893
7.38	2.264948	2.33671	1.949433
7.39	2.23806	2.323931	1.938036
7.4	2.21	2.310096	1.925691
7.41	2.180771	2.295176	1.91239
7.42	2.150215	2.279139	1.89813
7.43	2.118132	2.261955	1.882911
7.44	2.084325	2.243593	1.866731
7.45	2.048593	2.224021	1.849592
7.46	2.010739	2.203178	1.831491

7.47	1.970563	2.180867	1.812428
7.48	1.927866	2.156861	1.792403
7.49	1.88245	2.130932	1.771414
7.5	1.834115	2.102852	1.749463
7.51	1.782678	2.072395	1.726506
7.52	1.728017	2.039331	1.702343
7.53	1.670025	2.003434	1.676729
7.54	1.608597	1.964476	1.649423
7.55	1.543626	1.922228	1.620181
7.56	1.475006	1.876513	1.588759
7.57	1.402631	1.827351	1.554916
7.58	1.326393	1.774811	1.518408
7.59	1.246187	1.718961	1.478993
7.6	1.161907	1.659873	1.436427
7.61	1.073583	1.597615	1.390523
7.62	0.981795	1.532256	1.341315
7.63	0.887262	1.463866	1.288893
7.64	0.7907	1.392514	1.233347
7.65	0.692827	1.31827	1.174767
7.66	0.59436	1.241221	1.113241
7.67	0.496017	1.16153	1.048861
7.68	0.398516	1.079379	0.981716
7.69	0.302573	0.994947	0.911896
7.7	0.208907	0.908416	0.83949
7.71	0.118128	0.819966	0.764606
7.72	0.030425	0.72978	0.687422
7.73	-0.05412	0.638038	0.608137
7.74	-0.13543	0.54492	0.526945
7.75	-0.21342	0.450608	0.444044
7.76	-0.288	0.355289	0.35963
7.77	-0.3591	0.259181	0.2739
7.78	-0.42664	0.162504	0.187051
7.79	-0.49053	0.065482	0.099279
7.8	-0.55069	-0.02505	0.01078
7.81	-0.6071	-0.10185	-0.07821
7.82	-0.65989	-0.17838	-0.1673
7.83	-0.70928	-0.25449	-0.25605
7.84	-0.75548	-0.32998	-0.34405
7.85	-0.79868	-0.40469	-0.43085
7.86	-0.83909	-0.47844	-0.51603
7.87	-0.87692	-0.55104	-0.59915
7.88	-0.91236	-0.62228	-0.67979
7.89	-0.94563	-0.69198	-0.75751
7.9	-0.97692	-0.75995	-0.83189
7.91	-1.00644	-0.82598	-0.90257
7.92	-1.0343	-0.88989	-0.96954
7.93	-1.06065	-0.95148	-1.03286
7.94	-1.08559	-1.01056	-1.0926

7.95	-1.10927	-1.06693	-1.14882
7.96	-1.13179	-1.12045	-1.20158
7.97	-1.15329	-1.17121	-1.25095
7.98	-1.17388	-1.21935	-1.29699
7.99	-1.1937	-1.26501	-1.33977
8	-1.21286	-1.30834	-1.37934
8.01	-1.23148	-1.34946	-1.4158
8.02	-1.24967	-1.38852	-1.44923
8.03	-1.26753	-1.42567	-1.47977
8.04	-1.28515	-1.46104	-1.50754
8.05	-1.30263	-1.49478	-1.53264
8.06	-1.32007	-1.52698	-1.55521
8.07	-1.33756	-1.5576	-1.57536
8.08	-1.35521	-1.58657	-1.5932
8.09	-1.37312	-1.6138	-1.60887
8.1	-1.39137	-1.6392	-1.62247
8.11	-1.41003	-1.6627	-1.63415
8.12	-1.429	-1.68422	-1.64408
8.13	-1.44815	-1.70368	-1.65245
8.14	-1.46735	-1.72099	-1.65948
8.15	-1.48644	-1.73607	-1.66534
8.16	-1.50531	-1.74888	-1.67023
8.17	-1.52381	-1.75958	-1.67436
8.18	-1.54182	-1.76834	-1.67791
8.19	-1.55918	-1.77536	-1.68108
8.2	-1.57577	-1.78082	-1.68407
8.21	-1.59148	-1.7849	-1.68704
8.22	-1.60629	-1.78781	-1.69003
8.23	-1.62023	-1.78973	-1.69303
8.24	-1.63331	-1.79083	-1.69607
8.25	-1.64555	-1.79132	-1.69914
8.26	-1.65696	-1.79134	-1.70225
8.27	-1.66755	-1.79083	-1.70539
8.28	-1.67735	-1.78973	-1.70859
8.29	-1.68637	-1.78794	-1.71184
8.3	-1.69463	-1.78538	-1.71514
8.31	-1.70213	-1.78196	-1.7185
8.32	-1.70893	-1.7776	-1.72188
8.33	-1.71503	-1.77222	-1.72525
8.34	-1.72048	-1.76571	-1.72857
8.35	-1.7253	-1.75801	-1.73179
8.36	-1.72952	-1.74906	-1.73488
8.37	-1.73318	-1.73893	-1.73779
8.38	-1.7363	-1.72771	-1.7405
8.39	-1.73892	-1.71552	-1.74296
8.4	-1.74106	-1.70244	-1.74514
8.41	-1.74274	-1.6886	-1.74698
8.42	-1.7439	-1.67407	-1.74844

8.43	-1.74447	-1.65898	-1.74946
8.44	-1.74438	-1.64341	-1.74998
8.45	-1.74355	-1.62747	-1.74994
8.46	-1.7419	-1.61126	-1.74929
8.47	-1.73937	-1.59482	-1.74797
8.48	-1.73588	-1.57822	-1.74591
8.49	-1.73136	-1.56149	-1.74307
8.5	-1.72574	-1.54469	-1.73938
8.51	-1.71894	-1.52787	-1.7348
8.52	-1.71097	-1.51107	-1.72926
8.53	-1.70181	-1.49434	-1.72274
8.54	-1.69147	-1.47773	-1.7152
8.55	-1.67994	-1.4613	-1.70658
8.56	-1.66722	-1.44507	-1.69684
8.57	-1.65331	-1.42904	-1.68595
8.58	-1.63821	-1.4132	-1.67386
8.59	-1.62191	-1.39752	-1.66054
8.6	-1.60442	-1.38198	-1.64593
8.61	-1.58573	-1.36658	-1.63002
8.62	-1.56583	-1.35128	-1.61289
8.63	-1.54471	-1.33607	-1.59465
8.64	-1.52236	-1.32094	-1.57542
8.65	-1.49877	-1.30586	-1.55528
8.66	-1.47394	-1.29081	-1.53435
8.67	-1.44784	-1.27576	-1.51275
8.68	-1.42047	-1.26066	-1.49056
8.69	-1.39182	-1.24547	-1.46791
8.7	-1.36188	-1.23015	-1.4449
8.71	-1.33062	-1.21465	-1.4216
8.72	-1.2979	-1.19895	-1.39793
8.73	-1.26356	-1.18298	-1.37378
8.74	-1.22745	-1.16672	-1.34903
8.75	-1.18942	-1.15011	-1.32357
8.76	-1.1493	-1.13311	-1.29728
8.77	-1.10695	-1.11559	-1.27006
8.78	-1.0622	-1.09741	-1.24179
8.79	-1.0149	-1.07844	-1.21235
8.8	-0.9649	-1.05855	-1.18162
8.81	-0.9121	-1.03759	-1.14953
8.82	-0.85671	-1.01542	-1.11606
8.83	-0.79901	-0.99192	-1.08124
8.84	-0.73926	-0.96695	-1.04508
8.85	-0.67775	-0.94037	-1.00761
8.86	-0.61474	-0.91205	-0.96884
8.87	-0.55051	-0.88193	-0.9288
8.88	-0.48534	-0.84996	-0.88751
8.89	-0.41949	-0.81608	-0.84499
8.9	-0.35324	-0.78023	-0.80125

8.91	-0.28685	-0.74236	-0.7563
8.92	-0.22046	-0.70242	-0.71007
8.93	-0.15422	-0.66035	-0.66247
8.94	-0.08825	-0.6161	-0.61341
8.95	-0.02268	-0.56961	-0.5628
8.96	0.04234	-0.52086	-0.51055
8.97	0.106692	-0.46996	-0.45658
8.98	0.170238	-0.41704	-0.40079
8.99	0.232845	-0.36225	-0.3431
9	0.294381	-0.30572	-0.28341
9.01	0.354727	-0.24758	-0.22171
9.02	0.413831	-0.18799	-0.15822
9.03	0.471653	-0.12707	-0.09324
9.04	0.528155	-0.06497	-0.02708
9.05	0.583297	-0.00182	0.039963
9.06	0.637042	0.078651	0.1076
9.07	0.689351	0.160552	0.175528
9.08	0.740185	0.243213	0.243448
9.09	0.789505	0.326437	0.31106
9.1	0.837274	0.41003	0.378068
9.11	0.883465	0.493799	0.444212
9.12	0.928112	0.577547	0.509398
9.13	0.971259	0.661082	0.573573
9.14	1.012953	0.744207	0.636682
9.15	1.053238	0.826729	0.698671
9.16	1.092161	0.908445	0.759486
9.17	1.129767	0.989123	0.819075
9.18	1.166102	1.068521	0.877382
9.19	1.201211	1.1464	0.934354
9.2	1.23514	1.222519	0.989938
9.21	1.267954	1.296637	1.044074
9.22	1.29979	1.368515	1.09669
9.23	1.330805	1.43791	1.147706
9.24	1.361155	1.504584	1.197044
9.25	1.390997	1.568295	1.244627
9.26	1.420488	1.628871	1.290376
9.27	1.449784	1.686417	1.334213
9.28	1.479042	1.741104	1.37606
9.29	1.508418	1.793104	1.415838
9.3	1.538069	1.842591	1.453469
9.31	1.568123	1.889735	1.488915
9.32	1.598587	1.93471	1.522302
9.33	1.629443	1.977687	1.553792
9.34	1.660669	2.01884	1.583552
9.35	1.692246	2.058339	1.611746
9.36	1.724154	2.096324	1.638538
9.37	1.756372	2.1328	1.664093
9.38	1.788879	2.167738	1.688577

9.39	1.821657	2.201108	1.712154
9.4	1.854684	2.232883	1.734988
9.41	1.887922	2.263032	1.75721
9.42	1.921259	2.291529	1.778816
9.43	1.954566	2.318343	1.799767
9.44	1.987711	2.343446	1.820024
9.45	2.020566	2.366809	1.839549
9.46	2.053	2.388421	1.858303
9.47	2.084883	2.408335	1.876248
9.48	2.116086	2.426623	1.893344
9.49	2.146477	2.443357	1.909553
9.5	2.175927	2.458609	1.924837
9.51	2.204322	2.472449	1.939173
9.52	2.231606	2.484949	1.952608
9.53	2.25774	2.49618	1.965206
9.54	2.282685	2.506215	1.977028
9.55	2.306401	2.515124	1.988139
9.56	2.328849	2.52296	1.998601
9.57	2.34999	2.529703	2.008478
9.58	2.369784	2.535312	2.017833
9.59	2.388192	2.539748	2.026728
9.6	2.405174	2.54297	2.035228
9.61	2.420692	2.54494	2.043369
9.62	2.43471	2.545617	2.051091
9.63	2.447194	2.544961	2.058307
9.64	2.458108	2.542933	2.064928
9.65	2.467418	2.539493	2.07087
9.66	2.475089	2.534633	2.076044
9.67	2.481085	2.528468	2.080365
9.68	2.485373	2.521145	2.083744
9.69	2.487918	2.512813	2.086096
9.7	2.488684	2.503618	2.087334
9.71	2.487643	2.493708	2.087394
9.72	2.484791	2.48323	2.086309
9.73	2.480131	2.472331	2.084135
9.74	2.473666	2.461158	2.08093
9.75	2.465398	2.449859	2.07675
9.76	2.455329	2.438543	2.071652
9.77	2.443462	2.427167	2.065691
9.78	2.429799	2.41565	2.058925
9.79	2.414344	2.40391	2.05141
9.8	2.397098	2.391867	2.043203
9.81	2.378053	2.37944	2.034343
9.82	2.35715	2.366547	2.024805
9.83	2.334319	2.353108	2.014544
9.84	2.309491	2.339041	2.003517
9.85	2.282597	2.324265	1.991681
9.86	2.253565	2.308706	1.978993

9.87	2.222327	2.292316	1.965409
9.88	2.188812	2.275054	1.950886
9.89	2.152952	2.256877	1.935381
9.9	2.114675	2.237745	1.91885
9.91	2.073934	2.217616	1.901252
9.92	2.030763	2.196449	1.88255
9.93	1.985219	2.174203	1.86271
9.94	1.937357	2.150836	1.841699
9.95	1.887235	2.126306	1.819482
9.96	1.834908	2.100551	1.796023
9.97	1.780433	2.073422	1.77129
9.98	1.723865	2.044747	1.745248
9.99	1.665262	2.014357	1.717863
10	1.604678	1.982081	1.689099
10.01	1.542202	1.947748	1.65891
10.02	1.478038	1.911187	1.62719
10.03	1.412425	1.872228	1.59382
10.04	1.345598	1.830701	1.558681
10.05	1.277796	1.786434	1.521655
10.06	1.209254	1.739302	1.482623
10.07	1.140209	1.689365	1.441466
10.08	1.070898	1.636728	1.398065
10.09	1.001559	1.581494	1.352301
10.1	0.932427	1.523769	1.304056
10.11	0.86372	1.463657	1.253253
10.12	0.795572	1.401263	1.199988
10.13	0.728098	1.336692	1.144399
10.14	0.661412	1.270048	1.086623
10.15	0.595629	1.201436	1.026799
10.16	0.530864	1.130974	0.965064
10.17	0.46723	1.058831	0.901558
10.18	0.404843	0.985192	0.836417
10.19	0.343816	0.910238	0.76978
10.2	0.284265	0.834155	0.701784
10.21	0.226291	0.757123	0.632575
10.22	0.16994	0.679328	0.562323
10.23	0.115244	0.600951	0.491207
10.24	0.062238	0.522176	0.419403
10.25	0.010952	0.443186	0.34709
10.26	-0.03858	0.364153	0.274444
10.27	-0.08632	0.285198	0.201645
10.28	-0.13224	0.20643	0.128869
10.29	-0.17631	0.127959	0.056294
10.3	-0.2185	0.049896	-0.0159
10.31	-0.25878	-0.02188	-0.08753
10.32	-0.29723	-0.08275	-0.15837
10.33	-0.33393	-0.14304	-0.22819
10.34	-0.36897	-0.20265	-0.29675

10.35	-0.40244	-0.26152	-0.36383
10.36	-0.43441	-0.31953	-0.42918
10.37	-0.46497	-0.37657	-0.49258
10.38	-0.49421	-0.43252	-0.55379
10.39	-0.52222	-0.48724	-0.61258
10.4	-0.54908	-0.54061	-0.66871
10.41	-0.57488	-0.59251	-0.72201
10.42	-0.5997	-0.6428	-0.77247
10.43	-0.62363	-0.69135	-0.82015
10.44	-0.64677	-0.73805	-0.8651
10.45	-0.6692	-0.78276	-0.90738
10.46	-0.69101	-0.8254	-0.94703
10.47	-0.71229	-0.86603	-0.98411
10.48	-0.73313	-0.90479	-1.01868
10.49	-0.75361	-0.94177	-1.05078
10.5	-0.77384	-0.9771	-1.08047
10.51	-0.79389	-1.0109	-1.10781
10.52	-0.81379	-1.04327	-1.1329
10.53	-0.83359	-1.07433	-1.15584
10.54	-0.85331	-1.10421	-1.17674
10.55	-0.873	-1.133	-1.19569
10.56	-0.89268	-1.1608	-1.21281
10.57	-0.91238	-1.18755	-1.22819
10.58	-0.93215	-1.21316	-1.24195
10.59	-0.95201	-1.23755	-1.25419
10.6	-0.972	-1.26063	-1.26501
10.61	-0.99212	-1.2823	-1.27452
10.62	-1.01226	-1.3025	-1.28285
10.63	-1.03227	-1.32111	-1.29013
10.64	-1.05201	-1.33807	-1.29648
10.65	-1.07133	-1.35328	-1.30205
10.66	-1.0901	-1.36668	-1.30695
10.67	-1.10816	-1.37835	-1.31133
10.68	-1.12538	-1.38838	-1.31531
10.69	-1.14161	-1.39688	-1.31903
10.7	-1.1567	-1.40394	-1.32261
10.71	-1.17054	-1.40966	-1.32616
10.72	-1.18316	-1.41415	-1.32971
10.73	-1.19461	-1.4175	-1.33327
10.74	-1.20493	-1.41981	-1.33682
10.75	-1.21417	-1.42119	-1.34039
10.76	-1.2224	-1.42171	-1.34396
10.77	-1.22965	-1.42136	-1.34755
10.78	-1.23598	-1.42009	-1.35116
10.79	-1.24145	-1.41789	-1.35478
10.8	-1.24609	-1.4147	-1.35843
10.81	-1.24996	-1.41049	-1.36209
10.82	-1.25303	-1.40524	-1.3657

10.83	-1.25527	-1.39889	-1.36916
10.84	-1.25665	-1.39141	-1.3724
10.85	-1.25714	-1.38278	-1.37533
10.86	-1.2567	-1.37296	-1.37786
10.87	-1.25532	-1.36201	-1.37993
10.88	-1.25294	-1.34999	-1.38144
10.89	-1.24956	-1.33697	-1.3823
10.9	-1.24512	-1.323	-1.38244
10.91	-1.2396	-1.30816	-1.38178
10.92	-1.2329	-1.29251	-1.38026
10.93	-1.22492	-1.27611	-1.37784
10.94	-1.21557	-1.25903	-1.37447
10.95	-1.20475	-1.24134	-1.3701
10.96	-1.19235	-1.22308	-1.36469
10.97	-1.17828	-1.2043	-1.35819
10.98	-1.16245	-1.18502	-1.35055
10.99	-1.14474	-1.16528	-1.34173
11	-1.12506	-1.1451	-1.33167
11.01	-1.10334	-1.12451	-1.32034
11.02	-1.07953	-1.10355	-1.30773
11.03	-1.05364	-1.08223	-1.29384
11.04	-1.02565	-1.0606	-1.27867
11.05	-0.99556	-1.03867	-1.26221
11.06	-0.96334	-1.01647	-1.24448
11.07	-0.929	-0.99399	-1.22546
11.08	-0.89252	-0.9712	-1.20515
11.09	-0.8539	-0.94809	-1.18356
11.1	-0.81311	-0.92462	-1.16069
11.11	-0.77021	-0.90077	-1.13653
11.12	-0.7254	-0.87653	-1.11112
11.13	-0.67897	-0.85187	-1.08448
11.14	-0.63118	-0.82677	-1.05664
11.15	-0.5823	-0.80121	-1.02764
11.16	-0.5326	-0.77516	-0.99749
11.17	-0.48236	-0.74859	-0.96623
11.18	-0.43183	-0.72147	-0.9339
11.19	-0.38129	-0.69377	-0.90051
11.2	-0.33102	-0.66546	-0.86609
11.21	-0.28127	-0.6365	-0.83069
11.22	-0.23229	-0.60688	-0.79432
11.23	-0.18433	-0.57654	-0.75704
11.24	-0.13762	-0.54547	-0.71886
11.25	-0.09241	-0.51363	-0.67983
11.26	-0.04895	-0.481	-0.63998
11.27	-0.00746	-0.44764	-0.59934
11.28	0.031801	-0.41363	-0.55795
11.29	0.068595	-0.37902	-0.51585
11.3	0.102681	-0.34389	-0.47307

11.31	0.133863	-0.30831	-0.42966
11.32	0.162136	-0.27234	-0.38576
11.33	0.187541	-0.23607	-0.34152
11.34	0.21012	-0.19955	-0.29711
11.35	0.229914	-0.06514	-0.25267
11.36	0.246965	-0.05043	-0.20836
11.37	0.261314	-0.03573	-0.16434
11.38	0.273002	-0.0211	-0.12076
11.39	0.282071	-0.00659	-0.07777
11.4	0.288562	0.009792	-0.03554
11.41	0.292542	0.027613	0.005792
11.42	0.294182	0.045072	0.046134
11.43	0.293675	0.062101	0.085395
11.44	0.291216	0.078631	0.123489
11.45	0.287001	0.094592	0.160329
11.46	0.281224	0.109923	0.19583
11.47	0.274081	0.124588	0.229904
11.48	0.265766	0.138561	0.262466
11.49	0.256474	0.151812	0.293428
11.5	0.246401	0.164314	0.322705
11.51	0.235731	0.17604	0.350227
11.52	0.224616	0.186961	0.375996
11.53	0.213195	0.19705	0.400031
11.54	0.201609	0.206278	0.422352
11.55	0.189998	0.214618	0.442978
11.56	0.178504	0.222053	0.461927
11.57	0.167268	0.228612	0.47922
11.58	0.156429	0.234335	0.494875
11.59	0.146128	0.239261	0.508912
11.6	0.136507	0.24343	0.521349
11.61	0.127672	0.246883	0.532219
11.62	0.119601	0.249659	0.541598
11.63	0.112236	0.251799	0.549577
11.64	0.105522	0.253341	0.556246
11.65	0.099402	0.254325	0.561694
11.66	0.09382	0.254793	0.566013
11.67	0.088718	0.254785	0.56929
11.68	0.084041	0.254341	0.571618
11.69	0.079732	0.253504	0.573084
11.7	0.075735	0.252315	0.57378
11.71	0.072003	0.250814	0.573791
11.72	0.068522	0.249043	0.57319
11.73	0.065291	0.247043	0.572045
11.74	0.062306	0.244855	0.570424
11.75	0.059565	0.242521	0.568396
11.76	0.057063	0.240076	0.566028
11.77	0.054799	0.237532	0.56339
11.78	0.05277	0.234898	0.56055

11.79	0.050971	0.232178	0.557576
11.8	0.049401	0.229382	0.554537
11.81	0.048053	0.226515	0.551478
11.82	0.046902	0.223585	0.548364
11.83	0.045922	0.220599	0.545133
11.84	0.045085	0.217564	0.541727
11.85	0.044364	0.214486	0.538085
11.86	0.043731	0.21137	0.534149
11.87	0.04316	0.208206	0.529859
11.88	0.042622	0.204983	0.525155
11.89	0.042091	0.201686	0.519978
11.9	0.041539	0.198305	0.514268
11.91	0.040947	0.194827	0.507981
11.92	0.040326	0.191239	0.501123
11.93	0.039696	0.187529	0.493718
11.94	0.039076	0.183685	0.485788
11.95	0.038487	0.179693	0.477355
11.96	0.037948	0.175548	0.468441
11.97	0.037479	0.171262	0.459069
11.98	0.037099	0.166853	0.449261
11.99	0.036828	0.16234	0.439039
12	0.036685	0.157741	0.428427
12.01	0.036685	0.153074	0.417449
12.02	0.036813	0.148359	0.406151
12.03	0.03705	0.143612	0.394581
12.04	0.037378	0.138853	0.382788
12.05	0.037775	0.134099	0.370821
12.06	0.038224	0.129367	0.358729
12.07	0.038704	0.124655	0.346559
12.08	0.039197	0.119963	0.334361
12.09	0.039681	0.115286	0.322184
12.1	0.040139	0.110623	0.310076
12.11	0.040555	0.10597	0.29808
12.12	0.04093	0.101326	0.286213
12.13	0.04127	0.096687	0.274487
12.14	0.041579	0.09205	0.262915
12.15	0.041865	0.087414	0.251507
12.16	0.042132	0.082779	0.240276
12.17	0.042386	0.07817	0.229233
12.18	0.042633	0.073612	0.218389
12.19	0.042877	0.069134	0.207758
12.2	0.043125	0.064763	0.19735
12.21	0.043381	0.060526	0.187178
12.22	0.043641	0.05645	0.17726
12.23	0.043902	0.052564	0.167616
12.24	0.044158	0.048894	0.158264
12.25	0.044404	0.045468	0.149223
12.26	0.044638	0.042307	0.140514

12.27	0.044853	0.039408	0.132154
12.28	0.045046	0.036764	0.124163
12.29	0.045212	0.034365	0.11656
12.3	0.045346	0.032203	0.109364
12.31	0.045446	0.030268	0.102587
12.32	0.045518	0.028553	0.096214
12.33	0.045571	0.027049	0.090222
12.34	0.045612	0.025747	0.084588
12.35	0.045651	0.024638	0.079288
12.36	0.045696	0.023715	0.074301
12.37	0.045754	0.022973	0.069603
12.38	0.045835	0.022409	0.065172
12.39	0.045946	0.022017	0.060985
12.4	0.046096	0.021794	0.057018
12.41	0.046291	0.021737	0.05326
12.42	0.046523	0.021841	0.049733
12.43	0.046784	0.022102	0.046473
12.44	0.047063	0.022517	0.043514
12.45	0.047351	0.023081	0.040889
12.46	0.047639	0.02379	0.038633
12.47	0.047916	0.024629	0.03678
12.48	0.048174	0.025583	0.035364
12.49	0.048403	0.026638	0.034418
12.5	0.048593	0.027779	0.033978
12.51	0.048736	0.02899	0.034065
12.52	0.048834	0.030257	0.034652
12.53	0.048887	0.031565	0.035701
12.54	0.0489	0.032897	0.037172
12.55	0.048875	0.034241	0.039027
12.56	0.048813	0.03558	0.041227
12.57	0.048717	0.036909	0.043732
12.58	0.04859	0.038218	0.046505
12.59	0.048433	0.039502	0.049505
12.6	0.048251	0.040752	0.052695
12.61	0.048045	0.041963	0.056037
12.62	0.047821	0.043126	0.059502
12.63	0.047585	0.044235	0.063063
12.64	0.047343	0.045282	0.066692
12.65	0.047102	0.046261	0.070363
12.66	0.046867	0.047167	0.074049
12.67	0.046644	0.048002	0.077721
12.68	0.04644	0.048771	0.081353
12.69	0.046261	0.049481	0.084918
12.7	0.046112	0.050135	0.088389
12.71	0.045999	0.050739	0.091742
12.72	0.04592	0.051298	0.094972
12.73	0.045874	0.051818	0.098078
12.74	0.045857	0.052302	0.101058

12.75	0.045868	0.052757	0.10391
12.76	0.045904	0.053187	0.106635
12.77	0.045963	0.053593	0.109229
12.78	0.046043	0.053973	0.111692
12.79	0.046141	0.054329	0.114023
12.8	0.046255	0.054659	0.11622
12.81	0.046382	0.054964	0.118282
12.82	0.046523	0.055244	0.120215
12.83	0.046675	0.055499	0.122023
12.84	0.046837	0.055728	0.123712
12.85	0.047008	0.055931	0.125288
12.86	0.047187	0.056108	0.126756
12.87	0.047373	0.056263	0.128121
12.88	0.047564	0.056397	0.129389
12.89	0.047761	0.056514	0.130566
12.9	0.04796	0.056616	0.131656
12.91	0.048162	0.056706	0.132665
12.92	0.048368	0.056788	0.133597
12.93	0.048578	0.056863	0.134456
12.94	0.048794	0.056935	0.135248
12.95	0.049018	0.057007	0.135975
12.96	0.049249	0.05708	0.136641
12.97	0.04949	0.057156	0.137252
12.98	0.049742	0.057233	0.137811
12.99	0.050006	0.057312	0.138322
13	0.050283	0.057391	0.13879
13.01	0.050573	0.057472	0.139218
13.02	0.050874	0.057553	0.13961
13.03	0.05118	0.057635	0.139971
13.04	0.051487	0.057716	0.140305
13.05	0.051791	0.057797	0.140614
13.06	0.052088	0.057877	0.140904
13.07	0.052373	0.057956	0.141178
13.08	0.052641	0.058034	0.141439
13.09	0.052889	0.058111	0.141693
13.1	0.053112	0.058187	0.141943
13.11	0.053307	0.058261	0.142192
13.12	0.053477	0.058334	0.142439
13.13	0.053625	0.058405	0.142684
13.14	0.053757	0.058474	0.142926
13.15	0.053876	0.058541	0.143163
13.16	0.053987	0.058606	0.143395
13.17	0.054093	0.058669	0.143621
13.18	0.0542	0.05873	0.143839
13.19	0.054311	0.058791	0.144048
13.2	0.05443	0.05885	0.144247
13.21	0.05456	0.058909	0.144437
13.22	0.054703	0.058968	0.144617

13.23	0.054858	0.059028	0.144788
13.24	0.055025	0.059088	0.144953
13.25	0.055203	0.059149	0.145113
13.26	0.055391	0.059211	0.145268
13.27	0.055591	0.059275	0.14542
13.28	0.055801	0.05934	0.14557
13.29	0.056021	0.059407	0.14572
13.3	0.05625	0.059476	0.145871
13.31	0.056489	0.059547	0.146024
13.32	0.056734	0.059619	0.146178
13.33	0.056983	0.059694	0.146333
13.34	0.057232	0.059771	0.146489
13.35	0.057479	0.05985	0.146645
13.36	0.05772	0.059931	0.1468
13.37	0.057952	0.060015	0.146954
13.38	0.058174	0.060102	0.147107
13.39	0.05838	0.060191	0.147257
13.4	0.058569	0.060284	0.147404
13.41	0.058739	0.060381	0.147549
13.42	0.058893	0.060481	0.147691
13.43	0.059035	0.060584	0.147833
13.44	0.059171	0.060692	0.147976
13.45	0.059303	0.060805	0.148122
13.46	0.059437	0.060921	0.148272
13.47	0.059577	0.061042	0.148427
13.48	0.059728	0.061166	0.14859
13.49	0.059893	0.061294	0.14876
13.5	0.060078	0.061425	0.148941
13.51	0.060286	0.061558	0.149133
13.52	0.060513	0.061694	0.149335
13.53	0.060757	0.061831	0.149548
13.54	0.061014	0.06197	0.14977
13.55	0.061281	0.062109	0.15
13.56	0.061553	0.062249	0.150238
13.57	0.061827	0.06239	0.150484
13.58	0.062099	0.062529	0.150736
13.59	0.062367	0.062668	0.150993
13.6	0.062625	0.062805	0.151256
13.61	0.062871	0.062939	0.151523
13.62	0.063103	0.063071	0.151793
13.63	0.063318	0.063199	0.152065
13.64	0.063514	0.063323	0.152339
13.65	0.06369	0.063443	0.152612
13.66	0.063843	0.063557	0.152885
13.67	0.06397	0.063666	0.153155
13.68	0.064071	0.063769	0.153422
13.69	0.064143	0.063866	0.153685
13.7	0.064184	0.063957	0.153943

13.71	0.064193	0.064041	0.154194
13.72	0.064173	0.064117	0.154438
13.73	0.064127	0.064186	0.154671
13.74	0.06406	0.064248	0.154892
13.75	0.063977	0.064301	0.155099
13.76	0.06388	0.064346	0.155291
13.77	0.063775	0.064384	0.155465
13.78	0.063664	0.064413	0.15562
13.79	0.063552	0.064435	0.155754
13.8	0.063444	0.06445	0.155864
13.81	0.063342	0.064458	0.155951
13.82	0.063249	0.06446	0.156013
13.83	0.063168	0.064455	0.156052
13.84	0.063099	0.064445	0.156069
13.85	0.063045	0.064429	0.156065
13.86	0.063008	0.064409	0.156041
13.87	0.06299	0.064384	0.155998
13.88	0.062993	0.064355	0.155936
13.89	0.063018	0.064322	0.155857
13.9	0.063069	0.064288	0.155761
13.91	0.063145	0.064251	0.15565
13.92	0.063244	0.064213	0.155525
13.93	0.063364	0.064174	0.155388
13.94	0.063502	0.064135	0.155241
13.95	0.063654	0.064097	0.155086
13.96	0.063818	0.06406	0.154924
13.97	0.06399	0.064024	0.154758
13.98	0.064167	0.06399	0.154589
13.99	0.064346	0.063958	0.154419
14	0.064525	0.063929	0.154249
14.01	0.0647	0.063903	0.154082
14.02	0.06487	0.063881	0.153919
14.03	0.065035	0.063862	0.15376
14.04	0.065193	0.063847	0.153606
14.05	0.065343	0.063836	0.153458
14.06	0.065484	0.06383	0.153317
14.07	0.065616	0.063828	0.153184
14.08	0.065737	0.06383	0.153059
14.09	0.065847	0.063835	0.152944
14.1	0.065943	0.063843	0.15284
14.11	0.066027	0.063852	0.152746
14.12	0.066098	0.063863	0.152663
14.13	0.066157	0.063875	0.152589
14.14	0.066205	0.063887	0.152524
14.15	0.066243	0.063899	0.152466
14.16	0.06627	0.06391	0.152415
14.17	0.066289	0.063919	0.15237
14.18	0.0663	0.063928	0.15233

14.19	0.066303	0.063934	0.152295
14.2	0.0663	0.063937	0.152262
14.21	0.066291	0.063938	0.152232
14.22	0.066277	0.063935	0.152203
14.23	0.066258	0.063928	0.152173
14.24	0.066235	0.063918	0.152143
14.25	0.066209	0.063902	0.15211
14.26	0.066179	0.063882	0.152073
14.27	0.066148	0.063857	0.152031
14.28	0.066114	0.063828	0.151983
14.29	0.06608	0.063796	0.151928
14.3	0.066045	0.063762	0.151864
14.31	0.06601	0.063725	0.151791
14.32	0.065975	0.063686	0.151709
14.33	0.065941	0.063647	0.15162
14.34	0.065907	0.063608	0.151525
14.35	0.065874	0.063568	0.151425
14.36	0.065843	0.06353	0.151322
14.37	0.065813	0.063493	0.151218
14.38	0.065785	0.063458	0.151113
14.39	0.065758	0.063426	0.151008
14.4	0.065734	0.063397	0.150906
14.41	0.065712	0.063372	0.150807
14.42	0.065693	0.063352	0.150712
14.43	0.065677	0.063336	0.150624
14.44	0.065663	0.063326	0.150543
14.45	0.065654	0.063322	0.150471
14.46	0.065647	0.063372	0.150409
14.47	0.065644	0.063352	0.150358
14.48	0.065646	0.063336	0.150319
14.49	0.065652	0.063326	0.150295
14.5	0.065662	0.063322	0.150286

Table S9. Test Result Data of the Double Lane Change Test at an Adhesion Coefficient of 0.2

Time	carsim	DRL-UKF	UKF
0	#######	-0.02569	0.040666
0.01	-0.00413	-0.02493	0.041363
0.02	-0.00738	-0.02409	0.042141
0.03	-0.00979	-0.02316	0.043001
0.04	-0.01141	-0.02214	0.043949
0.05	-0.0123	-0.02101	0.044985
0.06	-0.01251	-0.01979	0.046113

0.07	-0.01208	-0.01847	0.047335
0.08	-0.01106	-0.01704	0.048655
0.09	-0.00951	-0.01551	0.050076
0.1	-0.00747	-0.01386	0.0516
0.11	-0.005	-0.01209	0.053229
0.12	-0.00214	-0.01021	0.054968
0.13	0.001055	-0.00821	0.056818
0.14	0.004535	-0.00609	0.058783
0.15	0.008252	-0.00383	0.060866
0.16	0.012154	-0.00145	0.063068
0.17	0.016193	0.001067	0.065394
0.18	0.020319	0.00372	0.067846
0.19	0.02448	0.006512	0.070426
0.2	0.028628	0.009446	0.073138
0.21	0.032718	0.01252	0.075979
0.22	0.036727	0.015705	0.078923
0.23	0.040637	0.018969	0.081939
0.24	0.04443	0.022276	0.084995
0.25	0.04809	0.025593	0.088061
0.26	0.051597	0.028886	0.091104
0.27	0.054935	0.032121	0.094095
0.28	0.058086	0.035264	0.097
0.29	0.061031	0.038282	0.099789
0.3	0.063754	0.04114	0.102432
0.31	0.066242	0.043811	0.104901
0.32	0.068497	0.046292	0.107194
0.33	0.07053	0.048584	0.109313
0.34	0.072348	0.050691	0.111261
0.35	0.07396	0.052615	0.113039
0.36	0.075375	0.054359	0.114651
0.37	0.076602	0.055925	0.116099
0.38	0.077648	0.057317	0.117385
0.39	0.078524	0.058537	0.118512
0.4	0.079236	0.059587	0.119481
0.41	0.079795	0.060472	0.120297
0.42	0.080207	0.0612	0.120968
0.43	0.080479	0.06178	0.121501
0.44	0.080619	0.062222	0.121908
0.45	0.080635	0.062535	0.122196
0.46	0.080532	0.062729	0.122374
0.47	0.08032	0.062813	0.122452
0.48	0.080005	0.062797	0.122439
0.49	0.079594	0.06269	0.122343
0.5	0.079095	0.062502	0.122175
0.51	0.078516	0.062241	0.121941
0.52	0.077868	0.061915	0.121648
0.53	0.077164	0.061529	0.1213
0.54	0.076417	0.061089	0.120901

0.55	0.07564	0.060601	0.120455
0.56	0.074844	0.060071	0.119968
0.57	0.074042	0.059505	0.119444
0.58	0.073247	0.058909	0.118886
0.59	0.072471	0.058289	0.118301
0.6	0.071728	0.05765	0.117691
0.61	0.071026	0.057	0.117062
0.62	0.070363	0.056341	0.116421
0.63	0.069737	0.055677	0.115776
0.64	0.069141	0.055012	0.115132
0.65	0.068572	0.054349	0.114499
0.66	0.068024	0.053693	0.113883
0.67	0.067494	0.053047	0.113291
0.68	0.066977	0.052414	0.11273
0.69	0.066469	0.051799	0.112209
0.7	0.065964	0.051205	0.111734
0.71	0.06546	0.050635	0.111309
0.72	0.064959	0.05009	0.110923
0.73	0.064464	0.049569	0.110561
0.74	0.063979	0.049072	0.110208
0.75	0.063506	0.048598	0.10985
0.76	0.063051	0.048149	0.109472
0.77	0.062615	0.047723	0.109059
0.78	0.062203	0.04732	0.108596
0.79	0.061818	0.04694	0.108069
0.8	0.061463	0.046583	0.107462
0.81	0.06114	0.046248	0.106772
0.82	0.060847	0.045929	0.106037
0.83	0.060579	0.045621	0.105306
0.84	0.060333	0.045318	0.104627
0.85	0.060105	0.045015	0.104051
0.86	0.05989	0.044705	0.103625
0.87	0.059684	0.044382	0.103399
0.88	0.059484	0.044041	0.103422
0.89	0.059284	0.043675	0.103742
0.9	0.059082	0.04328	0.104409
0.91	0.058875	0.042851	0.105456
0.92	0.058661	0.042398	0.106849
0.93	0.058444	0.041932	0.108542
0.94	0.058224	0.041463	0.110485
0.95	0.058003	0.041003	0.112631
0.96	0.057782	0.040564	0.114931
0.97	0.057562	0.040157	0.117337
0.98	0.057346	0.039792	0.1198
0.99	0.057134	0.039481	0.122273
1	0.056927	0.039236	0.124706
1.01	0.056728	0.039064	0.127064
1.02	0.056533	0.038961	0.129355

1.03	0.056342	0.038916	0.131602
1.04	0.056152	0.038923	0.133825
1.05	0.055962	0.038973	0.136046
1.06	0.05577	0.039056	0.138286
1.07	0.055573	0.039166	0.140566
1.08	0.055369	0.039292	0.142908
1.09	0.055158	0.039426	0.145332
1.1	0.054936	0.039561	0.14786
1.11	0.054703	0.039689	0.150501
1.12	0.054457	0.039813	0.153215
1.13	0.054197	0.039937	0.155949
1.14	0.053922	0.040067	0.158652
1.15	0.053632	0.040206	0.16127
1.16	0.053326	0.040359	0.163752
1.17	0.053003	0.040531	0.166044
1.18	0.052663	0.040726	0.168096
1.19	0.052303	0.040949	0.169854
1.2	0.051925	0.041205	0.171265
1.21	0.051523	0.041492	0.172294
1.22	0.051087	0.041797	0.172963
1.23	0.050602	0.042098	0.173312
1.24	0.050053	0.042375	0.173379
1.25	0.049425	0.042609	0.173203
1.26	0.048704	0.042778	0.172823
1.27	0.047875	0.042862	0.172279
1.28	0.046925	0.042842	0.171608
1.29	0.045837	0.042697	0.17085
1.3	0.044598	0.042408	0.170045
1.31	0.043194	0.041955	0.169219
1.32	0.041608	0.041331	0.168365
1.33	0.039828	0.04053	0.16746
1.34	0.037838	0.039547	0.166485
1.35	0.035623	0.038375	0.16542
1.36	0.03317	0.037008	0.164244
1.37	0.030462	0.035442	0.162937
1.38	0.027487	0.033669	0.161478
1.39	0.024229	0.031684	0.159848
1.4	0.020673	0.029481	0.158025
1.41	0.01681	0.027055	0.155994
1.42	0.012648	0.024396	0.153751
1.43	0.0082	0.021496	0.151298
1.44	0.003481	0.018346	0.148634
1.45	-0.0015	0.014937	0.145762
1.46	-0.00672	0.011262	0.142681
1.47	-0.01218	0.00731	0.139392
1.48	-0.01785	0.003074	0.135897
1.49	-0.02373	-0.00146	0.132196
1.5	-0.0298	-0.00629	0.128289

1.51	-0.03606	-0.01143	0.124179
1.52	-0.04247	-0.01686	0.119867
1.53	-0.04903	-0.02258	0.115354
1.54	-0.05571	-0.02856	0.110644
1.55	-0.06251	-0.0348	0.105738
1.56	-0.0694	-0.04128	0.100638
1.57	-0.07637	-0.04798	0.095348
1.58	-0.0834	-0.05489	0.089868
1.59	-0.09048	-0.06199	0.084201
1.6	-0.09758	-0.06928	0.078349
1.61	-0.1047	-0.07673	0.072315
1.62	-0.11188	-0.08435	0.066103
1.63	-0.11916	-0.09212	0.05972
1.64	-0.12658	-0.10005	0.053169
1.65	-0.1342	-0.10813	0.046457
1.66	-0.14204	-0.11635	0.039589
1.67	-0.15017	-0.12471	0.032571
1.68	-0.15862	-0.13321	0.025406
1.69	-0.16744	-0.14185	0.018102
1.7	-0.17667	-0.15061	0.010662
1.71	-0.18636	-0.1595	0.003088
1.72	-0.19654	-0.16856	-0.00464
1.73	-0.20727	-0.17782	-0.01255
1.74	-0.21858	-0.18732	-0.02066
1.75	-0.23051	-0.1971	-0.02901
1.76	-0.2431	-0.2072	-0.0376
1.77	-0.2564	-0.21766	-0.04648
1.78	-0.27045	-0.22852	-0.05566
1.79	-0.28528	-0.23981	-0.06518
1.8	-0.30094	-0.25158	-0.07505
1.81	-0.31745	-0.26386	-0.0853
1.82	-0.33478	-0.27671	-0.09595
1.83	-0.35287	-0.29017	-0.10703
1.84	-0.37168	-0.30428	-0.11856
1.85	-0.39114	-0.31909	-0.13057
1.86	-0.41122	-0.33466	-0.14308
1.87	-0.43185	-0.35102	-0.15611
1.88	-0.45299	-0.36822	-0.16969
1.89	-0.47458	-0.38632	-0.18384
1.9	-0.49657	-0.40535	-0.19859
1.91	-0.51891	-0.42536	-0.21396
1.92	-0.54159	-0.44637	-0.22999
1.93	-0.56458	-0.46838	-0.24672
1.94	-0.58787	-0.49139	-0.2642
1.95	-0.61145	-0.51542	-0.28248
1.96	-0.63529	-0.54048	-0.30159
1.97	-0.65938	-0.56657	-0.32158
1.98	-0.6837	-0.59369	-0.34249

1.99	-0.70824	-0.62187	-0.36437
2	-0.73298	-0.65111	-0.38725
2.01	-0.7579	-0.68138	-0.41116
2.02	-0.78294	-0.71262	-0.43605
2.03	-0.80806	-0.7447	-0.46184
2.04	-0.83319	-0.77753	-0.48844
2.05	-0.85829	-0.81099	-0.51579
2.06	-0.88329	-0.84498	-0.54379
2.07	-0.90815	-0.87939	-0.57239
2.08	-0.9328	-0.91411	-0.60149
2.09	-0.95719	-0.94904	-0.63103
2.1	-0.98126	-0.98406	-0.66091
2.11	-1.00497	-1.01908	-0.69108
2.12	-1.02827	-1.054	-0.72144
2.13	-1.05111	-1.08876	-0.7519
2.14	-1.07346	-1.12327	-0.7824
2.15	-1.09526	-1.15745	-0.81284
2.16	-1.11648	-1.19121	-0.84315
2.17	-1.13708	-1.22448	-0.87323
2.18	-1.157	-1.25719	-0.90301
2.19	-1.17622	-1.28924	-0.93241
2.2	-1.19468	-1.32056	-0.96134
2.21	-1.21234	-1.35107	-0.98973
2.22	-1.22921	-1.38072	-1.01751
2.23	-1.24526	-1.40947	-1.04466
2.24	-1.26049	-1.43726	-1.07111
2.25	-1.27489	-1.46405	-1.09681
2.26	-1.28846	-1.48978	-1.12174
2.27	-1.30118	-1.51443	-1.14582
2.28	-1.31305	-1.53792	-1.16903
2.29	-1.32406	-1.56023	-1.1913
2.3	-1.3342	-1.58129	-1.2126
2.31	-1.34347	-1.60108	-1.23287
2.32	-1.35187	-1.6196	-1.25204
2.33	-1.3594	-1.63685	-1.27006
2.34	-1.36608	-1.65285	-1.28686
2.35	-1.3719	-1.66762	-1.30237
2.36	-1.37688	-1.68117	-1.31653
2.37	-1.38101	-1.69351	-1.32926
2.38	-1.38431	-1.70466	-1.34051
2.39	-1.38677	-1.71462	-1.35021
2.4	-1.38841	-1.72342	-1.35828
2.41	-1.38923	-1.73106	-1.3647
2.42	-1.38923	-1.73755	-1.36949
2.43	-1.38841	-1.74292	-1.37273
2.44	-1.38678	-1.74717	-1.37448
2.45	-1.38433	-1.75031	-1.37481
2.46	-1.38106	-1.75236	-1.37377

2.47	-1.37699	-1.75333	-1.37144
2.48	-1.3721	-1.75323	-1.36788
2.49	-1.36641	-1.75208	-1.36315
2.5	-1.3599	-1.74988	-1.35732
2.51	-1.3526	-1.74665	-1.35044
2.52	-1.34451	-1.74237	-1.3425
2.53	-1.33567	-1.73701	-1.33348
2.54	-1.32609	-1.73054	-1.32336
2.55	-1.31582	-1.72294	-1.31212
2.56	-1.30486	-1.7142	-1.29974
2.57	-1.29325	-1.70428	-1.2862
2.58	-1.28101	-1.69315	-1.27148
2.59	-1.26817	-1.6808	-1.25555
2.6	-1.25475	-1.6672	-1.23839
2.61	-1.24075	-1.65232	-1.22001
2.62	-1.22606	-1.6361	-1.20046
2.63	-1.21054	-1.61849	-1.17983
2.64	-1.19405	-1.59942	-1.1582
2.65	-1.17645	-1.57884	-1.13564
2.66	-1.15761	-1.55668	-1.11224
2.67	-1.13738	-1.53289	-1.08807
2.68	-1.11562	-1.5074	-1.06323
2.69	-1.09219	-1.48016	-1.03778
2.7	-1.06696	-1.4511	-1.01182
2.71	-1.0398	-1.42018	-0.98538
2.72	-1.01062	-1.38742	-0.95839
2.73	-0.97934	-1.35284	-0.93073
2.74	-0.94588	-1.31649	-0.90229
2.75	-0.91018	-1.27839	-0.87295
2.76	-0.87216	-1.23857	-0.84261
2.77	-0.83173	-1.19707	-0.81114
2.78	-0.78883	-1.15392	-0.77844
2.79	-0.74337	-1.10914	-0.74438
2.8	-0.69528	-1.06278	-0.70885
2.81	-0.64455	-1.01487	-0.67179
2.82	-0.59146	-0.96547	-0.63322
2.83	-0.53633	-0.91464	-0.59325
2.84	-0.4795	-0.86245	-0.55196
2.85	-0.42131	-0.80897	-0.50943
2.86	-0.36209	-0.75424	-0.46574
2.87	-0.30217	-0.69835	-0.421
2.88	-0.2419	-0.64136	-0.37527
2.89	-0.18161	-0.58332	-0.32865
2.9	-0.12163	-0.52431	-0.28122
2.91	-0.06224	-0.46439	-0.23305
2.92	-0.00351	-0.40365	-0.18417
2.93	0.05457	-0.3422	-0.13459
2.94	0.111997	-0.28011	-0.08432

2.95	0.168768	-0.21749	-0.03336
2.96	0.224881	-0.15444	0.018264
2.97	0.280335	-0.09104	0.070551
2.98	0.335128	-0.02739	0.123488
2.99	0.389258	0.036413	0.177066
3	0.442725	0.100275	0.231273
3.01	0.495519	0.164107	0.28607
3.02	0.547611	0.227841	0.341296
3.03	0.598962	0.291414	0.396761
3.04	0.649538	0.354764	0.452275
3.05	0.699299	0.417829	0.507647
3.06	0.74821	0.480545	0.562687
3.07	0.796234	0.542849	0.617205
3.08	0.843333	0.604681	0.671011
3.09	0.889471	0.665976	0.723915
3.1	0.934611	0.726672	0.775726
3.11	0.978718	0.786701	0.826294
3.12	1.021765	0.845974	0.875634
3.13	1.063729	0.904395	0.923799
3.14	1.104585	0.961869	0.970845
3.15	1.14431	1.018303	1.016825
3.16	1.182879	1.073599	1.061794
3.17	1.220269	1.127664	1.105806
3.18	1.256454	1.180403	1.148915
3.19	1.291412	1.23172	1.191176
3.2	1.325117	1.281521	1.232644
3.21	1.357549	1.329721	1.273339
3.22	1.388701	1.37628	1.313156
3.23	1.418566	1.42117	1.351955
3.24	1.447141	1.46436	1.389597
3.25	1.474419	1.505821	1.425941
3.26	1.500398	1.545526	1.46085
3.27	1.52507	1.583443	1.494183
3.28	1.548433	1.619544	1.525801
3.29	1.57048	1.653799	1.555564
3.3	1.591207	1.68618	1.583335
3.31	1.61061	1.716656	1.609001
3.32	1.628685	1.745192	1.632568
3.33	1.645429	1.771753	1.654069
3.34	1.66084	1.796302	1.673538
3.35	1.674914	1.818804	1.691009
3.36	1.687649	1.839222	1.706514
3.37	1.699042	1.857522	1.720089
3.38	1.70909	1.873667	1.731766
3.39	1.717791	1.887622	1.74158
3.4	1.72514	1.89935	1.749563
3.41	1.731133	1.908827	1.755725
3.42	1.735751	1.91608	1.759984

3.43	1.738972	1.921146	1.76223
3.44	1.740773	1.924062	1.762357
3.45	1.741134	1.924866	1.760256
3.46	1.740032	1.923596	1.75582
3.47	1.737446	1.920289	1.748941
3.48	1.733353	1.914982	1.739512
3.49	1.727732	1.907714	1.727424
3.5	1.720561	1.898522	1.71257
3.51	1.711822	1.887425	1.694886
3.52	1.701514	1.874367	1.674482
3.53	1.689639	1.859277	1.65151
3.54	1.676199	1.842079	1.626123
3.55	1.661197	1.822701	1.598476
3.56	1.644635	1.80107	1.568719
3.57	1.626516	1.777111	1.537008
3.58	1.606841	1.750751	1.503495
3.59	1.585614	1.721918	1.468333
3.6	1.562837	1.690537	1.431674
3.61	1.538519	1.656577	1.393667
3.62	1.512702	1.620176	1.354429
3.63	1.485435	1.581514	1.314074
3.64	1.456765	1.54077	1.272716
3.65	1.426743	1.498125	1.230466
3.66	1.395416	1.453757	1.187438
3.67	1.362832	1.407849	1.143745
3.68	1.329042	1.360578	1.099499
3.69	1.294093	1.312124	1.054814
3.7	1.258033	1.262669	1.009803
3.71	1.220933	1.21239	0.964581
3.72	1.182938	1.161466	0.919283
3.73	1.144217	1.110074	0.874046
3.74	1.104938	1.058389	0.829005
3.75	1.065269	1.00659	0.784298
3.76	1.025376	0.954852	0.740061
3.77	0.985427	0.903354	0.696432
3.78	0.945591	0.852271	0.653547
3.79	0.906034	0.801781	0.611544
3.8	0.866925	0.752061	0.570558
3.81	0.82843	0.703287	0.530721
3.82	0.790711	0.655634	0.492134
3.83	0.753929	0.609278	0.454892
3.84	0.718247	0.564394	0.419091
3.85	0.683825	0.521157	0.384826
3.86	0.650824	0.479741	0.352192
3.87	0.619406	0.440323	0.321284
3.88	0.589732	0.403077	0.292199
3.89	0.561964	0.368179	0.26503
3.9	0.536262	0.335803	0.239874

3.91	0.512765	0.306101	0.216817
3.92	0.491519	0.279131	0.19591
3.93	0.472548	0.254929	0.177196
3.94	0.455875	0.23353	0.160719
3.95	0.441524	0.214968	0.146521
3.96	0.429518	0.199278	0.134644
3.97	0.419881	0.186496	0.125132
3.98	0.412636	0.176656	0.118028
3.99	0.407806	0.169794	0.113374
4	0.405415	0.165944	0.111213
4.01	0.405462	0.165119	0.111572
4.02	0.407845	0.167248	0.114417
4.03	0.41244	0.172235	0.119697
4.04	0.419119	0.179987	0.127362
4.05	0.427759	0.190408	0.13736
4.06	0.438232	0.203405	0.149642
4.07	0.450413	0.218882	0.164156
4.08	0.464177	0.236746	0.180853
4.09	0.479398	0.256903	0.199681
4.1	0.49595	0.279257	0.220591
4.11	0.51371	0.303699	0.243509
4.12	0.53256	0.330051	0.268274
4.13	0.552386	0.358121	0.294699
4.14	0.573073	0.387716	0.322602
4.15	0.594508	0.418644	0.351798
4.16	0.616574	0.450712	0.382101
4.17	0.639157	0.483726	0.413328
4.18	0.662143	0.517496	0.445294
4.19	0.685417	0.551826	0.477814
4.2	0.708864	0.586526	0.510704
4.21	0.732379	0.621411	0.543782
4.22	0.755894	0.656334	0.576879
4.23	0.779351	0.691156	0.609828
4.24	0.802692	0.725738	0.642463
4.25	0.82586	0.759943	0.674618
4.26	0.848795	0.793632	0.706126
4.27	0.87144	0.826665	0.73682
4.28	0.893736	0.858906	0.766535
4.29	0.915626	0.890214	0.795104
4.3	0.937051	0.920452	0.82236
4.31	0.95796	0.949495	0.848165
4.32	0.978319	0.977274	0.872494
4.33	0.998104	1.003732	0.89535
4.34	1.01729	1.028813	0.916735
4.35	1.03585	1.052462	0.936652
4.36	1.053759	1.074623	0.955104
4.37	1.07099	1.095239	0.972093
4.38	1.087519	1.114256	0.987623

4.39	1.10332	1.131617	1.001696
4.4	1.118366	1.147266	1.014315
4.41	1.132612	1.161172	1.025483
4.42	1.145924	1.173399	1.0352
4.43	1.158151	1.184037	1.043467
4.44	1.169138	1.193175	1.050285
4.45	1.178733	1.200902	1.055654
4.46	1.186782	1.207307	1.059575
4.47	1.193132	1.212479	1.06205
4.48	1.197631	1.216508	1.063078
4.49	1.200125	1.219483	1.06266
4.5	1.200461	1.221493	1.060797
4.51	1.198454	1.222569	1.057467
4.52	1.193792	1.222512	1.052563
4.53	1.186131	1.221063	1.045954
4.54	1.175128	1.217967	1.037509
4.55	1.160438	1.212965	1.027099
4.56	1.141718	1.205801	1.014593
4.57	1.118625	1.196215	0.99986
4.58	1.090813	1.183952	0.98277
4.59	1.05794	1.168754	0.963194
4.6	1.019662	1.150363	0.941
4.61	0.97574	1.128562	0.916067
4.62	0.926353	1.10329	0.888305
4.63	0.871785	1.074526	0.857635
4.64	0.81232	1.042251	0.823977
4.65	0.748244	1.006442	0.787249
4.66	0.679839	0.967081	0.747371
4.67	0.60739	0.924146	0.704264
4.68	0.531182	0.877616	0.657846
4.69	0.451499	0.827471	0.608038
4.7	0.368623	0.77369	0.554758
4.71	0.282856	0.716255	0.497958
4.72	0.194555	0.655163	0.437715
4.73	0.104093	0.590411	0.374137
4.74	0.011843	0.521998	0.307333
4.75	-0.08182	0.449921	0.237411
4.76	-0.17653	0.374181	0.164479
4.77	-0.2719	0.294775	0.088645
4.78	-0.36757	0.211701	0.010018
4.79	-0.46316	0.124958	-0.07129
4.8	-0.5583	0.034544	-0.15518
4.81	-0.65266	-0.05948	-0.2415
4.82	-0.7461	-0.15678	-0.32995
4.83	-0.83854	-0.25699	-0.4202
4.84	-0.92987	-0.35971	-0.51192
4.85	-1.02001	-0.46456	-0.60477
4.86	-1.10885	-0.57115	-0.69841

4.87	-1.19632	-0.6791	-0.79252
4.88	-1.28231	-0.78801	-0.88676
4.89	-1.36673	-0.89751	-0.98079
4.9	-1.4495	-1.00721	-1.07429
4.91	-1.53054	-1.11675	-1.16694
4.92	-1.60987	-1.22591	-1.25857
4.93	-1.68755	-1.33449	-1.34903
4.94	-1.76362	-1.44232	-1.43817
4.95	-1.83814	-1.54919	-1.52585
4.96	-1.91114	-1.65491	-1.61192
4.97	-1.9827	-1.75929	-1.69622
4.98	-2.05284	-1.86214	-1.77862
4.99	-2.12162	-1.96328	-1.85897
5	-2.18909	-2.0625	-1.93711
5.01	-2.25529	-2.15963	-2.01293
5.02	-2.32025	-2.25453	-2.08643
5.03	-2.38397	-2.34709	-2.15763
5.04	-2.44647	-2.43719	-2.22657
5.05	-2.50776	-2.52471	-2.29327
5.06	-2.56786	-2.60952	-2.35777
5.07	-2.62678	-2.69152	-2.42009
5.08	-2.68453	-2.77057	-2.48026
5.09	-2.74113	-2.84656	-2.53831
5.1	-2.79659	-2.91936	-2.59427
5.11	-2.85092	-2.98892	-2.6482
5.12	-2.90411	-3.05534	-2.70027
5.13	-2.95614	-3.11878	-2.75071
5.14	-3.00701	-3.17942	-2.79973
5.15	-3.05669	-3.23741	-2.84753
5.16	-3.10518	-3.29292	-2.89433
5.17	-3.15245	-3.34612	-2.94035
5.18	-3.1985	-3.39715	-2.98579
5.19	-3.24331	-3.4462	-3.03087
5.2	-3.28686	-3.49342	-3.0758
5.21	-3.32915	-3.53896	-3.12075
5.22	-3.37018	-3.58287	-3.16568
5.23	-3.40998	-3.62517	-3.21054
5.24	-3.44854	-3.66591	-3.25524
5.25	-3.48589	-3.70511	-3.29971
5.26	-3.52204	-3.74281	-3.34387
5.27	-3.55701	-3.77903	-3.38766
5.28	-3.5908	-3.8138	-3.43101
5.29	-3.62343	-3.84717	-3.47382
5.3	-3.65492	-3.87916	-3.51605
5.31	-3.68527	-3.90978	-3.55759
5.32	-3.7145	-3.93902	-3.59832
5.33	-3.74262	-3.96683	-3.6381
5.34	-3.76961	-3.99318	-3.67678

5.35	-3.79551	-4.01803	-3.71422
5.36	-3.8203	-4.04134	-3.75028
5.37	-3.844	-4.06307	-3.78482
5.38	-3.86661	-4.08319	-3.8177
5.39	-3.88814	-4.10164	-3.84876
5.4	-3.9086	-4.11841	-3.87788
5.41	-3.92799	-4.13344	-3.90493
5.42	-3.94631	-4.14668	-3.92993
5.43	-3.96357	-4.15806	-3.95294
5.44	-3.97975	-4.16751	-3.97398
5.45	-3.99488	-4.17499	-3.99311
5.46	-4.00893	-4.18042	-4.01037
5.47	-4.02192	-4.18374	-4.02581
5.48	-4.03385	-4.18489	-4.03947
5.49	-4.04472	-4.1838	-4.05139
5.5	-4.05452	-4.18042	-4.06162
5.51	-4.06325	-4.1747	-4.0702
5.52	-4.07094	-4.16672	-4.07717
5.53	-4.07758	-4.15659	-4.08253
5.54	-4.08318	-4.14441	-4.08632
5.55	-4.08775	-4.13027	-4.08856
5.56	-4.09131	-4.11428	-4.08928
5.57	-4.09385	-4.09655	-4.08849
5.58	-4.09539	-4.07717	-4.08622
5.59	-4.09594	-4.05625	-4.0825
5.6	-4.0955	-4.03389	-4.07735
5.61	-4.09408	-4.01019	-4.07082
5.62	-4.09167	-3.98524	-4.06308
5.63	-4.08826	-3.95913	-4.05434
5.64	-4.08382	-3.93195	-4.0448
5.65	-4.07836	-3.90377	-4.03466
5.66	-4.07184	-3.8747	-4.02411
5.67	-4.06427	-3.84481	-4.01336
5.68	-4.05562	-3.8142	-4.00261
5.69	-4.04589	-3.78295	-3.99207
5.7	-4.03505	-3.75115	-3.98193
5.71	-4.0231	-3.71888	-3.97233
5.72	-4.01003	-3.68617	-3.96313
5.73	-3.99584	-3.65304	-3.95415
5.74	-3.98054	-3.61952	-3.94519
5.75	-3.96411	-3.58563	-3.93604
5.76	-3.94655	-3.55139	-3.92652
5.77	-3.92787	-3.51682	-3.91643
5.78	-3.90807	-3.48195	-3.90558
5.79	-3.88713	-3.44681	-3.89376
5.8	-3.86507	-3.4114	-3.88078
5.81	-3.84188	-3.37577	-3.86649
5.82	-3.81755	-3.33994	-3.8509

5.83	-3.7921	-3.30397	-3.83407
5.84	-3.76551	-3.26787	-3.81606
5.85	-3.7378	-3.23171	-3.79691
5.86	-3.70896	-3.19552	-3.77669
5.87	-3.67899	-3.15934	-3.75545
5.88	-3.64789	-3.12321	-3.73324
5.89	-3.61567	-3.08717	-3.71013
5.9	-3.58232	-3.05126	-3.68617
5.91	-3.54784	-3.01552	-3.6614
5.92	-3.51221	-2.97999	-3.6358
5.93	-3.47543	-2.9447	-3.60935
5.94	-3.43745	-2.90967	-3.58201
5.95	-3.39828	-2.87496	-3.55376
5.96	-3.35788	-2.84057	-3.52456
5.97	-3.31624	-2.80656	-3.49439
5.98	-3.27334	-2.77296	-3.46321
5.99	-3.22916	-2.73979	-3.43101
6	-3.18368	-2.70708	-3.39773
6.01	-3.13689	-2.67487	-3.36338
6.02	-3.08878	-2.64308	-3.328
6.03	-3.03935	-2.61166	-3.29166
6.04	-2.9886	-2.58053	-3.25443
6.05	-2.93654	-2.54963	-3.21638
6.06	-2.88316	-2.51888	-3.17756
6.07	-2.82846	-2.48823	-3.13805
6.08	-2.77244	-2.45758	-3.09792
6.09	-2.71511	-2.42689	-3.05723
6.1	-2.65647	-2.39607	-3.01605
6.11	-2.5965	-2.36505	-2.97442
6.12	-2.53521	-2.33368	-2.93231
6.13	-2.47259	-2.3018	-2.88966
6.14	-2.40863	-2.26925	-2.8464
6.15	-2.34332	-2.23588	-2.80247
6.16	-2.27666	-2.20152	-2.75783
6.17	-2.20864	-2.16602	-2.71239
6.18	-2.13924	-2.12921	-2.66612
6.19	-2.06846	-2.09095	-2.61894
6.2	-1.9963	-2.05106	-2.57079
6.21	-1.92278	-2.00941	-2.52164
6.22	-1.84806	-1.96601	-2.47148
6.23	-1.77234	-1.92086	-2.42034
6.24	-1.69582	-1.87398	-2.36824
6.25	-1.61868	-1.82539	-2.3152
6.26	-1.54114	-1.7751	-2.26124
6.27	-1.46338	-1.72313	-2.2064
6.28	-1.38561	-1.66951	-2.15067
6.29	-1.30801	-1.61424	-2.0941
6.3	-1.23079	-1.55734	-2.03669

6.31	-1.15416	-1.49885	-1.97849
6.32	-1.07837	-1.43885	-1.91955
6.33	-1.0037	-1.37745	-1.85994
6.34	-0.93041	-1.31474	-1.79974
6.35	-0.85879	-1.25084	-1.73901
6.36	-0.78911	-1.18584	-1.67784
6.37	-0.72162	-1.11986	-1.61628
6.38	-0.65662	-1.05298	-1.55442
6.39	-0.59437	-0.98531	-1.49232
6.4	-0.53513	-0.91696	-1.43005
6.41	-0.47914	-0.84807	-1.36771
6.42	-0.42641	-0.77893	-1.30545
6.43	-0.37687	-0.70988	-1.24344
6.44	-0.33049	-0.64124	-1.18184
6.45	-0.28723	-0.57336	-1.12084
6.46	-0.24704	-0.50656	-1.0606
6.47	-0.20987	-0.44117	-1.00129
6.48	-0.17567	-0.37754	-0.94309
6.49	-0.14442	-0.31598	-0.88616
6.5	-0.11604	-0.25684	-0.83068
6.51	-0.09051	-0.20042	-0.77681
6.52	-0.06773	-0.14687	-0.72465
6.53	-0.04763	-0.09632	-0.67431
6.54	-0.03012	-0.04891	-0.62589
6.55	-0.01512	-0.00477	-0.57948
6.56	-0.00254	0.035988	-0.53518
6.57	0.007706	0.073222	-0.4931
6.58	0.015692	0.106806	-0.45333
6.59	0.021507	0.136612	-0.41598
6.6	0.025234	0.162511	-0.38114
6.61	0.026968	0.184412	-0.34888
6.62	0.026847	0.202381	-0.3192
6.63	0.025019	0.21652	-0.29206
6.64	0.021633	0.226935	-0.26742
6.65	0.016837	0.233728	-0.24524
6.66	0.010779	0.237003	-0.22549
6.67	0.003608	0.236864	-0.20813
6.68	-0.00453	0.233415	-0.19312
6.69	-0.01348	0.226759	-0.18042
6.7	-0.0231	0.217	-0.17
6.71	-0.03324	0.204287	-0.16181
6.72	-0.04378	0.188953	-0.15574
6.73	-0.05457	0.171373	-0.15168
6.74	-0.06549	0.151924	-0.1495
6.75	-0.07641	0.130985	-0.1491
6.76	-0.0872	0.108933	-0.15035
6.77	-0.09773	0.086143	-0.15313
6.78	-0.10787	0.062993	-0.15733

6.79	-0.11748	0.039861	-0.16283
6.8	-0.12645	0.017123	-0.16952
6.81	-0.13466	-0.00489	-0.17726
6.82	-0.14213	-0.02605	-0.1859
6.83	-0.14892	-0.04626	-0.19524
6.84	-0.15507	-0.06545	-0.20513
6.85	-0.16063	-0.08352	-0.21539
6.86	-0.16564	-0.1004	-0.22584
6.87	-0.17015	-0.11599	-0.2363
6.88	-0.17422	-0.13021	-0.2466
6.89	-0.17788	-0.14297	-0.25657
6.9	-0.18118	-0.15419	-0.26603
6.91	-0.18417	-0.1638	-0.27482
6.92	-0.18685	-0.17175	-0.28283
6.93	-0.18925	-0.17801	-0.28997
6.94	-0.19137	-0.18257	-0.29614
6.95	-0.19321	-0.18539	-0.30124
6.96	-0.19479	-0.18644	-0.30518
6.97	-0.19612	-0.1857	-0.30786
6.98	-0.1972	-0.18314	-0.30919
6.99	-0.19805	-0.17873	-0.30907
7	-0.19867	-0.17244	-0.3074
7.01	-0.19908	-0.1643	-0.30412
7.02	-0.19929	-0.15456	-0.29926
7.03	-0.19932	-0.14352	-0.29287
7.04	-0.19919	-0.1315	-0.28501
7.05	-0.19893	-0.11879	-0.27573
7.06	-0.19855	-0.1057	-0.26509
7.07	-0.19806	-0.09255	-0.25314
7.08	-0.1975	-0.07962	-0.23994
7.09	-0.19688	-0.06723	-0.22555
7.1	-0.19621	-0.05568	-0.21001
7.11	-0.19552	-0.04522	-0.19341
7.12	-0.19481	-0.0358	-0.17591
7.13	-0.1941	-0.02733	-0.15769
7.14	-0.19337	-0.01969	-0.13893
7.15	-0.19265	-0.0128	-0.11981
7.16	-0.19192	-0.00655	-0.10051
7.17	-0.19121	-0.00083	-0.08122
7.18	-0.19051	0.004459	-0.06211
7.19	-0.18983	0.009414	-0.04337
7.2	-0.18918	0.01414	-0.02518
7.21	-0.18855	0.018702	-0.00769
7.22	-0.18795	0.023018	0.009031
7.23	-0.18738	0.026968	0.024948
7.24	-0.18683	0.030434	0.040025
7.25	-0.18631	0.033296	0.054225
7.26	-0.1858	0.035435	0.067513

7.27	-0.18531	0.036732	0.079851
7.28	-0.18484	0.037067	0.091203
7.29	-0.18438	0.036321	0.101533
7.3	-0.18394	0.034376	0.110803
7.31	-0.18351	0.03116	0.118986
7.32	-0.18309	0.026803	0.126086
7.33	-0.18269	0.021483	0.132116
7.34	-0.18232	0.015376	0.137089
7.35	-0.18198	0.008661	0.141019
7.36	-0.18167	0.001515	0.143918
7.37	-0.1814	-0.00588	0.145799
7.38	-0.18118	-0.01336	0.146676
7.39	-0.181	-0.02073	0.146562
7.4	-0.18088	-0.02782	0.145469
7.41	-0.18081	-0.0345	0.14343
7.42	-0.18081	-0.04078	0.140556
7.43	-0.18089	-0.04673	0.136979
7.44	-0.18104	-0.05243	0.13283
7.45	-0.18129	-0.05792	0.128239
7.46	-0.18163	-0.06329	0.123337
7.47	-0.18207	-0.06859	0.118256
7.48	-0.18263	-0.07389	0.113126
7.49	-0.18331	-0.07925	0.108079
7.5	-0.18412	-0.08474	0.103245
7.51	-0.18506	-0.09041	0.098727
7.52	-0.18611	-0.09622	0.09452
7.53	-0.18726	-0.10212	0.090588
7.54	-0.18848	-0.10805	0.086897
7.55	-0.18975	-0.11395	0.083413
7.56	-0.19106	-0.11978	0.080101
7.57	-0.19237	-0.12547	0.076928
7.58	-0.19368	-0.13098	0.073858
7.59	-0.19496	-0.13624	0.070857
7.6	-0.19619	-0.14121	0.067892
7.61	-0.19735	-0.14584	0.064936
7.62	-0.19844	-0.15015	0.061997
7.63	-0.19945	-0.15415	0.059093
7.64	-0.20037	-0.15788	0.056241
7.65	-0.20121	-0.16137	0.053457
7.66	-0.20196	-0.16463	0.050759
7.67	-0.20261	-0.16769	0.048164
7.68	-0.20317	-0.17058	0.045689
7.69	-0.20363	-0.17333	0.04335
7.7	-0.20397	-0.17595	0.041166
7.71	-0.20422	-0.17847	0.03915
7.72	-0.20435	-0.18088	0.037308
7.73	-0.20438	-0.18316	0.035645
7.74	-0.2043	-0.18528	0.034163

7.75	-0.20413	-0.18725	0.032867
7.76	-0.20386	-0.18903	0.03176
7.77	-0.20349	-0.19061	0.030845
7.78	-0.20303	-0.19197	0.030127
7.79	-0.20247	-0.1931	0.029609
7.8	-0.20183	-0.19397	0.029295
7.81	-0.20111	-0.19458	0.029185
7.82	-0.2003	-0.19494	0.029262
7.83	-0.19944	-0.19508	0.02951
7.84	-0.19851	-0.19502	0.029907
7.85	-0.19754	-0.19479	0.030436
7.86	-0.19653	-0.19441	0.031078
7.87	-0.1955	-0.1939	0.031812
7.88	-0.19445	-0.19329	0.032622
7.89	-0.19339	-0.1926	0.033487
7.9	-0.19234	-0.19186	0.034388
7.91	-0.1913	-0.19109	0.035307
7.92	-0.19029	-0.19032	0.036224
7.93	-0.18931	-0.18957	0.037121
7.94	-0.18838	-0.18886	0.037979
7.95	-0.1875	-0.18821	0.038778
7.96	-0.18668	-0.18766	0.0395
7.97	-0.18594	-0.18723	0.040125
7.98	-0.18528	-0.18694	0.040635
7.99	-0.18471	-0.18681	0.04101
8	-0.18425	-0.18687	0.041232

Table S10. Test Result Data of the Double Lane Change Test at an Adhesion Coefficient of 0.8

Time	carsim	DRL-UKF	UKF
0	#######	-0.00088	-0.0004
0.01	-0.00093	-0.00088	-0.0004
0.02	-0.00106	#######	-0.0004
0.03	-0.00044	-0.00088	-0.0004
0.04	0.000869	-0.001	#######
0.05	0.002824	-0.00042	-0.00084
0.06	0.005372	0.000956	-0.00096
0.07	0.008463	0.003106	-0.0004
0.08	0.012047	0.005909	0.001
0.09	0.016073	0.00931	0.003247
0.1	0.02049	0.013252	0.006178
0.11	0.025247	0.01768	0.009733
0.12	0.030294	0.022539	0.013854

0.13	0.035581	0.027772	0.018484
0.14	0.041057	0.033324	0.023563
0.15	0.04667	0.039139	0.029034
0.16	0.052372	0.045162	0.034839
0.17	0.05811	0.051337	0.040918
0.18	0.063834	0.057609	0.047215
0.19	0.069494	0.063921	0.053671
0.2	0.075039	0.070218	0.060227
0.21	0.080425	0.076444	0.066826
0.22	0.085634	0.082543	0.073409
0.23	0.090653	0.088468	0.079918
0.24	0.095471	0.094197	0.086295
0.25	0.100075	0.099718	0.092489
0.26	0.104454	0.105018	0.098479
0.27	0.108596	0.110083	0.104251
0.28	0.112489	0.1149	0.109791
0.29	0.116121	0.119456	0.115087
0.3	0.11948	0.123738	0.120123
0.31	0.122557	0.127733	0.124886
0.32	0.12536	0.131428	0.129363
0.33	0.127898	0.134813	0.133539
0.34	0.130181	0.137896	0.137402
0.35	0.13222	0.140687	0.140941
0.36	0.134024	0.143199	0.144164
0.37	0.135604	0.145442	0.147082
0.38	0.13697	0.147426	0.149708
0.39	0.138132	0.149165	0.152053
0.4	0.1391	0.150667	0.154128
0.41	0.139884	0.151945	0.155945
0.42	0.140488	0.15301	0.157516
0.43	0.140918	0.153872	0.158852
0.44	0.141178	0.154537	0.159965
0.45	0.141272	0.15501	0.160866
0.46	0.141207	0.155295	0.161561
0.47	0.140985	0.1554	0.162055
0.48	0.140612	0.155327	0.162354
0.49	0.140093	0.155084	0.162463
0.5	0.139431	0.154673	0.162388
0.51	0.138635	0.154102	0.162133
0.52	0.137723	0.153375	0.161704
0.53	0.136714	0.152499	0.161107
0.54	0.135629	0.151495	0.160346
0.55	0.13449	0.150385	0.159431
0.56	0.133316	0.149192	0.158381
0.57	0.132129	0.147939	0.157221
0.58	0.130948	0.146648	0.155974
0.59	0.129794	0.145341	0.154663
0.6	0.128689	0.144043	0.153314

0.61	0.127647	0.142774	0.151948
0.62	0.126668	0.141558	0.15059
0.63	0.125746	0.140412	0.149264
0.64	0.124873	0.139335	0.147992
0.65	0.124044	0.13832	0.146795
0.66	0.123253	0.13736	0.145669
0.67	0.122494	0.136449	0.144607
0.68	0.121761	0.135579	0.143604
0.69	0.121047	0.134744	0.142651
0.7	0.120346	0.133937	0.141741
0.71	0.119654	0.133151	0.140869
0.72	0.118973	0.132381	0.140025
0.73	0.118305	0.131619	0.139204
0.74	0.117655	0.13087	0.138398
0.75	0.117025	0.130136	0.137602
0.76	0.11642	0.12942	0.136818
0.77	0.115842	0.128728	0.136051
0.78	0.115295	0.128062	0.135303
0.79	0.114781	0.127426	0.134579
0.8	0.114306	0.126824	0.133883
0.81	0.11387	0.12626	0.133218
0.82	0.113471	0.125736	0.132589
0.83	0.113103	0.125257	0.131999
0.84	0.112763	0.124818	0.131452
0.85	0.112447	0.124413	0.13095
0.86	0.112149	0.12404	0.130491
0.87	0.111865	0.123691	0.130069
0.88	0.111591	0.123363	0.129678
0.89	0.111322	0.123051	0.129314
0.9	0.111054	0.12275	0.128971
0.91	0.110784	0.122454	0.128644
0.92	0.110512	0.12216	0.128329
0.93	0.110239	0.121863	0.12802
0.94	0.109968	0.121563	0.127713
0.95	0.109699	0.121263	0.127402
0.96	0.109435	0.120965	0.127089
0.97	0.109177	0.120669	0.126775
0.98	0.108927	0.120379	0.126463
0.99	0.108686	0.120095	0.126154
1	0.108455	0.11982	0.125851
1.01	0.108236	0.119554	0.125554
1.02	0.108025	0.119301	0.125266
1.03	0.107817	0.11906	0.124989
1.04	0.107607	0.118828	0.124724
1.05	0.10739	0.118598	0.124472
1.06	0.107163	0.118367	0.124229
1.07	0.106919	0.118129	0.123989
1.08	0.106656	0.117879	0.123748

1.09	0.106366	0.117611	0.123499
1.1	0.106047	0.117321	0.123237
1.11	0.105695	0.117003	0.122957
1.12	0.105311	0.116652	0.122654
1.13	0.104898	0.116264	0.122321
1.14	0.104459	0.115842	0.121954
1.15	0.103997	0.115388	0.121549
1.16	0.103516	0.114905	0.121107
1.17	0.103017	0.114397	0.120633
1.18	0.102504	0.113867	0.120128
1.19	0.10198	0.113318	0.119597
1.2	0.101447	0.112754	0.119043
1.21	0.100906	0.112178	0.118469
1.22	0.100341	0.111592	0.117879
1.23	0.099736	0.110996	0.117276
1.24	0.099072	0.110375	0.116664
1.25	0.098332	0.10971	0.116042
1.26	0.097498	0.10898	0.115393
1.27	0.096552	0.108165	0.114697
1.28	0.095477	0.107248	0.113933
1.29	0.094255	0.106208	0.113082
1.3	0.092867	0.105025	0.112123
1.31	0.091298	0.10368	0.111035
1.32	0.089534	0.102154	0.109799
1.33	0.087563	0.100428	0.108393
1.34	0.085372	0.098487	0.106797
1.35	0.082951	0.096319	0.104993
1.36	0.080285	0.09391	0.102964
1.37	0.077364	0.091246	0.100697
1.38	0.074175	0.088314	0.098178
1.39	0.070706	0.085101	0.095393
1.4	0.066944	0.081593	0.092328
1.41	0.062882	0.077777	0.088969
1.42	0.058527	0.073639	0.085301
1.43	0.053892	0.06917	0.081312
1.44	0.048987	0.06438	0.076986
1.45	0.043825	0.059281	0.072314
1.46	0.038417	0.053885	0.067306
1.47	0.032776	0.048207	0.061975
1.48	0.026913	0.042259	0.056335
1.49	0.020841	0.036054	0.050398
1.5	0.01457	0.029605	0.04418
1.51	0.008114	0.022925	0.037693
1.52	0.001493	0.016027	0.03095
1.53	-0.00527	0.008925	0.023967
1.54	-0.01216	0.001642	0.016755
1.55	-0.01916	-0.00501	0.009331
1.56	-0.02623	-0.01156	0.001717

1.57	-0.03337	-0.0182	-0.0048
1.58	-0.04055	-0.02492	-0.01107
1.59	-0.04775	-0.0317	-0.01743
1.6	-0.05495	-0.03852	-0.02387
1.61	-0.06214	-0.04536	-0.03037
1.62	-0.06935	-0.0522	-0.0369
1.63	-0.07663	-0.05903	-0.04345
1.64	-0.08402	-0.06589	-0.05001
1.65	-0.09157	-0.0728	-0.05655
1.66	-0.09931	-0.07982	-0.06311
1.67	-0.10729	-0.08699	-0.06974
1.68	-0.11556	-0.09434	-0.07646
1.69	-0.12416	-0.10193	-0.08333
1.7	-0.13313	-0.10978	-0.09037
1.71	-0.14251	-0.11795	-0.09764
1.72	-0.15236	-0.12647	-0.10516
1.73	-0.1627	-0.13539	-0.11298
1.74	-0.17359	-0.14474	-0.12115
1.75	-0.18507	-0.15457	-0.12969
1.76	-0.19717	-0.16491	-0.13865
1.77	-0.20994	-0.17582	-0.14806
1.78	-0.22343	-0.18731	-0.15797
1.79	-0.23767	-0.19945	-0.16841
1.8	-0.25271	-0.21226	-0.17943
1.81	-0.26857	-0.22579	-0.19105
1.82	-0.28519	-0.24007	-0.20332
1.83	-0.3025	-0.25514	-0.21628
1.84	-0.32043	-0.27093	-0.22996
1.85	-0.33891	-0.28738	-0.24439
1.86	-0.35785	-0.30441	-0.25952
1.87	-0.37719	-0.32196	-0.27528
1.88	-0.39685	-0.33996	-0.2916
1.89	-0.41676	-0.35833	-0.30841
1.9	-0.43684	-0.37701	-0.32565
1.91	-0.45704	-0.39592	-0.34325
1.92	-0.47734	-0.415	-0.36114
1.93	-0.49776	-0.43419	-0.37925
1.94	-0.51831	-0.45347	-0.39753
1.95	-0.539	-0.47287	-0.4159
1.96	-0.55983	-0.4924	-0.43438
1.97	-0.58083	-0.51205	-0.45296
1.98	-0.60198	-0.53184	-0.47166
1.99	-0.62332	-0.55178	-0.49049
2	-0.64484	-0.57189	-0.50945
2.01	-0.66655	-0.59215	-0.52855
2.02	-0.6884	-0.6126	-0.54781
2.03	-0.71035	-0.63322	-0.56722
2.04	-0.73234	-0.65398	-0.58681

2.05	-0.75432	-0.67483	-0.60656
2.06	-0.77625	-0.69572	-0.62644
2.07	-0.79807	-0.71661	-0.64642
2.08	-0.81973	-0.73744	-0.66643
2.09	-0.84118	-0.75816	-0.68643
2.1	-0.86237	-0.77874	-0.70639
2.11	-0.88325	-0.79912	-0.72624
2.12	-0.90378	-0.81925	-0.74595
2.13	-0.9239	-0.83909	-0.76547
2.14	-0.94357	-0.85859	-0.78476
2.15	-0.96274	-0.8777	-0.80376
2.16	-0.98137	-0.89639	-0.82244
2.17	-0.99941	-0.9146	-0.84075
2.18	-1.01681	-0.9323	-0.85865
2.19	-1.03353	-0.94944	-0.87609
2.2	-1.04951	-0.96597	-0.89305
2.21	-1.06474	-0.98185	-0.90946
2.22	-1.07921	-0.99704	-0.9253
2.23	-1.09295	-1.0115	-0.94051
2.24	-1.106	-1.02525	-0.95506
2.25	-1.11838	-1.0383	-0.96891
2.26	-1.13011	-1.0507	-0.98208
2.27	-1.14122	-1.06246	-0.99459
2.28	-1.15174	-1.0736	-1.00646
2.29	-1.1617	-1.08416	-1.01772
2.3	-1.17112	-1.09416	-1.0284
2.31	-1.18002	-1.10362	-1.03851
2.32	-1.18838	-1.11256	-1.04809
2.33	-1.19621	-1.12101	-1.05715
2.34	-1.20347	-1.12896	-1.06572
2.35	-1.21015	-1.1364	-1.07381
2.36	-1.21623	-1.14329	-1.08143
2.37	-1.2217	-1.14964	-1.08855
2.38	-1.22654	-1.15542	-1.09515
2.39	-1.23074	-1.16062	-1.10123
2.4	-1.23427	-1.16521	-1.10677
2.41	-1.2371	-1.1692	-1.11175
2.42	-1.23917	-1.17255	-1.11615
2.43	-1.24039	-1.17525	-1.11997
2.44	-1.24066	-1.17722	-1.12318
2.45	-1.2399	-1.17837	-1.12576
2.46	-1.23803	-1.17863	-1.12765
2.47	-1.23495	-1.17791	-1.12875
2.48	-1.23057	-1.17613	-1.129
2.49	-1.22482	-1.1732	-1.12831
2.5	-1.21759	-1.16904	-1.1266
2.51	-1.20883	-1.16357	-1.1238
2.52	-1.1985	-1.15671	-1.11982

2.53	-1.18659	-1.14839	-1.11458
2.54	-1.1731	-1.13857	-1.10801
2.55	-1.15802	-1.12726	-1.10003
2.56	-1.14133	-1.11445	-1.09063
2.57	-1.12302	-1.10011	-1.0798
2.58	-1.10309	-1.08426	-1.06752
2.59	-1.08153	-1.06687	-1.05379
2.6	-1.05832	-1.04794	-1.03861
2.61	-1.03346	-1.02745	-1.02195
2.62	-1.00693	-1.00541	-1.00381
2.63	-0.97871	-0.98179	-0.98419
2.64	-0.94878	-0.95658	-0.96307
2.65	-0.91712	-0.92977	-0.94045
2.66	-0.88373	-0.90134	-0.91631
2.67	-0.84857	-0.87127	-0.89062
2.68	-0.81163	-0.83954	-0.86339
2.69	-0.7729	-0.80614	-0.83458
2.7	-0.73235	-0.77105	-0.80419
2.71	-0.69001	-0.73425	-0.7722
2.72	-0.64603	-0.69574	-0.73858
2.73	-0.60062	-0.65551	-0.70334
2.74	-0.55396	-0.61373	-0.66644
2.75	-0.50626	-0.57059	-0.62791
2.76	-0.45772	-0.52627	-0.58789
2.77	-0.40852	-0.48095	-0.54656
2.78	-0.35887	-0.43483	-0.50411
2.79	-0.30897	-0.3881	-0.4607
2.8	-0.25901	-0.34093	-0.41652
2.81	-0.20917	-0.29352	-0.37176
2.82	-0.1596	-0.24606	-0.32658
2.83	-0.11044	-0.19871	-0.28116
2.84	-0.0618	-0.15162	-0.2357
2.85	-0.01382	-0.10491	-0.19035
2.86	0.033362	-0.05871	-0.14524
2.87	0.079624	-0.01313	-0.1005
2.88	0.124834	0.036698	-0.05624
2.89	0.168859	0.087587	-0.01258
2.9	0.211571	0.137317	0.038366
2.91	0.252874	0.185745	0.091568
2.92	0.292829	0.232728	0.143559
2.93	0.331531	0.278162	0.194188
2.94	0.369075	0.322112	0.243306
2.95	0.405558	0.364684	0.290806
2.96	0.441075	0.405982	0.336754
2.97	0.475722	0.446113	0.38126
2.98	0.509596	0.485182	0.424436
2.99	0.542793	0.523295	0.466391
3	0.575407	0.560556	0.507236

3.01	0.607519	0.597072	0.547081
3.02	0.639137	0.632948	0.586036
3.03	0.670255	0.668271	0.624212
3.04	0.700866	0.703051	0.661718
3.05	0.730962	0.737281	0.698646
3.06	0.760536	0.770952	0.735008
3.07	0.789581	0.804058	0.770793
3.08	0.81809	0.83659	0.805996
3.09	0.846056	0.86854	0.840606
3.1	0.873471	0.899899	0.874617
3.11	0.900324	0.930662	0.908019
3.12	0.926583	0.960818	0.940804
3.13	0.952212	0.990356	0.972964
3.14	0.977176	1.019241	1.004492
3.15	1.001438	1.047433	1.035372
3.16	1.024963	1.074893	1.06557
3.17	1.047715	1.101582	1.095044
3.18	1.069657	1.127459	1.123752
3.19	1.090755	1.152486	1.151654
3.2	1.110971	1.176623	1.178707
3.21	1.130264	1.19983	1.204872
3.22	1.148562	1.222068	1.230106
3.23	1.165789	1.24329	1.254368
3.24	1.181868	1.263418	1.277617
3.25	1.196721	1.282368	1.299803
3.26	1.210271	1.300055	1.320847
3.27	1.222441	1.316393	1.340658
3.28	1.233153	1.331298	1.359148
3.29	1.242331	1.344685	1.376229
3.3	1.249897	1.356469	1.391812
3.31	1.255806	1.366564	1.405807
3.32	1.260138	1.374887	1.418126
3.33	1.263006	1.381387	1.428681
3.34	1.264521	1.386152	1.437382
3.35	1.264796	1.389306	1.444177
3.36	1.263943	1.390973	1.449159
3.37	1.262074	1.391276	1.452457
3.38	1.259302	1.390337	1.454199
3.39	1.255739	1.388282	1.454515
3.4	1.251496	1.385232	1.453535
3.41	1.246701	1.381312	1.451386
3.42	1.241538	1.376645	1.448198
3.43	1.236209	1.371371	1.444099
3.44	1.230911	1.365692	1.43922
3.45	1.225846	1.359829	1.433706
3.46	1.221214	1.354003	1.427769
3.47	1.217213	1.348431	1.42164
3.48	1.214043	1.343335	1.415548

3.49	1.211906	1.338934	1.409723
3.5	1.210999	1.335448	1.404396
3.51	1.211482	1.333096	1.399794
3.52	1.213348	1.332099	1.39615
3.53	1.216547	1.332631	1.393691
3.54	1.221031	1.334683	1.392649
3.55	1.22675	1.338202	1.393205
3.56	1.233657	1.343134	1.39535
3.57	1.241702	1.349425	1.399029
3.58	1.250837	1.357023	1.404185
3.59	1.261012	1.365872	1.410763
3.6	1.272179	1.37592	1.418706
3.61	1.284288	1.387113	1.427957
3.62	1.297289	1.399397	1.438462
3.63	1.311129	1.412717	1.450163
3.64	1.325757	1.427018	1.463006
3.65	1.34112	1.442242	1.476931
3.66	1.357167	1.458332	1.491882
3.67	1.373845	1.475232	1.507798
3.68	1.391104	1.492883	1.52462
3.69	1.408891	1.51123	1.542288
3.7	1.427155	1.530215	1.560742
3.71	1.445854	1.549781	1.579922
3.72	1.464993	1.56987	1.59977
3.73	1.484587	1.59044	1.620225
3.74	1.504652	1.611492	1.641228
3.75	1.525202	1.633046	1.662732
3.76	1.546253	1.655117	1.684742
3.77	1.567821	1.677722	1.707275
3.78	1.58992	1.700878	1.73035
3.79	1.612565	1.724603	1.753982
3.8	1.635774	1.748912	1.778191
3.81	1.659551	1.773822	1.802994
3.82	1.683872	1.799351	1.828408
3.83	1.708702	1.825506	1.85445
3.84	1.734009	1.852259	1.88114
3.85	1.759757	1.879573	1.908483
3.86	1.785914	1.90741	1.936452
3.87	1.812444	1.935733	1.965008
3.88	1.839315	1.964505	1.99411
3.89	1.866493	1.993689	2.023721
3.9	1.893942	2.023247	2.053801
3.91	1.921626	2.053142	2.084311
3.92	1.949487	2.083337	2.115212
3.93	1.977464	2.113789	2.146466
3.94	2.005494	2.144436	2.178034
3.95	2.033516	2.17521	2.20987
3.96	2.061468	2.206043	2.241911

3.97	2.089289	2.236867	2.274083
3.98	2.116917	2.267615	2.306318
3.99	2.14429	2.298218	2.338543
4	2.171347	2.328609	2.370688
4.01	2.198032	2.358719	2.402682
4.02	2.224318	2.388482	2.434454
4.03	2.250181	2.417836	2.465934
4.04	2.275599	2.446749	2.497049
4.05	2.300551	2.475199	2.527737
4.06	2.325015	2.503159	2.557965
4.07	2.348968	2.530607	2.587708
4.08	2.372389	2.557517	2.616939
4.09	2.395254	2.583865	2.645634
4.1	2.417543	2.609628	2.673767
4.11	2.439209	2.63478	2.701314
4.12	2.460108	2.659297	2.728247
4.13	2.480075	2.683129	2.754542
4.14	2.498943	2.706119	2.780174
4.15	2.516544	2.728083	2.80509
4.16	2.532712	2.748837	2.829125
4.17	2.54728	2.768198	2.852087
4.18	2.56008	2.785983	2.873784
4.19	2.570947	2.802007	2.894026
4.2	2.579713	2.816088	2.912619
4.21	2.586229	2.828042	2.929371
4.22	2.590411	2.837685	2.944092
4.23	2.592195	2.844852	2.956589
4.24	2.591514	2.849453	2.96667
4.25	2.588302	2.851414	2.974163
4.26	2.582494	2.850665	2.978973
4.27	2.574024	2.847132	2.981024
4.28	2.562827	2.840743	2.980241
4.29	2.548837	2.831427	2.976547
4.3	2.531988	2.81911	2.969868
4.31	2.512228	2.803721	2.960128
4.32	2.489568	2.785186	2.947251
4.33	2.464031	2.763451	2.931162
4.34	2.43564	2.738525	2.911786
4.35	2.404418	2.710434	2.889063
4.36	2.370391	2.679203	2.863003
4.37	2.333581	2.64486	2.833635
4.38	2.294012	2.60743	2.800985
4.39	2.251708	2.566939	2.765081
4.4	2.206692	2.523413	2.72595
4.41	2.15898	2.476878	2.683618
4.42	2.108559	2.427361	2.638114
4.43	2.055406	2.374879	2.589464
4.44	1.999499	2.319415	2.537696

4.45	1.940816	2.260947	2.482828
4.46	1.879335	2.199449	2.424843
4.47	1.815034	2.134898	2.363717
4.48	1.747892	2.067269	2.299424
4.49	1.677885	1.996538	2.231938
4.5	1.604993	1.922681	2.161235
4.51	1.529236	1.845674	2.08729
4.52	1.450812	1.765492	2.010076
4.53	1.36996	1.68216	1.929568
4.54	1.28692	1.595893	1.845742
4.55	1.201934	1.506956	1.758622
4.56	1.115239	1.415612	1.668434
4.57	1.027078	1.322127	1.575454
4.58	0.93769	1.226763	1.479958
4.59	0.847315	1.129786	1.382224
4.6	0.756193	1.031459	1.282525
4.61	0.664568	0.932046	1.18114
4.62	0.572703	0.831812	1.078343
4.63	0.480863	0.731025	0.974412
4.64	0.389313	0.629974	0.869622
4.65	0.29832	0.528949	0.764254
4.66	0.20815	0.428245	0.658609
4.67	0.119067	0.328152	0.552992
4.68	0.031338	0.228965	0.44771
4.69	-0.05477	0.130974	0.343069
4.7	-0.13899	0.034472	0.239372
4.71	-0.22112	-0.05203	0.136927
4.72	-0.30113	-0.13204	0.036039
4.73	-0.37906	-0.21006	-0.04984
4.74	-0.45494	-0.28607	-0.12649
4.75	-0.52883	-0.3601	-0.20122
4.76	-0.60074	-0.4322	-0.27402
4.77	-0.67072	-0.50238	-0.34494
4.78	-0.7388	-0.5707	-0.414
4.79	-0.80503	-0.63718	-0.48123
4.8	-0.86943	-0.70186	-0.54667
4.81	-0.93203	-0.76478	-0.61035
4.82	-0.9928	-0.82596	-0.67231
4.83	-1.05171	-0.88543	-0.73257
4.84	-1.1087	-0.94316	-0.79118
4.85	-1.16375	-0.99912	-0.84814
4.86	-1.21681	-1.05327	-0.90345
4.87	-1.26784	-1.10556	-0.95705
4.88	-1.3168	-1.15597	-1.00892
4.89	-1.36366	-1.20445	-1.05901
4.9	-1.40836	-1.25096	-1.1073
4.91	-1.4509	-1.29547	-1.15374
4.92	-1.49142	-1.33794	-1.19829

4.93	-1.53007	-1.37836	-1.24093
4.94	-1.56702	-1.41685	-1.28161
4.95	-1.60244	-1.45357	-1.32032
4.96	-1.63647	-1.48867	-1.35719
4.97	-1.66929	-1.52231	-1.39237
4.98	-1.70106	-1.55465	-1.42599
4.99	-1.73194	-1.58583	-1.45822
5	-1.7621	-1.61601	-1.48919
5.01	-1.79166	-1.64534	-1.51905
5.02	-1.82061	-1.67399	-1.54797
5.03	-1.84892	-1.70207	-1.57607
5.04	-1.87654	-1.72958	-1.60351
5.05	-1.90344	-1.75647	-1.63041
5.06	-1.92957	-1.78272	-1.65676
5.07	-1.95488	-1.80827	-1.68252
5.08	-1.97935	-1.83309	-1.70765
5.09	-2.00292	-1.85714	-1.73213
5.1	-2.02556	-1.88038	-1.75591
5.11	-2.0472	-1.90278	-1.77894
5.12	-2.06767	-1.92428	-1.80121
5.13	-2.08676	-1.94484	-1.82266
5.14	-2.10428	-1.96428	-1.84326
5.15	-2.12004	-1.98242	-1.86295
5.16	-2.13382	-1.99907	-1.88158
5.17	-2.14544	-2.01403	-1.89895
5.18	-2.1547	-2.02713	-1.9149
5.19	-2.16139	-2.03817	-1.92923
5.2	-2.16532	-2.04696	-1.94178
5.21	-2.16634	-2.05332	-1.95235
5.22	-2.16449	-2.05705	-1.96077
5.23	-2.15987	-2.05802	-1.96686
5.24	-2.15259	-2.05626	-1.97044
5.25	-2.14272	-2.05188	-1.97137
5.26	-2.13038	-2.04496	-1.96969
5.27	-2.11566	-2.03559	-1.96549
5.28	-2.09864	-2.02386	-1.95885
5.29	-2.07944	-2.00987	-1.94988
5.3	-2.05814	-1.99371	-1.93865
5.31	-2.03482	-1.97547	-1.92525
5.32	-2.00951	-1.95523	-1.90976
5.33	-1.9822	-1.93308	-1.89229
5.34	-1.95289	-1.90903	-1.87291
5.35	-1.92158	-1.88309	-1.85169
5.36	-1.88827	-1.85524	-1.82865
5.37	-1.85296	-1.8255	-1.8038
5.38	-1.81565	-1.79385	-1.77713
5.39	-1.77635	-1.76031	-1.74863
5.4	-1.73504	-1.72487	-1.71832

5.41	-1.69174	-1.68753	-1.68619
5.42	-1.6465	-1.64829	-1.65224
5.43	-1.59934	-1.60716	-1.61647
5.44	-1.55034	-1.56417	-1.57889
5.45	-1.49952	-1.51938	-1.53949
5.46	-1.44695	-1.47282	-1.49831
5.47	-1.39266	-1.42455	-1.4554
5.48	-1.33671	-1.3746	-1.41081
5.49	-1.27914	-1.32303	-1.36457
5.5	-1.22	-1.26987	-1.31672
5.51	-1.15938	-1.21518	-1.26732
5.52	-1.0975	-1.159	-1.2164
5.53	-1.03463	-1.10141	-1.16402
5.54	-0.97104	-1.04262	-1.1102
5.55	-0.907	-0.9829	-1.05503
5.56	-0.84276	-0.92249	-0.99872
5.57	-0.7786	-0.86165	-0.94151
5.58	-0.71479	-0.80062	-0.88365
5.59	-0.65159	-0.73967	-0.82537
5.6	-0.58926	-0.67905	-0.76691
5.61	-0.52806	-0.61901	-0.70853
5.62	-0.46821	-0.5598	-0.65046
5.63	-0.40991	-0.50166	-0.59294
5.64	-0.35336	-0.4448	-0.53623
5.65	-0.29875	-0.38941	-0.48054
5.66	-0.24631	-0.33569	-0.42607
5.67	-0.19622	-0.28382	-0.37302
5.68	-0.14869	-0.23399	-0.32155
5.69	-0.10391	-0.18641	-0.27187
5.7	-0.06211	-0.14125	-0.22414
5.71	-0.02343	-0.09872	-0.17856
5.72	0.012098	-0.059	-0.1353
5.73	0.044493	-0.02226	-0.09456
5.74	0.073773	0.013308	-0.05652
5.75	0.099952	0.048943	-0.02132
5.76	0.123049	0.08115	0.013913
5.77	0.143079	0.109947	0.051167
5.78	0.160059	0.135354	0.084838
5.79	0.174005	0.157387	0.114945
5.8	0.184935	0.176065	0.141506
5.81	0.192897	0.191406	0.16454
5.82	0.198072	0.203429	0.184067
5.83	0.200674	0.212187	0.200106
5.84	0.200915	0.21788	0.212675
5.85	0.199009	0.220741	0.221832
5.86	0.195168	0.221006	0.227783
5.87	0.189607	0.218909	0.230775
5.88	0.182539	0.214685	0.231052

5.89	0.174176	0.208568	0.22886
5.9	0.164733	0.200793	0.224443
5.91	0.154409	0.191594	0.218048
5.92	0.143354	0.181206	0.20992
5.93	0.131706	0.16985	0.200303
5.94	0.119602	0.15769	0.189443
5.95	0.107178	0.144877	0.17757
5.96	0.094571	0.131562	0.164857
5.97	0.081919	0.117895	0.151462
5.98	0.069359	0.104028	0.137542
5.99	0.057028	0.090111	0.123254
6	0.045062	0.076295	0.108757
6.01	0.033572	0.06273	0.094207
6.02	0.02256	0.049568	0.079763
6.03	0.012002	0.036929	0.065582
6.04	0.001873	0.024816	0.051821
6.05	-0.00785	0.013202	0.038607
6.06	-0.0172	0.00206	0.025944
6.07	-0.02618	-0.00746	0.013802
6.08	-0.03484	-0.01634	0.002154
6.09	-0.04319	-0.02487	-0.00714
6.1	-0.05126	-0.0331	-0.01565
6.11	-0.05907	-0.04103	-0.02383
6.12	-0.06661	-0.0487	-0.03171
6.13	-0.0739	-0.05611	-0.0393
6.14	-0.08092	-0.06328	-0.04665
6.15	-0.08767	-0.0702	-0.05375
6.16	-0.09415	-0.07687	-0.06062
6.17	-0.10036	-0.08329	-0.06725
6.18	-0.1063	-0.08945	-0.07364
6.19	-0.11196	-0.09534	-0.07978
6.2	-0.11733	-0.10098	-0.08568
6.21	-0.12243	-0.10636	-0.09133
6.22	-0.12724	-0.11147	-0.09673
6.23	-0.13175	-0.11631	-0.10188
6.24	-0.13597	-0.12088	-0.10677
6.25	-0.13988	-0.12516	-0.11141
6.26	-0.14348	-0.12917	-0.11579
6.27	-0.14677	-0.13289	-0.11989
6.28	-0.14973	-0.13631	-0.12373
6.29	-0.15237	-0.13943	-0.12729
6.3	-0.15468	-0.14225	-0.13057
6.31	-0.15666	-0.14476	-0.13356
6.32	-0.1583	-0.14695	-0.13626
6.33	-0.15964	-0.14882	-0.13866
6.34	-0.16067	-0.15039	-0.14076
6.35	-0.16141	-0.15166	-0.14256
6.36	-0.16186	-0.15263	-0.14406

6.37	-0.16205	-0.15334	-0.14527
6.38	-0.16199	-0.15377	-0.14621
6.39	-0.16167	-0.15395	-0.14688
6.4	-0.16112	-0.15389	-0.1473
6.41	-0.16035	-0.15359	-0.14747
6.42	-0.15939	-0.15307	-0.14741
6.43	-0.15826	-0.15233	-0.14712
6.44	-0.15699	-0.15142	-0.14662
6.45	-0.15562	-0.15034	-0.14592
6.46	-0.15416	-0.14914	-0.14504
6.47	-0.15266	-0.14783	-0.14401
6.48	-0.15113	-0.14645	-0.14286
6.49	-0.14962	-0.14503	-0.14161
6.5	-0.14814	-0.14358	-0.14029
6.51	-0.14672	-0.14214	-0.13892
6.52	-0.14536	-0.14073	-0.13753
6.53	-0.14406	-0.13938	-0.13615
6.54	-0.14282	-0.13809	-0.1348
6.55	-0.14163	-0.13686	-0.13351
6.56	-0.14049	-0.13568	-0.13228
6.57	-0.13939	-0.13455	-0.1311
6.58	-0.13834	-0.13346	-0.12996
6.59	-0.13733	-0.13242	-0.12888
6.6	-0.13636	-0.13143	-0.12784
6.61	-0.13542	-0.13047	-0.12685
6.62	-0.13451	-0.12954	-0.12589
6.63	-0.13364	-0.12865	-0.12497
6.64	-0.1328	-0.12779	-0.12409
6.65	-0.13201	-0.12696	-0.12323
6.66	-0.13124	-0.12616	-0.12241
6.67	-0.13052	-0.12541	-0.12161
6.68	-0.12984	-0.12468	-0.12085
6.69	-0.12919	-0.12399	-0.12013
6.7	-0.12859	-0.12334	-0.11943
6.71	-0.12802	-0.12273	-0.11877
6.72	-0.12749	-0.12216	-0.11815
6.73	-0.127	-0.12162	-0.11756
6.74	-0.12653	-0.12112	-0.11701
6.75	-0.12609	-0.12065	-0.1165
6.76	-0.12568	-0.12021	-0.11602
6.77	-0.12528	-0.11979	-0.11557
6.78	-0.12489	-0.11939	-0.11515
6.79	-0.12451	-0.11901	-0.11475
6.8	-0.12414	-0.11865	-0.11437
6.81	-0.12377	-0.11829	-0.114
6.82	-0.12341	-0.11794	-0.11365
6.83	-0.12305	-0.11759	-0.11331
6.84	-0.12269	-0.11724	-0.11297

6.85	-0.12234	-0.1169	-0.11264
6.86	-0.12199	-0.11656	-0.1123
6.87	-0.12165	-0.11622	-0.11197
6.88	-0.12132	-0.11589	-0.11165
6.89	-0.121	-0.11557	-0.11133
6.9	-0.12068	-0.11526	-0.11101
6.91	-0.12038	-0.11495	-0.11071
6.92	-0.12008	-0.11465	-0.1104
6.93	-0.1198	-0.11436	-0.11011
6.94	-0.11953	-0.11408	-0.10982
6.95	-0.11927	-0.11381	-0.10955
6.96	-0.11903	-0.11355	-0.10928
6.97	-0.11879	-0.11331	-0.10902
6.98	-0.11858	-0.11307	-0.10877
6.99	-0.11837	-0.11285	-0.10854
7	-0.11819	-0.11265	-0.10831
7.01	-0.11802	-0.11246	-0.1081
7.02	-0.11787	-0.11228	-0.1079
7.03	-0.11773	-0.11212	-0.10772
7.04	-0.11762	-0.11197	-0.10755
7.05	-0.11752	-0.11185	-0.1074
7.06	-0.11744	-0.11174	-0.10726
7.07	-0.11739	-0.11165	-0.10714
7.08	-0.11735	-0.11157	-0.10703
7.09	-0.11734	-0.11152	-0.10694
7.1	-0.11734	-0.11148	-0.10687
7.11	-0.11737	-0.11147	-0.10682
7.12	-0.11742	-0.11148	-0.10679
7.13	-0.11749	-0.1115	-0.10678
7.14	-0.11757	-0.11155	-0.10678
7.15	-0.11766	-0.11161	-0.10681
7.16	-0.11776	-0.11169	-0.10685
7.17	-0.11787	-0.11178	-0.10691
7.18	-0.11799	-0.11187	-0.10699
7.19	-0.11811	-0.11198	-0.10707
7.2	-0.11823	-0.11209	-0.10716
7.21	-0.11835	-0.1122	-0.10727
7.22	-0.11846	-0.11232	-0.10737
7.23	-0.11858	-0.11243	-0.10748
7.24	-0.11868	-0.11254	-0.10759
7.25	-0.11878	-0.11265	-0.1077
7.26	-0.11888	-0.11275	-0.1078
7.27	-0.11897	-0.11284	-0.1079
7.28	-0.11905	-0.11294	-0.108
7.29	-0.11912	-0.11302	-0.10809
7.3	-0.11918	-0.1131	-0.10818
7.31	-0.11923	-0.11316	-0.10826
7.32	-0.11928	-0.11322	-0.10833

7.33	-0.11932	-0.11327	-0.1084
7.34	-0.11936	-0.11331	-0.10846
7.35	-0.11941	-0.11335	-0.1085
7.36	-0.11946	-0.11339	-0.10854
7.37	-0.11952	-0.11344	-0.10858
7.38	-0.1196	-0.11349	-0.10862
7.39	-0.1197	-0.11355	-0.10866
7.4	-0.11981	-0.11362	-0.10871
7.41	-0.11995	-0.11371	-0.10877
7.42	-0.12013	-0.11382	-0.10884
7.43	-0.12034	-0.11396	-0.10892
7.44	-0.12059	-0.11412	-0.10903
7.45	-0.1209	-0.11432	-0.10916
7.46	-0.12127	-0.11456	-0.10931
7.47	-0.1217	-0.11486	-0.10951
7.48	-0.12221	-0.11521	-0.10974
7.49	-0.12279	-0.11562	-0.11002
7.5	-0.12346	-0.1161	-0.11036
7.51	-0.12422	-0.11665	-0.11075
7.52	-0.12506	-0.11729	-0.11121
7.53	-0.12597	-0.11801	-0.11174
7.54	-0.12694	-0.11881	-0.11235
7.55	-0.12795	-0.11967	-0.11304
7.56	-0.12899	-0.12059	-0.11381
7.57	-0.13006	-0.12155	-0.11463
7.58	-0.13113	-0.12254	-0.11551
7.59	-0.13221	-0.12356	-0.11643
7.6	-0.13327	-0.12458	-0.11738
7.61	-0.1343	-0.1256	-0.11835
7.62	-0.1353	-0.1266	-0.11933
7.63	-0.13625	-0.12759	-0.12031
7.64	-0.13714	-0.12853	-0.12127
7.65	-0.13797	-0.12944	-0.12221
7.66	-0.13872	-0.13029	-0.12312
7.67	-0.13938	-0.13107	-0.12399
7.68	-0.13995	-0.13178	-0.1248
7.69	-0.1404	-0.13241	-0.12555
7.7	-0.14074	-0.13295	-0.12623
7.71	-0.14095	-0.13338	-0.12684
7.72	-0.14104	-0.1337	-0.12735
7.73	-0.14102	-0.1339	-0.12777
7.74	-0.14088	-0.13399	-0.12807
7.75	-0.14065	-0.13397	-0.12827
7.76	-0.14031	-0.13384	-0.12835
7.77	-0.13989	-0.13361	-0.12833
7.78	-0.13939	-0.1333	-0.1282
7.79	-0.13881	-0.1329	-0.12799
7.8	-0.13816	-0.13242	-0.12769

7.81	-0.13744	-0.13187	-0.1273
7.82	-0.13668	-0.13125	-0.12684
7.83	-0.13586	-0.13057	-0.12631
7.84	-0.13499	-0.12984	-0.12572
7.85	-0.13409	-0.12906	-0.12507
7.86	-0.13316	-0.12824	-0.12437
7.87	-0.1322	-0.12739	-0.12363
7.88	-0.13123	-0.1265	-0.12284
7.89	-0.13024	-0.12559	-0.12202
7.9	-0.12924	-0.12466	-0.12117
7.91	-0.12825	-0.12373	-0.1203
7.92	-0.12726	-0.12278	-0.11942
7.93	-0.12629	-0.12184	-0.11852
7.94	-0.12533	-0.1209	-0.11761
7.95	-0.1244	-0.11997	-0.11671
7.96	-0.1235	-0.11906	-0.11581
7.97	-0.12264	-0.11818	-0.11492
7.98	-0.12183	-0.11733	-0.11405
7.99	-0.12106	-0.11651	-0.1132
8	-0.12035	-0.11573	-0.11239

Table S11. Test Result Data of the Sinusoidal Steering Test at an Adhesion Coefficient of 0.2

Time	carsim	DRL-UKF	UKF
0	#######	-0.00593	0.00079
0.01	-0.00021	-0.0075	-0.0008
0.02	-0.00075	-0.00895	-0.00227
0.03	-0.00159	-0.01028	-0.00362
0.04	-0.00272	-0.01153	-0.00488
0.05	-0.0041	-0.01269	-0.00606
0.06	-0.00573	-0.01379	-0.00717
0.07	-0.00758	-0.01483	-0.00823
0.08	-0.00963	-0.01584	-0.00925
0.09	-0.01185	-0.01682	-0.01024
0.1	-0.01424	-0.01778	-0.01122
0.11	-0.01676	-0.01875	-0.0122
0.12	-0.0194	-0.01974	-0.0132
0.13	-0.02213	-0.02075	-0.01422
0.14	-0.02495	-0.0218	-0.01529
0.15	-0.02781	-0.02292	-0.01642
0.16	-0.03071	-0.0241	-0.01762
0.17	-0.03363	-0.02536	-0.0189
0.18	-0.03653	-0.02673	-0.02028

0.19	-0.03941	-0.0282	-0.02177
0.2	-0.04224	-0.0298	-0.02339
0.21	-0.04501	-0.03153	-0.02515
0.22	-0.0477	-0.03338	-0.02702
0.23	-0.05032	-0.03533	-0.029
0.24	-0.05286	-0.03736	-0.03105
0.25	-0.05531	-0.03944	-0.03316
0.26	-0.05769	-0.04156	-0.03531
0.27	-0.05998	-0.0437	-0.03748
0.28	-0.06218	-0.04584	-0.03965
0.29	-0.06428	-0.04795	-0.04179
0.3	-0.06629	-0.05002	-0.04388
0.31	-0.06821	-0.05203	-0.04592
0.32	-0.07001	-0.05398	-0.04788
0.33	-0.07171	-0.05585	-0.04978
0.34	-0.07328	-0.05765	-0.0516
0.35	-0.07474	-0.05938	-0.05335
0.36	-0.07606	-0.06103	-0.05502
0.37	-0.07724	-0.0626	-0.05661
0.38	-0.07828	-0.06408	-0.05812
0.39	-0.07917	-0.06548	-0.05954
0.4	-0.0799	-0.06678	-0.06086
0.41	-0.08046	-0.068	-0.0621
0.42	-0.08088	-0.06912	-0.06325
0.43	-0.08116	-0.07016	-0.06431
0.44	-0.08132	-0.07112	-0.06529
0.45	-0.08137	-0.072	-0.06619
0.46	-0.08132	-0.07281	-0.06701
0.47	-0.08119	-0.07354	-0.06775
0.48	-0.08099	-0.07421	-0.06842
0.49	-0.08074	-0.07481	-0.06902
0.5	-0.08046	-0.07536	-0.06955
0.51	-0.08014	-0.07584	-0.07002
0.52	-0.07979	-0.07627	-0.07042
0.53	-0.07943	-0.07662	-0.07074
0.54	-0.07903	-0.07688	-0.07098
0.55	-0.07861	-0.07706	-0.07114
0.56	-0.07816	-0.07714	-0.07121
0.57	-0.07769	-0.07711	-0.07119
0.58	-0.07719	-0.07697	-0.07107
0.59	-0.07667	-0.0767	-0.07084
0.6	-0.07612	-0.0763	-0.0705
0.61	-0.07555	-0.07576	-0.07006
0.62	-0.07495	-0.07514	-0.06954
0.63	-0.07434	-0.07446	-0.06897
0.64	-0.07372	-0.07379	-0.0684
0.65	-0.07309	-0.07317	-0.06785
0.66	-0.07246	-0.07266	-0.06736

0.67	-0.07183	-0.07229	-0.06697
0.68	-0.07121	-0.07211	-0.0667
0.69	-0.0706	-0.07219	-0.06661
0.7	-0.07001	-0.07255	-0.06671
0.71	-0.06944	-0.07324	-0.06704
0.72	-0.06889	-0.07423	-0.06757
0.73	-0.06836	-0.07547	-0.06827
0.74	-0.06785	-0.07692	-0.06912
0.75	-0.06735	-0.07852	-0.07008
0.76	-0.06688	-0.08025	-0.07112
0.77	-0.06641	-0.08204	-0.07221
0.78	-0.06596	-0.08386	-0.07332
0.79	-0.06552	-0.08565	-0.07442
0.8	-0.06509	-0.08739	-0.07548
0.81	-0.06467	-0.08902	-0.07647
0.82	-0.06426	-0.09055	-0.07739
0.83	-0.06387	-0.09198	-0.07824
0.84	-0.06348	-0.09334	-0.07903
0.85	-0.06311	-0.09461	-0.07977
0.86	-0.06275	-0.09582	-0.08045
0.87	-0.06241	-0.09697	-0.08108
0.88	-0.06208	-0.09807	-0.08166
0.89	-0.06177	-0.09913	-0.0822
0.9	-0.06147	-0.10016	-0.0827
0.91	-0.0612	-0.10116	-0.08317
0.92	-0.06094	-0.10214	-0.08361
0.93	-0.06069	-0.10312	-0.08402
0.94	-0.06047	-0.1041	-0.08441
0.95	-0.06025	-0.10509	-0.0848
0.96	-0.06004	-0.10609	-0.08517
0.97	-0.05985	-0.10711	-0.08555
0.98	-0.05966	-0.10817	-0.08594
0.99	-0.05947	-0.10926	-0.08634
1	-0.0593	-0.1104	-0.08676
1.01	-0.05912	-0.11159	-0.0872
1.02	-0.05895	-0.11279	-0.08764
1.03	-0.05878	-0.11396	-0.08804
1.04	-0.05862	-0.11506	-0.08839
1.05	-0.05845	-0.11605	-0.08865
1.06	-0.05828	-0.11688	-0.0888
1.07	-0.0581	-0.11752	-0.0888
1.08	-0.05793	-0.11792	-0.08863
1.09	-0.05775	-0.11803	-0.08826
1.1	-0.05756	-0.11782	-0.08767
1.11	-0.05737	-0.11727	-0.08683
1.12	-0.05717	-0.11639	-0.08576
1.13	-0.05697	-0.11525	-0.08451
1.14	-0.05677	-0.11389	-0.0831

1.15	-0.05656	-0.11235	-0.08157
1.16	-0.05634	-0.1107	-0.07996
1.17	-0.05613	-0.10896	-0.07828
1.18	-0.05591	-0.1072	-0.07658
1.19	-0.05569	-0.10546	-0.07489
1.2	-0.05547	-0.10379	-0.07325
1.21	-0.05526	-0.10223	-0.07168
1.22	-0.05504	-0.10078	-0.07018
1.23	-0.05483	-0.09942	-0.06875
1.24	-0.05462	-0.09816	-0.06738
1.25	-0.05442	-0.09697	-0.06606
1.26	-0.05423	-0.09584	-0.06481
1.27	-0.05405	-0.09478	-0.0636
1.28	-0.05388	-0.09376	-0.06243
1.29	-0.05373	-0.09279	-0.0613
1.3	-0.05359	-0.09184	-0.06021
1.31	-0.05348	-0.09091	-0.05915
1.32	-0.05337	-0.09	-0.05812
1.33	-0.05326	-0.08911	-0.05712
1.34	-0.05316	-0.08826	-0.05615
1.35	-0.05304	-0.08743	-0.05522
1.36	-0.0529	-0.08664	-0.05432
1.37	-0.05273	-0.08588	-0.05346
1.38	-0.05253	-0.08516	-0.05264
1.39	-0.05229	-0.08449	-0.05186
1.4	-0.052	-0.08386	-0.05112
1.41	-0.05166	-0.08328	-0.05042
1.42	-0.05129	-0.08274	-0.04976
1.43	-0.05092	-0.08226	-0.04914
1.44	-0.05057	-0.08182	-0.04856
1.45	-0.05026	-0.08144	-0.04802
1.46	-0.05003	-0.08109	-0.04752
1.47	-0.04989	-0.0808	-0.04705
1.48	-0.04988	-0.08055	-0.04661
1.49	-0.05002	-0.08036	-0.04621
1.5	-0.05033	-0.0802	-0.04585
1.51	-0.05084	-0.0801	-0.04551
1.52	-0.05156	-0.08004	-0.04522
1.53	-0.0525	-0.08004	-0.04497
1.54	-0.05367	-0.08011	-0.04478
1.55	-0.05508	-0.08023	-0.04465
1.56	-0.05674	-0.08043	-0.04459
1.57	-0.05866	-0.0807	-0.04461
1.58	-0.06084	-0.08104	-0.04472
1.59	-0.0633	-0.08148	-0.04493
1.6	-0.06604	-0.08199	-0.04523
1.61	-0.06908	-0.08261	-0.04565
1.62	-0.0724	-0.08331	-0.04618

1.63	-0.076	-0.08412	-0.04683
1.64	-0.07987	-0.08502	-0.04759
1.65	-0.08401	-0.08604	-0.04847
1.66	-0.0884	-0.08716	-0.04948
1.67	-0.09303	-0.08839	-0.0506
1.68	-0.0979	-0.08974	-0.05185
1.69	-0.103	-0.0912	-0.05322
1.7	-0.10831	-0.09279	-0.05472
1.71	-0.11384	-0.0945	-0.05635
1.72	-0.11957	-0.09634	-0.0581
1.73	-0.12549	-0.0983	-0.05999
1.74	-0.13158	-0.1004	-0.06199
1.75	-0.13784	-0.10262	-0.06413
1.76	-0.14425	-0.10498	-0.06638
1.77	-0.15079	-0.10747	-0.06876
1.78	-0.15747	-0.11009	-0.07126
1.79	-0.16425	-0.11285	-0.07389
1.8	-0.17114	-0.11575	-0.07663
1.81	-0.17812	-0.11879	-0.07949
1.82	-0.18518	-0.12197	-0.08247
1.83	-0.1923	-0.12529	-0.08557
1.84	-0.19946	-0.12875	-0.08879
1.85	-0.20667	-0.13236	-0.09213
1.86	-0.21389	-0.1361	-0.0956
1.87	-0.22113	-0.13999	-0.09919
1.88	-0.22836	-0.14403	-0.10291
1.89	-0.23557	-0.14821	-0.10676
1.9	-0.24275	-0.15253	-0.11073
1.91	-0.24989	-0.157	-0.11484
1.92	-0.25697	-0.16161	-0.11906
1.93	-0.26401	-0.16636	-0.1234
1.94	-0.27099	-0.17124	-0.12785
1.95	-0.27791	-0.17625	-0.1324
1.96	-0.28478	-0.18138	-0.13704
1.97	-0.29158	-0.18663	-0.14177
1.98	-0.29831	-0.19199	-0.14658
1.99	-0.30498	-0.19746	-0.15146
2	-0.31157	-0.20304	-0.15641
2.01	-0.31809	-0.20872	-0.16141
2.02	-0.32453	-0.21449	-0.16647
2.03	-0.3309	-0.22035	-0.17159
2.04	-0.33719	-0.2263	-0.17675
2.05	-0.34341	-0.23233	-0.18197
2.06	-0.34956	-0.23844	-0.18723
2.07	-0.35563	-0.24461	-0.19254
2.08	-0.36163	-0.25085	-0.1979
2.09	-0.36756	-0.25716	-0.20329
2.1	-0.37341	-0.26352	-0.20873

2.11	-0.37919	-0.26993	-0.2142
2.12	-0.3849	-0.27639	-0.21972
2.13	-0.39053	-0.2829	-0.22527
2.14	-0.3961	-0.28945	-0.23086
2.15	-0.4016	-0.29604	-0.23649
2.16	-0.40702	-0.30266	-0.24216
2.17	-0.41238	-0.30932	-0.24787
2.18	-0.41768	-0.31602	-0.25362
2.19	-0.4229	-0.32274	-0.25941
2.2	-0.42806	-0.32948	-0.26524
2.21	-0.43316	-0.33625	-0.2711
2.22	-0.43819	-0.34303	-0.27701
2.23	-0.44315	-0.34982	-0.28294
2.24	-0.44805	-0.3566	-0.2889
2.25	-0.45288	-0.36338	-0.29488
2.26	-0.45765	-0.37014	-0.30087
2.27	-0.46234	-0.37688	-0.30687
2.28	-0.46698	-0.38359	-0.31288
2.29	-0.47154	-0.39026	-0.31889
2.3	-0.47604	-0.39688	-0.32489
2.31	-0.48047	-0.40345	-0.33088
2.32	-0.48483	-0.40996	-0.33686
2.33	-0.48912	-0.41642	-0.34281
2.34	-0.49334	-0.42281	-0.34875
2.35	-0.49749	-0.42914	-0.35466
2.36	-0.50156	-0.43541	-0.36055
2.37	-0.50556	-0.44162	-0.3664
2.38	-0.50949	-0.44776	-0.37221
2.39	-0.51334	-0.45383	-0.37799
2.4	-0.5171	-0.45983	-0.38372
2.41	-0.52079	-0.46576	-0.3894
2.42	-0.5244	-0.47161	-0.39504
2.43	-0.52794	-0.47739	-0.40063
2.44	-0.53138	-0.48308	-0.40617
2.45	-0.53475	-0.4887	-0.41166
2.46	-0.53804	-0.49423	-0.41709
2.47	-0.54125	-0.49966	-0.42247
2.48	-0.54437	-0.50501	-0.4278
2.49	-0.54741	-0.51027	-0.43307
2.5	-0.55037	-0.51542	-0.43829
2.51	-0.55325	-0.52048	-0.44344
2.52	-0.55604	-0.52543	-0.44854
2.53	-0.55875	-0.53028	-0.45358
2.54	-0.56137	-0.535	-0.45856
2.55	-0.56391	-0.53961	-0.46347
2.56	-0.56636	-0.54409	-0.46832
2.57	-0.56873	-0.54844	-0.47311
2.58	-0.57101	-0.55266	-0.47782

2.59	-0.57321	-0.55673	-0.48247
2.6	-0.57532	-0.56065	-0.48705
2.61	-0.57734	-0.56443	-0.49157
2.62	-0.57928	-0.56806	-0.49601
2.63	-0.58112	-0.57153	-0.50037
2.64	-0.58288	-0.57487	-0.50467
2.65	-0.58455	-0.57807	-0.50889
2.66	-0.58613	-0.58113	-0.51304
2.67	-0.58761	-0.58405	-0.51712
2.68	-0.58901	-0.58685	-0.52112
2.69	-0.59031	-0.58952	-0.52505
2.7	-0.59152	-0.59206	-0.5289
2.71	-0.59264	-0.59448	-0.53268
2.72	-0.59367	-0.59677	-0.53638
2.73	-0.59461	-0.59894	-0.54
2.74	-0.59546	-0.60099	-0.54355
2.75	-0.59622	-0.60291	-0.54702
2.76	-0.59691	-0.60471	-0.55042
2.77	-0.59751	-0.60637	-0.55374
2.78	-0.59804	-0.60791	-0.55699
2.79	-0.59849	-0.60931	-0.56016
2.8	-0.59887	-0.61058	-0.56326
2.81	-0.59918	-0.61173	-0.56628
2.82	-0.59941	-0.61273	-0.56923
2.83	-0.59956	-0.61361	-0.5721
2.84	-0.59963	-0.61435	-0.57489
2.85	-0.59962	-0.61496	-0.5776
2.86	-0.59952	-0.61544	-0.58023
2.87	-0.59933	-0.61578	-0.58277
2.88	-0.59904	-0.61599	-0.58523
2.89	-0.59866	-0.61607	-0.5876
2.9	-0.59817	-0.61601	-0.58988
2.91	-0.59758	-0.61582	-0.59207
2.92	-0.59688	-0.61551	-0.59417
2.93	-0.59607	-0.61506	-0.59618
2.94	-0.59515	-0.61449	-0.5981
2.95	-0.5941	-0.61381	-0.59993
2.96	-0.59294	-0.61301	-0.60167
2.97	-0.59165	-0.61211	-0.60332
2.98	-0.59023	-0.61109	-0.60487
2.99	-0.58868	-0.60998	-0.60634
3	-0.58699	-0.60877	-0.60772
3.01	-0.58517	-0.60746	-0.609
3.02	-0.58322	-0.60606	-0.61019
3.03	-0.58113	-0.60456	-0.61129
3.04	-0.57892	-0.60296	-0.61229
3.05	-0.57659	-0.60126	-0.6132
3.06	-0.57415	-0.59945	-0.61402

3.07	-0.57159	-0.59754	-0.61473
3.08	-0.56893	-0.59552	-0.61535
3.09	-0.56616	-0.59338	-0.61586
3.1	-0.56329	-0.59113	-0.61628
3.11	-0.56033	-0.58877	-0.61659
3.12	-0.55727	-0.58629	-0.6168
3.13	-0.55411	-0.58368	-0.61691
3.14	-0.55084	-0.58094	-0.6169
3.15	-0.54746	-0.57806	-0.61679
3.16	-0.54396	-0.57505	-0.61656
3.17	-0.54034	-0.57189	-0.61621
3.18	-0.5366	-0.56858	-0.61575
3.19	-0.53273	-0.56511	-0.61517
3.2	-0.52873	-0.56149	-0.61446
3.21	-0.52459	-0.55771	-0.61364
3.22	-0.52032	-0.55376	-0.61267
3.23	-0.51592	-0.54965	-0.61157
3.24	-0.5114	-0.54538	-0.61032
3.25	-0.50676	-0.54095	-0.6089
3.26	-0.50201	-0.53636	-0.60732
3.27	-0.49715	-0.53162	-0.60557
3.28	-0.49219	-0.52673	-0.60363
3.29	-0.48712	-0.52167	-0.60149
3.3	-0.48197	-0.51647	-0.59916
3.31	-0.47672	-0.51112	-0.59661
3.32	-0.47139	-0.50561	-0.59386
3.33	-0.46598	-0.49996	-0.5909
3.34	-0.46049	-0.49416	-0.58773
3.35	-0.45492	-0.48821	-0.58436
3.36	-0.44927	-0.48211	-0.58078
3.37	-0.44356	-0.47587	-0.577
3.38	-0.43777	-0.46948	-0.57303
3.39	-0.43193	-0.46295	-0.56885
3.4	-0.42602	-0.45628	-0.56448
3.41	-0.42005	-0.44947	-0.55992
3.42	-0.41403	-0.44251	-0.55516
3.43	-0.40795	-0.43543	-0.55021
3.44	-0.40183	-0.42822	-0.54508
3.45	-0.39565	-0.42089	-0.53976
3.46	-0.38943	-0.41345	-0.53427
3.47	-0.38316	-0.40589	-0.5286
3.48	-0.37686	-0.39823	-0.52275
3.49	-0.37052	-0.39047	-0.51674
3.5	-0.36414	-0.38262	-0.51056
3.51	-0.35772	-0.37468	-0.50422
3.52	-0.35128	-0.36665	-0.49772
3.53	-0.3448	-0.35856	-0.49106
3.54	-0.3383	-0.3504	-0.48426

3.55	-0.33176	-0.34218	-0.47732
3.56	-0.3252	-0.33391	-0.47025
3.57	-0.31862	-0.32561	-0.46305
3.58	-0.31201	-0.31727	-0.45572
3.59	-0.30537	-0.3089	-0.44828
3.6	-0.29872	-0.30052	-0.44073
3.61	-0.29204	-0.29213	-0.43307
3.62	-0.28535	-0.28374	-0.42532
3.63	-0.27864	-0.27535	-0.41748
3.64	-0.27192	-0.26696	-0.40959
3.65	-0.26519	-0.25858	-0.40163
3.66	-0.25845	-0.25021	-0.39364
3.67	-0.25171	-0.24185	-0.38562
3.68	-0.24497	-0.23352	-0.37758
3.69	-0.23822	-0.22521	-0.36955
3.7	-0.23148	-0.21693	-0.36152
3.71	-0.22475	-0.20869	-0.35351
3.72	-0.21803	-0.20048	-0.34553
3.73	-0.21133	-0.19233	-0.33756
3.74	-0.20466	-0.18424	-0.32961
3.75	-0.19802	-0.17622	-0.32168
3.76	-0.19141	-0.16827	-0.31376
3.77	-0.18485	-0.16042	-0.30585
3.78	-0.17834	-0.15266	-0.29794
3.79	-0.17188	-0.14502	-0.29004
3.8	-0.16549	-0.13748	-0.28215
3.81	-0.15916	-0.13008	-0.27426
3.82	-0.15291	-0.1228	-0.26637
3.83	-0.14673	-0.11564	-0.25851
3.84	-0.14064	-0.10862	-0.25066
3.85	-0.13462	-0.10172	-0.24285
3.86	-0.1287	-0.09496	-0.23508
3.87	-0.12286	-0.08832	-0.22735
3.88	-0.11711	-0.08182	-0.21968
3.89	-0.11147	-0.07545	-0.21207
3.9	-0.10592	-0.06921	-0.20453
3.91	-0.10048	-0.0631	-0.19706
3.92	-0.09513	-0.05712	-0.18967
3.93	-0.08988	-0.05126	-0.18236
3.94	-0.08471	-0.04552	-0.17513
3.95	-0.07963	-0.0399	-0.16798
3.96	-0.07463	-0.03439	-0.16091
3.97	-0.0697	-0.02898	-0.15393
3.98	-0.06484	-0.02367	-0.14702
3.99	-0.06004	-0.01846	-0.1402
4	-0.0553	-0.01334	-0.13347
4.01	-0.05061	-0.0083	-0.12682
4.02	-0.04597	-0.00336	-0.12026

4.03	-0.04137	0.001497	-0.1138
4.04	-0.03681	0.00626	-0.10742
4.05	-0.03228	0.010927	-0.10115
4.06	-0.02778	0.015499	-0.09498
4.07	-0.02331	0.019971	-0.08891
4.08	-0.01885	0.024344	-0.08295
4.09	-0.01441	0.028614	-0.0771
4.1	-0.00998	0.032779	-0.07136
4.11	-0.00555	0.03684	-0.06574
4.12	-0.00112	0.040801	-0.06023
4.13	0.003314	0.044671	-0.05484
4.14	0.007763	0.048457	-0.04955
4.15	0.01223	0.052166	-0.04437
4.16	0.016722	0.055805	-0.03929
4.17	0.021245	0.059383	-0.03432
4.18	0.025805	0.062906	-0.02944
4.19	0.030408	0.066382	-0.02466
4.2	0.035059	0.069818	-0.01997
4.21	0.039763	0.073221	-0.01537
4.22	0.044522	0.076596	-0.01086
4.23	0.049335	0.079947	-0.00644
4.24	0.054201	0.083278	-0.00211
4.25	0.059121	0.086592	0.002148
4.26	0.064094	0.089894	0.006319
4.27	0.06912	0.093188	0.01041
4.28	0.074198	0.096478	0.014424
4.29	0.079329	0.099767	0.018361
4.3	0.084511	0.103061	0.022223
4.31	0.089745	0.106361	0.026014
4.32	0.095026	0.109664	0.02974
4.33	0.100352	0.112968	0.033411
4.34	0.105717	0.116267	0.037035
4.35	0.111119	0.119557	0.040621
4.36	0.116554	0.122835	0.044176
4.37	0.122017	0.126095	0.04771
4.38	0.127506	0.129335	0.05123
4.39	0.133015	0.132551	0.054747
4.4	0.138542	0.135737	0.058267
4.41	0.144082	0.138891	0.061799
4.42	0.149632	0.142018	0.065348
4.43	0.155188	0.145124	0.068918
4.44	0.160747	0.148213	0.072513
4.45	0.166304	0.151293	0.076136
4.46	0.171857	0.154369	0.079792
4.47	0.177401	0.157446	0.083485
4.48	0.182933	0.160531	0.087218
4.49	0.188449	0.16363	0.090997
4.5	0.193945	0.166747	0.094824

4.51	0.199418	0.169889	0.098703
4.52	0.204867	0.173058	0.10263
4.53	0.210289	0.176256	0.1066
4.54	0.215682	0.179484	0.110609
4.55	0.221046	0.182745	0.114651
4.56	0.226379	0.18604	0.118722
4.57	0.231678	0.189372	0.122817
4.58	0.236943	0.192741	0.12693
4.59	0.242171	0.196151	0.131058
4.6	0.247361	0.199603	0.135195
4.61	0.252512	0.203099	0.139338
4.62	0.257622	0.206637	0.143492
4.63	0.262691	0.210219	0.147661
4.64	0.267719	0.213844	0.151852
4.65	0.272704	0.217511	0.15607
4.66	0.277646	0.221219	0.16032
4.67	0.282543	0.224969	0.16461
4.68	0.287397	0.22876	0.168944
4.69	0.292205	0.232592	0.173327
4.7	0.296966	0.236463	0.177767
4.71	0.301682	0.240374	0.182266
4.72	0.30635	0.244325	0.186818
4.73	0.310971	0.248316	0.191414
4.74	0.315544	0.252347	0.196048
4.75	0.32007	0.256417	0.200709
4.76	0.324548	0.260528	0.205391
4.77	0.328978	0.264678	0.210085
4.78	0.33336	0.268869	0.214782
4.79	0.337693	0.273099	0.219475
4.8	0.341977	0.27737	0.224155
4.81	0.346213	0.28168	0.228814
4.82	0.350398	0.286026	0.233453
4.83	0.354533	0.290404	0.23807
4.84	0.358616	0.294808	0.242667
4.85	0.362646	0.299236	0.247242
4.86	0.366623	0.303682	0.251797
4.87	0.370546	0.308142	0.256331
4.88	0.374414	0.312613	0.260844
4.89	0.378225	0.317088	0.265336
4.9	0.38198	0.321565	0.269808
4.91	0.385677	0.32604	0.274259
4.92	0.389315	0.330508	0.278688
4.93	0.392893	0.334967	0.283094
4.94	0.39641	0.339414	0.287477
4.95	0.399865	0.343845	0.291834
4.96	0.403257	0.348259	0.296165
4.97	0.406584	0.352652	0.300469
4.98	0.409847	0.357021	0.304745

4.99	0.413043	0.361363	0.308991
5	0.416171	0.365675	0.313206
5.01	0.419231	0.369954	0.31739
5.02	0.422221	0.374197	0.321541
5.03	0.425141	0.3784	0.325657
5.04	0.427988	0.382559	0.329738
5.05	0.430762	0.386673	0.333782
5.06	0.433461	0.390736	0.337788
5.07	0.436084	0.394745	0.341754
5.08	0.438631	0.398698	0.34568
5.09	0.441099	0.40259	0.349564
5.1	0.443488	0.406419	0.353405
5.11	0.445796	0.410181	0.357202
5.12	0.448023	0.413873	0.360957
5.13	0.450167	0.417495	0.364672
5.14	0.452227	0.421042	0.36835
5.15	0.454202	0.424514	0.371992
5.16	0.456092	0.427909	0.375602
5.17	0.457894	0.431223	0.379182
5.18	0.459609	0.434456	0.382734
5.19	0.461234	0.437604	0.38626
5.2	0.462769	0.440667	0.389763
5.21	0.464213	0.443641	0.393244
5.22	0.465565	0.446527	0.396702
5.23	0.466824	0.449323	0.400135
5.24	0.467988	0.452029	0.403541
5.25	0.469056	0.454644	0.406918
5.26	0.470027	0.457167	0.410264
5.27	0.470901	0.459599	0.413577
5.28	0.471676	0.461938	0.416854
5.29	0.47235	0.464184	0.420094
5.3	0.472924	0.466336	0.423295
5.31	0.473395	0.468393	0.426454
5.32	0.473768	0.470355	0.429569
5.33	0.474044	0.472221	0.43264
5.34	0.474226	0.473989	0.435662
5.35	0.474318	0.475658	0.438634
5.36	0.474323	0.477228	0.441555
5.37	0.474243	0.478698	0.444421
5.38	0.474081	0.480067	0.447231
5.39	0.473841	0.481333	0.449982
5.4	0.473525	0.482496	0.452673
5.41	0.473134	0.483554	0.455302
5.42	0.472665	0.484508	0.457866
5.43	0.472111	0.485355	0.460363
5.44	0.471466	0.486094	0.462793
5.45	0.470724	0.486725	0.465153
5.46	0.469878	0.487247	0.467441

5.47	0.468923	0.487657	0.469655
5.48	0.467853	0.487957	0.471795
5.49	0.46666	0.488143	0.473857
5.5	0.46534	0.488216	0.475841
5.51	0.463888	0.488174	0.477743
5.52	0.462303	0.488018	0.479562
5.53	0.460589	0.487748	0.481293
5.54	0.458745	0.487364	0.482934
5.55	0.456776	0.486868	0.48448
5.56	0.454682	0.48626	0.48593
5.57	0.452465	0.48554	0.48728
5.58	0.450127	0.48471	0.488525
5.59	0.44767	0.483768	0.489664
5.6	0.445096	0.482717	0.490693
5.61	0.442405	0.481555	0.491608
5.62	0.439597	0.480281	0.49241
5.63	0.436669	0.478892	0.493097
5.64	0.433618	0.477384	0.49367
5.65	0.430441	0.475755	0.494128
5.66	0.427137	0.474002	0.49447
5.67	0.423702	0.472121	0.494698
5.68	0.420135	0.47011	0.494809
5.69	0.416432	0.467966	0.494804
5.7	0.412592	0.465685	0.494683
5.71	0.408612	0.463265	0.494443
5.72	0.404494	0.460702	0.49408
5.73	0.400239	0.457992	0.493587
5.74	0.395849	0.455131	0.492957
5.75	0.391326	0.452116	0.492182
5.76	0.38667	0.448941	0.491258
5.77	0.381885	0.445605	0.490175
5.78	0.376971	0.442101	0.488929
5.79	0.37193	0.438428	0.487512
5.8	0.366764	0.43458	0.485918
5.81	0.361475	0.430555	0.48414
5.82	0.35607	0.426348	0.482176
5.83	0.350553	0.421958	0.480024
5.84	0.344931	0.41738	0.477683
5.85	0.33921	0.412613	0.475149
5.86	0.333397	0.407653	0.472422
5.87	0.327498	0.402497	0.469499
5.88	0.321519	0.397142	0.466379
5.89	0.315465	0.391586	0.46306
5.9	0.309344	0.385824	0.459539
5.91	0.30316	0.379857	0.455817
5.92	0.296918	0.373685	0.451895
5.93	0.29062	0.367311	0.447779
5.94	0.284269	0.360739	0.443471

5.95	0.277868	0.353972	0.438977
5.96	0.271419	0.347012	0.4343
5.97	0.264926	0.339864	0.429444
5.98	0.258391	0.332529	0.424413
5.99	0.251818	0.325011	0.419211
6	0.245208	0.317314	0.413843
6.01	0.238566	0.309441	0.408312
6.02	0.231892	0.301397	0.402618
6.03	0.22519	0.29319	0.396763
6.04	0.21846	0.284828	0.390747
6.05	0.211706	0.276315	0.38457
6.06	0.204929	0.26766	0.378233
6.07	0.198131	0.258869	0.371738
6.08	0.191314	0.249948	0.365083
6.09	0.18448	0.240906	0.35827
6.1	0.177631	0.231748	0.3513
6.11	0.17077	0.222481	0.344174
6.12	0.163897	0.213114	0.336902
6.13	0.157016	0.203654	0.329494
6.14	0.150127	0.194108	0.32196
6.15	0.143233	0.184486	0.314311
6.16	0.136335	0.174794	0.306557
6.17	0.129435	0.165041	0.29871
6.18	0.122535	0.155234	0.290778
6.19	0.115637	0.145382	0.282774
6.2	0.108742	0.135492	0.274706
6.21	0.101854	0.125572	0.266587
6.22	0.094982	0.115634	0.258425
6.23	0.088135	0.10569	0.250229
6.24	0.081325	0.095749	0.242009
6.25	0.074561	0.085825	0.233775
6.26	0.067854	0.075927	0.225536
6.27	0.061213	0.066068	0.2173
6.28	0.054649	0.05626	0.209079
6.29	0.048172	0.046513	0.20088
6.3	0.041793	0.036839	0.192713
6.31	0.03552	0.027248	0.184587
6.32	0.029355	0.017745	0.176504
6.33	0.023297	0.008336	0.168464
6.34	0.017347	-0.00098	0.160468
6.35	0.011505	-0.01019	0.152518
6.36	0.005772	-0.01929	0.144615
6.37	0.000146	-0.02829	0.136759
6.38	-0.00537	-0.03717	0.128951
6.39	-0.01078	-0.04593	0.121192
6.4	-0.01608	-0.05457	0.113484
6.41	-0.02128	-0.06309	0.105827
6.42	-0.02637	-0.07146	0.098226

6.43	-0.03137	-0.0797	0.090685
6.44	-0.03627	-0.08778	0.083209
6.45	-0.0411	-0.09571	0.075802
6.46	-0.04585	-0.10347	0.068468
6.47	-0.05053	-0.11106	0.061212
6.48	-0.05515	-0.11847	0.054039
6.49	-0.05971	-0.12569	0.046953
6.5	-0.06422	-0.13271	0.039959
6.51	-0.06869	-0.13953	0.033059
6.52	-0.07312	-0.14615	0.026253
6.53	-0.07753	-0.15256	0.019538
6.54	-0.08191	-0.15877	0.01291
6.55	-0.08628	-0.16478	0.006368
6.56	-0.09065	-0.17059	#######
6.57	-0.09501	-0.1762	-0.00647
6.58	-0.09939	-0.1816	-0.01277
6.59	-0.10379	-0.18681	-0.019
6.6	-0.1082	-0.19182	-0.02516
6.61	-0.11266	-0.19663	-0.03124
6.62	-0.11714	-0.20125	-0.03726
6.63	-0.12167	-0.20568	-0.04321
6.64	-0.12624	-0.20993	-0.04908
6.65	-0.13086	-0.21401	-0.05489
6.66	-0.13553	-0.21792	-0.06062
6.67	-0.14026	-0.22166	-0.06629
6.68	-0.14504	-0.22526	-0.07188
6.69	-0.14989	-0.2287	-0.07739
6.7	-0.15481	-0.232	-0.08284
6.71	-0.15979	-0.23516	-0.08821
6.72	-0.16485	-0.23819	-0.09351
6.73	-0.16996	-0.2411	-0.09873
6.74	-0.17514	-0.24389	-0.10389
6.75	-0.18038	-0.24659	-0.10898
6.76	-0.18567	-0.24918	-0.114
6.77	-0.191	-0.25169	-0.11895
6.78	-0.19639	-0.25412	-0.12384
6.79	-0.20182	-0.25647	-0.12867
6.8	-0.20729	-0.25877	-0.13343
6.81	-0.21279	-0.26101	-0.13813
6.82	-0.21833	-0.2632	-0.14278
6.83	-0.2239	-0.26536	-0.14737
6.84	-0.22949	-0.26751	-0.1519
6.85	-0.2351	-0.26964	-0.15639
6.86	-0.24072	-0.27177	-0.16084
6.87	-0.24636	-0.27391	-0.16524
6.88	-0.25201	-0.27608	-0.1696
6.89	-0.25766	-0.27828	-0.17393
6.9	-0.26332	-0.28053	-0.17823

6.91	-0.26897	-0.28283	-0.18249
6.92	-0.27461	-0.28519	-0.18673
6.93	-0.28025	-0.28761	-0.19094
6.94	-0.28587	-0.2901	-0.19513
6.95	-0.29148	-0.29264	-0.19928
6.96	-0.29708	-0.29525	-0.20341
6.97	-0.30265	-0.29792	-0.20751
6.98	-0.3082	-0.30066	-0.21159
6.99	-0.31372	-0.30347	-0.21564
7	-0.31922	-0.30635	-0.21967
7.01	-0.32469	-0.30929	-0.22367
7.02	-0.33013	-0.31231	-0.22765
7.03	-0.33553	-0.31541	-0.23161
7.04	-0.3409	-0.31858	-0.23557
7.05	-0.34623	-0.32183	-0.23953
7.06	-0.35152	-0.32515	-0.24348
7.07	-0.35678	-0.32857	-0.24745
7.08	-0.362	-0.33206	-0.25142
7.09	-0.36717	-0.33564	-0.25542
7.1	-0.37231	-0.33931	-0.25944
7.11	-0.3774	-0.34307	-0.26348
7.12	-0.38245	-0.34691	-0.26756
7.13	-0.38745	-0.35082	-0.27167
7.14	-0.39241	-0.3548	-0.27581
7.15	-0.39732	-0.35884	-0.27998
7.16	-0.40219	-0.36293	-0.28418
7.17	-0.40701	-0.36706	-0.28841
7.18	-0.41179	-0.37122	-0.29268
7.19	-0.41652	-0.37541	-0.29698
7.2	-0.4212	-0.37962	-0.30131
7.21	-0.42584	-0.38384	-0.30568
7.22	-0.43043	-0.38808	-0.31008
7.23	-0.43497	-0.39232	-0.3145
7.24	-0.43946	-0.39657	-0.31896
7.25	-0.44391	-0.40084	-0.32343
7.26	-0.44831	-0.40511	-0.32792
7.27	-0.45266	-0.4094	-0.33244
7.28	-0.45697	-0.41369	-0.33696
7.29	-0.46122	-0.418	-0.34149
7.3	-0.46543	-0.42232	-0.34603
7.31	-0.46959	-0.42664	-0.35058
7.32	-0.47371	-0.43098	-0.35513
7.33	-0.47777	-0.43532	-0.35968
7.34	-0.48178	-0.43966	-0.36424
7.35	-0.48574	-0.44401	-0.36879
7.36	-0.48965	-0.44836	-0.37335
7.37	-0.4935	-0.45271	-0.37791
7.38	-0.4973	-0.45706	-0.38246

7.39	-0.50105	-0.46141	-0.38702
7.4	-0.50474	-0.46575	-0.39157
7.41	-0.50838	-0.47008	-0.39611
7.42	-0.51196	-0.47439	-0.40066
7.43	-0.51548	-0.47869	-0.40519
7.44	-0.51894	-0.48297	-0.40972
7.45	-0.52234	-0.48721	-0.41423
7.46	-0.52568	-0.49142	-0.41873
7.47	-0.52896	-0.49559	-0.42322
7.48	-0.53218	-0.49971	-0.42769
7.49	-0.53534	-0.50379	-0.43214
7.5	-0.53843	-0.5078	-0.43657
7.51	-0.54146	-0.51175	-0.44098
7.52	-0.54442	-0.51564	-0.44536
7.53	-0.54732	-0.51947	-0.44971
7.54	-0.55015	-0.52324	-0.45404
7.55	-0.55291	-0.52694	-0.45834
7.56	-0.55559	-0.53058	-0.4626
7.57	-0.55821	-0.53417	-0.46684
7.58	-0.56075	-0.53769	-0.47104
7.59	-0.56322	-0.54115	-0.4752
7.6	-0.56561	-0.54455	-0.47933
7.61	-0.56792	-0.5479	-0.48342
7.62	-0.57016	-0.55118	-0.48746
7.63	-0.57231	-0.55438	-0.49147
7.64	-0.57439	-0.55752	-0.49544
7.65	-0.57638	-0.56058	-0.49936
7.66	-0.57829	-0.56355	-0.50325
7.67	-0.58011	-0.56643	-0.50709
7.68	-0.58184	-0.56922	-0.51088
7.69	-0.58349	-0.57191	-0.51463
7.7	-0.58504	-0.5745	-0.51834
7.71	-0.58651	-0.57698	-0.522
7.72	-0.58788	-0.57935	-0.52561
7.73	-0.58916	-0.58162	-0.52917
7.74	-0.59034	-0.58377	-0.53268
7.75	-0.59143	-0.58582	-0.53613
7.76	-0.59242	-0.58776	-0.53952
7.77	-0.59332	-0.58959	-0.54285
7.78	-0.59411	-0.59131	-0.54612
7.79	-0.59481	-0.59293	-0.54932
7.8	-0.59541	-0.59443	-0.55245
7.81	-0.5959	-0.59583	-0.55551
7.82	-0.5963	-0.59712	-0.5585
7.83	-0.5966	-0.59831	-0.56141
7.84	-0.59681	-0.59938	-0.56426
7.85	-0.59692	-0.60035	-0.56703
7.86	-0.59695	-0.60122	-0.56973

7.87	-0.59689	-0.60197	-0.57235
7.88	-0.59674	-0.60262	-0.5749
7.89	-0.59651	-0.60317	-0.57738
7.9	-0.59621	-0.60361	-0.57978
7.91	-0.59582	-0.60394	-0.58211
7.92	-0.59535	-0.60417	-0.58437
7.93	-0.5948	-0.60429	-0.58655
7.94	-0.59415	-0.6043	-0.58865
7.95	-0.5934	-0.60421	-0.59068
7.96	-0.59255	-0.60402	-0.59263
7.97	-0.59158	-0.60372	-0.5945
7.98	-0.59051	-0.60331	-0.59629
7.99	-0.58931	-0.6028	-0.598
8	-0.58799	-0.60218	-0.59964
8.01	-0.58654	-0.60146	-0.60118
8.02	-0.58496	-0.60063	-0.60265
8.03	-0.58325	-0.59969	-0.60403
8.04	-0.58142	-0.59865	-0.60532
8.05	-0.57947	-0.5975	-0.60652
8.06	-0.57741	-0.59624	-0.60762
8.07	-0.57523	-0.59488	-0.60864
8.08	-0.57294	-0.5934	-0.60955
8.09	-0.57054	-0.59181	-0.61037
8.1	-0.56803	-0.59012	-0.61108
8.11	-0.56542	-0.58831	-0.61169
8.12	-0.5627	-0.58638	-0.6122
8.13	-0.55987	-0.58433	-0.61261
8.14	-0.55693	-0.58216	-0.61291
8.15	-0.55387	-0.57986	-0.61311
8.16	-0.55069	-0.57741	-0.61321
8.17	-0.54738	-0.57483	-0.61321
8.18	-0.54395	-0.5721	-0.6131
8.19	-0.54039	-0.56923	-0.61289
8.2	-0.53669	-0.56619	-0.61258
8.21	-0.53286	-0.56299	-0.61217
8.22	-0.52888	-0.55964	-0.61165
8.23	-0.52477	-0.55612	-0.611
8.24	-0.52053	-0.55243	-0.61022
8.25	-0.51615	-0.54859	-0.6093
8.26	-0.51163	-0.54458	-0.60822
8.27	-0.50697	-0.5404	-0.60698
8.28	-0.50218	-0.53606	-0.60556
8.29	-0.49726	-0.53155	-0.60395
8.3	-0.4922	-0.52687	-0.60214
8.31	-0.48701	-0.52202	-0.60013
8.32	-0.4817	-0.51701	-0.59791
8.33	-0.47626	-0.51183	-0.59548
8.34	-0.47072	-0.50649	-0.59284

8.35	-0.46507	-0.50098	-0.59
8.36	-0.45933	-0.49531	-0.58694
8.37	-0.4535	-0.48947	-0.58368
8.38	-0.44759	-0.48348	-0.58021
8.39	-0.44162	-0.47733	-0.57653
8.4	-0.43558	-0.47102	-0.57264
8.41	-0.42949	-0.46455	-0.56854
8.42	-0.42334	-0.45793	-0.56424
8.43	-0.41714	-0.45115	-0.55974
8.44	-0.4109	-0.44423	-0.55504
8.45	-0.40461	-0.43717	-0.55014
8.46	-0.39828	-0.42996	-0.54506
8.47	-0.3919	-0.42261	-0.53978
8.48	-0.38549	-0.41513	-0.53432
8.49	-0.37904	-0.40752	-0.52868
8.5	-0.37255	-0.39978	-0.52286
8.51	-0.36603	-0.39191	-0.51687
8.52	-0.35948	-0.38393	-0.51071
8.53	-0.3529	-0.37584	-0.50437
8.54	-0.3463	-0.36765	-0.49787
8.55	-0.33967	-0.35938	-0.49121
8.56	-0.33302	-0.35103	-0.48438
8.57	-0.32635	-0.34262	-0.4774
8.58	-0.31966	-0.33415	-0.47026
8.59	-0.31295	-0.32563	-0.46297
8.6	-0.30624	-0.31708	-0.45552
8.61	-0.29951	-0.3085	-0.44793
8.62	-0.29277	-0.29991	-0.44021
8.63	-0.28601	-0.29129	-0.43237
8.64	-0.27925	-0.28266	-0.42444
8.65	-0.27247	-0.27403	-0.41642
8.66	-0.26568	-0.2654	-0.40833
8.67	-0.25888	-0.25677	-0.40019
8.68	-0.25206	-0.24814	-0.39202
8.69	-0.24523	-0.23954	-0.38382
8.7	-0.23838	-0.23095	-0.37563
8.71	-0.23152	-0.22238	-0.36745
8.72	-0.22465	-0.21385	-0.35927
8.73	-0.21778	-0.20536	-0.35111
8.74	-0.21091	-0.19693	-0.34296
8.75	-0.20406	-0.18856	-0.33481
8.76	-0.19723	-0.18026	-0.32667
8.77	-0.19043	-0.17205	-0.31853
8.78	-0.18366	-0.16393	-0.31039
8.79	-0.17694	-0.15591	-0.30224
8.8	-0.17026	-0.148	-0.2941
8.81	-0.16364	-0.14022	-0.28595
8.82	-0.15708	-0.13256	-0.2778

8.83	-0.15059	-0.12502	-0.26966
8.84	-0.14418	-0.11762	-0.26154
8.85	-0.13784	-0.11034	-0.25345
8.86	-0.13159	-0.1032	-0.24538
8.87	-0.12544	-0.09619	-0.23736
8.88	-0.11938	-0.08931	-0.22939
8.89	-0.11343	-0.08257	-0.22147
8.9	-0.10758	-0.07597	-0.21361
8.91	-0.10186	-0.06951	-0.20583
8.92	-0.09624	-0.06318	-0.19811
8.93	-0.09074	-0.05698	-0.19048
8.94	-0.08533	-0.05091	-0.18292
8.95	-0.08003	-0.04495	-0.17544
8.96	-0.07482	-0.03911	-0.16804
8.97	-0.0697	-0.03338	-0.16073
8.98	-0.06467	-0.02774	-0.15351
8.99	-0.05972	-0.0222	-0.14637
9	-0.05485	-0.01675	-0.13933
9.01	-0.05005	-0.01139	-0.13238
9.02	-0.04532	-0.00612	-0.12552
9.03	-0.04066	-0.00094	-0.11877
9.04	-0.03605	0.004151	-0.11212
9.05	-0.03148	0.00914	-0.10557
9.06	-0.02697	0.014028	-0.09913
9.07	-0.02249	0.01881	-0.0928
9.08	-0.01804	0.023484	-0.08658
9.09	-0.01362	0.028046	-0.08047
9.1	-0.00922	0.032493	-0.07449
9.11	-0.00484	0.036823	-0.06862
9.12	-0.00046	0.041043	-0.06287
9.13	0.003913	0.045159	-0.05723
9.14	0.008296	0.049179	-0.05171
9.15	0.012694	0.053112	-0.0463
9.16	0.017112	0.056964	-0.041
9.17	0.02156	0.060745	-0.03581
9.18	0.026043	0.06446	-0.03071
9.19	0.03057	0.068118	-0.02572
9.2	0.035147	0.071727	-0.02083
9.21	0.039781	0.075294	-0.01604
9.22	0.044473	0.078823	-0.01134
9.23	0.049222	0.082317	-0.00674
9.24	0.054028	0.08578	-0.00224
9.25	0.058892	0.089215	0.00218
9.26	0.063813	0.092626	0.006503
9.27	0.06879	0.096017	0.010737
9.28	0.073824	0.099391	0.014881
9.29	0.078915	0.102752	0.018938
9.3	0.084062	0.106103	0.022908

9.31	0.089264	0.109446	0.026793
9.32	0.094519	0.11278	0.030602
9.33	0.099821	0.116102	0.034344
9.34	0.105168	0.119409	0.038028
9.35	0.110556	0.122697	0.041663
9.36	0.11598	0.125965	0.045258
9.37	0.121436	0.129209	0.048823
9.38	0.126921	0.132427	0.052367
9.39	0.13243	0.135614	0.055898
9.4	0.13796	0.138769	0.059426
9.41	0.143506	0.14189	0.06296
9.42	0.149065	0.144982	0.066505
9.43	0.154632	0.148049	0.070066
9.44	0.160204	0.151098	0.073648
9.45	0.165775	0.154135	0.077256
9.46	0.171343	0.157165	0.080894
9.47	0.176902	0.160193	0.084568
9.48	0.182449	0.163227	0.088283
9.49	0.18798	0.166271	0.092042
9.5	0.19349	0.169331	0.095853
9.51	0.198976	0.172413	0.099716
9.52	0.204435	0.175518	0.10363
9.53	0.209867	0.17865	0.107587
9.54	0.215269	0.181808	0.111583
9.55	0.220639	0.184997	0.115612
9.56	0.225976	0.188216	0.119668
9.57	0.231279	0.191469	0.123746
9.58	0.236545	0.194757	0.12784
9.59	0.241774	0.198082	0.131945
9.6	0.246962	0.201445	0.136056
9.61	0.252109	0.204849	0.140167
9.62	0.257215	0.208294	0.144285
9.63	0.262278	0.211781	0.148415
9.64	0.267298	0.21531	0.152564
9.65	0.272275	0.218882	0.156737
9.66	0.277208	0.222496	0.160943
9.67	0.282097	0.226154	0.165186
9.68	0.286941	0.229856	0.169473
9.69	0.29174	0.233603	0.173811
9.7	0.296493	0.237394	0.178207
9.71	0.301201	0.241231	0.182663
9.72	0.305861	0.245113	0.187174
9.73	0.310475	0.249038	0.191732
9.74	0.315042	0.253006	0.196328
9.75	0.319561	0.257016	0.200953
9.76	0.324032	0.261068	0.2056
9.77	0.328454	0.26516	0.21026
9.78	0.332828	0.269292	0.214924

9.79	0.337153	0.273463	0.219584
9.8	0.341428	0.277672	0.224232
9.81	0.345654	0.281918	0.22886
9.82	0.349828	0.286198	0.233468
9.83	0.353951	0.290508	0.238054
9.84	0.358022	0.294845	0.24262
9.85	0.362041	0.299204	0.247164
9.86	0.366005	0.303583	0.251688
9.87	0.369915	0.307977	0.256191
9.88	0.37377	0.312384	0.260673
9.89	0.377569	0.316799	0.265135
9.9	0.381311	0.32122	0.269575
9.91	0.384996	0.325642	0.273994
9.92	0.388622	0.330062	0.278392
9.93	0.392189	0.334477	0.282767
9.94	0.395695	0.338883	0.287118
9.95	0.39914	0.343279	0.291445
9.96	0.402521	0.34766	0.295748
9.97	0.405839	0.352024	0.300024
9.98	0.409092	0.356366	0.304273
9.99	0.412279	0.360685	0.308494
10	0.415399	0.364977	0.312687
10.01	0.41845	0.369238	0.316851
10.02	0.421433	0.373465	0.320983
10.03	0.424345	0.377655	0.325084
10.04	0.427184	0.381803	0.32915
10.05	0.42995	0.385907	0.333181
10.06	0.432642	0.389962	0.337176
10.07	0.435257	0.393965	0.341133
10.08	0.437795	0.397913	0.34505
10.09	0.440255	0.401801	0.348926
10.1	0.442634	0.405626	0.35276
10.11	0.444932	0.409385	0.356551
10.12	0.447148	0.413076	0.3603
10.13	0.44928	0.416695	0.364009
10.14	0.451327	0.420241	0.367682
10.15	0.453289	0.423712	0.37132
10.16	0.455164	0.427105	0.374926
10.17	0.456951	0.430418	0.378501
10.18	0.45865	0.433648	0.382048
10.19	0.460258	0.436795	0.38557
10.2	0.461776	0.439854	0.389069
10.21	0.463201	0.442825	0.392545
10.22	0.464534	0.445706	0.395999
10.23	0.465774	0.448498	0.399427
10.24	0.46692	0.451198	0.402828
10.25	0.467971	0.453807	0.406199
10.26	0.468928	0.456324	0.409539

10.27	0.469788	0.458749	0.412846
10.28	0.470553	0.461081	0.416117
10.29	0.471221	0.463318	0.41935
10.3	0.471792	0.465462	0.422543
10.31	0.472265	0.46751	0.425695
10.32	0.472641	0.469463	0.428803
10.33	0.472924	0.471318	0.431864
10.34	0.473114	0.473076	0.434878
10.35	0.473213	0.474735	0.437841
10.36	0.473224	0.476294	0.440752
10.37	0.473147	0.477753	0.443608
10.38	0.472985	0.47911	0.446408
10.39	0.47274	0.480364	0.449149
10.4	0.472413	0.481515	0.451829
10.41	0.472005	0.482561	0.454446
10.42	0.471512	0.483502	0.456999
10.43	0.47093	0.484336	0.459485
10.44	0.470253	0.485062	0.461902
10.45	0.469476	0.48568	0.46425
10.46	0.468594	0.486187	0.466526
10.47	0.467603	0.486584	0.468728
10.48	0.466497	0.486869	0.470855
10.49	0.465271	0.487041	0.472904
10.5	0.463921	0.487099	0.474875
10.51	0.462442	0.487042	0.476765
10.52	0.460836	0.48687	0.478571
10.53	0.459104	0.486585	0.480289
10.54	0.457248	0.486186	0.481917
10.55	0.45527	0.485675	0.483451
10.56	0.453172	0.485052	0.484888
10.57	0.450955	0.484317	0.486224
10.58	0.448622	0.483472	0.487457
10.59	0.446175	0.482516	0.488583
10.6	0.443615	0.481452	0.489599
10.61	0.440944	0.480277	0.490502
10.62	0.438159	0.478992	0.491291
10.63	0.435257	0.477591	0.491966
10.64	0.432236	0.476073	0.492526
10.65	0.429092	0.474434	0.492972
10.66	0.425822	0.472671	0.493304
10.67	0.422424	0.470783	0.49352
10.68	0.418894	0.468764	0.493621
10.69	0.415229	0.466614	0.493606
10.7	0.411427	0.464328	0.493475
10.71	0.407485	0.461903	0.493228
10.72	0.403403	0.459337	0.492857
10.73	0.399184	0.456624	0.492356
10.74	0.394829	0.453761	0.491719

10.75	0.39034	0.450745	0.490939
10.76	0.385717	0.447571	0.490009
10.77	0.380963	0.444235	0.488922
10.78	0.376078	0.440733	0.487672
10.79	0.371066	0.437062	0.486252
10.8	0.365926	0.433218	0.484654
10.81	0.360662	0.429197	0.482874
10.82	0.355279	0.424995	0.480908
10.83	0.349783	0.420611	0.478755
10.84	0.344181	0.41604	0.476412
10.85	0.338479	0.411281	0.473879
10.86	0.332684	0.40633	0.471153
10.87	0.326801	0.401184	0.468232
10.88	0.320838	0.395841	0.465115
10.89	0.3148	0.390298	0.461799
10.9	0.308695	0.384551	0.458284
10.91	0.302528	0.378599	0.454568
10.92	0.296301	0.372445	0.450653
10.93	0.290019	0.36609	0.446545
10.94	0.283684	0.359538	0.442247
10.95	0.277299	0.352791	0.437764
10.96	0.270866	0.345854	0.433098
10.97	0.264389	0.33873	0.428255
10.98	0.25787	0.33142	0.423238
10.99	0.251313	0.323928	0.418051
11	0.244719	0.316258	0.412698
11.01	0.238092	0.308412	0.407184
11.02	0.231434	0.300397	0.401508
11.03	0.224746	0.29222	0.395671
11.04	0.21803	0.283888	0.389674
11.05	0.211289	0.275406	0.383517
11.06	0.204523	0.266782	0.3772
11.07	0.197735	0.258023	0.370725
11.08	0.190926	0.249134	0.364091
11.09	0.184098	0.240124	0.3573
11.1	0.177254	0.230998	0.350351
11.11	0.170394	0.221763	0.343247
11.12	0.163521	0.212428	0.335996
11.13	0.156639	0.203	0.328609
11.14	0.149749	0.193487	0.321097
11.15	0.142854	0.183897	0.313471
11.16	0.135957	0.174238	0.30574
11.17	0.12906	0.164517	0.297916
11.18	0.122167	0.154744	0.290008
11.19	0.115279	0.144925	0.282028
11.2	0.1084	0.135068	0.273986
11.21	0.101533	0.125183	0.265892
11.22	0.094686	0.115279	0.257756

11.23	0.087868	0.105369	0.249588
11.24	0.08109	0.095463	0.241395
11.25	0.07436	0.085573	0.233188
11.26	0.067687	0.075709	0.224976
11.27	0.061082	0.065884	0.216769
11.28	0.054553	0.056109	0.208574
11.29	0.048109	0.046394	0.200402
11.3	0.041761	0.036752	0.192262
11.31	0.035516	0.027192	0.184161
11.32	0.029375	0.017719	0.176103
11.33	0.023339	0.008339	0.168088
11.34	0.017409	-0.00095	0.160116
11.35	0.011585	-0.01013	0.15219
11.36	0.005868	-0.01921	0.144309
11.37	0.000257	-0.02818	0.136476
11.38	-0.00525	-0.03703	0.12869
11.39	-0.01064	-0.04577	0.120953
11.4	-0.01593	-0.05439	0.113266
11.41	-0.02111	-0.06288	0.105631
11.42	-0.02619	-0.07123	0.098051
11.43	-0.03118	-0.07945	0.09053
11.44	-0.03607	-0.08751	0.083073
11.45	-0.04089	-0.09542	0.075686
11.46	-0.04563	-0.10316	0.068371
11.47	-0.0503	-0.11073	0.061134
11.48	-0.05492	-0.11812	0.053979
11.49	-0.05948	-0.12533	0.046911
11.5	-0.06399	-0.13234	0.039933
11.51	-0.06846	-0.13915	0.03305
11.52	-0.0729	-0.14575	0.02626
11.53	-0.07731	-0.15216	0.01956
11.54	-0.0817	-0.15836	0.012947
11.55	-0.08608	-0.16436	0.006419
11.56	-0.09046	-0.17016	#######
11.57	-0.09484	-0.17576	-0.00639
11.58	-0.09923	-0.18117	-0.01269
11.59	-0.10364	-0.18637	-0.0189
11.6	-0.10807	-0.19138	-0.02505
11.61	-0.11253	-0.19619	-0.03112
11.62	-0.11703	-0.2008	-0.03713
11.63	-0.12157	-0.20523	-0.04307
11.64	-0.12615	-0.20948	-0.04894
11.65	-0.13078	-0.21356	-0.05473
11.66	-0.13545	-0.21747	-0.06046
11.67	-0.14019	-0.22122	-0.06612
11.68	-0.14498	-0.22482	-0.0717
11.69	-0.14984	-0.22826	-0.07721
11.7	-0.15476	-0.23156	-0.08265

11.71	-0.15975	-0.23473	-0.08802
11.72	-0.1648	-0.23776	-0.09331
11.73	-0.16992	-0.24068	-0.09853
11.74	-0.1751	-0.24348	-0.10369
11.75	-0.18034	-0.24618	-0.10877
11.76	-0.18562	-0.24878	-0.11379
11.77	-0.19096	-0.2513	-0.11874
11.78	-0.19634	-0.25373	-0.12363
11.79	-0.20176	-0.2561	-0.12845
11.8	-0.20722	-0.2584	-0.13321
11.81	-0.21272	-0.26065	-0.13791
11.82	-0.21824	-0.26285	-0.14256
11.83	-0.22379	-0.26502	-0.14714
11.84	-0.22937	-0.26717	-0.15168
11.85	-0.23497	-0.26931	-0.15617
11.86	-0.24058	-0.27145	-0.16061
11.87	-0.2462	-0.2736	-0.16501
11.88	-0.25184	-0.27578	-0.16938
11.89	-0.25748	-0.27799	-0.1737
11.9	-0.26312	-0.28024	-0.178
11.91	-0.26876	-0.28255	-0.18227
11.92	-0.2744	-0.28492	-0.18651
11.93	-0.28002	-0.28734	-0.19072
11.94	-0.28564	-0.28983	-0.1949
11.95	-0.29124	-0.29238	-0.19906
11.96	-0.29683	-0.29499	-0.20319
11.97	-0.3024	-0.29767	-0.20729
11.98	-0.30795	-0.30041	-0.21137
11.99	-0.31347	-0.30322	-0.21542
12	-0.31896	-0.3061	-0.21945
12.01	-0.32443	-0.30905	-0.22345
12.02	-0.32986	-0.31207	-0.22743
12.03	-0.33526	-0.31517	-0.2314
12.04	-0.34062	-0.31834	-0.23536
12.05	-0.34595	-0.3216	-0.23931
12.06	-0.35124	-0.32493	-0.24327
12.07	-0.35649	-0.32834	-0.24724
12.08	-0.3617	-0.33184	-0.25122
12.09	-0.36687	-0.33542	-0.25521
12.1	-0.372	-0.33909	-0.25923
12.11	-0.37709	-0.34285	-0.26328
12.12	-0.38213	-0.34669	-0.26736
12.13	-0.38713	-0.3506	-0.27146
12.14	-0.39208	-0.35458	-0.2756
12.15	-0.39699	-0.35862	-0.27977
12.16	-0.40185	-0.3627	-0.28397
12.17	-0.40667	-0.36683	-0.28821
12.18	-0.41145	-0.37099	-0.29247

12.19	-0.41618	-0.37518	-0.29677
12.2	-0.42086	-0.37939	-0.3011
12.21	-0.4255	-0.38361	-0.30547
12.22	-0.43009	-0.38784	-0.30987
12.23	-0.43464	-0.39208	-0.31429
12.24	-0.43914	-0.39633	-0.31874
12.25	-0.44359	-0.4006	-0.32322
12.26	-0.448	-0.40487	-0.32771
12.27	-0.45235	-0.40915	-0.33222
12.28	-0.45666	-0.41345	-0.33674
12.29	-0.46092	-0.41775	-0.34127
12.3	-0.46513	-0.42206	-0.34581
12.31	-0.46929	-0.42639	-0.35036
12.32	-0.47341	-0.43072	-0.35491
12.33	-0.47746	-0.43506	-0.35946
12.34	-0.48147	-0.4394	-0.36401
12.35	-0.48543	-0.44375	-0.36856
12.36	-0.48933	-0.4481	-0.37312
12.37	-0.49318	-0.45245	-0.37767
12.38	-0.49697	-0.45679	-0.38222
12.39	-0.50071	-0.46113	-0.38677
12.4	-0.5044	-0.46547	-0.39132
12.41	-0.50803	-0.4698	-0.39586
12.42	-0.5116	-0.47411	-0.4004
12.43	-0.51511	-0.47841	-0.40493
12.44	-0.51857	-0.48268	-0.40946
12.45	-0.52197	-0.48692	-0.41397
12.46	-0.52531	-0.49113	-0.41847
12.47	-0.52858	-0.49529	-0.42295
12.48	-0.5318	-0.49941	-0.42741
12.49	-0.53495	-0.50348	-0.43186
12.5	-0.53804	-0.50749	-0.43628
12.51	-0.54107	-0.51144	-0.44069
12.52	-0.54403	-0.51533	-0.44506
12.53	-0.54692	-0.51915	-0.44942
12.54	-0.54975	-0.52291	-0.45374
12.55	-0.55251	-0.52661	-0.45803
12.56	-0.55519	-0.53025	-0.4623
12.57	-0.55781	-0.53383	-0.46653
12.58	-0.56035	-0.53735	-0.47072
12.59	-0.56282	-0.54081	-0.47489
12.6	-0.56521	-0.54421	-0.47901
12.61	-0.56752	-0.54755	-0.48309
12.62	-0.56975	-0.55082	-0.48714
12.63	-0.57191	-0.55403	-0.49114
12.64	-0.57398	-0.55716	-0.49511
12.65	-0.57597	-0.56022	-0.49903
12.66	-0.57787	-0.56319	-0.50291

12.67	-0.57968	-0.56607	-0.50674
12.68	-0.58141	-0.56885	-0.51054
12.69	-0.58305	-0.57154	-0.51428
12.7	-0.58459	-0.57413	-0.51799
12.71	-0.58605	-0.5766	-0.52164
12.72	-0.58741	-0.57897	-0.52525
12.73	-0.58867	-0.58123	-0.52881
12.74	-0.58985	-0.58338	-0.53232
12.75	-0.59093	-0.58543	-0.53576
12.76	-0.59192	-0.58736	-0.53915
12.77	-0.59282	-0.58919	-0.54248
12.78	-0.59362	-0.59092	-0.54575
12.79	-0.59434	-0.59253	-0.54894
12.8	-0.59496	-0.59404	-0.55207
12.81	-0.59549	-0.59544	-0.55513
12.82	-0.59593	-0.59673	-0.55812
12.83	-0.59627	-0.59792	-0.56103
12.84	-0.59651	-0.599	-0.56387
12.85	-0.59666	-0.59997	-0.56664
12.86	-0.5967	-0.60083	-0.56934
12.87	-0.59663	-0.60159	-0.57196
12.88	-0.59646	-0.60224	-0.57451
12.89	-0.59618	-0.60278	-0.57699
12.9	-0.59579	-0.60321	-0.57939
12.91	-0.59529	-0.60353	-0.58172
12.92	-0.59467	-0.60375	-0.58397
12.93	-0.59393	-0.60385	-0.58614
12.94	-0.59308	-0.60385	-0.58824
12.95	-0.59211	-0.60374	-0.59025
12.96	-0.59101	-0.60352	-0.59219
12.97	-0.5898	-0.60319	-0.59405
12.98	-0.58847	-0.60275	-0.59583
12.99	-0.58701	-0.6022	-0.59752
13	-0.58543	-0.60155	-0.59913
13.01	-0.58373	-0.60078	-0.60066
13.02	-0.5819	-0.59991	-0.60209
13.03	-0.57996	-0.59892	-0.60344
13.04	-0.5779	-0.59782	-0.6047
13.05	-0.57573	-0.5966	-0.60587
13.06	-0.57345	-0.59527	-0.60693
13.07	-0.57107	-0.59383	-0.60791
13.08	-0.56859	-0.59227	-0.60878
13.09	-0.56601	-0.59059	-0.60955
13.1	-0.56334	-0.58879	-0.61022
13.11	-0.56058	-0.58687	-0.61078
13.12	-0.55772	-0.58483	-0.61123
13.13	-0.55477	-0.58266	-0.61158
13.14	-0.55173	-0.58035	-0.61182

13.15	-0.54858	-0.57791	-0.61195
13.16	-0.54532	-0.57532	-0.61198
13.17	-0.54196	-0.57259	-0.6119
13.18	-0.53849	-0.56971	-0.61171
13.19	-0.5349	-0.56667	-0.61141
13.2	-0.5312	-0.56347	-0.61101
13.21	-0.52738	-0.5601	-0.61049
13.22	-0.52344	-0.55657	-0.60986
13.23	-0.51938	-0.55288	-0.60909
13.24	-0.51521	-0.54902	-0.60819
13.25	-0.51093	-0.545	-0.60714
13.26	-0.50653	-0.54082	-0.60594
13.27	-0.50202	-0.53648	-0.60456
13.28	-0.4974	-0.53198	-0.603
13.29	-0.49268	-0.52731	-0.60126
13.3	-0.48784	-0.52249	-0.59932
13.31	-0.4829	-0.51751	-0.59717
13.32	-0.47786	-0.51237	-0.59481
13.33	-0.47271	-0.50708	-0.59225
13.34	-0.46747	-0.50163	-0.58948
13.35	-0.46213	-0.49602	-0.5865
13.36	-0.4567	-0.49027	-0.58333
13.37	-0.45119	-0.48436	-0.57994
13.38	-0.44558	-0.47831	-0.57636
13.39	-0.4399	-0.47211	-0.57257
13.4	-0.43413	-0.46577	-0.56859
13.41	-0.42829	-0.45928	-0.5644
13.42	-0.42238	-0.45265	-0.56002
13.43	-0.4164	-0.44588	-0.55544
13.44	-0.41035	-0.43898	-0.55068
13.45	-0.40424	-0.43196	-0.54572
13.46	-0.39809	-0.4248	-0.54059
13.47	-0.39188	-0.41752	-0.53528
13.48	-0.38562	-0.41012	-0.52979
13.49	-0.37932	-0.40261	-0.52412
13.5	-0.37299	-0.39498	-0.51829
13.51	-0.36662	-0.38725	-0.51229
13.52	-0.36022	-0.37941	-0.50613
13.53	-0.35379	-0.37148	-0.49981
13.54	-0.34733	-0.36347	-0.49334
13.55	-0.34084	-0.35539	-0.48671
13.56	-0.33432	-0.34724	-0.47993
13.57	-0.32778	-0.33904	-0.473
13.58	-0.32121	-0.33079	-0.46593
13.59	-0.31461	-0.3225	-0.45872
13.6	-0.30799	-0.31418	-0.45137
13.61	-0.30134	-0.30584	-0.44389
13.62	-0.29467	-0.29748	-0.4363

13.63	-0.28797	-0.28911	-0.42859
13.64	-0.28126	-0.28073	-0.4208
13.65	-0.27452	-0.27234	-0.41294
13.66	-0.26776	-0.26396	-0.40502
13.67	-0.26098	-0.25557	-0.39705
13.68	-0.25418	-0.2472	-0.38906
13.69	-0.24735	-0.23883	-0.38106
13.7	-0.24051	-0.23048	-0.37306
13.71	-0.23365	-0.22214	-0.36508
13.72	-0.22677	-0.21384	-0.35711
13.73	-0.21989	-0.20557	-0.34916
13.74	-0.21301	-0.19734	-0.34122
13.75	-0.20613	-0.18917	-0.33328
13.76	-0.19926	-0.18107	-0.32535
13.77	-0.1924	-0.17304	-0.31742
13.78	-0.18557	-0.16509	-0.3095
13.79	-0.17877	-0.15724	-0.30157
13.8	-0.172	-0.14949	-0.29363
13.81	-0.16528	-0.14185	-0.28569
13.82	-0.1586	-0.13432	-0.27774
13.83	-0.15198	-0.1269	-0.2698
13.84	-0.14542	-0.1196	-0.26187
13.85	-0.13893	-0.11243	-0.25396
13.86	-0.13253	-0.10537	-0.24608
13.87	-0.12622	-0.09843	-0.23822
13.88	-0.12	-0.09163	-0.23041
13.89	-0.1139	-0.08494	-0.22265
13.9	-0.1079	-0.07839	-0.21494
13.91	-0.10203	-0.07197	-0.20728
13.92	-0.09627	-0.06568	-0.19969
13.93	-0.09063	-0.0595	-0.19217
13.94	-0.0851	-0.05344	-0.18472
13.95	-0.07968	-0.04749	-0.17733
13.96	-0.07436	-0.04165	-0.17002
13.97	-0.06914	-0.03591	-0.16278
13.98	-0.06403	-0.03026	-0.15562
13.99	-0.059	-0.0247	-0.14854
14	-0.05407	-0.01923	-0.14154
14.01	-0.04922	-0.01383	-0.13462
14.02	-0.04446	-0.00852	-0.1278
14.03	-0.03976	-0.0033	-0.12106
14.04	-0.03514	0.001836	-0.11442
14.05	-0.03057	0.006876	-0.10787
14.06	-0.02606	0.01182	-0.10143
14.07	-0.0216	0.016662	-0.09508
14.08	-0.01718	0.021401	-0.08885
14.09	-0.01279	0.026031	-0.08272
14.1	-0.00843	0.03055	-0.0767

14.11	-0.00409	0.034955	-0.07079
14.12	0.00023	0.039252	-0.065
14.13	0.00455	0.043449	-0.05931
14.14	0.008872	0.047553	-0.05374
14.15	0.013205	0.05157	-0.04827
14.16	0.017557	0.055508	-0.0429
14.17	0.021936	0.059375	-0.03764
14.18	0.02635	0.063177	-0.03247
14.19	0.030808	0.066923	-0.02741
14.2	0.035317	0.070618	-0.02244
14.21	0.039885	0.07427	-0.01756
14.22	0.044513	0.077883	-0.01278
14.23	0.0492	0.08146	-0.00809
14.24	0.053949	0.085005	-0.0035
14.25	0.058758	0.08852	0.001004
14.26	0.063628	0.092009	0.005414
14.27	0.068559	0.095475	0.009732
14.28	0.073552	0.098923	0.013959
14.29	0.078607	0.102354	0.018095
14.3	0.083724	0.105772	0.022141
14.31	0.088903	0.10918	0.026098
14.32	0.09414	0.112576	0.029975
14.33	0.09943	0.115957	0.03378
14.34	0.10477	0.119321	0.037523
14.35	0.110154	0.122664	0.041214
14.36	0.115577	0.125983	0.044861
14.37	0.121036	0.129277	0.048474
14.38	0.126527	0.132541	0.052062
14.39	0.132043	0.135774	0.055635
14.4	0.137582	0.138973	0.059202
14.41	0.143138	0.142135	0.062772
14.42	0.148707	0.145266	0.06635
14.43	0.154285	0.148371	0.069941
14.44	0.159867	0.151455	0.073552
14.45	0.16545	0.154524	0.077186
14.46	0.171029	0.157582	0.080848
14.47	0.1766	0.160637	0.084545
14.48	0.182159	0.163693	0.088281
14.49	0.1877	0.166756	0.092061
14.5	0.193221	0.169831	0.09589
14.51	0.198717	0.172923	0.099772
14.52	0.204187	0.176035	0.103702
14.53	0.209627	0.179169	0.107675
14.54	0.215037	0.182326	0.111686
14.55	0.220415	0.18551	0.115728
14.56	0.22576	0.188721	0.119795
14.57	0.231068	0.191963	0.123883
14.58	0.236339	0.195237	0.127986

14.59	0.241572	0.198545	0.132097
14.6	0.246763	0.201889	0.136211
14.61	0.251912	0.205271	0.140325
14.62	0.257018	0.208692	0.144443
14.63	0.262081	0.212153	0.148571
14.64	0.2671	0.215655	0.152716
14.65	0.272074	0.219198	0.156884
14.66	0.277003	0.222784	0.161082
14.67	0.281886	0.226413	0.165317
14.68	0.286723	0.230087	0.169595
14.69	0.291514	0.233805	0.173922
14.7	0.296257	0.23757	0.178305
14.71	0.300953	0.24138	0.182748
14.72	0.305601	0.245237	0.187245
14.73	0.310202	0.249138	0.191789
14.74	0.314754	0.253082	0.19637
14.75	0.319258	0.257069	0.20098
14.76	0.323714	0.261097	0.205611
14.77	0.328122	0.265165	0.210255
14.78	0.332481	0.269272	0.214903
14.79	0.336792	0.273417	0.219547
14.8	0.341054	0.2776	0.224179
14.81	0.345267	0.281817	0.228792
14.82	0.34943	0.286067	0.233384
14.83	0.353544	0.290347	0.237956
14.84	0.357606	0.294653	0.242506
14.85	0.361616	0.298981	0.247036
14.86	0.365574	0.30333	0.251545
14.87	0.369478	0.307695	0.256033
14.88	0.373328	0.312073	0.260499
14.89	0.377123	0.316461	0.264945
14.9	0.380862	0.320857	0.269369
14.91	0.384545	0.325255	0.273771
14.92	0.38817	0.329655	0.278152
14.93	0.391735	0.334051	0.282509
14.94	0.39524	0.338441	0.286844
14.95	0.398684	0.342821	0.291154
14.96	0.402063	0.347189	0.29544
14.97	0.405378	0.351541	0.2997
14.98	0.408627	0.355873	0.303934
14.99	0.411809	0.360182	0.308141
15	0.414922	0.364466	0.312322
15.01	0.417964	0.36872	0.316474
15.02	0.420936	0.372941	0.320596
15.03	0.423836	0.377126	0.324688
15.04	0.426664	0.38127	0.328746
15.05	0.429418	0.385371	0.332771
15.06	0.432098	0.389424	0.33676

15.07	0.434704	0.393425	0.340712
15.08	0.437233	0.397372	0.344625
15.09	0.439687	0.40126	0.348497
15.1	0.442063	0.405085	0.352328
15.11	0.444361	0.408845	0.356116
15.12	0.44658	0.412537	0.359863
15.13	0.448718	0.416158	0.363571
15.14	0.450773	0.419706	0.367242
15.15	0.452745	0.423179	0.370878
15.16	0.454631	0.426575	0.374483
15.17	0.456429	0.42989	0.378058
15.18	0.458139	0.433124	0.381604
15.19	0.459759	0.436273	0.385126
15.2	0.461287	0.439336	0.388624
15.21	0.462723	0.442309	0.3921
15.22	0.464064	0.445193	0.395554
15.23	0.465311	0.447987	0.398982
15.24	0.466463	0.450689	0.402383
15.25	0.467519	0.453301	0.405754
15.26	0.468478	0.455819	0.409094
15.27	0.46934	0.458245	0.412399
15.28	0.470105	0.460578	0.41567
15.29	0.470771	0.462816	0.418902
15.3	0.471338	0.46496	0.422094
15.31	0.471806	0.467008	0.425244
15.32	0.472177	0.46896	0.42835
15.33	0.472451	0.470814	0.431409
15.34	0.472632	0.47257	0.43442
15.35	0.472722	0.474228	0.437381
15.36	0.472722	0.475785	0.440289
15.37	0.472634	0.477241	0.443142
15.38	0.472461	0.478595	0.445938
15.39	0.472205	0.479846	0.448676
15.4	0.471868	0.480994	0.451353
15.41	0.47145	0.482037	0.453966
15.42	0.470948	0.482974	0.456515
15.43	0.470357	0.483804	0.458998
15.44	0.469673	0.484527	0.461412
15.45	0.46889	0.485141	0.463756
15.46	0.468003	0.485645	0.466028
15.47	0.467008	0.486038	0.468227
15.48	0.465901	0.48632	0.47035
15.49	0.464675	0.486489	0.472396
15.5	0.463327	0.486544	0.474363
15.51	0.461853	0.486484	0.47625
15.52	0.460252	0.48631	0.478052
15.53	0.458525	0.486023	0.479768
15.54	0.456674	0.485622	0.481392

15.55	0.454699	0.485109	0.482923
15.56	0.452602	0.484484	0.484357
15.57	0.450383	0.483747	0.48569
15.58	0.448043	0.4829	0.48692
15.59	0.445583	0.481942	0.488042
15.6	0.443005	0.480875	0.489054
15.61	0.440309	0.479697	0.489953
15.62	0.437493	0.478408	0.490737
15.63	0.434556	0.477004	0.491408
15.64	0.431497	0.475482	0.491964
15.65	0.428314	0.473838	0.492405
15.66	0.425005	0.47207	0.492732
15.67	0.421569	0.470176	0.492943
15.68	0.418004	0.468151	0.493038
15.69	0.414309	0.465993	0.493018
15.7	0.410482	0.463699	0.492882
15.71	0.406522	0.461266	0.492628
15.72	0.402429	0.458689	0.492251
15.73	0.398204	0.455967	0.491744
15.74	0.393848	0.453093	0.491101
15.75	0.389361	0.450065	0.490314
15.76	0.384743	0.446879	0.489377
15.77	0.379996	0.443532	0.488282
15.78	0.37512	0.440018	0.487023
15.79	0.370115	0.436335	0.485594
15.8	0.364983	0.432478	0.483987
15.81	0.359724	0.428444	0.482196
15.82	0.354345	0.42423	0.480219
15.83	0.348853	0.419833	0.478054
15.84	0.343254	0.41525	0.475699
15.85	0.337555	0.410479	0.473154
15.86	0.331762	0.405515	0.470415
15.87	0.325884	0.400357	0.467482
15.88	0.319925	0.395002	0.464352
15.89	0.313893	0.389446	0.461025
15.9	0.307796	0.383687	0.457497
15.91	0.301638	0.377723	0.453769
15.92	0.295423	0.371556	0.449844
15.93	0.289154	0.365189	0.445726
15.94	0.282834	0.358625	0.441418
15.95	0.276464	0.351867	0.436924
15.96	0.270048	0.344918	0.43225
15.97	0.263589	0.337782	0.427397
15.98	0.257087	0.330462	0.422372
15.99	0.250548	0.32296	0.417177
16	0.243972	0.315279	0.411816
16.01	0.237363	0.307424	0.406294
16.02	0.230721	0.299401	0.40061

16.03	0.22405	0.291215	0.394766
16.04	0.217351	0.282875	0.388762
16.05	0.210624	0.274387	0.382599
16.06	0.203873	0.265758	0.376277
16.07	0.197099	0.256994	0.369796
16.08	0.190303	0.248103	0.363157
16.09	0.183488	0.239091	0.356361
16.1	0.176655	0.229965	0.349408
16.11	0.169805	0.220731	0.3423
16.12	0.162942	0.211399	0.335046
16.13	0.156068	0.201975	0.327658
16.14	0.149186	0.192467	0.320144
16.15	0.142298	0.182884	0.312517
16.16	0.135408	0.173232	0.304786
16.17	0.128519	0.163521	0.296963
16.18	0.121632	0.153757	0.289058
16.19	0.114751	0.143949	0.281082
16.2	0.107878	0.134104	0.273045
16.21	0.101018	0.124232	0.264957
16.22	0.094178	0.114342	0.256829
16.23	0.087369	0.104446	0.248669
16.24	0.080598	0.094555	0.240486
16.25	0.073877	0.084681	0.232289
16.26	0.067213	0.074834	0.224088
16.27	0.060617	0.065027	0.215892
16.28	0.054098	0.055269	0.20771
16.29	0.047665	0.045574	0.19955
16.3	0.041328	0.03595	0.191423
16.31	0.035094	0.02641	0.183335
16.32	0.028965	0.016958	0.17529
16.33	0.022941	0.007599	0.167287
16.34	0.017022	-0.00166	0.159329
16.35	0.011207	-0.01083	0.151416
16.36	0.005498	-0.01988	0.14355
16.37	-0.00011	-0.02883	0.13573
16.38	-0.00561	-0.03766	0.127959
16.39	-0.011	-0.04638	0.120237
16.4	-0.01629	-0.05497	0.112565
16.41	-0.02148	-0.06344	0.104945
16.42	-0.02657	-0.07177	0.09738
16.43	-0.03156	-0.07996	0.089876
16.44	-0.03647	-0.08801	0.082435
16.45	-0.0413	-0.09589	0.075064
16.46	-0.04606	-0.10361	0.067766
16.47	-0.05075	-0.11116	0.060545
16.48	-0.05537	-0.11853	0.053405
16.49	-0.05995	-0.12572	0.046352
16.5	-0.06448	-0.13271	0.03939

16.51	-0.06896	-0.1395	0.032521
16.52	-0.07341	-0.14608	0.025745
16.53	-0.07783	-0.15247	0.019058
16.54	-0.08223	-0.15865	0.012458
16.55	-0.08662	-0.16464	0.005942
16.56	-0.091	-0.17042	-0.00049
16.57	-0.09538	-0.176	-0.00685
16.58	-0.09977	-0.18139	-0.01313
16.59	-0.10418	-0.18658	-0.01934
16.6	-0.10861	-0.19157	-0.02547
16.61	-0.11307	-0.19636	-0.03154
16.62	-0.11756	-0.20097	-0.03754
16.63	-0.12208	-0.20538	-0.04347
16.64	-0.12665	-0.20962	-0.04933
16.65	-0.13127	-0.21369	-0.05512
16.66	-0.13593	-0.21759	-0.06084
16.67	-0.14065	-0.22133	-0.06649
16.68	-0.14542	-0.22492	-0.07207
16.69	-0.15026	-0.22836	-0.07757
16.7	-0.15516	-0.23166	-0.08301
16.71	-0.16013	-0.23482	-0.08837
16.72	-0.16516	-0.23785	-0.09366
16.73	-0.17026	-0.24077	-0.09887
16.74	-0.17542	-0.24357	-0.10402
16.75	-0.18063	-0.24627	-0.1091
16.76	-0.1859	-0.24888	-0.11411
16.77	-0.19121	-0.2514	-0.11906
16.78	-0.19657	-0.25384	-0.12394
16.79	-0.20197	-0.2562	-0.12876
16.8	-0.20741	-0.25851	-0.13352
16.81	-0.21289	-0.26075	-0.13821
16.82	-0.2184	-0.26296	-0.14285
16.83	-0.22393	-0.26513	-0.14744
16.84	-0.22949	-0.26728	-0.15197
16.85	-0.23508	-0.26942	-0.15646
16.86	-0.24067	-0.27156	-0.1609
16.87	-0.24628	-0.27371	-0.1653
16.88	-0.2519	-0.27588	-0.16966
16.89	-0.25752	-0.27809	-0.17398
16.9	-0.26315	-0.28034	-0.17827
16.91	-0.26877	-0.28265	-0.18254
16.92	-0.27438	-0.28502	-0.18677
16.93	-0.27999	-0.28744	-0.19098
16.94	-0.28558	-0.28993	-0.19516
16.95	-0.29117	-0.29248	-0.19931
16.96	-0.29673	-0.29509	-0.20343
16.97	-0.30228	-0.29777	-0.20752
16.98	-0.30781	-0.30051	-0.21159

16.99	-0.31331	-0.30332	-0.21564
17	-0.31878	-0.3062	-0.21965
17.01	-0.32423	-0.30915	-0.22365
17.02	-0.32964	-0.31217	-0.22762
17.03	-0.33502	-0.31526	-0.23158
17.04	-0.34037	-0.31843	-0.23553
17.05	-0.34568	-0.32168	-0.23947
17.06	-0.35096	-0.32501	-0.24342
17.07	-0.3562	-0.32841	-0.24737
17.08	-0.3614	-0.3319	-0.25134
17.09	-0.36656	-0.33548	-0.25533
17.1	-0.37167	-0.33914	-0.25934
17.11	-0.37675	-0.34288	-0.26337
17.12	-0.38178	-0.34671	-0.26744
17.13	-0.38677	-0.35061	-0.27154
17.14	-0.39172	-0.35457	-0.27567
17.15	-0.39662	-0.35859	-0.27983
17.16	-0.40147	-0.36267	-0.28402
17.17	-0.40628	-0.36678	-0.28824
17.18	-0.41104	-0.37092	-0.2925
17.19	-0.41576	-0.3751	-0.29678
17.2	-0.42043	-0.37929	-0.30111
17.21	-0.42506	-0.38349	-0.30546
17.22	-0.42963	-0.38771	-0.30984
17.23	-0.43416	-0.39193	-0.31426
17.24	-0.43865	-0.39617	-0.3187
17.25	-0.44308	-0.40042	-0.32316
17.26	-0.44747	-0.40467	-0.32764
17.27	-0.45182	-0.40894	-0.33213
17.28	-0.45611	-0.41322	-0.33664
17.29	-0.46036	-0.41751	-0.34116
17.3	-0.46456	-0.42181	-0.34568
17.31	-0.46872	-0.42612	-0.35021
17.32	-0.47283	-0.43043	-0.35475
17.33	-0.47689	-0.43476	-0.35928
17.34	-0.48089	-0.43908	-0.36382
17.35	-0.48485	-0.44342	-0.36836
17.36	-0.48876	-0.44775	-0.37291
17.37	-0.49261	-0.45208	-0.37745
17.38	-0.49641	-0.45641	-0.38199
17.39	-0.50015	-0.46074	-0.38652
17.4	-0.50384	-0.46506	-0.39106
17.41	-0.50747	-0.46937	-0.39559
17.42	-0.51104	-0.47367	-0.40012
17.43	-0.51455	-0.47795	-0.40464
17.44	-0.51801	-0.48221	-0.40915
17.45	-0.5214	-0.48644	-0.41365
17.46	-0.52474	-0.49063	-0.41813

17.47	-0.52801	-0.49479	-0.42261
17.48	-0.53123	-0.49889	-0.42706
17.49	-0.53438	-0.50295	-0.4315
17.5	-0.53747	-0.50695	-0.43591
17.51	-0.54049	-0.51089	-0.4403
17.52	-0.54346	-0.51476	-0.44467
17.53	-0.54635	-0.51858	-0.44901
17.54	-0.54918	-0.52233	-0.45333
17.55	-0.55194	-0.52602	-0.45761
17.56	-0.55464	-0.52966	-0.46186
17.57	-0.55726	-0.53323	-0.46609
17.58	-0.5598	-0.53674	-0.47027
17.59	-0.56227	-0.54019	-0.47443
17.6	-0.56467	-0.54359	-0.47854
17.61	-0.56699	-0.54692	-0.48262
17.62	-0.56923	-0.5502	-0.48666
17.63	-0.57138	-0.5534	-0.49066
17.64	-0.57346	-0.55653	-0.49461
17.65	-0.57545	-0.55958	-0.49853
17.66	-0.57736	-0.56255	-0.5024
17.67	-0.57918	-0.56543	-0.50624
17.68	-0.58091	-0.56822	-0.51003
17.69	-0.58256	-0.57091	-0.51377
17.7	-0.58411	-0.5735	-0.51747
17.71	-0.58557	-0.57598	-0.52112
17.72	-0.58694	-0.57835	-0.52473
17.73	-0.58821	-0.58061	-0.52828
17.74	-0.58939	-0.58276	-0.53178
17.75	-0.59048	-0.58481	-0.53523
17.76	-0.59148	-0.58675	-0.53862
17.77	-0.59238	-0.58858	-0.54194
17.78	-0.59318	-0.59031	-0.54521
17.79	-0.59389	-0.59193	-0.5484
17.8	-0.59451	-0.59344	-0.55153
17.81	-0.59504	-0.59484	-0.55459
17.82	-0.59546	-0.59614	-0.55757
17.83	-0.5958	-0.59733	-0.56049
17.84	-0.59603	-0.59842	-0.56333
17.85	-0.59617	-0.59939	-0.5661
17.86	-0.59621	-0.60026	-0.5688
17.87	-0.59616	-0.60103	-0.57142
17.88	-0.596	-0.60168	-0.57397
17.89	-0.59575	-0.60223	-0.57645
17.9	-0.5954	-0.60268	-0.57885
17.91	-0.59495	-0.60301	-0.58118
17.92	-0.5944	-0.60324	-0.58344
17.93	-0.59374	-0.60337	-0.58561
17.94	-0.59298	-0.60338	-0.58772

17.95	-0.59212	-0.60329	-0.58974
17.96	-0.59115	-0.6031	-0.59169
17.97	-0.59007	-0.60279	-0.59356
17.98	-0.58889	-0.60238	-0.59534
17.99	-0.5876	-0.60187	-0.59705
18	-0.58619	-0.60124	-0.59867
18.01	-0.58467	-0.60051	-0.60022
18.02	-0.58304	-0.59968	-0.60168
18.03	-0.5813	-0.59873	-0.60305
18.04	-0.57944	-0.59768	-0.60433
18.05	-0.57746	-0.59652	-0.60552
18.06	-0.57537	-0.59524	-0.60661
18.07	-0.57316	-0.59386	-0.60761
18.08	-0.57083	-0.59236	-0.60852
18.09	-0.56838	-0.59074	-0.60932
18.1	-0.56581	-0.58902	-0.61002
18.11	-0.56312	-0.58717	-0.61061
18.12	-0.5603	-0.58521	-0.6111
18.13	-0.55737	-0.58312	-0.61149
18.14	-0.55432	-0.5809	-0.61177
18.15	-0.55116	-0.57855	-0.61194
18.16	-0.54788	-0.57605	-0.61202
18.17	-0.54448	-0.57342	-0.61198
18.18	-0.54097	-0.57063	-0.61185
18.19	-0.53735	-0.5677	-0.61161
18.2	-0.53361	-0.5646	-0.61127
18.21	-0.52977	-0.56134	-0.61082
18.22	-0.52581	-0.55792	-0.61026
18.23	-0.52173	-0.55434	-0.60957
18.24	-0.51754	-0.55059	-0.60875
18.25	-0.51321	-0.54668	-0.60778
18.26	-0.50876	-0.5426	-0.60665
18.27	-0.50418	-0.53836	-0.60536
18.28	-0.49946	-0.53395	-0.60389
18.29	-0.4946	-0.52938	-0.60223
18.3	-0.4896	-0.52464	-0.60037
18.31	-0.48446	-0.51973	-0.5983
18.32	-0.47917	-0.51466	-0.59601
18.33	-0.47377	-0.50942	-0.59352
18.34	-0.46824	-0.50402	-0.59082
18.35	-0.46261	-0.49846	-0.58791
18.36	-0.45688	-0.49274	-0.5848
18.37	-0.45106	-0.48686	-0.58147
18.38	-0.44517	-0.48082	-0.57794
18.39	-0.43921	-0.47462	-0.57419
18.4	-0.43319	-0.46827	-0.57025
18.41	-0.42713	-0.46176	-0.56609
18.42	-0.42101	-0.4551	-0.56174

18.43	-0.41486	-0.44829	-0.55718
18.44	-0.40866	-0.44134	-0.55243
18.45	-0.40243	-0.43425	-0.54748
18.46	-0.39615	-0.42702	-0.54235
18.47	-0.38984	-0.41966	-0.53703
18.48	-0.38348	-0.41216	-0.53153
18.49	-0.3771	-0.40455	-0.52585
18.5	-0.37068	-0.39681	-0.51999
18.51	-0.36423	-0.38895	-0.51396
18.52	-0.35774	-0.38098	-0.50777
18.53	-0.35123	-0.37292	-0.50141
18.54	-0.3447	-0.36476	-0.49488
18.55	-0.33814	-0.35652	-0.4882
18.56	-0.33156	-0.34821	-0.48136
18.57	-0.32496	-0.33984	-0.47436
18.58	-0.31834	-0.33142	-0.46722
18.59	-0.3117	-0.32295	-0.45992
18.6	-0.30505	-0.31445	-0.45248
18.61	-0.29839	-0.30593	-0.4449
18.62	-0.29172	-0.29738	-0.43719
18.63	-0.28503	-0.28883	-0.42937
18.64	-0.27833	-0.28026	-0.42146
18.65	-0.27161	-0.27169	-0.41347
18.66	-0.26488	-0.26312	-0.40542
18.67	-0.25814	-0.25456	-0.39732
18.68	-0.25137	-0.24601	-0.38919
18.69	-0.2446	-0.23748	-0.38105
18.7	-0.2378	-0.22897	-0.37291
18.71	-0.23099	-0.22049	-0.36478
18.72	-0.22416	-0.21204	-0.35667
18.73	-0.21734	-0.20364	-0.34858
18.74	-0.21051	-0.1953	-0.34049
18.75	-0.2037	-0.18702	-0.33241
18.76	-0.19691	-0.17882	-0.32435
18.77	-0.19014	-0.1707	-0.31628
18.78	-0.18341	-0.16267	-0.30822
18.79	-0.17671	-0.15474	-0.30016
18.8	-0.17007	-0.14692	-0.2921
18.81	-0.16347	-0.13923	-0.28403
18.82	-0.15694	-0.13165	-0.27597
18.83	-0.15048	-0.1242	-0.26791
18.84	-0.14409	-0.11687	-0.25988
18.85	-0.13778	-0.10968	-0.25187
18.86	-0.13155	-0.10261	-0.24389
18.87	-0.12542	-0.09567	-0.23596
18.88	-0.11938	-0.08887	-0.22807
18.89	-0.11345	-0.0822	-0.22024
18.9	-0.10764	-0.07568	-0.21247

18.91	-0.10193	-0.06928	-0.20477
18.92	-0.09634	-0.06303	-0.19714
18.93	-0.09086	-0.0569	-0.18959
18.94	-0.08548	-0.05089	-0.18211
18.95	-0.0802	-0.045	-0.17471
18.96	-0.07501	-0.03922	-0.1674
18.97	-0.06992	-0.03355	-0.16016
18.98	-0.06491	-0.02798	-0.15301
18.99	-0.05998	-0.0225	-0.14595
19	-0.05513	-0.01712	-0.13897
19.01	-0.05036	-0.01182	-0.13209
19.02	-0.04565	-0.0066	-0.12529
19.03	-0.04101	-0.00147	-0.1186
19.04	-0.03642	0.003558	-0.112
19.05	-0.03188	0.008495	-0.10551
19.06	-0.02738	0.013332	-0.09912
19.07	-0.02292	0.018068	-0.09283
19.08	-0.01849	0.022698	-0.08666
19.09	-0.01409	0.027219	-0.0806
19.1	-0.00971	0.031629	-0.07466
19.11	-0.00534	0.035927	-0.06883
19.12	-0.00098	0.040116	-0.06312
19.13	0.003385	0.044206	-0.05753
19.14	0.007756	0.048203	-0.05204
19.15	0.012142	0.052115	-0.04667
19.16	0.016549	0.055948	-0.0414
19.17	0.020985	0.05971	-0.03624
19.18	0.025458	0.063408	-0.03118
19.19	0.029974	0.06705	-0.02622
19.2	0.03454	0.070642	-0.02135
19.21	0.039162	0.074192	-0.01659
19.22	0.043841	0.077703	-0.01191
19.23	0.048578	0.081181	-0.00733
19.24	0.053372	0.084627	-0.00284
19.25	0.058223	0.088047	0.001552
19.26	0.063132	0.091443	0.005859
19.27	0.068097	0.094821	0.010077
19.28	0.073119	0.098182	0.014207
19.29	0.078197	0.101533	0.018251
19.3	0.083333	0.104875	0.022209
19.31	0.088524	0.108212	0.026083
19.32	0.093769	0.111542	0.029882
19.33	0.099061	0.114862	0.033615
19.34	0.104398	0.118168	0.03729
19.35	0.109775	0.121458	0.040917
19.36	0.115189	0.124728	0.044505
19.37	0.120634	0.127975	0.048063
19.38	0.126107	0.131196	0.0516

19.39	0.131604	0.134387	0.055125
19.4	0.137121	0.137546	0.058648
19.41	0.142654	0.140672	0.062176
19.42	0.148198	0.143767	0.065716
19.43	0.153749	0.146838	0.069272
19.44	0.159304	0.149892	0.072848
19.45	0.164858	0.152932	0.076451
19.46	0.170407	0.155966	0.080085
19.47	0.175947	0.158999	0.083754
19.48	0.181474	0.162037	0.087463
19.49	0.186984	0.165086	0.091218
19.5	0.192473	0.168151	0.095023
19.51	0.197936	0.171237	0.098882
19.52	0.203372	0.174347	0.10279
19.53	0.20878	0.177483	0.106742
19.54	0.214157	0.180646	0.110733
19.55	0.219502	0.183838	0.114756
19.56	0.224814	0.18706	0.118807
19.57	0.23009	0.190316	0.122879
19.58	0.235329	0.193605	0.126968
19.59	0.240529	0.196931	0.131067
19.6	0.245689	0.200294	0.135171
19.61	0.250807	0.203696	0.139277
19.62	0.255882	0.207139	0.143388
19.63	0.260915	0.210621	0.147512
19.64	0.265905	0.214145	0.151654
19.65	0.270851	0.217711	0.155821
19.66	0.275754	0.221318	0.160019
19.67	0.280612	0.224968	0.164254
19.68	0.285425	0.228661	0.168533
19.69	0.290194	0.232397	0.172862
19.7	0.294917	0.236177	0.177247
19.71	0.299595	0.240002	0.181692
19.72	0.304226	0.243871	0.186192
19.73	0.308811	0.247782	0.190737
19.74	0.313349	0.251736	0.19532
19.75	0.31784	0.255731	0.199931
19.76	0.322283	0.259767	0.204564
19.77	0.326678	0.263843	0.209208
19.78	0.331024	0.267958	0.213857
19.79	0.335321	0.272111	0.2185
19.8	0.339569	0.276301	0.223131
19.81	0.343767	0.280528	0.227742
19.82	0.347915	0.284788	0.232332
19.83	0.352011	0.289078	0.2369
19.84	0.356056	0.293393	0.241448
19.85	0.360048	0.297731	0.245973
19.86	0.363987	0.302087	0.250478

19.87	0.367873	0.306459	0.254961
19.88	0.371705	0.310842	0.259423
19.89	0.375481	0.315233	0.263864
19.9	0.379202	0.319629	0.268283
19.91	0.382867	0.324025	0.272681
19.92	0.386475	0.328419	0.277057
19.93	0.390024	0.332808	0.28141
19.94	0.393513	0.337188	0.285739
19.95	0.396941	0.341557	0.290044
19.96	0.400306	0.345911	0.294323
19.97	0.403608	0.350248	0.298576
19.98	0.406845	0.354564	0.302802
19.99	0.410016	0.358855	0.307001
20	0.41312	0.363121	0.311171
20.01	0.416155	0.367356	0.315311
20.02	0.41912	0.371557	0.31942
20.03	0.422015	0.375721	0.323498
20.04	0.424837	0.379844	0.327541
20.05	0.427586	0.383923	0.331549
20.06	0.430261	0.387954	0.335521
20.07	0.432861	0.391932	0.339454
20.08	0.435384	0.395856	0.343348
20.09	0.437829	0.39972	0.347201
20.1	0.440195	0.403521	0.351012
20.11	0.442482	0.407257	0.354779
20.12	0.444687	0.410924	0.358505
20.13	0.446809	0.41452	0.362191
20.14	0.448849	0.418043	0.365841
20.15	0.450803	0.42149	0.369455
20.16	0.452672	0.424861	0.373038
20.17	0.454453	0.428151	0.37659
20.18	0.456146	0.43136	0.380114
20.19	0.457749	0.434484	0.383613
20.2	0.459262	0.437523	0.387089
20.21	0.460683	0.440472	0.390543
20.22	0.462012	0.443333	0.393974
20.23	0.463248	0.446104	0.39738
20.24	0.46439	0.448785	0.400758
20.25	0.46544	0.451376	0.404108
20.26	0.466396	0.453874	0.407427
20.27	0.467257	0.456282	0.410713
20.28	0.468024	0.458596	0.413963
20.29	0.468696	0.460818	0.417176
20.3	0.469272	0.462947	0.42035
20.31	0.469753	0.464981	0.423483
20.32	0.470139	0.46692	0.426572
20.33	0.47043	0.468764	0.429616
20.34	0.470628	0.47051	0.432612

20.35	0.470733	0.472158	0.435558
20.36	0.470745	0.473706	0.438453
20.37	0.470666	0.475155	0.441293
20.38	0.470495	0.476502	0.444076
20.39	0.470234	0.477746	0.446801
20.4	0.469884	0.478887	0.449466
20.41	0.469445	0.479923	0.452068
20.42	0.468919	0.480855	0.454605
20.43	0.468309	0.481681	0.457077
20.44	0.467617	0.482402	0.459482
20.45	0.466847	0.483016	0.461817
20.46	0.465999	0.483523	0.464082
20.47	0.465078	0.483924	0.466276
20.48	0.464084	0.484217	0.468396
20.49	0.463022	0.484403	0.470441
20.5	0.461892	0.48448	0.47241
20.51	0.460699	0.484449	0.474301
20.52	0.459452	0.484312	0.476112
20.53	0.45816	0.484072	0.477842
20.54	0.456833	0.48373	0.479488
20.55	0.455481	0.483289	0.481051
20.56	0.454114	0.482753	0.482526
20.57	0.452741	0.482122	0.483913
20.58	0.451372	0.4814	0.485211
20.59	0.450018	0.48059	0.486416
20.6	0.448688	0.479693	0.487529
20.61	0.44739	0.478713	0.488546
20.62	0.446125	0.477652	0.489471
20.63	0.444893	0.476515	0.490304
20.64	0.443693	0.475306	0.491049
20.65	0.442525	0.474028	0.491706
20.66	0.441388	0.472685	0.492277
20.67	0.440282	0.471281	0.492766
20.68	0.439206	0.469819	0.493173
20.69	0.438159	0.468303	0.493501
20.7	0.437142	0.466738	0.493751
20.71	0.436153	0.465125	0.493926
20.72	0.435193	0.463465	0.494025
20.73	0.434262	0.461754	0.494048
20.74	0.433361	0.459992	0.493996
20.75	0.43249	0.458175	0.493867
20.76	0.431649	0.456303	0.493663
20.77	0.430839	0.454373	0.493382
20.78	0.430061	0.452384	0.493024
20.79	0.429314	0.450333	0.49259
20.8	0.428599	0.448218	0.492079
20.81	0.427917	0.446042	0.491493
20.82	0.427266	0.443817	0.49084

20.83	0.426644	0.44156	0.490132
20.84	0.426051	0.43929	0.489379
20.85	0.425484	0.437022	0.488591
20.86	0.424944	0.434774	0.487779
20.87	0.424427	0.432562	0.486954
20.88	0.423934	0.430405	0.486126
20.89	0.423462	0.428318	0.485306
20.9	0.423011	0.426319	0.484505
20.91	0.422578	0.424425	0.483732
20.92	0.422162	0.422653	0.483
20.93	0.421763	0.42102	0.482317
20.94	0.421378	0.419543	0.481696
20.95	0.421007	0.418238	0.481146
20.96	0.420648	0.417124	0.480678
20.97	0.4203	0.416217	0.480303
20.98	0.41996	0.415533	0.480032
20.99	0.419629	0.415091	0.479874
21	0.419304	0.414907	0.479841

Table S12. Test Result Data of the Sinusoidal Steering Test at an Adhesion Coefficient of 0.8

Time	carsim	DRL-UKF	UKF
0	#######	0.01253	0.021621
0.01	0.002556	0.012705	0.021826
0.02	0.004779	0.012798	0.021933
0.03	0.006679	0.012811	0.021949
0.04	0.008271	0.012749	0.021877
0.05	0.009567	0.012617	0.021722
0.06	0.010579	0.012418	0.02149
0.07	0.011321	0.012156	0.021184
0.08	0.011806	0.011837	0.020811
0.09	0.012045	0.011463	0.020374
0.1	0.012052	0.011039	0.019879
0.11	0.01184	0.010569	0.019331
0.12	0.011421	0.010058	0.018734
0.13	0.010808	0.009509	0.018093
0.14	0.010014	0.008927	0.017413
0.15	0.009052	0.008316	0.016699
0.16	0.007934	0.007679	0.015956
0.17	0.006674	0.007022	0.015188
0.18	0.005283	0.006348	0.014401
0.19	0.003775	0.005662	0.013599
0.2	0.002163	0.004967	0.012787

0.21	0.000459	0.004266	0.011969
0.22	-0.00132	0.003554	0.011136
0.23	-0.00316	0.002822	0.010282
0.24	-0.00503	0.002065	0.009397
0.25	-0.00694	0.001274	0.008473
0.26	-0.00885	0.000442	0.007501
0.27	-0.01075	-0.00044	0.006474
0.28	-0.01263	-0.00137	0.005381
0.29	-0.01446	-0.00237	0.004216
0.3	-0.01624	-0.00344	0.002969
0.31	-0.01794	-0.00458	0.001635
0.32	-0.01956	-0.00579	0.000228
0.33	-0.0211	-0.00704	-0.00124
0.34	-0.02255	-0.00833	-0.00274
0.35	-0.0239	-0.00964	-0.00427
0.36	-0.02515	-0.01096	-0.00581
0.37	-0.02629	-0.01226	-0.00734
0.38	-0.02733	-0.01355	-0.00884
0.39	-0.02825	-0.0148	-0.0103
0.4	-0.02904	-0.016	-0.0117
0.41	-0.02972	-0.01714	-0.01304
0.42	-0.03027	-0.01822	-0.01429
0.43	-0.03072	-0.01923	-0.01548
0.44	-0.03106	-0.02018	-0.01659
0.45	-0.03131	-0.02107	-0.01763
0.46	-0.03146	-0.02189	-0.0186
0.47	-0.03154	-0.02266	-0.0195
0.48	-0.03154	-0.02337	-0.02032
0.49	-0.03148	-0.02403	-0.02108
0.5	-0.03135	-0.02462	-0.02177
0.51	-0.03118	-0.02516	-0.02239
0.52	-0.03095	-0.02563	-0.02293
0.53	-0.03068	-0.02603	-0.02339
0.54	-0.03036	-0.02634	-0.02375
0.55	-0.02999	-0.02657	-0.02401
0.56	-0.02959	-0.0267	-0.02416
0.57	-0.02915	-0.02672	-0.02419
0.58	-0.02867	-0.02663	-0.02408
0.59	-0.02816	-0.02641	-0.02384
0.6	-0.02761	-0.02605	-0.02345
0.61	-0.02704	-0.02557	-0.02291
0.62	-0.02644	-0.02499	-0.02226
0.63	-0.02582	-0.02435	-0.02155
0.64	-0.02518	-0.02371	-0.02082
0.65	-0.02452	-0.0231	-0.02013
0.66	-0.02386	-0.02256	-0.01951
0.67	-0.02319	-0.02214	-0.01902
0.68	-0.02252	-0.02189	-0.0187

0.69	-0.02185	-0.02184	-0.0186
0.7	-0.0212	-0.02204	-0.01876
0.71	-0.02055	-0.02252	-0.01922
0.72	-0.01991	-0.02326	-0.01996
0.73	-0.01928	-0.02421	-0.02093
0.74	-0.01867	-0.02534	-0.0221
0.75	-0.01806	-0.02661	-0.02343
0.76	-0.01747	-0.02798	-0.02488
0.77	-0.01688	-0.0294	-0.02642
0.78	-0.0163	-0.03085	-0.028
0.79	-0.01574	-0.03228	-0.02959
0.8	-0.01518	-0.03366	-0.03114
0.81	-0.01463	-0.03494	-0.03263
0.82	-0.01409	-0.03614	-0.03406
0.83	-0.01356	-0.03725	-0.03541
0.84	-0.01304	-0.03829	-0.03669
0.85	-0.01254	-0.03926	-0.03791
0.86	-0.01205	-0.04016	-0.03906
0.87	-0.01158	-0.041	-0.04014
0.88	-0.01113	-0.04179	-0.04115
0.89	-0.01069	-0.04253	-0.04209
0.9	-0.01028	-0.04323	-0.04297
0.91	-0.00988	-0.0439	-0.04378
0.92	-0.00951	-0.04454	-0.04453
0.93	-0.00915	-0.04516	-0.04524
0.94	-0.00881	-0.04577	-0.04589
0.95	-0.00849	-0.04637	-0.04652
0.96	-0.00817	-0.04698	-0.04711
0.97	-0.00787	-0.04759	-0.04769
0.98	-0.00758	-0.04822	-0.04826
0.99	-0.0073	-0.04888	-0.04882
1	-0.00703	-0.04957	-0.04938
1.01	-0.00676	-0.05029	-0.04995
1.02	-0.0065	-0.05102	-0.0505
1.03	-0.00624	-0.05171	-0.05098
1.04	-0.00599	-0.05233	-0.05135
1.05	-0.00574	-0.05285	-0.05159
1.06	-0.0055	-0.05323	-0.05164
1.07	-0.00526	-0.05344	-0.05147
1.08	-0.00503	-0.05343	-0.05105
1.09	-0.0048	-0.05317	-0.05033
1.1	-0.00457	-0.05263	-0.04927
1.11	-0.00435	-0.05179	-0.04786
1.12	-0.00413	-0.05066	-0.04611
1.13	-0.00392	-0.0493	-0.04408
1.14	-0.0037	-0.04775	-0.04181
1.15	-0.00349	-0.04605	-0.03934
1.16	-0.00328	-0.04425	-0.03672

1.17	-0.00307	-0.04238	-0.034
1.18	-0.00285	-0.04049	-0.03121
1.19	-0.00264	-0.03862	-0.02841
1.2	-0.00242	-0.03682	-0.02563
1.21	-0.0022	-0.03512	-0.02292
1.22	-0.00198	-0.03351	-0.02028
1.23	-0.00177	-0.032	-0.01771
1.24	-0.00155	-0.03058	-0.01521
1.25	-0.00134	-0.02923	-0.01278
1.26	-0.00114	-0.02796	-0.01043
1.27	-0.00095	-0.02675	-0.00815
1.28	-0.00077	-0.0256	-0.00595
1.29	-0.00061	-0.0245	-0.00381
1.3	-0.00046	-0.02344	-0.00176
1.31	-0.00033	-0.02242	0.000226
1.32	-0.00021	-0.02143	0.002131
1.33	#######	-0.02049	0.003958
1.34	2.17E-05	-0.01959	0.005703
1.35	0.000151	-0.01873	0.007363
1.36	0.000299	-0.01792	0.008938
1.37	0.000471	-0.01715	0.010423
1.38	0.000677	-0.01644	0.011818
1.39	0.000923	-0.01578	0.013119
1.4	0.001216	-0.01517	0.014323
1.41	0.001559	-0.01462	0.01543
1.42	0.001931	-0.01412	0.016438
1.43	0.002307	-0.01367	0.017345
1.44	0.002661	-0.01329	0.018152
1.45	0.002969	-0.01295	0.018858
1.46	0.003204	-0.01267	0.019461
1.47	0.003342	-0.01245	0.019961
1.48	0.003357	-0.01228	0.020358
1.49	0.003224	-0.01216	0.02065
1.5	0.002917	-0.0121	0.020837
1.51	0.002413	-0.01209	0.020916
1.52	0.001701	-0.01214	0.020882
1.53	0.000773	-0.01226	0.020727
1.54	-0.00038	-0.01245	0.020444
1.55	-0.00177	-0.01273	0.020025
1.56	-0.00341	-0.01308	0.019463
1.57	-0.00529	-0.01354	0.018749
1.58	-0.00744	-0.01409	0.017878
1.59	-0.00986	-0.01475	0.016841
1.6	-0.01256	-0.01552	0.015632
1.61	-0.01554	-0.01641	0.014244
1.62	-0.0188	-0.01742	0.012686
1.63	-0.02234	-0.01855	0.010968
1.64	-0.02613	-0.0198	0.009099

1.65	-0.03017	-0.02117	0.00709
1.66	-0.03445	-0.02265	0.00495
1.67	-0.03897	-0.02425	0.00269
1.68	-0.0437	-0.02596	0.000319
1.69	-0.04865	-0.02779	-0.00215
1.7	-0.0538	-0.02973	-0.00472
1.71	-0.05915	-0.03179	-0.00736
1.72	-0.06467	-0.03397	-0.0101
1.73	-0.07037	-0.03627	-0.01293
1.74	-0.07622	-0.0387	-0.01587
1.75	-0.08223	-0.04126	-0.01892
1.76	-0.08836	-0.04397	-0.0221
1.77	-0.09462	-0.04682	-0.02542
1.78	-0.101	-0.04982	-0.02887
1.79	-0.10747	-0.05298	-0.03247
1.8	-0.11404	-0.0563	-0.03623
1.81	-0.12068	-0.05978	-0.04016
1.82	-0.12739	-0.06343	-0.04425
1.83	-0.13415	-0.06725	-0.0485
1.84	-0.14094	-0.07123	-0.0529
1.85	-0.14776	-0.07537	-0.05745
1.86	-0.15458	-0.07967	-0.06215
1.87	-0.1614	-0.08413	-0.06698
1.88	-0.1682	-0.08875	-0.07195
1.89	-0.17496	-0.09353	-0.07706
1.9	-0.18168	-0.09846	-0.08228
1.91	-0.18833	-0.10355	-0.08763
1.92	-0.19492	-0.10879	-0.09309
1.93	-0.20144	-0.11416	-0.09866
1.94	-0.20789	-0.11965	-0.10431
1.95	-0.21426	-0.12527	-0.11005
1.96	-0.22055	-0.13099	-0.11587
1.97	-0.22677	-0.13682	-0.12175
1.98	-0.23289	-0.14273	-0.12769
1.99	-0.23893	-0.14872	-0.13367
2	-0.24488	-0.15478	-0.13969
2.01	-0.25074	-0.1609	-0.14574
2.02	-0.2565	-0.16708	-0.15182
2.03	-0.26217	-0.17332	-0.15794
2.04	-0.26775	-0.1796	-0.16411
2.05	-0.27323	-0.18593	-0.17032
2.06	-0.27863	-0.1923	-0.17659
2.07	-0.28394	-0.19871	-0.18292
2.08	-0.28916	-0.20516	-0.18932
2.09	-0.29429	-0.21164	-0.19579
2.1	-0.29934	-0.21815	-0.20233
2.11	-0.3043	-0.22468	-0.20895
2.12	-0.30918	-0.23124	-0.21565

2.13	-0.31397	-0.2378	-0.2224
2.14	-0.31869	-0.24436	-0.22921
2.15	-0.32334	-0.25093	-0.23605
2.16	-0.32791	-0.25748	-0.24293
2.17	-0.33241	-0.26401	-0.24982
2.18	-0.33685	-0.27052	-0.25672
2.19	-0.34122	-0.277	-0.26362
2.2	-0.34554	-0.28344	-0.27051
2.21	-0.34979	-0.28983	-0.27737
2.22	-0.35399	-0.29617	-0.28421
2.23	-0.35813	-0.30244	-0.29101
2.24	-0.36222	-0.30866	-0.29777
2.25	-0.36625	-0.3148	-0.30449
2.26	-0.37022	-0.32086	-0.31117
2.27	-0.37414	-0.32684	-0.31779
2.28	-0.37801	-0.33272	-0.32435
2.29	-0.38183	-0.33851	-0.33084
2.3	-0.38559	-0.3442	-0.33727
2.31	-0.3893	-0.34978	-0.34363
2.32	-0.39297	-0.35524	-0.3499
2.33	-0.39658	-0.36059	-0.35608
2.34	-0.40013	-0.36582	-0.36216
2.35	-0.40364	-0.37094	-0.36813
2.36	-0.40709	-0.37593	-0.37399
2.37	-0.41048	-0.3808	-0.37971
2.38	-0.41383	-0.38554	-0.38529
2.39	-0.41711	-0.39016	-0.39073
2.4	-0.42034	-0.39464	-0.39601
2.41	-0.42352	-0.399	-0.40113
2.42	-0.42664	-0.40322	-0.40609
2.43	-0.4297	-0.40731	-0.41087
2.44	-0.4327	-0.41128	-0.41549
2.45	-0.43565	-0.41512	-0.41993
2.46	-0.43854	-0.41884	-0.42421
2.47	-0.44136	-0.42244	-0.42832
2.48	-0.44413	-0.42592	-0.43225
2.49	-0.44683	-0.42928	-0.436
2.5	-0.44948	-0.43252	-0.43959
2.51	-0.45206	-0.43565	-0.44299
2.52	-0.45457	-0.43867	-0.44622
2.53	-0.45702	-0.44157	-0.44928
2.54	-0.4594	-0.44435	-0.45216
2.55	-0.46171	-0.44701	-0.45486
2.56	-0.46394	-0.44956	-0.4574
2.57	-0.4661	-0.45198	-0.45976
2.58	-0.46818	-0.45429	-0.46194
2.59	-0.47018	-0.45647	-0.46396
2.6	-0.4721	-0.45854	-0.4658

2.61	-0.47393	-0.46047	-0.46747
2.62	-0.47568	-0.4623	-0.46898
2.63	-0.47735	-0.464	-0.47034
2.64	-0.47894	-0.4656	-0.47154
2.65	-0.48044	-0.46709	-0.47259
2.66	-0.48187	-0.46849	-0.47351
2.67	-0.48323	-0.46979	-0.47429
2.68	-0.48451	-0.47099	-0.47496
2.69	-0.48572	-0.47212	-0.4755
2.7	-0.48685	-0.47316	-0.47593
2.71	-0.48792	-0.47412	-0.47625
2.72	-0.48891	-0.47501	-0.47647
2.73	-0.48983	-0.47583	-0.4766
2.74	-0.49068	-0.47657	-0.47663
2.75	-0.49144	-0.47725	-0.47658
2.76	-0.49212	-0.47785	-0.47645
2.77	-0.49272	-0.47839	-0.47623
2.78	-0.49323	-0.47886	-0.47595
2.79	-0.49366	-0.47927	-0.47559
2.8	-0.49399	-0.47962	-0.47517
2.81	-0.49423	-0.4799	-0.47469
2.82	-0.49438	-0.48012	-0.47415
2.83	-0.49444	-0.48028	-0.47356
2.84	-0.49441	-0.48037	-0.47291
2.85	-0.49429	-0.4804	-0.47221
2.86	-0.49409	-0.48037	-0.47147
2.87	-0.4938	-0.48028	-0.47068
2.88	-0.49342	-0.48012	-0.46984
2.89	-0.49296	-0.47989	-0.46897
2.9	-0.49241	-0.47961	-0.46805
2.91	-0.49179	-0.47925	-0.4671
2.92	-0.49108	-0.47884	-0.46611
2.93	-0.49027	-0.47836	-0.46507
2.94	-0.48937	-0.47782	-0.46398
2.95	-0.48836	-0.47723	-0.46284
2.96	-0.48724	-0.47658	-0.46164
2.97	-0.48601	-0.47589	-0.46038
2.98	-0.48466	-0.47514	-0.45905
2.99	-0.48319	-0.47435	-0.45765
3	-0.48158	-0.47351	-0.45618
3.01	-0.47984	-0.47263	-0.45462
3.02	-0.47796	-0.4717	-0.45299
3.03	-0.47597	-0.47073	-0.45126
3.04	-0.47385	-0.4697	-0.44945
3.05	-0.47162	-0.46862	-0.44754
3.06	-0.46929	-0.46748	-0.44554
3.07	-0.46685	-0.46627	-0.44344
3.08	-0.46432	-0.465	-0.44124

3.09	-0.4617	-0.46367	-0.43893
3.1	-0.459	-0.46226	-0.43652
3.11	-0.45622	-0.46077	-0.43399
3.12	-0.45336	-0.45921	-0.43135
3.13	-0.45041	-0.45757	-0.42859
3.14	-0.44737	-0.45586	-0.42571
3.15	-0.44424	-0.45406	-0.42272
3.16	-0.441	-0.45219	-0.41959
3.17	-0.43766	-0.45023	-0.41634
3.18	-0.43421	-0.44819	-0.41297
3.19	-0.43064	-0.44607	-0.40946
3.2	-0.42696	-0.44387	-0.40581
3.21	-0.42315	-0.44158	-0.40203
3.22	-0.41922	-0.4392	-0.39811
3.23	-0.41518	-0.43673	-0.39405
3.24	-0.41103	-0.43418	-0.38985
3.25	-0.40679	-0.43152	-0.38551
3.26	-0.40245	-0.42877	-0.38101
3.27	-0.39804	-0.42592	-0.37637
3.28	-0.39354	-0.42297	-0.37158
3.29	-0.38898	-0.41992	-0.36664
3.3	-0.38435	-0.41676	-0.36155
3.31	-0.37966	-0.41349	-0.3563
3.32	-0.37492	-0.41011	-0.35089
3.33	-0.37012	-0.40662	-0.34534
3.34	-0.36527	-0.40301	-0.33965
3.35	-0.36036	-0.3993	-0.33381
3.36	-0.3554	-0.39546	-0.32784
3.37	-0.35039	-0.39151	-0.32173
3.38	-0.34532	-0.38744	-0.31549
3.39	-0.3402	-0.38325	-0.30912
3.4	-0.33503	-0.37894	-0.30263
3.41	-0.32981	-0.3745	-0.29602
3.42	-0.32453	-0.36995	-0.28929
3.43	-0.31921	-0.36527	-0.28244
3.44	-0.31384	-0.36048	-0.27547
3.45	-0.30842	-0.35557	-0.26838
3.46	-0.30295	-0.35056	-0.26117
3.47	-0.29744	-0.34543	-0.25383
3.48	-0.29188	-0.34019	-0.24638
3.49	-0.28629	-0.33485	-0.2388
3.5	-0.28064	-0.32941	-0.23111
3.51	-0.27496	-0.32386	-0.22329
3.52	-0.26924	-0.31822	-0.21536
3.53	-0.26348	-0.31247	-0.20732
3.54	-0.25768	-0.30662	-0.19919
3.55	-0.25183	-0.30066	-0.19096
3.56	-0.24595	-0.2946	-0.18264

3.57	-0.24004	-0.28844	-0.17425
3.58	-0.23408	-0.28217	-0.16578
3.59	-0.22809	-0.27579	-0.15726
3.6	-0.22206	-0.26931	-0.14867
3.61	-0.21599	-0.26273	-0.14004
3.62	-0.20989	-0.25604	-0.13136
3.63	-0.20377	-0.24925	-0.12266
3.64	-0.19761	-0.24236	-0.11395
3.65	-0.19144	-0.23539	-0.10522
3.66	-0.18524	-0.22832	-0.09651
3.67	-0.17903	-0.22117	-0.08781
3.68	-0.17281	-0.21394	-0.07914
3.69	-0.16658	-0.20664	-0.07052
3.7	-0.16035	-0.19926	-0.06194
3.71	-0.15411	-0.19181	-0.05342
3.72	-0.14788	-0.18431	-0.04497
3.73	-0.14166	-0.17674	-0.03659
3.74	-0.13547	-0.16914	-0.02829
3.75	-0.12931	-0.16149	-0.02007
3.76	-0.12319	-0.15382	-0.01193
3.77	-0.11712	-0.14612	-0.00388
3.78	-0.11111	-0.1384	0.004076
3.79	-0.10516	-0.13068	0.011934
3.8	-0.09928	-0.12296	0.01969
3.81	-0.09348	-0.11525	0.02734
3.82	-0.08777	-0.10755	0.034881
3.83	-0.08213	-0.09985	0.042313
3.84	-0.07658	-0.09215	0.049633
3.85	-0.07111	-0.08446	0.056841
3.86	-0.06572	-0.07677	0.063935
3.87	-0.06042	-0.06907	0.070914
3.88	-0.0552	-0.06138	0.077776
3.89	-0.05006	-0.05369	0.084519
3.9	-0.04501	-0.04599	0.091143
3.91	-0.04004	-0.03828	0.097645
3.92	-0.03514	-0.03058	0.104019
3.93	-0.03032	-0.02289	0.110259
3.94	-0.02557	-0.0152	0.116359
3.95	-0.02088	-0.00754	0.122312
3.96	-0.01625	9.36E-05	0.128111
3.97	-0.01166	0.007696	0.133751
3.98	-0.00713	0.01526	0.139225
3.99	-0.00263	0.022778	0.144526
4	0.001834	0.030245	0.149648
4.01	0.006268	0.037653	0.154588
4.02	0.010679	0.044996	0.159352
4.03	0.015069	0.052267	0.163952
4.04	0.019445	0.05946	0.168398

4.05	0.023811	0.066567	0.172701
4.06	0.028172	0.073581	0.17687
4.07	0.032532	0.080497	0.180917
4.08	0.036896	0.087305	0.184851
4.09	0.041269	0.094001	0.188684
4.1	0.045656	0.100577	0.192425
4.11	0.05006	0.107027	0.196084
4.12	0.054488	0.113354	0.199669
4.13	0.058943	0.119559	0.203185
4.14	0.063431	0.125647	0.206638
4.15	0.067955	0.131618	0.210035
4.16	0.072521	0.137478	0.213381
4.17	0.077134	0.143228	0.216682
4.18	0.081798	0.148871	0.219944
4.19	0.086518	0.15441	0.223174
4.2	0.091299	0.159848	0.226377
4.21	0.096143	0.165187	0.229558
4.22	0.10105	0.170424	0.232718
4.23	0.106017	0.175555	0.235854
4.24	0.11104	0.180575	0.238966
4.25	0.116117	0.18548	0.242052
4.26	0.121246	0.190267	0.245111
4.27	0.126422	0.19493	0.248142
4.28	0.131644	0.199467	0.251144
4.29	0.136908	0.203873	0.254114
4.3	0.142211	0.208143	0.257053
4.31	0.147552	0.212275	0.259959
4.32	0.152924	0.216269	0.262836
4.33	0.158325	0.220128	0.26569
4.34	0.163751	0.223854	0.268526
4.35	0.169196	0.227449	0.271348
4.36	0.174658	0.230915	0.274162
4.37	0.180131	0.234254	0.276972
4.38	0.185612	0.237469	0.279784
4.39	0.191097	0.24056	0.282602
4.4	0.196581	0.243531	0.285432
4.41	0.202061	0.246385	0.288277
4.42	0.207533	0.249135	0.291141
4.43	0.212993	0.251793	0.294024
4.44	0.218437	0.254373	0.296928
4.45	0.223864	0.256889	0.299855
4.46	0.229268	0.259356	0.302807
4.47	0.234647	0.261785	0.305784
4.48	0.239997	0.264191	0.308788
4.49	0.245315	0.266588	0.311822
4.5	0.250596	0.26899	0.314886
4.51	0.255839	0.271406	0.317982
4.52	0.261041	0.273842	0.321105

4.53	0.266202	0.276295	0.324254
4.54	0.271319	0.278767	0.327423
4.55	0.276392	0.281258	0.33061
4.56	0.281418	0.283768	0.333811
4.57	0.286398	0.286297	0.337021
4.58	0.291329	0.288846	0.340237
4.59	0.296211	0.291414	0.343456
4.6	0.301042	0.294003	0.346673
4.61	0.30582	0.296612	0.349886
4.62	0.310546	0.299242	0.353095
4.63	0.315219	0.301895	0.3563
4.64	0.319838	0.304571	0.359502
4.65	0.324404	0.307271	0.362702
4.66	0.328916	0.309997	0.3659
4.67	0.333374	0.31275	0.369097
4.68	0.337777	0.31553	0.372295
4.69	0.342125	0.318339	0.375492
4.7	0.346418	0.321178	0.378691
4.71	0.350655	0.324047	0.381893
4.72	0.354837	0.326949	0.3851
4.73	0.358964	0.329885	0.388317
4.74	0.363036	0.332856	0.391548
4.75	0.367053	0.335865	0.394796
4.76	0.371015	0.338912	0.398065
4.77	0.374923	0.341999	0.40136
4.78	0.378777	0.345129	0.404684
4.79	0.382578	0.348301	0.40804
4.8	0.386324	0.351519	0.411434
4.81	0.390017	0.354782	0.414867
4.82	0.393655	0.35808	0.418333
4.83	0.397239	0.361405	0.421825
4.84	0.400767	0.364745	0.425336
4.85	0.404239	0.36809	0.428858
4.86	0.407654	0.371431	0.432383
4.87	0.411011	0.374756	0.435905
4.88	0.41431	0.378055	0.439415
4.89	0.41755	0.381318	0.442907
4.9	0.42073	0.384535	0.446373
4.91	0.423849	0.387697	0.449806
4.92	0.426908	0.390803	0.453196
4.93	0.429905	0.393852	0.456537
4.94	0.432839	0.396844	0.45982
4.95	0.435709	0.39978	0.463037
4.96	0.438515	0.40266	0.466179
4.97	0.441257	0.405484	0.469238
4.98	0.443932	0.408252	0.472206
4.99	0.446542	0.410963	0.475076
5	0.449083	0.413619	0.477838

5.01	0.451557	0.41622	0.480486
5.02	0.453962	0.418766	0.483015
5.03	0.456298	0.421259	0.485424
5.04	0.458563	0.423702	0.487709
5.05	0.460757	0.426096	0.489868
5.06	0.462878	0.428443	0.491899
5.07	0.464927	0.430743	0.493798
5.08	0.466902	0.433	0.495563
5.09	0.468803	0.435214	0.497192
5.1	0.470628	0.437387	0.498681
5.11	0.472377	0.43952	0.500029
5.12	0.474049	0.441611	0.501231
5.13	0.475644	0.443657	0.502284
5.14	0.477162	0.445655	0.503184
5.15	0.4786	0.447601	0.503928
5.16	0.47996	0.449494	0.504513
5.17	0.48124	0.451329	0.504934
5.18	0.48244	0.453103	0.505188
5.19	0.483559	0.454815	0.505272
5.2	0.484597	0.45646	0.505181
5.21	0.485552	0.458036	0.504914
5.22	0.486426	0.459546	0.504468
5.23	0.487216	0.460991	0.503844
5.24	0.487922	0.462373	0.50304
5.25	0.488543	0.463696	0.502057
5.26	0.48908	0.464961	0.500893
5.27	0.489531	0.466171	0.499548
5.28	0.489895	0.467327	0.498022
5.29	0.490173	0.468433	0.496314
5.3	0.490363	0.469491	0.494423
5.31	0.490465	0.470503	0.492351
5.32	0.490483	0.471465	0.490109
5.33	0.49042	0.472377	0.487707
5.34	0.490281	0.473236	0.48516
5.35	0.490069	0.474039	0.482478
5.36	0.489787	0.474783	0.479675
5.37	0.48944	0.475466	0.476762
5.38	0.489031	0.476086	0.473751
5.39	0.488565	0.47664	0.470656
5.4	0.488045	0.477125	0.467487
5.41	0.487472	0.477539	0.464256
5.42	0.486841	0.477881	0.460967
5.43	0.486141	0.478147	0.457622
5.44	0.485366	0.478336	0.454222
5.45	0.484504	0.478446	0.450771
5.46	0.483549	0.478474	0.447271
5.47	0.48249	0.478419	0.443723
5.48	0.481319	0.478279	0.44013

5.49	0.480027	0.478051	0.436495
5.5	0.478606	0.477733	0.432819
5.51	0.477048	0.477324	0.429104
5.52	0.475356	0.476822	0.425349
5.53	0.473535	0.476226	0.421555
5.54	0.471588	0.475536	0.417719
5.55	0.46952	0.47475	0.41384
5.56	0.467335	0.473867	0.409919
5.57	0.465038	0.472887	0.405953
5.58	0.462633	0.471808	0.401942
5.59	0.460124	0.47063	0.397884
5.6	0.457516	0.469352	0.393779
5.61	0.454812	0.467971	0.389627
5.62	0.45201	0.466485	0.385427
5.63	0.449106	0.46489	0.381183
5.64	0.446097	0.463181	0.376895
5.65	0.442979	0.461355	0.372566
5.66	0.43975	0.459408	0.368196
5.67	0.436405	0.457335	0.363788
5.68	0.432941	0.455133	0.359342
5.69	0.429356	0.452797	0.354862
5.7	0.425645	0.450325	0.350348
5.71	0.421806	0.447712	0.3458
5.72	0.417842	0.44496	0.341213
5.73	0.413757	0.442071	0.336578
5.74	0.409554	0.439047	0.331887
5.75	0.405238	0.43589	0.327133
5.76	0.400812	0.432602	0.322309
5.77	0.396281	0.429186	0.317406
5.78	0.391647	0.425643	0.312416
5.79	0.386915	0.421976	0.307332
5.8	0.382088	0.418187	0.302146
5.81	0.377171	0.414277	0.296851
5.82	0.372169	0.410247	0.291446
5.83	0.367087	0.406098	0.28593
5.84	0.36193	0.401829	0.280304
5.85	0.356704	0.397441	0.274565
5.86	0.351413	0.392934	0.268715
5.87	0.346064	0.388308	0.262753
5.88	0.340662	0.383564	0.256678
5.89	0.335211	0.378702	0.250489
5.9	0.329718	0.373723	0.244188
5.91	0.324186	0.368626	0.237772
5.92	0.318616	0.363414	0.231244
5.93	0.313006	0.358089	0.224606
5.94	0.307356	0.352654	0.217859
5.95	0.301664	0.347112	0.211005
5.96	0.295931	0.341464	0.204045

5.97	0.290154	0.335714	0.196981
5.98	0.284333	0.329863	0.189816
5.99	0.278467	0.323914	0.18255
6	0.272556	0.317869	0.175185
6.01	0.266597	0.311732	0.167724
6.02	0.260595	0.305505	0.160167
6.03	0.25455	0.29919	0.152518
6.04	0.248465	0.292792	0.144778
6.05	0.242343	0.286312	0.13695
6.06	0.236186	0.279753	0.129035
6.07	0.229996	0.273119	0.121036
6.08	0.223775	0.266413	0.112954
6.09	0.217527	0.259637	0.104793
6.1	0.211254	0.252795	0.096553
6.11	0.204957	0.245888	0.088238
6.12	0.198639	0.238917	0.079853
6.13	0.192304	0.231881	0.071404
6.14	0.185954	0.224779	0.062896
6.15	0.17959	0.21761	0.054336
6.16	0.173217	0.210375	0.045728
6.17	0.166837	0.203072	0.037079
6.18	0.160452	0.1957	0.028394
6.19	0.154065	0.188259	0.01968
6.2	0.147679	0.180749	0.010942
6.21	0.141298	0.173169	0.002186
6.22	0.134928	0.165525	-0.00658
6.23	0.128579	0.157825	-0.01535
6.24	0.122259	0.150074	-0.02412
6.25	0.115977	0.142279	-0.03289
6.26	0.109741	0.134447	-0.04163
6.27	0.103561	0.126585	-0.05036
6.28	0.097444	0.1187	-0.05905
6.29	0.0914	0.110798	-0.06771
6.3	0.085437	0.102885	-0.07633
6.31	0.079563	0.094968	-0.0849
6.32	0.073778	0.087049	-0.09341
6.33	0.068083	0.079129	-0.10187
6.34	0.06248	0.071208	-0.11025
6.35	0.056967	0.063289	-0.11857
6.36	0.051546	0.055372	-0.12681
6.37	0.046216	0.047459	-0.13497
6.38	0.040978	0.039549	-0.14305
6.39	0.035832	0.031646	-0.15102
6.4	0.030779	0.023749	-0.15891
6.41	0.025817	0.015862	-0.16668
6.42	0.020942	0.007989	-0.17435
6.43	0.016145	0.000139	-0.18189
6.44	0.011421	-0.00768	-0.18931

6.45	0.006762	-0.01546	-0.19659
6.46	0.002162	-0.0232	-0.20373
6.47	-0.00239	-0.03089	-0.21072
6.48	-0.00689	-0.03851	-0.21756
6.49	-0.01136	-0.04607	-0.22422
6.5	-0.01579	-0.05356	-0.23072
6.51	-0.0202	-0.06096	-0.23703
6.52	-0.02459	-0.06827	-0.24316
6.53	-0.02897	-0.07549	-0.2491
6.54	-0.03333	-0.08261	-0.25485
6.55	-0.03769	-0.08962	-0.26042
6.56	-0.04205	-0.09652	-0.26578
6.57	-0.04641	-0.10329	-0.27096
6.58	-0.05078	-0.10993	-0.27593
6.59	-0.05517	-0.11644	-0.2807
6.6	-0.05957	-0.12281	-0.28527
6.61	-0.064	-0.12903	-0.28963
6.62	-0.06845	-0.1351	-0.29379
6.63	-0.07293	-0.14102	-0.29776
6.64	-0.07744	-0.14681	-0.30154
6.65	-0.082	-0.15245	-0.30513
6.66	-0.08659	-0.15795	-0.30856
6.67	-0.09123	-0.16332	-0.31181
6.68	-0.09592	-0.16854	-0.3149
6.69	-0.10066	-0.17364	-0.31783
6.7	-0.10546	-0.1786	-0.32062
6.71	-0.11031	-0.18343	-0.32326
6.72	-0.11523	-0.18813	-0.32575
6.73	-0.1202	-0.1927	-0.3281
6.74	-0.12522	-0.19715	-0.33031
6.75	-0.13029	-0.20146	-0.33238
6.76	-0.13541	-0.20564	-0.33431
6.77	-0.14057	-0.20969	-0.3361
6.78	-0.14578	-0.21361	-0.33774
6.79	-0.15103	-0.21741	-0.33925
6.8	-0.15631	-0.22107	-0.34062
6.81	-0.16163	-0.22461	-0.34185
6.82	-0.16697	-0.22803	-0.34295
6.83	-0.17235	-0.23134	-0.34394
6.84	-0.17774	-0.23454	-0.34484
6.85	-0.18316	-0.23765	-0.34564
6.86	-0.18859	-0.24067	-0.34637
6.87	-0.19403	-0.24361	-0.34704
6.88	-0.19947	-0.24649	-0.34767
6.89	-0.20492	-0.2493	-0.34825
6.9	-0.21037	-0.25207	-0.34882
6.91	-0.21581	-0.25479	-0.34937
6.92	-0.22124	-0.25747	-0.34993

6.93	-0.22666	-0.26011	-0.35048
6.94	-0.23207	-0.26273	-0.35103
6.95	-0.23745	-0.26533	-0.3516
6.96	-0.24282	-0.26791	-0.35217
6.97	-0.24816	-0.27047	-0.35277
6.98	-0.25347	-0.27303	-0.35338
6.99	-0.25875	-0.27559	-0.35403
7	-0.264	-0.27814	-0.3547
7.01	-0.26921	-0.28071	-0.35541
7.02	-0.27438	-0.28329	-0.35616
7.03	-0.27951	-0.28589	-0.35694
7.04	-0.2846	-0.28851	-0.35776
7.05	-0.28964	-0.29116	-0.35861
7.06	-0.29463	-0.29384	-0.3595
7.07	-0.29958	-0.29657	-0.36042
7.08	-0.30448	-0.29934	-0.36138
7.09	-0.30933	-0.30215	-0.36238
7.1	-0.31412	-0.30503	-0.36341
7.11	-0.31886	-0.30796	-0.36447
7.12	-0.32355	-0.31095	-0.36558
7.13	-0.32818	-0.314	-0.36673
7.14	-0.33275	-0.3171	-0.36794
7.15	-0.33727	-0.32025	-0.3692
7.16	-0.34173	-0.32345	-0.37052
7.17	-0.34614	-0.32669	-0.37191
7.18	-0.35049	-0.32998	-0.37337
7.19	-0.35479	-0.3333	-0.37491
7.2	-0.35902	-0.33667	-0.37653
7.21	-0.3632	-0.34007	-0.37823
7.22	-0.36733	-0.3435	-0.38001
7.23	-0.37139	-0.34696	-0.38187
7.24	-0.3754	-0.35043	-0.38379
7.25	-0.37936	-0.35393	-0.38576
7.26	-0.38326	-0.35744	-0.38778
7.27	-0.3871	-0.36095	-0.38984
7.28	-0.39088	-0.36447	-0.39192
7.29	-0.39461	-0.36799	-0.39404
7.3	-0.39829	-0.3715	-0.39616
7.31	-0.40191	-0.375	-0.3983
7.32	-0.40547	-0.37849	-0.40044
7.33	-0.40898	-0.38196	-0.40259
7.34	-0.41243	-0.38542	-0.40474
7.35	-0.41583	-0.38887	-0.4069
7.36	-0.41917	-0.39229	-0.40907
7.37	-0.42245	-0.39569	-0.41124
7.38	-0.42568	-0.39908	-0.41341
7.39	-0.42884	-0.40244	-0.4156
7.4	-0.43195	-0.40577	-0.41778

7.41	-0.435	-0.40908	-0.41997
7.42	-0.43799	-0.41235	-0.42217
7.43	-0.44092	-0.41558	-0.42435
7.44	-0.44379	-0.41877	-0.42653
7.45	-0.4466	-0.42189	-0.42869
7.46	-0.44936	-0.42496	-0.43083
7.47	-0.45204	-0.42795	-0.43295
7.48	-0.45467	-0.43087	-0.43504
7.49	-0.45724	-0.4337	-0.43709
7.5	-0.45974	-0.43643	-0.4391
7.51	-0.46219	-0.43907	-0.44107
7.52	-0.46456	-0.44162	-0.44299
7.53	-0.46688	-0.44406	-0.44487
7.54	-0.46913	-0.44642	-0.44668
7.55	-0.47131	-0.44869	-0.44844
7.56	-0.47342	-0.45087	-0.45015
7.57	-0.47547	-0.45297	-0.45179
7.58	-0.47745	-0.45499	-0.45336
7.59	-0.47936	-0.45693	-0.45486
7.6	-0.48119	-0.4588	-0.4563
7.61	-0.48296	-0.46059	-0.45765
7.62	-0.48465	-0.46232	-0.45894
7.63	-0.48626	-0.46398	-0.46015
7.64	-0.4878	-0.46556	-0.4613
7.65	-0.48927	-0.46708	-0.46237
7.66	-0.49065	-0.46852	-0.46339
7.67	-0.49196	-0.4699	-0.46433
7.68	-0.49318	-0.47121	-0.46521
7.69	-0.49433	-0.47245	-0.46604
7.7	-0.49539	-0.47362	-0.4668
7.71	-0.49636	-0.47473	-0.46751
7.72	-0.49725	-0.47576	-0.46815
7.73	-0.49806	-0.47673	-0.46873
7.74	-0.49878	-0.47762	-0.46925
7.75	-0.49942	-0.47844	-0.4697
7.76	-0.49998	-0.47919	-0.47008
7.77	-0.50045	-0.47987	-0.47039
7.78	-0.50084	-0.48046	-0.47063
7.79	-0.50115	-0.48098	-0.47079
7.8	-0.50137	-0.48143	-0.47087
7.81	-0.50152	-0.48179	-0.47086
7.82	-0.50158	-0.48208	-0.47078
7.83	-0.50156	-0.48229	-0.47063
7.84	-0.50145	-0.48244	-0.4704
7.85	-0.50127	-0.48251	-0.4701
7.86	-0.50101	-0.48253	-0.46973
7.87	-0.50067	-0.48248	-0.4693
7.88	-0.50025	-0.48238	-0.46881

7.89	-0.49975	-0.48222	-0.46827
7.9	-0.49917	-0.48202	-0.46766
7.91	-0.49851	-0.48176	-0.46701
7.92	-0.49777	-0.48146	-0.46629
7.93	-0.49695	-0.48111	-0.46552
7.94	-0.49605	-0.4807	-0.46468
7.95	-0.49505	-0.48025	-0.46376
7.96	-0.49396	-0.47974	-0.46277
7.97	-0.49278	-0.47918	-0.46169
7.98	-0.49151	-0.47856	-0.46053
7.99	-0.49013	-0.47789	-0.45928
8	-0.48865	-0.47716	-0.45792
8.01	-0.48707	-0.47637	-0.45647
8.02	-0.48538	-0.47553	-0.45491
8.03	-0.48359	-0.47462	-0.45324
8.04	-0.48169	-0.47365	-0.45147
8.05	-0.47968	-0.47262	-0.44959
8.06	-0.47757	-0.47152	-0.44761
8.07	-0.47535	-0.47036	-0.44552
8.08	-0.47302	-0.46914	-0.44331
8.09	-0.47058	-0.46784	-0.441
8.1	-0.46803	-0.46648	-0.43858
8.11	-0.46538	-0.46505	-0.43604
8.12	-0.46261	-0.46355	-0.43339
8.13	-0.45974	-0.46197	-0.43063
8.14	-0.45676	-0.46032	-0.42775
8.15	-0.45367	-0.4586	-0.42475
8.16	-0.45047	-0.4568	-0.42163
8.17	-0.44717	-0.45493	-0.41839
8.18	-0.44376	-0.45297	-0.41503
8.19	-0.44025	-0.45094	-0.41155
8.2	-0.43663	-0.44882	-0.40793
8.21	-0.43291	-0.44662	-0.40419
8.22	-0.42908	-0.44433	-0.40033
8.23	-0.42514	-0.44196	-0.39632
8.24	-0.4211	-0.43949	-0.39219
8.25	-0.41695	-0.43693	-0.38792
8.26	-0.41269	-0.43428	-0.3835
8.27	-0.40832	-0.43153	-0.37895
8.28	-0.40384	-0.42868	-0.37425
8.29	-0.39925	-0.42572	-0.36941
8.3	-0.39454	-0.42267	-0.36441
8.31	-0.38972	-0.4195	-0.35927
8.32	-0.3848	-0.41622	-0.35398
8.33	-0.37979	-0.41283	-0.34855
8.34	-0.37469	-0.40933	-0.34297
8.35	-0.36952	-0.40571	-0.33725
8.36	-0.36429	-0.40197	-0.33141

8.37	-0.359	-0.39812	-0.32542
8.38	-0.35367	-0.39414	-0.31932
8.39	-0.34831	-0.39003	-0.31308
8.4	-0.34293	-0.3858	-0.30673
8.41	-0.33753	-0.38144	-0.30026
8.42	-0.33212	-0.37696	-0.29366
8.43	-0.32669	-0.37235	-0.28695
8.44	-0.32123	-0.36761	-0.28012
8.45	-0.31574	-0.36276	-0.27316
8.46	-0.31021	-0.35779	-0.26608
8.47	-0.30464	-0.35271	-0.25888
8.48	-0.29903	-0.34751	-0.25155
8.49	-0.29337	-0.34219	-0.24409
8.5	-0.28766	-0.33677	-0.2365
8.51	-0.28189	-0.33124	-0.22879
8.52	-0.27606	-0.32561	-0.22095
8.53	-0.27018	-0.31986	-0.213
8.54	-0.26425	-0.31401	-0.20494
8.55	-0.25828	-0.30804	-0.19678
8.56	-0.25227	-0.30196	-0.18852
8.57	-0.24621	-0.29578	-0.18017
8.58	-0.24012	-0.28948	-0.17174
8.59	-0.234	-0.28306	-0.16324
8.6	-0.22785	-0.27654	-0.15467
8.61	-0.22168	-0.26989	-0.14603
8.62	-0.21548	-0.26314	-0.13735
8.63	-0.20926	-0.25629	-0.12862
8.64	-0.20302	-0.24932	-0.11987
8.65	-0.19676	-0.24227	-0.11111
8.66	-0.19048	-0.23511	-0.10233
8.67	-0.18418	-0.22787	-0.09357
8.68	-0.17787	-0.22054	-0.08482
8.69	-0.17155	-0.21312	-0.0761
8.7	-0.16521	-0.20563	-0.06742
8.71	-0.15886	-0.19806	-0.05879
8.72	-0.15251	-0.19043	-0.05021
8.73	-0.14616	-0.18273	-0.0417
8.74	-0.13982	-0.17499	-0.03325
8.75	-0.13351	-0.16719	-0.02487
8.76	-0.12723	-0.15936	-0.01657
8.77	-0.12098	-0.15151	-0.00835
8.78	-0.11478	-0.14363	-0.00021
8.79	-0.10864	-0.13573	0.007838
8.8	-0.10256	-0.12783	0.015793
8.81	-0.09654	-0.11993	0.023649
8.82	-0.0906	-0.11202	0.031405
8.83	-0.08474	-0.10412	0.039057
8.84	-0.07895	-0.09621	0.046605

8.85	-0.07325	-0.0883	0.054047
8.86	-0.06763	-0.08039	0.061379
8.87	-0.0621	-0.07248	0.068601
8.88	-0.05666	-0.06456	0.07571
8.89	-0.0513	-0.05663	0.082704
8.9	-0.04605	-0.0487	0.089582
8.91	-0.04089	-0.04076	0.09634
8.92	-0.03582	-0.03282	0.102973
8.93	-0.03084	-0.02489	0.109473
8.94	-0.02594	-0.01697	0.115833
8.95	-0.02111	-0.00907	0.122047
8.96	-0.01636	-0.0012	0.128108
8.97	-0.01167	0.006645	0.134008
8.98	-0.00704	0.014446	0.139741
8.99	-0.00247	0.022201	0.145301
9	0.002056	0.029904	0.150679
9.01	0.006536	0.037546	0.155871
9.02	0.010978	0.045122	0.160885
9.03	0.015388	0.052624	0.16573
9.04	0.019771	0.060043	0.170413
9.05	0.024133	0.067373	0.174945
9.06	0.028481	0.074606	0.179335
9.07	0.032819	0.081735	0.183592
9.08	0.037155	0.088753	0.187725
9.09	0.041493	0.095651	0.191743
9.1	0.04584	0.102423	0.195655
9.11	0.050201	0.109062	0.19947
9.12	0.054582	0.115569	0.203195
9.13	0.058989	0.121947	0.206837
9.14	0.063427	0.128199	0.210403
9.15	0.067902	0.134326	0.2139
9.16	0.072418	0.140331	0.217335
9.17	0.076983	0.146217	0.220714
9.18	0.081601	0.151986	0.224045
9.19	0.086277	0.15764	0.227333
9.2	0.091018	0.163182	0.230587
9.21	0.095828	0.168614	0.233811
9.22	0.100705	0.173932	0.237006
9.23	0.105645	0.179133	0.240171
9.24	0.110647	0.184214	0.243304
9.25	0.115706	0.189172	0.246406
9.26	0.120819	0.194002	0.249474
9.27	0.125984	0.1987	0.252507
9.28	0.131198	0.203265	0.255506
9.29	0.136457	0.207691	0.258467
9.3	0.141758	0.211976	0.261392
9.31	0.147098	0.216116	0.264279
9.32	0.152473	0.220114	0.267132

9.33	0.157879	0.22397	0.269957
9.34	0.163311	0.227687	0.27276
9.35	0.168766	0.231267	0.275544
9.36	0.174238	0.234712	0.278315
9.37	0.179724	0.238025	0.281078
9.38	0.18522	0.241206	0.283839
9.39	0.19072	0.244258	0.286603
9.4	0.196222	0.247183	0.289374
9.41	0.20172	0.249985	0.292157
9.42	0.207211	0.252676	0.294955
9.43	0.212691	0.255271	0.297771
9.44	0.218156	0.257783	0.300605
9.45	0.223603	0.260228	0.30346
9.46	0.229027	0.262618	0.306338
9.47	0.234425	0.264968	0.309242
9.48	0.239792	0.267293	0.312173
9.49	0.245126	0.269606	0.315133
9.5	0.250421	0.271922	0.318125
9.51	0.255676	0.274253	0.321149
9.52	0.260887	0.276601	0.324203
9.53	0.266054	0.278967	0.327283
9.54	0.271176	0.28135	0.330384
9.55	0.276251	0.283753	0.333504
9.56	0.281279	0.286174	0.336638
9.57	0.286257	0.288614	0.339782
9.58	0.291185	0.291074	0.342933
9.59	0.296062	0.293554	0.346086
9.6	0.300886	0.296055	0.349238
9.61	0.305656	0.298576	0.352386
9.62	0.310372	0.301119	0.355529
9.63	0.315034	0.303685	0.358667
9.64	0.319642	0.306274	0.361802
9.65	0.324195	0.308889	0.364932
9.66	0.328694	0.311529	0.368059
9.67	0.333138	0.314196	0.371183
9.68	0.337528	0.316891	0.374305
9.69	0.341863	0.319615	0.377423
9.7	0.346143	0.322368	0.38054
9.71	0.350368	0.325153	0.383655
9.72	0.354538	0.327971	0.386774
9.73	0.358653	0.330823	0.389899
9.74	0.362713	0.333712	0.393036
9.75	0.366719	0.336639	0.396189
9.76	0.370669	0.339607	0.399362
9.77	0.374565	0.342618	0.40256
9.78	0.378407	0.345672	0.405788
9.79	0.382193	0.348773	0.409049
9.8	0.385925	0.351922	0.412349

9.81	0.389602	0.355119	0.415689
9.82	0.393225	0.358355	0.419065
9.83	0.396791	0.361621	0.422469
9.84	0.400302	0.364906	0.425895
9.85	0.403757	0.368199	0.429334
9.86	0.407154	0.37149	0.432779
9.87	0.410495	0.374769	0.436224
9.88	0.413777	0.378025	0.439662
9.89	0.417002	0.381248	0.443084
9.9	0.420168	0.384428	0.446484
9.91	0.423275	0.387555	0.449854
9.92	0.426322	0.390628	0.453186
9.93	0.429308	0.393648	0.456473
9.94	0.432233	0.396613	0.459705
9.95	0.435096	0.399524	0.462875
9.96	0.437897	0.402382	0.465973
9.97	0.440634	0.405184	0.468993
9.98	0.443306	0.407933	0.471925
9.99	0.445914	0.410627	0.474762
10	0.448456	0.413267	0.477495
10.01	0.450932	0.415853	0.480116
10.02	0.45334	0.418386	0.482623
10.03	0.455679	0.420868	0.485012
10.04	0.457949	0.4233	0.48728
10.05	0.460149	0.425684	0.489425
10.06	0.462278	0.428022	0.491443
10.07	0.464334	0.430314	0.493333
10.08	0.466317	0.432564	0.495091
10.09	0.468225	0.434772	0.496714
10.1	0.470059	0.436939	0.4982
10.11	0.471816	0.439068	0.499545
10.12	0.473496	0.441155	0.500747
10.13	0.475098	0.443197	0.501801
10.14	0.476621	0.445191	0.502703
10.15	0.478065	0.447134	0.503449
10.16	0.479428	0.449023	0.504036
10.17	0.48071	0.450855	0.504461
10.18	0.481909	0.452626	0.504718
10.19	0.483026	0.454334	0.504804
10.2	0.484058	0.455975	0.504716
10.21	0.485006	0.457546	0.50445
10.22	0.48587	0.459051	0.504005
10.23	0.48665	0.460491	0.503381
10.24	0.487347	0.461868	0.502576
10.25	0.487961	0.463185	0.501592
10.26	0.488493	0.464444	0.500426
10.27	0.488943	0.465649	0.499079
10.28	0.489311	0.466801	0.497551

10.29	0.4896	0.467903	0.49584
10.3	0.489807	0.468957	0.493946
10.31	0.489935	0.469965	0.491871
10.32	0.489983	0.470925	0.489625
10.33	0.489948	0.471834	0.48722
10.34	0.48983	0.472689	0.484667
10.35	0.489627	0.473487	0.48198
10.36	0.489339	0.474226	0.479169
10.37	0.488964	0.474902	0.476248
10.38	0.488501	0.475512	0.473227
10.39	0.487949	0.476053	0.47012
10.4	0.487307	0.476523	0.466939
10.41	0.486573	0.476919	0.463692
10.42	0.485747	0.477239	0.460386
10.43	0.484827	0.477481	0.45702
10.44	0.483814	0.477643	0.453598
10.45	0.482707	0.477723	0.450121
10.46	0.481503	0.477719	0.446591
10.47	0.480204	0.47763	0.443011
10.48	0.478808	0.477454	0.439382
10.49	0.477313	0.477189	0.435705
10.5	0.475721	0.476833	0.431985
10.51	0.474029	0.476384	0.42822
10.52	0.472239	0.475842	0.424413
10.53	0.470351	0.475205	0.420563
10.54	0.468368	0.474473	0.416669
10.55	0.46629	0.473644	0.412731
10.56	0.464118	0.472719	0.408749
10.57	0.461854	0.471695	0.404723
10.58	0.459498	0.470573	0.400653
10.59	0.457052	0.469351	0.396538
10.6	0.454517	0.468028	0.392378
10.61	0.451893	0.466603	0.388173
10.62	0.449178	0.465073	0.383925
10.63	0.446369	0.463434	0.379635
10.64	0.443465	0.461682	0.375304
10.65	0.440461	0.459813	0.370935
10.66	0.437356	0.457823	0.366529
10.67	0.434146	0.455709	0.362086
10.68	0.43083	0.453466	0.35761
10.69	0.427403	0.451091	0.353101
10.7	0.423864	0.44858	0.348562
10.71	0.420211	0.44593	0.343992
10.72	0.416444	0.443143	0.339385
10.73	0.412566	0.440221	0.334734
10.74	0.408578	0.437167	0.330032
10.75	0.404484	0.433983	0.325271
10.76	0.400284	0.430672	0.320445

10.77	0.39598	0.427237	0.315546
10.78	0.391576	0.423679	0.310567
10.79	0.387071	0.420003	0.3055
10.8	0.38247	0.41621	0.300338
10.81	0.377773	0.412302	0.295075
10.82	0.372986	0.40828	0.289709
10.83	0.368112	0.404145	0.284242
10.84	0.363157	0.399897	0.278671
10.85	0.358124	0.395535	0.272997
10.86	0.353019	0.391061	0.267219
10.87	0.347846	0.386474	0.261338
10.88	0.34261	0.381775	0.255353
10.89	0.337314	0.376964	0.249264
10.9	0.331964	0.372041	0.24307
10.91	0.326564	0.367007	0.236772
10.92	0.321113	0.361864	0.23037
10.93	0.315612	0.356614	0.223866
10.94	0.310058	0.351261	0.217259
10.95	0.304453	0.345806	0.210552
10.96	0.298794	0.340253	0.203745
10.97	0.293082	0.334603	0.196839
10.98	0.287316	0.328859	0.189834
10.99	0.281495	0.323023	0.182733
11	0.275618	0.317099	0.175535
11.01	0.269687	0.311089	0.168242
11.02	0.263701	0.304994	0.160856
11.03	0.257666	0.298818	0.153377
11.04	0.251584	0.292563	0.145806
11.05	0.245457	0.286232	0.138147
11.06	0.239289	0.279826	0.130399
11.07	0.233083	0.273349	0.122564
11.08	0.226842	0.266802	0.114644
11.09	0.220569	0.260188	0.10664
11.1	0.214266	0.25351	0.098553
11.11	0.207936	0.246769	0.090387
11.12	0.201582	0.239965	0.082145
11.13	0.195206	0.233097	0.073834
11.14	0.18881	0.226165	0.065462
11.15	0.182396	0.219167	0.057034
11.16	0.175966	0.212102	0.048556
11.17	0.169522	0.20497	0.040035
11.18	0.163066	0.19777	0.031477
11.19	0.156601	0.190501	0.022888
11.2	0.150128	0.183161	0.014275
11.21	0.14365	0.175752	0.005645
11.22	0.137176	0.168277	-0.003
11.23	0.130714	0.160741	-0.01165
11.24	0.124273	0.153151	-0.0203

11.25	0.117863	0.145512	-0.02895
11.26	0.111491	0.137828	-0.03758
11.27	0.105168	0.130106	-0.0462
11.28	0.098901	0.12235	-0.0548
11.29	0.092701	0.114566	-0.06337
11.3	0.086575	0.10676	-0.07191
11.31	0.080532	0.098936	-0.08041
11.32	0.074576	0.091096	-0.08887
11.33	0.068707	0.083242	-0.09728
11.34	0.062929	0.075373	-0.10564
11.35	0.057245	0.067492	-0.11393
11.36	0.051656	0.0596	-0.12216
11.37	0.046164	0.051698	-0.13032
11.38	0.040772	0.043786	-0.13841
11.39	0.035483	0.035867	-0.14641
11.4	0.030298	0.027941	-0.15433
11.41	0.025218	0.020011	-0.16215
11.42	0.020238	0.012083	-0.16988
11.43	0.015351	0.004165	-0.17749
11.44	0.010548	-0.00374	-0.18499
11.45	0.005824	-0.01161	-0.19236
11.46	0.00117	-0.01945	-0.1996
11.47	-0.00342	-0.02725	-0.2067
11.48	-0.00795	-0.035	-0.21364
11.49	-0.01244	-0.0427	-0.22042
11.5	-0.01688	-0.05032	-0.22704
11.51	-0.02129	-0.05788	-0.23348
11.52	-0.02567	-0.06536	-0.23974
11.53	-0.03003	-0.07275	-0.24582
11.54	-0.03437	-0.08004	-0.25171
11.55	-0.03869	-0.08723	-0.25742
11.56	-0.04301	-0.09431	-0.26295
11.57	-0.04732	-0.10128	-0.26828
11.58	-0.05164	-0.10811	-0.27342
11.59	-0.05597	-0.11481	-0.27836
11.6	-0.06031	-0.12138	-0.28311
11.61	-0.06467	-0.12779	-0.28766
11.62	-0.06905	-0.13406	-0.29202
11.63	-0.07346	-0.14018	-0.29619
11.64	-0.07791	-0.14615	-0.30017
11.65	-0.08239	-0.15198	-0.30398
11.66	-0.08691	-0.15766	-0.30761
11.67	-0.09148	-0.1632	-0.31107
11.68	-0.0961	-0.1686	-0.31437
11.69	-0.10077	-0.17386	-0.3175
11.7	-0.10551	-0.17897	-0.32048
11.71	-0.1103	-0.18395	-0.32332
11.72	-0.11516	-0.18879	-0.326

11.73	-0.12007	-0.19348	-0.32853
11.74	-0.12505	-0.19804	-0.33091
11.75	-0.13007	-0.20246	-0.33314
11.76	-0.13515	-0.20674	-0.33522
11.77	-0.14027	-0.21089	-0.33715
11.78	-0.14544	-0.21489	-0.33894
11.79	-0.15065	-0.21875	-0.34057
11.8	-0.1559	-0.22248	-0.34206
11.81	-0.16119	-0.22607	-0.3434
11.82	-0.16652	-0.22953	-0.3446
11.83	-0.17187	-0.23287	-0.34569
11.84	-0.17725	-0.23609	-0.34666
11.85	-0.18265	-0.23921	-0.34753
11.86	-0.18807	-0.24224	-0.34833
11.87	-0.1935	-0.24518	-0.34905
11.88	-0.19894	-0.24805	-0.34971
11.89	-0.20439	-0.25084	-0.35034
11.9	-0.20984	-0.25357	-0.35092
11.91	-0.21529	-0.25626	-0.35149
11.92	-0.22073	-0.25889	-0.35205
11.93	-0.22616	-0.26149	-0.3526
11.94	-0.23158	-0.26405	-0.35314
11.95	-0.23699	-0.26659	-0.35368
11.96	-0.24237	-0.2691	-0.35424
11.97	-0.24773	-0.2716	-0.3548
11.98	-0.25306	-0.27409	-0.35538
11.99	-0.25836	-0.27658	-0.35598
12	-0.26362	-0.27907	-0.35661
12.01	-0.26885	-0.28157	-0.35726
12.02	-0.27403	-0.28409	-0.35795
12.03	-0.27918	-0.28662	-0.35868
12.04	-0.28427	-0.28918	-0.35943
12.05	-0.28933	-0.29177	-0.36022
12.06	-0.29434	-0.2944	-0.36103
12.07	-0.29929	-0.29706	-0.36189
12.08	-0.3042	-0.29977	-0.36277
12.09	-0.30905	-0.30254	-0.36369
12.1	-0.31386	-0.30535	-0.36464
12.11	-0.3186	-0.30823	-0.36562
12.12	-0.32329	-0.31117	-0.36664
12.13	-0.32792	-0.31416	-0.36771
12.14	-0.3325	-0.31721	-0.36883
12.15	-0.33702	-0.3203	-0.37
12.16	-0.34149	-0.32345	-0.37124
12.17	-0.34589	-0.32665	-0.37254
12.18	-0.35025	-0.32989	-0.37391
12.19	-0.35454	-0.33317	-0.37536
12.2	-0.35878	-0.33649	-0.37689

12.21	-0.36296	-0.33985	-0.37851
12.22	-0.36709	-0.34324	-0.38022
12.23	-0.37116	-0.34667	-0.38199
12.24	-0.37517	-0.35011	-0.38383
12.25	-0.37913	-0.35358	-0.38573
12.26	-0.38302	-0.35706	-0.38768
12.27	-0.38687	-0.36056	-0.38967
12.28	-0.39065	-0.36405	-0.3917
12.29	-0.39438	-0.36755	-0.39375
12.3	-0.39805	-0.37105	-0.39582
12.31	-0.40166	-0.37454	-0.3979
12.32	-0.40522	-0.37802	-0.4
12.33	-0.40872	-0.3815	-0.4021
12.34	-0.41217	-0.38495	-0.40422
12.35	-0.41555	-0.3884	-0.40634
12.36	-0.41888	-0.39182	-0.40848
12.37	-0.42215	-0.39523	-0.41062
12.38	-0.42536	-0.39862	-0.41278
12.39	-0.42852	-0.40198	-0.41494
12.4	-0.43162	-0.40532	-0.41711
12.41	-0.43466	-0.40864	-0.41928
12.42	-0.43764	-0.41192	-0.42146
12.43	-0.44057	-0.41516	-0.42364
12.44	-0.44344	-0.41835	-0.42581
12.45	-0.44624	-0.42148	-0.42797
12.46	-0.44899	-0.42455	-0.43011
12.47	-0.45168	-0.42756	-0.43223
12.48	-0.4543	-0.43048	-0.43433
12.49	-0.45687	-0.43332	-0.43639
12.5	-0.45937	-0.43606	-0.43841
12.51	-0.46181	-0.43871	-0.44039
12.52	-0.46419	-0.44126	-0.44233
12.53	-0.4665	-0.44372	-0.44422
12.54	-0.46875	-0.44608	-0.44606
12.55	-0.47093	-0.44836	-0.44784
12.56	-0.47304	-0.45055	-0.44956
12.57	-0.47509	-0.45265	-0.45123
12.58	-0.47706	-0.45468	-0.45282
12.59	-0.47897	-0.45663	-0.45435
12.6	-0.4808	-0.4585	-0.45581
12.61	-0.48256	-0.46031	-0.45719
12.62	-0.48425	-0.46204	-0.4585
12.63	-0.48586	-0.4637	-0.45973
12.64	-0.4874	-0.46529	-0.4609
12.65	-0.48886	-0.46681	-0.46199
12.66	-0.49024	-0.46826	-0.46302
12.67	-0.49154	-0.46964	-0.46399
12.68	-0.49275	-0.47095	-0.46489

12.69	-0.49389	-0.47219	-0.46572
12.7	-0.49495	-0.47336	-0.4665
12.71	-0.49591	-0.47447	-0.46722
12.72	-0.4968	-0.4755	-0.46787
12.73	-0.4976	-0.47647	-0.46846
12.74	-0.49833	-0.47736	-0.46899
12.75	-0.49896	-0.47818	-0.46944
12.76	-0.49952	-0.47893	-0.46983
12.77	-0.5	-0.4796	-0.47014
12.78	-0.5004	-0.48019	-0.47038
12.79	-0.50072	-0.48071	-0.47054
12.8	-0.50097	-0.48115	-0.47062
12.81	-0.50113	-0.48151	-0.47062
12.82	-0.50122	-0.4818	-0.47054
12.83	-0.50122	-0.48201	-0.47039
12.84	-0.50114	-0.48215	-0.47016
12.85	-0.50097	-0.48223	-0.46986
12.86	-0.50072	-0.48224	-0.46949
12.87	-0.50038	-0.48219	-0.46905
12.88	-0.49994	-0.48208	-0.46856
12.89	-0.49941	-0.48192	-0.468
12.9	-0.49878	-0.4817	-0.46739
12.91	-0.49805	-0.48144	-0.46672
12.92	-0.49722	-0.48113	-0.46599
12.93	-0.4963	-0.48076	-0.4652
12.94	-0.49528	-0.48035	-0.46434
12.95	-0.49416	-0.47988	-0.46341
12.96	-0.49294	-0.47936	-0.46239
12.97	-0.49164	-0.47878	-0.4613
12.98	-0.49024	-0.47815	-0.46011
12.99	-0.48874	-0.47746	-0.45883
13	-0.48716	-0.47671	-0.45746
13.01	-0.48548	-0.4759	-0.45597
13.02	-0.48371	-0.47503	-0.45439
13.03	-0.48185	-0.4741	-0.4527
13.04	-0.47989	-0.47311	-0.4509
13.05	-0.47785	-0.47206	-0.449
13.06	-0.47571	-0.47094	-0.44699
13.07	-0.47348	-0.46975	-0.44487
13.08	-0.47115	-0.4685	-0.44264
13.09	-0.46873	-0.46718	-0.4403
13.1	-0.46622	-0.4658	-0.43785
13.11	-0.46361	-0.46434	-0.43529
13.12	-0.4609	-0.46282	-0.43262
13.13	-0.4581	-0.46122	-0.42983
13.14	-0.4552	-0.45955	-0.42692
13.15	-0.45221	-0.4578	-0.4239
13.16	-0.44911	-0.45598	-0.42076

13.17	-0.44591	-0.45408	-0.41749
13.18	-0.44261	-0.45211	-0.41411
13.19	-0.43921	-0.45005	-0.4106
13.2	-0.4357	-0.44791	-0.40696
13.21	-0.43209	-0.44569	-0.4032
13.22	-0.42837	-0.44339	-0.3993
13.23	-0.42455	-0.441	-0.39528
13.24	-0.42062	-0.43852	-0.39112
13.25	-0.41658	-0.43595	-0.38683
13.26	-0.41243	-0.43328	-0.3824
13.27	-0.40818	-0.43053	-0.37783
13.28	-0.40381	-0.42767	-0.37312
13.29	-0.39933	-0.42472	-0.36827
13.3	-0.39474	-0.42166	-0.36328
13.31	-0.39004	-0.41851	-0.35814
13.32	-0.38524	-0.41525	-0.35286
13.33	-0.38034	-0.41188	-0.34744
13.34	-0.37536	-0.4084	-0.34188
13.35	-0.3703	-0.40481	-0.33619
13.36	-0.36517	-0.4011	-0.33037
13.37	-0.35999	-0.39728	-0.32443
13.38	-0.35476	-0.39334	-0.31836
13.39	-0.34949	-0.38929	-0.31217
13.4	-0.34418	-0.38511	-0.30586
13.41	-0.33886	-0.3808	-0.29944
13.42	-0.3335	-0.37637	-0.2929
13.43	-0.32812	-0.37183	-0.28625
13.44	-0.32271	-0.36716	-0.27948
13.45	-0.31726	-0.36238	-0.27259
13.46	-0.31177	-0.35749	-0.26558
13.47	-0.30623	-0.35248	-0.25845
13.48	-0.30065	-0.34736	-0.2512
13.49	-0.29502	-0.34214	-0.24382
13.5	-0.28933	-0.33681	-0.23632
13.51	-0.28358	-0.33138	-0.22869
13.52	-0.27778	-0.32585	-0.22095
13.53	-0.27193	-0.32021	-0.21309
13.54	-0.26602	-0.31446	-0.20512
13.55	-0.26007	-0.30861	-0.19705
13.56	-0.25408	-0.30264	-0.18889
13.57	-0.24805	-0.29657	-0.18065
13.58	-0.24198	-0.29038	-0.17232
13.59	-0.23588	-0.28408	-0.16392
13.6	-0.22975	-0.27767	-0.15546
13.61	-0.22359	-0.27114	-0.14694
13.62	-0.21741	-0.2645	-0.13836
13.63	-0.2112	-0.25776	-0.12975
13.64	-0.20496	-0.25091	-0.12111

13.65	-0.1987	-0.24396	-0.11246
13.66	-0.19241	-0.23691	-0.10379
13.67	-0.1861	-0.22976	-0.09514
13.68	-0.17977	-0.22253	-0.08649
13.69	-0.17341	-0.21521	-0.07787
13.7	-0.16702	-0.20781	-0.06928
13.71	-0.16061	-0.20033	-0.06074
13.72	-0.15419	-0.19277	-0.05225
13.73	-0.14776	-0.18515	-0.04382
13.74	-0.14134	-0.17748	-0.03545
13.75	-0.13493	-0.16975	-0.02714
13.76	-0.12854	-0.16198	-0.0189
13.77	-0.12219	-0.15418	-0.01074
13.78	-0.11587	-0.14635	-0.00266
13.79	-0.10961	-0.13849	0.005341
13.8	-0.1034	-0.13063	0.013247
13.81	-0.09727	-0.12275	0.021058
13.82	-0.0912	-0.11487	0.028773
13.83	-0.08521	-0.10698	0.036389
13.84	-0.07931	-0.09908	0.043906
13.85	-0.07348	-0.09117	0.051323
13.86	-0.06774	-0.08325	0.058637
13.87	-0.06209	-0.07531	0.065847
13.88	-0.05654	-0.06737	0.072953
13.89	-0.05109	-0.0594	0.079952
13.9	-0.04573	-0.05143	0.086843
13.91	-0.04048	-0.04343	0.093624
13.92	-0.03533	-0.03543	0.100289
13.93	-0.03028	-0.02742	0.10683
13.94	-0.02531	-0.01942	0.11324
13.95	-0.02043	-0.01144	0.119511
13.96	-0.01563	-0.00347	0.125636
13.97	-0.0109	0.00447	0.131608
13.98	-0.00624	0.012375	0.137419
13.99	-0.00165	0.020239	0.143063
14	0.002889	0.028052	0.148531
14.01	0.007372	0.03581	0.153818
14.02	0.011809	0.043502	0.158932
14.03	0.016206	0.051122	0.163879
14.04	0.020569	0.058662	0.168669
14.05	0.024904	0.066114	0.17331
14.06	0.029219	0.073469	0.17781
14.07	0.03352	0.080721	0.182178
14.08	0.037813	0.087861	0.186422
14.09	0.042105	0.094881	0.19055
14.1	0.046402	0.101773	0.194571
14.11	0.050711	0.108532	0.198493
14.12	0.055037	0.115157	0.202324

14.13	0.059388	0.121652	0.206069
14.14	0.063769	0.128016	0.209736
14.15	0.068186	0.134254	0.213333
14.16	0.072645	0.140366	0.216865
14.17	0.077152	0.146356	0.22034
14.18	0.081714	0.152224	0.223765
14.19	0.086336	0.157974	0.227146
14.2	0.091024	0.163606	0.230491
14.21	0.095784	0.169123	0.233805
14.22	0.100613	0.174522	0.237089
14.23	0.105509	0.179798	0.240339
14.24	0.110469	0.184949	0.243556
14.25	0.11549	0.189971	0.246738
14.26	0.120568	0.19486	0.249883
14.27	0.125701	0.199613	0.25299
14.28	0.130887	0.204226	0.256058
14.29	0.136121	0.208696	0.259084
14.3	0.141402	0.213018	0.262068
14.31	0.146725	0.217191	0.265009
14.32	0.152087	0.221216	0.267912
14.33	0.157483	0.225094	0.270781
14.34	0.162908	0.228828	0.273623
14.35	0.168358	0.232419	0.276441
14.36	0.173829	0.235871	0.279242
14.37	0.179315	0.239185	0.282031
14.38	0.184813	0.242363	0.284813
14.39	0.190316	0.245408	0.287594
14.4	0.195822	0.248321	0.290378
14.41	0.201326	0.251107	0.293171
14.42	0.206823	0.253778	0.295975
14.43	0.21231	0.256349	0.298793
14.44	0.217782	0.258835	0.301627
14.45	0.223237	0.261249	0.304479
14.46	0.22867	0.263607	0.307351
14.47	0.234076	0.265923	0.310246
14.48	0.239453	0.268211	0.313167
14.49	0.244797	0.270487	0.316114
14.5	0.250103	0.272765	0.319092
14.51	0.255368	0.275056	0.3221
14.52	0.26059	0.277365	0.325136
14.53	0.265768	0.27969	0.328197
14.54	0.270901	0.282034	0.331278
14.55	0.275988	0.284396	0.334376
14.56	0.281026	0.286776	0.337487
14.57	0.286016	0.289175	0.340606
14.58	0.290955	0.291594	0.343731
14.59	0.295842	0.294033	0.346858
14.6	0.300676	0.296492	0.349982

14.61	0.305455	0.298972	0.3531
14.62	0.31018	0.301475	0.356212
14.63	0.314851	0.304	0.359319
14.64	0.319466	0.306549	0.36242
14.65	0.324026	0.309123	0.365516
14.66	0.32853	0.311723	0.368607
14.67	0.332979	0.314351	0.371694
14.68	0.337373	0.317006	0.374776
14.69	0.34171	0.319691	0.377855
14.7	0.345991	0.322406	0.38093
14.71	0.350215	0.325153	0.384002
14.72	0.354384	0.327933	0.387075
14.73	0.358496	0.330749	0.390155
14.74	0.362554	0.333602	0.393245
14.75	0.366556	0.336496	0.396351
14.76	0.370503	0.339431	0.399477
14.77	0.374396	0.342411	0.402628
14.78	0.378234	0.345437	0.405809
14.79	0.382018	0.348511	0.409024
14.8	0.385749	0.351636	0.412278
14.81	0.389426	0.354811	0.415574
14.82	0.393049	0.358028	0.418908
14.83	0.396618	0.361277	0.422271
14.84	0.400132	0.364548	0.425656
14.85	0.40359	0.367828	0.429058
14.86	0.406992	0.371109	0.432468
14.87	0.410337	0.37438	0.43588
14.88	0.413625	0.37763	0.439287
14.89	0.416855	0.380848	0.442682
14.9	0.420026	0.384024	0.446057
14.91	0.423139	0.38715	0.449405
14.92	0.426191	0.390224	0.452719
14.93	0.429182	0.393244	0.455989
14.94	0.432111	0.396212	0.459209
14.95	0.434976	0.399127	0.462368
14.96	0.437777	0.401989	0.46546
14.97	0.440512	0.404798	0.468475
14.98	0.44318	0.407553	0.471406
14.99	0.445781	0.410256	0.474244
15	0.448312	0.412904	0.476981
15.01	0.450774	0.4155	0.479609
15.02	0.453165	0.418043	0.482125
15.03	0.455486	0.420535	0.484525
15.04	0.457735	0.422978	0.486807
15.05	0.459914	0.425374	0.488966
15.06	0.462021	0.427723	0.491001
15.07	0.464056	0.430028	0.492908
15.08	0.466019	0.432289	0.494684

15.09	0.467909	0.434508	0.496326
15.1	0.469727	0.436687	0.49783
15.11	0.471472	0.438827	0.499195
15.12	0.473143	0.440924	0.500415
15.13	0.474738	0.442976	0.501487
15.14	0.476257	0.444979	0.502408
15.15	0.477698	0.446931	0.503172
15.16	0.47906	0.448828	0.503777
15.17	0.480342	0.450667	0.504218
15.18	0.481542	0.452444	0.504492
15.19	0.48266	0.454157	0.504594
15.2	0.483694	0.455803	0.504521
15.21	0.484643	0.457378	0.50427
15.22	0.485507	0.458885	0.503838
15.23	0.486287	0.460326	0.503226
15.24	0.486981	0.461704	0.502433
15.25	0.487591	0.46302	0.501459
15.26	0.488116	0.464278	0.500302
15.27	0.488556	0.465479	0.498963
15.28	0.488912	0.466627	0.49744
15.29	0.489184	0.467724	0.495733
15.3	0.489371	0.468772	0.493842
15.31	0.489474	0.469773	0.491767
15.32	0.489496	0.470725	0.48952
15.33	0.489441	0.471626	0.487112
15.34	0.489311	0.472474	0.484557
15.35	0.489109	0.473265	0.481866
15.36	0.48884	0.473998	0.479053
15.37	0.488505	0.47467	0.476129
15.38	0.488109	0.475278	0.473106
15.39	0.487654	0.475821	0.469998
15.4	0.487144	0.476296	0.466817
15.41	0.48658	0.4767	0.463574
15.42	0.485956	0.477031	0.460272
15.43	0.485262	0.477287	0.456914
15.44	0.484491	0.477467	0.453502
15.45	0.483633	0.477568	0.450039
15.46	0.482681	0.477589	0.446527
15.47	0.481625	0.477526	0.442968
15.48	0.480457	0.477379	0.439364
15.49	0.479168	0.477145	0.435718
15.5	0.477751	0.476822	0.432032
15.51	0.476198	0.476409	0.428307
15.52	0.474511	0.475904	0.424545
15.53	0.472694	0.475305	0.420742
15.54	0.470752	0.474613	0.4169
15.55	0.468689	0.473826	0.413016
15.56	0.466508	0.472943	0.40909

15.57	0.464213	0.471963	0.40512
15.58	0.46181	0.470884	0.401107
15.59	0.4593	0.469707	0.397048
15.6	0.45669	0.468429	0.392944
15.61	0.453981	0.46705	0.388794
15.62	0.451172	0.465565	0.384598
15.63	0.44826	0.463972	0.380358
15.64	0.445242	0.462265	0.376076
15.65	0.442116	0.460441	0.371752
15.66	0.438879	0.458496	0.36739
15.67	0.435527	0.456426	0.36299
15.68	0.432058	0.454227	0.358553
15.69	0.42847	0.451895	0.354081
15.7	0.424759	0.449426	0.349576
15.71	0.420924	0.446817	0.345037
15.72	0.416966	0.444069	0.340459
15.73	0.412889	0.441185	0.335833
15.74	0.408695	0.438165	0.331151
15.75	0.404388	0.435013	0.326407
15.76	0.399971	0.43173	0.321592
15.77	0.395447	0.428319	0.316698
15.78	0.390819	0.424782	0.311718
15.79	0.386089	0.42112	0.306643
15.8	0.381261	0.417336	0.301467
15.81	0.376339	0.413431	0.296182
15.82	0.371328	0.409406	0.290788
15.83	0.366234	0.405262	0.285282
15.84	0.361063	0.400998	0.279665
15.85	0.355821	0.396615	0.273936
15.86	0.350515	0.392113	0.268094
15.87	0.34515	0.387492	0.26214
15.88	0.339734	0.382753	0.256072
15.89	0.334271	0.377896	0.24989
15.9	0.328767	0.37292	0.243593
15.91	0.323229	0.367827	0.237182
15.92	0.317656	0.362619	0.230658
15.93	0.312046	0.357298	0.224022
15.94	0.306398	0.351867	0.217276
15.95	0.300711	0.346328	0.210423
15.96	0.294985	0.340683	0.203464
15.97	0.289217	0.334935	0.196401
15.98	0.283406	0.329087	0.189236
15.99	0.277551	0.323141	0.181972
16	0.271651	0.317099	0.174608
16.01	0.265706	0.310964	0.167149
16.02	0.259716	0.304739	0.159595
16.03	0.253685	0.298427	0.151949
16.04	0.247614	0.29203	0.144213

16.05	0.241506	0.285552	0.136389
16.06	0.235363	0.278996	0.128479
16.07	0.229187	0.272364	0.120484
16.08	0.222981	0.265659	0.112408
16.09	0.216746	0.258885	0.104252
16.1	0.210485	0.252044	0.096019
16.11	0.204201	0.245139	0.08771
16.12	0.197897	0.238169	0.079331
16.13	0.191574	0.231135	0.070888
16.14	0.185237	0.224034	0.062387
16.15	0.178888	0.216868	0.053833
16.16	0.172531	0.209635	0.045232
16.17	0.166167	0.202334	0.03659
16.18	0.159802	0.194965	0.027912
16.19	0.153436	0.187527	0.019205
16.2	0.147074	0.18002	0.010473
16.21	0.140719	0.172445	0.001724
16.22	0.134378	0.164806	-0.00704
16.23	0.12806	0.15711	-0.0158
16.24	0.121771	0.149365	-0.02457
16.25	0.11552	0.141577	-0.03332
16.26	0.109316	0.133752	-0.04206
16.27	0.103164	0.125897	-0.05077
16.28	0.097075	0.11802	-0.05945
16.29	0.091055	0.110126	-0.0681
16.3	0.085112	0.102222	-0.07671
16.31	0.079253	0.094315	-0.08526
16.32	0.07348	0.086406	-0.09376
16.33	0.067794	0.078496	-0.10219
16.34	0.062194	0.070587	-0.11056
16.35	0.056682	0.062679	-0.11886
16.36	0.051258	0.054774	-0.12708
16.37	0.045923	0.046872	-0.13522
16.38	0.040676	0.038976	-0.14328
16.39	0.03552	0.031085	-0.15124
16.4	0.030453	0.023202	-0.1591
16.41	0.025477	0.015328	-0.16686
16.42	0.020584	0.007469	-0.17451
16.43	0.015769	-0.00037	-0.18204
16.44	0.011026	-0.00817	-0.18945
16.45	0.006346	-0.01594	-0.19672
16.46	0.001725	-0.02366	-0.20384
16.47	-0.00285	-0.03134	-0.21082
16.48	-0.00737	-0.03895	-0.21764
16.49	-0.01186	-0.04649	-0.2243
16.5	-0.01631	-0.05397	-0.23078
16.51	-0.02074	-0.06136	-0.23708
16.52	-0.02515	-0.06866	-0.24319

16.53	-0.02955	-0.07587	-0.24912
16.54	-0.03393	-0.08298	-0.25487
16.55	-0.03831	-0.08998	-0.26042
16.56	-0.04268	-0.09686	-0.26577
16.57	-0.04705	-0.10362	-0.27093
16.58	-0.05143	-0.11026	-0.27589
16.59	-0.05582	-0.11676	-0.28065
16.6	-0.06022	-0.12312	-0.28521
16.61	-0.06465	-0.12933	-0.28957
16.62	-0.06909	-0.13539	-0.29372
16.63	-0.07357	-0.14131	-0.29768
16.64	-0.07807	-0.14708	-0.30145
16.65	-0.08261	-0.15271	-0.30505
16.66	-0.08719	-0.15821	-0.30846
16.67	-0.09181	-0.16357	-0.31171
16.68	-0.09648	-0.16879	-0.3148
16.69	-0.10119	-0.17387	-0.31773
16.7	-0.10597	-0.17883	-0.32051
16.71	-0.1108	-0.18365	-0.32315
16.72	-0.11568	-0.18835	-0.32564
16.73	-0.12062	-0.19291	-0.32799
16.74	-0.12562	-0.19735	-0.3302
16.75	-0.13066	-0.20165	-0.33226
16.76	-0.13574	-0.20583	-0.33419
16.77	-0.14087	-0.20988	-0.33597
16.78	-0.14605	-0.2138	-0.33761
16.79	-0.15126	-0.21759	-0.33912
16.8	-0.15651	-0.22125	-0.34048
16.81	-0.16179	-0.22478	-0.34171
16.82	-0.1671	-0.22819	-0.34281
16.83	-0.17244	-0.2315	-0.3438
16.84	-0.1778	-0.2347	-0.34469
16.85	-0.18319	-0.2378	-0.34549
16.86	-0.18858	-0.24082	-0.34622
16.87	-0.19399	-0.24376	-0.34689
16.88	-0.1994	-0.24663	-0.34751
16.89	-0.20482	-0.24944	-0.34809
16.9	-0.21024	-0.25219	-0.34866
16.91	-0.21565	-0.2549	-0.34921
16.92	-0.22105	-0.25757	-0.34975
16.93	-0.22644	-0.26021	-0.3503
16.94	-0.23182	-0.26282	-0.35085
16.95	-0.23718	-0.2654	-0.35141
16.96	-0.24252	-0.26797	-0.35198
16.97	-0.24784	-0.27052	-0.35257
16.98	-0.25313	-0.27306	-0.35318
16.99	-0.25838	-0.2756	-0.35382
17	-0.26361	-0.27815	-0.35449

17.01	-0.2688	-0.28069	-0.35519
17.02	-0.27395	-0.28326	-0.35593
17.03	-0.27905	-0.28584	-0.3567
17.04	-0.28412	-0.28844	-0.35751
17.05	-0.28914	-0.29107	-0.35835
17.06	-0.29412	-0.29374	-0.35923
17.07	-0.29905	-0.29644	-0.36014
17.08	-0.30394	-0.29919	-0.36109
17.09	-0.30877	-0.30199	-0.36207
17.1	-0.31355	-0.30485	-0.36308
17.11	-0.31827	-0.30776	-0.36414
17.12	-0.32295	-0.31073	-0.36523
17.13	-0.32756	-0.31376	-0.36637
17.14	-0.33213	-0.31684	-0.36756
17.15	-0.33663	-0.31997	-0.36881
17.16	-0.34109	-0.32315	-0.37011
17.17	-0.34548	-0.32638	-0.37148
17.18	-0.34982	-0.32964	-0.37293
17.19	-0.3541	-0.33295	-0.37444
17.2	-0.35833	-0.33629	-0.37604
17.21	-0.3625	-0.33967	-0.37773
17.22	-0.36661	-0.34307	-0.37949
17.23	-0.37066	-0.3465	-0.38132
17.24	-0.37466	-0.34996	-0.38321
17.25	-0.3786	-0.35343	-0.38516
17.26	-0.38249	-0.35691	-0.38716
17.27	-0.38632	-0.3604	-0.38919
17.28	-0.39009	-0.36389	-0.39126
17.29	-0.39382	-0.36739	-0.39334
17.3	-0.39748	-0.37088	-0.39545
17.31	-0.40109	-0.37435	-0.39756
17.32	-0.40465	-0.37782	-0.39967
17.33	-0.40816	-0.38128	-0.4018
17.34	-0.4116	-0.38472	-0.40393
17.35	-0.41499	-0.38815	-0.40607
17.36	-0.41833	-0.39155	-0.40821
17.37	-0.42161	-0.39494	-0.41036
17.38	-0.42483	-0.39831	-0.41251
17.39	-0.42799	-0.40165	-0.41467
17.4	-0.43109	-0.40497	-0.41684
17.41	-0.43413	-0.40826	-0.41901
17.42	-0.43711	-0.41152	-0.42118
17.43	-0.44003	-0.41474	-0.42335
17.44	-0.44289	-0.41791	-0.4255
17.45	-0.4457	-0.42103	-0.42765
17.46	-0.44844	-0.42408	-0.42977
17.47	-0.45112	-0.42706	-0.43187
17.48	-0.45374	-0.42997	-0.43395

17.49	-0.4563	-0.43279	-0.43598
17.5	-0.4588	-0.43552	-0.43798
17.51	-0.46124	-0.43815	-0.43994
17.52	-0.46361	-0.44068	-0.44185
17.53	-0.46592	-0.44312	-0.44371
17.54	-0.46817	-0.44547	-0.44552
17.55	-0.47036	-0.44773	-0.44728
17.56	-0.47247	-0.44991	-0.44897
17.57	-0.47452	-0.45201	-0.45061
17.58	-0.4765	-0.45402	-0.45218
17.59	-0.4784	-0.45596	-0.45368
17.6	-0.48024	-0.45783	-0.45511
17.61	-0.482	-0.45963	-0.45647
17.62	-0.48369	-0.46135	-0.45775
17.63	-0.4853	-0.46301	-0.45897
17.64	-0.48684	-0.46459	-0.46012
17.65	-0.48829	-0.46611	-0.4612
17.66	-0.48967	-0.46756	-0.46221
17.67	-0.49097	-0.46893	-0.46316
17.68	-0.49219	-0.47024	-0.46405
17.69	-0.49333	-0.47148	-0.46488
17.7	-0.49438	-0.47266	-0.46565
17.71	-0.49534	-0.47376	-0.46636
17.72	-0.49623	-0.4748	-0.46701
17.73	-0.49703	-0.47576	-0.4676
17.74	-0.49774	-0.47666	-0.46812
17.75	-0.49838	-0.47748	-0.46858
17.76	-0.49893	-0.47823	-0.46897
17.77	-0.4994	-0.4789	-0.46929
17.78	-0.4998	-0.4795	-0.46953
17.79	-0.50011	-0.48002	-0.4697
17.8	-0.50034	-0.48047	-0.46978
17.81	-0.5005	-0.48083	-0.46979
17.82	-0.50058	-0.48113	-0.46972
17.83	-0.50058	-0.48134	-0.46957
17.84	-0.50049	-0.48149	-0.46935
17.85	-0.50031	-0.48158	-0.46906
17.86	-0.50005	-0.48159	-0.46871
17.87	-0.4997	-0.48155	-0.46828
17.88	-0.49926	-0.48145	-0.4678
17.89	-0.49872	-0.4813	-0.46725
17.9	-0.49808	-0.48109	-0.46665
17.91	-0.49735	-0.48083	-0.466
17.92	-0.49652	-0.48053	-0.46528
17.93	-0.49559	-0.48017	-0.46451
17.94	-0.49457	-0.47976	-0.46366
17.95	-0.49345	-0.47929	-0.46274
17.96	-0.49223	-0.47877	-0.46173

17.97	-0.49093	-0.4782	-0.46065
17.98	-0.48952	-0.47757	-0.45947
17.99	-0.48803	-0.47688	-0.4582
18	-0.48644	-0.47613	-0.45684
18.01	-0.48476	-0.47533	-0.45536
18.02	-0.48299	-0.47446	-0.45378
18.03	-0.48113	-0.47353	-0.4521
18.04	-0.47917	-0.47254	-0.45031
18.05	-0.47712	-0.47149	-0.44841
18.06	-0.47498	-0.47037	-0.4464
18.07	-0.47275	-0.46918	-0.44429
18.08	-0.47042	-0.46793	-0.44206
18.09	-0.46799	-0.46661	-0.43973
18.1	-0.46548	-0.46523	-0.43728
18.11	-0.46287	-0.46377	-0.43472
18.12	-0.46016	-0.46224	-0.43205
18.13	-0.45736	-0.46064	-0.42926
18.14	-0.45446	-0.45897	-0.42636
18.15	-0.45145	-0.45722	-0.42333
18.16	-0.44835	-0.4554	-0.42019
18.17	-0.44515	-0.4535	-0.41693
18.18	-0.44185	-0.45152	-0.41354
18.19	-0.43844	-0.44946	-0.41003
18.2	-0.43493	-0.44732	-0.40639
18.21	-0.43132	-0.4451	-0.40262
18.22	-0.42759	-0.44279	-0.39873
18.23	-0.42377	-0.44039	-0.3947
18.24	-0.41983	-0.43791	-0.39054
18.25	-0.41579	-0.43534	-0.38624
18.26	-0.41165	-0.43267	-0.38181
18.27	-0.40739	-0.42991	-0.37724
18.28	-0.40303	-0.42705	-0.37253
18.29	-0.39856	-0.42409	-0.36767
18.3	-0.39398	-0.42104	-0.36268
18.31	-0.3893	-0.41788	-0.35754
18.32	-0.38452	-0.41461	-0.35225
18.33	-0.37964	-0.41124	-0.34683
18.34	-0.37468	-0.40777	-0.34127
18.35	-0.36965	-0.40417	-0.33558
18.36	-0.36455	-0.40047	-0.32976
18.37	-0.35939	-0.39665	-0.32382
18.38	-0.35418	-0.39271	-0.31775
18.39	-0.34893	-0.38866	-0.31156
18.4	-0.34365	-0.38448	-0.30525
18.41	-0.33833	-0.38018	-0.29883
18.42	-0.33299	-0.37575	-0.2923
18.43	-0.32762	-0.37121	-0.28565
18.44	-0.32221	-0.36655	-0.27888

18.45	-0.31677	-0.36177	-0.27199
18.46	-0.31128	-0.35688	-0.26498
18.47	-0.30575	-0.35188	-0.25786
18.48	-0.30018	-0.34677	-0.25061
18.49	-0.29455	-0.34155	-0.24324
18.5	-0.28887	-0.33623	-0.23574
18.51	-0.28314	-0.3308	-0.22812
18.52	-0.27735	-0.32528	-0.22038
18.53	-0.27151	-0.31965	-0.21253
18.54	-0.26563	-0.31391	-0.20458
18.55	-0.2597	-0.30806	-0.19652
18.56	-0.25372	-0.30211	-0.18837
18.57	-0.24771	-0.29605	-0.18014
18.58	-0.24166	-0.28987	-0.17183
18.59	-0.23557	-0.28359	-0.16345
18.6	-0.22946	-0.27719	-0.155
18.61	-0.22331	-0.27067	-0.14649
18.62	-0.21713	-0.26405	-0.13793
18.63	-0.21093	-0.25732	-0.12934
18.64	-0.2047	-0.25048	-0.12072
18.65	-0.19845	-0.24355	-0.11208
18.66	-0.19217	-0.23651	-0.10344
18.67	-0.18587	-0.22939	-0.0948
18.68	-0.17955	-0.22217	-0.08617
18.69	-0.17321	-0.21487	-0.07757
18.7	-0.16684	-0.20749	-0.06901
18.71	-0.16046	-0.20002	-0.06049
18.72	-0.15407	-0.19249	-0.05202
18.73	-0.14767	-0.18489	-0.04361
18.74	-0.14128	-0.17724	-0.03525
18.75	-0.13491	-0.16954	-0.02697
18.76	-0.12856	-0.16179	-0.01875
18.77	-0.12224	-0.15401	-0.01062
18.78	-0.11597	-0.14621	-0.00256
18.79	-0.10974	-0.13838	0.005413
18.8	-0.10358	-0.13054	0.013293
18.81	-0.09748	-0.12269	0.021077
18.82	-0.09145	-0.11484	0.028765
18.83	-0.08549	-0.10698	0.036353
18.84	-0.07962	-0.09911	0.043842
18.85	-0.07382	-0.09123	0.05123
18.86	-0.06811	-0.08334	0.058515
18.87	-0.06249	-0.07543	0.065697
18.88	-0.05695	-0.06752	0.072773
18.89	-0.05151	-0.05958	0.079744
18.9	-0.04617	-0.05164	0.086607
18.91	-0.04093	-0.04367	0.09336
18.92	-0.03579	-0.0357	0.099997

18.93	-0.03073	-0.02772	0.106511
18.94	-0.02576	-0.01975	0.112895
18.95	-0.02088	-0.0118	0.11914
18.96	-0.01607	-0.00386	0.12524
18.97	-0.01133	0.004051	0.131187
18.98	-0.00666	0.011927	0.136975
18.99	-0.00205	0.019762	0.142595
19	0.002501	0.027547	0.148041
19.01	0.007001	0.035276	0.153308
19.02	0.011455	0.04294	0.158401
19.03	0.015869	0.050533	0.163329
19.04	0.02025	0.058046	0.168101
19.05	0.024603	0.065472	0.172724
19.06	0.028935	0.072803	0.177208
19.07	0.033251	0.080031	0.18156
19.08	0.037559	0.087149	0.185789
19.09	0.041865	0.09415	0.189904
19.1	0.046173	0.101025	0.193913
19.11	0.050492	0.107768	0.197824
19.12	0.054827	0.11438	0.201644
19.13	0.059183	0.120863	0.20538
19.14	0.063567	0.127218	0.209039
19.15	0.067985	0.133448	0.212628
19.16	0.072443	0.139554	0.216153
19.17	0.076947	0.145538	0.219623
19.18	0.081503	0.151403	0.223042
19.19	0.086116	0.15715	0.226419
19.2	0.090794	0.162781	0.229761
19.21	0.095541	0.168297	0.233072
19.22	0.100354	0.173696	0.236352
19.23	0.105233	0.178973	0.239601
19.24	0.110172	0.184125	0.242817
19.25	0.115171	0.189149	0.245998
19.26	0.120225	0.194041	0.249143
19.27	0.125333	0.198797	0.25225
19.28	0.130491	0.203414	0.255317
19.29	0.135697	0.207888	0.258345
19.3	0.140947	0.212215	0.26133
19.31	0.146239	0.216393	0.264272
19.32	0.151569	0.220423	0.267177
19.33	0.156932	0.224307	0.270048
19.34	0.162324	0.228046	0.272891
19.35	0.167741	0.231644	0.275711
19.36	0.173178	0.235103	0.278514
19.37	0.178632	0.238423	0.281304
19.38	0.184098	0.241609	0.284087
19.39	0.189571	0.24466	0.286868
19.4	0.195048	0.247581	0.289652

19.41	0.200525	0.250374	0.292443
19.42	0.205996	0.253053	0.295246
19.43	0.211459	0.255631	0.298061
19.44	0.216909	0.258122	0.300891
19.45	0.222342	0.260543	0.303739
19.46	0.227755	0.262906	0.306606
19.47	0.233142	0.265226	0.309496
19.48	0.238501	0.267517	0.31241
19.49	0.243828	0.269794	0.315351
19.5	0.249117	0.272072	0.31832
19.51	0.254366	0.274362	0.32132
19.52	0.259573	0.276667	0.324347
19.53	0.264736	0.278988	0.327399
19.54	0.269855	0.281325	0.33047
19.55	0.274927	0.28368	0.333558
19.56	0.279951	0.286052	0.336659
19.57	0.284926	0.288443	0.339768
19.58	0.289851	0.290853	0.342883
19.59	0.294723	0.293282	0.346
19.6	0.299543	0.295732	0.349114
19.61	0.304307	0.298202	0.352224
19.62	0.309017	0.300694	0.355328
19.63	0.313672	0.303209	0.358426
19.64	0.318273	0.305748	0.361519
19.65	0.322818	0.308312	0.364607
19.66	0.327309	0.310902	0.36769
19.67	0.331745	0.313518	0.370769
19.68	0.336126	0.316163	0.373843
19.69	0.340452	0.318836	0.376912
19.7	0.344723	0.321539	0.379977
19.71	0.348939	0.324272	0.383039
19.72	0.3531	0.327039	0.386101
19.73	0.357206	0.329841	0.389169
19.74	0.361257	0.33268	0.392247
19.75	0.365253	0.335558	0.39534
19.76	0.369193	0.338478	0.398452
19.77	0.373079	0.341441	0.401589
19.78	0.376909	0.34445	0.404756
19.79	0.380684	0.347506	0.407956
19.8	0.384404	0.350613	0.411195
19.81	0.388068	0.353769	0.414475
19.82	0.391677	0.356967	0.417792
19.83	0.39523	0.360196	0.421139
19.84	0.398726	0.363447	0.424508
19.85	0.402166	0.366707	0.427892
19.86	0.40555	0.369968	0.431284
19.87	0.408876	0.373218	0.434678
19.88	0.412146	0.376448	0.438066

19.89	0.415358	0.379647	0.441442
19.9	0.418512	0.382804	0.444797
19.91	0.421608	0.385911	0.448126
19.92	0.424645	0.388966	0.451419
19.93	0.427622	0.391969	0.454669
19.94	0.430539	0.39492	0.457867
19.95	0.433393	0.397818	0.461006
19.96	0.436184	0.400664	0.464076
19.97	0.438912	0.403457	0.467071
19.98	0.441574	0.406197	0.46998
19.99	0.444171	0.408884	0.472797
20	0.4467	0.411519	0.475513
20.01	0.449162	0.4141	0.478121
20.02	0.451555	0.416629	0.480616
20.03	0.453879	0.419108	0.482996
20.04	0.456133	0.421538	0.485258
20.05	0.458316	0.423921	0.4874
20.06	0.460427	0.426258	0.489417
20.07	0.462466	0.428551	0.491307
20.08	0.464432	0.4308	0.493068
20.09	0.466324	0.433009	0.494696
20.1	0.468142	0.435178	0.496188
20.11	0.469884	0.437307	0.497542
20.12	0.47155	0.439396	0.498752
20.13	0.47314	0.441439	0.499817
20.14	0.474652	0.443435	0.500731
20.15	0.476086	0.445379	0.501491
20.16	0.47744	0.447269	0.502092
20.17	0.478715	0.449102	0.502531
20.18	0.479909	0.450874	0.502804
20.19	0.481021	0.452582	0.502907
20.2	0.482052	0.454223	0.502836
20.21	0.482999	0.455795	0.502587
20.22	0.483864	0.457299	0.50216
20.23	0.484646	0.458737	0.501553
20.24	0.485346	0.460113	0.500767
20.25	0.485963	0.461428	0.499801
20.26	0.486499	0.462685	0.498653
20.27	0.486952	0.463887	0.497325
20.28	0.487324	0.465036	0.495814
20.29	0.487614	0.466134	0.494121
20.3	0.487822	0.467184	0.492244
20.31	0.48795	0.468187	0.490186
20.32	0.487996	0.469142	0.487956
20.33	0.487963	0.470046	0.485566
20.34	0.48785	0.470896	0.48303
20.35	0.487657	0.47169	0.480358
20.36	0.487386	0.472424	0.477563

20.37	0.487036	0.473096	0.474657
20.38	0.486608	0.473704	0.471652
20.39	0.486103	0.474244	0.46856
20.4	0.485522	0.474714	0.465394
20.41	0.484864	0.475111	0.462164
20.42	0.484132	0.475435	0.458874
20.43	0.48333	0.475683	0.455528
20.44	0.482459	0.475856	0.452127
20.45	0.481522	0.475952	0.448674
20.46	0.480522	0.47597	0.445173
20.47	0.479462	0.475909	0.441626
20.48	0.478345	0.475768	0.438036
20.49	0.477172	0.475546	0.434405
20.5	0.475947	0.475242	0.430736
20.51	0.474674	0.474855	0.427032
20.52	0.473361	0.474387	0.423296
20.53	0.472019	0.473837	0.419528
20.54	0.470658	0.473206	0.415732
20.55	0.469288	0.472496	0.411909
20.56	0.46792	0.471706	0.408062
20.57	0.466564	0.470839	0.404192
20.58	0.465231	0.469894	0.400302
20.59	0.463929	0.468873	0.396394
20.6	0.46267	0.467776	0.39247
20.61	0.461462	0.466604	0.388532
20.62	0.460303	0.465359	0.384588
20.63	0.459192	0.464042	0.380643
20.64	0.458125	0.462655	0.376704
20.65	0.457099	0.4612	0.372779
20.66	0.456112	0.459677	0.368874
20.67	0.45516	0.45809	0.364995
20.68	0.454242	0.456439	0.36115
20.69	0.453352	0.454726	0.357346
20.7	0.45249	0.452952	0.353588
20.71	0.451653	0.45112	0.349883
20.72	0.450841	0.449231	0.346223
20.73	0.450056	0.447286	0.342603
20.74	0.449301	0.445287	0.339013
20.75	0.448575	0.443235	0.335448
20.76	0.447881	0.441133	0.331899
20.77	0.44722	0.438981	0.328358
20.78	0.446594	0.436781	0.324819
20.79	0.446005	0.434535	0.321273
20.8	0.445453	0.432244	0.317713
20.81	0.44494	0.429913	0.314136
20.82	0.444464	0.427553	0.310556
20.83	0.44402	0.425181	0.306991
20.84	0.443607	0.42281	0.303461

20.85	0.443221	0.420457	0.299984
20.86	0.442859	0.418137	0.296578
20.87	0.442518	0.415864	0.293262
20.88	0.442195	0.413655	0.290055
20.89	0.441887	0.411523	0.286974
20.9	0.44159	0.409484	0.28404
20.91	0.441302	0.407553	0.28127
20.92	0.441019	0.405746	0.278683
20.93	0.440738	0.404077	0.276298
20.94	0.440457	0.402561	0.274133
20.95	0.440173	0.401215	0.272206
20.96	0.439881	0.400052	0.270537
20.97	0.439579	0.399088	0.269144
20.98	0.439265	0.398338	0.268046
20.99	0.438934	0.397818	0.26726
21	0.438584	0.397542	0.266807

Table S13. Slalom Test Result Data

Time	Actual Value	Estimated Value
0	-3.48E-08	0.007897233
0.01	-0.004743223	0.008898165
0.02	-0.008295412	0.010043381
0.03	-0.010733371	0.01132833
0.04	-0.012133864	0.012748462
0.05	-0.01257366	0.014299224
0.06	-0.012129524	0.015976066
0.07	-0.010878224	0.017774436
0.08	-0.008896525	0.019689784
0.09	-0.006261196	0.021717558
0.1	-0.003049003	0.023853208
0.11	0.000663288	0.026092181
0.12	0.00479891	0.028429928
0.13	0.009281095	0.030861896
0.14	0.014033079	0.033383535
0.15	0.018978092	0.035990294
0.16	0.02403937	0.038677621
0.17	0.029140144	0.041440966
0.18	0.034203649	0.044275776
0.19	0.039153118	0.047177502
0.2	0.043911783	0.050141592
0.21	0.048413622	0.05315751
0.22	0.052635584	0.056190783

0.23	0.056565362	0.059200954
0.24	0.060190649	0.062147563
0.25	0.063499139	0.064990154
0.26	0.066478524	0.067688269
0.27	0.069116497	0.070201449
0.28	0.071400751	0.072489238
0.29	0.073318981	0.074511176
0.3	0.074858877	0.076226807
0.31	0.076012623	0.07760582
0.32	0.076790359	0.078658495
0.33	0.077206714	0.07940526
0.34	0.077276316	0.079866543
0.35	0.077013796	0.080062773
0.36	0.076433781	0.080014378
0.37	0.075550901	0.079741784
0.38	0.074379786	0.07926542
0.39	0.072935064	0.078605715
0.4	0.071231364	0.077783096
0.41	0.06928849	0.076817637
0.42	0.067146944	0.075727992
0.43	0.064852401	0.074532463
0.44	0.062450538	0.073249351
0.45	0.05998703	0.071896956
0.46	0.057507553	0.070493579
0.47	0.055057784	0.069057521
0.48	0.052683398	0.067607082
0.49	0.050430071	0.066160564
0.5	0.048343479	0.064736267
0.51	0.046460308	0.063350227
0.52	0.044781285	0.062009417
0.53	0.043298144	0.060718547
0.54	0.042002621	0.059482324
0.55	0.040886453	0.058305458
0.56	0.039941374	0.057192657
0.57	0.039159121	0.056148631
0.58	0.038531429	0.055178086
0.59	0.038050034	0.054285733
0.6	0.037706672	0.053476279
0.61	0.037492199	0.052754702
0.62	0.037393954	0.052127051
0.63	0.037398397	0.051599642
0.64	0.037491988	0.051178793
0.65	0.037661186	0.050870821
0.66	0.037892451	0.050682043
0.67	0.038172243	0.050618776
0.68	0.038487021	0.050687337
0.69	0.038823246	0.050894044
0.7	0.039167376	0.051245213

0.71	0.039507798	0.051741382
0.72	0.039840598	0.052359972
0.73	0.040163792	0.053072625
0.74	0.040475392	0.05385098
0.75	0.040773412	0.05466668
0.76	0.041055865	0.055491366
0.77	0.041320765	0.056296679
0.78	0.041566127	0.05705426
0.79	0.041789962	0.057735751
0.8	0.041990285	0.058312792
0.81	0.042164507	0.058762379
0.82	0.042307628	0.059082917
0.83	0.042414047	0.059278168
0.84	0.042478162	0.05935189
0.85	0.042494371	0.059307844
0.86	0.042457071	0.05914979
0.87	0.042360661	0.058881487
0.88	0.042199539	0.058506696
0.89	0.041968104	0.058029177
0.9	0.041660752	0.057452689
0.91	0.041273271	0.056779802
0.92	0.040807001	0.056008325
0.93	0.04026467	0.055134874
0.94	0.039649006	0.054156067
0.95	0.038962738	0.053068523
0.96	0.038208595	0.051868859
0.97	0.037389304	0.050553691
0.98	0.036507594	0.049119639
0.99	0.035566194	0.04756332
1	0.034567831	0.045881351
1.01	0.033518544	0.044075207
1.02	0.032437607	0.042165789
1.03	0.031347604	0.040178856
1.04	0.03027112	0.038140167
1.05	0.029230737	0.036075478
1.06	0.02824904	0.034010549
1.07	0.027348613	0.031971139
1.08	0.026552041	0.029983004
1.09	0.025881905	0.028071905
1.1	0.025360792	0.026263598
1.11	0.025004534	0.024579858
1.12	0.024801968	0.02302652
1.13	0.024735177	0.021605433
1.14	0.024786247	0.020318447
1.15	0.024937262	0.019167413
1.16	0.025170309	0.018154181
1.17	0.025467471	0.017280601
1.18	0.025810835	0.016548522

1.19	0.026182484	0.015959795
1.2	0.026564505	0.01551627
1.21	0.026941768	0.015217068
1.22	0.027310293	0.015050395
1.23	0.027668885	0.015001727
1.24	0.02801635	0.01505654
1.25	0.028351492	0.015200313
1.26	0.028673117	0.015418521
1.27	0.028980031	0.015696641
1.28	0.029271039	0.016020149
1.29	0.029544947	0.016374523
1.3	0.029800559	0.016745238
1.31	0.03003695	0.017119781
1.32	0.030254263	0.017493669
1.33	0.030452912	0.01786443
1.34	0.03063331	0.01822959
1.35	0.030795868	0.018586676
1.36	0.030941	0.018933216
1.37	0.031069118	0.019266736
1.38	0.031180635	0.019584764
1.39	0.031275963	0.019884826
1.4	0.031355516	0.020164448
1.41	0.031419102	0.020421473
1.42	0.031464115	0.020654997
1.43	0.031487345	0.020864431
1.44	0.031485585	0.021049187
1.45	0.031455622	0.021208675
1.46	0.03139425	0.021342306
1.47	0.031298257	0.021449492
1.48	0.031164434	0.021529643
1.49	0.030989572	0.021582172
1.5	0.030770461	0.021606487
1.51	0.030506478	0.0216033
1.52	0.030207342	0.021578511
1.53	0.029885358	0.02153932
1.54	0.029552832	0.021492927
1.55	0.029222069	0.021446531
1.56	0.028905374	0.021407331
1.57	0.028615052	0.021382528
1.58	0.028363409	0.02137932
1.59	0.02816275	0.021404909
1.6	0.028025381	0.021466492
1.61	0.027959944	0.021569731
1.62	0.027960436	0.021714129
1.63	0.028017189	0.021897654
1.64	0.028120539	0.022118269
1.65	0.028260817	0.022373941
1.66	0.028428359	0.022662635

1.67	0.028613498	0.022982317
1.68	0.028806568	0.023330951
1.69	0.028997901	0.023706504
1.7	0.029177833	0.02410694
1.71	0.029339382	0.024530336
1.72	0.029486309	0.02497521
1.73	0.029625061	0.025440187
1.74	0.029762084	0.025923897
1.75	0.029903825	0.026424967
1.76	0.030056731	0.026942025
1.77	0.030227247	0.027473697
1.78	0.030421821	0.028018613
1.79	0.030646899	0.028575399
1.8	0.030908927	0.029142683
1.81	0.031216346	0.029719563
1.82	0.031585567	0.030307023
1.83	0.032034998	0.030906514
1.84	0.032583044	0.031519491
1.85	0.033248112	0.032147405
1.86	0.034048606	0.032791711
1.87	0.035002934	0.033453861
1.88	0.036129501	0.034135308
1.89	0.037446713	0.034837506
1.9	0.038972977	0.035561906
1.91	0.040715554	0.036310067
1.92	0.042637132	0.037083956
1.93	0.044689252	0.037885647
1.94	0.046823457	0.03871721
1.95	0.048991291	0.039580719
1.96	0.051144296	0.040478246
1.97	0.053234014	0.041411863
1.98	0.055211989	0.042383643
1.99	0.057029762	0.043395657
2	0.058638877	0.044449978
2.01	0.059998266	0.045547383
2.02	0.061096422	0.046683464
2.03	0.061929226	0.047852519
2.04	0.06249256	0.049048847
2.05	0.062782307	0.050266743
2.06	0.062794348	0.051500507
2.07	0.062524566	0.052744435
2.08	0.061968842	0.053992825
2.09	0.061123059	0.055239974
2.1	0.059983098	0.056480181
2.11	0.058553164	0.057707275
2.12	0.056870751	0.058913221
2.13	0.054981675	0.060089515
2.14	0.052931752	0.061227655

2.15	0.050766799	0.062319136
2.16	0.048532631	0.063355456
2.17	0.046275066	0.064328111
2.18	0.04403992	0.065228599
2.19	0.041873008	0.066048416
2.2	0.039820147	0.066779059
2.21	0.037919266	0.067413911
2.22	0.036176745	0.0679539
2.23	0.034591076	0.068401839
2.24	0.033160752	0.068760542
2.25	0.031884266	0.069032822
2.26	0.030760109	0.069221494
2.27	0.029786774	0.06932937
2.28	0.028962754	0.069359265
2.29	0.028286542	0.069313992
2.3	0.027756629	0.069196366
2.31	0.027369603	0.069009037
2.32	0.027114429	0.068754018
2.33	0.026978165	0.068433157
2.34	0.026947872	0.068048304
2.35	0.027010608	0.067601309
2.36	0.027153432	0.067094019
2.37	0.027363405	0.066528286
2.38	0.027627584	0.065905958
2.39	0.02793303	0.065228885
2.4	0.028266802	0.064498917
2.41	0.028616208	0.063717625
2.42	0.02896955	0.062885479
2.43	0.029315383	0.062002669
2.44	0.029642257	0.061069388
2.45	0.029938726	0.060085826
2.46	0.030193342	0.059052175
2.47	0.030394656	0.057968627
2.48	0.030531222	0.056835372
2.49	0.030591592	0.055652603
2.5	0.030564317	0.054420512
2.51	0.030440223	0.053139446
2.52	0.030219227	0.05181039
2.53	0.029903515	0.050434482
2.54	0.029495276	0.049012864
2.55	0.028996698	0.047546676
2.56	0.02840997	0.046037058
2.57	0.02773728	0.044485151
2.58	0.026980815	0.042892094
2.59	0.026142765	0.041259028
2.6	0.025225318	0.039587094
2.61	0.02423257	0.037878302
2.62	0.023176256	0.036138144

2.63	0.022070017	0.03437298
2.64	0.020927496	0.032589172
2.65	0.019762335	0.030793082
2.66	0.018588177	0.028991072
2.67	0.017418663	0.027189502
2.68	0.016267437	0.025394734
2.69	0.01514814	0.023613131
2.7	0.014074415	0.021851052
2.71	0.013059704	0.020115295
2.72	0.01211665	0.018414393
2.73	0.011257694	0.016757315
2.74	0.010495278	0.01515303
2.75	0.009841844	0.013610506
2.76	0.009309834	0.012138712
2.77	0.00891169	0.010746616
2.78	0.008659854	0.009443188
2.79	0.008566769	0.008237395
2.8	0.008644875	0.007138207
2.81	0.008899961	0.006152349
2.82	0.009311197	0.005277576
2.83	0.009851101	0.004509396
2.84	0.010492187	0.003843322
2.85	0.011206973	0.003274865
2.86	0.011967975	0.002799534
2.87	0.012747709	0.002412841
2.88	0.013518692	0.002110296
2.89	0.01425344	0.001887411
2.9	0.01492447	0.001739696
2.91	0.015511287	0.001662576
2.92	0.016021351	0.001651135
2.93	0.016469114	0.001700372
2.94	0.016869024	0.001805287
2.95	0.017235533	0.001960877
2.96	0.017583089	0.002162141
2.97	0.017926144	0.002404079
2.98	0.018279147	0.002681688
2.99	0.018656547	0.002989968
3	0.019072796	0.003323917
3.01	0.019537568	0.003678857
3.02	0.020041435	0.004051399
3.03	0.020570198	0.004438476
3.04	0.021109653	0.004837023
3.05	0.021645598	0.005243974
3.06	0.022163833	0.005656263
3.07	0.022650155	0.006070823
3.08	0.023090363	0.006484588
3.09	0.023470254	0.006894494
3.1	0.023775627	0.007297472

3.11	0.023995034	0.007691016
3.12	0.024128044	0.008074846
3.13	0.024176978	0.008449244
3.14	0.02414416	0.008814489
3.15	0.024031913	0.009170861
3.16	0.023842557	0.00951864
3.17	0.023578417	0.009858106
3.18	0.023241815	0.010189539
3.19	0.022835073	0.010513218
3.2	0.022360513	0.010829425
3.21	0.021822802	0.011139214
3.22	0.02123598	0.011446751
3.23	0.020616432	0.011756975
3.24	0.01998054	0.012074828
3.25	0.019344689	0.012405248
3.26	0.018725264	0.012753178
3.27	0.018138647	0.013123557
3.28	0.017601224	0.013521326
3.29	0.017129377	0.013951425
3.3	0.016739491	0.014418795
3.31	0.016440484	0.014926409
3.32	0.016211407	0.01546938
3.33	0.016023846	0.01604085
3.34	0.015849388	0.016633964
3.35	0.015659617	0.017241866
3.36	0.01542612	0.017857701
3.37	0.015120483	0.018474613
3.38	0.01471429	0.019085746
3.39	0.014179128	0.019684245
3.4	0.013486583	0.020263253
3.41	0.012612738	0.020815825
3.42	0.011551671	0.021334646
3.43	0.010301955	0.021812314
3.44	0.008862165	0.022241424
3.45	0.007230877	0.022614572
3.46	0.005406665	0.022924355
3.47	0.003388103	0.023163369
3.48	0.001173767	0.023324209
3.49	-0.00123777	0.023399471
3.5	-0.003847931	0.023381752
3.51	-0.006653329	0.023264257
3.52	-0.009631322	0.023042623
3.53	-0.012754453	0.022713097
3.54	-0.015995265	0.022271925
3.55	-0.019326303	0.021715355
3.56	-0.022720111	0.021039634
3.57	-0.026149232	0.020241007
3.58	-0.029586209	0.019315721

3.59	-0.033003588	0.018260024
3.6	-0.036373911	0.017070162
3.61	-0.039669547	0.015744571
3.62	-0.042862161	0.01429045
3.63	-0.045923245	0.012717185
3.64	-0.048824289	0.011034164
3.65	-0.051536782	0.009250775
3.66	-0.054032215	0.007376406
3.67	-0.05628208	0.005420443
3.68	-0.058257865	0.003392274
3.69	-0.059931062	0.001301287
3.7	-0.061273161	-0.000802981
3.71	-0.062264179	-0.002887307
3.72	-0.062918241	-0.005005103
3.73	-0.063257998	-0.007147896
3.74	-0.063306101	-0.00930721
3.75	-0.063085202	-0.01147457
3.76	-0.062617951	-0.013641502
3.77	-0.061927002	-0.01579953
3.78	-0.061035004	-0.017940181
3.79	-0.059964609	-0.020054978
3.8	-0.058738468	-0.022135447
3.81	-0.057380208	-0.024174357
3.82	-0.055917354	-0.026169447
3.83	-0.054378405	-0.028119701
3.84	-0.052791863	-0.030024103
3.85	-0.051186227	-0.031881636
3.86	-0.049589996	-0.033691284
3.87	-0.048031673	-0.035452029
3.88	-0.046539755	-0.037162855
3.89	-0.045142744	-0.038822746
3.9	-0.043869139	-0.040430685
3.91	-0.042738744	-0.041985317
3.92	-0.041736577	-0.043483926
3.93	-0.040838957	-0.04492346
3.94	-0.040022205	-0.046300864
3.95	-0.039262642	-0.047613086
3.96	-0.038536589	-0.048857072
3.97	-0.037820365	-0.050029768
3.98	-0.037090292	-0.051128121
3.99	-0.03632269	-0.052149078
4	-0.035493879	-0.053089584
4.01	-0.03458507	-0.053946524
4.02	-0.033597033	-0.054716539
4.03	-0.032535426	-0.055396208
4.04	-0.03140591	-0.055982107
4.05	-0.030214142	-0.056470816
4.06	-0.028965783	-0.056858913

4.07	-0.027666491	-0.057142977
4.08	-0.026321927	-0.057319586
4.09	-0.024937749	-0.057385318
4.1	-0.023519616	-0.057336751
4.11	-0.022072267	-0.057169797
4.12	-0.020596754	-0.056877696
4.13	-0.01909321	-0.056453022
4.14	-0.017561766	-0.055888347
4.15	-0.016002554	-0.055176246
4.16	-0.014415706	-0.054309291
4.17	-0.012801354	-0.053280055
4.18	-0.011159629	-0.052081112
4.19	-0.009490664	-0.050705034
4.2	-0.00779459	-0.049144396
4.21	-0.00607312	-0.047394674
4.22	-0.00433429	-0.045462956
4.23	-0.002587716	-0.043359236
4.24	-0.000843016	-0.041093505
4.25	0.000890194	-0.038675755
4.26	0.002602297	-0.03611598
4.27	0.004283677	-0.033424171
4.28	0.005924717	-0.030610321
4.29	0.007515799	-0.027684422
4.3	0.009047308	-0.024656466
4.31	0.010512299	-0.021536033
4.32	0.011914522	-0.018331051
4.33	0.013260398	-0.015049033
4.34	0.014556348	-0.011697494
4.35	0.015808795	-0.008283948
4.36	0.017024161	-0.004815911
4.37	0.018208867	-0.001300896
4.38	0.019369335	0.002366262
4.39	0.020511986	0.00613201
4.4	0.021643243	0.009923415
4.41	0.022765778	0.013731607
4.42	0.023867266	0.017543802
4.43	0.024931632	0.021346238
4.44	0.025942801	0.025125153
4.45	0.0268847	0.028866784
4.46	0.027741254	0.032557368
4.47	0.028496388	0.036183144
4.48	0.029134028	0.039730349
4.49	0.029638098	0.043185221
4.5	0.029992526	0.046533996
4.51	0.030190397	0.049765306
4.52	0.030261444	0.052877351
4.53	0.030244561	0.055870725
4.54	0.030178642	0.058746021

4.55	0.03010258	0.061503833
4.56	0.030055269	0.064144754
4.57	0.030075604	0.066669379
4.58	0.030202477	0.0690783
4.59	0.030474783	0.07137211
4.6	0.030931416	0.073551405
4.61	0.03160361	0.075617965
4.62	0.032491966	0.077578323
4.63	0.033589423	0.0794402
4.64	0.034888923	0.081211318
4.65	0.036383407	0.082899397
4.66	0.038065814	0.084512159
4.67	0.039929087	0.086057324
4.68	0.041966165	0.087542614
4.69	0.044169989	0.08897575
4.7	0.0465335	0.090364453
4.71	0.049049104	0.091716738
4.72	0.051707065	0.093041804
4.73	0.054497114	0.094349141
4.74	0.05740898	0.095648242
4.75	0.060432393	0.0969486
4.76	0.063557083	0.098259705
4.77	0.066772779	0.09959105
4.78	0.070069213	0.100952128
4.79	0.073436112	0.102352429
4.8	0.076863208	0.103801446
4.81	0.080338652	0.105306522
4.82	0.083844287	0.106866399
4.83	0.087360377	0.108477671
4.84	0.090867186	0.110136932
4.85	0.094344979	0.111840773
4.86	0.09777402	0.11358579
4.87	0.101134574	0.115368576
4.88	0.104406905	0.117185723
4.89	0.107571277	0.119033825
4.9	0.110607956	0.120909476
4.91	0.113500974	0.122809161
4.92	0.116249442	0.124728941
4.93	0.118856239	0.126664767
4.94	0.121324244	0.128612591
4.95	0.123656337	0.130568365
4.96	0.125855396	0.132528042
4.97	0.127924301	0.134487573
4.98	0.129865932	0.136442911
4.99	0.131683166	0.138390007
5	0.133378885	0.140324813
5.01	0.134958514	0.142243887
5.02	0.136437671	0.144146202

5.03	0.137834519	0.146031335
5.04	0.139167225	0.147898867
5.05	0.140453953	0.149748374
5.06	0.141712866	0.151579436
5.07	0.142962131	0.15339163
5.08	0.144219912	0.155184534
5.09	0.145504372	0.156957728
5.1	0.146833678	0.158710789
5.11	0.148222608	0.160444171
5.12	0.149672402	0.162161827
5.13	0.151180914	0.163868585
5.14	0.152745998	0.165569275
5.15	0.154365508	0.167268725
5.16	0.156037297	0.168971764
5.17	0.157759221	0.17068322
5.18	0.159529133	0.172407922
5.19	0.161344887	0.174150698
5.2	0.163204337	0.175916378
5.21	0.165108335	0.177708511
5.22	0.167069724	0.179525541
5.23	0.169104344	0.18136463
5.24	0.171228036	0.183222943
5.25	0.173456639	0.185097645
5.26	0.175805995	0.186985899
5.27	0.178291944	0.188884869
5.28	0.180930326	0.190791719
5.29	0.183736981	0.192703614
5.3	0.186727751	0.194617718
5.31	0.189913568	0.196531244
5.32	0.193285736	0.198441606
5.33	0.196830652	0.200346266
5.34	0.200534714	0.202242687
5.35	0.204384317	0.204128332
5.36	0.20836586	0.206000664
5.37	0.212465738	0.207857145
5.38	0.216670348	0.209695238
5.39	0.220966088	0.211512406
5.4	0.225339354	0.213306111
5.41	0.229771608	0.215074695
5.42	0.23422457	0.21682001
5.43	0.238655026	0.218544784
5.44	0.243019759	0.220251749
5.45	0.247275556	0.221943634
5.46	0.251379201	0.22362317
5.47	0.255287479	0.225293085
5.48	0.258957176	0.226956111
5.49	0.262345076	0.228614977
5.5	0.265407964	0.230272412

5.51	0.26811224	0.231930636
5.52	0.270462762	0.233589815
5.53	0.272474001	0.235249605
5.54	0.274160431	0.236909661
5.55	0.275536522	0.23856964
5.56	0.276616749	0.240229196
5.57	0.277415581	0.241887985
5.58	0.277947493	0.243545663
5.59	0.278226955	0.245201885
5.6	0.278268441	0.246856306
5.61	0.278088017	0.248508269
5.62	0.277708128	0.250155866
5.63	0.277152815	0.251796874
5.64	0.276446118	0.253429073
5.65	0.275612077	0.255050239
5.66	0.274674732	0.256658152
5.67	0.273658123	0.25825059
5.68	0.27258629	0.259825331
5.69	0.271483273	0.261380154
5.7	0.270373113	0.262912836
5.71	0.269274918	0.264419995
5.72	0.268188073	0.265893604
5.73	0.267107031	0.267324475
5.74	0.266026245	0.268703418
5.75	0.264940168	0.270021246
5.76	0.263843254	0.271268771
5.77	0.262729955	0.272436804
5.78	0.261594726	0.273516156
5.79	0.260432018	0.274497641
5.8	0.259236285	0.275372068
5.81	0.258000679	0.276131305
5.82	0.256713144	0.276771435
5.83	0.255360323	0.277289598
5.84	0.253928859	0.277682933
5.85	0.252405394	0.277948578
5.86	0.250776571	0.278083672
5.87	0.249029034	0.278085354
5.88	0.247149425	0.277950763
5.89	0.245124386	0.277677038
5.9	0.242940561	0.277261318
5.91	0.240589228	0.276700812
5.92	0.238080207	0.275993015
5.93	0.235427955	0.275135493
5.94	0.232646927	0.274125811
5.95	0.229751579	0.272961534
5.96	0.226756368	0.271640229
5.97	0.22367575	0.270159461
5.98	0.220524181	0.268516794

5.99	0.217316116	0.266709796
6	0.214066012	0.264736031
6.01	0.210785926	0.262594306
6.02	0.207478316	0.260288397
6.03	0.204143243	0.257823322
6.04	0.200780766	0.255204095
6.05	0.197390946	0.252435736
6.06	0.193973842	0.24952326
6.07	0.190529514	0.246471684
6.08	0.187058022	0.243286026
6.09	0.183559427	0.239971302
6.1	0.180033787	0.23653253
6.11	0.176478294	0.232973135
6.12	0.17287866	0.229290176
6.13	0.16921773	0.225479121
6.14	0.165478348	0.221535439
6.15	0.161643358	0.217454597
6.16	0.157695603	0.213232064
6.17	0.153617927	0.208863308
6.18	0.149393176	0.204343796
6.19	0.145004192	0.199668997
6.2	0.140433819	0.194834379
6.21	0.135667745	0.189836886
6.22	0.130703027	0.184679367
6.23	0.125539566	0.179366148
6.24	0.120177263	0.173901554
6.25	0.114616018	0.16828991
6.26	0.108855733	0.162535542
6.27	0.102896308	0.156642775
6.28	0.096737644	0.150615935
6.29	0.090379642	0.144459348
6.3	0.083822202	0.138177337
6.31	0.077071197	0.131774489
6.32	0.070156389	0.125256427
6.33	0.06311351	0.118629033
6.34	0.055978293	0.111898189
6.35	0.04878647	0.105069778
6.36	0.041573776	0.098149684
6.37	0.034375943	0.091143787
6.38	0.027228704	0.084057971
6.39	0.020167791	0.076898119
6.4	0.013228938	0.069670113
6.41	0.006445555	0.062380942
6.42	-0.00015824	0.055042025
6.43	-0.006560652	0.047665887
6.44	-0.012739885	0.040265052
6.45	-0.018674144	0.032852046
6.46	-0.024341634	0.025439394

6.47	-0.029720559	0.01803962
6.48	-0.034789124	0.010665251
6.49	-0.039525534	0.00332881
6.5	-0.043907993	-0.00376874
6.51	-0.047928245	-0.01064797
6.52	-0.051632186	-0.01745634
6.53	-0.055079254	-0.024182969
6.54	-0.058328885	-0.030816976
6.55	-0.061440513	-0.037347481
6.56	-0.064473576	-0.043763604
6.57	-0.06748751	-0.050054464
6.58	-0.070541751	-0.056209181
6.59	-0.073695735	-0.062216873
6.6	-0.077008897	-0.068066661
6.61	-0.080526218	-0.073750525
6.62	-0.084234852	-0.079271883
6.63	-0.088107496	-0.084637016
6.64	-0.092116849	-0.089852205
6.65	-0.096235607	-0.09492373
6.66	-0.100436468	-0.099857869
6.67	-0.10469213	-0.104660905
6.68	-0.108975291	-0.109339116
6.69	-0.113258648	-0.113898783
6.7	-0.117514899	-0.118346185
6.71	-0.121723384	-0.12268656
6.72	-0.125890013	-0.126920967
6.73	-0.130027337	-0.131049424
6.74	-0.13414791	-0.135071947
6.75	-0.138264283	-0.138988553
6.76	-0.142389008	-0.142799258
6.77	-0.146534639	-0.146504079
6.78	-0.150713726	-0.150103033
6.79	-0.154938822	-0.153596137
6.8	-0.15922248	-0.156983406
6.81	-0.163566756	-0.160265448
6.82	-0.167931727	-0.163445218
6.83	-0.172266974	-0.166526265
6.84	-0.176522079	-0.169512134
6.85	-0.180646622	-0.172406371
6.86	-0.184590185	-0.175212524
6.87	-0.188302349	-0.177934139
6.88	-0.191732696	-0.180574762
6.89	-0.194830806	-0.18313794
6.9	-0.197546262	-0.185627219
6.91	-0.199840854	-0.188044472
6.92	-0.201725216	-0.190384881
6.93	-0.20322219	-0.192641954
6.94	-0.204354619	-0.194809198

6.95	-0.205145346	-0.196880123
6.96	-0.205617214	-0.198848234
6.97	-0.205793066	-0.200707042
6.98	-0.205695746	-0.202450054
6.99	-0.205348095	-0.204070777
7	-0.204772958	-0.20556272
7.01	-0.203994989	-0.206920983
7.02	-0.203046096	-0.208147027
7.03	-0.201959999	-0.209243908
7.04	-0.200770417	-0.210214681
7.05	-0.19951107	-0.211062399
7.06	-0.198215678	-0.211790117
7.07	-0.196917961	-0.21240089
7.08	-0.195651639	-0.212897772
7.09	-0.194450431	-0.213283817
7.1	-0.193348057	-0.21356208
7.11	-0.192373901	-0.213736181
7.12	-0.191540004	-0.213812007
7.13	-0.190854069	-0.213796007
7.14	-0.1903238	-0.213694635
7.15	-0.189956902	-0.213514341
7.16	-0.189761079	-0.213261577
7.17	-0.189744035	-0.212942795
7.18	-0.189913474	-0.212564446
7.19	-0.1902771	-0.212132981
7.2	-0.190842618	-0.211654853
7.21	-0.191611142	-0.211135386
7.22	-0.192557428	-0.2105754
7.23	-0.193649643	-0.20997459
7.24	-0.194855953	-0.209332648
7.25	-0.196144526	-0.208649268
7.26	-0.197483526	-0.207924144
7.27	-0.198841122	-0.20715697
7.28	-0.20018548	-0.206347438
7.29	-0.201484765	-0.205495243
7.3	-0.202707146	-0.204600079
7.31	-0.203827845	-0.203661047
7.32	-0.204850315	-0.202674882
7.33	-0.205785064	-0.201637731
7.34	-0.206642603	-0.200545736
7.35	-0.207433441	-0.199395042
7.36	-0.208168087	-0.198181793
7.37	-0.208857049	-0.196902133
7.38	-0.209510839	-0.195552207
7.39	-0.210139964	-0.194128158
7.4	-0.210754935	-0.192626132
7.41	-0.211361596	-0.191043492
7.42	-0.211947134	-0.189382477

7.43	-0.212494073	-0.187646549
7.44	-0.212984936	-0.185839166
7.45	-0.213402245	-0.18396379
7.46	-0.213728524	-0.182023879
7.47	-0.213946295	-0.180022893
7.48	-0.214038082	-0.177964293
7.49	-0.213986407	-0.175851539
7.5	-0.213773794	-0.173688089
7.51	-0.213391371	-0.171478482
7.52	-0.212864685	-0.169231559
7.53	-0.21222789	-0.166957242
7.54	-0.21151514	-0.16466545
7.55	-0.210760588	-0.162366103
7.56	-0.209998386	-0.160069122
7.57	-0.209262688	-0.157784426
7.58	-0.208587647	-0.155521936
7.59	-0.208007417	-0.153291571
7.6	-0.207556151	-0.151103252
7.61	-0.2072532	-0.14896599
7.62	-0.207058712	-0.146885162
7.63	-0.206918032	-0.144865238
7.64	-0.206776505	-0.142910686
7.65	-0.206579476	-0.141025975
7.66	-0.206272291	-0.139215574
7.67	-0.205800295	-0.137483952
7.68	-0.205108834	-0.135835578
7.69	-0.204143252	-0.13427492
7.7	-0.202848896	-0.132806447
7.71	-0.201187585	-0.131432663
7.72	-0.199187037	-0.130148208
7.73	-0.196891446	-0.128945759
7.74	-0.194345003	-0.127817989
7.75	-0.191591903	-0.126757574
7.76	-0.188676337	-0.12575719
7.77	-0.185642498	-0.124809511
7.78	-0.182534579	-0.123907214
7.79	-0.179396773	-0.123042973
7.8	-0.176273273	-0.122209463
7.81	-0.173201271	-0.121399296
7.82	-0.170189958	-0.120604824
7.83	-0.167241525	-0.119818334
7.84	-0.164358162	-0.119032115
7.85	-0.16154206	-0.118238455
7.86	-0.15879541	-0.117429642
7.87	-0.156120401	-0.116597964
7.88	-0.153519226	-0.115735708
7.89	-0.150994074	-0.114835164
7.9	-0.148547136	-0.113888618

7.91	-0.146180019	-0.112889108
7.92	-0.143891995	-0.111832666
7.93	-0.141681751	-0.110716074
7.94	-0.139547977	-0.109536112
7.95	-0.13748936	-0.108289562
7.96	-0.135504589	-0.106973205
7.97	-0.133592351	-0.105583823
7.98	-0.131751335	-0.104118197
7.99	-0.129980229	-0.102573107
8	-0.128277721	-0.100945336
8.01	-0.12663136	-0.099231594
8.02	-0.124984139	-0.097428315
8.03	-0.123267913	-0.09553186
8.04	-0.121414535	-0.093538592
8.05	-0.119355858	-0.091444874
8.06	-0.117023738	-0.089247068
8.07	-0.114350028	-0.086941536
8.08	-0.111266582	-0.084524642
8.09	-0.107705253	-0.081992748
8.1	-0.103597897	-0.079342216
8.11	-0.098888607	-0.076567008
8.12	-0.09357044	-0.073651481
8.13	-0.087648694	-0.070577592
8.14	-0.081128666	-0.067327298
8.15	-0.074015652	-0.063882555
8.16	-0.066314951	-0.06022532
8.17	-0.058031859	-0.056337549
8.18	-0.049171674	-0.052201199
8.19	-0.039739693	-0.047798226
8.2	-0.029741213	-0.043110588
8.21	-0.019198258	-0.038125605
8.22	-0.008199756	-0.032852058
8.23	0.00314864	-0.027304094
8.24	0.014741275	-0.021495857
8.25	0.026472495	-0.015441495
8.26	0.038236647	-0.009155152
8.27	0.049928076	-0.002650976
8.28	0.061441128	0.004259734
8.29	0.072670149	0.011502013
8.3	0.083509486	0.018928458
8.31	0.093872616	0.026526426
8.32	0.103749544	0.034292112
8.33	0.113149409	0.04222392
8.34	0.122081348	0.050320254
8.35	0.130554499	0.058579517
8.36	0.138577999	0.067000115
8.37	0.146160985	0.07558045
8.38	0.153312597	0.084318928

8.39	0.16004197	0.093213952
8.4	0.166358243	0.102263926
8.41	0.172278656	0.111460698
8.42	0.177852856	0.12076989
8.43	0.183138596	0.130150566
8.44	0.188193625	0.139561792
8.45	0.193075694	0.148962633
8.46	0.197842554	0.158312153
8.47	0.202551956	0.167569418
8.48	0.20726165	0.176693492
8.49	0.212029388	0.185643441
8.5	0.216912919	0.19437833
8.51	0.221968745	0.202869139
8.52	0.227248371	0.211134509
8.53	0.232802049	0.219204996
8.54	0.238680035	0.227111156
8.55	0.244932584	0.234883546
8.56	0.251609948	0.242552722
8.57	0.258762384	0.25014924
8.58	0.266440144	0.257703655
8.59	0.274693484	0.265246525
8.6	0.283572658	0.272808405
8.61	0.293120041	0.280414283
8.62	0.303346493	0.288066872
8.63	0.314254995	0.295763316
8.64	0.325848527	0.303500758
8.65	0.338130069	0.311276344
8.66	0.351102604	0.319087216
8.67	0.36476911	0.326930519
8.68	0.379132569	0.334803398
8.69	0.394195962	0.342702995
8.7	0.409962269	0.350626456
8.71	0.426410867	0.358573733
8.72	0.443426717	0.36655601
8.73	0.460871178	0.374587282
8.74	0.478605606	0.382681543
8.75	0.49649136	0.390852786
8.76	0.514389798	0.399115005
8.77	0.532162276	0.407482194
8.78	0.549670152	0.415968346
8.79	0.566774785	0.424587456
8.8	0.583337532	0.433353517
8.81	0.59923935	0.442273394
8.82	0.614439594	0.451325441
8.83	0.62891722	0.460480884
8.84	0.642651183	0.469710947
8.85	0.655620437	0.478986856
8.86	0.667803937	0.488279837

8.87	0.679180639	0.497561113
8.88	0.689729497	0.506801912
8.89	0.699429467	0.515973458
8.9	0.708259503	0.525046976
8.91	0.71620664	0.533998292
8.92	0.723290233	0.54282163
8.93	0.729537716	0.551515815
8.94	0.734976524	0.560079671
8.95	0.739634091	0.568512024
8.96	0.743537852	0.576811698
8.97	0.74671524	0.584977517
8.98	0.749193691	0.593008307
8.99	0.75100064	0.600902891
9	0.752163519	0.608660095
9.01	0.752713219	0.616278354
9.02	0.752694444	0.623754549
9.03	0.752155355	0.631085172
9.04	0.751144111	0.638266715
9.05	0.749708873	0.645295669
9.06	0.7478978	0.652168527
9.07	0.745759052	0.65888178
9.08	0.743340789	0.665431921
9.09	0.740691171	0.67181544
9.1	0.737858357	0.678028831
9.11	0.734885295	0.684066625
9.12	0.731794081	0.689915519
9.13	0.728601598	0.69556025
9.14	0.725324729	0.700985555
9.15	0.721980357	0.706176171
9.16	0.718585367	0.711116835
9.17	0.71515664	0.715792285
9.18	0.71171106	0.720187256
9.19	0.70826551	0.724286487
9.2	0.704836874	0.728074714
9.21	0.701434075	0.731535735
9.22	0.698034196	0.734649587
9.23	0.69460636	0.73739537
9.24	0.691119692	0.739752181
9.25	0.687543314	0.74169912
9.26	0.68384635	0.743215284
9.27	0.679997923	0.744279772
9.28	0.675967156	0.744871682
9.29	0.671723174	0.744970113
9.3	0.667235099	0.744554164
9.31	0.662474682	0.743606547
9.32	0.657424185	0.742124437
9.33	0.652068494	0.740108621
9.34	0.646392497	0.737559889

9.35	0.640381083	0.734479028
9.36	0.63401914	0.730866827
9.37	0.627291555	0.726724075
9.38	0.620183216	0.72205156
9.39	0.612679011	0.71685007
9.4	0.604763829	0.711120394
9.41	0.596426905	0.704865241
9.42	0.587674871	0.698095002
9.43	0.578518706	0.690821987
9.44	0.56896939	0.683058507
9.45	0.559037903	0.674816875
9.46	0.548735223	0.666109399
9.47	0.538072331	0.656948393
9.48	0.527060206	0.647346166
9.49	0.515709828	0.637315029
9.5	0.504032176	0.626867294
9.51	0.492024043	0.61601319
9.52	0.479625475	0.604754612
9.53	0.46676233	0.593091377
9.54	0.453360467	0.581023298
9.55	0.439345746	0.56855019
9.56	0.424644023	0.555671868
9.57	0.40918116	0.542388146
9.58	0.392883013	0.52869884
9.59	0.375675443	0.514603763
9.6	0.357484307	0.50010273
9.61	0.338279507	0.48519459
9.62	0.318207114	0.469874333
9.63	0.297457242	0.454135981
9.64	0.276220003	0.437973557
9.65	0.254685511	0.421381086
9.66	0.23304388	0.404352589
9.67	0.211485222	0.38688209
9.68	0.190199652	0.368963613
9.69	0.169377283	0.350591181
9.7	0.149208228	0.331758817
9.71	0.129855601	0.31247251
9.72	0.111374519	0.292786114
9.73	0.093793099	0.272765451
9.74	0.077139458	0.252476339
9.75	0.061441713	0.2319846
9.76	0.046727982	0.211356055
9.77	0.033026382	0.190656522
9.78	0.02036503	0.169951823
9.79	0.008772043	0.149307778
9.8	-0.001724462	0.128790207
9.81	-0.011115095	0.108454367
9.82	-0.019465378	0.088313253

9.83	-0.026859561	0.068369299
9.84	-0.033381893	0.048624937
9.85	-0.039116625	0.0290826
9.86	-0.044148005	0.00974472
9.87	-0.048560283	-0.008939305
9.88	-0.052437709	-0.026959941
9.89	-0.055864534	-0.044778907
9.9	-0.058925005	-0.062393884
9.91	-0.061707486	-0.079792829
9.92	-0.064316786	-0.096924786
9.93	-0.066861828	-0.11372907
9.94	-0.069451535	-0.130144996
9.95	-0.072194828	-0.146111882
9.96	-0.075200632	-0.161569042
9.97	-0.078577867	-0.176455793
9.98	-0.082435458	-0.19071145
9.99	-0.086882325	-0.204275329
10	-0.092027392	-0.217086747
10.01	-0.097953974	-0.229105807
10.02	-0.104642958	-0.240375776
10.03	-0.112049623	-0.250960706
10.04	-0.12012925	-0.260924652
10.05	-0.128837117	-0.270331667
10.06	-0.138128504	-0.279245806
10.07	-0.147958691	-0.287731122
10.08	-0.158282957	-0.29585167
10.09	-0.169056582	-0.303671503
10.1	-0.180234845	-0.311254676
10.11	-0.191771703	-0.318649835
10.12	-0.20361582	-0.325844003
10.13	-0.215714536	-0.332808796
10.14	-0.228015191	-0.339515828
10.15	-0.240465126	-0.345936717
10.16	-0.25301168	-0.352043077
10.17	-0.265602196	-0.357806524
10.18	-0.278184012	-0.363198674
10.19	-0.29070447	-0.368191142
10.2	-0.30311091	-0.372755544
10.21	-0.315351048	-0.376876586
10.22	-0.327374101	-0.380591332
10.23	-0.339129665	-0.383949938
10.24	-0.350567333	-0.387002557
10.25	-0.361636699	-0.389799345
10.26	-0.372287357	-0.392390457
10.27	-0.382468901	-0.394826047
10.28	-0.392130924	-0.39715627
10.29	-0.401223021	-0.399431281
10.3	-0.409694786	-0.401701234

10.31	-0.417505103	-0.404004246
10.32	-0.42465002	-0.40633028
10.33	-0.431134876	-0.408657261
10.34	-0.436965008	-0.410963112
10.35	-0.442145756	-0.413225757
10.36	-0.446682457	-0.415423123
10.37	-0.450580451	-0.417533132
10.38	-0.453845076	-0.419533709
10.39	-0.456481669	-0.421402779
10.4	-0.45849557	-0.423118265
10.41	-0.459898699	-0.424664872
10.42	-0.460729306	-0.426054417
10.43	-0.461032222	-0.427305496
10.44	-0.460852279	-0.428436707
10.45	-0.460234308	-0.429466645
10.46	-0.459223141	-0.430413908
10.47	-0.457863609	-0.431297092
10.48	-0.456200545	-0.432134793
10.49	-0.454278779	-0.432945609
10.5	-0.452143144	-0.433748135
10.51	-0.449834259	-0.434557284
10.52	-0.447375901	-0.435373224
10.53	-0.444787633	-0.43619244
10.54	-0.442089019	-0.437011416
10.55	-0.439299623	-0.437826636
10.56	-0.43643901	-0.438634585
10.57	-0.433526744	-0.439431747
10.58	-0.430582389	-0.440214606
10.59	-0.427625508	-0.440979647
10.6	-0.424675667	-0.441723353
10.61	-0.421747991	-0.442440994
10.62	-0.418839854	-0.443122985
10.63	-0.415944194	-0.443758522
10.64	-0.413053945	-0.444336805
10.65	-0.410162044	-0.444847033
10.66	-0.407261427	-0.445278404
10.67	-0.404345031	-0.445620118
10.68	-0.401405792	-0.445861371
10.69	-0.398436645	-0.445991365
10.7	-0.395430528	-0.445999296
10.71	-0.392378828	-0.445872004
10.72	-0.389266743	-0.445586887
10.73	-0.386077921	-0.445118983
10.74	-0.382796012	-0.444443331
10.75	-0.379404664	-0.443534968
10.76	-0.375887527	-0.442368934
10.77	-0.37222825	-0.440920265
10.78	-0.368410482	-0.439164001

10.79	-0.364417871	-0.43707518
10.8	-0.360234068	-0.43462884
10.81	-0.355852845	-0.431803312
10.82	-0.351308471	-0.428590101
10.83	-0.34664534	-0.424984004
10.84	-0.341907845	-0.420979817
10.85	-0.337140379	-0.416572339
10.86	-0.332387336	-0.411756366
10.87	-0.327693109	-0.406526695
10.88	-0.323102092	-0.400878125
10.89	-0.318658678	-0.394805452
10.9	-0.314407259	-0.388303473
10.91	-0.310377242	-0.381373474
10.92	-0.306538079	-0.37404269
10.93	-0.302844235	-0.366344846
10.94	-0.299250174	-0.358313664
10.95	-0.295710362	-0.349982868
10.96	-0.292179262	-0.341386183
10.97	-0.288611341	-0.332557332
10.98	-0.284961062	-0.323530039
10.99	-0.28118289	-0.314338027
11	-0.27723129	-0.30501502
11.01	-0.273070313	-0.295592239
11.02	-0.268702358	-0.286090893
11.03	-0.26413941	-0.276529688
11.04	-0.259393452	-0.266927331
11.05	-0.254476471	-0.257302527
11.06	-0.249400451	-0.247673984
11.07	-0.244177377	-0.238060406
11.08	-0.238819233	-0.2284805
11.09	-0.233338006	-0.218952974
11.1	-0.227745679	-0.209496531
11.11	-0.222052666	-0.200128185
11.12	-0.216263091	-0.190858169
11.13	-0.210379509	-0.181695021
11.14	-0.204404471	-0.17264728
11.15	-0.198340532	-0.163723484
11.16	-0.192190244	-0.154932173
11.17	-0.18595616	-0.146281885
11.18	-0.179640835	-0.137781159
11.19	-0.17324682	-0.129438533
11.2	-0.166776669	-0.121262546
11.21	-0.160229615	-0.113257137
11.22	-0.15359161	-0.105407843
11.23	-0.146845283	-0.097695603
11.24	-0.139973268	-0.090101353
11.25	-0.132958194	-0.082606032
11.26	-0.125782693	-0.075190576

11.27	-0.118429396	-0.067835925
11.28	-0.110880934	-0.060523015
11.29	-0.103119938	-0.053232783
11.3	-0.09512904	-0.045946169
11.31	-0.086896912	-0.038647474
11.32	-0.078436396	-0.031334466
11.33	-0.069766374	-0.024008276
11.34	-0.060905731	-0.016670037
11.35	-0.051873349	-0.00932088
11.36	-0.042688111	-0.001961939
11.37	-0.0333689	0.005675939
11.38	-0.023934601	0.013419811
11.39	-0.014404095	0.021170391
11.4	-0.004796266	0.028926491
11.41	0.004868877	0.036688782
11.42	0.014566825	0.044465377
11.43	0.024271943	0.052266252
11.44	0.033958595	0.060101378
11.45	0.043601147	0.067980729
11.46	0.053173963	0.07591428
11.47	0.062651409	0.083912004
11.48	0.072007849	0.091983874
11.49	0.081217648	0.100139865
11.5	0.090255172	0.10838995
11.51	0.099094203	0.116739543
11.52	0.107706196	0.125175825
11.53	0.116062023	0.133681416
11.54	0.124132558	0.142238937
11.55	0.131888673	0.15083101
11.56	0.13930124	0.159440255
11.57	0.146341132	0.168049293
11.58	0.152979223	0.176640745
11.59	0.159186383	0.185197232
11.6	0.164933487	0.193701374
11.61	0.170207228	0.202136119
11.62	0.17505759	0.210485713
11.63	0.179550374	0.21873473
11.64	0.183751386	0.226867741
11.65	0.18772643	0.234869321
11.66	0.191541308	0.242724041
11.67	0.195261826	0.250416474
11.68	0.198953786	0.257931193
11.69	0.202682993	0.265252772
11.7	0.206515251	0.272365782
11.71	0.210514797	0.279258843
11.72	0.214739602	0.285936763
11.73	0.219246073	0.292408396
11.74	0.224090614	0.298682596

11.75	0.229329631	0.304768218
11.76	0.235019528	0.310674117
11.77	0.241216712	0.316409147
11.78	0.247977587	0.321982161
11.79	0.255358559	0.327402015
11.8	0.263416033	0.332677563
11.81	0.27219073	0.337818626
11.82	0.281660631	0.342838896
11.83	0.291788035	0.347753031
11.84	0.302535239	0.352575689
11.85	0.31386454	0.357321529
11.86	0.325738235	0.36200521
11.87	0.338118623	0.366641389
11.88	0.350968001	0.371244725
11.89	0.364248666	0.375829877
11.9	0.377922915	0.380411503
11.91	0.391947194	0.385004787
11.92	0.406254533	0.389627013
11.93	0.420772112	0.394295992
11.94	0.435427109	0.399029535
11.95	0.450146702	0.403845452
11.96	0.464858071	0.408761552
11.97	0.479488395	0.413795647
11.98	0.49396485	0.418965547
11.99	0.508214618	0.424289062
12	0.522164875	0.429784002
12.01	0.535749198	0.43546136
12.02	0.548926754	0.441304854
12.03	0.561663107	0.447291386
12.04	0.57392382	0.453397857
12.05	0.585674457	0.459601166
12.06	0.596880582	0.465878215
12.07	0.60750776	0.472205906
12.08	0.617521553	0.478561138
12.09	0.626887527	0.484920813
12.1	0.635571244	0.491261831
12.11	0.643547125	0.497565798
12.12	0.65082501	0.503833135
12.13	0.657423598	0.510068967
12.14	0.663361586	0.516278419
12.15	0.668657672	0.522466617
12.16	0.673330552	0.528638687
12.17	0.677398924	0.534799753
12.18	0.680881486	0.540954942
12.19	0.683796935	0.547109378
12.2	0.686163968	0.553268186
12.21	0.688003488	0.559433689
12.22	0.689345216	0.565596993

12.23	0.690221081	0.571746397
12.24	0.690663008	0.577870205
12.25	0.690702926	0.583956719
12.26	0.69037276	0.589994238
12.27	0.689704439	0.595971067
12.28	0.688729889	0.601875505
12.29	0.687481038	0.607695856
12.3	0.685989812	0.61342042
12.31	0.684282371	0.61903687
12.32	0.682361803	0.62453036
12.33	0.680225429	0.629885415
12.34	0.677870569	0.635086559
12.35	0.675294544	0.640118317
12.36	0.672494674	0.644965215
12.37	0.669468281	0.649611776
12.38	0.666212684	0.654042526
12.39	0.662725204	0.658241989
12.4	0.659003162	0.66219469
12.41	0.655041894	0.665883329
12.42	0.650828797	0.669283304
12.43	0.646349285	0.672368191
12.44	0.64158877	0.675111564
12.45	0.636532666	0.677486995
12.46	0.631166386	0.679468061
12.47	0.625475343	0.681028335
12.48	0.619444949	0.68214139
12.49	0.613060619	0.682780803
12.5	0.606307765	0.682920146
12.51	0.59917649	0.682536212
12.52	0.591675659	0.68161867
12.53	0.583818827	0.680160406
12.54	0.575619546	0.678154307
12.55	0.567091373	0.675593259
12.56	0.558247861	0.672470149
12.57	0.549102565	0.668777864
12.58	0.539669039	0.66450929
12.59	0.529960838	0.659657313
12.6	0.519991516	0.654214822
12.61	0.509768715	0.648177899
12.62	0.499276426	0.641555427
12.63	0.488492728	0.634359483
12.64	0.4773957	0.626602147
12.65	0.465963419	0.618295496
12.66	0.454173966	0.60945161
12.67	0.442005417	0.600082568
12.68	0.429435853	0.590200448
12.69	0.416443352	0.57981733
12.7	0.403005992	0.568945291

12.71	0.389113544	0.557593826
12.72	0.374802545	0.54576209
12.73	0.360121225	0.533446656
12.74	0.345117812	0.520644093
12.75	0.329840538	0.507350973
12.76	0.314337629	0.493563867
12.77	0.298657317	0.479279346
12.78	0.28284783	0.464493982
12.79	0.266957398	0.449204344
12.8	0.25103425	0.433407005
12.81	0.235135804	0.417106228
12.82	0.219356237	0.400337049
12.83	0.203798913	0.383142197
12.84	0.188567196	0.365564401
12.85	0.173764453	0.34764639
12.86	0.159494047	0.329430891
12.87	0.145859343	0.310960636
12.88	0.132963707	0.292278351
12.89	0.120910503	0.273426766
12.9	0.109803096	0.254448609
12.91	0.099719361	0.235386445
12.92	0.09063521	0.216282173
12.93	0.082501068	0.19717753
12.94	0.075267357	0.178114251
12.95	0.068884499	0.159134071
12.96	0.063302919	0.140278726
12.97	0.058473039	0.121589952
12.98	0.054345283	0.103109483
12.99	0.050870072	0.084879056
13	0.047997831	0.066940406
13.01	0.045672579	0.04933195
13.02	0.043812722	0.032078825
13.03	0.042330265	0.015202852
13.04	0.041137209	-0.001213476
13.05	0.040145559	-0.016505106
13.06	0.039267318	-0.031375201
13.07	0.038414489	-0.045802983
13.08	0.037499075	-0.05976767
13.09	0.03643308	-0.073248481
13.1	0.035128507	-0.086224637
13.11	0.033516498	-0.098677002
13.12	0.031604754	-0.110593026
13.13	0.029420112	-0.121961804
13.14	0.026989411	-0.132772433
13.15	0.024339491	-0.143014007
13.16	0.02149719	-0.152675621
13.17	0.018489346	-0.161746372
13.18	0.015342799	-0.170215354

13.19	0.012084386	-0.178071664
13.2	0.008740948	-0.185304396
13.21	0.005328698	-0.191910542
13.22	0.001821354	-0.197918679
13.23	-0.00181799	-0.203365278
13.24	-0.005626241	-0.208286811
13.25	-0.009640306	-0.212719752
13.26	-0.013897091	-0.216700571
13.27	-0.018433501	-0.220265742
13.28	-0.023286445	-0.223451736
13.29	-0.028492829	-0.226295025
13.3	-0.034089558	-0.228832083
13.31	-0.040100098	-0.23109297
13.32	-0.046494146	-0.233082108
13.33	-0.053227959	-0.234797508
13.34	-0.060257792	-0.23623718
13.35	-0.067539901	-0.237399135
13.36	-0.075030542	-0.238281385
13.37	-0.082685972	-0.238881939
13.38	-0.090462445	-0.239198809
13.39	-0.098316219	-0.239230005
13.4	-0.106203548	-0.238973538
13.41	-0.11408552	-0.238434543
13.42	-0.121942543	-0.237646651
13.43	-0.129759858	-0.236650616
13.44	-0.137522703	-0.235487193
13.45	-0.14521632	-0.234197137
13.46	-0.152825946	-0.232821201
13.47	-0.160336822	-0.231400142
13.48	-0.167734188	-0.229974712
13.49	-0.175003284	-0.228585668
13.5	-0.182129348	-0.227273763
13.51	-0.189095475	-0.226072653
13.52	-0.195876172	-0.224987598
13.53	-0.202443803	-0.22401676
13.54	-0.208770728	-0.223158298
13.55	-0.21482931	-0.222410375
13.56	-0.220591912	-0.221771151
13.57	-0.226030895	-0.221238787
13.58	-0.231118621	-0.220811444
13.59	-0.235827453	-0.220487283
13.6	-0.240129752	-0.220264465
13.61	-0.24400402	-0.220141611
13.62	-0.247453317	-0.22011918
13.63	-0.250486842	-0.22019809
13.64	-0.253113792	-0.220379261
13.65	-0.255343368	-0.220663611
13.66	-0.257184768	-0.221052058

13.67	-0.258647191	-0.221545523
13.68	-0.259739836	-0.222144922
13.69	-0.260471902	-0.222851176
13.7	-0.260852587	-0.223665203
13.71	-0.260895199	-0.224587191
13.72	-0.260629478	-0.225614404
13.73	-0.260089273	-0.226743376
13.74	-0.259308432	-0.227970641
13.75	-0.258320802	-0.229292731
13.76	-0.257160234	-0.230706181
13.77	-0.255860574	-0.232207524
13.78	-0.254455672	-0.233793293
13.79	-0.252979376	-0.235460023
13.8	-0.251465534	-0.237204246
13.81	-0.249942973	-0.239020143
13.82	-0.248420433	-0.240892475
13.83	-0.246901632	-0.242803652
13.84	-0.245390289	-0.244736083
13.85	-0.24389012	-0.246672175
13.86	-0.242404846	-0.248594338
13.87	-0.240938183	-0.250484981
13.88	-0.239493851	-0.252326512
13.89	-0.238075566	-0.25410134
13.9	-0.236687048	-0.255791873
13.91	-0.235326822	-0.257381794
13.92	-0.233972642	-0.258859873
13.93	-0.232597071	-0.260216155
13.94	-0.231172671	-0.261440685
13.95	-0.229672004	-0.262523507
13.96	-0.228067632	-0.263454665
13.97	-0.226332117	-0.264224205
13.98	-0.224438021	-0.264822169
13.99	-0.222357906	-0.265238604
14	-0.220064335	-0.265463553
14.01	-0.217537406	-0.265486173
14.02	-0.214787362	-0.265292076
14.03	-0.211831987	-0.264865984
14.04	-0.208689059	-0.264192621
14.05	-0.205376361	-0.263256711
14.06	-0.201911674	-0.262042976
14.07	-0.198312777	-0.26053614
14.08	-0.194597453	-0.258720927
14.09	-0.190783483	-0.256582059
14.1	-0.186888646	-0.254104261
14.11	-0.182923471	-0.251276725
14.12	-0.178869474	-0.248106519
14.13	-0.174700914	-0.244605184
14.14	-0.170392054	-0.240784257

14.15	-0.165917156	-0.236655277
14.16	-0.16125048	-0.232229782
14.17	-0.156366289	-0.227519311
14.18	-0.151238843	-0.222535402
14.19	-0.145842405	-0.217289594
14.2	-0.140151236	-0.211793426
14.21	-0.134146741	-0.206056534
14.22	-0.127838902	-0.200080948
14.23	-0.121244844	-0.193866798
14.24	-0.114381692	-0.187414213
14.25	-0.107266572	-0.180723321
14.26	-0.09991661	-0.17379425
14.27	-0.09234893	-0.16662713
14.28	-0.084580658	-0.15922209
14.29	-0.076628919	-0.151579258
14.3	-0.068510839	-0.143698764
14.31	-0.060246834	-0.135584071
14.32	-0.051870484	-0.127251993
14.33	-0.04341866	-0.118722677
14.34	-0.034928234	-0.11001627
14.35	-0.026436076	-0.101152922
14.36	-0.017979057	-0.09215278
14.37	-0.009594048	-0.083035991
14.38	-0.001317921	-0.073822705
14.39	0.006812454	-0.064533068
14.4	0.014760205	-0.05518723
14.41	0.022487998	-0.045803093
14.42	0.029956646	-0.036389585
14.43	0.037126496	-0.026953387
14.44	0.043957899	-0.017501182
14.45	0.050411201	-0.008039651
14.46	0.056446753	0.000598299
14.47	0.062024902	0.004571555
14.48	0.067105998	0.008540308
14.49	0.071650389	0.012501751
14.5	0.075618424	0.016453078
14.51	0.078979897	0.020389992
14.52	0.081742381	0.024302239
14.53	0.083922897	0.028178074
14.54	0.085538462	0.032005752
14.55	0.086606095	0.035773529
14.56	0.087142817	0.039469661
14.57	0.087165645	0.043082402
14.58	0.086691599	0.046600008
14.59	0.085737698	0.050010734
14.6	0.084320961	0.053302836
14.61	0.08246515	0.056466091
14.62	0.080221003	0.059496359

14.63	0.077645999	0.062391022
14.64	0.074797618	0.065147462
14.65	0.071733343	0.067763062
14.66	0.068510652	0.070235203
14.67	0.065187026	0.072561268
14.68	0.061819945	0.074738639
14.69	0.05846689	0.076764697
14.7	0.055185341	0.078636825
14.71	0.052025112	0.080352667
14.72	0.049005351	0.081910922
14.73	0.046137537	0.083310549
14.74	0.043433154	0.084550509
14.75	0.04090368	0.085629762
14.76	0.038560598	0.086547269
14.77	0.036415388	0.08730199
14.78	0.034479532	0.087892885
14.79	0.032764509	0.088318915
14.8	0.031281802	0.088579039
14.81	0.030037422	0.088673587
14.82	0.029015504	0.088608357
14.83	0.028194716	0.088390516
14.84	0.027553724	0.088027229
14.85	0.027071195	0.087525664
14.86	0.026725794	0.086892986
14.87	0.02649619	0.086136363
14.88	0.026361048	0.085262961
14.89	0.026299035	0.084279947
14.9	0.026288818	0.083194486
14.91	0.02631173	0.082013312
14.92	0.026359773	0.080741418
14.93	0.026427615	0.079383367
14.94	0.026509925	0.077943717
14.95	0.026601371	0.07642703
14.96	0.026696622	0.074837867
14.97	0.026790346	0.073180788
14.98	0.026877211	0.071460353
14.99	0.026951886	0.069681124
15	0.02700904	0.067847661
15.01	0.027044812	0.06596421
15.02	0.027061228	0.064033765
15.03	0.027061783	0.062059004
15.04	0.027049975	0.060042606
15.05	0.0270293	0.057987251
15.06	0.027003254	0.055895616
15.07	0.026975333	0.053770381
15.08	0.026949036	0.051614225
15.09	0.026927857	0.049429827
15.1	0.026915293	0.047219865

15.11	0.026913976	0.044988034
15.12	0.026923076	0.042742095
15.13	0.026940897	0.040490821
15.14	0.026965746	0.038242987
15.15	0.026995928	0.036007369
15.16	0.027029746	0.033792743
15.17	0.027065508	0.031607882
15.18	0.027101517	0.029461562
15.19	0.027136078	0.027362558
15.2	0.027167498	0.025319645
15.21	0.027194139	0.023340595
15.22	0.027214598	0.021429166
15.23	0.027227531	0.019588111
15.24	0.027231593	0.017820187
15.25	0.027225438	0.016128146
15.26	0.027207723	0.014514744
15.27	0.027177101	0.012982736
15.28	0.02713223	0.011534875
15.29	0.027071763	0.010173915
15.3	0.026994356	0.008902613
15.31	0.026899511	0.007723461
15.32	0.026790118	0.00663791
15.33	0.026669912	0.00564715
15.34	0.02654263	0.004752372
15.35	0.026412009	0.003954766
15.36	0.026281784	0.003255521
15.37	0.026155692	0.002655829
15.38	0.02603747	0.002156879
15.39	0.025930852	0.001759861
15.4	0.025839577	0.001465966
15.41	0.02576619	0.001274776
15.42	0.02570848	0.001179444
15.43	0.025663046	0.001171516
15.44	0.025626486	0.001242537
15.45	0.0255954	0.001384052
15.46	0.025566386	0.001587607
15.47	0.025536044	0.001844747
15.48	0.025500971	0.002147018
15.49	0.025457767	0.002485964
15.5	0.025403031	0.002853132
15.51	0.025334538	0.003240881
15.52	0.02525477	0.003644824
15.53	0.025167387	0.004061391
15.54	0.025076046	0.00448701
15.55	0.024984406	0.00491811
15.56	0.024896126	0.005351118
15.57	0.024814866	0.005782464
15.58	0.024744283	0.006208575

15.59	0.024688036	0.006625881
15.6	0.024649784	0.007030809
15.61	0.024632096	0.00742033
15.62	0.024633178	0.007793585
15.63	0.024650147	0.008150254
15.64	0.024680121	0.008490022
15.65	0.024720216	0.008812568
15.66	0.024767549	0.009117577
15.67	0.024819236	0.009404729
15.68	0.024872396	0.009673707
15.69	0.024924143	0.009924193
15.7	0.024971596	0.010155869
15.71	0.025012458	0.010368534
15.72	0.025046782	0.010562452
15.73	0.025075209	0.010738005
15.74	0.025098378	0.010895573
15.75	0.025116929	0.011035538
15.76	0.025131502	0.01115828
15.77	0.025142737	0.01126418
15.78	0.025151275	0.011353621
15.79	0.025157755	0.011426981
15.8	0.025162818	0.011484644
15.81	0.025167076	0.011527203
15.82	0.025171033	0.011556112
15.83	0.025175168	0.011573037
15.84	0.025179957	0.011579646
15.85	0.025185877	0.011577604
15.86	0.025193405	0.01156858
15.87	0.025203019	0.01155424
15.88	0.025215197	0.01153625
15.89	0.025230415	0.011516278
15.9	0.02524915	0.01149599
15.91	0.025271872	0.011476816
15.92	0.025299018	0.011459238
15.93	0.025331018	0.011443498
15.94	0.0253683	0.011429842
15.95	0.025411294	0.011418512
15.96	0.02546043	0.011409752
15.97	0.025516136	0.011403806
15.98	0.025578841	0.011400919
15.99	0.025648976	0.011401333
16	0.02572697	0.011405293
16.01	0.025812821	0.011412985
16.02	0.025904805	0.011424365
16.03	0.026000768	0.011439332
16.04	0.026098556	0.011457785
16.05	0.026196014	0.011479622
16.06	0.026290989	0.011504742

16.07	0.026381325	0.011533043
16.08	0.026464868	0.011564425
16.09	0.026539464	0.011598785
16.1	0.026602959	0.011636024
16.11	0.026653869	0.011676011
16.12	0.026693397	0.011718509
16.13	0.026723416	0.011763254
16.14	0.026745799	0.011809979
16.15	0.02676242	0.01185842
16.16	0.026775152	0.01190831
16.17	0.026785868	0.011959386
16.18	0.026796442	0.012011382
16.19	0.026808746	0.012064033
16.2	0.026824655	0.012117073
16.21	0.026845571	0.012170277
16.22	0.026871016	0.012223578
16.23	0.026900041	0.012276947
16.24	0.026931696	0.012330359
16.25	0.026965033	0.012383785
16.26	0.026999103	0.012437198
16.27	0.027032958	0.01249057
16.28	0.027065647	0.012543874
16.29	0.027096222	0.012597082
16.3	0.027123735	0.012650167
16.31	0.027147372	0.012703064
16.32	0.027166862	0.012755561
16.33	0.027182072	0.012807409
16.34	0.027192867	0.012858357
16.35	0.027199112	0.012908157
16.36	0.027200673	0.012956559
16.37	0.027197416	0.013003313
16.38	0.027189207	0.013048171
16.39	0.02717591	0.013090882
16.4	0.027157391	0.013131198
16.41	0.027133859	0.013168945
16.42	0.027106892	0.01320426
16.43	0.02707841	0.013237353
16.44	0.027050336	0.013268439
16.45	0.027024589	0.013297728
16.46	0.027003091	0.013325432
16.47	0.026987763	0.013351765
16.48	0.026980526	0.013376939
16.49	0.0269833	0.013401165
16.5	0.026998007	0.013424655
16.51	0.027025983	0.01344757
16.52	0.027066228	0.013469851
16.53	0.027117157	0.01349139
16.54	0.027177184	0.013512078

16.55	0.027244724	0.013531803
16.56	0.027318193	0.013550457
16.57	0.027396005	0.01356793
16.58	0.027476575	0.013584112
16.59	0.027558319	0.013598894
16.6	0.02763965	0.013612166
16.61	0.027719235	0.013623865
16.62	0.02779674	0.013634123
16.63	0.027872084	0.013643117
16.64	0.027945182	0.013651026
16.65	0.028015954	0.013658027
16.66	0.028084316	0.013664299
16.67	0.028150185	0.01367002
16.68	0.02821348	0.013675368
16.69	0.028274117	0.013680521
16.7	0.028332014	0.013685657
16.71	0.028387035	0.013690909
16.72	0.028438831	0.013696227
16.73	0.028486998	0.013701516
16.74	0.028531134	0.01370668
16.75	0.028570835	0.013711625
16.76	0.028605699	0.013716255
16.77	0.028635322	0.013720475
16.78	0.028659301	0.01372419
16.79	0.028677234	0.013727304
16.8	0.028688717	0.013729723
16.81	0.028694077	0.013731428
16.82	0.028696558	0.01373271
16.83	0.028700134	0.013733937
16.84	0.028708779	0.013735476
16.85	0.028726466	0.013737696
16.86	0.028757169	0.013740964
16.87	0.028804863	0.013745649
16.88	0.02887352	0.013752117
16.89	0.028967115	0.013760736
16.9	0.029089621	0.013771876
16.91	0.029243028	0.013785807
16.92	0.029421385	0.01380242
16.93	0.029616759	0.013821508
16.94	0.029821215	0.013842867
16.95	0.03002682	0.013866289
16.96	0.030225638	0.013891569
16.97	0.030409736	0.013918502
16.98	0.030571179	0.013946882
16.99	0.030702033	0.013976502
17	0.030794364	0.014007156
17.01	0.030842515	0.014038657
17.02	0.030849937	0.014070883

17.03	0.03082236	0.014103729
17.04	0.030765513	0.014137092
17.05	0.030685125	0.014170868
17.06	0.030586925	0.014204952
17.07	0.030476642	0.014239241
17.08	0.030360006	0.01427363
17.09	0.030242746	0.014308015
17.1	0.03013059	0.014342292
17.11	0.030028033	0.014376326
17.12	0.029934631	0.014409859
17.13	0.029848705	0.014442602
17.14	0.029768575	0.014474265
17.15	0.029692563	0.014504559
17.16	0.029618989	0.014533196
17.17	0.029546174	0.014559885
17.18	0.029472439	0.014584338
17.19	0.029396105	0.014606265
17.2	0.029315493	0.014625378
17.21	0.029229331	0.01464144
17.22	0.029137976	0.014654431
17.23	0.029042191	0.014664383
17.24	0.028942741	0.01467133
17.25	0.028840391	0.014675303
17.26	0.028735903	0.014676335
17.27	0.028630042	0.014674459
17.28	0.028523572	0.014669708
17.29	0.028417258	0.014662114
17.3	0.028311862	0.01465171
17.31	0.028208116	0.014638528
17.32	0.028106614	0.014622597
17.33	0.028007917	0.014603946
17.34	0.027912586	0.014582603
17.35	0.027821183	0.014558597
17.36	0.027734268	0.014531956
17.37	0.027652402	0.01450271
17.38	0.027576147	0.014470887
17.39	0.027506063	0.014436515
17.4	0.027442711	0.014399622
17.41	0.027386198	0.014360205
17.42	0.02733481	0.014318121
17.43	0.02728638	0.014273197
17.44	0.02723874	0.014225256
17.45	0.027189721	0.014174125
17.46	0.027137157	0.014119628
17.47	0.027078878	0.014061591
17.48	0.027012718	0.013999839
17.49	0.026936508	0.013934197
17.5	0.02684808	0.01386449

17.51	0.026746042	0.013790634
17.52	0.026632104	0.013712909
17.53	0.026508752	0.013631684
17.54	0.026378471	0.01354733
17.55	0.026243747	0.013460217
17.56	0.026107064	0.013370716
17.57	0.02597091	0.013279195
17.58	0.025837769	0.013186026
17.59	0.025710126	0.013091579
17.6	0.025590468	0.012996223
17.61	0.025480699	0.012900282
17.62	0.025380399	0.012803895
17.63	0.025288569	0.012707151
17.64	0.025204209	0.01261014
17.65	0.025126317	0.012512955
17.66	0.025053894	0.012415686
17.67	0.024985939	0.012318424
17.68	0.024921453	0.012221258
17.69	0.024859434	0.012124281
17.7	0.024798884	0.012027583
17.71	0.024739023	0.011931299
17.72	0.024679962	0.01183575
17.73	0.024622034	0.011741299
17.74	0.024565571	0.011648309
17.75	0.024510906	0.011557146
17.76	0.02445837	0.011468173
17.77	0.024408297	0.011381755
17.78	0.024361019	0.011298256
17.79	0.024316869	0.011218039
17.8	0.024276179	0.011141469
17.81	0.024239153	0.011068833
17.82	0.024205482	0.01100011
17.83	0.024174729	0.010935203
17.84	0.024146455	0.010874014
17.85	0.024120223	0.010816445
17.86	0.024095596	0.010762398
17.87	0.024072136	0.010711777
17.88	0.024049406	0.010664482
17.89	0.024026967	0.010620416
17.9	0.024004382	0.010579482
17.91	0.023981214	0.010541581
17.92	0.023957025	0.010506617
17.93	0.023931377	0.010474491
17.94	0.023903833	0.010445106
17.95	0.023873955	0.010418363
17.96	0.023841306	0.010394166
17.97	0.023805447	0.010372416
17.98	0.023765942	0.010353016

17.99	0.023722353	0.010335867
18	0.023674242	0.010320874

Table S14. Double Lane Change Test Result Data

Time	Actual Value	Estimated Value
0	-3.48E-08	-0.074207964
0.01	-0.006282505	-0.074207964
0.02	-0.009789487	-0.073898299
0.03	-0.011200866	-0.074207964
0.04	-0.010979545	-0.073878318
0.05	-0.009095302	-0.073001528
0.06	-0.006096908	-0.071662226
0.07	-0.002180465	-0.069957671
0.08	0.002420081	-0.068344843
0.09	0.007343314	-0.065256504
0.1	0.012541652	-0.061058093
0.11	0.017733077	-0.056035384
0.12	0.02285824	-0.047816059
0.13	0.027856786	-0.039781327
0.14	0.032637344	-0.03209262
0.15	0.037157334	-0.025035591
0.16	0.041431521	-0.018663812
0.17	0.045520949	-0.012744653
0.18	0.049389495	-0.007256453
0.19	0.053071292	-0.002180364
0.2	0.056553329	0.00273648
0.21	0.059893737	0.00771771
0.22	0.063121841	0.01021992
0.23	0.066164714	0.013394719
0.24	0.06903324	0.017328189
0.25	0.071704802	0.022249068
0.26	0.074154481	0.028151077
0.27	0.076376269	0.034895538
0.28	0.078338792	0.04231849
0.29	0.080040844	0.050292498
0.3	0.081392419	0.058723487
0.31	0.082487297	0.067442597
0.32	0.083270799	0.076278448
0.33	0.083786767	0.085117575
0.34	0.084034351	0.093768138
0.35	0.084065083	0.102014564
0.36	0.08390899	0.10972418

0.37	0.083521043	0.116675986
0.38	0.083034152	0.122876003
0.39	0.082291004	0.128345106
0.4	0.081445297	0.132819706
0.41	0.080505587	0.136113311
0.42	0.079433961	0.138248937
0.43	0.078245136	0.13928932
0.44	0.076953566	0.139284583
0.45	0.075533377	0.138334884
0.46	0.074069543	0.136484614
0.47	0.072498488	0.133793667
0.48	0.070796375	0.130299221
0.49	0.069016695	0.126069758
0.5	0.06728588	0.121152615
0.51	0.065433691	0.115623373
0.52	0.063606511	0.10955981
0.53	0.06178232	0.103010245
0.54	0.060032296	0.096059403
0.55	0.058405449	0.088827777
0.56	0.056847885	0.081367341
0.57	0.055354894	0.073751144
0.58	0.054034642	0.066069573
0.59	0.052842312	0.058365385
0.6	0.05172681	0.050763164
0.61	0.050783049	0.043399517
0.62	0.049901899	0.036353914
0.63	0.049156012	0.02972466
0.64	0.048423243	0.023574074
0.65	0.04781325	0.017850383
0.66	0.04724167	0.012590066
0.67	0.046699278	0.007782467
0.68	0.046235106	0.003465948
0.69	0.045845621	-0.000333065
0.7	0.045610103	-0.003606792
0.71	0.045481469	-0.006374526
0.72	0.045520377	-0.008640505
0.73	0.045657716	-0.010431132
0.74	0.046038321	-0.011759415
0.75	0.04659854	-0.012577978
0.76	0.047361653	-0.012867255
0.77	0.048357115	-0.012602733
0.78	0.049569725	-0.011806658
0.79	0.050916984	-0.010443644
0.8	0.052393805	-0.008490494
0.81	0.054082348	-0.005930032
0.82	0.055881239	-0.002744914
0.83	0.057775308	0.000992564
0.84	0.059693032	0.005190187

0.85	0.06168422	0.009764732
0.86	0.063626349	0.014678429
0.87	0.065489097	0.019891964
0.88	0.067343201	0.025368671
0.89	0.069105444	0.030992437
0.9	0.070733202	0.03672796
0.91	0.072336926	0.042528564
0.92	0.073801451	0.048333585
0.93	0.075191074	0.054286467
0.94	0.076373839	0.060432054
0.95	0.077484206	0.066765951
0.96	0.078479809	0.073246315
0.97	0.079355503	0.079786637
0.98	0.080106764	0.086330732
0.99	0.080718375	0.092857129
1	0.081252411	0.099251708
1.01	0.081673924	0.105418834
1.02	0.081994209	0.111310094
1.03	0.082224892	0.11676168
1.04	0.082385211	0.121720004
1.05	0.082501404	0.126160765
1.06	0.082499604	0.130060896
1.07	0.082387721	0.133445393
1.08	0.082230142	0.136303436
1.09	0.081954485	0.138626843
1.1	0.081513269	0.140430631
1.11	0.081009946	0.141743675
1.12	0.080344989	0.142520299
1.13	0.07956392	0.142718057
1.14	0.078622648	0.142327427
1.15	0.077556995	0.141331858
1.16	0.076342358	0.139722962
1.17	0.074961953	0.137478607
1.18	0.073454448	0.134598402
1.19	0.071803284	0.131098867
1.2	0.069979689	0.12698003
1.21	0.068044414	0.122223681
1.22	0.066000152	0.116833369
1.23	0.063830098	0.110800575
1.24	0.061547644	0.104081027
1.25	0.059113791	0.0967088
1.26	0.056637501	0.088680869
1.27	0.053904297	0.080055761
1.28	0.051105186	0.070798326
1.29	0.048148376	0.060898094
1.3	0.045088391	0.050416522
1.31	0.041892099	0.039443899
1.32	0.03852215	0.028095098

1.33	0.035107317	0.016670637
1.34	0.031536135	0.005324141
1.35	0.027880348	-0.005766803
1.36	0.024093967	-0.016362162
1.37	0.020296215	-0.026392694
1.38	0.016450125	-0.03551665
1.39	0.01252915	-0.043570147
1.4	0.008616397	-0.050480263
1.41	0.004620892	-0.056115736
1.42	0.000648732	-0.060610259
1.43	-0.003379634	-0.064451282
1.44	-0.007472357	-0.067613219
1.45	-0.011557618	-0.070199462
1.46	-0.015682631	-0.072448449
1.47	-0.019896412	-0.074465695
1.48	-0.02423703	-0.076439638
1.49	-0.028733296	-0.078511826
1.5	-0.033447791	-0.080812446
1.51	-0.038437605	-0.083545427
1.52	-0.043729546	-0.086586582
1.53	-0.049339115	-0.08968119
1.54	-0.055322383	-0.092920136
1.55	-0.061707413	-0.096425651
1.56	-0.068502635	-0.100213152
1.57	-0.075741066	-0.104297082
1.58	-0.083439039	-0.108786147
1.59	-0.09158511	-0.113710106
1.6	-0.100170798	-0.119046621
1.61	-0.109202075	-0.124768378
1.62	-0.118628629	-0.130888257
1.63	-0.128422576	-0.137357722
1.64	-0.138564713	-0.144138042
1.65	-0.149008521	-0.151135257
1.66	-0.159698776	-0.158501486
1.67	-0.170590861	-0.166199832
1.68	-0.181636926	-0.174169464
1.69	-0.192782768	-0.18233308
1.7	-0.20397254	-0.190652094
1.71	-0.2151764	-0.199276418
1.72	-0.226369592	-0.208273118
1.73	-0.237519893	-0.217663394
1.74	-0.248641945	-0.227454184
1.75	-0.259725446	-0.237592971
1.76	-0.270764719	-0.24784105
1.77	-0.281765114	-0.258175728
1.78	-0.292733376	-0.268599699
1.79	-0.303663787	-0.279122137
1.8	-0.314549263	-0.289758504

1.81	-0.325410315	-0.300328889
1.82	-0.336246029	-0.310765698
1.83	-0.347061371	-0.32107131
1.84	-0.357848846	-0.331268999
1.85	-0.36861481	-0.341422611
1.86	-0.379356131	-0.351534634
1.87	-0.390039155	-0.361607161
1.88	-0.400689183	-0.371640278
1.89	-0.411281467	-0.381633074
1.9	-0.421804032	-0.391584155
1.91	-0.432273004	-0.401495548
1.92	-0.44272792	-0.411366643
1.93	-0.453160921	-0.421283378
1.94	-0.463600691	-0.431259189
1.95	-0.474083678	-0.44130124
1.96	-0.484654221	-0.4514207
1.97	-0.495295449	-0.461627646
1.98	-0.505996759	-0.471934549
1.99	-0.516809058	-0.482357086
2	-0.527768361	-0.492912068
2.01	-0.538872215	-0.503611536
2.02	-0.550050467	-0.514468713
2.03	-0.561329449	-0.525410072
2.04	-0.572733501	-0.536431686
2.05	-0.584190705	-0.547532882
2.06	-0.595654474	-0.558711331
2.07	-0.607182508	-0.569962911
2.08	-0.618730381	-0.581282811
2.09	-0.630240106	-0.592661211
2.1	-0.641681256	-0.604080897
2.11	-0.653008303	-0.615522837
2.12	-0.664183758	-0.626961424
2.13	-0.67511426	-0.638361849
2.14	-0.685737729	-0.649689041
2.15	-0.696047954	-0.660907534
2.16	-0.705966761	-0.671974592
2.17	-0.715435486	-0.682847186
2.18	-0.724427499	-0.693481427
2.19	-0.732915483	-0.703830085
2.2	-0.740872762	-0.713851299
2.21	-0.748280589	-0.723503347
2.22	-0.755126614	-0.732747617
2.23	-0.761396718	-0.741554053
2.24	-0.767059159	-0.74989025
2.25	-0.77209436	-0.757725264
2.26	-0.776499685	-0.765029538
2.27	-0.780169391	-0.771778465
2.28	-0.783223568	-0.777937044

2.29	-0.785433494	-0.783480391
2.3	-0.78687929	-0.788371775
2.31	-0.787428256	-0.792575853
2.32	-0.787014395	-0.796055633
2.33	-0.785726237	-0.798767618
2.34	-0.783311125	-0.800673591
2.35	-0.779890044	-0.801725611
2.36	-0.77529685	-0.801889088
2.37	-0.769598575	-0.801112438
2.38	-0.762736116	-0.799365596
2.39	-0.754703959	-0.796606798
2.4	-0.745609064	-0.792717206
2.41	-0.735377961	-0.787676365
2.42	-0.724032937	-0.781449633
2.43	-0.711389597	-0.774022944
2.44	-0.697649871	-0.765400217
2.45	-0.682638737	-0.755594525
2.46	-0.666529094	-0.744585431
2.47	-0.649295331	-0.732379254
2.48	-0.630965989	-0.71896015
2.49	-0.611570687	-0.704319049
2.5	-0.591066057	-0.688416938
2.51	-0.569422888	-0.671071164
2.52	-0.546604132	-0.652273096
2.53	-0.522553492	-0.631999001
2.54	-0.497335859	-0.610217291
2.55	-0.471025703	-0.586905753
2.56	-0.443608636	-0.562065774
2.57	-0.41501094	-0.535711227
2.58	-0.385378341	-0.507892263
2.59	-0.354644731	-0.478622847
2.6	-0.322801943	-0.448035061
2.61	-0.289994119	-0.416324122
2.62	-0.256403009	-0.383223427
2.63	-0.222097667	-0.348933373
2.64	-0.18725571	-0.313743781
2.65	-0.151975185	-0.277658018
2.66	-0.116294695	-0.240965015
2.67	-0.080261851	-0.203710667
2.68	-0.044003169	-0.165415204
2.69	-0.007605958	-0.126805447
2.7	0.028842889	-0.088395056
2.71	0.065237182	-0.050280226
2.72	0.10143253	-0.014208487
2.73	0.13747215	0.019085522
2.74	0.173299232	0.050579463
2.75	0.208884145	0.084909174
2.76	0.244242855	0.12449088

2.77	0.279358212	0.168691191
2.78	0.314175664	0.215288592
2.79	0.348700657	0.266398299
2.8	0.383021125	0.316196645
2.81	0.417186361	0.36442771
2.82	0.451223692	0.413257433
2.83	0.485124644	0.462920462
2.84	0.51892537	0.512565187
2.85	0.552635566	0.558228634
2.86	0.586174882	0.59776476
2.87	0.619586669	0.631385354
2.88	0.652874342	0.660872808
2.89	0.686047182	0.684457666
2.9	0.719036873	0.708305428
2.91	0.751809863	0.732653407
2.92	0.784307653	0.756650283
2.93	0.816417115	0.780814512
2.94	0.84805987	0.805133325
2.95	0.879166027	0.828757223
2.96	0.909587499	0.851513965
2.97	0.93926217	0.873697958
2.98	0.968141377	0.895120987
2.99	0.996206013	0.915973832
3	1.023357038	0.936231885
3.01	1.049533836	0.955825818
3.02	1.07471386	0.974697398
3.03	1.098856084	0.992797277
3.04	1.12193207	1.010076096
3.05	1.143827466	1.026484705
3.06	1.164387746	1.041898416
3.07	1.183470496	1.056175713
3.08	1.200757997	1.06923462
3.09	1.216456732	1.080980386
3.1	1.230013634	1.091302799
3.11	1.241429804	1.100077189
3.12	1.250342316	1.1071657
3.13	1.256745255	1.112426602
3.14	1.260523365	1.115717333
3.15	1.261445164	1.116901818
3.16	1.259588835	1.115962998
3.17	1.255107716	1.112949581
3.18	1.247998534	1.10789307
3.19	1.238368245	1.100796716
3.2	1.226369251	1.091711287
3.21	1.212072896	1.08067781
3.22	1.195318251	1.067769439
3.23	1.176395255	1.053078752
3.24	1.155316799	1.036736531

3.25	1.132405786	1.018895812
3.26	1.107707227	0.999688145
3.27	1.081373473	0.979226583
3.28	1.053653891	0.957598689
3.29	1.024733796	0.934937043
3.3	0.994750229	0.911431231
3.31	0.963915206	0.887649216
3.32	0.932389798	0.863455102
3.33	0.900363052	0.839143654
3.34	0.867994073	0.814932628
3.35	0.835403572	0.790969114
3.36	0.802769999	0.767356617
3.37	0.770268578	0.744179406
3.38	0.738041222	0.721519781
3.39	0.706313306	0.699478575
3.4	0.675266108	0.678004682
3.41	0.645021394	0.656761547
3.42	0.615789065	0.636142978
3.43	0.587704041	0.616105443
3.44	0.560847841	0.596654522
3.45	0.535315783	0.577856082
3.46	0.51128997	0.559817953
3.47	0.488948123	0.542650758
3.48	0.468295565	0.526452885
3.49	0.449427411	0.511341757
3.5	0.432552787	0.497545933
3.51	0.417686292	0.485098695
3.52	0.404783416	0.473923804
3.53	0.393816619	0.46404833
3.54	0.384709153	0.455419754
3.55	0.377330449	0.448043806
3.56	0.371464235	0.441903621
3.57	0.366952181	0.437053523
3.58	0.363749883	0.433486764
3.59	0.361677315	0.431061885
3.6	0.360582353	0.429463279
3.61	0.360398048	0.42892932
3.62	0.360948839	0.429452144
3.63	0.362150558	0.430909376
3.64	0.363964136	0.433253236
3.65	0.366298651	0.436365168
3.66	0.369099508	0.440139765
3.67	0.372419857	0.444402135
3.68	0.376324898	0.449020036
3.69	0.380767357	0.45398364
3.7	0.385794874	0.45933597
3.71	0.391360702	0.464772314
3.72	0.397570754	0.470312226

3.73	0.404367539	0.475964982
3.74	0.411506376	0.481741446
3.75	0.419117623	0.487658572
3.76	0.427102422	0.493707946
3.77	0.43522633	0.499861975
3.78	0.443439186	0.506105213
3.79	0.451639304	0.512380555
3.8	0.459562571	0.518681337
3.81	0.467025284	0.524916912
3.82	0.47398543	0.530980326
3.83	0.480253066	0.536766405
3.84	0.485664014	0.542168489
3.85	0.490308683	0.547066604
3.86	0.493830091	0.551367931
3.87	0.496386128	0.554984795
3.88	0.49787933	0.557828657
3.89	0.498233824	0.559819845
3.9	0.497455017	0.560844905
3.91	0.495454907	0.560855983
3.92	0.492232505	0.559822206
3.93	0.487807673	0.55772609
3.94	0.482154283	0.554557827
3.95	0.47528482	0.550332945
3.96	0.467357015	0.545075454
3.97	0.458344763	0.5388161
3.98	0.448420933	0.53162126
3.99	0.437784811	0.523575931
4	0.4264962	0.514838734
4.01	0.414734046	0.50553123
4.02	0.402683573	0.495780096
4.03	0.390436532	0.485775687
4.04	0.378143335	0.47564764
4.05	0.365959738	0.465523503
4.06	0.354035716	0.455531847
4.07	0.342549291	0.445596035
4.08	0.331616233	0.435837977
4.09	0.321440758	0.426391456
4.1	0.312220621	0.417385976
4.11	0.304020587	0.408937237
4.12	0.297013316	0.401160603
4.13	0.291335266	0.394113902
4.14	0.287049315	0.38790614
4.15	0.28416499	0.382649098
4.16	0.282898058	0.378441397
4.17	0.283177509	0.375537377
4.18	0.285022476	0.374236955
4.19	0.288445401	0.374582761
4.2	0.29343666	0.376576097

4.21	0.299866973	0.380185388
4.22	0.307802613	0.385381659
4.23	0.317107252	0.392128118
4.24	0.327762433	0.400368864
4.25	0.339658338	0.410032916
4.26	0.352776679	0.42103148
4.27	0.366958955	0.43332426
4.28	0.382244972	0.446559103
4.29	0.398250413	0.460561864
4.3	0.415173755	0.475029684
4.31	0.432662691	0.489873182
4.32	0.450766827	0.504974142
4.33	0.469416568	0.520216517
4.34	0.488335576	0.535484871
4.35	0.507588254	0.550687063
4.36	0.527121706	0.565733707
4.37	0.546899753	0.580619574
4.38	0.566871812	0.595397653
4.39	0.587030764	0.610093082
4.4	0.607388872	0.624882497
4.41	0.627893806	0.639754826
4.42	0.648448325	0.654700647
4.43	0.669064846	0.669718921
4.44	0.689706722	0.684822373
4.45	0.710315364	0.699994132
4.46	0.730853392	0.715236846
4.47	0.751243948	0.730454119
4.48	0.771348169	0.745516257
4.49	0.791111349	0.760368395
4.5	0.810404037	0.774953643
4.51	0.829056094	0.789207321
4.52	0.847018691	0.803067176
4.53	0.864232563	0.816457792
4.54	0.880608565	0.829309466
4.55	0.896096632	0.841552051
4.56	0.91057489	0.853099503
4.57	0.923908019	0.863816483
4.58	0.936085684	0.873589545
4.59	0.947012401	0.882340848
4.6	0.956521409	0.890007189
4.61	0.964626863	0.896537705
4.62	0.971337476	0.901889978
4.63	0.976676644	0.906014009
4.64	0.980307798	0.908860027
4.65	0.982419526	0.910375551
4.66	0.982698555	0.910557344
4.67	0.981081105	0.909412973
4.68	0.977603079	0.906941244

4.69	0.972158261	0.903120818
4.7	0.964797217	0.897950241
4.71	0.955670763	0.891423908
4.72	0.944780829	0.883558257
4.73	0.932330134	0.874385868
4.74	0.918275311	0.863946105
4.75	0.902761237	0.852288066
4.76	0.885792454	0.839429841
4.77	0.867361909	0.825435283
4.78	0.847569104	0.810378688
4.79	0.826397797	0.794326053
4.8	0.803837876	0.777327947
4.81	0.779996581	0.759430016
4.82	0.75474969	0.741004089
4.83	0.728280104	0.721861588
4.84	0.700686126	0.702083814
4.85	0.671942063	0.681878546
4.86	0.642152968	0.66135768
4.87	0.611277259	0.640543511
4.88	0.579262699	0.619438326
4.89	0.54611413	0.598051759
4.9	0.511872934	0.576367788
4.91	0.476483895	0.554251196
4.92	0.439984743	0.531317071
4.93	0.402607582	0.507738876
4.94	0.364373769	0.483232051
4.95	0.325435675	0.457665695
4.96	0.28610028	0.430646979
4.97	0.246561279	0.401986701
4.98	0.20704307	0.372182997
4.99	0.167763707	0.341542214
5	0.129050196	0.309783647
5.01	0.091167878	0.277753401
5.02	0.054348264	0.245705114
5.03	0.018897554	0.212991375
5.04	-0.01496924	0.177569508
5.05	-0.047129889	0.139079466
5.06	-0.077466046	0.098217867
5.07	-0.105936579	0.056400911
5.08	-0.132536134	0.012828948
5.09	-0.157354978	-0.031284464
5.1	-0.180405275	-0.075596038
5.11	-0.201780675	-0.1194607
5.12	-0.221652408	-0.162806722
5.13	-0.240152934	-0.202643937
5.14	-0.257427068	-0.236783402
5.15	-0.273707473	-0.264609507
5.16	-0.289177246	-0.286924677

5.17	-0.303988816	-0.305161132
5.18	-0.318336894	-0.319048045
5.19	-0.332411474	-0.330859635
5.2	-0.34632757	-0.340192469
5.21	-0.360196276	-0.348280893
5.22	-0.374144134	-0.35536757
5.23	-0.388264585	-0.363719784
5.24	-0.402588312	-0.373228137
5.25	-0.417154091	-0.38417182
5.26	-0.432004688	-0.396115461
5.27	-0.447093516	-0.408815124
5.28	-0.462366182	-0.42219023
5.29	-0.477838229	-0.435432316
5.3	-0.493475382	-0.448932174
5.31	-0.509262471	-0.462745069
5.32	-0.525188156	-0.476853408
5.33	-0.541251953	-0.491235392
5.34	-0.557498247	-0.505868658
5.35	-0.573912828	-0.52073198
5.36	-0.59053357	-0.53581013
5.37	-0.607416205	-0.551097108
5.38	-0.624572	-0.566596336
5.39	-0.64207806	-0.582322659
5.4	-0.659959135	-0.598319425
5.41	-0.678257769	-0.61466163
5.42	-0.697033383	-0.63137754
5.43	-0.716263162	-0.648492783
5.44	-0.735977992	-0.666032741
5.45	-0.756182782	-0.68402288
5.46	-0.776872339	-0.702483798
5.47	-0.798062046	-0.72143453
5.48	-0.819710608	-0.740882805
5.49	-0.841700154	-0.760827511
5.5	-0.863986382	-0.781232395
5.51	-0.886438328	-0.802016422
5.52	-0.908877657	-0.823138036
5.53	-0.931216932	-0.844541114
5.54	-0.953288481	-0.866154416
5.55	-0.974954402	-0.887901703
5.56	-0.996159649	-0.909696456
5.57	-1.016753875	-0.931443743
5.58	-1.036711229	-0.953049235
5.59	-1.055907503	-0.974407118
5.6	-1.074147667	-0.995407933
5.61	-1.09140271	-1.01594701
5.62	-1.107538464	-1.035912129
5.63	-1.122442763	-1.055195791
5.64	-1.136042005	-1.07369741

5.65	-1.148228123	-1.091304278
5.66	-1.158863393	-1.10791463
5.67	-1.167919371	-1.123413241
5.68	-1.175121664	-1.137690016
5.69	-1.180658201	-1.150638125
5.7	-1.184281531	-1.162160627
5.71	-1.185986667	-1.172173227
5.72	-1.185984288	-1.180605631
5.73	-1.184010641	-1.187416255
5.74	-1.18058161	-1.192556359
5.75	-1.175269403	-1.196013492
5.76	-1.168374904	-1.197757344
5.77	-1.159869464	-1.197798242
5.78	-1.149802843	-1.196124699
5.79	-1.138141047	-1.192754917
5.8	-1.124962119	-1.187701919
5.81	-1.110126293	-1.180970803
5.82	-1.093703558	-1.172656909
5.83	-1.075456464	-1.16260882
5.84	-1.055606335	-1.150804821
5.85	-1.033794275	-1.137232199
5.86	-1.010348118	-1.121874728
5.87	-0.985191542	-1.104738701
5.88	-0.958563124	-1.085850063
5.89	-0.930537243	-1.06522352
5.9	-0.901058487	-1.042791167
5.91	-0.870127032	-1.018221946
5.92	-0.837761346	-0.991434864
5.93	-0.803848758	-0.962572024
5.94	-0.768446765	-0.931668035
5.95	-0.731690068	-0.898743697
5.96	-0.693671451	-0.863867916
5.97	-0.654579377	-0.827100211
5.98	-0.614577002	-0.788516939
5.99	-0.57378644	-0.748266436
6	-0.532348217	-0.706555452
6.01	-0.490428979	-0.663847309
6.02	-0.448122896	-0.620285412
6.03	-0.405603018	-0.576029095
6.04	-0.363003594	-0.531047555
6.05	-0.320498611	-0.485362146
6.06	-0.278306432	-0.439333725
6.07	-0.236585303	-0.393364022
6.08	-0.195509701	-0.347738801
6.09	-0.155280827	-0.302797536
6.1	-0.116062244	-0.258696309
6.11	-0.077999441	-0.214784832
6.12	-0.04130245	-0.171896417

6.13	-0.00615314	-0.130496827
6.14	0.027328616	-0.090541078
6.15	0.059056445	-0.052866508
6.16	0.088942645	-0.018380346
6.17	0.116952684	0.012348016
6.18	0.143045199	0.039692531
6.19	0.167231687	0.066001017
6.2	0.189491276	0.094469722
6.21	0.209817461	0.125352943
6.22	0.228167137	0.156994819
6.23	0.244511895	0.189547367
6.24	0.258838137	0.223516281
6.25	0.271120984	0.255543967
6.26	0.281366505	0.28690741
6.27	0.289614448	0.319310746
6.28	0.295913494	0.350593792
6.29	0.300294521	0.378838566
6.3	0.302827592	0.401104767
6.31	0.303565632	0.415989098
6.32	0.302611589	0.425866484
6.33	0.300080062	0.431044741
6.34	0.296091828	0.430726794
6.35	0.290751958	0.428727522
6.36	0.284205931	0.424850153
6.37	0.27670105	0.41821352
6.38	0.268318174	0.41074648
6.39	0.259182638	0.402354349
6.4	0.249619953	0.392790919
6.41	0.239601775	0.382725269
6.42	0.229284728	0.372278165
6.43	0.218871587	0.361470474
6.44	0.208334863	0.350397742
6.45	0.197863344	0.339393462
6.46	0.187404692	0.328550743
6.47	0.177034425	0.317960815
6.48	0.166793908	0.307717727
6.49	0.156663076	0.297856262
6.5	0.146728289	0.28854417
6.51	0.136926077	0.27964618
6.52	0.127270778	0.271061831
6.53	0.117706274	0.262647706
6.54	0.108276697	0.254451654
6.55	0.099006219	0.246198055
6.56	0.089786699	0.237854236
6.57	0.080745684	0.229415517
6.58	0.071873597	0.220144801
6.59	0.063130803	0.209467367
6.6	0.054593724	0.196571644

6.61	0.04622951	0.181008425
6.62	0.038143204	0.162707135
6.63	0.030332618	0.142225681
6.64	0.022898771	0.119352782
6.65	0.015766856	0.094699959
6.66	0.008941503	0.068962176
6.67	0.002583658	0.042290392
6.68	-0.003477123	0.016102882
6.69	-0.009096731	-0.008684614
6.7	-0.014390687	-0.031207324
6.71	-0.019327935	-0.050469501
6.72	-0.023934484	-0.066112002
6.73	-0.028242788	-0.078570367
6.74	-0.032294186	-0.087781263
6.75	-0.036068627	-0.094171019
6.76	-0.039569474	-0.098272143
6.77	-0.04284482	-0.100218594
6.78	-0.045871512	-0.100714375
6.79	-0.048631349	-0.100150026
6.8	-0.051179963	-0.098599414
6.81	-0.053485662	-0.096705182
6.82	-0.055524703	-0.094695068
6.83	-0.057320752	-0.092566868
6.84	-0.058834505	-0.090243964
6.85	-0.060078776	-0.087706054
6.86	-0.061047111	-0.085092924
6.87	-0.061698745	-0.082507725
6.88	-0.062151324	-0.080026898
6.89	-0.062334402	-0.07761461
6.9	-0.062304287	-0.075292307
6.91	-0.062107733	-0.073139349
6.92	-0.061748636	-0.071153684
6.93	-0.061259949	-0.0693558
6.94	-0.060672217	-0.067792128
6.95	-0.059991067	-0.066460213
6.96	-0.059231779	-0.065368849
6.97	-0.058411176	-0.064444995
6.98	-0.057512922	-0.063608493
6.99	-0.056628351	-0.062851938
7	-0.055692483	-0.062178134
7.01	-0.054754115	-0.061487261
7.02	-0.053842451	-0.060770012
7.03	-0.052910542	-0.060016811
7.04	-0.052060987	-0.05920597
7.05	-0.051226309	-0.058352126
7.06	-0.050414534	-0.057466383
7.07	-0.049666349	-0.056568355
7.08	-0.048997387	-0.055665096

7.09	-0.048370432	-0.054782671
7.1	-0.047768299	-0.053928318
7.11	-0.04719712	-0.053103649
7.12	-0.046684287	-0.05231674
7.13	-0.046231699	-0.051563422
7.14	-0.045736327	-0.050844056
7.15	-0.045342836	-0.050158904
7.16	-0.044952218	-0.04951175
7.17	-0.044643663	-0.048895867
7.18	-0.044294392	-0.048317955
7.19	-0.044035606	-0.047769405
7.2	-0.043735952	-0.047258516
7.21	-0.043551271	-0.046788838
7.22	-0.043422367	-0.046358966
7.23	-0.043311661	-0.045977635
7.24	-0.043257578	-0.045648601
7.25	-0.043264994	-0.04538611
7.26	-0.04333933	-0.045199779
7.27	-0.043471543	-0.045103017
7.28	-0.043662399	-0.045096109
7.29	-0.043908023	-0.045187383
7.3	-0.044193816	-0.045372455
7.31	-0.044528243	-0.045645807
7.32	-0.044900002	-0.046004256
7.33	-0.04528915	-0.046438288
7.34	-0.045700208	-0.046932914
7.35	-0.046087945	-0.04747409
7.36	-0.046550905	-0.048038379
7.37	-0.046977529	-0.048607795
7.38	-0.047371632	-0.049166708
7.39	-0.047742622	-0.049705035
7.4	-0.048109733	-0.050205932
7.41	-0.048430962	-0.050666111
7.42	-0.048712387	-0.051073588
7.43	-0.048982184	-0.051424299
7.44	-0.049209625	-0.051721785
7.45	-0.049406799	-0.05195512
7.46	-0.049532896	-0.052129475
7.47	-0.049684595	-0.052245817
7.48	-0.049799259	-0.052309407
7.49	-0.049846937	-0.052357086
7.5	-0.049900988	-0.052385601
